# Reprogramming RiPP scaffolds through skeletal editing unlocks chemical space

**DOI:** 10.1039/d6sc03088b

**Published:** 2026-05-26

**Authors:** Hiroshige Ogawa, Longhui Yu, Shangzhao Li, Yuuya Nagata, Tsz Ki Chan, Yudai Matsuda, Jing Liu, Yong-Xin Li, Hugh Nakamura

**Affiliations:** a The Hong Kong University of Science and Technology Clear Water Bay Hong Kong SAR China hnakamura@ust.hk; b Autonomous Polymer Design and Discovery Group Research Center for Macromolecules and Biomaterials, National Institute for Materials Science (NIMS) 1-2-1 Sengen Tsukuba Ibaraki 305-0047 Japan; c City University of Hong Kong Tat Chee Avenue Kowloon Hong Kong SAR China; d Department of Chemistry and The Swire Institute of Marine Science, The University of Hong Kong Hong Kong SAR China

## Abstract

In this study, we report (1) a scalable, systematic, and general synthetic approach for the supply of ribosomally synthesized and post-translationally modified peptides (RiPPs) bearing Tyr–Trp cross-linkages, and (2) the comprehensive expansion of novel chemical space through their skeletal diversification. In recent years, numerous biaryl-containing peptides have been discovered, and some of these RiPPs exhibit potent biological activities. However, despite the high metabolic stability and strong target protein binding generally attributed to biaryl RiPPs, their significant strain and rigidity have limited the availability of general synthetic methods. Here, we demonstrate the high versatility of modular synthetic strategies for the construction of RiPPs and achieve the synthesis of a variety of RiPPs containing Tyr–Trp cross-linkages. Furthermore, skeletal diversification *via* scaffold hopping enables access to artificial RiPP scaffolds incorporating quinazoline and quinoline motifs, whose preparation has previously been challenging.

## Abstract 2

We herein report the expansion of chemical space achieved through skeletal editing at a late stage of the synthesis of biaryl-containing RiPPs.

## Graphical abstract

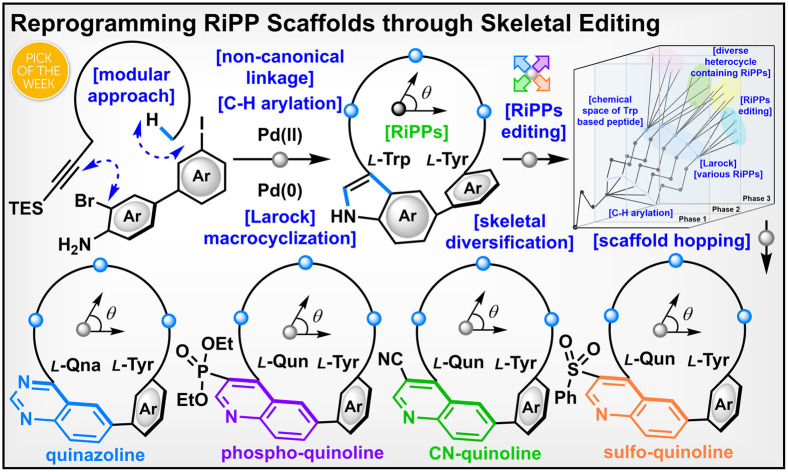

## Introduction

**Fig. 1:**
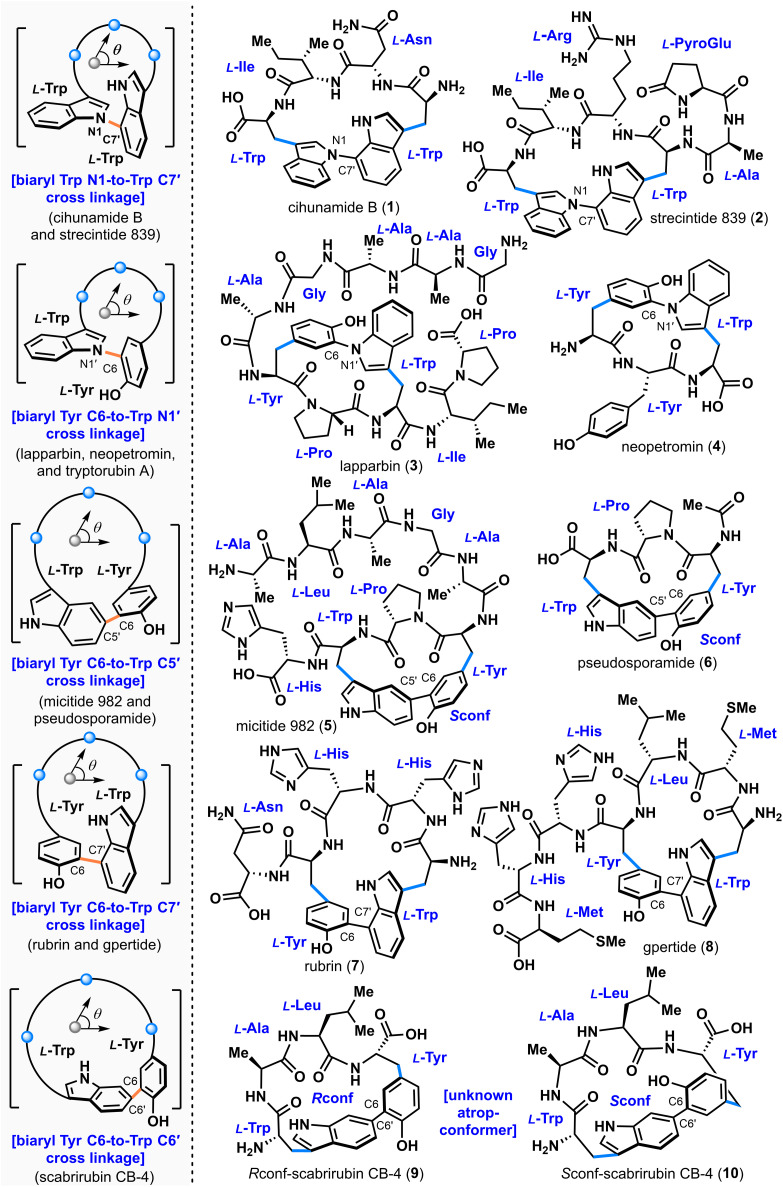
Representative noncanonical biaryl cyclic peptides.

Advances in genome mining technologies, which enable the discovery of previously overlooked but potentially valuable genes from biological genomes, have led in recent years to the identification of numerous ribosomally synthesized and post-translationally modified peptides (RiPPs) that were not recognized before.^1–6^ Because the core scaffolds of RiPPs can, to a large extent, be predicted from their genomic sequences, the number of newly reported RiPPs has been increasing at an explosive pace worldwide.^7–10^ Among them are noncanonical cyclic peptides bearing rigid biaryl motifs embedded within their macrocyclic frameworks (Fig. 1).^6,11–18^ Compared with conventional cyclic peptides, these rigid cyclic peptides are characterized by enhanced stability in biological systems and reduced susceptibility to proteolytic degradation.^19–22^ In addition, their conformational rigidity is considered to promote stronger binding to target proteins than that achieved by more flexible cyclic peptides, thereby conferring higher selectivity.^23–25^ Some of these noncanonical cyclic peptides also exhibit potent biological activities, including antimicrobial properties.^26–34^

Despite their promise as attractive and potentially valuable drug discovery seeds, general and scalable synthetic routes to such rigid peptides remain limited to date.^9,12,14,35–38^ For example, enzymatic production often affords only several to several tens of milligrams of the final products, which is disadvantageous in terms of scalability and the preparation of analogues for further activity optimization.^9,12,35,37,38^ In the case of purely chemical synthesis, conventional macrocyclization methods are frequently not applicable to these rigid noncanonical cyclic peptides.^39–43^ For instance, it has been shown that the Trp *N*1-to-Trp *C*7′ cross-linkage present in cihunamide B (1)^44^ and strecintide 839 (2)^45^ cannot be constructed by classical intramolecular (or intermolecular) Ullmann couplings using CuI, which represent a standard approach to biaryl macrocyclization. Likewise, the Tyr *C*6-to-Trp *N*1′ cross-linkage found in lapparbin (3)^46^ is not accessible *via* intramolecular Ullmann coupling. Furthermore, the highly strained cyclic framework of micitide 982 (5), which contains a Tyr *C*6-to-Trp *C*5′ cross-linkage, has been shown to be inaccessible by conventional macrocyclization using standard amide coupling reagents such as HATU, EDCI, PyBOP, or COMU.^47^ Thus, even though medium-sized molecules are attracting increased attention as a new modality in drug discovery and the importance of cyclic peptides has been growing, robust, scalable, and generally applicable synthetic methods for these rigid noncanonical cyclic peptides—particularly those that enable systematic analog synthesis—have yet to be established.

**Fig. 2:**
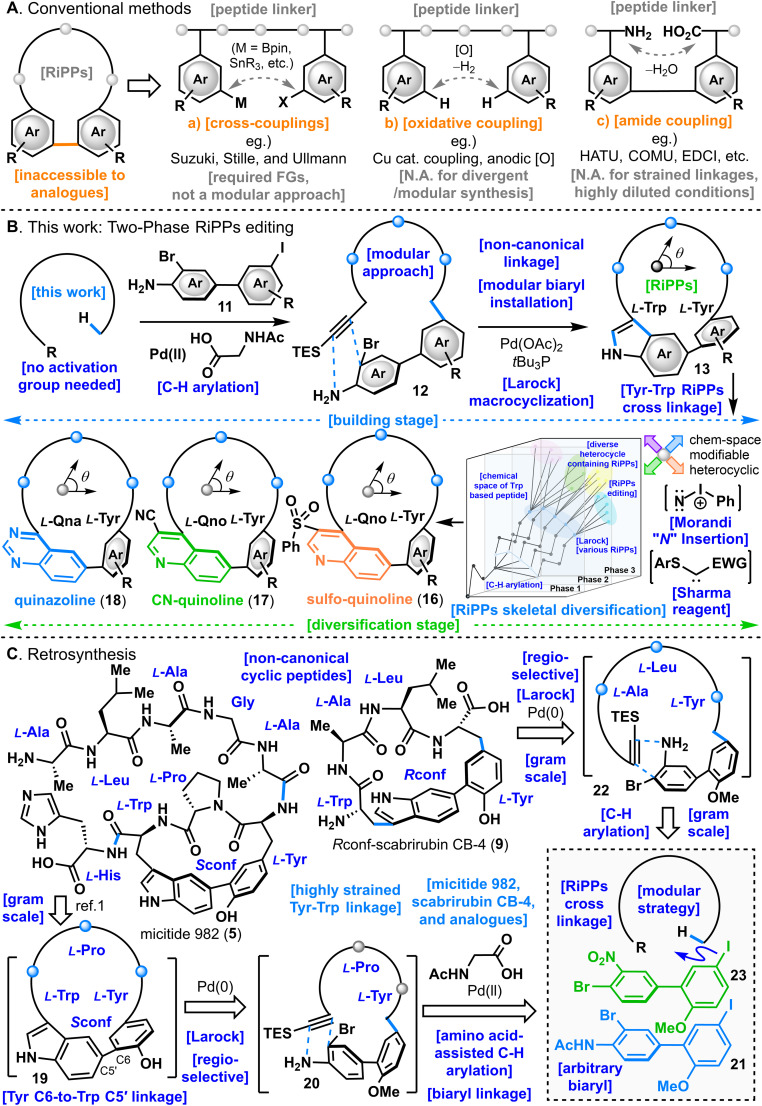
(A) Conventional methods to construct RiPPs skeletons. (B) Concept of this work. (C) Retrosynthetic analyses of micitide 982 (5) and scabrirubin CB-4 (9).

Conventional synthetic approaches to cyclic peptides bearing biaryl architectures are illustrated in Fig. 2A. Among the most reliable methods for achieving sp^2^–sp^2^ coupling, Suzuki–Miyaura^48–50^ and Stille couplings^51^ represent highly effective reactions for constructing biaryl linkages. However, these strategies require prior, separate functionalization of both aromatic rings (*e.g.*, halogenation, borylation, or stannylation). This prerequisite limits the modularity of the synthesis, as diverse biaryl motifs cannot be introduced at late stages, rendering these methods unsuitable for analog generation. In contrast, oxidative coupling, which directly connects two aromatic rings without the need for prefunctionalization, offers practical advantages for the efficient synthesis of natural products and specific compounds.^36^ Nevertheless, oxidative coupling is strongly dependent on the electronic density of the aromatic substrates, restricting its applicability to particular amino acid units and thereby limiting its utility in analog synthesis. Furthermore, the inability to freely introduce biaryl units at advanced stages of synthesis reduces the modularity of this approach. Intramolecular amide bond formation for macrocyclization has long been one of the most representative methods for cyclic peptide synthesis.^39–43^ However, when rigid, linear biaryl units are present in the substrate, the associated strain may prevent intramolecular amide coupling from proceeding.^47,52,53^ Even when macrocyclization is successful, extremely dilute conditions are often required, posing challenges for analog synthesis and scalability.

Beyond these established strategies, there exists a chemical space comprising molecular entities that are theoretically accessible but remain practically restricted due to the lack of generalized synthetic routes.^32,54–66^ Some of these molecular assemblies may exhibit biological activities or mechanisms of action distinct from known natural products or pharmaceuticals, potentially contributing to novel therapeutic modalities. In recent years, global efforts have focused on developing new molecular transformation strategies, including skeletal editing and scaffold rearrangements, to efficiently access such chemical space.^67–80^ Expansion of this space has enabled entry into molecular classes that were previously difficult to synthesize.

Our group has recently contributed to the development of general, modular synthetic methods for rigid noncanonical cyclic peptides, exemplified by RiPPs such as cihunamide B (1),^44^ strecintide 839 (2),^45^ lapparbin (3),^46^ neopetromin (4),^81^ and micitide 982 (5).^47^ These methods allow gram-scale supply of noncanonical cyclic peptides but rely on Larock macrocyclization, which has limited applicability to natural scaffolds, particularly beyond indole-derived frameworks. To overcome this limitation, we envisioned a two-phase strategy: in the first stage, biaryl-containing RiPP scaffolds would be assembled using a modular and scalable approach; in the second stage, skeletal diversification would be applied to edit the indole framework, thereby granting access to previously inaccessible regions of chemical space (Fig. 2B).

As demonstrated in our recent synthesis of lapparbin (3),^46^ biaryl motifs can be introduced into amino acid units *via* C–H arylation, followed by Pd(0)-catalyzed Larock macrocyclization to generate Tyr–Trp RiPP cross-linkages at an early stage. Subsequent application of skeletal diversification strategies, such as those recently reported by Morandi^82–84^ and Sharma^85,86^ for the synthesis of quinazolines and quinolines, is expected to expand RiPP chemical space into previously inaccessible domains. The hallmark of this approach lies in the early-stage construction of RiPP scaffolds using modular, scalable methods, followed by skeletal diversification to furnish diverse quinazoline and quinoline derivatives from common intermediates, thereby broadening chemical space.

Guided by this concept, we targeted micitide 982 (5)^18^ and scabrirubin CB-4 (9, 10),^14^ both Tyr–Trp cross-linked RiPPs, for chemical space expansion through a two-phase sequence combining C–H arylation and skeletal diversification (Fig. 2C). Scabrirubin CB-4 (9, 10) is a non-natural atropo-peptide recently generated by combinatorial biosynthesis.^14^ Its atropisomeric configuration has not yet been reported. In this study, we planned the synthesis of scabrirubin CB-4 (9, 10) to determine its atropisomeric arrangement. Scabrirubin CB-4 (9, 10) was expected to arise from cyclization precursor 22*via* regioselective Larock macrocyclization. Precursor 22 would be prepared by introducing biaryl derivative 23 into an amino acid unit through carboxylic acid-directed C–H arylation. A key advantage of this synthetic strategy is its modularity, enabling comprehensive access to RiPPs bearing diverse biaryl units through analogous approaches. For example, micitide 982 (5) was planned to be synthesized from biaryl derivative 21, introduced into an amino acid unit *via* carboxylic acid-directed C–H arylation to furnish precursor 20. Precursor 20 would then undergo regioselective Larock macrocyclization, followed by side-chain elaboration, to yield micitide 982 (5). Furthermore, both scabrirubin CB-4 (9, 10) and micitide 982 (5) are expected to serve as platforms for skeletal diversification, enabling expansion into RiPP derivatives bearing quinazoline or quinoline frameworks—chemical spaces that have previously been difficult to access. Overall, this synthetic strategy represents a versatile, modular supply method applicable not only to scabrirubin CB-4 (9, 10) and micitide 982 (5) but also to a wide range of RiPPs.

## Results and discussion

**Scheme 1:**
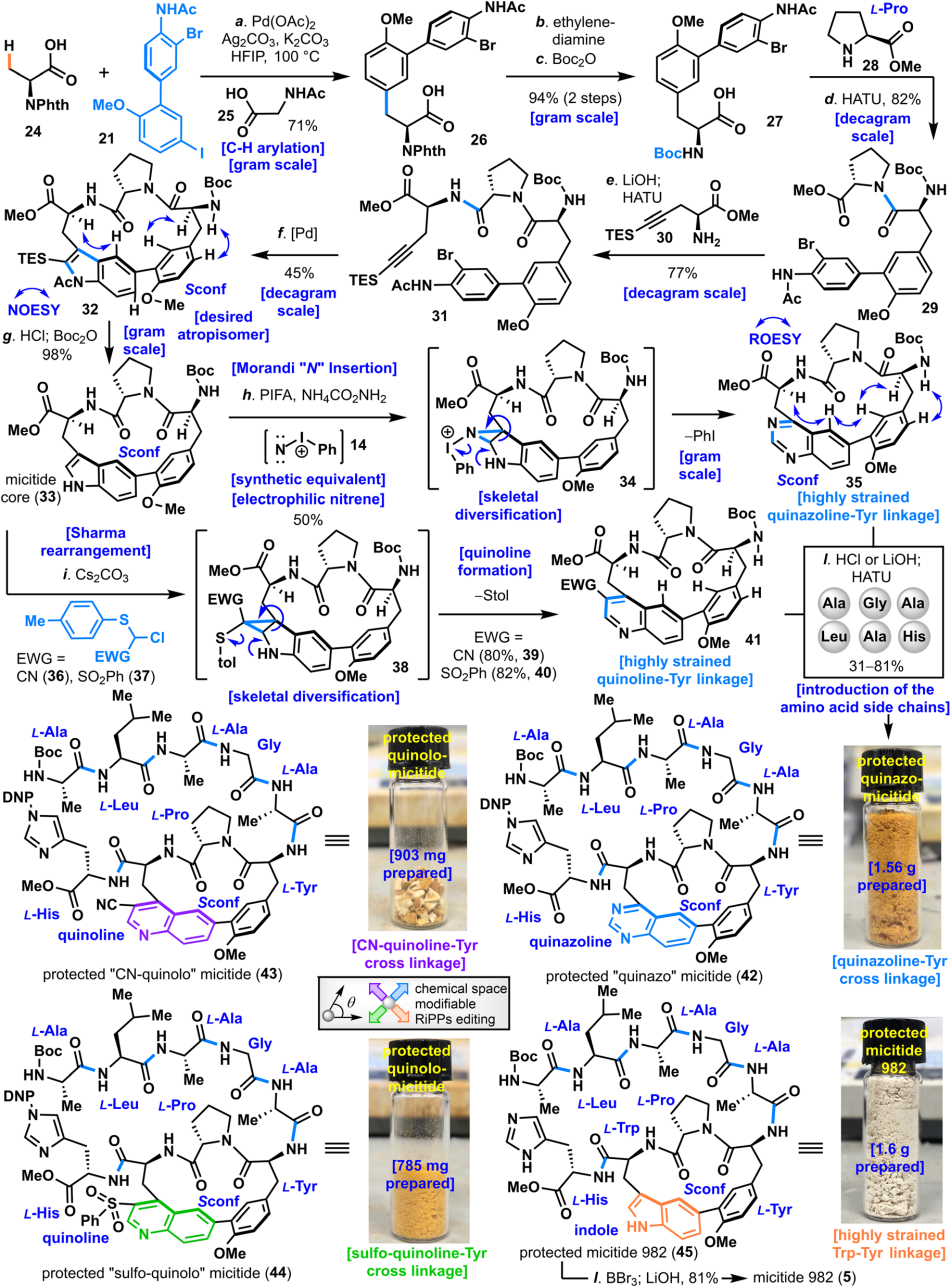
Total synthesis of micitide 982 (5) and its skeletal diversification. ^a^For detailed reagents and conditions, see the SI.

The total synthesis of micitide 982 (5) commenced with the C–H arylation of biaryl unit 21 and alanine derivative 24 (Scheme 1). Our group has recently reported an electrochemical approach to the synthesis of micitide 982 (5).^47^ In that method, C–C bond formation between a serine-derived alkyl bromide and a biaryl unit required Ni-catalysis, necessitating prior activation of the serine alcohol moiety through conversion into the corresponding alkyl bromide derivative. However, introduction of the biaryl fragment into the amino acid unit *via* Ni-catalyzed electrochemical C–C bond formation was hampered by competing side reactions such as reduction, elimination, and dimerization of the alkyl bromide precursor, limiting the yield to a maximum of 47%.

By contrast, C–H arylation provides a direct means of introducing aromatic units into amino acid residues without the need for prior activation. Numerous C–H arylation reactions have been reported.^87–94^ However, many of these methods require a directing group at the *C*–terminus, typically an aminoquinoline.^95–107^ For application to total synthesis, removal of the directing group is required, which entails multistep manipulations and suffers from low overall yields.^104,106,108–112^ Initially, C–H activation of an *N*–phthaloyl alanine derivative bearing an aminoquinoline at the C–terminus with biaryl 23 was attempted. Although the C–H activation proceeded in good yield, subsequent protecting-group manipulation required three steps, and the overall yield was not satisfactory (25%), highlighting the limited practicality of this approach (see SI). Thus, the development of a more practical methodology was essential.

More recently, carboxylic acid-free C–H arylations of amino acids that do not require aminoquinoline ligands have been developed.^95^ The advantages of these methods include not only the direct incorporation of aromatic fragments into amino acids but also the avoidance of side reactions (reduction, elimination, dimerization) associated with the use of serine-derived alkyl bromides.^47^ Based on this rationale, a direct C–H arylation strategy was adopted to introduce biaryl unit 21 into commercially available alanine derivative 24. This transformation proceeded efficiently using glycine derivative 25 as a ligand, affording compound 26 in 71% yield on a gram scale.

Compound 26 was subsequently converted into carboxylic acid 27 by removal of the phthalimide group with ethylenediamine, followed by Boc protection. Sequential condensation of proline derivative 28 and alkyne fragment 30 with 27 using HATU furnished cyclization precursor 31. Regioselective Larock macrocyclization^44,45,47,52,81,113–125^ of precursor 31 under Pd(0) catalysis afforded cyclic compound 32 bearing a Tyr *C*6–to–Trp *C*5′ cross-linkage. Removal of the TES group from 32 yielded compound 33, possessing the RiPP scaffold.

Next, skeletal diversification was employed to expand the chemical space of RiPP scaffold 33 toward quinazoline and quinoline derivatives. Morandi and co-workers recently reported a nitrene-mediated scaffold hopping of indoles to quinazoline derivatives.^82–84^ In this transformation, the high nucleophilicity of the indole and the strong electrophilicity of the nitrene facilitate aziridine formation at the *C*2 and *C*3 positions, followed by ring opening to enable scaffold hopping and generate quinazoline derivatives.

Conversion of the indole moiety of tryptophan residues within peptides to a quinazoline scaffold is attractive from a drug-discovery perspective.^82–84^ This transformation retains the indole's planar topology and hydrophobic interactions while markedly altering its hydrogen-bonding pattern. The quinazoline core replaces the indole's hydrogen-bond donor with two hydrogen-bond acceptors, and such skeletal editing of tryptophan is expected to modulate a compound's target selectivity and pharmacokinetic properties.^82–84^ Moreover, tryptophan is prone to oxidation, which can reduce bioavailability and complicate formulation processes.^126,127^ Skeletal editing to a quinazoline therefore has the potential to enhance oxidative resistance and address these issues. Application of this method to RiPP scaffold 33 was expected to enable access to quinazoline-containing RiPPs, which had previously been difficult to obtain due to strain. In parallel, Sharma and co-workers demonstrated that carbene precursors bearing toluenethiol substituents are effective for indole scaffold hopping, enabling facile conversion to quinoline derivatives.^85^ Quinoline, like quinazoline, alters the hydrogen-bonding pattern of tryptophan and is expected to enhance metabolic stability.

These strategies were evaluated for RiPP scaffold 33. Nitrene precursor 14 was generated *in situ* using PIFA and ammonium carbamate, effecting “*N*” insertion into scaffold 33. The scaffold hopping proceeded smoothly, affording quinazoline derivative 35 bearing a Tyr cross-linkage *via* intermediate 34 in 50% yield. ROESY analysis and DFT calculations revealed that compound 35 possesses an *S*–configured atropisomeric axis. Notably, the atropisomeric axis of scaffold 33 was retained after “*N*” insertion. To date, scaffold hopping has not been applied to biaryl cyclic peptides. Thus, this study demonstrates that skeletal diversification enables late-stage, scalable, and efficient conversion of rigid biaryl cyclic peptides into artificial analogs with potentially distinct physical properties and biological activities.

Carbene-mediated ring expansion of RiPP scaffold 33 was also investigated. Using carbene precursors 36 and 37, bearing toluenethiol and electron-withdrawing substituents, scaffold hopping of 33 was successfully achieved. The reaction proceeded efficiently *via* intermediate 38 to furnish quinoline derivatives 39 and 40, each containing a Tyr cross-linkage, in good yields. Substituents such as –CN and –SO_2_Ph were successfully incorporated into the quinoline framework. These results demonstrate that scaffold hopping of RiPP scaffold 33 proceeds rapidly, enabling facile access to quinazoline derivative 35 and quinoline derivatives 39 (–CN) and 40 (–SO_2_Ph) from a common indole precursor.

The synthesized RiPP scaffold 33, quinazoline derivative 35, and quinoline derivatives 39 (–CN) and 40 (–SO_2_Ph) were each elaborated by sequential introduction of amino acid side chains to yield micitide 982 (5), protected “quinazo” micitide (42), protected “CN-quinolo” micitide (43), and protected “sulfo-quinolo” micitide (44). The modular construction of RiPP scaffolds *via* C–H arylation, followed by scaffold hopping, enabled the efficient and scalable generation of artificial RiPPs containing quinazoline and quinoline motifs from a single intermediate—an achievement of particular significance given the prior inaccessibility of such structures.

**Scheme 2:**
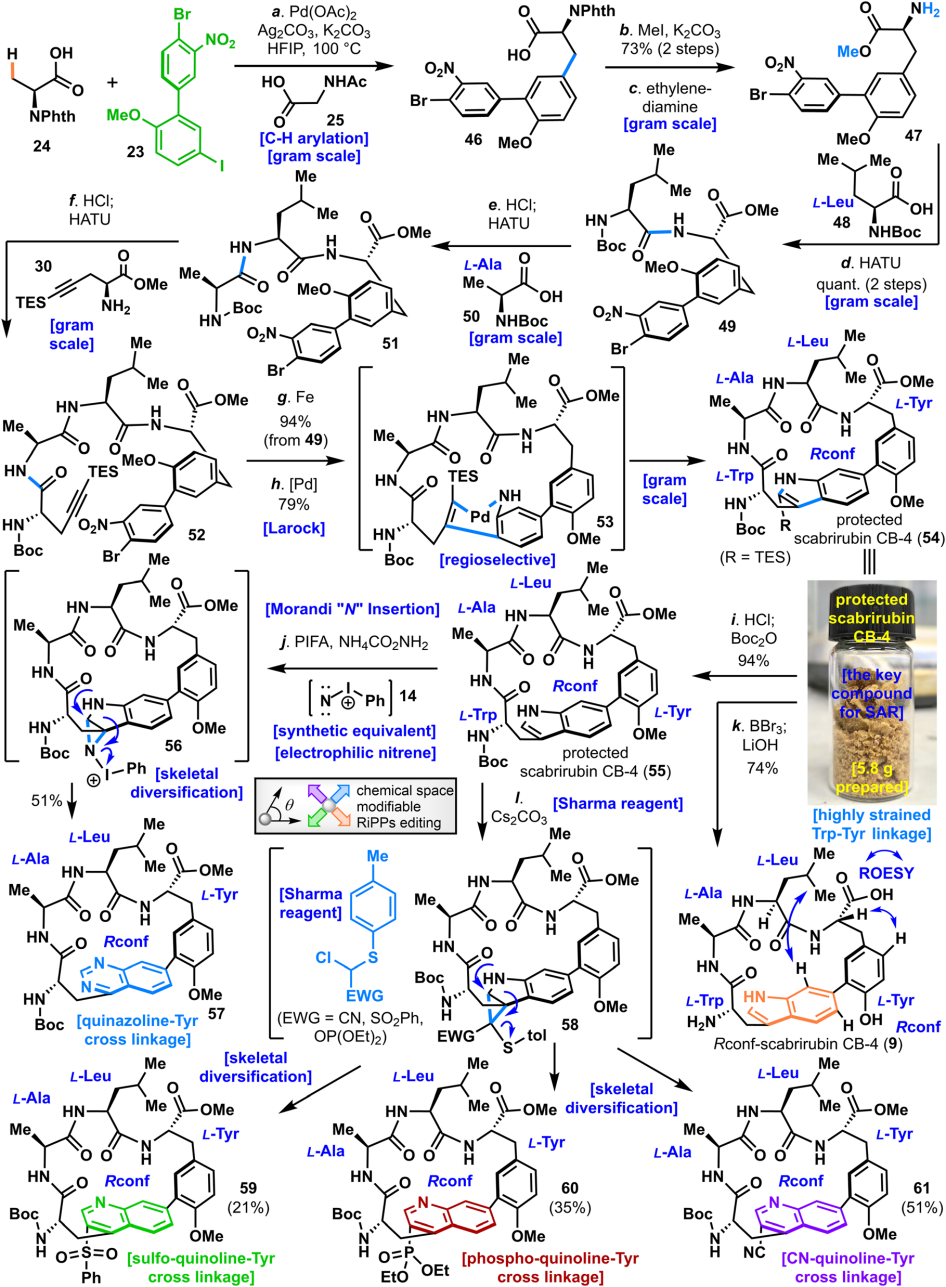
Total synthesis of *R*_conf_–scabrirubin CB-4 (9) and its skeletal diversification. ^a^For detailed reagents and conditions, see the SI.

Finally, expansion of RiPP chemical space was pursued with scabrirubin CB-4 (9 or 10).^14^ As noted above, scabrirubin CB-4 is a non-natural atropopeptide generated by combinatorial biosynthesis, and its atropisomeric configuration has not yet been reported. Accordingly, construction of the RiPP scaffold by C–H arylation and Larock macrocyclization, followed by scaffold hopping, was applied to scabrirubin CB-4 (Scheme 2). In analogy to the synthesis of micitide 982 (5), coupling of biaryl unit 23 with alanine derivative 24*via* C–H arylation introduced the aromatic fragment into the amino acid residue, affording compound 46. Compound 46 was then converted into amine 47 by methyl esterification of the carboxylic acid, followed by phthalimide removal.

Sequential condensation of amine 47 with leucine derivative 48 and alanine derivative 50 using HATU furnished compound 51. The synthesized compound 51 was then subjected to amide coupling with alkyne fragment 30 to furnish macrocyclization precursor 52 on a gram scale. After reduction of the nitro group to the corresponding aniline, a Pd(0)-catalyzed regioselective Larock macrocyclization was attempted. Optimization revealed that the Larock macrocyclization proceeded efficiently to afford intermediate 53 and, subsequently, the protected scabrirubin CB-4 (54), a RiPP bearing a Tyr *C*6–to–Trp *C*6′ cross-linkage. Notably, this route proved amenable to scale-up, delivering 5.8 g of protected scabrirubin CB-4 (54). Global deprotection of 54 using BBr_3_ followed by LiOH afforded, for the first time, scabrirubin CB-4 (9), an atropopeptide featuring a Tyr *C*6–to–Trp *C*6′ cross-linkage. The atropisomeric configuration of scabrirubin CB-4 (9) was assigned as *R* by ROESY analysis. Interestingly, although the HRMS data of synthetic *R*_conf_–scabrirubin CB-4 (9) matched those reported for natural scabrirubin CB-4, the ^1^H NMR spectra did not coincide.^14^ Scabrirubin CB-4 (55) is biosynthesized from a ribosomally synthesized precursor peptide that undergoes post-translational modifications; therefore, the amino-acid backbone is expected to be in the L-configuration.^14,26^ On this basis, we infer that the previously reported, configurationally unassigned scabrirubin CB-4 (55) likely corresponds to the opposite, *S* configuration.

To assess the thermodynamic stability of the cyclized product, protected scabrirubin CB-4 (55) was heated in toluene at 100 °C. No formation of *S*–conformation of scabrirubin CB-4 (10) was observed, suggesting that the synthetic *R*–conformation of scabrirubin CB-4 (9) is thermodynamically stable. Because the Larock indole synthesis requires prolonged heating at 110 °C, it is consistent that the synthetic scabrirubin CB-4 possesses a thermodynamically favored *R*–conformation. By contrast, the Tyr–Trp cross-link in the biosynthetic pathway is formed at room temperature, mediated by P450, which could lead to a different atropisomeric outcome. We next explored the expansion of chemical space *via* skeletal diversification of the obtained atropo-RiPP, *R*-conf-protected scabrirubin CB-4 (55). As a first step, the “*N*–insertion” strategy reported by Morandi and co-workers was applied.^82–84^ Treatment of 55 with PIFA and NH_4_CO_2_NH_2_ effected a smoothring-expansion, proceeding *via* intermediate 56, to provide “quinazo”-scabrirubin CB-4 (57), a RiPP bearing a quinazoline–Tyr cross-linkage. In parallel, we examined the skeletal diversification of *R*_conf_–protected scabrirubin CB-4 (55) using carbene precursors bearing a toluenethiol substituent, as described by Sharma and co-workers.^85^ Sharma *et al.* have demonstrated that a variety of substituents (*e.g.*, –CN, –SO_2_Ph, –PO(OEt)_2_) can be introduced *via* such carbene precursors. Accordingly, a series of carbene precursors was prepared and evaluated for scaffold hopping of the biaryl macrocycle 55. This screening revealed that 55 could be converted into quinoline derivatives 59–61, bearing –CN, –SO_2_Ph, or –PO(OEt)_2_ substituents, respectively. Of note, the skeletal diversification of *R*_conf_–protected scabrirubin CB-4 (55) proceeded in generally lower yields than the corresponding transformations of the micitide scaffold 33. For example, the sulfo-quinoline–Tyr cross-linked product 59 was obtained in 21% isolated yield, whereas the phospho-quinoline–Tyr cross-linked analog 60 was isolated in 35% yield. This outcome can be rationalized by the higher intrinsic strain within *R*_conf_–protected scabrirubin CB-4 (55), which likely slows the ring-expansion step from intermediate 58. Indeed, species consistent with intermediate 58 were observed by TLC and MS analysis. Intermediate 58, corresponding to sulfo-quinoline scabrirubin 59, was isolated as an inseparable mixture of four stereoisomers by silica-gel chromatography. Treatment of this mixture with DIPEA in toluene at 100 °C induced ring-expansion to give compound 59, albeit in low yield. In contrast, in the case of skeletal editing using micitide core 33, no cyclopropane intermediate was detected by the LC-MS and TLC analysis with any of the carbene precursors employed. The ring expansion from the intermediate proceeded smoothly at room temperature. Consequently, yields for the overall skeletal editing process were relatively higher than those for the scabrirubin skeleton. These results suggest that the higher intrinsic strain of the scabrirubin framework impedes ring-expansion from intermediate 58. The finding that the degree of macrocyclic strain in RiPPs can influence the rate of ring-expansion reactions is noteworthy and merits further attention.

**Fig. 3:**
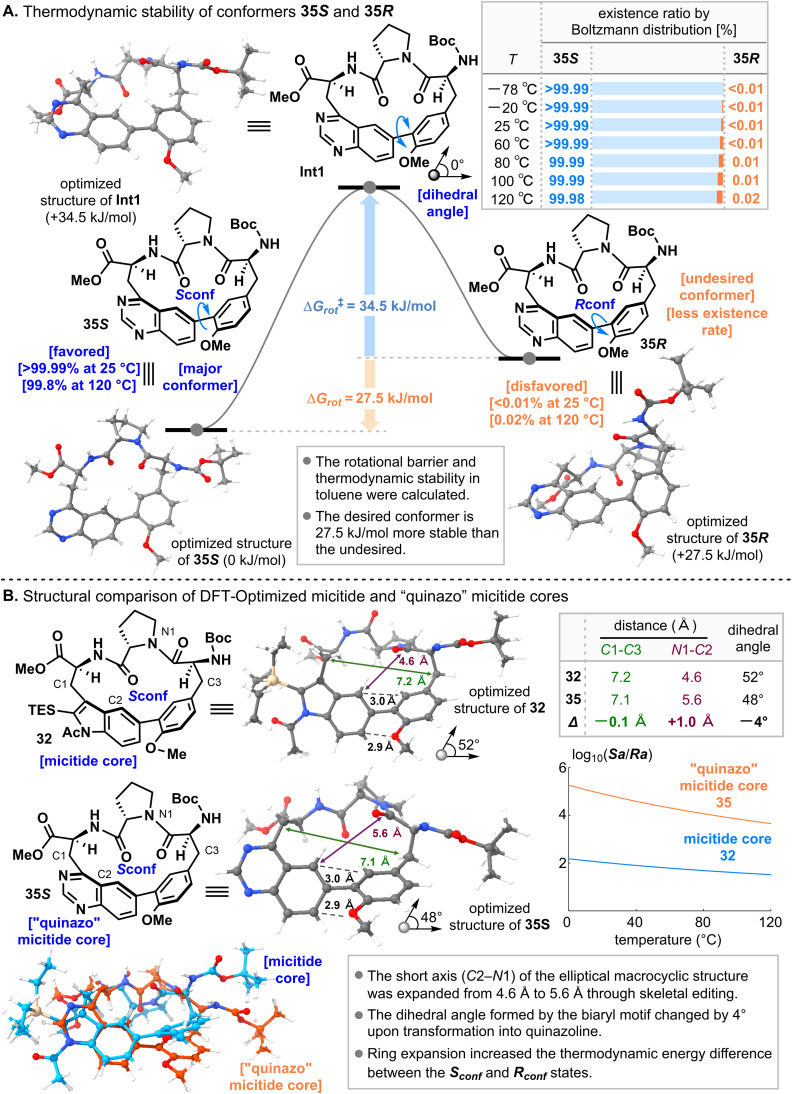
(A) DFT calculations on the thermodynamic stability of 35*S* and 35*R*. (B) Structural comparison of DFT-optimized structures.

The quinazoline–Tyr cross-linked product 35, obtained by scaffold hopping of the RiPP micitide core 33, was shown by ROESY to possess an *S*–configured chiral axis. It is particularly interesting that the atropisomeric configuration of the starting RiPP micitide scaffold 33 is preserved throughout the ring-expansion process to give quinazoline 35. To gain further insight, the rotational barrier of the quinazoline–Tyr cross-linkage in 35 was investigated by DFT calculations (Fig. 3). For the *S*– and *R*–atropisomers, 35*S* and 35*R*, the calculated barrier for rotation from 35*S* to 35*R* was Δ*G*_rot_^‡^ = 34.5 kJ mol^−1^. In general, atropisomers require a barrier of approximately 90 kJ mol^−1^ or higher to be separable at room temperature.^128^ Thus, rotation about the chiral axis from 35*S* to 35*R* is, in principle, feasible at room temperature. However, comparison of the relative thermodynamic stabilities of 35*S* and 35*R* revealed that 35*R* is higher in energy than 35*S* by +27.5 kJ mol^−1^. According to a Boltzmann distribution at 25 °C, this corresponds to a 35*S*/35*R* ratio of >99.99:<0.01. Therefore, although rotation from 35*S* to 35*R* is theoretically possible at room temperature, the overwhelming thermodynamic preference for 35*S* ensures that, in practice, 35*S* is obtained in >99% abundance—consistent with experimental observations. Indeed, the single atropisomeric quinazoline 35 obtained by scaffold hopping was assigned as 35*S* (*S*–configuration) by ROESY, and no trace of 35*R* was detected experimentally. Furthermore, even at an elevated temperature of 120 °C, the Boltzmann distribution predicts a 35*S*/35*R* ratio of 99.98 : 0.02, indicating that 35*S* is highly stable and that its chiral axis is retained under high-temperature conditions.

To evaluate the effect of quinazoline incorporation on the molecular properties of RiPPs, a structural comparison was performed between the DFT-optimized micitide core 32 (ref. 47) and the “quinazo” micitide core 35 (Fig. 3B). The major axis (*C*1–*C*3) of the elliptical macrocyclic structure differed by only 0.1 Å between the two cores, whereas the minor axis (*N*1–*C*2) was suggested to be 1.0 Å longer in the “quinazo” micitide core 35. The dihedral angle was 52° in micitide core 32, while in “quinazo” micitide core 35 it was 48°.

For the corresponding Larock macrocyclization product 32, the rotational barrier between the *S*– and *R*–atropisomers was calculated to be Δ*G*_rot_^‡^ = 32.6 kJ mol^−1^, comparable to that of 35 (Δ*G*_rot_^‡^ = 34.5 kJ mol^−1^).^47^ Intriguingly, DFT analysis showed that 32S is more stable than 32R by 11.4 kJ mol^−1^.^47^ This energy difference is substantially smaller than that between 35S and 35R (Δ*G*_rot_ = +27.5 kJ mol^−1^). Thus, conversion of the indole unit to a quinazoline *via* scaffold hopping increases the energy gap between the *S* and *R* atropisomers, thereby enhancing the atropisomeric stability of the RiPP scaffold. Assuming a Boltzmann distribution, the relative populations of the *S*_conf_ and *R*_conf_ states were calculated from the thermodynamic energy differences obtained by DFT, and the common logarithm of the isomeric ratio, log_10_(*S*_conf_/*R*_conf_), was plotted as a function of temperature (Fig. 3B). At all temperatures examined, the isomeric ratio of the “quinazo” micitide core was approximately 10^3^-fold greater than that of the micitide core, indicating that the equilibrium is strongly shifted toward the *S*_conf_ state. For example, at 25 °C, the isomeric ratio of micitide core 32 was *S*_conf_ : *R*_conf_ = 99 : 1, whereas that of the “quinazo” micitide core 35 was *S*_conf_ : *R*_conf_ = 6.6 × 10^4^ : 1, suggesting the strong shift of the equilibrium toward the *S*_conf_ state.

This effect is likely attributable to the replacement of the five-membered indole ring with a six-membered quinazoline moiety, which significantly alters the overall macrocyclic conformation. Upon superimposition of the DFT-optimized structures of micitide core 32 (sky blue, Fig. 3B) and the ‘quinazo’ micitide core 35*S* (orange, Fig. 3B), a pronounced alteration of the entire ring architecture is observed. Skeletal editing to introduce quinazoline induced not only local changes around the biaryl motif but also substantial alterations in the overall cyclic framework. This structural reorganization is presumed to have caused a pronounced shift in the equilibrium between the *S*_conf_ and *R*_conf_.

**Fig. 4:**
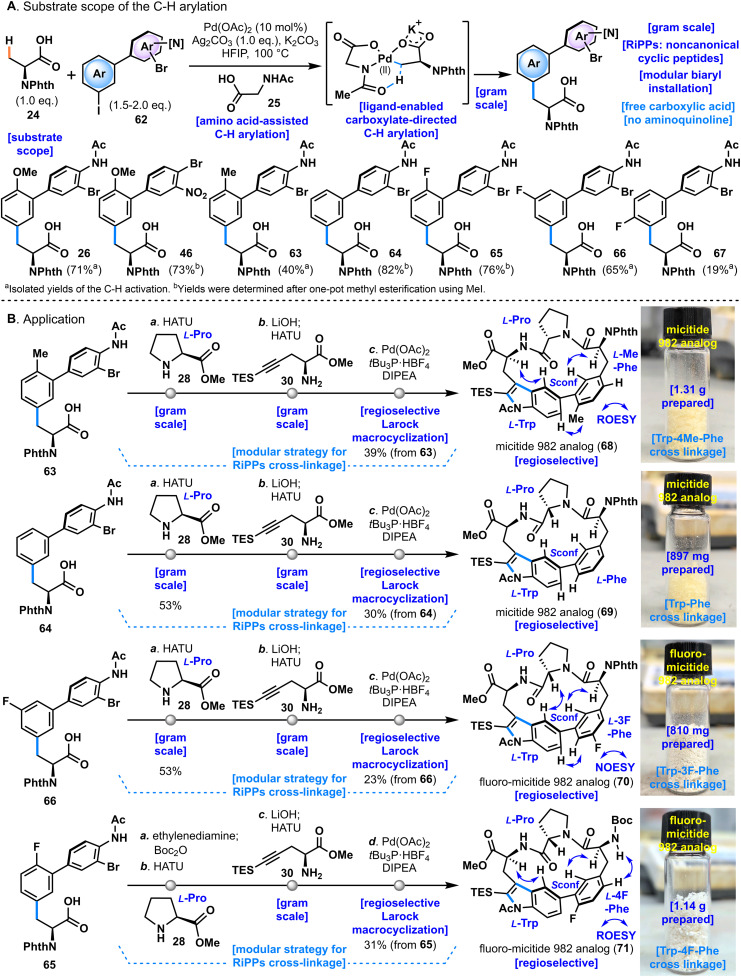
(A) Substrate scope of the C–H arylation. (B) Modular synthesis of RiPP scaffolds based on Larock cyclization. ^*a*^For detailed reagents and conditions, see the SI.

To further validate the generality and utility of the modular synthesis of RiPP scaffolds based on C–H arylation and regioselective Larock macrocyclization, we examined a range of non-natural biaryl units (Fig. 4A). In addition to biaryl motifs that can be elaborated into micitide 982 (5) and scabrirubin CB-4 (9) (compounds 26 and 46), amino-acid-assisted C–H arylation was successfully applied to substrates in which the aromatic ring on the tyrosine side was substituted with a methyl group, affording the corresponding carboxylic acid 63.

**Fig. 5:**
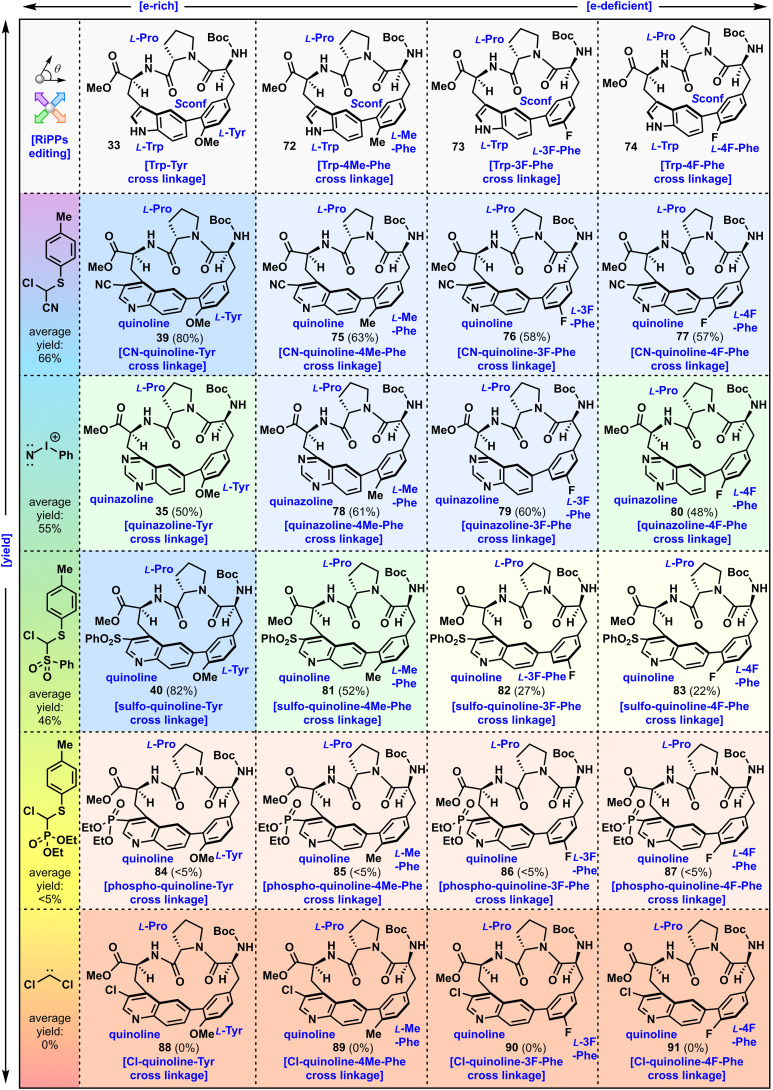
Heat map for the skeletal diversification of the RiPPs core. ^*a*^For detailed reagents and conditions, see the SI.

Introduction of RiPP scaffolds onto the obtained biaryl units was next investigated (Fig. 4B). A key feature of this synthetic strategy is its ability to provide a variety of biaryl-containing RiPPs in a modular manner. Thus, carboxylic acid 63 was first coupled with proline derivative 28 using HATU, followed by attachment of alkyne fragment 30 to furnish the corresponding macrocyclization precursor. The macrocyclization proceeded efficiently in the presence of Pd(0) and a bulky ligand, affording the micitide 982 analogue 68, bearing a Trp–4Me–Phe cross-linkage, on a 1.31 g scale. Likewise, application of the same modular sequence to carboxylic acid 64 provided RiPP scaffold 69, featuring a Trp–Phe cross-linkage, on an 897 mg scale. Fluorinated, electron-deficient RiPP scaffolds are expected to exhibit increased oxidative stability and enhanced metabolic stability in biological systems.^129–131^ Accordingly, macrocyclization of carboxylic acids 66 and 65 was examined under analogous conditions. The modular sequence again proceeded smoothly to afford fluorinated artificial RiPPs 70 and 71, bearing Trp–3F–Phe and Trp–4F–Phe cross-linkages, respectively. Collectively, these results demonstrate that a broad range of biaryl motifs can be incorporated into RiPP frameworks using the present modular strategy based on C–H arylation and Larock macrocyclization. Systematic expansion of chemical space *via* skeletal diversification of the resulting RiPP scaffolds was then pursued to evaluate the generality of this approach (Fig. 5). Carbene precursors bearing various substituents (–CN, –SO_2_Ph, –PO(OEt)_2_), as reported by Sharma and co-workers,^85^ together with the nitrene-mediated “*N*–insertion” protocol developed by Morandi and co-workers,^82–84^ were applied to RiPP derivatives 33, 72–74. When a carbene precursor bearing a –CN substituent was employed, skeletal diversification of the RiPPs proceeded rapidly, enabling efficient synthesis of a series of biaryl macrocyclic peptides (39, 75–77) incorporating quinoline cores, with an average isolated yield of 66%. Notably, both electron-rich and electron-deficient RiPP scaffolds were competent substrates for this quinoline-forming transformation.

The next most efficient manifold involved nitrene-mediated “*N*–insertion” to construct quinazoline cores. This protocol was successfully applied to all tested RiPP derivatives (33, 72–74), providing quinazoline-containing artificial RiPPs 35 and 78–80. As in the quinoline series, both electron-rich and electron-poor RiPPs were tolerated, and the average isolated yield was 55%. In contrast, when carbene precursors bearing a –SO_2_Ph substituent were used for skeletal diversification, quinoline derivatives 40 and 81–83 were obtained in a somewhat lower average yield of 46%. In this case, electron-deficient RiPP scaffolds (82, 83) tended to give diminished yields, whereas more electron-rich substrates afforded higher yields. In contrast to the case of scabrirubin, no cyclopropane intermediate was observed when the micitide core was employed. On the other hand, for substrates with low yields, although the starting material was completely consumed, multiple unidentified byproducts were obtained. These observations suggest that, in such substrates, cyclopropanation of the indole by the carbene species does proceed, but several undesired side reactions compete simultaneously.

Further studies revealed that the use of carbene precursors bearing a –PO(OEt)_2_ substituent resulted in almost no formation of quinoline derivatives 84–87; only trace amounts (<5%) were detected for both electron-poor and electron-rich substrates. Finally, as a comparative experiment, classical dichlorocarbene was employed for scaffold hopping of the RiPPs. However, none of the desired products 88–91 were obtained, and only decomposition of the substrates was observed. This outcome is plausibly attributable to the rapid generation of dichlorocarbene, which likely leads to a high steady-state carbene concentration in the reaction mixture, thereby promoting overreaction and undesired side processes.

## Conclusions

Owing to dramatic advances in genome mining, numerous RiPPs have been discovered in recent years, some of which exhibit potent antibacterial and antitumor activities.^1–6^ In the present study, we have developed a general modular synthetic strategy for RiPPs bearing Tyr–Trp cross-linkages, based on C–H arylation. This approach enables gram-scale access to RiPPs that were previously difficult to obtain. Furthermore, we have demonstrated that these RiPPs can undergo skeletal diversification *via* carbene- and nitrene-mediated transformations to afford artificial biaryl RiPPs incorporating quinazoline–Tyr and quinoline–Tyr cross-linkages, thereby providing systematic access to previously unexplored regions of chemical space. As medium-sized peptides are emerging as a promising modality in drug discovery, the demand for conformationally constrained cyclic peptides with high metabolic stability and strong target protein binding is expected to continue to grow.^132–134^ Accordingly, the streamlined synthesis of biaryl-containing RiPPs, together with their skeletal diversification demonstrated herein, is expected to contribute meaningfully toward addressing pressing societal and pharmaceutical demands.

## Author contributions

^†^H. O., Y. L. contributed equally to this work. H. O., Y. L., S. L., T-K. C., and H. N. conducted the experiments. H. N. conceptualized and designed the synthetic strategy. Y. N. conducted the computational study. J. L., Y-X. L. and Y. M. contributed insights into isolation, purification, and structural elucidation. The manuscript was prepared by H. N. with the feedback of all other authors. H. O. and Y. L. prepared the supplementary information under the guidance of H. N.

## Conflicts of interest

The authors declare no competing interests.

## Supplementary Material

SC-017-D6SC03088B-s001

## Data Availability

The authors declare that the data supporting the findings of this study are available within the article and the supplementary information (SI) as well as from the authors upon request. Supplementary information: experimental procedures, experimental equipment, characterization data, computational results, HRMS, and NMR spectra for all new compounds. See DOI: https://doi.org/10.1039/d6sc03088b.
