# Supplementary material for: Reprogramming RiPP scaffolds through skeletal editing unlocks chemical space
Source: Chem Sci. 2026 May 26;17(28):13835–49. doi: 10.1039/d6sc03088b (PMC13250620; doi:10.1039/d6sc03088b)

Supplementary Information (SI) for Chemical Science.
This journal is © The Royal Society of Chemistry 2026

SUPPORTING INFORMATION

Reprogramming RiPP Scaffolds through Skeletal Editing Unlocks Chemical Space

Hiroshige Ogawa, §[a] Longhui Yu, §[a] Shangzhao Li, [a] Yuuya Nagata, [b] Tsz Ki Chan, [c] Yudai Matsuda, [c] Jing Liu, [d] Yong-Xin Li, [d] Hugh Nakamura*[a]

[a]Department of Chemistry, The Hong Kong University of Science and Technology, Clear Water Bay, 999077 Hong Kong SAR, China

[b]Autonomous Polymer Design and Discovery Group Research Center for Macromolecules and Biomaterials, National Institute forMaterials Science (NIMS), 1-2-1 Sengen, Tsukuba, Ibaraki 305-0047,Japan

[c]Department of Chemistry, City University of Hong Kong, Hong Kong SAR, China

[d]Department of Chemistry and The Swire Institute of Marine Science, The University of Hong Kong, 999077 Hong Kong SAR, China

([§]These authors contributed equally to this work.)

General Experimental

Reagents were purchased from commercial sources (Bide, Energy, TCI, and Sigma-Aldrich) and used without further purification unless otherwise stated. Yields refer to chromatographically unless otherwise stated. Reactions were monitored by LC/MS and thin layer chromatography (TLC). TLC was performed using 0.2-0.25 mm silica plates, and using short-wave UV light as the visualizing agent, phosphomolybdic acid with $Ce(SO_4)_2$, or $KMnO_4$ via heat as developing agents. NMR spectra were recorded on Bruker AVII 400 and JEOL 600 instruments and are calibrated using residual undeuterated solvent ($CHCl_3$ at 7.26 ppm ^{1}H NMR, 77.16 ppm ^{13}C NMR). The following abbreviations were used to explain multiplicities: s = singlet, d = doublet, t = triplet, q = quartet, m = multiplet, br = broad. Column chromatography was performed using 230-400 mesh silica gel, and PTLC was performed using 0.2-0.25 mm silica plates. High-resolution mass spectra (HRMS) were recorded on an Agilent LC/MSD TOF mass spectrometer by electrospray ionization time of flight reflectron experiments.

Abbreviations

Ac	acetyl
aq.	aqueous
Boc	*tert*-butoxycarbonyl
Bu	butyl
cat.	catalyst
DFT	density functional theory
DIPEA	*N*-diisopropylethylamine
DMSO	dimethyl sulfoxide
HATU	1-[bis(dimethylamino)methylene]-1H-1,2,3-triazolo[4,5-b]pyridinium 3-oxide hexafluorophosphate
Ph	phenyl
PPh_3	triphenylphosphine
rt	25 °C
TFA	trifluoroacetic acid
THF	tetrahydrofuran
dppe	1,2-Bis(diphenylphosphino)ethane
dppf	1,1'-Bis(diphenylphosphino)ferrocene
Phth	Phtalimide
Phg	Phenylglycine
DNP	Dinitrophenyl
AQ	Aminoquinoline

Table of Contents

General Experimental 2
Synthetic route 4
C-H Arylation and One-Pot Methyl Esterification (General Procedure B) 6
Synthesis of micitide 982 core 6
Key NOESY correlation of micitide core (32) 10
Skeletal Editing of Micitide Core 10
Key ROESY correlation of quinazomicitide core (35) 12
Sidechain Elongation of the Micitide Core Analogues (General Procedure C) 13
Synthesis of Scabrirubin CB-4 19
Key NOESY Correlation of *Rconf*-scabrirubin CB-4 24
LC-MS trace of synthetic scabrirubin CB-4 25
Skeletal Editing of Scabrirubin CB-4 25
Substrate Scope of CH arylation 29
Key ROESY Correlations of Compound 68 34
Key ROESY Correlations of Compound 70 37
Key NOESY Correlations of Compound S26 41
Skeletal Editing Employing Unnatural Micitide Core 42
Reaction optimization and discussion 49
Optimization table of carboxylate-assisted C-H activation 49
Aminoquinoline-directed C-H activation 49
Optimization table of Larock cyclization 51
Assessment of the thermodynamic stability of protected *R*-conf scabrirubin CB-4 (55) 52
Skeletal editing of scabrirubin core: stepwise procedure 52
LC-MS spectrum of cyclopropane intermediate S29 53
Computational Details 54
Cartesian Coordinates 55
NMR Spectrums 62

Synthetic route

Scheme S1. Total synthesis of micitide 982 (**1**) and its skeletal diversification.

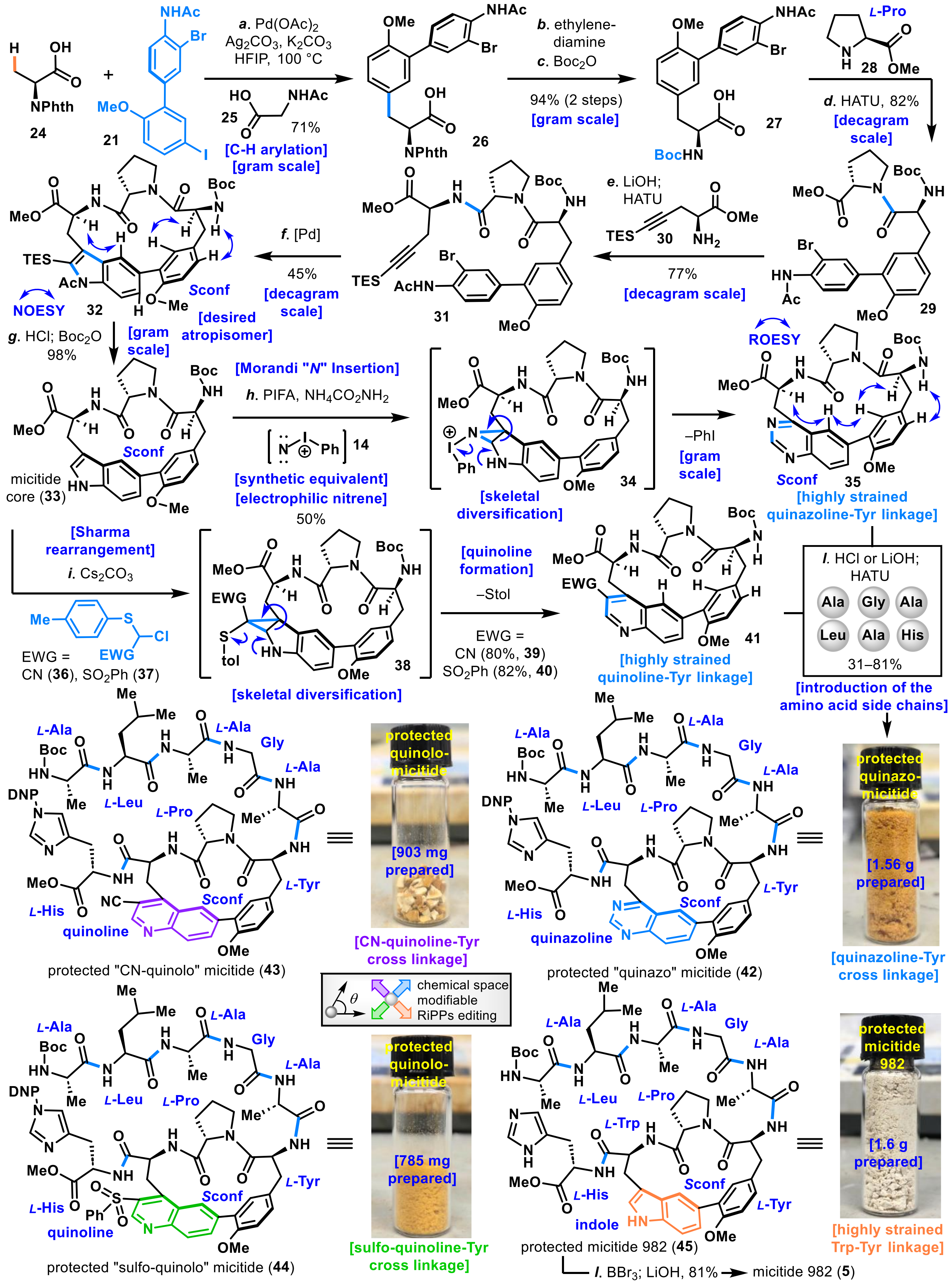

Scheme S2. Total synthesis of R_{conf}-scabrirubin CB-4 (**9**) and its skeletal diversification.

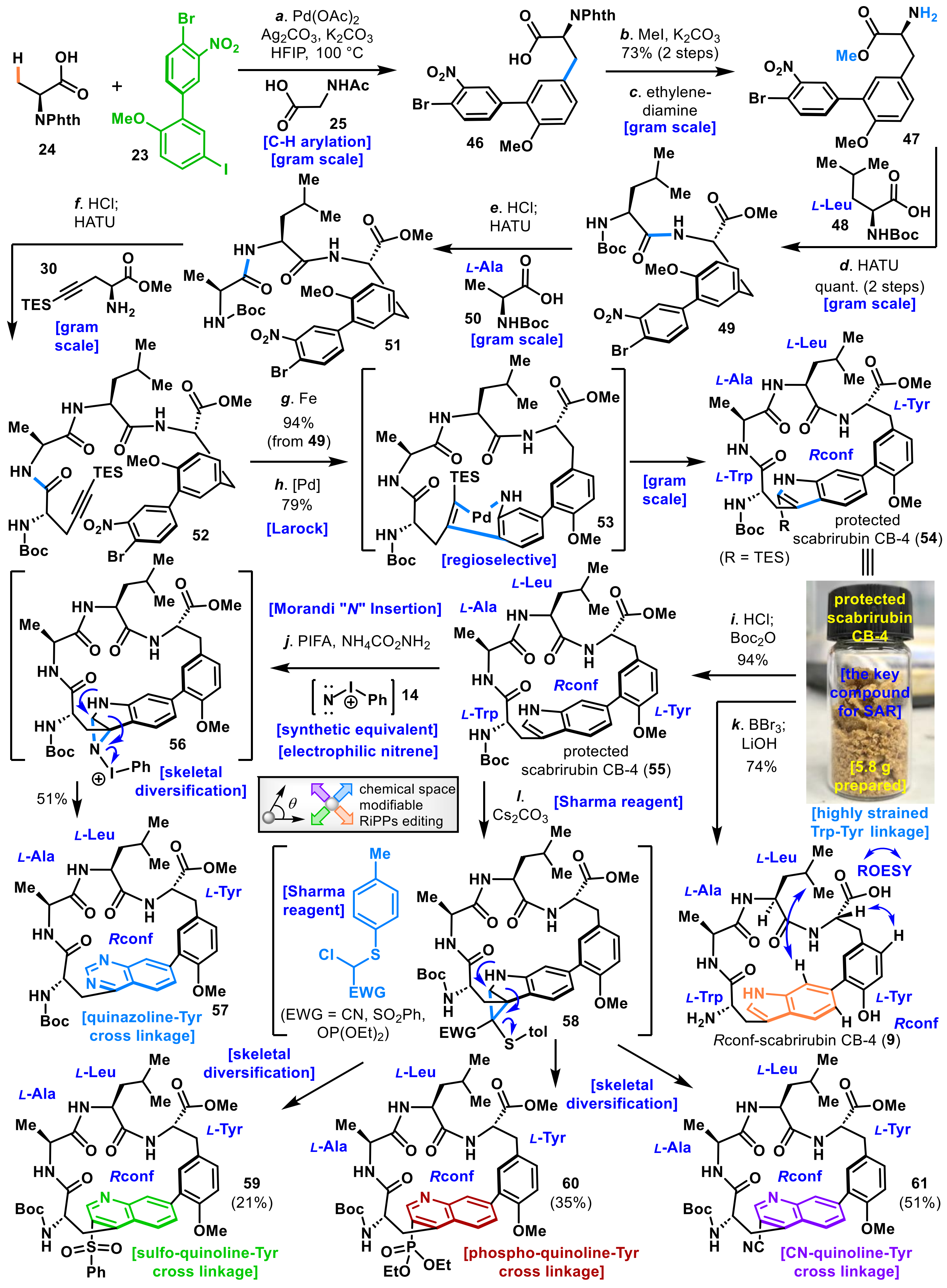

C-H Arylation (General Procedure A)

Biaryl iodide **21** was prepared according to the procedure previously reported by our group.[2]

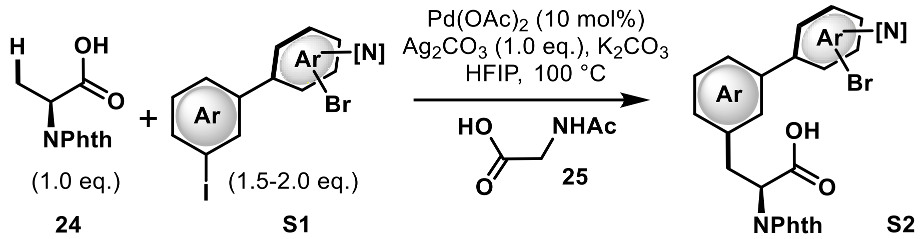

A mixture of Pht-Ala-OH **24** (1.0 eq.), aryl iodide **S1** (1.5 eq.), $Pd(OAc)_2$ (0.1 eq.), Ag_2CO_3 (1.0 eq.), Ac-Gly-OH (0.3 eq.) and K_2CO_3 (0.5 eq.), HFIP (0.1 M) in a sealed tube was heated at 100 °C with vigorous stirring for 12 hours. The reaction mixture was cooled to 25 °C, quenched with 1N HCl aq., and diluted with EtOAc. The mixture was filtered through Celite, and the aqueous phase was extracted with EtOAc three times. The organic phase was combined and dried over Na_2SO_4. The residue was purified by preparative thin-layer chromatography or silica gel column chromatography.

C-H Arylation and One-Pot Methyl Esterification (General Procedure B)

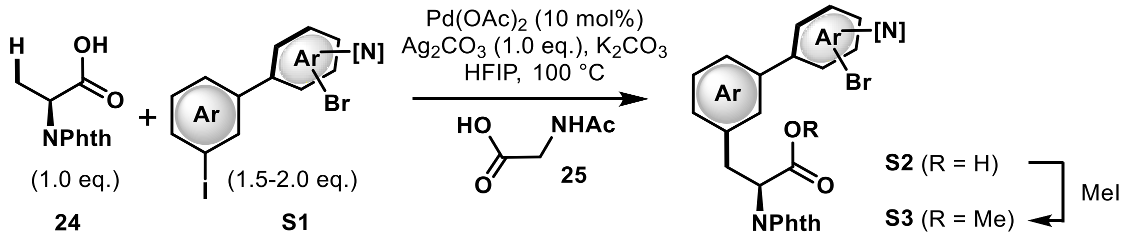

A mixture of Pht-Ala-OH **24** (1.0 eq.), aryl iodide **S1** (1.5 eq.), $Pd(OAc)_2$ (224.5 mg, 1.0 mmol, 0.1 eq.), Ag_2CO_3 (1.38 g, 5.0 mmol, 1.0 eq.), Ac-Gly-OH (234 mg, 2.0 mmol, 0.4 eq.) and K_2CO_3 (345 mg, 2.5 mmol, 0.5 eq.), HFIP (50 mL) in a sealed tube was heated at 100 °C with vigorous stirring for 12 hours. The reaction mixture was cooled to 25 °C and directly evaporated under vacuum. To the residue, K_2CO_3 (5 eq.), acetone (0.1 M), MeI (30 eq.) were added sequentially, and the reaction was stirred at 25 °C for 2 h. The reaction was quenched with water, extracted with EtOAc three times, and the combined organic layers were washed with brine, dried over Na_2SO_4, and evaporated under vacuum. The residue was purified by preparative thin-layer chromatography or silica gel column chromatography.

Synthesis of micitide 982 core

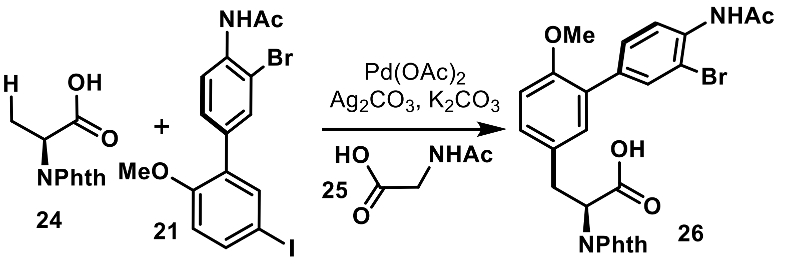

On 5.0 mmol scale, **General Procedure A** was followed with aryl iodide **21**. Purification by silica gel column chromatography (EtOAc:Hexane 1:2 to 4:1) gave the title compound **26**. (1.89 g, 71% yield)

Compound 26

Physical State: off-white solid

^{1}H NMR (600 MHz, DMSO-*d*$_6$): δ 9.46 (s, 1H), 7.81 (s, 4H), 7.39 (m, 2H), 6.94 (m, 4H), 5.06 (s, 1H), 3.62 (s, 3H), 3.28 (m, 3H), 2.05 (s, 3H).

^{13}C NMR (151 MHz, DMSO-*d*$_6$): δ 170.64, 169.15, 167.80, 155.14, 137.01, 135.52, 133.09, 131.34, 131.12, 130.24, 130.18, 129.07, 127.82, 127.00, 123.91, 117.85, 112.18, 79.70, 55.99, 53.87, 33.60, 23.76.

HRMS (ESI-TOF): calculated for $C_{26}H_{21}BrN_2NaO_6^+$ [M+Na]$^+$: 559.0475, found: 559.0484.

TLC: R_f = 0.2 (10:1 DCM:Methanol, $Ce_2(SO_4)_3$ in phosphomolybdic acid).

[α]$^{25}_{D}$: -68.6 (*c* = 0.1, $CHCl_3$)

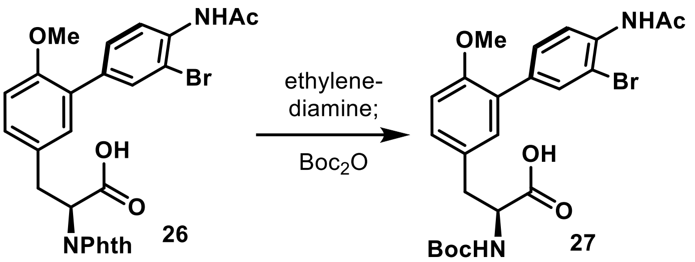

Compound **26** (1.89 g, 3.5 mmol, 1.0 eq.) was dissolved in *n*-BuOH (35 mL), and ethylenediamine (2.35 mL, 35.2 mmol, 10.0 eq.) was added. The reaction was stirred at 25 °C for 15 h. The reaction mixture was directly concentrated under reduced pressure to give the crude. The crude was dissolved in THF (35 mL), H_2O (3.5 mL). $NaHCO_3$ (2.06 g, 24.5 mmol, 7.0 eq.) and $(Boc)_2O$ (4.0 mL, 17.5 mmol, 5.0 eq.) were added sequentially. The reaction mixture was stirred at 25 °C for 3 h and quenched by saturated *aq.* NH_4Cl, extracted with DCM/MeOH = 10:1 for three times. The organic layers were combined and washed with saturated *aq.* NaCl, over Na_2SO_4. Organic layers were concentrated under reduced pressure and purified by silica gel chromatography to give compound **27** (1.54 g, 94% yield over two steps).

Note: A total of 21 g compound **27** was obtained.

Compound 27

Physical State: colorless oil

^{1}H NMR (600 MHz, $CDCl_3$, a mixture of two rotamers, A:B = 3:1, Signals of the major rotamer are reported.): δ 8.23 (d, *J* = 8.2 Hz, 1H): 7.66 (m, 2H), 7.42 (m, 1H), 7.08 (m, 2H), 6.84 (d, *J* = 8.3 Hz, 1H), 5.13 (d, *J* = 7.1 Hz, 1H), 4.47 (m, 1H), 3.74 (s, 3H), 3.06 (m, 2H), 2.21 (s, 3H), 1.34 (s, 9H).

^{13}C NMR (151 MHz, $CDCl_3$, a mixture of two rotamers, A:B = 3:1, Signals of the major rotamer are reported.): 175.42, 168.97, 155.50, 135.88, 134.23, 133.00, 132.41, 131.77, 130.07, 129.61, 128.57, 128.39, 121.79, 113.26, 111.48, 55.69, 54.57, 37.08, 28.40, 24.78.

HRMS (ESI-TOF): calculated for $C_{23}H_{27}BrN_2NaO_6^+$ [M+Na]$^+$: 529.0945, found: 529.0953.

TLC: R_f = 0.5 (only EtOAc, $Ce_2(SO_4)_3$ in phosphomolybdic acid).

[α]$^{25}_{D}$: +38.8 (*c* = 1.0, $CHCl_3$)

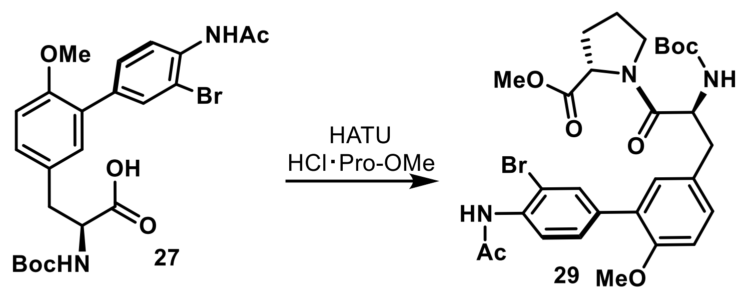

To the mixture of compound **27** (11.6 g, 23.1 mmol, 1.0 eq.), methyl L-prolinate (5.75 g, 34.7 mmol, 1.5 eq.), DIPEA (20.4 mL, 116 mmol, 5.0 eq.), was added HATU (13.2 g, 34.7 mmol, 1.5 eq.). The reaction mixture was stirred at for 3 h and quenched with *aq.* 0.5 M HCl, extracted with EtOAc three times. The organic layers were combined and washed with saturated *aq.* NaCl. The organic layers were concentrated under reduced pressure to give and purified by silica gel chromatography to give compound **29** (11.7 g, 82%).

Compound 29

Physical State: white solid

^{1}H NMR (400 MHz, DMSO-d_6, 60 °C a mixture of two rotamers, A:B = 4:1): δ 9.26 (s, 1H), 7.59 (m, 5H), 7.19 (s, 1H), 7.02 (d, J = 9.0 Hz, 1H), 6.70 (m, 1H), 4.39 (m, 2H), 3.76 (s, 3H), 3.62 (s, 3H), 3.54 (m, 1H), 2.93 (m, 1H), 2.78 (dd, J = 13.3, 9.1 Hz, 1H), 2.17 (m, 1H), 2.11 (s, 3H), 1.93 (m, 2H), 1.83 (m, 1H), 1.29 (s, 9H).

^{3}C NMR (101 MHz, DMSO-d_6, 60 °C, a mixture of two rotamers, A:B = 4:1): δ 172.00, 169.99, 168.40, 154.74, 136.66, 134.99, 133.25, 132.65, 132.54, 132.23, 131.74, 131.72, 131.35, 131.25, 129.99, 129.82, 128.64, 128.52, 128.41, 127.27, 125.94, 116.97, 111.70, 78.95, 78.00, 58.47, 58.34, 55.58, 53.29, 51.92, 51.45, 46.28, 45.89, 35.74, 30.59, 28.35, 27.92, 24.46, 23.04, 21.46.

HRMS (ESI-TOF): calculated for $C_{29}H_{36}BrN_3NaO_7^+$ [M+Na]$^+$: 640.1629, found: 640.1633.

TLC: R_f = 0.7 (only EtOAc, $Ce_2(SO_4)_3$ in phosphomolybdic acid).

[α]$^{25}_D$: -18.5 (c = 1.0, $CHCl_3$)

Note: Due to rotamerism at room temperature, NMR spectra for structural assignment were recorded at 60 °C in DMSO-d6. However, even at this elevated temperature, the rotamers remained partially unresolved. Signals of the major rotamers were reported.

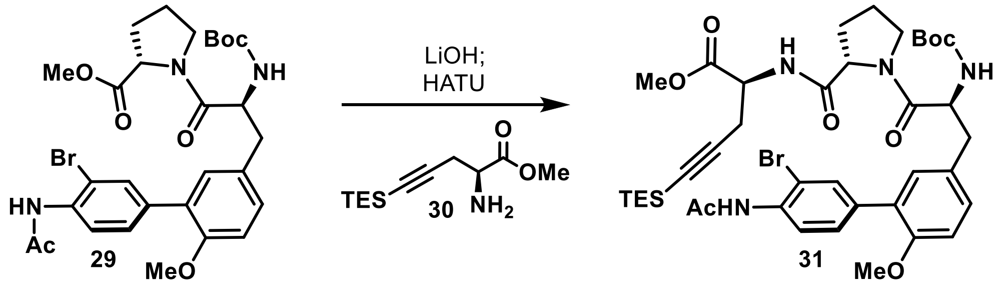

Compound **29** (20.0 g, 32.4 mmol, 1.0 eq.) was dissolved in THF/MeOH/H_2O = 1:1:1 (600 mL), then LiOH·H_2O (8.16 g, 194 mmol, 6.0 eq.) was added, the reaction mixture was stirred at 25 °C for 1 h. The reaction mixture was acidified with *aq.* 0.5M HCl gradually and extracted with DCM/MeOH = 10:1 three times. The organic layers were combined and washed with saturated *aq.* NaCl. The organic layers were concentrated under reduced pressure to give the crude. The crude was dissolved in DMF (320 mL), compound **30** (15.1 g, 42.5 mmol, 1.3 eq.), DIPEA (28.9 mL, 162 mmol, 5.0 eq.), and HATU (18.5 g, 48.6 mmol, 1.5 eq.) were added sequentially. The reaction mixture was stirred at 25 °C for 1.5 h, quenched by saturated *aq.*

0.5 M HCl, extracted with Hexane:EtOAc = 1:1 three times, the organic layers were combined and washed with saturated *aq.* NaCl. The organic layers were concentrated under reduced pressure and purified by silica gel chromatography to give compound **31** (26.8 g, 77% yield). The characteristics data were identical to those previously reported by our group, obtained via a different synthetic route.[2]

Compound 31

Physical State: white solid

^{1}H NMR (400 MHz, DMSO-*d*$_6$, 60 °C, a mixture of two rotamers, A:B = 4:1, Signals of the major rotamer are reportd.): δ 9.25 (s, 1H), 7.97 (d, *J* = 7.1 Hz, 1H), 7.60 (m, 4H), 7.26 (m, 2H), 7.01 (d, *J* = 8.0 Hz, 1H), 6.69 (m, 1H), 4.44 (s, 3H), 3.76 (s, 3H), 3.62 (m, 5H), 2.77 (m, 4H), 2.11 (s, 3H), 1.99 (m, 3H), 1.28 (s, 9H), 0.94 (t, *J* = 7.8 Hz, 9H), 0.54 (q, *J* = 7.6 Hz, 6H).

^{13}C NMR (101 MHz, DMSO-*d*$_6$, 60 °C, a mixture of two rotamers, A:B = 4:1, Signals of the major rotamer are reported.): δ 171.30, 170.40, 170.26, 168.37, 154.71, 136.64, 135.00, 132.63, 131.74, 131.71, 131.35, 131.25, 131.19, 130.09, 129.95, 128.61, 128.53, 128.41, 127.25, 125.94, 116.96, 111.74, 103.40, 83.70, 77.93, 59.29, 55.58, 53.40, 51.69, 51.27, 46.56, 35.80, 28.61, 27.92, 24.28, 23.04, 22.44, 6.97, 3.85.

HRMS (ESI-TOF): calculated for $C_{40}H_{55}BrN_4NaO_8Si^+$ $[M+Na]^+$: 849.2865, found: 849.2870.

TLC: R_f = 0.6 (3:2 EtOAc : Hexane, $Ce_2(SO_4)_3$ in phosphomolybdic acid).

$[\alpha]^{25}_{D}$: +10.2 (*c* = 0.1, $CHCl_3$)

Note: Due to rotamerism at room temperature, NMR spectra for structural assignment were recorded at 60 °C in DMSO-d6. However, even at this elevated temperature, the rotamers remained partially unresolved. Signals of the major rotamers were reported.

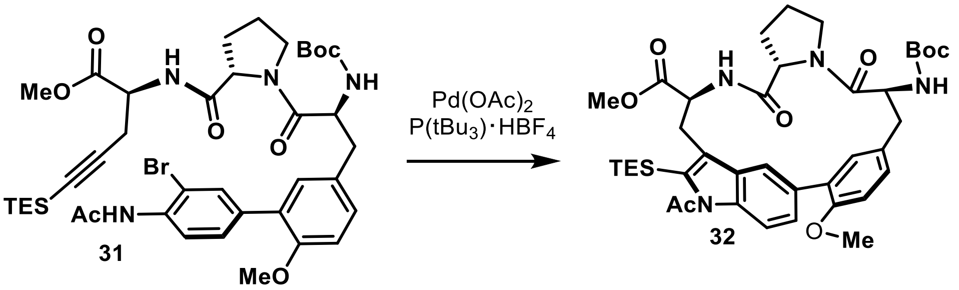

The reaction was conducted following the previously reported procedure. The characteristic data were identical to those previously reported.[2]

Compound 32

Physical State: white solid

^{1}H NMR (600 MHz, CDCl$_3$): δ 7.82 (d, *J* = 8.8 Hz, 1H), 7.61 (d, *J* = 8.8 Hz, 1H), 7.41 (s, 1H), 7.34 (s, 1H), 7.01 (d, *J* = 8.3 Hz, 1H), 6.86 (d, *J* = 8.3 Hz, 1H), 6.79 (m, 1H), 5.17 (m, 1H), 4.93 (t, *J* = 9.1 Hz, 1H), 4.78 (t, *J* = 7.5 Hz, 1H), 4.45 (m, 1H), 3.81 (s, 3H), 3.77 (m, 1H), 3.72 (m, 1H), 3.68 (s, 3H), 3.65 (m, 1H), 3.14 (dd, *J* = 13.6, 6.7 Hz, 1H), 3.05 (m, 2H), 2.82 (s, 3H), 2.21 (m, 1H), 2.08 (m, 1H), 1.96 (m, 2H), 1.71 (m, 1H), 1.41 (s, 9H), 0.92 (t, *J* = 7.5 Hz, 9H), 0.85 (m, 6H).

^{13}C NMR (151 MHz, CDCl$_3$): 173.43, 171.46, 169.73, 169.60, 155.48, 155.45, 136.07, 135.27, 133.72, 133.28, 132.55, 132.02, 130.88, 129.14, 127.40, 127.20, 121.21, 112.70, 110.50, 79.49, 59.95, 55.57, 52.52, 52.49, 51.48, 47.30, 35.85, 30.13, 29.37, 28.47, 26.62, 25.01, 8.27, 6.39.

HRMS (ESI-TOF): calculated for $C_{40}H_{54}N_4NaO_8Si^+$ $[M+Na]^+$: 769.3603, found: 769.3612.

TLC: R_f = 0.3 (1:1 EtOAc : Hexane, $Ce_2(SO_4)_3$ in phosphomolybdic acid).

$[\alpha]^{25}_{D}$: -36.2 (*c* = 0.1, $CHCl_3$)

Key NOESY correlation of micitide core (32)

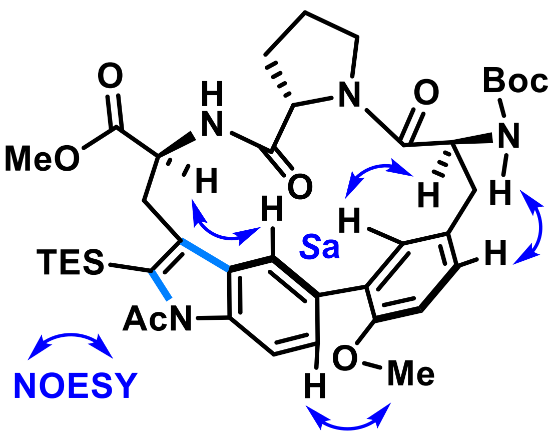

32 ($CDCl_3$, NOESY, 600 MHz)

Skeletal Editing of Micitide Core

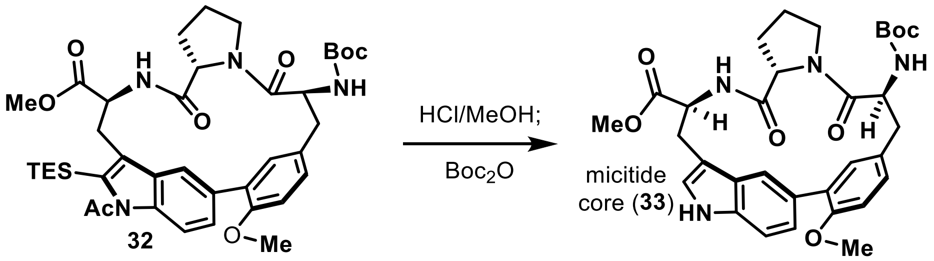

Compound **32** (5.64 g, 7.56 mmol, 1.0 equiv.) was dissolved in MeOH (140 mL, 0.05 M), and SO_2Cl_2 (20 mL) was added dropwise at 0 °C. The reaction mixture was stirred at 25 °C for 2h. Then, the mixture was directly concentrated under reduced pressure to give the crude primary amine. The crude was then dissolved in the THF:sat. $NaHCO_3$ aq. 10:1 (140 mL, 0.05 M). Boc_2O (18.8 g, 86.1 mmol, 11.4 equiv.) was added and the reaction was stirred for 2h at 25 °C. After confirming the full conversion of the primary amine by LC/MS, the reaction was diluted with H_2O (200 mL) and extracted with EtOAc three times. The organic layers were combined, dried over Na_2SO_4, and concentrated under reduced pressure to give the crude. The crude was purified by flash column chromatography to give the compound **33** (4.39 g, 98%).

Compound 33
Physical State: white solid
^{1}H NMR (400 MHz, $CDCl_3$): δ 8.18 (s, 1H), 7.66 (d, J = 9.0 Hz, 1H), 7.59 – 7.43 (m, 2H), 7.36 (s, 1H), 7.26 (d, J = 7.8 Hz, 1H), 6.98 (d, J = 8.3 Hz, 1H), 6.92 (s, 1H), 6.86 (d, J = 8.5 Hz, 1H), 6.84 – 6.78 (m, 1H), 5.28 (d, J = 8.2 Hz, 1H), 4.86 (t, J = 7.9 Hz, 1H), 4.78 (t, J = 5.7 Hz, 1H), 4.51 (d, J = 5.1 Hz, 1H), 3.85 – 3.74 (m, 4H), 3.75 – 3.62 (m, 4H), 3.26 (dd, J = 14.9, 6.3 Hz, 1H), 3.19 – 3.07 (m, 2H), 3.02 (d, J = 15.0 Hz, 1H), 2.13 (d, J = 24.8 Hz, 1H), 2.09 – 2.01 (m, 1H), 2.00 – 1.82 (m, 2H), 1.43 (s, 9H).
^{13}C NMR (101 MHz, $CDCl_3$): δ 173.81, 171.19, 169.83, 155.49, 155.44, 135.58, 134.17, 132.16, 130.90, 129.99, 129.73, 128.49, 126.96, 124.34, 121.53, 121.02, 114.42, 110.61, 109.60, 79.43, 60.06, 55.57, 53.52, 52.40, 51.70, 47.17, 35.81, 31.05, 29.53, 28.44, 24.88.
HRMS (ESI-TOF): calculated for $C_{32}H_{38}N_4NaO_7^+$ $[M+Na]^+$: 613.2638, found: 613.2643.
TLC: R_f = 0.6 (1:0 EtOAc : Hexane, $Ce_2(SO_4)_3$ in phosphomolybdic acid).
$[\alpha]^{21}_D$: +137.0 (c = 1.0, $CHCl_3$)

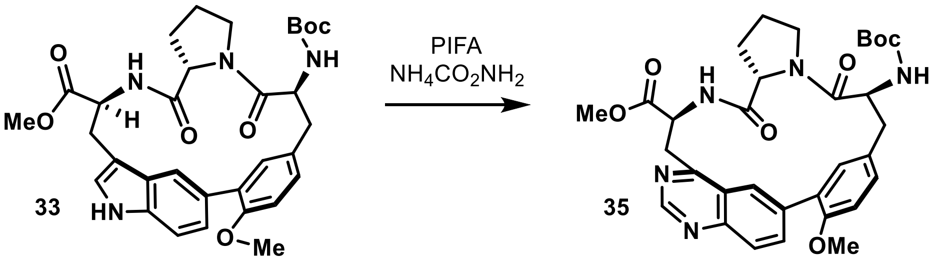

In a culture tube equipped with a stirring bar, the compound **33** (1.77 g, 3.00 mmol, 1.0 eq.) and ammonium carbamate (2.34 g, 30 mmol, 10.0 equiv) were dissolved in MeOH (60 mL, 0.05 M). The resulting solution was cooled to 0 °C, and then PIFA (5.16 g, 12.0 mmol, 4.0 equiv) was added in one portion. The reaction was stirred at 0 °C for 1h and quenched with saturated aq. $Na_2S_2O_3$. The mixture was extracted with EtOAc (5mL) three times. The organic layers were combined and washed with brine and concentrated under reduced pressure to give the crude. The crude was purified by silicagel column chromatography (EtOAc:Hexane:Et_3N 33:66:1 to 100:0:1) to give the title compound **35** (910 mg, 50% yield)

Compound 35
Physical State: white solid
^{1}H NMR (400 MHz, $CDCl_3$): δ 9.19 (s, 1H), 8.36 (dd, *J* = 8.8, 1.8 Hz, 1H), 8.02 (d, *J* = 8.9 Hz, 1H), 7.99 (s, 0H), 7.46 (d, *J* = 2.3 Hz, 1H), 7.12 (dd, *J* = 8.4, 2.2 Hz, 1H), 6.92 (d, *J* = 8.4 Hz, 1H), 6.84 (s, 1H), 5.13 (d, *J* = 6.4 Hz, 1H), 5.02 (t, *J* = 9.5 Hz, 1H), 4.81 (t, *J* = 7.7 Hz, 1H), 4.44 (dd, *J* = 7.9, 4.2 Hz, 1H), 3.90 – 3.78 (m, 3H), 3.84 (s, 3H), 3.76 (s, 3H), 3.54 (dd, *J* = 13.3, 10.0 Hz, 1H), 3.21 (dd, *J* = 13.6, 7.0 Hz, 1H), 3.15 – 3.07 (m, 1H), 2.22 – 2.14 (m, 2H), 2.06 – 1.97 (m, 2H), 1.44 (s, 9H).
^{13}C NMR (101 MHz, $CDCl_3$): δ 172.17, 171.33, 170.13, 167.01, 155.67, 155.38, 153.99, 149.56, 139.26, 136.90, 133.89, 132.25, 128.35, 127.59, 127.41, 125.09, 123.40, 110.76, 79.68, 60.29, 55.63, 52.80, 51.56, 47.43, 40.14, 35.65, 29.51, 28.49, 25.22, 25.11.
HRMS (ESI-TOF): calculated for $C_{32}H_{37}N_5NaO_7^+$ $[M+Na]^+$: 626.2591, found: 626.2692.
TLC: R_f = 0.2 (10:1 EtOAc : Acetone, $Ce_2(SO_4)_3$ in phosphomolybdic acid).
$[\alpha]^{21}_D$: +69.9 (*c* = 1.0, $CHCl_3$)

Key ROESY correlation of quinazomicitide core (35)

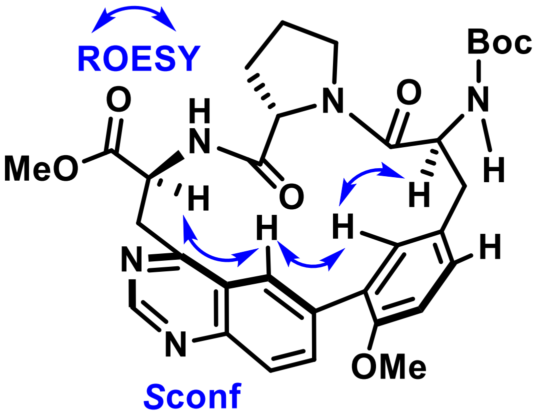

35 (CDCl$_3$, ROESY, 400 MHz)

The conformation of the macrocycle was deduced to be *Sconf*.

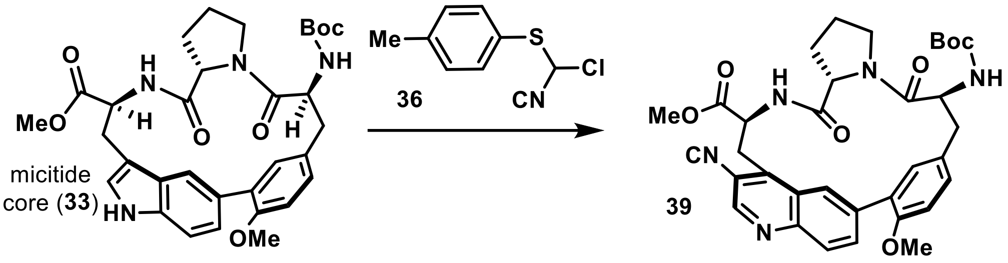

To a solution of compound **33** (500 mg, 847 μmol, 1.0 eq.) and Cs_2CO_3 (966 mg, 2.96 mmol, 3.5 eq.) in THF (15 mL, 0.05 M), sulfenylcarbene precursor **36** (334 mg, 1.69 mmol, 2.0 eq.) was added portion-wise (0.5 equiv. every 1 h) at room temperature, and the resulting mixture was stirred for the next 5 h. The reaction was quenched with saturated aq. $Na_2S_2O_3$, extracted with EtOAc (5mL) three times. The organic layers were combined and washed with brine and concentrated under reduced pressure to give the crude. The crude was purified by silica gel column chromatography (EtOAc:Hexane 1:4 to 2:1) to give the title compound **39** (425 mg, 80% yield)

Compound 39

^{1}H NMR (400 MHz, CDCl$_3$): δ 8.95 (s, 1H), 8.29 (dd, *J* = 8.9, 1.7 Hz, 1H), 8.13 (d, *J* = 8.8 Hz, 1H), 7.93 (d, *J* = 1.8 Hz, 1H), 7.59 – 7.43 (m, 2H), 7.12 (dd, *J* = 8.4, 2.2 Hz, 1H), 6.92 (d, *J* = 8.4 Hz, 1H), 5.29 (d, *J* = 8.5 Hz, 1H), 4.97 (ddd, *J* = 9.8, 6.4, 4.8 Hz, 1H), 4.82 (t, *J* = 7.8 Hz, 1H), 4.40 (dd, *J* = 7.8, 4.2 Hz, 1H), 3.83 (s, 3H), 3.81 (s, 3H), 3.79 – 3.73 (m, 2H), 3.70 – 3.62 (m, 2H), 3.20 (dd, *J* = 13.6, 7.1 Hz, 1H), 3.11 – 3.03 (m, 1H), 2.36 – 1.72 (m, 4H), 1.42 (s, 9H).

^{13}C NMR (101 MHz, CDCl$_3$): δ 171.14, 170.87, 169.01, 154.78, 154.74, 148.90, 148.41, 147.37, 139.26, 134.87, 132.92, 131.82, 131.51, 131.41, 128.35, 128.00, 127.88, 127.26, 126.88, 124.36, 123.41, 116.36, 110.02, 106.58, 78.96, 59.34, 54.88, 52.67, 50.78, 50.53, 46.73, 35.09, 35.00, 28.76, 27.76, 24.34.

HRMS (ESI-TOF): calculated for $C_{34}H_{37}N_5NaO_7^+$ $[M+Na]^+$: 650.2591, found: 650.2591.

TLC: R_f = 0.7 (2:1 EtOAc : Hexane, $Ce_2(SO_4)_3$ in phosphomolybdic acid).

$[\alpha]^{21}_D$: +36.6 (*c* = 1.0, $CHCl_3$)

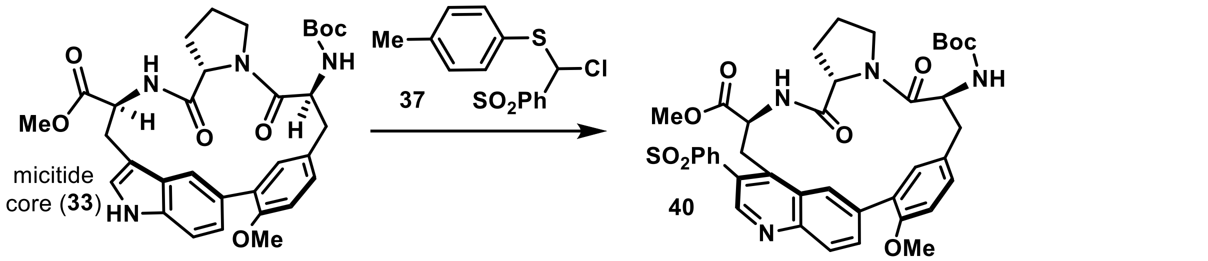

To a solution of compound **33** (1.00 g, 1.69 mmol, 1.0 eq.) and Cs_2CO_3 (2.76 g, 8.47 mmol, 5.0 eq.) in THF (17 mL, 0.1 M), sulfenylcarbene precursor **37** (1.32 g, 4.24 mmol, 2.5 eq.) was added portion-wise (0.5 equiv. every 1 h) at room temperature, and the resulting mixture was stirred for the next 5 h. The reaction was quenched with saturated aq. $Na_2S_2O_3$, extracted with EtOAc (30 mL) three times. The organic layers were combined and washed with brine and concentrated under reduced pressure to give the crude. The crude was purified by silica gel column chromatography (EtOAc:Hexane 1:3 to 2:1) to give the title compound **40** (1.02 mg, 82% yield)

Compound 40

^{1}H NMR (400 MHz, $CDCl_3$): δ 9.40 (s, 1H), 8.28 (dd, *J* = 8.8, 1.6 Hz, 1H), 8.13 (d, *J* = 8.8 Hz, 1H), 8.03 (s, 0H), 7.83 (d, *J* = 8.0 Hz, 1H), 7.82 (s, 1H), 7.55 – 7.42 (m, 4H), 7.31 (d, *J* = 10.2 Hz, 1H), 7.12 (dd, *J* = 8.4, 2.2 Hz, 1H), 6.87 (d, *J* = 8.4 Hz, 1H), 5.22 (d, *J* = 8.7 Hz, 1H), 5.04 (t, *J* = 9.8 Hz, 1H), 4.82 (t, *J* = 7.8 Hz, 1H), 4.59 (dd, *J* = 13.3, 10.9 Hz, 1H), 4.39 (dd, *J* = 7.8, 4.8 Hz, 1H), 3.78 (s, 3H), 3.75 (s, 3H), 3.74 – 3.64 (m, 2H), 3.53 (d, *J* = 13.3 Hz, 1H), 3.18 (dd, *J* = 13.5, 7.4 Hz, 1H), 3.07 (d, *J* = 13.5 Hz, 1H), 2.14 – 1.99 (m, 2H), 1.96 – 1.81 (m, 2H), 1.39 (s, 9H).

^{13}C NMR (101 MHz, $CDCl_3$): δ 171.39, 171.03, 169.91, 155.46, 155.32, 148.74, 147.92, 145.07, 142.15, 139.76, 135.42, 133.54, 132.27, 132.20, 129.52, 128.92, 128.31, 127.50, 127.06, 126.11, 124.91, 110.56, 79.58, 60.18, 55.49, 52.76, 52.51, 51.47, 47.41, 35.86, 32.10, 29.19, 28.36, 25.06.

HRMS (ESI-TOF): calculated for $C_{39}H_{42}N_4NaO_9S^+$ $[M+Na]^+$: 765.2570, found: 765.2573.

TLC: R_f = 0.7 (2:1 EtOAc : Hexane, $Ce_2(SO_4)_3$ in phosphomolybdic acid).

$[\alpha]^{21}_D$: −12.1 (*c* = 0.5, $CHCl_3$)

Sidechain Elongation of the Micitide Core Analogues (General Procedure C)

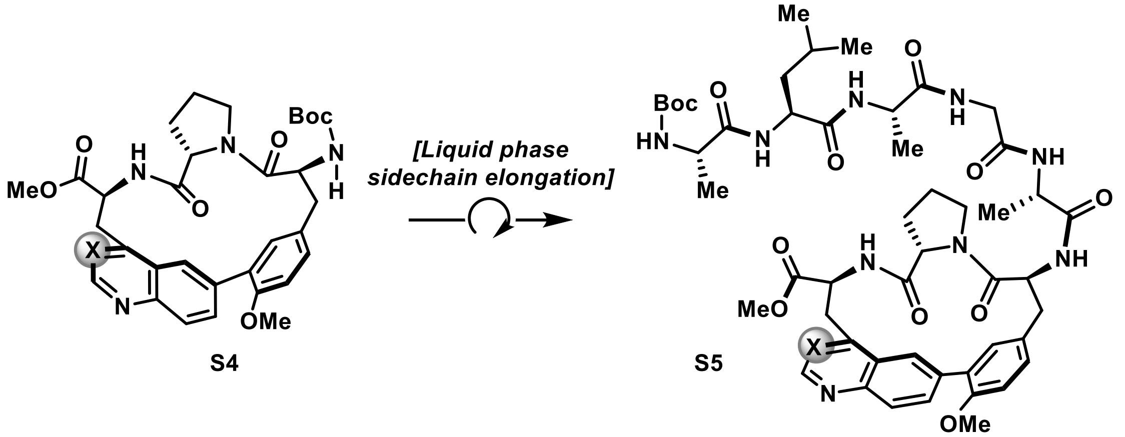

1) Elongation towards the N-terminal

5 amino acids attached to the *N*-terminal of the micitide core was introduced by repeating the following procedure; The Boc-protected amine (**S4**, 1.0 eq.) was dissolved in DCM (0.1 M). 4.0 M HCl in 1,4-dioxane (0.4 M) was added ddropwise. The reaction mixture was stirred at 25 °C for 2 h. The solvent was removed

under reduced pressure to give the crude. The crude was dissolved in DMF (0.1 M), corresponding carboxylic acid (3.0 eq.), DIPEA (6.0 eq.), and HATU (2.0 eq.) were added sequentially. The reaction was stirred at 25 °C for 2 h and quenched by saturated *aq.* NH_4Cl and extracted with EtOAc three times. The organic layers were combined, washed with saturated *aq.* $NaHCO_3$ three times. The organic layers were concentrated under reduced pressure to give the coupled product. Following this procedure, L-Ala-OH, Gly-OH, L-Ala-OH, L-Leu-OH, and L-Ala-OH were introduced sequentially.

2) Hydrolysis and introduction of NH_2-His(DNP)-OMe

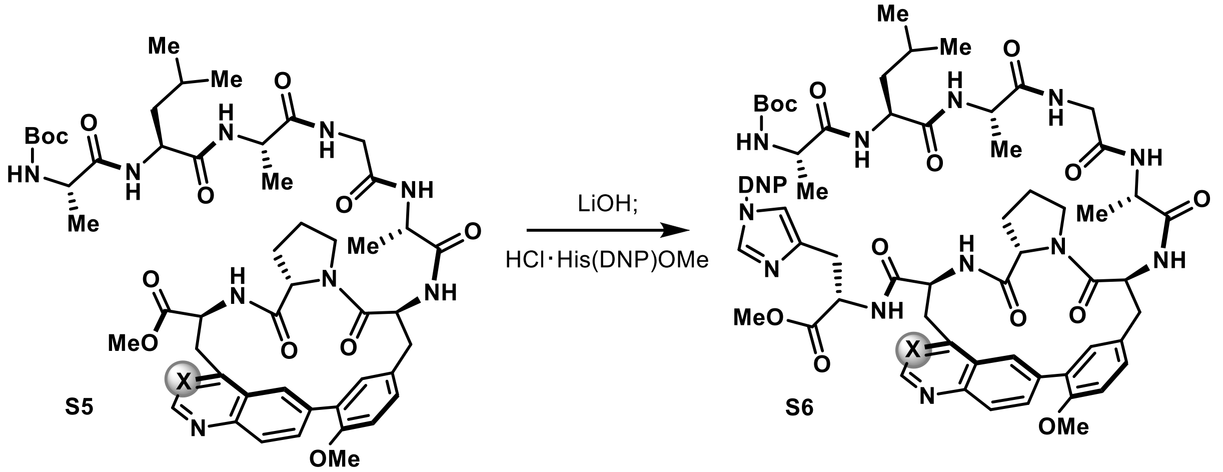

The crude methyl ester **S5** was dissolved in THF/MeOH/H_2O = 1/1/1 (0.1 M). LiOH·H_2O (6 eq.) was added. The reaction was stirred at 25 °C for 0.5 h and acidified with aq. 0.5 M HCl. The solvent was removed under reduced pressure to give the crude. The crude was then dissolved in DMF (0.1 M). ClH_3N-His(DNP)-OMe (1.5 eq.), DIPEA (6.0 eq.), and HATU (2 eq.) were added sequentially. The reaction was stirred at 25 °C for 2 h and quenched by saturated *aq.* NH_4Cl, extracted with EtOAc three times. Organic layers were concentrated under reduced pressure and purified by silica gel chromatography to give the coupled product **S6**.

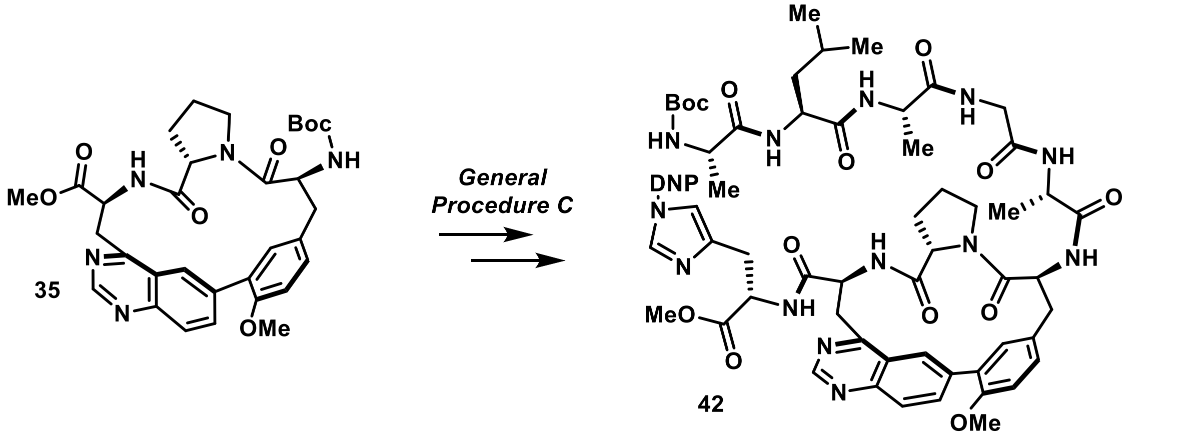

On 1.49 mmol scale, **General Procedure C** was followed with quinazoline containing micitide core **35**. Purification by silica gel column chromatography (EtOAc:Hexane 4:1 to Acetone:EtOAc 1:20) gave the title compound **42**. (1.56 g, 81% yield from **35**)

Compound 42

^{1}H NMR (400 MHz, CD_3OD): δ ^{1}H NMR (400 MHz, MeOD) δ 9.03 (s, 1H), 8.87 (s, 1H), 8.58 (s, 1H), 8.38 (t, *J* = 8.4 Hz, 1H), 8.11 (d, *J* = 6.8 Hz, 1H), 7.91 (s, 2H), 7.82 (t, *J* = 3.7 Hz, 1H), 7.46 (d, *J* = 6.4 Hz, 1H), 7.18 (d, *J* = 7.8 Hz, 2H), 7.13 (s, 1H), 7.05 (t, *J* = 6.4 Hz, 1H), 5.05 (s, 1H), 4.90 – 4.79 (m, 3H), 4.59 – 4.52 (m, 1H), 4.39 (dd, *J* = 14.6, 7.4 Hz, 2H), 4.28 (d, *J* = 12.9 Hz, 1H), 4.09 (q, *J* = 7.2 Hz, 1H), 3.85 (s, 3H), 3.81

(d, J = 62.7 Hz, 5H), 3.79 (s, 3H), 3.69 – 3.53 (m, 1H), 3.28 – 3.07 (m, 4H), 1.91 – 1.84 (m, 2H), 1.74 – 1.65 (m, 2H), 1.63 – 1.56 (m, 2H), 1.44 (s, 9H), 1.38 (d, J = 7.1 Hz, 3H), 1.41 – 1.32 (m, 1H), 1.30 (d, J = 6.4 Hz, 6H), 0.93 (d, J = 6.9 Hz, 3H), 0.90 (d, J = 6.9 Hz, 3H).
^{13}C NMR (101 MHz, $CDCl_3$): δ 174.27, 173.28, 172.70, 172.19, 171.55, 170.78, 170.21, 169.32, 168.56, 168.23, 155.92, 155.00, 152.47, 148.14, 146.56, 143.58, 138.92, 137.81, 136.76, 136.55, 133.96, 132.85, 131.97, 128.99, 128.53, 127.53, 127.01, 126.78, 125.51, 124.85, 122.55, 120.38, 117.38, 110.24, 78.69, 77.18, 69.50, 65.62, 64.75, 62.25, 54.13, 51.82, 51.20, 51.03, 50.30, 49.78, 49.00, 48.52, 41.46, 39.59, 38.39, 38.17, 34.09, 32.72, 31.05, 29.57, 28.84, 28.74, 28.46, 28.06, 26.70, 24.05, 23.74, 23.52, 22.88, 22.01, 21.72, 21.49, 19.92, 16.07, 15.98, 15.57, 12.44, 12.38, 9.36.
HRMS (ESI-TOF): calculated for $C_{61}H_{75}N_{15}NaO_{17}^+$ $[M+Na]^+$: 1312.5363, found: 1312.5363.
TLC: R_f = 0.2 (1:10 MeOH:EtOAc, $Ce_2(SO_4)_3$ in phosphomolybdic acid).
$[\alpha]^{21}_D$: +124.2 (c = 0.5, MeOH)

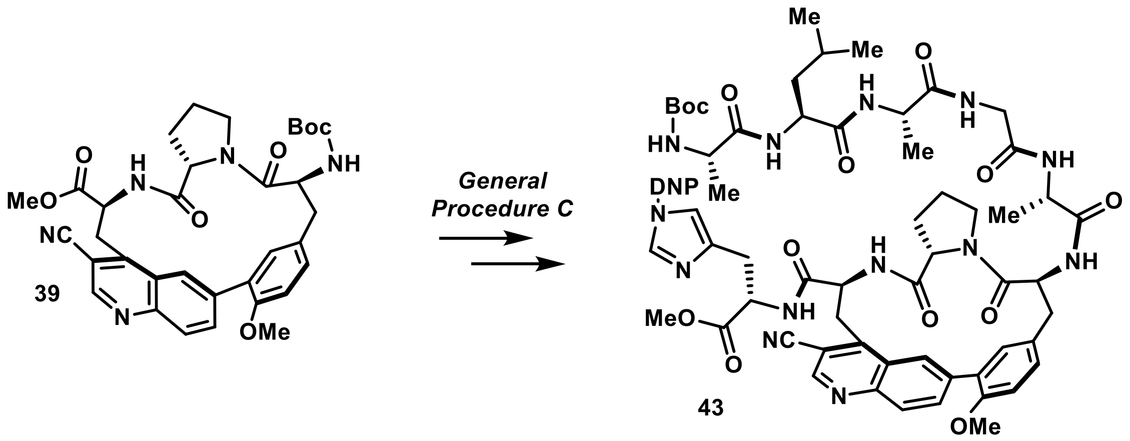

On 0.960 mmol scale, **General Procedure C** was followed with micitide core **39**. Purification by silica gel column chromatography (EtOAc:Hexane 4:1 to Acetone:EtOAc 1:20) gave the title compound **43**. (903 mg, 72% yield from **43**)

Compound 43
^{1}H NMR (400 MHz, DMSO-*d6*): δ 9.23 (d, J = 9.5 Hz, 1H), 8.98 (s, 1H), 8.91 (d, J = 2.6 Hz, 1H), 8.62 (dd, J = 8.8, 2.6 Hz, 1H), 8.31 (d, J = 7.9 Hz, 1H), 8.26 (dd, J = 8.8, 1.6 Hz, 1H), 8.18 – 8.14 (m, 1H), 8.12 – 8.05 (m, 2H), 8.01 (d, J = 6.8 Hz, 1H), 7.98 – 7.92 (m, 2H), 7.89 (d, J = 8.8 Hz, 1H), 7.79 (d, J = 8.2 Hz, 1H), 7.51 (br, J = 2.1 Hz, 1H), 7.39 (d, J = 7.4 Hz, 1H), 7.10 – 7.07 (m, 2H), 7.02 (d, J = 8.5 Hz, 1H), 6.97 (d, J = 7.4 Hz, 1H), 4.85 – 4.75 (m, 2H), 4.65 (q, J = 6.9 Hz, 1H), 4.48 (dd, J = 8.5, 4.6 Hz, 1H), 4.36 – 4.14 (m, 4H), 4.00 – 3.89 (m, 1H), 3.78 (s, 3H), 3.76 – 3.63 (m, 5H), 3.62 (s, 3H), 3.21 – 3.12 (m, 1H), 2.95 – 2.81 (m, 3H), 2.11 – 1.91 (m, 1H), 1.83 – 1.73 (m, 1H), 1.64 – 1.57 (m, 2H), 1.48 – 1.40 (m, 2H), 1.36 (s, 9H), 1.23 (d, J = 5.1 Hz, 3H), 1.22 – 1.20 (m, 4H), 1.15 (d, J = 7.1 Hz, 3H), 0.85 (d, J = 6.4 Hz, 3H), 0.82 (d, J = 6.1 Hz, 3H).
^{13}C NMR (101 MHz, DMSO-*d6*): δ 172.59, 172.34, 171.96, 171.75, 171.61, 171.12, 170.90, 169.55, 169.44, 168.24, 167.67, 155.01, 154.81, 149.74, 149.34, 147.04, 146.05, 143.20, 138.72, 138.61, 138.53, 136.92, 136.89, 134.37, 134.31, 133.09, 132.28, 129.27, 129.24, 128.54, 128.51, 128.29, 127.79, 126.91, 124.90, 124.22, 121.10, 117.07, 116.74, 116.44, 110.98, 106.95, 78.05, 69.58, 65.57, 58.77, 55.35, 51.80, 51.79, 51.70, 51.64, 50.60, 50.53, 49.55, 48.24, 46.62, 41.69, 34.61, 33.07, 30.50, 29.77, 29.61, 29.55, 28.92, 28.12, 27.93, 24.33, 23.79, 23.05, 22.94, 22.21, 22.07, 21.27, 17.90, 17.69, 17.61.
HRMS (ESI-TOF): calculated for $C_{63}H_{75}N_{15}NaO_{17}^+$ $[M+Na]^+$: 1336.5363, found: 1336.5363.

TLC: R_f = 0.7 (1:10 MeOH:EtOAc, $Ce_2(SO_4)_3$ in phosphomolybdic acid).
$[\alpha]^{21}_D$: +52.2 (*c* = 1.0, MeOH)

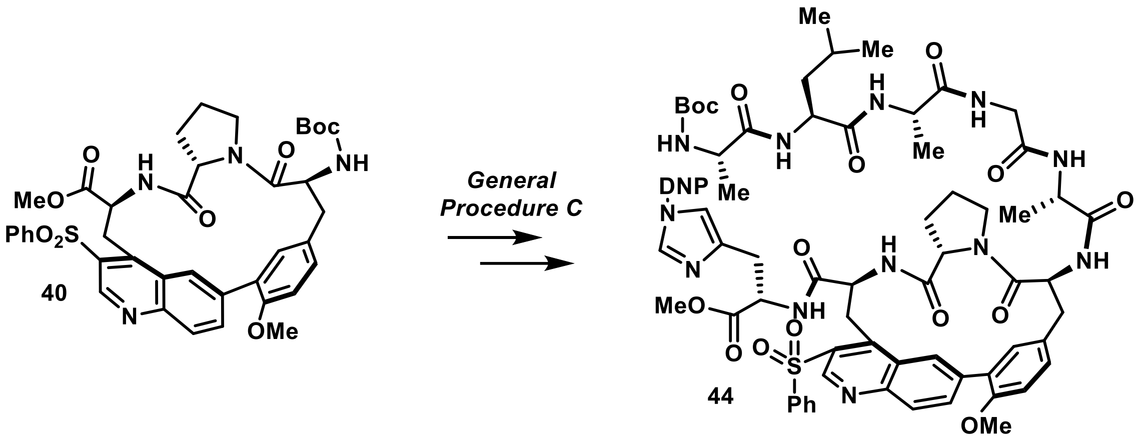

On 0.960 mmol scale, **General Procedure C** was followed with micitide core **40**. Purification by silica gel column chromatography (EtOAc:Hexane 4:1 to Acetone:EtOAc 1:20) gave the title compound **44**. (785 mg, 40% yield from **40**)

Compound 44

^{1}H NMR (400 MHz, CD_3OD): δ 8.95 (s, 2H), 8.69 – 8.60 (m, 3H), 8.00 – 7.84 (m, 10H), 7.24 – 7.12 (m, 2H), 4.79 – 4.72 (m, 2H), 4.45 – 4.12 (m, 4H), 4.12 – 3.99 (m, 1H), 3.87 – 3.76 (m, 3H), 3.73 (s, 6H), 3.69 – 3.66 (m, 3H), 3.25 – 3.11 (m, 2H), 3.05 – 2.91 (m, 4H), 1.72 – 1.51 (m, 6H), 1.45 (s, 9H), 1.37 – 1.26 (m, 10H), 0.98 – 0.87 (m, 6H).

^{13}C NMR (101 MHz, CD_3OD): δ 174.68, 174.49, 172.79, 172.67, 172.13, 172.11, 171.34, 169.44, 160.28, 156.02, 155.05, 146.61, 146.52, 143.75, 143.62, 143.58, 137.90, 137.79, 136.72, 136.41, 134.14, 134.02, 129.38, 129.20, 129.09, 128.78, 127.75, 127.60, 126.80, 120.46, 120.38, 118.12, 117.45, 117.33, 110.76, 78.80, 68.96, 65.64, 56.50, 54.14, 52.03, 51.63, 51.47, 50.88, 39.34, 38.24, 38.15, 36.68, 34.85, 32.73, 31.05, 29.56, 29.42, 29.36, 28.76, 28.05, 26.71, 24.27, 23.78, 23.51, 22.87, 22.00, 21.51, 21.47, 20.40, 19.92, 19.44, 19.22, 18.44, 16.34, 15.89, 15.44.

HRMS (ESI-TOF): calculated for $C_{68}H_{81}N_{14}O_{19}S^+$ $[M+H]^+$: 1429.5523, found: 1429.5524.

TLC: R_f = 0.6 (1:10 MeOH:EtOAc, $Ce_2(SO_4)_3$ in phosphomolybdic acid).
$[\alpha]^{21}_D$: +20.0 (*c* = 1.0, MeOH)

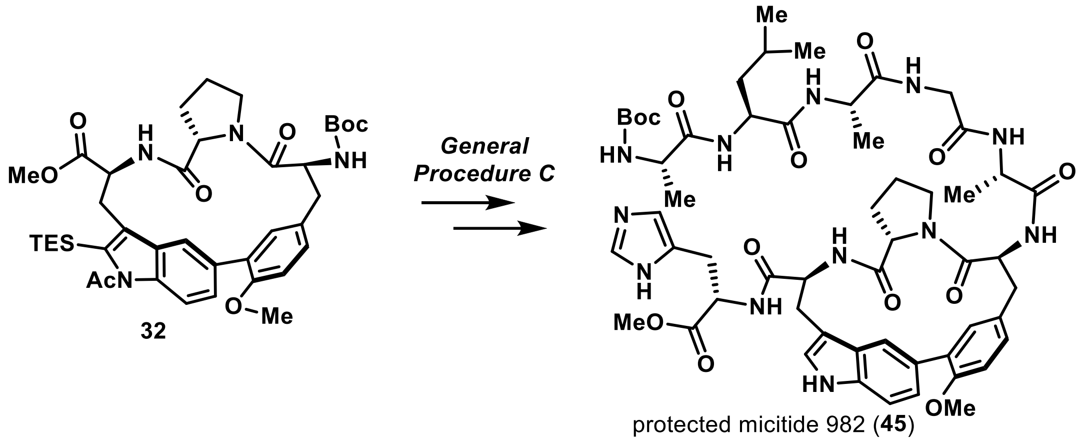

On 4.98 mmol scale, **General Procedure C** was followed with micitide core **40**. In case of micitide core **32**, histidine methyl ester dihydrochloride was used instead of ClH_3N-His(DNP)-OMe, using the same amide coupling conditions. purification by silica gel column chromatography (EtOAc:Hexane 4:1 to MeOH:EtOAc 1:4) gave the title compound **44**. (1.67 g, 31% yield from **32**) The characteristics data were identical to those previously reported by our group, obtained via a different synthetic route.[2]

Note: Boc removal after the introduction of Gly was conducted using the following conditions to remove the acetyl group on the indole ring:

The Boc-protected amine was dissolved in MeOH (150 mL). $SOCl_2$ (20.5 mL) was added dropwise at 0 °C. The reaction mixture was stirred at rt for 1 h. The solvent was removed under reduced pressure to give the ammonium salt.

Protected micitide 982 (45)

Physical State: white solid

^{1}H NMR (600 MHz, CD_3OD): δ 7.91 – 6.80 (m, 8H), 4.67 (dd, J = 8.2, 5.0 Hz, 1H), 4.38 – 4.33 (m, 2H), 4.28 – 4.22 (m, 2H), 4.08 – 4.02 (m, 2H), 3.93 – 3.75 (m, 6H), 3.71 (s, 6H), 3.25 – 3.15 (m, 2H), 3.13 – 3.04 (m, 2H), 1.74 – 1.67 (m, 2H), 1.64 – 1.54 (m, 2H), 1.44 (s, 9H), 1.33 – 1.28 (m, 10H), 0.95 (d, J = 6.6 Hz, 6H).

^{13}C NMR (151 MHz, CD_3OD): 176.62, 176.44, 175.64, 175.54, 174.99, 174.88, 172.27, 171.46, 171.36, 157.97, 156.97, 135.55, 135.52, 132.14, 118.84, 118.79, 80.72, 53.56, 53.51, 53.33, 53.06, 52.11, 51.97, 51.33, 51.11, 50.56, 43.54, 41.37, 41.30, 41.17, 28.72, 28.53, 28.36, 25.82, 25.74, 23.46, 21.94, 21.87, 17.99, 17.84, 17.63, 17.42, 9.25.

HRMS (ESI-TOF): calculated for $C_{55}H_{75}N_{12}O_{13}^+$ $[M+H]^+$: 1111.5571, found: 1111.5576.

TLC: R_f = 0.5 (EtOAc:*n*BuOH:AcOH:H_2O 9:9:1:1, $Ce_2(SO_4)_3$ in phosphomolybdic acid).

$[\alpha]^{25}_D$: −27.8 (c = 0.1, MeOH)

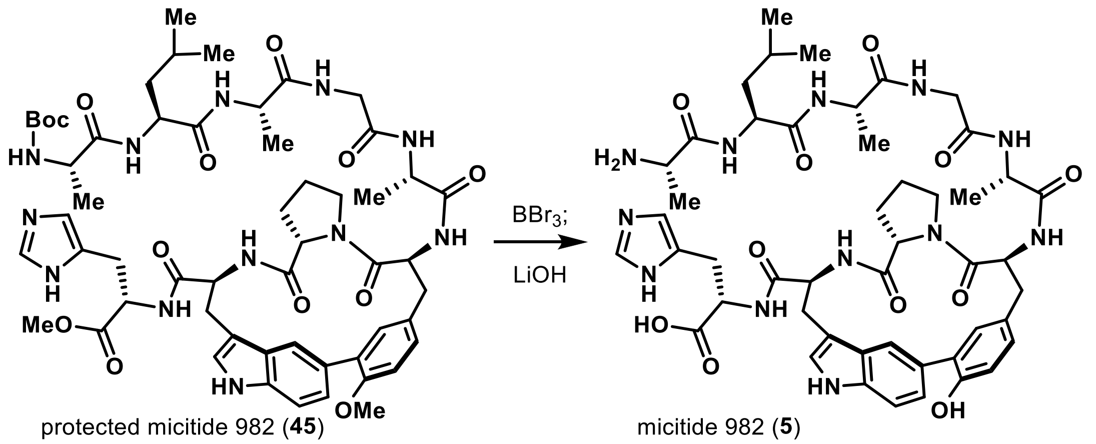

Compound **45** (80.0 mg, 72.0 μml, 1.0 eq.) was dissolved in DCM (15 mL, 0.05 M), BBr_3 (1M in DCM, 1.4 mL, 1.4 mmol, 20 eq.) was added dropwise at 0 °C under N_2, the reaction was stirred at 25 °C for 1 h, checked by LC-MS and quenched by MeOH dropwise at 0 °C, then it was concentrated under reduced pressure to give the residue. The residue was dissolved in THF/H_2O/MeOH = 1:1:1 (6.0 mL), LiOH (200 mg, 4.77 mmol, 66.2 eq.) was added, checked by LC-MS, the reaction was stirred at 25 °C for 2 h, acidified with 0.5 M HCl to adjust the pH = 3, the solvents was removed under reduced pressure and the residue was purified by C18 silica gel (manual reverse-phase silica gel) using H_2O followed by MeOH/H_2O/TFA = 40:60:1 as the mobile phase to give micitide 982 (**1**) (57.3 mg, 81% yield). The characterization data were identical to those reported in the isolation paper by Li *et al.*[1] and to those previously reported by our group.[2]

Compound 5 (micitide 982)
Physical State: white solid
^{1}H NMR (600 MHz, DMSO-*d6*): δ 10.70 (brs, 1H), 9.15 (s, 1H), 8.86 (d, *J* = 9.4 Hz, 1H), 8.69 (m, 1H), 8.43 (d, *J* = 7.9 Hz, 1H), 8.14 (s, 1H), 8.14 (d, *J* = 4.0 Hz, 1H), 8.07 (d, *J* = 7.1 Hz, 1H), 7.76 (d, *J* = 7.9 Hz, 1H), 7.65 (dd, *J* = 8.7, 1.2 Hz, 1H), 7.35 (s, 1H), 7.30 (s, 1H), 7.28 (d, *J* = 8.2 Hz, 1H), 7.22 (brs, 1H), 7.08 (d, *J* = 7.4 Hz, 1H), 6.99 (d, *J* = 2.2 Hz, 1H), 6.74 (m, 1H), 6.74 (m, 1H), 4.76 (t, *J* = 7.6 Hz, 1H), 4.63 (dd, *J* = 7.7, 3.4 Hz, 1H), 4.57 (m, 1H), 4.52 (m, 1H), 4.35 (m, 1H), 4.25 (qd, *J* = 6.5, 2.7 Hz, 1H), 4.25 (qd, *J* = 6.5, 2.7 Hz, 1H), 3.86 (q, *J* = 7.3 Hz, 1H), 3.79 (dd, *J* = 15.4, 6.1 Hz, 2H), 3.66 (m, 2H), 3.61 (dd, *J* = 15.5, 6.7 Hz, 1H), 3.22 (dd, *J* = 15.2, 6.1 Hz, 1H), 3.08 − 2.99 (m, 2H), 3.07 − 2.85 (m, 2H), 2.82 (m, 1H), 2.05 − 1.68 (m, 2H), 2.00 − 1.91 (m, 2H), 1.64 (m, 1H), 1.48 (m, 2H), 1.34 (d, *J* = 6.9 Hz, 3H), 1.23 (d, *J* = 7.3 Hz, 3H), 1.23 (d, *J* = 7.3 Hz, 3H), 0.90 (d, *J* = 6.3 Hz, 3H), 0.87 (d, *J* = 6.4 Hz, 3H),
^{13}C NMR (151 MHz, DMSO-*d6*): 173.0, 172.5, 172.3, 172, 171.9, 171.6, 169.8, 168.9, 168.3, 153.2, 135.7, 134.1, 133.5, 130.1, 130, 129.5, 128.2, 127.1, 126.0, 123.6, 122.7, 120.1, 116.4, 115.5, 113.8, 110.1, 59.1, 54.0, 51.5, 51.3, 51.0, 49.0, 48.8, 48.4, 47, 42.2, 40.7, 35.1, 31.2, 29.7, 26.5, 24.6, 24.4, 23.4, 21.7, 18.2, 18.2, 17.5.
HRMS (ESI-TOF): calculated for $C_{48}H_{62}N_{12}O_{11}^+$ $[M+2H]^{2+}$: 492.2409, found: 492.2425.
$[\alpha]^{25}_D$: −54.4 (*c* = 0.1, MeOH)

Synthesis of Scabrirubin CB-4

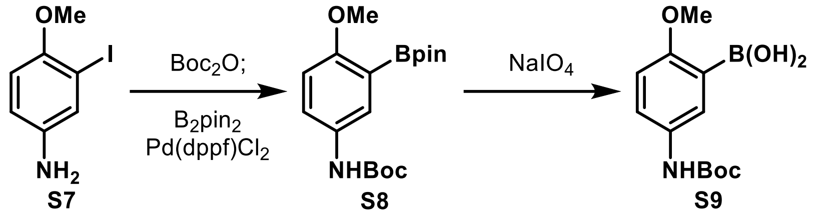

A mixture of aniline **S7** (15.0 g, 60.2 mmol, 1.0 eq.) and Boc_2O (19.8 g, 90.4 mmol, 1.5 eq.) was heated at 80 °C under vacuum. After completion of the reaction, confirmed by TLC, the mixture was cooled down to 25 °C. To the crude, KOAc (17.7 g, 181 mmol, 3.0 equiv.), B_2pin_2 (18.3 g, 72.2 mmol, 1.2 equiv.) and Pd(dppf)Cl_2 (2.20 g, 3.01 mmol, 0.05 equiv.) were added. The flask was evacuated and backfilled with N_2 three times. Dry DMSO (200 mL, 0.2 M) was added, and the reaction was stirred in a preheated oil bath set to 90 °C. After 6h, the reaction was cooled to 25 °C. The crude mixture was then diluted with water (1000 mL) and extracted with EtOAc (500 mL) three times. The organic phase was combined, washed with brine (1000 mL) three times, dried over Na_2SO_4, and evaporated under vacuum. The crude mixture was filtered through a pad of silica gel (EtOAc:Hexane 1:3) to give crude **S8**. The crude **S8** was dissolved in acetone/water = 1:1 (300 mL, 0.2 M), then $NaIO_4$ (38.6 g, 181 mmol, 3.0 equiv.) and NH_4OAc (27.8 g, 361 mmol, 6.0 equiv.) were added. The reaction was stirred at 25 °C for 15h and quenched with 1N HCl aq. (500 mL). The aqueous phase was extracted with EtOAc (500 mL) three times. The organic phase was combined, dried over Na_2SO_4, and evaporated under vacuum. The residue was triturated with EtOAc:Hexane 1:2 and dried under vacuum to yield boronic acid **S9** as a white solid. (9.76 g, 61% from **S7**)

Compound S9
Physical State: white solid
^{1}H NMR (600 MHz, CD_3OD, mixture of rotamers, A:B = 3:2): δ 7.57 (s, 1H, **B**), 7.45 (d, J = 7.2 Hz, 1H, **B**), 7.38 (d, J = 8.8 Hz, 1H, **A**), 7.19 (s, 1H, **A**), 6.91 (d, J = 8.6 Hz, 1H, **B**), 6.88 (d, J = 8.8 Hz, 1H, **A**), 3.85 (s, 3H, **B**), 3.77 (s, 3H, **A**), 1.50 (s, 9H).
^{13}C NMR (151 MHz, CD_3OD, mixture of rotamers, A:B = 3:2): δ 160.23, 157.39, 154.45, 132.17, 132.11, 110.08, 109.89, 79.28, 54.64, 27.42.
HRMS (ESI-TOF): calculated for $C_{12}H_{18}BNNaO_5^+$ $[M+Na]^+$: 290.1176, found: 290.1175.
TLC: R_f = 0.6 (2:1 EtOAc : Hexane, $Ce_2(SO_4)_3$ in phosphomolybdic acid).

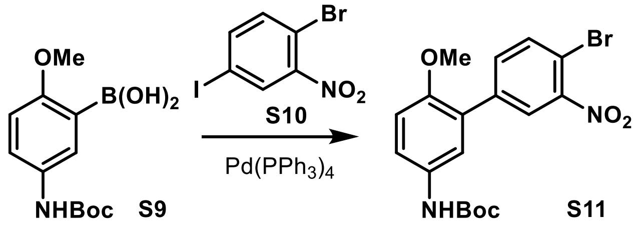

To a solution of compound **S9** (9.76 g, 36.5 mmol, 1.0 equiv.) in Dioxane:H_2O 2:1 (180 mL, 0.2 M) was added aryl iodide **S10** (14.3 g, 43.8 mmol, 1.2 equiv.), K_2CO_3 (25.2 g, 183 mmol, 5.0 equiv.) and Pd(PPh_3)$_4$ (2.11 g, 1.83 mmol, 0.05 equiv.). The reaction was stirred at 80 °C for 6 h, then the reaction mixture was allowed to cool to 25 °C, diluted with water, extracted with EtOAc three times, and the combined organic layers were washed with brine, dried over Na_2SO_4, and evaporated under vacuum. The crude was purified by silica gel chromatography (hexane:EtOAc 10:1 to 2:1) to give compound **S11** (10.4 g, 67% yield).

Compound S11

Physical State: pale yellow solid

^{1}H NMR (400 MHz, CDCl$_3$): δ 8.02 (d, J = 2.1 Hz, 1H), 7.72 (d, J = 8.3 Hz, 1H), 7.57 (dd, J = 8.3, 2.1 Hz, 1H), 7.39 (s, 1H), 7.31 (d, J = 8.4 Hz, 1H), 6.92 (d, J = 8.8 Hz, 1H), 6.54 (s, 1H), 3.79 (s, 3H), 1.51 (s, 9H).

^{13}C NMR (101 MHz, CDCl$_3$): δ 152.49, 151.62, 148.82, 138.22, 133.86, 133.57, 131.34, 126.48, 125.94, 120.75, 120.09, 112.01, 111.37, 79.97, 55.30, 27.73.

HRMS (CI-TOF): calculated for $C_{18}H_{20}BrN_2O_5^+$ [M+H]$^+$: 423.0556, found: 423.0557.

TLC: R$_f$ = 0.4 (1:9 EtOAc : Hexane, $Ce_2(SO_4)_3$ in phosphomolybdic acid).

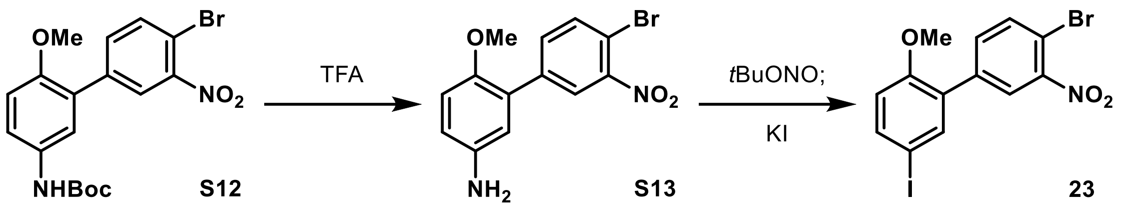

To a solution of **S12** (10.4 g, 24.6 mmol, 1.0 equiv.) in DCM (120 mL, 0.2 M), TFA (30 mL, 0.8 M) was added. The reaction mixture was stirred at 25 °C for 1 h. The solvent and TFA were removed under reduced pressure to give crude. To a solution of the crude and *p*TsOH·H_2O (18.7 g, 98.2 mmol, 4.0 equiv.) in MeCN (130 mL, 0.2 M), *t*BuONO (4.4 mL, 36.8 mmol, 1.5 equiv.) was added dropwise at 0 °C. The reaction was stirred at 0 °C for 1 h, then KI (24.4 g, 14.7 mmol, 6.0 equiv.) in H_2O (30 mL) was added dropwise at 0 °C. The reaction mixture was stirred at 25 °C for 4 h, quenched by saturated *aq.* $NaHCO_3$ and saturated *aq.* $Na_2S_2O_3$, extracted with DCM three times. The organic layers were combined and washed with saturated *aq.* $Na_2S_2O_3$. Organic layers were concentrated under reduced pressure and purified by silica gel chromatography (hexane:EtOAc 10:1 to 2:1) to give compound **23** (10.6 g, 76%).

Compound 23

Physical State: colorless solid

^{1}H NMR (400 MHz, CDCl$_3$): δ 7.99 (d, J = 2.1 Hz, 1H), 7.75 (d, J = 8.3 Hz, 1H), 7.66 (dd, J = 8.7, 2.3 Hz, 1H), 7.58 (d, J = 2.3 Hz, 1H), 7.54 (dd, J = 8.4, 2.1 Hz, 1H), 6.77 (d, J = 8.7 Hz, 1H), 3.81 (s, 3H).

^{13}C NMR (151 MHz, CDCl$_3$): δ 152.49, 151.62, 148.82, 138.22, 133.86, 133.57, 131.34, 126.48, 125.94, 120.75, 120.09, 112.01, 111.37, 79.97, 55.30, 27.73.

HRMS (CI-TOF): calculated for $C_{13}H_9BrINO_3^+$ [M+H]$^+$: 433.8889, found: 433.8890.

TLC: R$_f$ = 0.5 (1:3 EtOAc : Hexane, $Ce_2(SO_4)_3$ in phosphomolybdic acid).

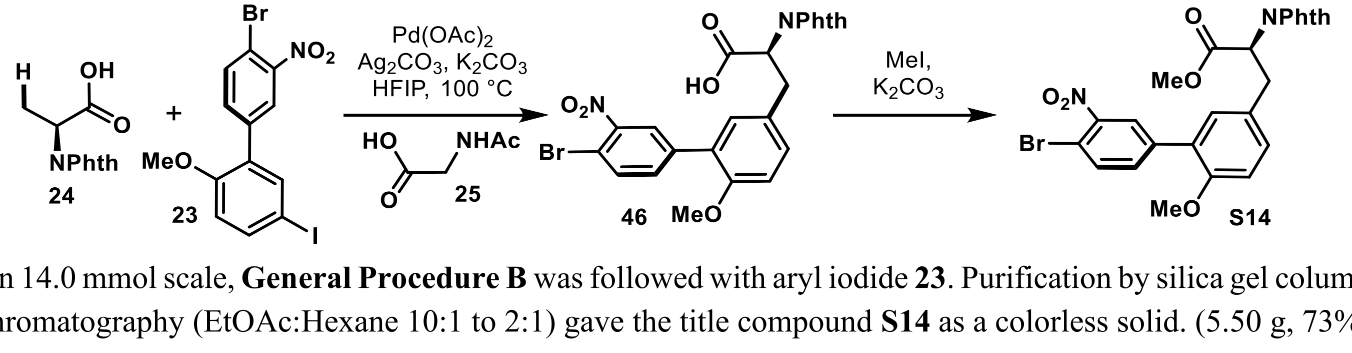

On 14.0 mmol scale, **General Procedure B** was followed with aryl iodide **23**. Purification by silica gel column chromatography (EtOAc:Hexane 10:1 to 2:1) gave the title compound **S14** as a colorless solid. (5.50 g, 73%)

Compound S14

^{1}H NMR (400 MHz, CDCl$_3$): δ 7.80 (dd, J = 5.5, 3.1 Hz, 2H), 7.72 (dd, J = 5.6, 3.0 Hz, 3H), 7.64 (d, J = 8.4 Hz, 1H), 7.36 (dd, J = 8.3, 2.1 Hz, 1H), 7.20 (dd, J = 8.4, 2.3 Hz, 1H), 7.03 (d, J = 2.3 Hz, 1H), 6.83 (d, J = 8.4 Hz, 1H), 5.14 (dd, J = 11.1, 5.4 Hz, 1H), 3.79 (s, 3H), 3.73 (s, 3H), 3.62 – 3.47 (m, 2H).

^{13}C NMR (101 MHz, CDCl$_3$): δ 168.58, 166.83, 154.45, 138.19, 133.71, 133.35, 130.84, 130.12, 129.95, 128.63, 126.16, 125.67, 122.95, 111.80, 110.92, 54.92, 52.56, 52.36, 33.07.

HRMS (CI-TOF): calculated for $C_{25}H_{20}BrN_2O_7^+$ [M+Na]$^+$: 539.0454, found: 539.0449.

TLC: R$_f$ = 0.7 (1:1 EtOAc : Hexane, $Ce_2(SO_4)_3$ in phosphomolybdic acid).

[α]^{21}D: −108.5 (c = 1.0, $CHCl_3$)

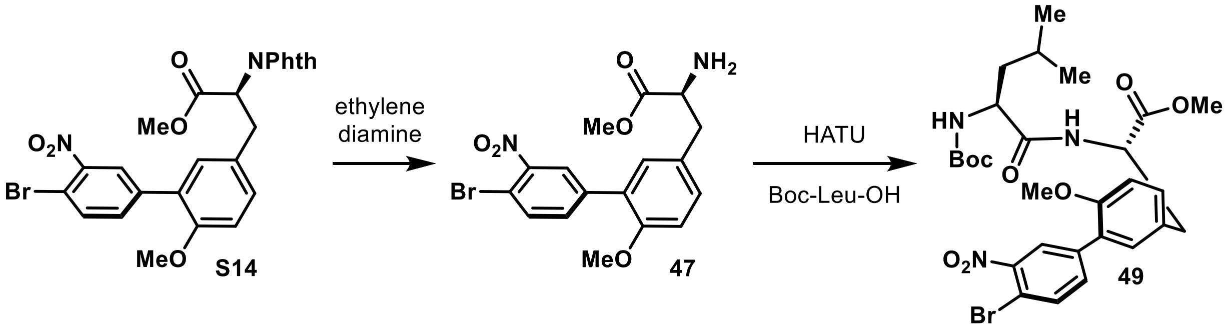

Compound **S14** (5.50 g, 10.2 mmol, 1.0 eq.) was dissolved in n-BuOH (70 mL, 0.2 M), and ethylenediamine (5.8 mL, 102 mmol, 10 eq.) was added. The reaction was stirred at 25 °C for 15 h. The reaction mixture was diluted with toluene (15 mL) and directly concentrated under reduced pressure to give the crude. To the crude, Boc-Leu-OH (16.2 g, 70.0 mmol, 6.9 eq.), and DIPEA (15 mL, 84 mmol, 8.2 eq.), was added HATU (26.6 g, 70 mmol, 6.9 eq.). The reaction mixture was stirred at for 3 h and quenched with *aq.* 0.5 M HCl, extracted with EtOAc three times. The organic layers were combined and washed with saturated *aq.* NaCl. The organic layers were concentrated under reduced pressure to give and purified by silica gel chromatography (EtOAc:Hexane 9:1 to 1:3) to give compound **49** (6.05 g, quant.).

Compound 49

^{1}H NMR (400 MHz, CD$_3$OD, 60 °C): δ 8.01 (dd, J = 3.6, 1.9 Hz, 1H), 7.79 (d, J = 3.4 Hz, 1H), 7.66 (ddd, J = 8.3, 3.5, 2.0 Hz, 1H), 7.24 (d, J = 2.4 Hz, 2H), 7.04 (d, J = 3.8 Hz, 1H), 4.08 – 3.95 (m, 2H), 3.81 (s, 3H), 3.31 (s, 3H), 3.19 – 3.09 (m, 1H), 2.98 (ddd, J = 13.1, 8.3, 3.8 Hz, 1H), 1.74 – 1.63 (m, 1H), 1.60 – 1.49 (m, 2H), 1.45 (s, 9H), 0.99 – 0.90 (m, 6H).

^{13}C NMR (101 MHz, CD$_3$OD, 60 °C): δ 174.71, 174.08, 172.12, 156.36, 155.48, 149.96, 139.48, 134.10, 133.90, 130.86, 129.73, 126.81, 125.77, 111.78, 111.24, 79.48, 54.99, 54.55, 53.96, 53.63, 41.17, 40.70, 38.80, 38.71, 38.49, 36.51, 27.41, 27.36, 24.65, 24.52, 22.06, 22.00, 21.89, 20.68, 20.62.

HRMS (ESI-TOF): calculated for $C_{27}H_{36}BrN_3NaO_7^+$ [M+Na]$^+$: 616.1634, found: 616.1631.

TLC: R$_f$ = 0.6 (2:1 Acetone : Hexane, $Ce_2(SO_4)_3$ in phosphomolybdic acid).

[α]^{21}D: −91.3 (c = 1.0, $CHCl_3$)

Note: Due to rotamerism at room temperature, NMR spectra for structural assignment were recorded at 60 °C in CD$_3$OD. However, even at this elevated temperature, the rotamers remained partially unresolved.

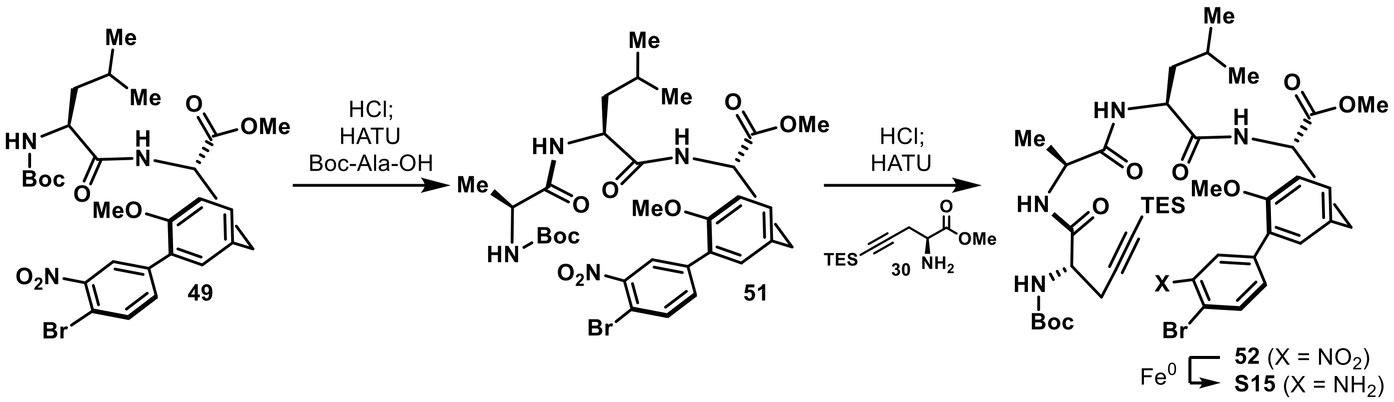

Compound **49** (6.03 g, 9.71 mmol, 1.0 eq.) was dissolved in DCM (100 mL, 0.1 M), 4.0 M HCl in 1,4-dioxane (25 mL, 0.4 M) was added. The reaction was stirred at 25 °C for 1 h. The solvent was removed under reduced pressure to give the crude. The crude was dissolved in DMF (50 mL, 0.2 M), Boc-Ala-OH (5.51 g, 29.1 mmol, 3.0 eq.), DIPEA (10.2 mL, 58.3 mmol, 6.0 eq.), and HATU (7.38 g, 19.4 mmol, 2.0 eq.) were added sequentially. The reaction was stirred at 25 °C for 8 h and quenched by saturated *aq.* NH_4Cl, extracted with EtOAc three times. The organic layers were combined and washed with saturated *aq.* $NaHCO_3$. Organic layers were concentrated under reduced pressure to give the crude **51**. The crude **51** was dissolved in DCM (100 mL, 0.1 M), 4.0 M HCl in 1,4-dioxane (25 mL, 0.4 M) was added. The reaction was stirred at 25 °C for 1 h. The solvent was removed under reduced pressure to give the crude. The crude was dissolved in DMF (50 mL, 0.2 M), and compound **30** (7.38 g, 19.4 mmol, 2.0 eq.), DIPEA (10.2 mL, 58.3 mmol, 6.0 eq.), and HATU (7.38 g, 19.4 mmol, 2.0 eq.) were added sequentially. The reaction was stirred at 25 °C for 8 h and quenched by saturated *aq.* NH_4Cl, extracted with EtOAc three times. The organic layers were combined and washed with saturated *aq.* $NaHCO_3$. Organic layers were concentrated under reduced pressure to give the crude **52**. The crude was dissolved in EtOH (100 mL, 0.1 M) and H_2O (50 mL, 0.2 M). Iron powder (5.42 g, 97.1 mmol, 10 equiv) was added, and the solution was heated at 50 °C.0 After 8 h, the mixture was filtered through a pad of Celite®, washed with DCM:MeOH 10:1, and neutralized with saturated aqueous $NaHCO_3$. The solution was extracted with DCM:MeOH 10:1 (3 x 500 mL). The extracts were dried over Na_2SO_4 and concentrated under reduced pressure. The crude was purified via flash column chromatography (DCM:MeOH 1:0 to 20:1) to afford aniline **S15** (7.99 g, 94% yield from **49**).

Compound S15

^{1}H NMR (600 MHz, CD_3OD, 60 °C): δ 7.31 (d, J = 8.2 Hz, 1H), 7.10 (d, J = 2.4 Hz, 1H), 7.07 (d, J = 2.4 Hz, 1H), 6.94 (d, J = 2.1 Hz, 1H), 6.92 (d, J = 8.4 Hz, 1H), 6.67 (dd, J = 8.2, 2.2 Hz, 1H), 4.63 (dd, J = 8.6, 5.4 Hz, 1H), 4.38 (dt, J = 9.2, 4.5 Hz, 1H), 4.26 (q, J = 7.0 Hz, 1H), 4.13 (dd, J = 8.9, 4.8 Hz, 1H), 3.72 (s, 3H), 3.67 (s, 3H), 3.11 (dd, J = 14.1, 5.4 Hz, 1H), 2.96 (dd, J = 14.1, 8.7 Hz, 1H), 2.70 (dd, J = 17.2, 4.7 Hz, 1H), 2.57 (dd, J = 17.1, 8.9 Hz, 1H), 1.58 (tt, J = 8.0, 5.5 Hz, 2H), 1.43 (s, 9H), 1.33 (d, J = 7.1 Hz, 1H), 1.25 (d, J = 7.2 Hz, 3H), 0.98 – 0.94 (m, 9H), 0.87 (d, J = 6.5 Hz, 3H), 0.84 (d, J = 6.5 Hz, 3H), 0.59 – 0.50 (m, 6H).

^{13}C NMR (151 MHz, CD_3OD, 60 °C): δ 173.22, 173.12, 172.01, 171.79, 156.54, 155.51, 144.63, 138.86, 131.37, 131.01, 129.96, 129.21, 128.89, 120.12, 116.98, 111.46, 107.26, 103.28, 79.68, 54.83, 53.96, 53.88, 51.76, 51.45, 49.28, 40.64, 36.10, 27.42, 24.46, 22.58, 22.06, 20.70, 16.86, 6.55, 4.03.

HRMS (ESI-TOF): calculated for $C_{42}H_{63}BrN_5O_8Si^+$ $[M+H]^+$: 872.3629, found: 872.3625.

TLC: R$_f$ = 0.4 (2:1 Acetone : Hexane, $Ce_2(SO_4)_3$ in phosphomolybdic acid).

$[\alpha]^{21}_D$: −143.5 (c = 1.0, $CHCl_3$)

Note: Due to rotamerism at room temperature, NMR spectra for structural assignment were recorded at 60 °C in CD_3OD. However, even at this elevated temperature, the rotamers remained partially unresolved.

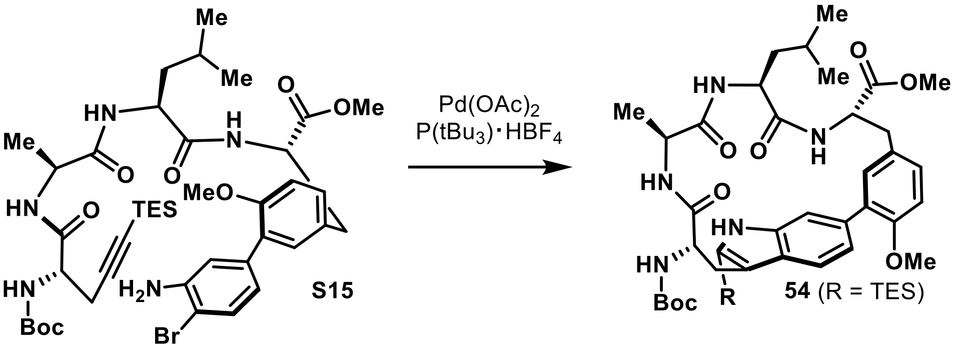

A round-bottom flask was charged with a magnetic stirring bar, compound **S15** (8.06 g, 9.20 mmol, 1.0 equiv.), $Pd(OAc)_2$ (1.04 g, 4.62 mmol, 0.5 equiv.), *t*$Bu_3P{\cdot}HBF_4$ (2.68 g, 9.20 mmol, 1.0 equiv.), and DIPEA (16 mL, 92.5 mmol, 10.0 equiv.), purged with N_2 three times. The reaction mix ture was stirred at 110 °C under nitrogen for 12 h. The reaction mixture was concentrated under reduced pressure and purified by silica gel chromatography (EtOAc:hexane 3:1 to 9:1) to give the compound **54** (5.76 g, 79% yield).

Compound 54

^{1}H NMR (400 MHz, $CDCl_3$): δ 8.03 (s, 1H), 7.71 (d, *J* = 8.3 Hz, 1H), 7.55 (s, 1H), 7.09 (d, *J* = 8.2 Hz, 1H), 7.04 – 6.85 (m, 3H), 6.36 (d, *J* = 7.9 Hz, 1H), 5.89 (d, *J* = 5.4 Hz, 1H), 5.43 (s, 1H), 5.32 (d, *J* = 7.9 Hz, 1H), 4.77 (ddd, *J* = 7.9, 6.1, 4.3 Hz, 1H), 4.39 (d, *J* = 39.5 Hz, 2H), 4.05 (d, *J* = 6.6 Hz, 1H), 3.85 (s, 3H), 3.80 (s, 3H), 3.43 (dd, *J* = 14.1, 3.7 Hz, 1H), 3.28 – 3.13 (m, 2H), 2.97 (dd, *J* = 13.0, 6.6 Hz, 1H), 1.47 (s, 9H), 1.48 – 1.39 (m, 11H), 1.33 – 1.23 (m, 1H), 0.98 (q, *J* = 9.8 Hz, 6H), 0.94 – 0.88 (m, 6H), 0.73 (d, *J* = 6.1 Hz, 3H).

^{13}C NMR (101 MHz, $CDCl_3$): δ 171.32, 170.98, 170.35, 170.21, 154.66, 137.78, 134.42, 132.65, 132.44, 130.60, 129.63, 127.41, 126.77, 120.23, 119.53, 119.13, 114.94, 111.32, 110.90, 79.27, 55.12, 52.65, 51.94, 51.35, 48.94, 39.80, 35.98, 28.41, 27.67, 23.87, 22.35, 20.88, 16.49, 6.82, 3.00.

HRMS (ESI-TOF): calculated for $C_{42}H_{61}N_5NaO_8Si^+$ $[M+Na]^+$: 814.4187, found: 814.4182.

TLC: R_f = 0.6 (2:1 Acetone : Hexane, $Ce_2(SO_4)_3$ in phosphomolybdic acid).

$[\alpha]^{21}_D$: −41.2 (*c* = 1.0, $CHCl_3$)

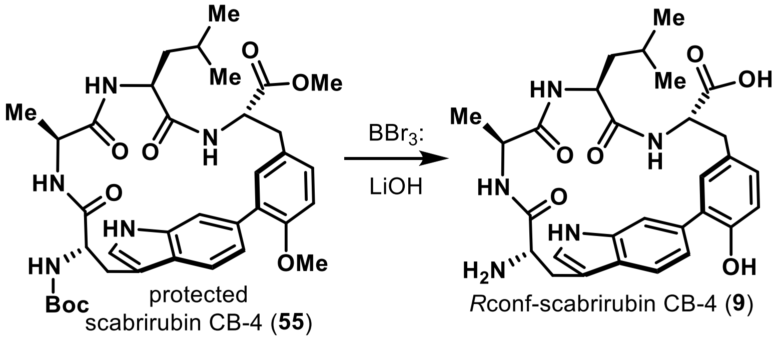

Compound **55** (100 mg, 0.123 mmol, 1.0 equiv.) was dissolved in DCM (2.5 mL, 0.05 M), 1.8 mL of BBr_3 (1M in DCM, 1.8 mmol, 15 eq.) was added dropwise at 0 °C under N_2, the reaction was stirred at 25 °C. After 2h. the reaction was quenched by dropwise addition of MeOH (10 mL) at 0 °C, then it was concentrated under reduced pressure to give the residue. The residue was dissolved in THF/H_2O/MeOH = 1:1:1 (3.0 mL, 0.03 M),

LiOH (155 mg, 3.68 mmol, 30 eq.) was added. The reaction was stirred at 25 °C for 2 h, acidified with 0.5 M HCl to adjust the pH = 3, the solvents were removed under reduced pressure, and the residue was purified by C18 silica gel (MeOH:H_2O:TFA 10:90:1 to 75:25:1) to give the title compound **9** (49.9 mg, 74% yield).

Compound 9

^{1}H NMR (600 MHz, DMSO-*d6*): δ 10.88 (d, *J* = 2.4 Hz, 1H), 9.33 (s, 1H), 8.39 (s, 2H), 8.25 (d, *J* = 8.4 Hz, 1H), 7.88 (d, *J* = 7.2 Hz, 1H), 7.66 (d, *J* = 1.4 Hz, 1H), 7.46 (d, *J* = 8.3 Hz, 1H), 7.37 (d, *J* = 2.3 Hz, 1H), 7.21 (d, *J* = 2.4 Hz, 1H), 7.14 (dd, *J* = 8.3, 1.5 Hz, 1H), 6.91 (dd, *J* = 8.2, 2.2 Hz, 1H), 6.81 (d, *J* = 8.2 Hz, 1H), 6.51 (d, *J* = 7.7 Hz, 1H), 4.36 (ddd, *J* = 11.5, 8.4, 3.3 Hz, 1H), 4.08 (ddt, *J* = 11.6, 6.8, 4.6 Hz, 2H), 3.83 (dd, *J* = 10.1, 3.7 Hz, 1H), 3.24 (dd, *J* = 13.7, 10.9 Hz, 1H), 3.13 (dd, *J* = 13.8, 3.9 Hz, 1H), 2.99 (dd, *J* = 14.7, 3.3 Hz, 1H), 2.94 – 2.84 (m, 1H), 1.35 – 1.25 (m, 1H), 1.20 – 1.14 (m, 1H), 1.05 (d, *J* = 6.7 Hz, 3H), 0.97 – 0.89 (m, 1H), 0.77 (d, *J* = 6.6 Hz, 3H), 0.74 (d, *J* = 6.5 Hz, 3H).

^{13}C NMR (151 MHz, DMSO-*d6*): δ 171.32, 170.98, 170.35, 170.21, 154.66, 137.78, 134.42, 132.65, 132.44, 130.60, 129.63, 127.41, 126.77, 120.23, 119.53, 119.13, 114.94, 111.32, 110.90, 79.27, 55.12, 52.65, 51.94, 51.35, 48.94, 39.80, 35.98, 28.41, 27.67, 23.87, 22.35, 20.88, 16.49, 6.82, 3.00.

HRMS (ESI-TOF): calculated for $C_{29}H_{36}N_5O_6^+$ $[M+H]^+$: 550.2666, found: 550.2671.

TLC: R_f = 0.6 (2:1 Acetone : Hexane, $Ce_2(SO_4)_3$ in phosphomolybdic acid).

$[\alpha]^{25}_D$: -16.2 (*c* = 0.1, MeOH)

Key NOESY Correlation of *Rconf*-scabrirubin CB-4

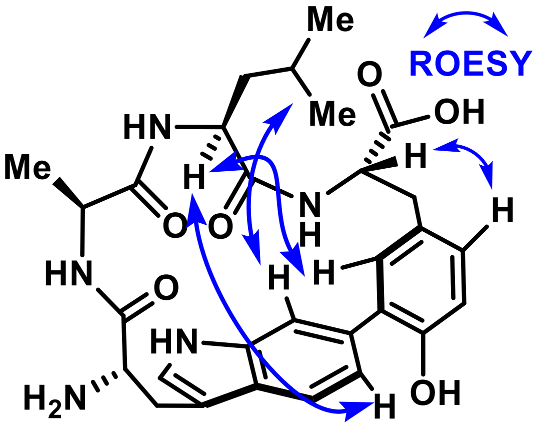

*R*conf-scabrirubin CB-4 (**9**)
(DMSO-*d6*, ROESY, 400 MHz)

LC-MS trace of synthetic scabrirubin CB-4

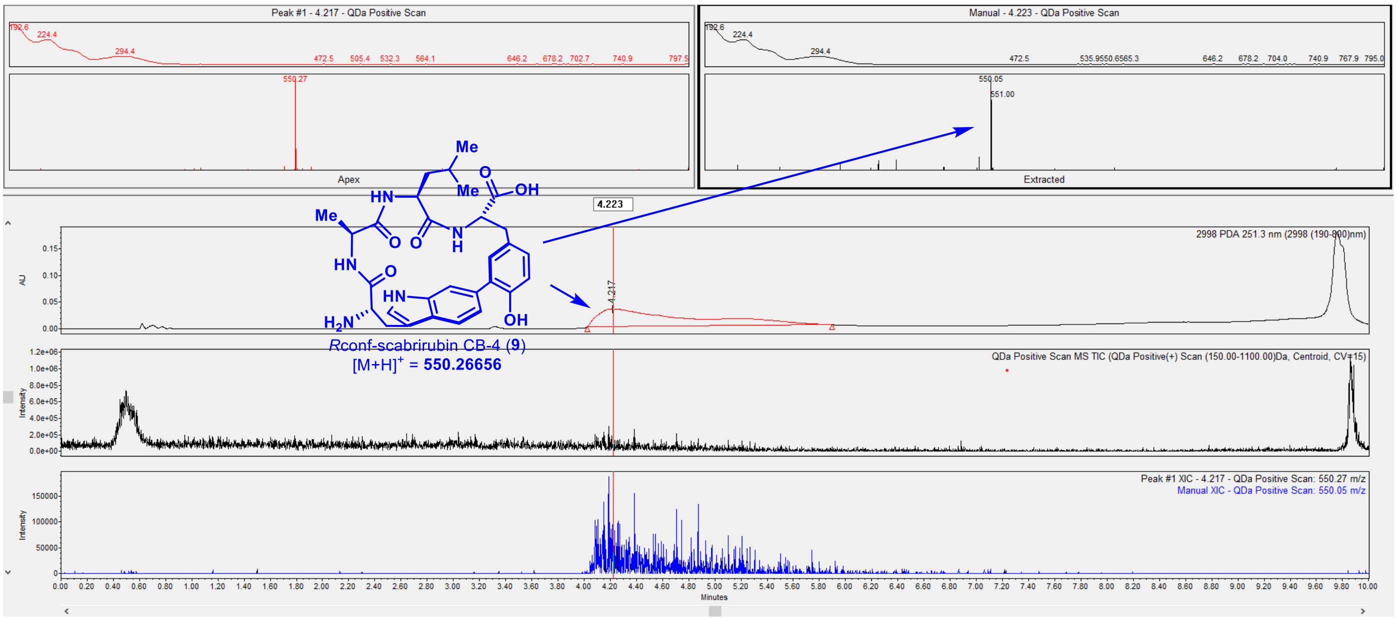

cc

Skeletal Editing of Scabrirubin CB-4

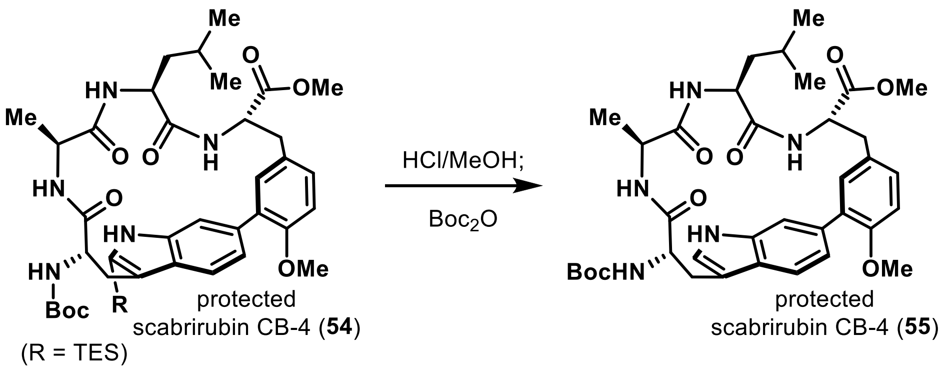

Compound **54** (1.23 g, 1.55 mmol, 1.0 equiv.) was dissolved in MeOH (14 mL, 0.1 M) and cooled to 0 °C using an ice bath. SO_2Cl_2 (2mL) was added dropwise at 0 , and the reaction was stirred at °C. After 2h. the reaction was directly concentrated under vacuum to give the residue. The residue was dissolved in DCM (15 mL, 0.1 M). Et_3N (1.1 mL, 7.75 mmol, 5.0 eq.) and Boc_2O (0.43 mL, 1.86 mmol, 1.2 eq.) were added sequentially. The reaction was stirred at 25 °C for 1h, then quenched with saturated *aq.* NH_4Cl, ext eracted with EtOAc three times. The organic layers were combined and washed with brine. Organic layers were concentrated under reduced pressure to give the crude. The crude was purified by silicagel column chromatography (EtOAc:Hexane 1:3 to 1:3) to give the title compound **55** (982 mg, 94% yield)

Compound 55

^{1}H NMR (400 MHz, $CDCl_3$): δ 8.54 (s, 1H), 7.64 (d, J = 8.3 Hz, 1H), 7.48 (s, 1H), 7.10 (dd, J = 8.2, 1.4 Hz, 1H), 7.01 (s, 1H), 6.97 – 6.84 (m, 3H), 6.34 (s, 1H), 6.13 (s, 1H), 5.99 (d, J = 5.3 Hz, 1H), 5.34 (s, 1H), 4.78 (dt, J = 7.6, 4.8 Hz, 1H), 4.39 (s, 1H), 4.08 (dd, J = 13.9, 7.4 Hz, 2H), 3.82 (s, 3H), 3.81 (s, 3H), 3.41 (dd, J = 14.4, 3.7 Hz, 1H), 3.26 (dd, J = 14.1, 4.7 Hz, 1H), 3.08 (q, J = 13.4 Hz, 2H), 1.51 – 1.45 (m, 2H), 1.45 (s, 9H), 1.35 (s, 1H), 1.29 (d, J = 6.4 Hz, 3H), 0.80 (d, J = 5.8 Hz, 3H), 0.75 (d, J = 5.7 Hz, 3H).

^{13}C NMR (101 MHz, CDCl$_3$): δ 170.96, 170.72, 170.54, 170.02, 154.73, 154.43, 135.73, 134.36, 132.20, 131.07, 127.40, 126.76, 125.61, 123.22, 120.67, 119.18, 111.84, 111.00, 109.34, 79.40, 55.24, 52.75, 51.97, 51.36, 48.48, 40.57, 35.45, 29.08, 27.71, 23.93, 22.10, 21.31, 16.89.
HRMS (ESI-TOF): calculated for $C_{36}H_{47}N_5NaO_8^+$ $[M+Na]^+$: 700.3322, found: 700.3319.
TLC: R_f = 0.4 (2:1 EtOAc : Hexane, $Ce_2(SO_4)_3$ in phosphomolybdic acid).
$[\alpha]^{21}_D$: -36.9 (c = 0.1, MeOH)

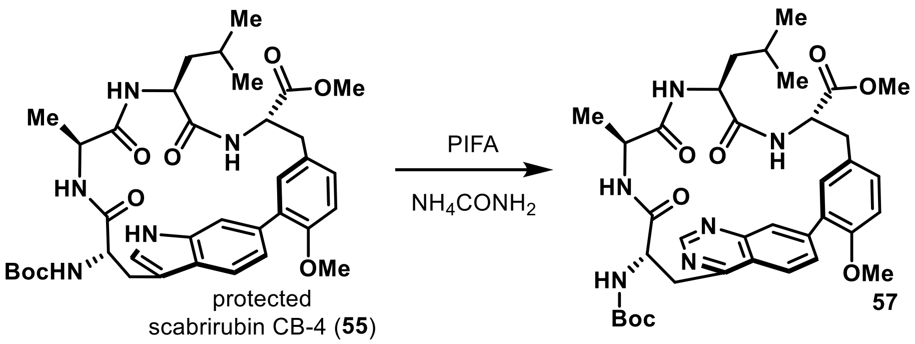

In a culture tube equipped with a stirring bar, the compound **55** (60 mg, 88.6 μmol, 1.0 eq.) and ammonium carbamate (69.2 mg, 886 μmol, 10.0 equiv) were dissolved in MeOH (1.7 mL, 0.05 M). The resulting solution was cooled to 0 °C, and then PIFA (152 mg, 354 μmol, 4.0 equiv) was added in one portion. The reaction was stirred at 0 °C for 1h and quenched with saturated aq. $Na_2S_2O_3$. The mixture was extracted with EtOAc (5mL) three times. The organic layers were combined and washed with brine and concentrated under reduced pressure to give the crude. The crude was purified by preparative thin-layer chromatography (EtOAc:Hexane 9:1) to give the title compound **57** (31.2 mg, 51% yield)

Compound 57
^{1}H NMR (400 MHz, CDCl$_3$): δ 9.23 (s, 1H), 8.15 (s, 1H), 8.13 (s, 1H), 7.72 (d, J = 8.8 Hz, 1H), 7.09 – 7.04 (m, 1H), 7.05 (d, J = 7.3 Hz, 1H), 6.95 (d, J = 8.5 Hz, 1H), 6.36 (s, 1H), 6.13 (s, 1H), 5.97 (s, 1H), 5.75 (s, 1H), 4.80 (t, J = 7.7 Hz, 1H), 4.58 (s, 1H), 4.13 – 4.03 (m, 1H), 3.88 (s, 3H), 3.81 (s, 3H), 3.69 – 3.57 (m, 1H), 3.22 (dd, J = 14.2, 4.4 Hz, 1H), 3.10 (dd, J = 14.1, 5.4 Hz, 1H), 2.29 – 1.82 (m, 2H), 1.48 – 1.45 (m, 1H), 1.44 (s, 9H), 1.12 (d, J = 7.1 Hz, 3H), 0.85 (d, J = 5.8 Hz, 3H), 0.81 (d, J = 5.8 Hz, 3H).
^{13}C NMR (101 MHz, CDCl$_3$): δ 171.58, 171.25, 170.27, 170.23, 155.43, 154.36, 154.27, 150.26, 145.17, 145.15, 134.25, 130.46, 129.99, 129.03, 128.85, 127.91, 125.25, 123.27, 111.34, 80.42, 55.65, 53.16, 52.63, 52.21, 49.34, 41.30, 36.11, 29.71, 28.30, 24.65, 22.74, 21.93, 17.36.
HRMS (ESI-TOF): calculated for $C_{36}H_{46}N_6NaO_8^+$ $[M+Na]^+$: 713.3275, found: 713.3280.
TLC: R_f = 0.6 (1:0 EtOAc : Hexane, $Ce_2(SO_4)_3$ in phosphomolybdic acid).
$[\alpha]^{21}_D$: −40.0 (c = 0.1, CDCl$_3$)

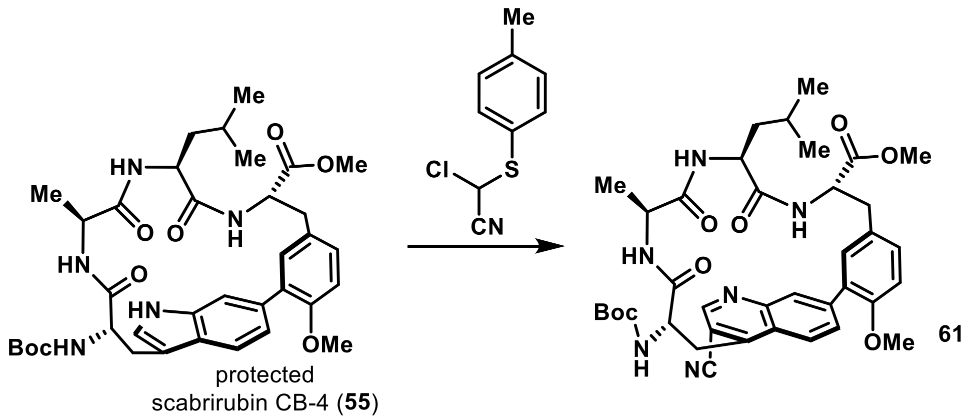

To a solution of compound **55** (30 mg, 44.3 μmol, 1.0 eq.) and Cs_2CO_3 (50.5 mg, 155 μmol, 3.5 eq.) in THF (1 mL, 0.05 M), 2-chloro-2-(p-tolylthio)acetonitrile (13.1 mg, 66.5 μmol, 1.5 eq.) was added portion-wise (0.5 equiv. every 1 h) at room temperature, and the resulting mixture was stirred for next 4 h. The reaction was quenched with saturated aq. $Na_2S_2O_3$, extracted with EtOAc (5mL) three times. The organic layers were combined and washed with brine and concentrated under reduced pressure to give the crude. The crude was purified by preparative thin-layer chromatography (EtOAc:Hexane 3:1) to give the title compound **61** (16.1 mg, 51% yield)

Compound 61

^{1}H NMR (400 MHz, CDCl$_3$): δ 8.91 (s, 1H), 8.27 (s, 1H), 8.22 (d, J = 8.6 Hz, 1H), 7.80 (d, J = 8.5 Hz, 1H), 7.32 (s, 1H), 7.03 (d, J = 8.2 Hz, 1H), 6.95 (d, J = 8.3 Hz, 1H), 6.13 (d, J = 19.2 Hz, 2H), 5.66 (s, 1H), 5.56 (s, 1H), 4.84 (s, 1H), 4.54 (q, J = 9.6 Hz, 1H), 3.98 (m, 2H), 3.89 (s, 3H), 3.82 (s, 3H), 3.23 (dd, J = 14.6, 5.5 Hz, 1H), 3.11 (dd, J = 14.6, 4.7 Hz, 1H), 1.48 (s, 11H), 1.26 (s, 1H), 1.13 (d, J = 6.7 Hz, 3H), 0.92 (d, J = 5.8 Hz, 3H), 0.89 (d, J = 5.2 Hz, 3H).

^{13}C NMR (101 MHz, CDCl$_3$): δ 171.00, 170.19, 169.53, 168.24, 154.79, 154.59, 149.49, 147.93, 142.67, 134.16, 132.26, 130.74, 130.26, 129.09, 128.51, 126.93, 124.01, 123.95, 116.10, 110.49, 106.34, 86.52, 55.00, 52.57, 52.27, 52.00, 48.05, 40.36, 35.39, 34.32, 27.69, 24.05, 22.14, 21.25, 17.04.

HRMS (ESI-TOF): calculated for $C_{38}H_{46}N_6NaO_8^+$ $[M+Na]^+$: 737.3275, found: 737.3272.

TLC: R_f = 0.7 (3:1 EtOAc : Hexane, $Ce_2(SO_4)_3$ in phosphomolybdic acid).

$[\alpha]^{21}_D$: −97.4 (c = 0.5, CDCl$_3$)

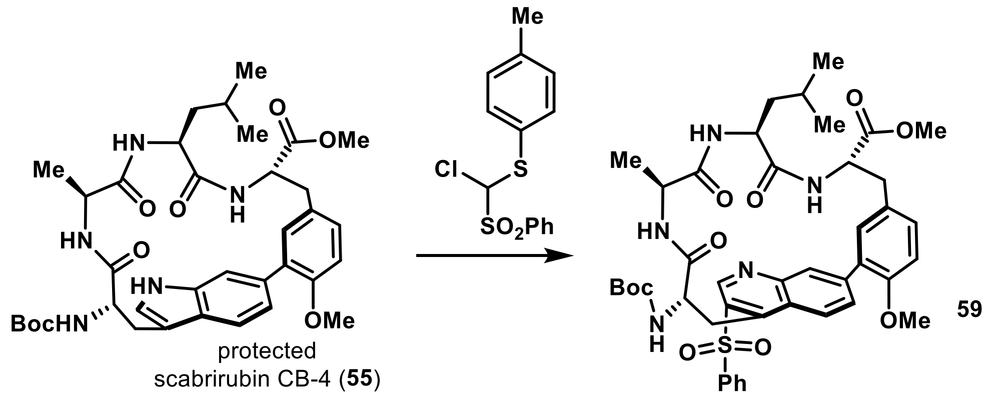

To a solution of compound **55** (30 mg, 44.3 μmol, 1.0 eq.) and Cs_2CO_3 (50.5 mg, 155 μmol, 3.5 eq.) in THF (1 mL, 0.05 M), (chloro(phenylsulfonyl)methyl)(p-tolyl)sulfane (27.6 mg, 88.6 μmol, 2.0 eq.) was added portion-wise (0.5 equiv. every 1 h) at room temperature, and the resulting mixture was stirred for next 4 h.

The reaction was quenched with saturated aq. $Na_2S_2O_3$, extracted with EtOAc (5mL) three times. The organic layers were combined and washed with brine and concentrated under reduced pressure to give the crude. The crude was purified by preparative thin-layer chromatography (EtOAc:Hexane 3:1) to give the title compound **59** (7.7 mg, 21% yield)

Compound 59

^{1}H NMR (400 MHz, CD_3OD, 60 °C): δ 10.35 (s, 1H), 8.46 (d, *J* = 15.4 Hz, 1H), 8.07 (d, *J* = 7.8 Hz, 1H), 7.69 (d, *J* = 8.3 Hz, 1H), 7.48 – 7.42 (m, 2H), 7.10 (ddd, *J* = 12.2, 8.1, 3.8 Hz, 5H), 6.99 (d, *J* = 8.2 Hz, 1H), 4.61 – 4.51 (m, 1H), 4.30 (q, *J* = 6.5 Hz, 1H), 4.20 (q, *J* = 5.9 Hz, 1H), 3.97 – 3.88 (m, 1H), 3.85 – 3.80 (m, 3H), 3.77 (s, 6H), 3.23 – 3.13 (m, 2H), 3.14 – 3.01 (m, 2H), 1.58 (d, *J* = 5.5 Hz, 2H), 1.48 – 1.44 (m, 10H), 1.02 – 0.98 (m, 3H), 0.88 (d, *J* = 6.3 Hz, 3H), 0.82 (d, *J* = 6.1 Hz, 3H).
^{13}C NMR (101 MHz, CD_3OD, 60 °C): δ 172.29, 171.41, 171.22, 170.95, 154.63, 147.80, 147.29, 142.68, 136.25, 133.07, 132.64, 132.28, 131.59, 129.53, 128.82, 128.40, 127.92, 127.22, 126.78, 125.69, 125.00, 123.80, 120.02, 118.09, 111.56, 110.57, 110.40, 108.71, 69.34, 54.16, 53.04, 50.84, 50.80, 50.67, 39.77, 39.67, 34.44, 28.72, 26.93, 26.76, 26.64, 23.63, 23.49, 21.45, 21.28, 20.18, 20.01, 15.74.
HRMS (ESI-TOF): calculated for $C_{43}H_{51}N_5NaO_{10}S^+$ $[M+Na]^+$: 852.3254, found: 852.3251.
TLC: R_f = 0.4 (3:1 EtOAc : Hexane, $Ce_2(SO_4)_3$ in phosphomolybdic acid).
$[\alpha]^{21}_{D}$: −173.0 (*c* = 0.5, $CDCl_3$)

Note: Due to rotamerism at room temperature, NMR spectra for structural assignment were recorded at 60 °C in CD_3OD. However, even at this elevated temperature, the rotamers remained partially unresolved. Singnals of the major isomer were reported.

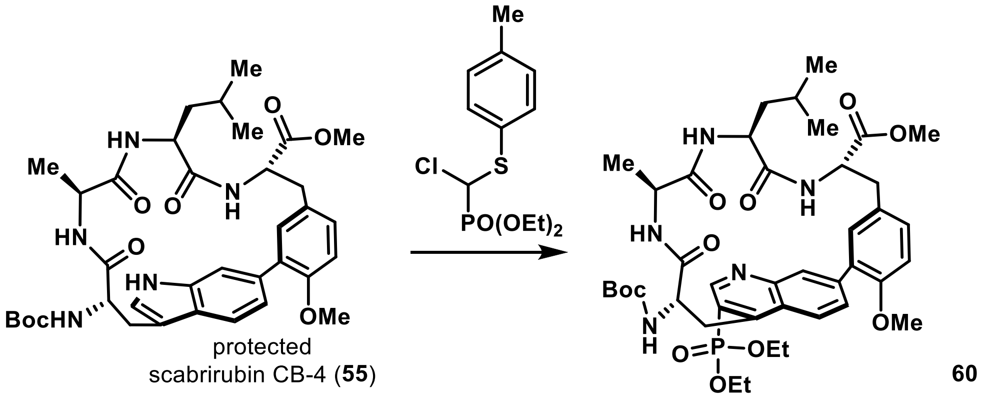

To a solution of compound **55** (30 mg, 44.3 μmol, 1.0 eq.) and Cs_2CO_3 (50.5 mg, 155 μmol, 3.5 eq.) in THF (1 mL, 0.05 M), diethyl (chloro(p-tolylthio)methyl)phosphonate (27.6 mg, 88.6 μmol, 2.0 eq.) was added portion-wise (0.5 equiv. every 1 h) at room temperature, and the resulting mixture was stirred for next 4 h. The reaction was quenched with saturated aq. $Na_2S_2O_3$, extracted with EtOAc (5mL) three times. The organic layers were combined and washed with brine and concentrated under reduced pressure to give the crude. The crude was purified by preparative thin-layer chromatography (EtOAc:Hexane 10:1) to give the title compound **60** (12.8 mg, 35% yield)

Compound 60

^{1}H NMR (400 MHz, $CDCl_3$): δ 9.16 (d, *J* = 5.3 Hz, 1H), 8.39 (d, *J* = 1.8 Hz, 1H), 8.28 (d, *J* = 8.8 Hz, 1H), 7.75 (dd, *J* = 8.7, 1.7 Hz, 1H), 7.04 (dd, *J* = 8.2, 2.0 Hz, 1H), 6.95 (d, *J* = 8.4 Hz, 2H), 6.39 (s, 1H), 6.28 (s,

1H), 5.89 (s, 1H), 5.83 (s, 1H), 4.89 (dt, J = 8.5, 5.0 Hz, 1H), 4.39 – 4.04 (m, 9H), 3.89 (s, 3H), 3.81 (s, 3H), 3.24 (dd, J = 14.3, 4.4 Hz, 1H), 3.10 (dd, J = 14.3, 5.1 Hz, 1H), 1.50 – 1.28 (m, 18H), 1.17 (d, J = 5.9 Hz, 3H), 0.90 (d, J = 6.2 Hz, 3H), 0.88 (d, J = 6.4 Hz, 3H).
^{13}C NMR (101 MHz, CDCl$_3$): δ 170.91, 170.48, 170.47, 170.25, 155.10, 155.00, 151.38, 151.25, 148.52, 141.26, 133.68, 130.46, 129.77, 129.37, 129.06, 128.33, 127.29, 125.03, 110.72, 99.36, 76.60, 62.26, 62.20, 62.13, 62.08, 56.48, 54.96, 52.42, 52.17, 51.94, 48.63, 40.36, 35.79, 31.30, 29.07, 29.03, 28.74, 27.62, 25.60, 24.06, 22.15, 22.07, 21.39, 16.42, 15.81, 15.75, 15.69, 13.50.
HRMS (ESI-TOF): calculated for $C_{41}H_{56}N_5NaO_{11}P^+$ [M+Na]$^+$: 848.3612, found: 848.3615.
TLC: R$_f$ = 0.4 (10:1 EtOAc : Hexane, $Ce_2(SO_4)_3$ in phosphomolybdic acid).
[α]^{21}$_D$: −40.0 (c = 0.1, CDCl$_3$)

Substrate Scope of CH arylation

Biaryl iodides correspond to compound **63**, **64**, **66**, and **67** were prepared according to the procedure described previously.[2]

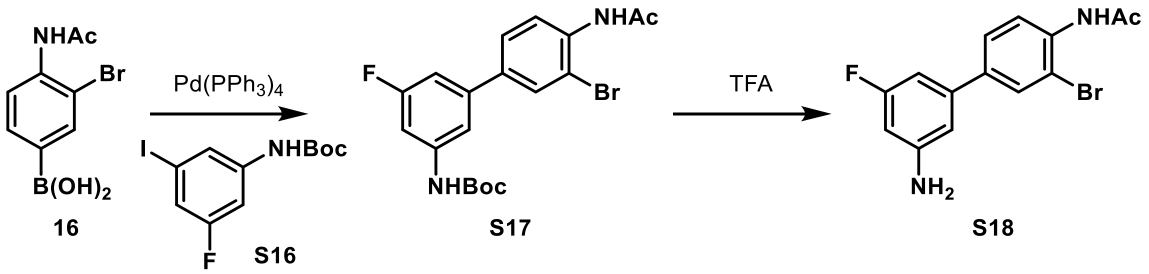

Boronic acid **16** was prepared according to the procedure previously reported by our group.[2] To a solution of compound **16** (8.32 g, 32 mmol, 1.2 eq.) in toluene/H_2O/EtOH (4:2:1, 140 mL) was added compound **S16** (9.0 g, 26.7 mmol, 1.0 eq.), K_2CO_3 (18.5 g, 134 mmol, 5.0 eq.) and Pd(PPh$_3$)$_4$ (1.54 g, 1.33 mmol, 0.05 eq.). The reaction was stirred at 80 °C under nitrogen atmosphere for 6 h, checked by LC-MS, then it was cooled to 25 °C and diluted with water, extracted with EtOAc for three times and the organic layers were combined, washed with saturated *aq.* NaCl, over Na_2SO_4, and removed under reduced pressure to give the residue, the residue was purified by silica gel chromatography to give compound **S17** (11.3 g, 42% yield). To a solution of compound **S17** (4.74 g, 11.2 mmol, 1.0 eq.) in DCM (50 mL) was added TFA (11.2 mL), the reaction was stirred at 25 °C for 3 h, the solvent and excess TFA were removed under reduced pressure, quenched by saturated *aq.* $NaHCO_3$, extracted with DCM/MeOH (10:1) for three times, over Na_2SO_4, and removed under reduced pressure to give the compound **S18** in quantitative yield.

Compound S9
Physical State: yellow oil
^{1}H NMR (600 MHz, CDCl$_3$): δ 8.29 (d, J = 8.2 Hz, 1H), 7.65 (m, 2H), 7.39 (m, 1H), 6.55 (m, 2H), 6.32 (dd, J = 10.4, 2.1 Hz, 1H), 3.92 (br, 2H), 2.22 (s, 3H).
^{13}C NMR (151 MHz, CDCl$_3$): δ 168.51, 164.24 (d, J = 243.3 Hz), 148.69 (d, J = 11.5 Hz), 141.84 (d, J = 9.9 Hz), 137.49, 135.22, 130.51, 126.85, 122.13, 113.75, 109.09, 103.75 (d, J = 22.8 Hz), 101.22 (d, J = 24.8 Hz), 24.90.
^{19}F NMR (565 MHz, CDCl$_3$): δ -112.56.
HRMS (ESI-TOF): calculated for $C_{14}H_{13}BrFN_2O^+$ [M+H]$^+$: 323.0190, found: 323.0196.
TLC: R$_f$ = 0.7 (1:1 EtOAc : Hexane, $Ce_2(SO_4)_3$ in phosphomolybdic acid).

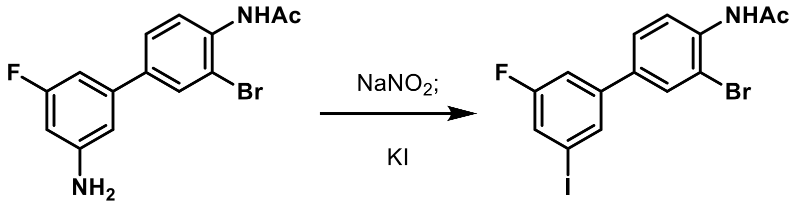

S18 S19

To a solution of the compound **S18** (3.6 g, 11.2 mmol, 1.0 eq.) in MeCN (100 mL) at 0 °C was added *p*TsOH·H_2O (8.51 g, 44.7 mmol, 4.0 eq.), $NaNO_2$ (1.0 g, 14.5 mmol, 1.3 eq.) in H_2O (20 mL) sequentially. The reaction was stirred at 25 °C for 3 h, then the KI (12.1 g, 72.7 mmol, 6.5 eq.) in H_2O (20 mL) was added dropwise at 0 °C, the reaction was stirred at 25 °C for 12 h, checked by LC-MS, quenched by saturated *aq.* $NaHCO_3$ and saturated *aq.* $Na_2S_2O_3$, extracted with EtOAc for three times, the organic layers were combined, washed with saturated *aq.* NaCl and removed under reduced pressure to give the residue, the residue was purified by silica gel chromatography to give compound **S19** (2.7 g, 56%).

Compound S19
Physical State: white solid
^{1}H NMR (600 MHz, $CDCl_3$): δ 8.43 (d, *J* = 8.4 Hz, 1H), 7.69 (d, *J* = 2.1 Hz, 1H), 7.65 (t, *J* = 1.4 Hz, 2H), 7.46 (dd, *J* = 8.6, 2.1 Hz, 1H), 7.40 (ddd, *J* = 7.7, 2.2, 1.5 Hz, 1H), 7.18 (m, 1H), 2.26 (s, 3H).
^{13}C NMR (151 MHz, $CDCl_3$): δ168.37, 162.65 (d, *J* = 252.0 Hz), 142.91 (d, *J* = 7.9 Hz), 135.94, 135.27, 131.81 (d, *J* = 2.6 Hz), 130.58, 127.08, 123.88 (d, *J* = 23.7 Hz), 121.98, 113.68, 113.54, 94.11 (d, *J* = 8.6 Hz), 25.02.
^{19}F NMR (565 MHz, $CDCl_3$): δ -110.28.
HRMS (ESI-TOF): calculated for $C_{14}H_{11}BrFINO^+$ $[M+H]^+$: 433.9047, found: 433.9056.
TLC: R_f = 0.3 (1:5 EtOAc : Hexane, $Ce_2(SO_4)_3$ in phosphomolybdic acid.

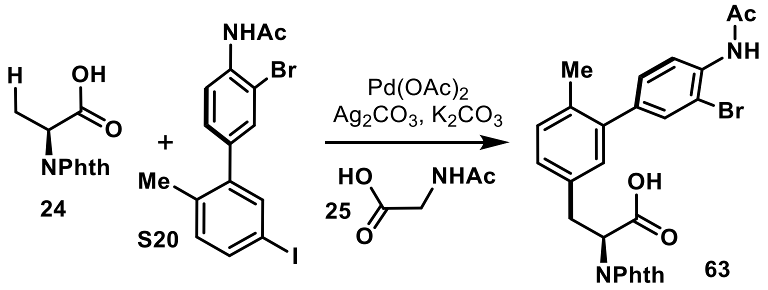

On 0.2 mmol scale, **General Procedure A** was followed with aryl iodide **S20**. Purification by preparative thin-layer chromatography (MeOH:EtOAc 1:20) gave the title compound **63**. (41.7 mg, 40% yield)

Compound 63
Physical State: yellow oil
^{1}H NMR (600 MHz, CDCl$_3$): δ 8.17 (d, *J* = 8.3 Hz, 1H), 7.69 (m, 2H), 7.61 (d, *J* = 5.3 Hz, 3H), 7.13 (s, 1H), 6.99 (t, *J* = 7.3 Hz, 2H), 6.89 (d, *J* = 8.1 Hz, 1H), 6.78 (s, 1H), 4.98 (s, 1H), 3.59 (m, 3H), 2.24 (s, 3H), 2.07 (s, 3H).
^{13}C NMR (151 MHz, CDCl$_3$): 168.52, 167.82, 139.80, 138.94, 134.21, 133.73, 132.41, 131.63, 130.76, 130.19, 129.09, 128.28, 123.54, 121.37, 112.85, 29.79, 24.94, 20.01.
HRMS (ESI-TOF): calculated for $C_{26}H_{21}BrN_2NaO_5^+$ [M+Na]$^+$: 543.0526, found: 543.0533.
TLC: R$_f$ = 0.2 (10:1 EtOAc : MeOH, $Ce_2(SO_4)_3$ in phosphomolybdic acid).
[α]^{25}$_D$: −99.8 (*c* = 0.1, CHCl$_3$)

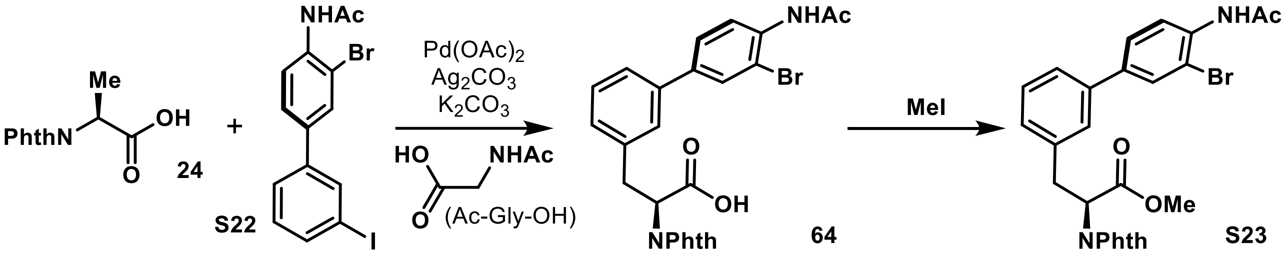

On 0.2 mmol scale, **General Procedure B** was followed with aryl iodide **S22**. Purification by preparative thin-layer chromatography (EtOAc:Hexane 1:2) gave the title compound **23** (62.2 mg, 82% yield).

Compound S23
Physical State: white solid
^{1}H NMR (400 MHz, CDCl$_3$): δ 8.30 (d, *J* = 8.6 Hz, 1H), 7.79 (dd, *J* = 5.5, 3.1 Hz, 2H), 7.69 (dd, *J* = 5.5, 3.1 Hz, 2H), 7.62 (s, 1H), 7.54 (d, *J* = 2.1 Hz, 1H), 7.35 – 7.22 (m, 4H), 7.17 (dt, *J* = 7.3, 1.7 Hz, 1H), 5.20 (dd, *J* = 11.1, 5.4 Hz, 1H), 3.80 (s, 3H), 3.69 – 3.54 (m, 2H), 2.25 (s, 3H).
^{13}C NMR (101 MHz, CDCl$_3$): δ 169.27, 167.45, 139.37, 137.45, 134.26, 131.54, 130.43, 129.19, 128.21, 127.36, 126.91, 125.50, 123.56, 121.88, 53.17, 52.99, 34.65.
HRMS (ESI-TOF): calculated for $C_{26}H_{21}BrN_2NaO_5^+$ [M+Na]$^+$: 543.0532, found: 543.0528.
TLC: R$_f$ = 0.5 (1:1 EtOAc:Hexane, $Ce_2(SO_4)_3$ in phosphomolybdic acid).
[α]^{21}$_D$: −106.7 (*c* = 1.0, CHCl$_3$)

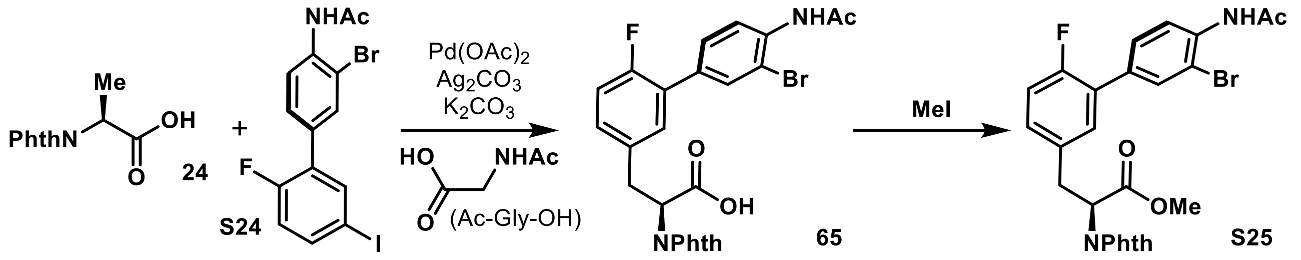

On 0.2 mmol scale, **General Procedure B** was followed with aryl iodide **S24**. Purification by preparative thin-layer chromatography (EtOAc:Hexane 1:2) gave the title compound **S25** (56.0 mg, 76% yield).

Compound S25

Physical State: white solid

^{1}H NMR (400 MHz, CDCl$_3$): δ 8.32 (d, J = 8.5 Hz, 1H), 7.81 (dd, J = 5.5, 3.1 Hz, 2H), 7.72 (dd, J = 5.5, 3.0 Hz, 2H), 7.64 (s, 1H), 7.55 – 7.40 (m, 1H), 7.25 (d, J = 9.2 Hz, 1H), 7.21 – 7.06 (m, 2H), 6.97 (dd, J = 10.4, 8.1 Hz, 1H), 5.15 (dd, J = 11.0, 5.5 Hz, 1H), 3.79 (s, 3H), 3.67 – 3.49 (m, 2H), 2.25 (s, 3H).

^{13}C NMR (101 MHz, CDCl$_3$): δ 168.48, 166.80, 159.18, 156.71, 134.46, 133.74, 132.33, 131.68 (d, J = 6.7 Hz), 131.62, 130.82, 130.14 (d, J = 3.5 Hz), 129.17 (d, J = 8.5 Hz), 128.21 (d, J = 3.0 Hz), 123.00, 120.78, 115.81 (d, J = 22.9 Hz), 52.46, 52.39, 33.28, 24.31.

^{19}F NMR (376 MHz, CDCl$_3$): δ -120.45.

HRMS (ESI-TOF): calculated for $C_{26}H_{20}BrFN_2NaO_5^+$ [M+Na]$^+$: 561.0437, found: 561.0436.

TLC: R$_f$ = 0.5 (1:1 EtOAc:Hexane, $Ce_2(SO_4)_3$ in phosphomolybdic acid).

[α]^{21}$_D$: −78.4 (c = 1.0, CHCl$_3$)

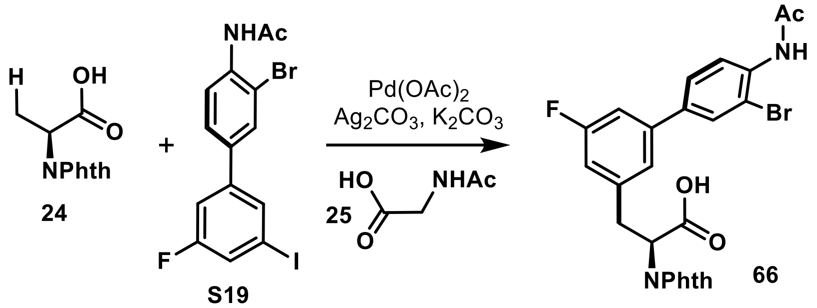

On 0.2 mmol scale, **General Procedure A** was followed with aryl iodide **S19**. Purification by preparative thin-layer chromatography (MeOH:EtOAc 1:20) gave the title compound **66** (68.1 mg, 65% yield).

Compound 66

Physical State: yellow oil

^{1}H NMR (600 MHz, CDCl$_3$): δ 8.22 (s, 1H), 7.71 (m, 2H), 7.61 (m, 3H), 7.45 (s, 1H), 7.21 (d, J = 7.4 Hz, 1H), 7.00 (s, 1H), 6.92 (d, J = 9.4 Hz, 1H), 6.80 (d, J = 8.4 Hz, 1H), 5.12 (m, 2H), 3.48 (m, 2H), 2.23 (s, 3H).

^{13}C NMR (151 MHz, CDCl$_3$): 172.91, 168.80, 167.72, 163.10 (d, J = 246.9 Hz), 141.29, 140.19, 136.80, 135.17, 134.38, 131.50, 130.46, 126.91, 123.65, 123.05, 122.11, 115.11 (d, J = 21.5 Hz), 113.71, 112.36 (d, J = 22.6 Hz), 53.20, 34.57, 24.90.

HRMS (ESI-TOF): calculated for $C_{25}H_{18}BrFN_2NaO_5^+$ [M+Na]$^+$: 547.0275, found: 547.0282.

^{19}F NMR (565 MHz, CDCl$_3$): δ -112.332.

TLC: R$_f$ = 0.2 (10:1 EtOAc Methanol, $Ce_2(SO_4)_3$ in phosphomolybdic acid).

[α]^{25}$_D$: -43.4 (c = 0.1, CHCl$_3$)

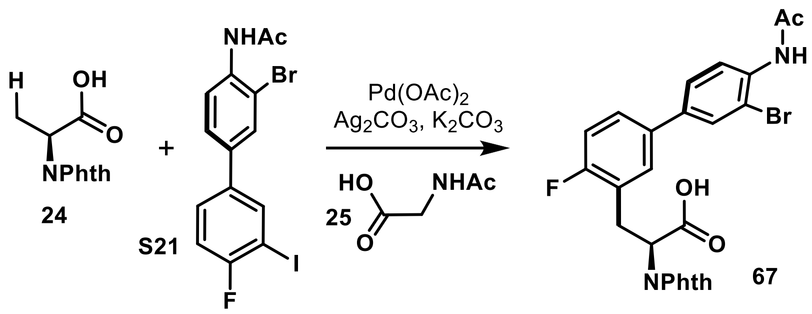

On 0.2 mmol scale, **General Procedure A** was followed with aryl iodide **S21**. Purification by preparative thin-layer chromatography (MeOH:EtOAc 1:20) gave the title compound **67** (10.0 mg, 19% yield).

Compound 67

Physical State: yellow oil

^{1}H NMR (400 MHz, $CDCl_3$): δ 8.21 (d, *J* = 8.4 Hz, 1H), 7.84 (dd, *J* = 5.3, 3.1 Hz, 1H), 7.73 (m, 3H), 7.64 (m, 2H), 7.38 (s, 1H), 7.18 (m, 2H), 6.98 (t, *J* = 9.0 Hz, 1H), 5.18 (d, *J* = 8.3 Hz, 1H), 4.78 (s, 1H), 3.56 (m, 2H), 2.25 (s, 3H).

^{13}C NMR (101 MHz, $CDCl_3$): 172.97, 168.65, 167.55, 161.25 (d, *J* = 248.1 Hz), 137.20, 135.31, 134.66, 131.94, 131.56, 130.30, 129.78, 127.42, 126.77, 124.63 (d, *J* = 16.0 Hz), 123.62, 122.09, 115.93 (d, *J* = 22.3 Hz), 113.65, 28.91, 24.89, 15.25.

HRMS (ESI-TOF): calculated for $C_{25}H_{18}BrFN_2NaO_5^+$ $[M+Na]^+$: 547.0275, found: 547.0282.

^{19}F NMR (565 MHz, $CDCl_3$) δ -119.31.

TLC: R_f = 0.2 (10:1 EtOAc : MeOH, $Ce_2(SO_4)_3$ in phosphomolybdic acid).

$[\alpha]^{25}_D$: -13.0 (*c* = 0.1, $CHCl_3$)

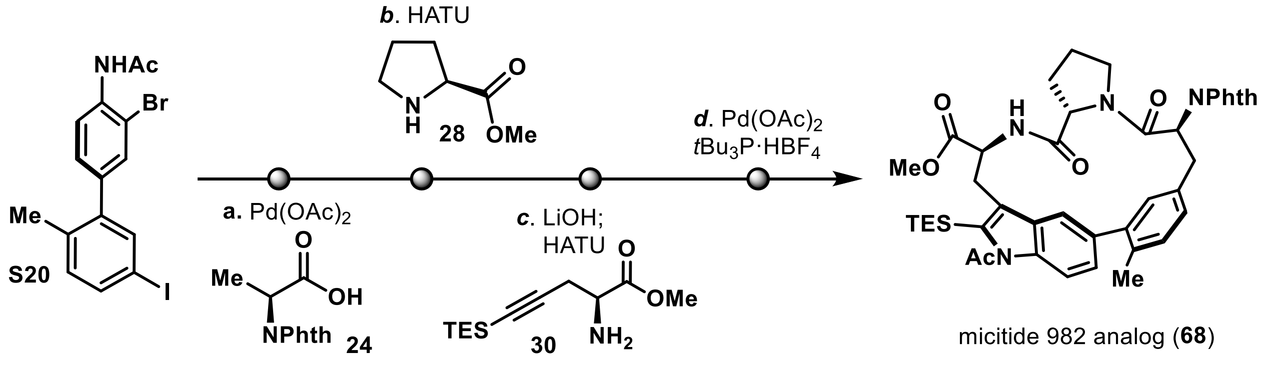

On 5.0 mmol scale, **General Procedure A** was followed with aryl iodide **S20**. After completion of the CH arylation, the crude was directly used in the subsequent amide coupling right after the removal of the solvent under vacuum. The crude was dissolved in DMF (50 mL, 0.1 M), then L-Proline methyl ester (2.48 g, 15 mmol, 3.0 eq.), HATU (3.8 g, 10.0 mmol, 2.0 eq.) and DIPEA (4.43 mL, 25.0 mmol, 5.0 eq.) was added, the reaction was stirred at for 2 h. The reaction was quenched by saturated *aq.* NH_4Cl, extracted with EtOAc for three times. The organic layers were combined, washed with 1N HCl *aq.*, and concentrated under vacuum to give the crude. The crude was dissolved in THF/MeOH/H_2O = 1:1:1 (50 mL, 0.1 M), and LiOH·H_2O (1.26 g, 30.0 mmol, 6.0 eq.) was added, the reaction was stirred at 25 °C for 1 h. The reaction was quenched by the addition of 1N HCl *aq.* and extracted with DCM/MeOH = 10:1 for three times. The organic layers were combined, dried over Na_2SO_4, and concentrated under vacuum. The crude was then dissolved in DMF (50 mL, 0.1 M), compound **30** (1.33 g, 5.50 mmol, 1.1 eq.), HATU (3.80 g, 10.0 mmol, 2.0 eq.), and DIPEA (1.8

mL, 25.0 mmol, 5.0 eq.) were added sequentially. The reaction was stirred at 25 °C for 1.5 h, quenched by saturated *aq.* NH_4Cl and extracted with EtOAc three times. The organic layers were combined, washed with 1N HCl *aq.*, dride over Na_2SO_4 and concentrated under vacuum to give the residue. The residue was filtered thorough a pad of silicagel (EtOAc:Hexane 2:1) to give the crude. The crude was dissolved in toluene (250 mL, 0.02 M), $Pd(OAc)_2$ (554 mg, 2.47 mmol, 0.5 eq.), $t$$Bu_3P{\cdot}HBF_4$ (1.43 g, 4.94 mmol, 1.0 eq.) and DIPEA (8.6 mL, 49.4 mmol, 10.0 eq.) was added. The reaction was stirred at 110 °C under nitrogen for 12 h. The solvent was removed under reduced pressure to give the residue, the residue was purified by silica gel chromatography to give the compound **68** (1.31 g, 39% yield from aryl iodide **S20**).

Compound 68
Physical State: colorless solid
^{1}H NMR (400 MHz, $CDCl_3$): δ 7.91 (s, 1H), 7.91 – 7.85 (m, 2H), 7.76 (dd, J = 5.5, 2.6 Hz, 2H), 7.70 (s, 1H), 7.60 (dd, J = 8.8, 1.9 Hz, 1H), 7.58 – 7.51 (m, 1H), 7.34 (d, J = 8.4 Hz, 1H), 7.23 (d, J = 2.2 Hz, 2H), 5.05 (t, J = 8.8 Hz, 1H), 4.98 (dd, J = 11.7, 2.1 Hz, 1H), 4.45 (dt, J = 8.6, 2.4 Hz, 1H), 4.22 (t, J = 11.5 Hz, 1H), 3.83 – 3.72 (m, 1H), 3.64 (s, 3H), 3.52 (ddd, J = 10.5, 8.2, 3.1 Hz, 1H), 3.28 (d, J = 14.7 Hz, 1H), 3.11 (td, J = 9.4, 6.8 Hz, 1H), 2.81 (s, 3H), 2.69 (dd, J = 12.5, 2.1 Hz, 1H), 2.47 (s, 3H), 2.18 – 1.89 (m, 3H), 1.66 – 1.49 (m, 1H), 0.96 – 0.76 (m, 15H).
^{13}C NMR (101 MHz, $CDCl_3$): δ 172.74, 170.98, 169.43, 167.10, 165.76, 139.78, 136.37, 136.19, 136.03, 135.09, 134.45, 134.17, 133.39, 133.00, 131.54, 131.40, 131.05, 128.08, 125.60, 123.67, 122.83, 112.57, 61.10, 54.41, 52.23, 52.03, 46.99, 36.21, 31.03, 29.05, 26.52, 25.01, 20.94, 8.20, 6.05.
HRMS (ESI-TOF): calculated for $C_{43}H_{48}N_4NaO_7Si^+$ $[M+Na]^+$: 783.3190 found: 783.3187.
TLC: R_f = 0.5 (2:1 EtOAc:Hexane, $Ce_2(SO_4)_3$ in phosphomolybdic acid).
$[\alpha]^{21}_D$: +50.9 (c = 1.0, $CHCl_3$)

Key ROESY Correlations of Compound 68

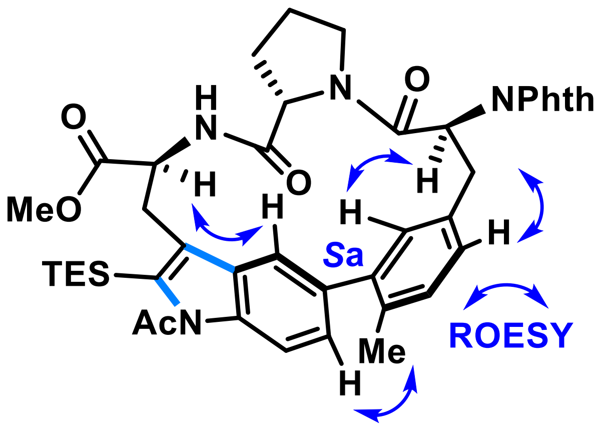

68 ($CDCl_3$, 400 MHz)

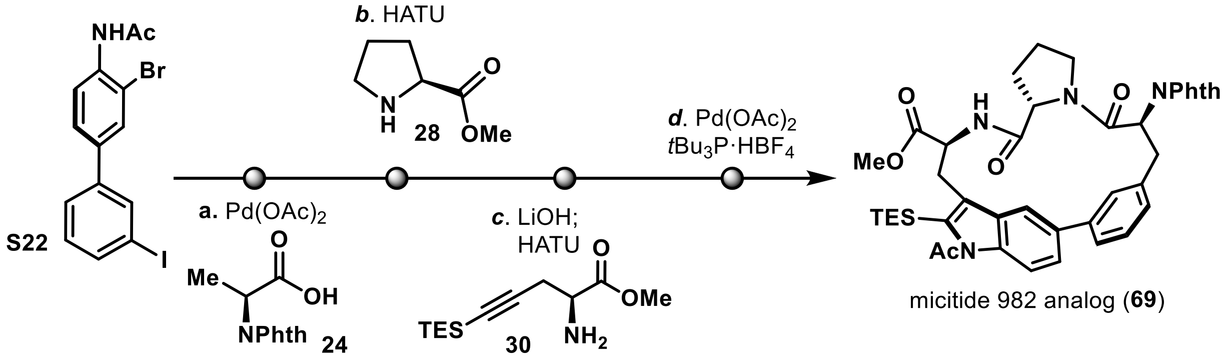

On 4.0 mmol scale, **General Procedure A** was followed with aryl iodide **S22**. After completion of the CH arylation, the crude was directly used in the subsequent amide coupling right after the removal of the solvent under vacuum. The crude was dissolved in DMF (40 mL, 0.1 M), then L-Proline methyl ester (1.98 g, 12 mmol, 3.0 eq.), HATU (3.04 g, 8.00 mmol, 2.0 eq.) and DIPEA (3.5 mL, 20 mmol, 5.0 eq.) was added, the reaction was stirred at for 2 h. The reaction was quenched by saturated *aq.* NH_4Cl, extracted with EtOAc for three times. The organic layers were combined, washed with 1N HCl *aq.*, and concentrated under vacuum to give the crude. The crude was dissolved in THF/MeOH/H_2O = 1:1:1 (40 mL, 0.1 M), and LiOH·H_2O (1.01 g, 24.0 mmol, 6.0 eq.) was added, the reaction was stirred at 25 °C for 1 h. The reaction was quenched by the addition of 1N HCl *aq.* and extracted with DCM/MeOH = 10:1 for three times. The organic layers were combined, dried over Na_2SO_4, and concentrated under vacuum. The crude was then dissolved in DMF (40 mL, 0.1 M), compound **30** (1.06 g, 4.40 mmol, 1.1 eq.), HATU (3.04 g, 8.00 mmol, 2.0 eq.), and DIPEA (3.5 mL, 20.0 mmol, 5.0 eq.) were added sequentially. The reaction was stirred at 25 °C for 1.5 h, quenched by saturated *aq.* NH_4Cl and extracted with EtOAc three times. The organic layers were combined, washed with 1N HCl *aq.*, dride over Na_2SO_4 and concentrated under vacuum to give the residue. The residue was filtered thorough a pad of silicagel (EtOAc:Hexane 2:1) to give the crude. The crude was dissolved in toluene (200 mL, 0.02 M), $Pd(OAc)_2$ (450 mg, 2.01 mmol, 0.50 eq.), *t*Bu_3P·HBF_4 (1.16 g, 4.01 mmol, 1.0 eq.) and DIPEA (7.0 mL, 40.1 mmol, 10 eq.) was added. The reaction was stirred at 110 °C under nitrogen for 12 h. The solvent was removed under reduced pressure to give the residue, the residue was purified by silica gel chromatography to give the compound **69** (810 mg, 30% yield from aryl iodide **69**).

Compound 69
Physical State: white solid
^{1}H NMR (400 MHz, CDCl3): δ 8.35 (s, 1H), 7.88 (m, 2H), 7.77 (m, 2H), 7.69 – 7.54 (m, 4H), 7.40 (brs, 1H), 7.27 (m, 1H), 5.02 (d, *J* = 11.5 Hz, 1H), 4.95 (t, *J* = 10.5 Hz, 1H), 4.57 (d, *J* = 8.1 Hz, 1H), 4.39 (t, *J* = 12.3 Hz, 1H), 3.77 (brs, 1H), 3.63 (s, 3H), 3.31 (d, *J* = 14.8 Hz, 1H), 3.07 (s, 1H), 2.78 (s, 3H), 2.66 (d, *J* = 12.6 Hz, 1H), 2.27 – 1.97 (m, 2H), 1.77 (s, 1H), 1.39 – 1.08 (m, 2H), 0.84 – 0.60 (m, 15H).
^{13}C NMR (101 MHz, CDCl3): δ 172.17, 170.50, 168.74, 166.34, 165.23, 139.06, 137.32, 135.84, 134.66, 133.83, 132.53, 130.90, 130.85, 129.94, 128.24, 127.62, 124.17, 123.06, 122.10, 118.72, 113.34, 60.50, 53.33, 51.75, 51.60, 46.36, 36.02, 29.98, 28.62, 25.91, 24.29, 7.45, 5.28.
HRMS (ESI-TOF): calculated for $C_{42}H_{46}N_4NaO_7Si^+$ $[M+Na]^+$: 769.3033, found: 769.3033.
TLC: R_f = 0.5 (2:1 EtOAc : Hexane, $Ce_2(SO_4)_3$ in phosphomolybdic acid).
$[\alpha]^{21}_D$: +77.3 (*c* = 1.0, $CHCl_3$)

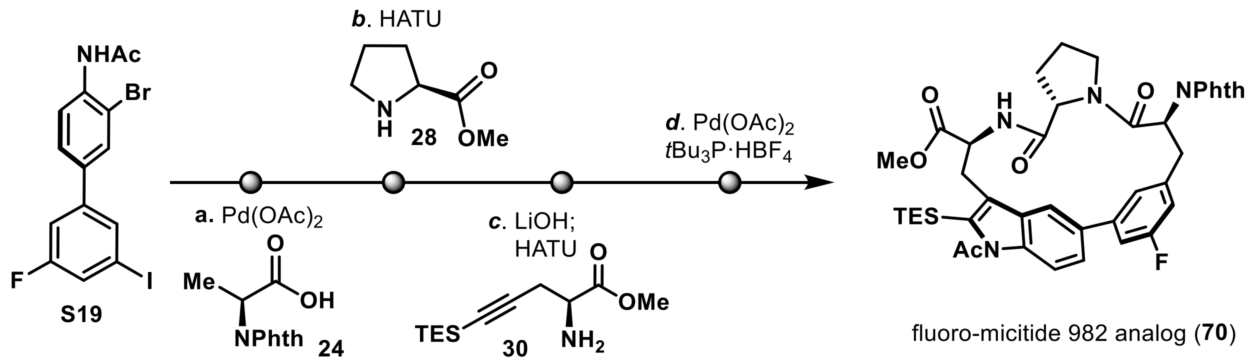

fluoro-micitide 982 analog (**70**)

On 5.0 mmol scale, **General Procedure A** was followed with aryl iodide **S19**. After completion of the CH arylation, the crude was directly used in the subsequent amide coupling right after the removal of the solvent under vacuum. The crude was dissolved in DMF (50 mL, 0.1 M), then L-Proline methyl ester (2.48 g, 15 mmol, 3.0 eq.), HATU (3.8 g, 10.0 mmol, 2.0 eq.) and DIPEA (4.43 mL, 25.0 mmol, 5.0 eq.) was added, the reaction was stirred at for 2 h. The reaction was quenched by saturated *aq.* NH_4Cl, extracted with EtOAc for three times. The organic layers were combined, washed with 1N HCl *aq.*, and concentrated under vacuum to give the crude. The crude was dissolved in THF/MeOH/H_2O = 1:1:1 (50 mL, 0.1 M), and LiOH·H_2O (1.26 g, 30.0 mmol, 6.0 eq.) was added, the reaction was stirred at 25 °C for 1 h. The reaction was quenched by the addition of 1N HCl *aq.* and extracted with DCM/MeOH = 10:1 for three times. The organic layers were combined, dried over Na_2SO_4, and concentrated under vacuum. The crude was then dissolved in DMF (50 mL, 0.1 M), compound **30** (1.33 g, 5.50 mmol, 1.1 eq.), HATU (3.80 g, 10.0 mmol, 2.0 eq.), and DIPEA (1.8 mL, 25.0 mmol, 5.0 eq.) were added sequentially. The reaction was stirred at 25 °C for 1.5 h, quenched by saturated *aq.* NH_4Cl and extracted with EtOAc three times. The organic layers were combined, washed with 1N HCl *aq.*, dride over Na_2SO_4 and concentrated under vacuum to give the residue. The residue was filtered thorough a pad of silicagel (EtOAc:Hexane 2:1) to give the crude. The crude was dissolved in toluene (170 mL, 0.03 M), $Pd(OAc)_2$ (380 mg, 1,69 mmol, 0.34 eq.), *t*Bu_3P·HBF_4 (982 mg, 3.38 mmol, 0.68 eq.) and DIPEA (5.9 mL, 33.8 mmol, 6.8 eq.) was added. The reaction was stirred at 110 ºC under nitrogen for 12 h. The solvent was removed under reduced pressure to give the residue, the residue was purified by silica gel chromatography to give the compound **70** (897 mg, 23% yield from aryl iodide **S19**).

Compound 70
Physical State: white solid
^{1}H NMR (600 MHz, $CDCl_3$): δ 8.14 (s, 1H), 7.86 (m, 4H), 7.75 (m, 2H), 7.57 (m, 2H), 7.27 (d, *J* = 10.2 Hz, 1H), 6.96 (d, *J* = 9.1 Hz, 1H), 5.02 (dd, *J* = 11.5, 1.6 Hz, 1H), 4.95 (t, *J* = 9.1 Hz, 1H), 4.57 (dd, *J* = 8.1, 2.5 Hz, 1H), 4.44 (t, *J* = 12.1 Hz, 1H), 3.76 (m, 1H), 3.64 (m, 1H), 3.61 (s, 3H), 3.29 (d, *J* = 14.5 Hz, 1H), 3.07 (m, 1H), 2.74 (s, 3H), 2.60 (m, 1H), 2.17 (m, 1H), 2.03 (m, 2H), 1.78 (m, 1H), 0.65 (m, 12H), 0.56 (m, 3H).
^{13}C NMR (151 MHz, $CDCl_3$): δ 172.82, 170.27 (d, *J* = 261.9 Hz), 166.96, 165.75, 164.19, 162.57, 141.87 (d, *J* = 8.1 Hz), 139.94 (d, *J* = 7.7 Hz), 136.83, 136.68, 134.59, 134.11, 133.22, 131.59, 126.21, 123.78, 122.71, 119.51, 114.83 (d, *J* = 21.3 Hz), 114.10, 111.72 (d, *J* = 22.0 Hz), 61.34, 53.77, 52.37, 52.28, 47.19, 36.48, 30.81, 29.51, 26.59, 24.92, 8.09, 5.85.
^{19}F NMR (376 MHz, $CDCl_3$): δ -113.96.
HRMS (ESI-TOF): calculated for $C_{42}H_{46}BrFN_4NaO_7Si^+$ $[M+Na]^+$: 867.2195, found: 867.2205.
TLC: R_f = 0.7 (7:3 EtOAc : Hexane, $Ce_2(SO_4)_3$ in phosphomolybdic acid).
$[\alpha]^{25}_D$: -71.6 (*c* = 0.2, $CHCl_3$)

Key ROESY Correlations of Compound 70

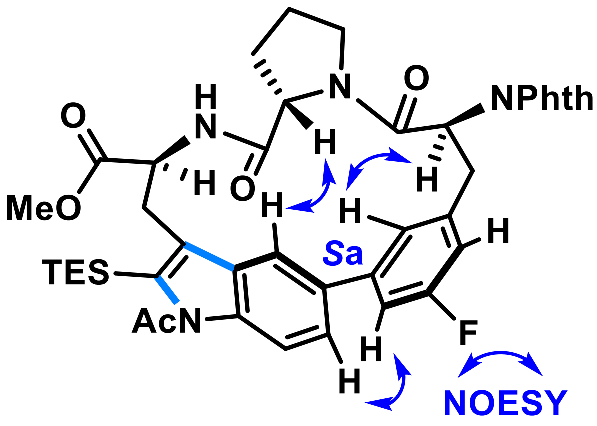

70 ($CDCl_3$, 600 MHz)

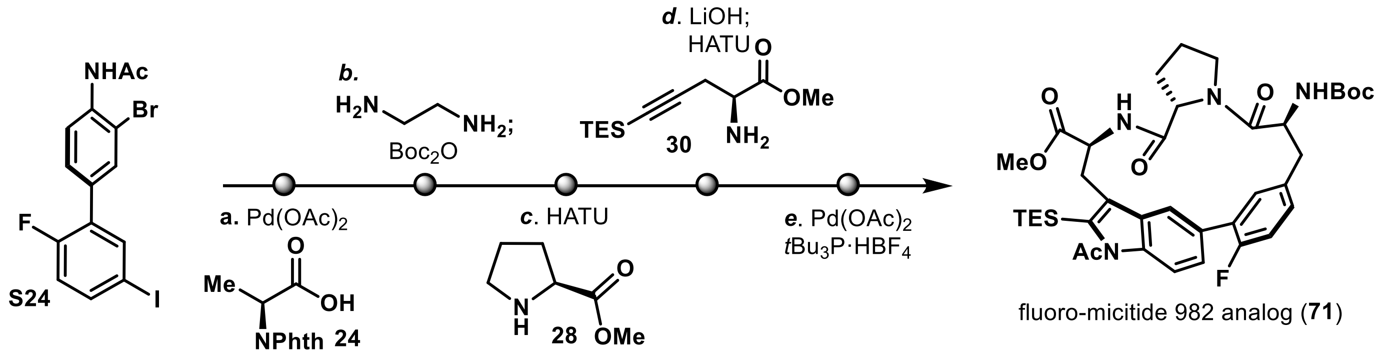

fluoro-micitide 982 analog (**71**)

On a 5.0 mmol scale, **General Procedure A** was followed with aryl iodide **S24**. After completion of the CH arylation, the crude was directly used in the subsequent reaction right after the removal of the solvent under vacuum. The crude was dissolved in *n*-BuOH (50 mL, 0.1 M), and ethylenediamine (3.3 mL, 50 mmol, 10.0 eq.) was added. The reaction was stirred at 25 °C for 15 h. The reaction mixture was directly concentrated under reduced pressure to give the crude. The crude was dissolved in THF (50 mL, 0.1 M), saturated *aq.* $NaHCO_3$ (5 mL, 1.0 M), and $(Boc)_2O$ (8.0 mL, 35.0 mmol, 7.0 eq.) were added sequentially. The reaction mixture was stirred at 25 °C for 3 h and quenched by 1N HCl *aq.*, extracted with DCM/MeOH = 10:1 three times. The organic layers were combined and washed with saturated *aq.* NaCl, and dried over Na_2SO_4. Organic layers were concentrated under reduced pressure and filtered through a pad of silicagel (EtOAc:Hexane 1:10 to 1:0) to give a crude. The crude was dissolved in DMF (50 mL, 0.1 M), then L-Proline methyl ester (2.48 g, 15 mmol, 3.0 eq.), HATU (3.8 g, 10.0 mmol, 2.0 eq.) and DIPEA (4.43 mL, 25.0 mmol, 5.0 eq.) was added, the reaction was stirred at for 2 h. The reaction was quenched by saturated *aq.* NH_4Cl, extracted with EtOAc for three times. The organic layers were combined, washed with 1N HCl *aq.*, and concentrated under vacuum to give the crude. The crude was dissolved in THF/MeOH/H_2O = 1:1:1 (50 mL, 0.1 M), and LiOH·H_2O (1.26 g, 30.0 mmol, 6.0 eq.) was added, the reaction was stirred at 25 °C for 1 h. The reaction was quenched by the addition of 1N HCl *aq.* and extracted with DCM/MeOH = 10:1 for three times. The organic layers were combined, dried over Na_2SO_4, and concentrated under vacuum. The crude was then dissolved in DMF (50 mL, 0.1 M), compound **30** (1.33 g, 5.50 mmol, 1.1 eq.), HATU (3.80 g, 10.0 mmol, 2.0 eq.), and DIPEA (1.8 mL, 25.0 mmol, 5.0 eq.) were added sequentially. The reaction was stirred at 25 °C for 2 h, quenched by saturated *aq.* NH_4Cl and extracted with EtOAc three times. The organic layers were combined, washed with 1N HCl *aq.*, dride over Na_2SO_4 and concentrated under vacuum to give the residue. The residue was filtered

thorough a pad of silicagel (EtOAc:Hexane 2:1) to give the crude. The crude was dissolved in toluene (250 mL, 0.02 M), $Pd(OAc)_2$ (554 mg, 2.47 mmol, 0.5 eq.), $t$$Bu_3P·HBF_4$ (1.43 g, 4.94 mmol, 1.0 eq.) and DIPEA (8.6 mL, 49.4 mmol, 10.0 eq.) was added. The reaction was stirred at 110 °C under nitrogen for 12 h. The solvent was removed under reduced pressure to give the residue, the residue was purified by silica gel chromatography to give the compound **71** (1.14 g, 31% yield from aryl iodide **71**). The characteristics data were identical to those previously reported by our group.[2]

Compound 71
Physical State: white solid
^{1}H NMR (600 MHz, $CDCl_3$): δ 7.77 – 7.70 (m, 1H), 7.66 (d, J = 8.6 Hz, 1H), 7.57 (s, 1H), 7.48 (s, 1H), 7.45 (d, J = 7.3 Hz, 1H), 7.02 – 6.88 (m, 2H), 5.49 – 5.40 (m, 1H), 4.97 (t, J = 9.3 Hz, 1H), 4.85 (t, J = 7.4 Hz, 1H), 4.36 (t, J = 6.5 Hz, 1H), 3.83 – 3.73 (m, 1H), 3.73 – 3.62 (m, 3H), 3.22 – 3.13 (m, 1H), 3.09 (d, J = 13.6 Hz, 1H), 2.97 (d, J = 14.4 Hz, 1H), 2.84 (s, 3H), 2.10 (dd, J = 12.9, 6.7 Hz, 1H), 1.95 – 1.86 (m, 2H), 1.77 (d, J = 12.4 Hz, 1H), 1.32 (s, 9H), 0.92 (dt, J = 92.4, 7.3 Hz, 9H), 0.87 (q, J = 7.3 Hz, 2H).
^{13}C NMR (151 MHz, $CDCl_3$): δ = 173.52, 171.58, 169.59, 169.49, 159.90, 158.26, 155.46, 136.48, 135.90, 133.39, 132.74, 131.77, 131.38, 131.10, 130.71, 127.51, 127.43, 126.54, 126.49, 120.95, 115.14, 114.99, 113.38, 79.55, 60.14, 52.57, 52.26, 51.35, 47.43, 35.87, 30.08, 29.48, 28.34, 26.62, 26.02, 24.97, 8.25, 6.31.
^{19}F NMR (565 MHz, $CDCl_3$): δ –121.97.
HRMS (ESI-TOF): calculated for $C_{39}H_{51}FN_4NaO_7Si^+$ $[M+Na]^+$: 757.3409, found: 757.3405.
TLC: R_f = 0.6 (1:1 EtOAc : Hexane, $Ce_2(SO_4)_3$ in phosphomolybdic acid).
$[\alpha]^{25}_D$: 51.4 (c = 0.2, MeOH)

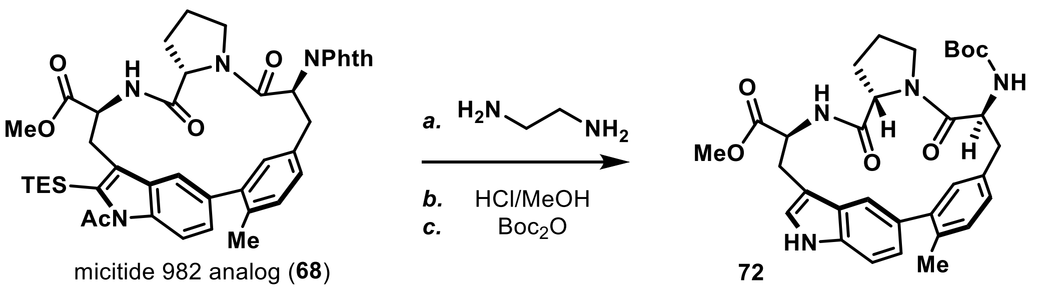

Compound **68** (840 mg, 1.10 mmol, 1.0 eq.) was dissolved in n-BuOH (11 mL, 0.1 M), and ethylenediamine (0.73 mL, 11 mmol, 10 eq.) was added. The reaction was stirred at 25 °C for 15 h. The reaction mixture was diluted with toluene (20mL) and directly concentrated under reduced pressure to give the crude. The crude was dissolved in MeOH (22 mL, 0.05 M), and SO_2Cl_2 (4.4 mL) was added dropwise at 0 °C. The reaction mixture was stirred at 25 °C for 2h. Then, the mixture was directly concentrated under reduced pressure to give the crude primary amine. The crure was then dissolved in the THF:saturated $aq.$ $NaHCO_3$ 10:1 (22 mL, 0.05 M). Boc_2O (1.77 mL, 7.70 mmol, 7 equiv.) was added and the reaction was stirred for 2h at 25 °C. After confirming the full conversion of the primary amine by LC/MS, the reaction was diluted with H_2O (200 mL) and extracted with EtOAc three times. The organic layers were combined, dried over Na_2SO_4, and concentrated under reduced pressure to give the crude. The crude was purified by flash column chromatography (EtOAc:Hexane 1:4 to 4:1) to give the compound **72** (630 mg, 99% yield).

Compound 72

Physical State: white solid

^{1}H NMR (400 MHz, $CDCl_3$): δ 8.25 (s, 1H), 7.43 (s, 1H), 7.27 (dd, *J* = 6.4, 1.8 Hz, 2H), 7.25 – 7.21 (m, 1H), 7.17 (d, *J* = 7.7 Hz, 1H), 6.98 – 6.94 (m, 2H), 6.86 (t, *J* = 8.3 Hz, 1H), 5.28 (d, *J* = 8.5 Hz, 1H), 4.83 – 4.73 (m, 2H), 4.53 (dd, *J* = 8.3, 3.2 Hz, 1H), 3.84 – 3.75 (m, 1H), 3.72 (s, 3H), 3.71 – 3.63 (m, 1H), 3.24 (dd, *J* = 15.0, 5.7 Hz, 1H), 3.17 (dd, *J* = 13.5, 6.3 Hz, 1H), 3.10 (dd, *J* = 13.5, 2.1 Hz, 1H), 3.03 – 2.93 (m, 1H), 2.40 (s, 3H), 2.25 – 1.89 (m, 4H), 1.45 (s, 9H).

^{13}C NMR (101 MHz, $CDCl_3$): δ 173.10, 170.51, 169.19, 154.77, 141.34, 134.71, 132.95, 132.73, 132.32, 131.43, 129.41, 127.97, 126.40, 123.05, 121.25, 120.88, 113.98, 108.88, 78.86, 59.41, 53.14, 51.84, 51.15, 46.49, 35.57, 30.34, 28.90, 27.81, 25.33, 24.20, 20.24.

HRMS (ESI-TOF): calculated for $C_{32}H_{38}N_4NaO_6^+$ $[M+Na]^+$: 597.2689, found: 597.2686.

TLC: R_f = 0.6 (1:0 EtOAc : Hexane, $Ce_2(SO_4)_3$ in phosphomolybdic acid).

$[\alpha]^{21}_D$: +84.1 (*c* = 1.0, $CHCl_3$)

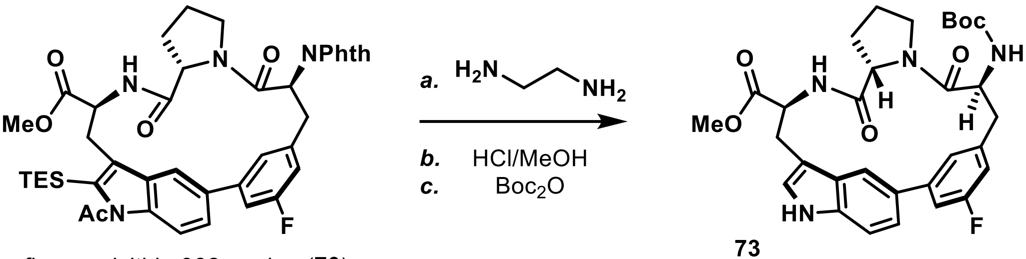

fluoro-micitide 982 analog (**70**) **73**

Compound **70** (507 mg, 0.663 mmol, 1.0 eq.) was dissolved in *n*-BuOH (6 mL, 0.1 M), and ethylenediamine (0.44 mL, 6.6 mmol, 10 eq.) was added. The reaction was stirred at 25 °C for 15 h. The reaction mixture was diluted with toluene (15 mL) and directly concentrated under reduced pressure to give the crude. The crude was dissolved in MeOH (12 mL, 0.05 M), and SO_2Cl_2 (2.4 mL) was added dropwise at 0 °C. The reaction mixture was stirred at 25 °C for 2h. Then, the mixture was directly concentrated under reduced pressure to give the crude primary amine. The crure was then dissolved in the THF:saturated *aq.* $NaHCO_3$ 10:1 (12 mL, 0.05 M). Boc_2O (1.1 mL, 4.6 mmol, 7 equiv.) was added and the reaction was stirred for 2h at 25 °C. After confirming the full conversion of the primary amine by LC/MS, the reaction was diluted with H_2O (200 mL) and extracted with EtOAc three times. The organic layers were combined, dried over Na_2SO_4, and concentrated under reduced pressure to give the crude. The crude was purified by flash column chromatography (EtOAc:Hexane 1:4 to 4:1) to give the compound **73** (371 mg, 97% yield).

Compound 73

Physical State: white solid

^{1}H NMR (400 MHz, CD_3OD): δ 7.56 (s, 1H), 7.52 (dd, *J* = 8.6, 4.9 Hz, 2H), 7.35 (d, *J* = 8.7 Hz, 1H), 7.09 (d, *J* = 5.4 Hz, 1H), 7.06 – 6.98 (m, 2H), 4.87 (d, *J* = 11.4 Hz, 1H), 4.80 – 4.75 (m, 1H), 4.67 – 4.59 (m, 1H), 3.88 – 3.80 (m, 2H), 3.74 (s, 3H), 3.42 (dt, *J* = 13.4, 6.3 Hz, 1H), 3.18 (t, *J* = 6.0 Hz, 2H), 3.04 (d, *J* = 15.1 Hz, 1H), 2.80 (d, *J* = 6.5 Hz, 1H), 2.27 – 1.93 (m, 4H), 1.44 (s, 9H).

^{13}C NMR (101 MHz, $CDCl_3$): δ 172.59, 171.74, 169.19, 157.27, 155.01, 135.83, 133.06, 130.03, 128.98, 126.29, 125.81, 122.00, 121.93, 121.77, 119.21, 114.15, 113.92, 113.30, 109.46, 78.93, 59.24, 52.49, 50.75, 34.41, 29.40, 28.64, 26.62, 23.85.

^{19}F NMR (377 MHz, CD_3OD): δ -123.39.

HRMS (ESI-TOF): calculated for $C_{31}H_{35}FN_4NaO_6^+$ $[M+Na]^+$: 601.2438, found: 601.2437.

TLC: R_f = 0.5 (1:0 EtOAc : Hexane, $Ce_2(SO_4)_3$ in phosphomolybdic acid).
$[\alpha]^{21}_D$: +92.1 (c = 1.0, $CHCl_3$)

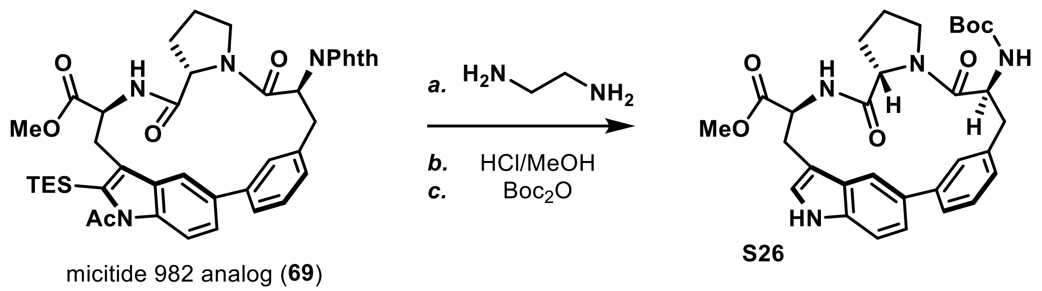

Compound **69** (200 mg, 0.268 mmol, 1.0 eq.) was dissolved in *n*-BuOH (5 mL, 0.1 M), and ethylenediamine (0.18 mL, 2.7 mmol, 10 eq.) was added. The reaction was stirred at 25 °C for 15 h. The reaction mixture was diluted with toluene (15 mL) and directly concentrated under reduced pressure to give the crude. The crude was dissolved in MeOH (5 mL, 0.1 M), and SO_2Cl_2 (1.0 mL) was added dropwise at 0 °C. The reaction mixture was stirred at 25 °C for 2h. Then, the mixture was directly concentrated under reduced pressure to give the crude primary amine. The crure was then dissolved in the THF:saturated *aq.* $NaHCO_3$ 10:1 (5.5 mL, 0.1 M). Boc_2O (0.43 mL, 1.9 mmol, 7.0 equiv.) was added and the reaction was stirred for 2h at 25 °C. After confirming the full conversion of the primary amine by LC/MS, the reaction was diluted with H_2O (200 mL) and extracted with EtOAc three times. The organic layers were combined, dried over Na_2SO_4, and concentrated under reduced pressure to give the crude. The crude was purified by flash column chromatography (EtOAc:Hexane 1:4 to 4:1) to give the compound **S26** (148 mg, 99% yield).

Compound S26
Physical State: white solid
^{1}H NMR (600 MHz, $CDCl_3$): δ 8.08 (s, 1H), 7.65 (s, 1H), 7.56 (s, 1H), 7.50 (d, J = 7.2 Hz, 1H), 7.44 (dd, J = 8.4, 1.9 Hz, 1H), 7.36 – 7.28 (m, 1H), 7.30 (t, J = 7.6 Hz, 1H), 5.20 (s, 1H), 5.04 – 4.95 (m, 1H), 4.82 (ddd, J = 8.2, 5.4, 2.7 Hz, 1H), 4.58 – 4.51 (m, 1H), 3.91 – 3.84 (m, 1H), 3.73 (t, J = 3.2 Hz, 1H), 3.72 (s, 3H), 3.30 – 3.16 (m, 3H), 3.06 – 3.00 (m, 1H), 2.25 – 2.19 (m, 1H), 2.13 – 2.07 (m, 1H), 2.04 – 1.93 (m, 2H), 1.41 (s, 9H).
^{13}C NMR (151 MHz, $CDCl_3$): δ 173.84, 171.45, 169.93, 155.49, 141.90, 136.06, 134.99, 132.85, 130.52, 128.75, 127.90, 127.35, 125.31, 122.04, 121.32, 119.46, 114.47, 111.03, 79.53, 60.23, 53.08, 52.53, 51.81, 47.32, 36.59, 31.22, 29.58, 28.47, 25.06.
HRMS (ESI-TOF): calculated for $C_{31}H_{36}N_4NaO_6^+$ $[M+Na]^+$: 583.2533, found: 583.2535.
TLC: R_f = 0.6 (1:0 EtOAc : Hexane, $Ce_2(SO_4)_3$ in phosphomolybdic acid).
$[\alpha]^{21}_D$: +169.6 (c = 1.0, $CHCl_3$)

Key NOESY Correlations of Compound S26

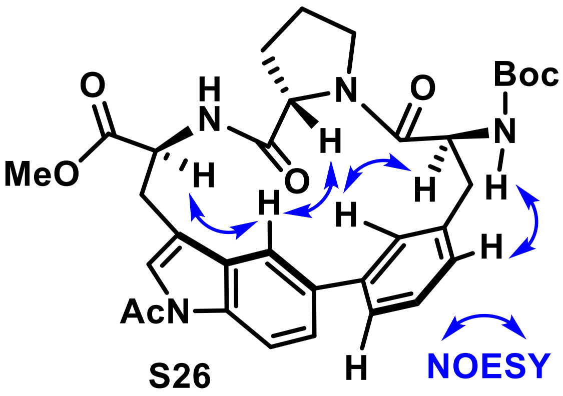

S26 (CDCl$_3$, 400 MHz)

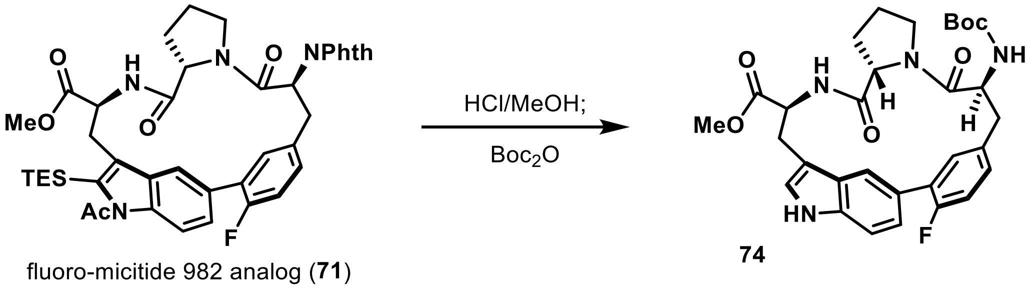

fluoro-micitide 982 analog (**71**)

Compound **71** (651 mg, 0.886 mmol, 1.0 eq.) was dissolved in MeOH (18 mL, 0.05 M), and SO_2Cl_2 (3.6 mL) was added dropwise at 0 °C. The reaction mixture was stirred at 25 °C for 2h. Then, the mixture was directly concentrated under reduced pressure to give the crude primary amine. The crude was then dissolved in the THF:saturated *aq.* $NaHCO_3$ 10:1 (36 mL, 0.05 M). Boc_2O (1.8 mL, 6.2 mmol, 7 equiv.) was added and the reaction was stirred for 2h at 25 °C. After confirming the full conversion of the primary amine by LC/MS, the reaction was diluted with H_2O (100 mL) and extracted with EtOAc three times. The organic layers were combined, dried over Na_2SO_4, and concentrated under reduced pressure to give the crude. The crude was purified by flash column chromatography (EtOAc:Hexane 1:4 to 4:1) to give the compound **74** (487 mg, 95% yield).

Compound 74
Physical State: white solid
^{1}H NMR (600 MHz, CDCl$_3$): δ 7.52 (s, 2H), 7.45 (dd, *J* = 5.2, 2.6 Hz, 1H), 7.28 (d, *J* = 5.3 Hz, 1H), 7.14 (s, 1H), 7.02 – 6.92 (m, 1H), 6.97 – 6.87 (m, 2H), 5.31 (t, *J* = 17.1 Hz, 1H), 4.93 – 4.84 (m, 1H), 4.78 (t, *J* = 7.4 Hz, 1H), 4.45 (s, 1H), 3.74 (s, 3H), 3.74 – 3.65 (m, 2H), 3.27 – 3.06 (m, 3H), 2.99 (t, *J* = 15.5 Hz, 1H), 2.17 – 1.80 (m, 4H), 1.37 (s, 9H).
^{13}C NMR (151 MHz, CDCl$_3$): δ 173.92, 171.44, 169.63, 159.98, 158.34, 155.50, 136.01, 133.98, 130.68, 130.20, 129.24, 127.14, 123.56, 121.99, 120.77, 114.97, 114.51, 110.38, 79.62, 60.19, 54.47, 54.44, 53.25, 52.50, 51.63, 47.32, 38.70, 35.82, 31.11, 29.65, 28.44, 24.91.
^{19}F NMR (565 MHz, CDCl$_3$): δ -122.22.
HRMS (ESI-TOF): calculated for $C_{31}H_{35}FN_4NaO_6^+$ [M+Na]$^+$: 601.2438, found: 601.2440.
TLC: R_f = 0.5 (1:0 EtOAc : Hexane, $Ce_2(SO_4)_3$ in phosphomolybdic acid).
$[\alpha]^{21}_D$: +286.5 (*c* = 1.0, CHCl$_3$)

Skeletal Editing Employing Unnatural Micitide Core

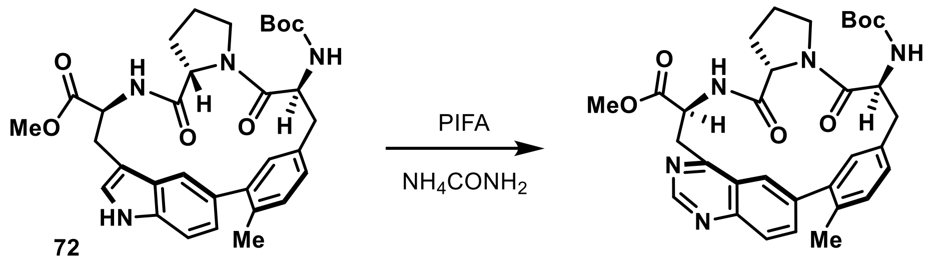

72

78

In a culture tube equipped with a stirring bar, compound **72** (21.3 mg, 37.1 μmol, 1.0 eq.) and ammonium carbamate (29.0 mg, 371 μmol, 10.0 equiv) were dissolved in MeOH (2 mL, 0.02 M). The resulting solution was cooled to 0 °C, and then PIFA (63.8 mg, 148 μmol, 4.0 equiv) was added in one portion. The reaction was stirred at 0 °C for 1h and quenched with saturated aq. $Na_2S_2O_3$. The mixture was extracted with EtOAc (5mL) three times. The organic layers were combined and washed with brine and concentrated under reduced pressure to give the crude. The crude was purified by preparative thin-layer chromatography (EtOAc:Hexane 9:1) to give the title compound **78** (13.4 mg, 61% yield)

Compound 78
Physical State: white solid
^{1}H NMR (400 MHz, $CDCl_3$, A mixture of rotamers A:B = 2.4:1, Signals of the major rotamer were reported): δ 9.21 (s, 9H), 8.13 (s, 1H), 8.04 (d, *J* = 4.2 Hz, 1H), 7.54 (s, 1H), 7.23 (d, *J* = 8.3 Hz, 1H), 6.96 – 6.85 (m, 2H), 5.16 (d, *J* = 9.5 Hz, 1H), 4.84 (q, *J* = 8.5 Hz, 1H), 4.46 (dd, *J* = 7.9, 4.6 Hz, 1H), 3.86 (dd, *J* = 13.8, 4.7 Hz, 2H), 3.76 (s, 3H), 3.66 (d, *J* = 1.1 Hz, 1H), 3.55 (dd, *J* = 12.9, 10.3 Hz, 1H), 3.32 (dd, *J* = 13.4, 7.3 Hz, 1H), 3.16 (dd, *J* = 36.2, 13.2 Hz, 2H), 2.35 (s, 3H), 2.10 – 1.98 (m, 2H), 1.80 – 1.61 (m, 2H), 1.44 (s, 9H).
^{13}C NMR (101 MHz, $CDCl_3$, A mixture of rotamers A:B = 2.4:1): δ 171.25, 170.60, 169.14, 166.47, 160.85, 153.62, 153.46, 149.19, 148.67, 141.21, 139.15, 135.15, 132.41, 130.01, 129.16, 127.15, 125.60, 125.21, 123.30, 122.71, 117.13, 112.13, 79.21, 79.00, 66.18, 59.80, 59.55, 52.19, 52.12, 50.91, 50.80, 50.51, 46.82, 46.70, 40.06, 39.28, 35.47, 35.33, 33.38, 29.77, 29.08, 28.83, 27.77, 27.70, 24.65, 24.48, 23.14, 22.34, 19.95.
HRMS (ESI-TOF): calculated for $C_{32}H_{37}N_5NaO_6^+$ [M+Na]$^+$: 610.2642, found: 610.2643.
TLC: R_f = 0.6 (1:0 EtOAc : Hexane, $Ce_2(SO_4)_3$ in phosphomolybdic acid).
[α]$^{21}_{D}$: +160.2 (*c* = 0.5, $CHCl_3$)

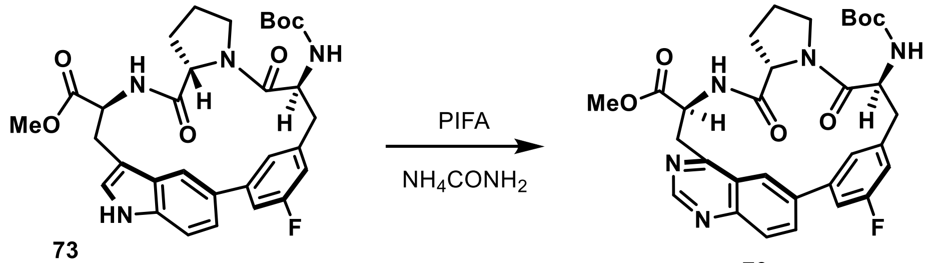

73

79

In a culture tube equipped with a stirring bar, compound **73** (41.4 mg, 71.6 μmol, 1.0 eq.) and ammonium carbamate (55.6 mg, 712 μmol, 10.0 equiv) were dissolved in MeOH (4 mL, 0.02 M). The resulting solution was cooled to 0 °C, and then PIFA (122 mg, 28.5 μmol, 4.0 equiv) was added in one portion. The reaction was stirred at 0 °C for 1h and quenched with saturated aq. $Na_2S_2O_3$. The mixture was extracted with EtOAc (5mL) three times. The organic layers were combined and washed with brine and concentrated under reduced

pressure to give the crude. The crude was purified by preparative thin-layer chromatography (EtOAc:Hexane 9:1) to give the title compound **79** (25.3 mg, 60% yield)

Compound 79
Physical State: white solid
^{1}H NMR (400 MHz, CD_3OD): δ 9.13 (s, 1H), 8.37 – 8.29 (m, 1H), 8.16 (s, 1H), 8.08 (d, *J* = 9.1 Hz, 1H), 7.65 (d, *J* = 7.6 Hz, 1H), 7.23 – 7.12 (m, 2H), 5.08 (d, *J* = 9.8 Hz, 1H), 4.85 – 4.77 (m, 1H), 4.55 (dd, *J* = 8.4, 5.3 Hz, 1H), 3.99 (d, *J* = 13.7 Hz, 1H), 3.89 – 3.80 (m, 2H), 3.70 (s, 3H), 3.66 – 3.54 (m, 1H), 3.30 – 3.13 (m, 2H), 2.38 – 2.25 (m, 1H), 2.22 – 2.10 (m, 1H), 2.09 – 1.99 (m, 1H), 1.99 – 1.91 (m, 1H), 1.45 (s, 9H).
^{13}C NMR (101 MHz, CD_3OD): δ 171.80, 170.50, 169.17, 167.97, 159.73, 152.86, 148.45, 135.95, 135.24, 135.17, 132.93, 132.03, 131.95, 127.04, 125.72 (d, *J* = 15.2 Hz), 124.48, 122.40, 114.53 (d, *J* = 23.3 Hz), 78.99, 59.37, 50.95, 50.58, 38.77, 34.35, 28.52, 26.61, 24.10.
^{19}F NMR (565 MHz, CD_3OD): δ -122.62.
HRMS (ESI-TOF): calculated for $C_{31}H_{34}FN_5NaO_6^+$ $[M+Na]^+$: 614.2391, found: 610.2394.
TLC: R_f = 0.5 (1:0 EtOAc : Hexane, $Ce_2(SO_4)_3$ in phosphomolybdic acid).
$[\alpha]^{21}{}_D$: +91.9 (*c* = 0.5, $CHCl_3$)

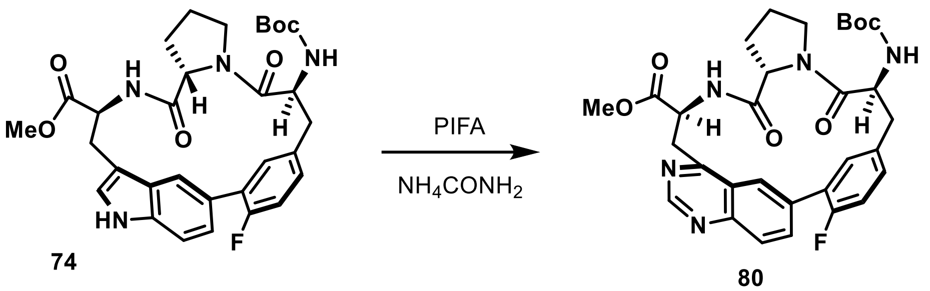

74 80

In a culture tube equipped with a stirring bar, compound **74** (41.2 mg, 71.2 μmol, 1.0 eq.) and ammonium carbamate (55.6 mg, 712 μmol, 10.0 equiv) were dissolved in MeOH (4 mL, 0.02 M). The resulting solution was cooled to 0 °C, and then PIFA (122 mg, 28.5 μmol, 4.0 equiv) was added in one portion. The reaction was stirred at 0 °C for 1h and quenched with saturated aq. $Na_2S_2O_3$. The mixture was extracted with EtOAc (5mL) three times. The organic layers were combined and washed with brine and concentrated under reduced pressure to give the crude. The crude was purified by preparative thin-layer chromatography (EtOAc:Hexane 9:1) to give the title compound **80** (20.1 mg, 48% yield)

Compound 80
Physical State: white solid
^{1}H NMR (400 MHz, $CDCl_3$): δ 9.22 (s, 1H), 8.26 (dd, *J* = 8.6, 3.6 Hz, 1H), 8.09 (d, *J* = 8.8 Hz, 1H), 8.06 (s, 1H), 7.59 (d, *J* = 7.3 Hz, 1H), 7.32 (dd, *J* = 11.8, 7.4 Hz, 1H), 7.15 – 7.02 (m, 2H), 5.29 (q, *J* = 5.7 Hz, 1H), 5.07 (t, *J* = 9.8 Hz, 1H), 4.86 (t, *J* = 7.7 Hz, 1H), 4.41 (dd, *J* = 7.6, 4.8 Hz, 1H), 3.85 – 3.75 (m, 3H), 3.76 (s, 3H), 3.51 (ddd, *J* = 13.0, 10.1, 2.2 Hz, 1H), 3.26 (dd, *J* = 13.6, 7.1 Hz, 1H), 3.14 (d, *J* = 13.6 Hz, 1H), 2.16 (d, *J* = 13.7 Hz, 2H), 1.94 (d, *J* = 11.7 Hz, 2H), 1.39 (s, 9H).
^{13}C NMR (101 MHz, $CDCl_3$): δ 171.47, 170.79, 169.11, 166.52, 159.71, 157.25, 154.66, 153.64, 148.97, 135.70, 134.96, 132.91, 132.09, 130.75, 128.04, 125.94, 124.39, 122.62, 114.90, 114.68, 79.08, 59.63, 52.11, 50.72, 50.58, 46.82, 39.61, 35.01, 28.93, 27.70, 24.46.
^{19}F NMR (377 MHz, $CDCl_3$): δ -121.16.
HRMS (ESI-TOF): calculated for $C_{31}H_{34}FN_5NaO_6^+$ $[M+Na]^+$: 614.2391, found: 610.2396.

TLC: R_f = 0.5 (1:0 EtOAc : Hexane, $Ce_2(SO_4)_3$ in phosphomolybdic acid).
[α]^{21}D: +103.2 (*c* = 0.1, $CHCl_3$)

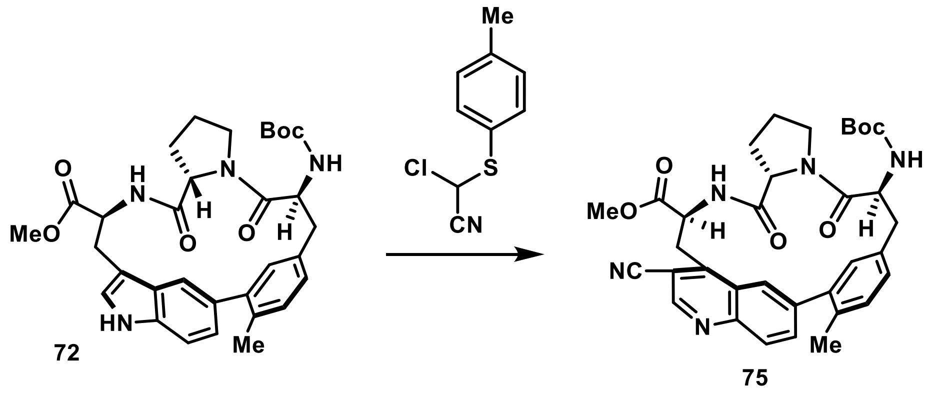

To a solution of compound **72** (43.8 mg, 76.3 μmol, 1.0 eq.) and Cs_2CO_3 (125 mg, 382 μmol, 5.0 eq.) in THF (2 mL, 0.05 M), 2-chloro-2-(p-tolylthio)acetonitrile (22.5 mg, 152 μmol, 1.5 eq.) was added portion-wise (0.5 equiv. every 1 h) at room temperature, and the resulting mixture was stirred for next 4 h. The reaction was quenched with saturated aq. $Na_2S_2O_3$, extracted with EtOAc (5mL) three times. The organic layers were combined and washed with brine and concentrated under reduced pressure to give the crude. The crude was purified by preparative thin-layer chromatography (EtOAc:Hexane 3:1) to give the title compound **75** (40.2 mg, 63% yield)

Compound 75
Physical State: pale yellow solid
^{1}H NMR (400 MHz, $CDCl_3$, a mixture of two rotamers A:B = 2.2:1, Signals of the major rotamers were reported): δ 8.98 (s, 1H), 8.25 (d, *J* = 8.7 Hz, 1H), 8.14 (s, 1H), 8.09 (d, *J* = 8.7 Hz, 1H), 7.24 – 6.88 (m, 3H), 5.19 (d, *J* = 7.7 Hz, 1H), 4.84 (q, *J* = 7.4 Hz, 1H), 4.50 – 4.40 (m, 1H), 3.83 (s, 3H), 3.82 (s, 2H), 3.71 (t, *J* = 9.5 Hz, 2H), 3.32 (dd, *J* = 13.9, 7.0 Hz, 1H), 3.18 (d, *J* = 13.3 Hz, 1H), 3.13 – 3.04 (m, 1H), 2.27 (s, 3H), 2.19 (d, *J* = 10.9 Hz, 2H), 2.01 (s, 2H), 1.43 (s, 9H).
^{13}C NMR (101 MHz, $CDCl_3$, a mixture of two rotamers A:B = 2.2:1): δ ^{13}C NMR (101 MHz, $CDCl_3$) δ 170.94, 170.77, 170.59, 170.53, 169.19, 169.06, 163.25, 160.81, 154.62, 154.57, 149.14, 148.85, 148.76, 148.68, 147.83, 147.33, 141.99, 140.42, 140.35, 140.00, 138.68, 137.60, 137.52, 133.90, 133.09, 132.17, 132.11, 131.06, 130.71, 130.10, 129.95, 128.69, 125.50, 125.47, 124.53, 124.50, 124.28, 122.61, 117.34, 117.13, 116.29, 116.15, 112.47, 112.25, 107.34, 106.88, 79.26, 79.07, 69.85, 66.18, 59.75, 59.36, 52.85, 52.78, 50.89, 50.76, 50.62, 50.26, 46.85, 46.68, 38.09, 35.77, 35.55, 35.49, 35.21, 33.38, 31.30, 29.77, 29.07, 28.79, 28.74, 28.71, 28.49, 28.28, 27.77, 27.69, 24.65, 24.42, 23.85, 23.14, 22.34, 22.07, 20.03.
HRMS (ESI-TOF): calculated for $C_{34}H_{37}N_5NaO_6^+$ $[M+Na]^+$: 634.2642, found: 634.2643.
TLC: R_f = 0.5 (3:1 EtOAc : Hexane, $Ce_2(SO_4)_3$ in phosphomolybdic acid).
[α]^{21}D: +115.9 (*c* = 1.0, $CHCl_3$)

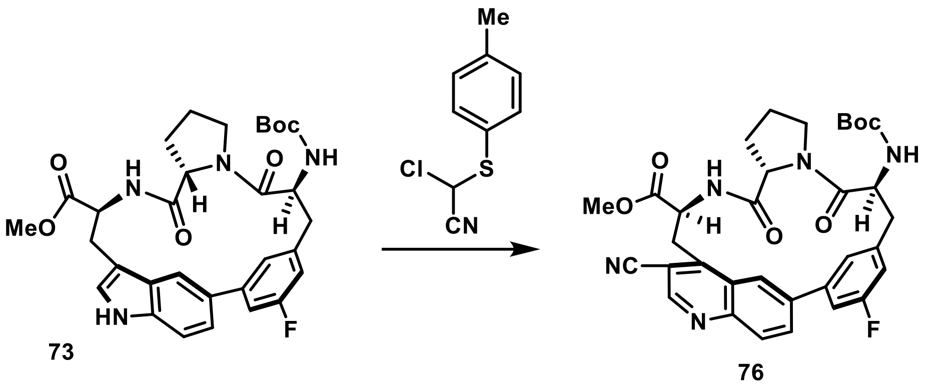

To a solution of compound **73** (43.8 mg, 76.3 μmol, 1.0 eq.) and Cs_2CO_3 (125 mg, 382 μmol, 5.0 eq.) in THF (2 mL, 0.05 M), 2-chloro-2-(p-tolylthio)acetonitrile (30.1 mg, 152 μmol, 2.0 eq.) was added portion-wise (0.5 equiv. every 1 h) at room temperature, and the resulting mixture was stirred for next 4 h. The reaction was quenched with saturated aq. $Na_2S_2O_3$, extracted with EtOAc (5mL) three times. The organic layers were combined and washed with brine and concentrated under reduced pressure to give the crude. The crude was purified by preparative thin-layer chromatography (EtOAc:Hexane 3:1) to give the title compound **76** (27.2 mg, 58% yield)

Compound 76
Physical State: pale yellow solid
^{1}H NMR (400 MHz, CD_3OD): δ 9.00 (s, 1H), 8.28 – 8.06 (m, 3H), 7.70 – 7.60 (m, 1H), 7.25 – 7.10 (m, 2H), 5.03 (d, *J* = 9.0 Hz, 1H), 4.80 (d, *J* = 7.1 Hz, 1H), 4.57 (dd, *J* = 8.8, 5.1 Hz, 1H), 3.90 – 3.79 (m, 4H), 3.77 (s, 3H), 3.31 – 3.20 (m, 1H), 3.19 – 3.08 (m, 1H), 2.32 (dt, *J* = 12.6, 7.5 Hz, 1H), 2.23 – 2.12 (m, 1H), 2.12 – 2.00 (m, 1H), 2.01 – 1.94 (m, 1H), 1.48 (s, 9H).
^{13}C NMR (101 MHz, CD_3OD): δ 172.33, 170.93, 169.71, 157.89, 155.64, 150.76, 149.68, 147.73, 136.85, 133.89 (d, *J* = 7.6 Hz), 133.43, 132.58 (d, *J* = 8.2 Hz), 131.86, 129.17, 125.06, 124.36, 116.24, 115.16 (d, *J* = 23.1 Hz), 108.00, 79.65, 59.93, 52.09, 51.48, 51.24, 35.44, 35.15, 29.10, 27.29, 27.26, 24.70.
^{19}F NMR (377 MHz, CD_3OD): δ -122.30.
HRMS (ESI-TOF): calculated for $C_{33}H_{34}FN_5NaO_6^+$ [M+Na]$^+$: 638.2391, found: 638.2391.
TLC: R_f = 0.5 (3:1 EtOAc : Hexane, $Ce_2(SO_4)_3$ in phosphomolybdic acid).
$[\alpha]^{21}_D$: +98.5 (*c* = 0.3, $CHCl_3$)

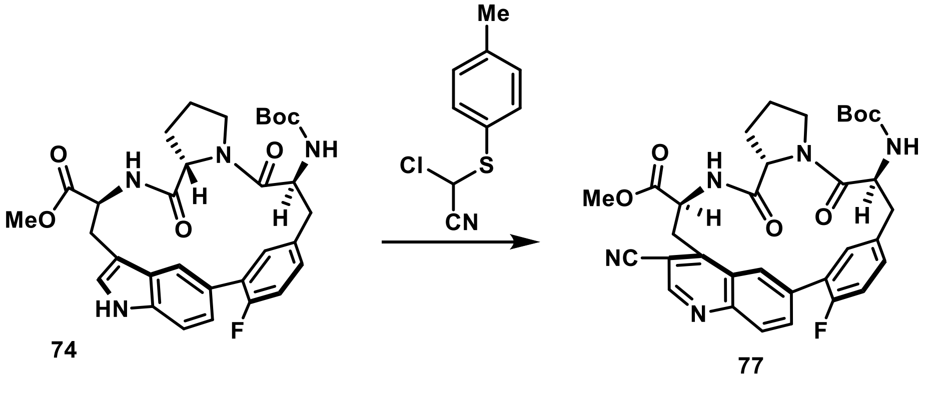

To a solution of compound **74** (43.8 mg, 76.3 μmol, 1.0 eq.) and Cs_2CO_3 (125 mg, 382 μmol, 5.0 eq.) in THF (2 mL, 0.05 M), 2-chloro-2-(p-tolylthio)acetonitrile (30.1 mg, 152 μmol, 2.0 eq.) was added portion-wise (0.5 equiv. every 1 h) at room temperature, and the resulting mixture was stirred for next 4 h. The reaction was

quenched with saturated aq. $Na_2S_2O_3$, extracted with EtOAc (5mL) three times. The organic layers were combined and washed with brine and concentrated under reduced pressure to give the crude. The crude was purified by preparative thin-layer chromatography (EtOAc:Hexane 3:1) to give the title compound **77** (46.6 mg, 57% yield)

Compound 77

Physical State: pale yellow solid

^{1}H NMR (400 MHz, CD_3OD): δ 8.99 (s, 1H), 8.20 (s, 2H), 8.01 (s, 1H), 7.60 (dd, *J* = 7.8, 2.1 Hz, 1H), 7.50 (d, *J* = 10.0 Hz, 1H), 7.16 – 7.03 (m, 2H), 5.30 (d, *J* = 8.4 Hz, 1H), 5.01 (dt, *J* = 10.0, 5.7 Hz, 1H), 4.86 (t, *J* = 7.7 Hz, 1H), 4.38 (dd, *J* = 7.6, 4.9 Hz, 1H), 3.83 (s, 3H), 3.82 – 3.72 (m, 2H), 3.67 (d, *J* = 5.9 Hz, 2H), 3.33 – 3.22 (m, 1H), 3.15 – 3.06 (m, 1H), 2.23 – 1.88 (m, 4H), 1.39 (s, 9H).

^{13}C NMR (101 MHz, CD_3OD): δ 170.97, 170.67, 169.06, 159.66, 157.19, 154.65, 148.86 (d, *J* = 9.2 Hz), 147.61, 136.44, 133.57 (d, *J* = 8.0 Hz), 132.74, 132.32 (d, *J* = 8.5 Hz), 130.80, 129.58, 124.41, 123.56, 116.21, 114.91 (d, *J* = 22.5 Hz), 107.03, 79.15, 59.58, 52.86, 50.70, 50.38, 46.83, 35.35, 35.10, 28.85, 27.70, 24.45.

^{19}F NMR (565 MHz, CD_3OD): δ -124.57.

HRMS (ESI-TOF): calculated for $C_{33}H_{34}FN_5NaO_6^+$ $[M+Na]^+$: 638.2391, found: 638.2394.

TLC: R_f = 0.5 (3:1 EtOAc : Hexane, $Ce_2(SO_4)_3$ in phosphomolybdic acid).

$[\alpha]^{21}_D$: +90.1 (*c* = 0.3, $CHCl_3$)

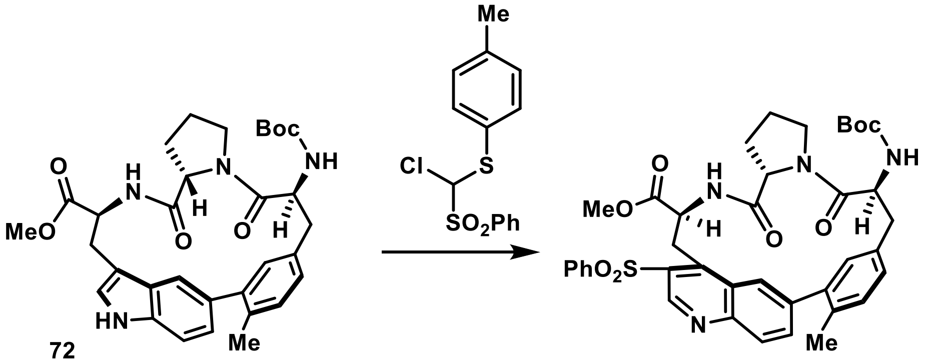

72 81

To a solution of compound **72** (43.8 mg, 76.3 μmol, 1.0 eq.) and Cs_2CO_3 (125 mg, 382 μmol, 5.0 eq.) in THF (2 mL, 0.05 M), (chloro(phenylsulfonyl)methyl)(p-tolyl)sulfane (47.6 mg, 152 μmol, 2.0 eq.) was added portion-wise (0.5 equiv. every 1 h) at room temperature, and the resulting mixture was stirred for next 4 h. The reaction was quenched with saturated aq. $Na_2S_2O_3$, extracted with EtOAc (5mL) three times. The organic layers were combined and washed with brine and concentrated under reduced pressure to give the crude. The crude was purified by preparative thin-layer chromatography (EtOAc:Hexane 3:1) to give the title compound **81** (28.8 mg, 52% yield)

Compound 81

Physical State: pale yellow solid

^{1}H NMR (400 MHz, $CDCl_3$. A mixture of rotamers A:B = 2.6:1, Signals of the major rotamers were reported): δ 8.99 (s, 1H), 8.20 (s, 2H), 8.01 (s, 1H), 7.60 (dd, *J* = 7.8, 2.1 Hz, 1H), 7.50 (d, *J* = 10.0 Hz, 1H), 7.16 – 7.03 (m, 2H), 5.30 (d, *J* = 8.4 Hz, 1H), 5.01 (dt, *J* = 10.0, 5.7 Hz, 1H), 4.86 (t, *J* = 7.7 Hz, 1H), 4.38 (dd, *J* = 7.6, 4.9 Hz, 1H), 3.83 (s, 3H), 3.82 – 3.72 (m, 2H), 3.67 (d, *J* = 5.9 Hz, 2H), 3.33 – 3.22 (m, 1H), 3.15 – 3.06 (m, 1H), 2.23 – 1.88 (m, 4H), 1.39 (s, 9H).

^{13}C NMR (101 MHz, $CDCl_3$, A mixture of rotamers A:B = 2.6:1) : δ 170.97, 170.67, 169.06, 159.66, 157.19, 154.65, 148.86 (d, J = 9.2 Hz), 147.61, 136.44, 133.57 (d, J = 8.0 Hz), 132.74, 132.32 (d, J = 8.5 Hz), 130.80, 129.58, 124.41, 123.56, 116.21, 114.91 (d, J = 22.5 Hz), 107.03, 79.15, 59.58, 52.86, 50.70, 50.38, 46.83, 35.35, 35.10, 28.85, 27.70, 24.45.
HRMS (ESI-TOF): calculated for $C_{39}H_{42}N_4NaO_8S^+$ [M+Na]$^+$: 749.2621, found: 749.2617.
TLC: R_f = 0.5 (3:1 EtOAc : Hexane, $Ce_2(SO_4)_3$ in phosphomolybdic acid).
[α]$^{21}_D$: +36.2 (c = 0.5, $CHCl_3$)

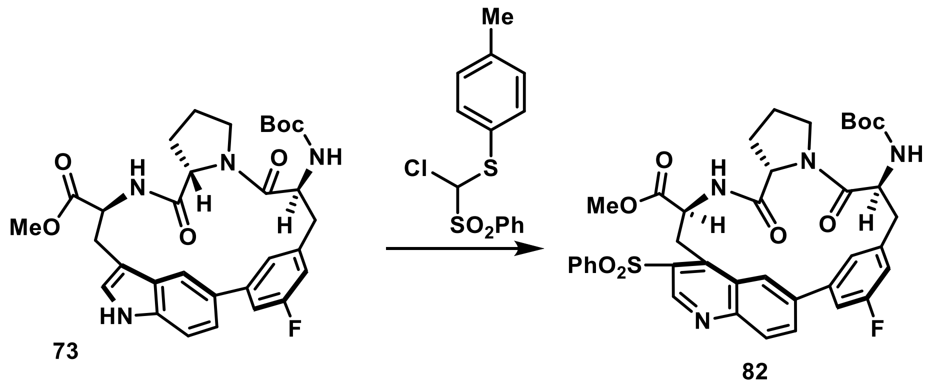

73

82

To a solution of compound **73** (43.8 mg, 76.3 μmol, 1.0 eq.) and Cs_2CO_3 (125 mg, 382 μmol, 5.0 eq.) in THF (2 mL, 0.05 M), (chloro(phenylsulfonyl)methyl)(p-tolyl)sulfane (47.6 mg, 152 μmol, 2.0 eq.) was added portion-wise (0.5 equiv. every 1 h) at room temperature, and the resulting mixture was stirred for next 4 h. The reaction was quenched with saturated aq. $Na_2S_2O_3$, extracted with EtOAc (5mL) three times. The organic layers were combined and washed with brine and concentrated under reduced pressure to give the crude. The crude was purified by preparative thin-layer chromatography (EtOAc:Hexane 3:1) to give the title compound **82** (14.5 mg, 27% yield)

Compound 82
Physical State: pale yellow solid
^{1}H NMR (600 MHz, CD_3OD): δ 9.35 (s, 1H), 8.20 (s, 1H), 8.13 (d, J = 14.4 Hz, 2H), 7.93 (s, 2H), 7.68 – 7.58 (m, 1H), 7.62 – 7.52 (m, 3H), 7.19 – 7.15 (m, 1H), 7.13 – 7.06 (m, 1H), 5.04 (d, J = 11.3 Hz, 1H), 4.76 (d, J = 7.7 Hz, 1H), 4.62 – 4.55 (m, 1H), 4.50 (t, J = 4.4 Hz, 3H), 3.85 – 3.78 (m, 1H), 3.77 – 3.69 (m, 1H), 3.65 (s, 3H), 3.59 (dd, J = 13.9, 1.7 Hz, 1H), 3.23 – 3.17 (m, 1H), 3.11 – 3.05 (m, 1H), 2.25 – 2.19 (m, 1H), 2.17 – 2.06 (m, 1H), 1.98 – 1.92 (m, 1H), 1.91 – 1.84 (m, 1H), 1.39 (s, 9H).
^{13}C NMR (151 MHz, CD_3OD) : δ 172.41, 170.79, 169.91, 160.01, 158.37, 155.73, 148.43, 148.14, 146.51, 141.92, 137.02, 134.07 (d, J = 7.5 Hz), 133.83, 133.51, 133.21, 132.62, 131.97, 129.61, 129.22, 127.12, 126.16, 124.84, 115.22 (d, J = 22.6 Hz), 79.69, 60.01, 52.62, 51.66, 51.52, 35.23, 31.58, 29.05, 27.32, 24.77.
^{19}F NMR (565 MHz, CD_3OD): δ -122.19.
HRMS (ESI-TOF): calculated for $C_{38}H_{39}FN_4NaO_8S^+$ [M+Na]$^+$: 753.2370, found: 753.2371.
TLC: R_f = 0.5 (3:1 EtOAc : Hexane, $Ce_2(SO_4)_3$ in phosphomolybdic acid).
[α]$^{21}_D$: +71.2 (c = 0.3, $CHCl_3$)

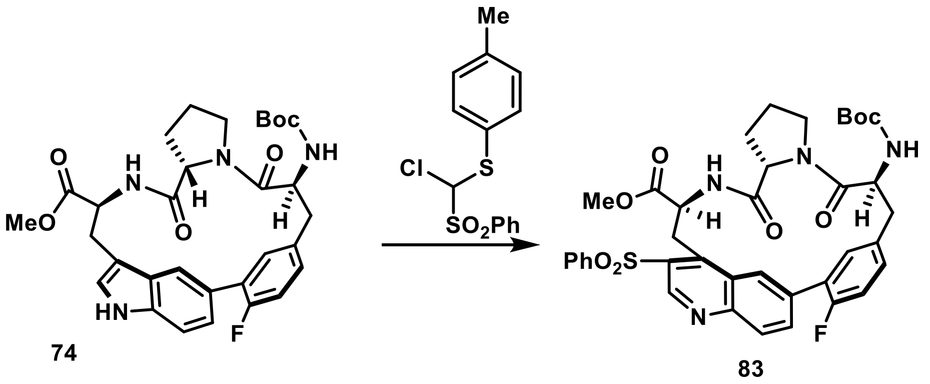

To a solution of compound **74** (43.8 mg, 76.3 µmol, 1.0 eq.) and Cs_2CO_3 (125 mg, 382 µmol, 5.0 eq.) in THF (2 mL, 0.05 M), (chloro(phenylsulfonyl)methyl)(p-tolyl)sulfane (47.6 mg, 152 µmol, 2.0 eq.) was added portion-wise (0.5 equiv. every 1 h) at room temperature, and the resulting mixture was stirred for next 4 h. The reaction was quenched with saturated aq. $Na_2S_2O_3$, extracted with EtOAc (5mL) three times. The organic layers were combined and washed with brine and concentrated under reduced pressure to give the crude. The crude was purified by preparative thin-layer chromatography (EtOAc:Hexane 3:1) to give the title compound **83** (12.4 mg, 22% yield)

Compound 83
Physical State: pale yellow solid
^{1}H NMR (400 MHz, $CDCl_3$): δ 9.41 (s, 1H), 8.21 (s, 2H), 8.12 (s, 1H), 7.84 (d, *J* = 8.0 Hz, 2H), 7.64 (d, *J* = 7.6 Hz, 1H), 7.55 (t, *J* = 7.3 Hz, 2H), 7.49 (dd, *J* = 8.3, 6.4 Hz, 3H), 7.15 – 7.07 (m, 1H), 7.06 – 6.99 (m, 1H), 5.27 – 5.20 (m, 1H), 5.07 (t, *J* = 10.4 Hz, 1H), 4.86 (t, *J* = 7.8 Hz, 1H), 4.64 (t, *J* = 13.7 Hz, 1H), 4.37 – 4.29 (m, 1H), 3.77 (s, 3H), 3.75 – 3.72 (m, 1H), 3.60 – 3.49 (m, 1H), 3.24 (dd, *J* = 13.5, 7.3 Hz, 1H), 3.15 – 3.07 (m, 1H), 2.09 – 1.69 (m, 4H), 1.34 (s, 9H).
^{13}C NMR (101 MHz, $CDCl_3$) : δ 170.69, 170.47, 169.08, 159.65, 157.19, 154.64, 148.22, 147.81, 144.49, 141.32, 136.31, 133.47 (d, *J* = 7.7 Hz), 133.05, 132.67, 132.24, 132.12 (d, *J* = 8.4 Hz), 129.44, 129.01, 126.44, 125.52, 124.43, 114.68 (d, *J* = 22.7 Hz), 79.05, 59.73, 52.22, 51.73, 50.60, 46.88, 35.15, 28.64, 27.63, 24.42.
^{19}F NMR (376 MHz, CD_3OD): δ -121.04.
HRMS (ESI-TOF): calculated for $C_{38}H_{39}FN_4NaO_8S^+$ [M+Na]$^+$: 753.2370, found: 753.2371.
TLC: R_f = 0.5 (3:1 EtOAc : Hexane, $Ce_2(SO_4)_3$ in phosphomolybdic acid).
$[\alpha]^{21}_D$: +51.8 (*c* = 0.3, $CHCl_3$)

Reaction optimization and discussion

Optimization table of carboxylate-assisted C-H activation

Table S1. Conditions screening of carboxylate-assisted C-H activation

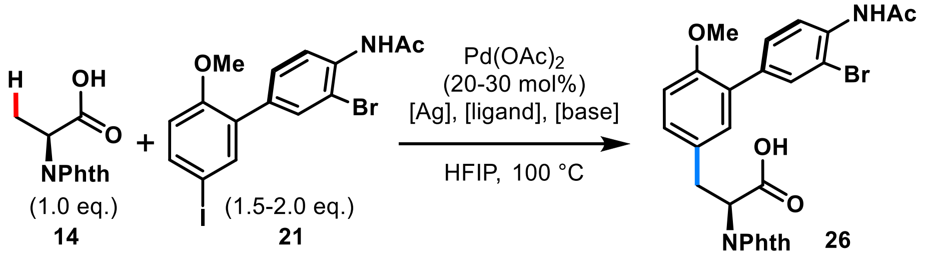

entry	[Ag] 1.0 eq.	[ligand] 0.6 eq.	[base] 0.5 eq.	yield[a]
1	Ag_2CO_3	Ac-*L*-Phg-OH	K_2CO_3	60%
2	Ag_2CO_3	Ac-*L*-Leu-OH	K_2CO_3	67%
3	Ag_2CO_3	Ac-*L*-isoLeu-OH	K_2CO_3	70%
4	Ag_2CO_3	Ac-*L*-Phe-OH	K_2CO_3	71%
5	Ag_2CO_3	Ac-*L*-Ala-OH	K_2CO_3	73%
6[b]	Ag_2CO_3	Ac-*L*-Val-OH	K_2CO_3	75%
7	Ag_2CO_3	Ac-*D*-Leu-OH	K_2CO_3	65%
8	Ag_2CO_3	Ac-*D*-Phe-OH	K_2CO_3	63%
9[b]	Ag_2CO_3	Ac-Gly-OH	K_2CO_3	89%
10[c]	Ag_2CO_3	Ac-Gly-OH	K_2CO_3	71%
11	Ag_2CO_3	Boc-Gly-OH	K_2CO_3	trace
12	$AgBF_4$	Ac-Gly-OH	K_2CO_3	15%
13	AgOAc	Ac-Gly-OH	K_2CO_3	61%
14	Ag_2CO_3	Ac-Gly-OH	Na_2CO_3	42%
15	Ag_2CO_3	Ac-Gly-OH	Rb_2CO_3	64%
16	Ag_2CO_3	Ac-Gly-OH	KOAc	61%
17	Ag_2CO_3	Ac-Gly-OH	NaOAc	38%

[a] Isolated yields. [b] 0.2 mmol scale. [c] Reaction was carried out on 1.1 g of **19** (5 mmol scale). 20 mol% $Pd(OAc)_2$, 0.4 eq. Ac-Gly-OH, 1.5 eq. **18** and 50 mL HFIP were used for this scale.

Aminoquinoline-directed C-H activation

Scheme S3. Overview of aminoquinoline-directed C-H activation

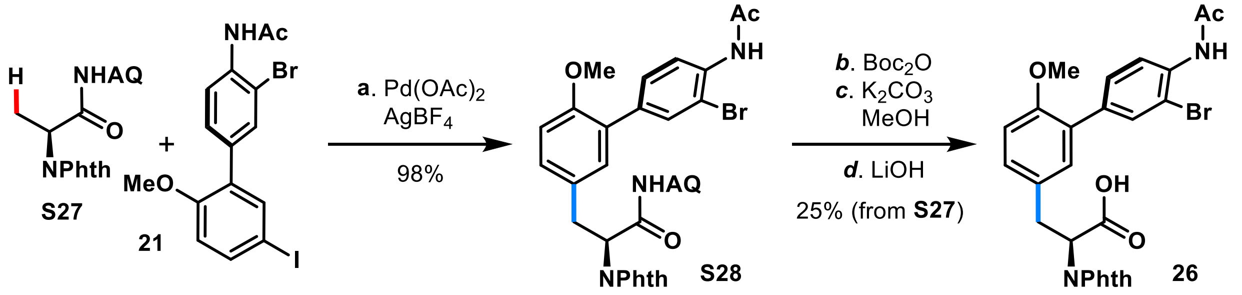

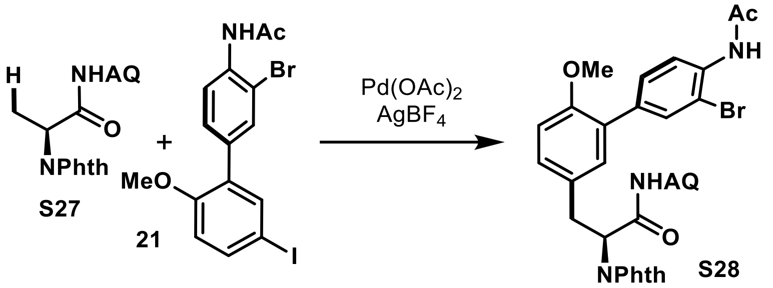

The compound **21** (104 mg, 0.3 mmol, 1.0 eq.) and compound **S27** was dissolved in *t*AmylOH (5 mL). Pd(OAc)$_2$ (8.98 mg, 0.04 mmol, 0.2 eq.), AgBF$_4$ (58.4 mg, 0.3 mmol, 1.0 eq.) were added. The reaction mixture was stirred at 80 °C under nitrogen for 24 h. The reaction mixture was concentrated under reduced pressure and purified by preparative thin-layer chromatography (EtOAc:Acetone 10:1) to give the compound **S28** (130 mg, 98% yield).

Physical State: white solid
^{1}H NMR (400 MHz, CDCl$_3$): δ 8.43 (d, *J* = 8.4 Hz, 1H), 7.69 (d, *J* = 2.1 Hz, 1H), 7.65 (t, *J* = 1.4 Hz, 2H), 7.46 (dd, *J* = 8.6, 2.1 Hz, 1H), 7.40 (ddd, *J* = 7.7, 2.2, 1.5 Hz, 1H), 7.18 (m, 1H), 2.26 (s, 3H).
^{13}C NMR (101 MHz, CDCl$_3$): δ 168.21, 167.94, 166.41, 155.3 5, 148.32, 138.48, 136.26, 134.34, 133.84, 132.71, 131.62, 131.23, 129.61, 129.39, 128.97, 128.65, 127.85, 127.29, 123.62, 122.04, 121.64, 121.13, 116.78, 111.59, 56.48, 55.56, 33.91, 24.91.
HRMS (ESI-TOF): calculated for $C_{35}H_{27}BrN_4NaO_5^+$ [M+Na]$^+$: 685.1063, found: 685.1065.
TLC: R$_f$ = 0.5 (10:1 EtOAc : Acetone, $Ce_2(SO_4)_3$ in phosphomolybdic acid).
[α]$^{25}_D$: +68.2 (*c* = 1.0, CHCl$_3$)

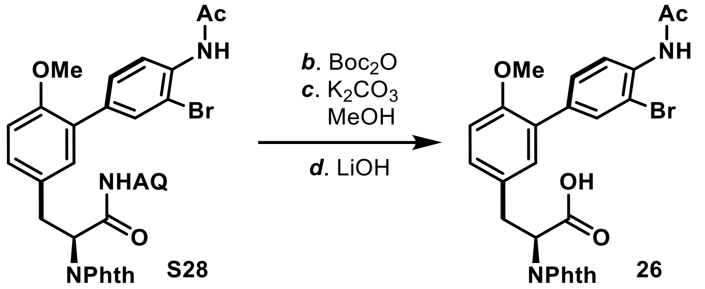

The compound **S28** (3.32 g, 5.00 mmol, 1.0 eq.) was dissolved in MeCN (60 mL, 0.083 M). DMAP (611 mg, 5.0 mmol, 1.0 eq.), DIPEA (5.0 mL, 29 mmol, 5.7 eq.), and Boc$_2$O (25 mL, 110 mmol, 22 eq.) were added sequentially. The reaction mixture was stirred at 70 °C for 12h and quenched with saturated NH$_4$Cl *aq.* , extracted with DCM (100 mL) three times. The organic layers were dried over Na$_2$SO$_4$ and concentrated under reduced pressure to give the crude. The crude was dissolved in MeOH (50 mL, 0.1 M). To this solution, K$_2$CO$_3$ (7.20 g, 36.2 mmol, 5.0 eq.) was added, and the reaction was stirred at 25 °C for 4 h. The reaction was quenched with 1N HCl *aq.* and extracted with EtOAc (100 mL) three times. The organic layers were combined, washed with brine, dried over Na$_2$SO$_4$, and concentrated under reduced pressure to give the crude. The crude was dissolved in THF/MeOH/H$_2$O = 1:1:1 (60 mL, 0.083 M), then LiOH·H$_2$O (1.26 g, 30.0 mmol, 6.0 eq.) was added. The reaction mixture was stirred at 25 °C for 1 h. The reaction mixture was acidified with 0.5M HCl *aq.* and extracted with DCM/MeOH = 10:1 three times. The organic layers were combined and washed with brine. The organic layers were concentrated under reduced pressure to give the crude. The crude was purified by silica gel column chromatography (EtOAc:Hexane 9:1 to 2:1) to give the title compound **26** (619 mg, 25% yield). The characteristic data were identical to the compound synthesized using carboxylate-assisted C-H activation.

Compound 26
Physical State: off-white solid
^{1}H NMR (600 MHz, DMSO-d_6): δ 9.46 (s, 1H), 7.81 (s, 4H), 7.39 (m, 2H), 6.94 (m, 4H), 5.06 (s, 1H), 3.62 (s, 3H), 3.28 (m, 3H), 2.05 (s, 3H).
^{13}C NMR (151 MHz, DMSO-d_6): δ 170.64, 169.15, 167.80, 155.14, 137.01, 135.52, 133.09, 131.34, 131.12, 130.24, 130.18, 129.07, 127.82, 127.00, 123.91, 117.85, 112.18, 79.70, 55.99, 53.87, 33.60, 23.76.
HRMS (ESI-TOF): calculated for $C_{26}H_{21}BrN_2NaO_6^+$ [M+Na]$^+$: 559.0475, found: 559.0484.
TLC: R_f = 0.2 (10:1 DCM:Methanol, $Ce_2(SO_4)_3$ in phosphomolybdic acid).
[α]$^{25}_D$: -68.6 (*c* = 0.1, $CHCl_3$)

Optimization table of Larock cyclization

Table S2. Conditions screening of Larock cyclization

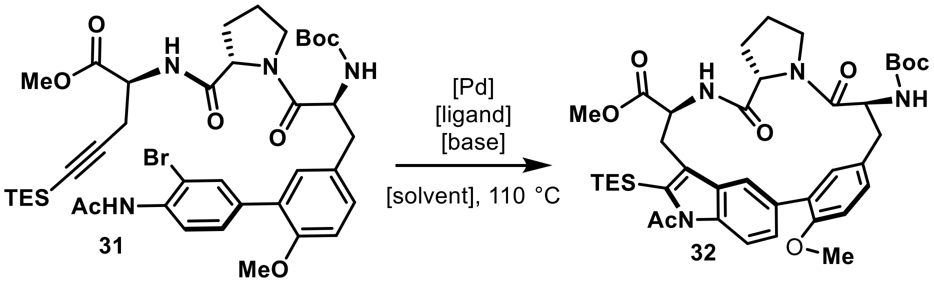

entry	[Pd] 0.5 eq.	[ligand] 1.0 eq.	[base] 10 eq.	[solvent] 1 mL	yield[a]
1	$Pd(OAc)_2$	PPh_3	DIPEA	PhMe	22%
2	$Pd(OAc)_2$	PCy_3	DIPEA	PhMe	51%
3	$Pd(OAc)_2$	$P(OMe)_3$	DIPEA	PhMe	0%
4	$Pd(OAc)_2$	dppe (0.5)	DIPEA	PhMe	31%
5	$Pd(OAc)_2$	dppf (0.5)	DIPEA	PhMe	29%
6	Pd(*t*$Bu_3P)_2$	—	DIPEA	PhMe	35%
7	$Pd(OAc)_2$	*t*$Bu_3P\cdot HBF_4$	DIPEA	PhMe	59%
8[b]	$Pd(OAc)_2$	*t*$Bu_3P\cdot HBF_4$	DIPEA	PhMe	45%
9	$Pd(OAc)_2$	*t*$Bu_3P\cdot HBF_4$	Cy_2NMe	PhMe	50%
10	$Pd(OAc)_2$	*t*$Bu_3P\cdot HBF_4$	DBU	PhMe	30%
11	$Pd(OAc)_2$	*t*$Bu_3P\cdot HBF_4$	K_2CO_3	PhMe	42%
12	$Pd(OAc)_2$	*t*$Bu_3P\cdot HBF_4$	Cs_2CO_3	PhMe	trace
13	$Pd(OAc)_2$	*t*$Bu_3P\cdot HBF_4$	NaOAc	PhMe	36%

[a] Isolated yields. [b] The reaction was performed on 18.2 g scale. PhMe (0.06 M) were used for this scale.

Assessment of the thermodynamic stability of protected *R*-conf scabrirubin CB-4 (55)

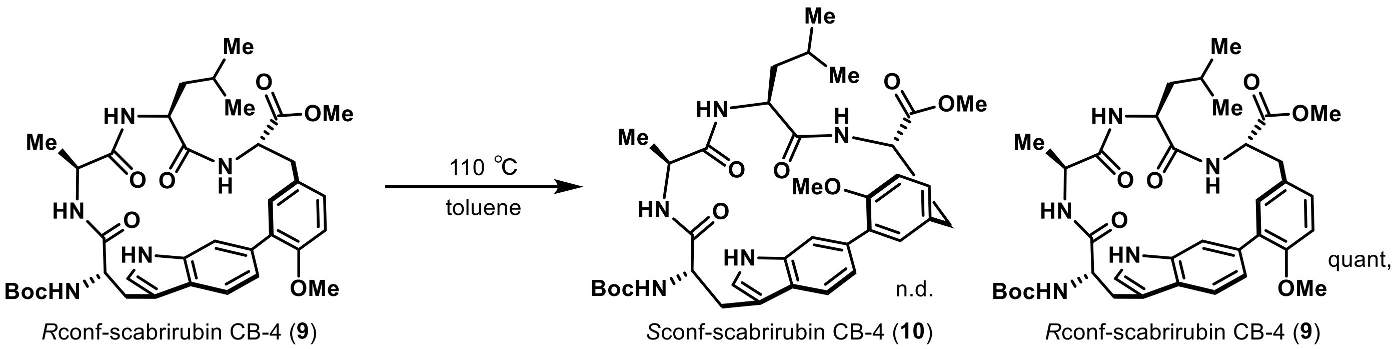

The compound **9** (10.3 mg, 15.2 μmol, 1.0 eq.) was dissolved in toluene (2 mL, 7.6 mM) and the solution was heated at 110 °C for 12h. The solvent was removed under reduced pressure, and the crude was purified by preparative thin-layer chromatography (EtOAc:Hexane 2:1) to recover the starting material **9** (10.2 mg, quant.). The conformational isomer **10** was not observed.

Skeletal editing of scabrirubin core: stepwise procedure

Scheme S4. Overview of aminoquinoline-directed C-H activation.

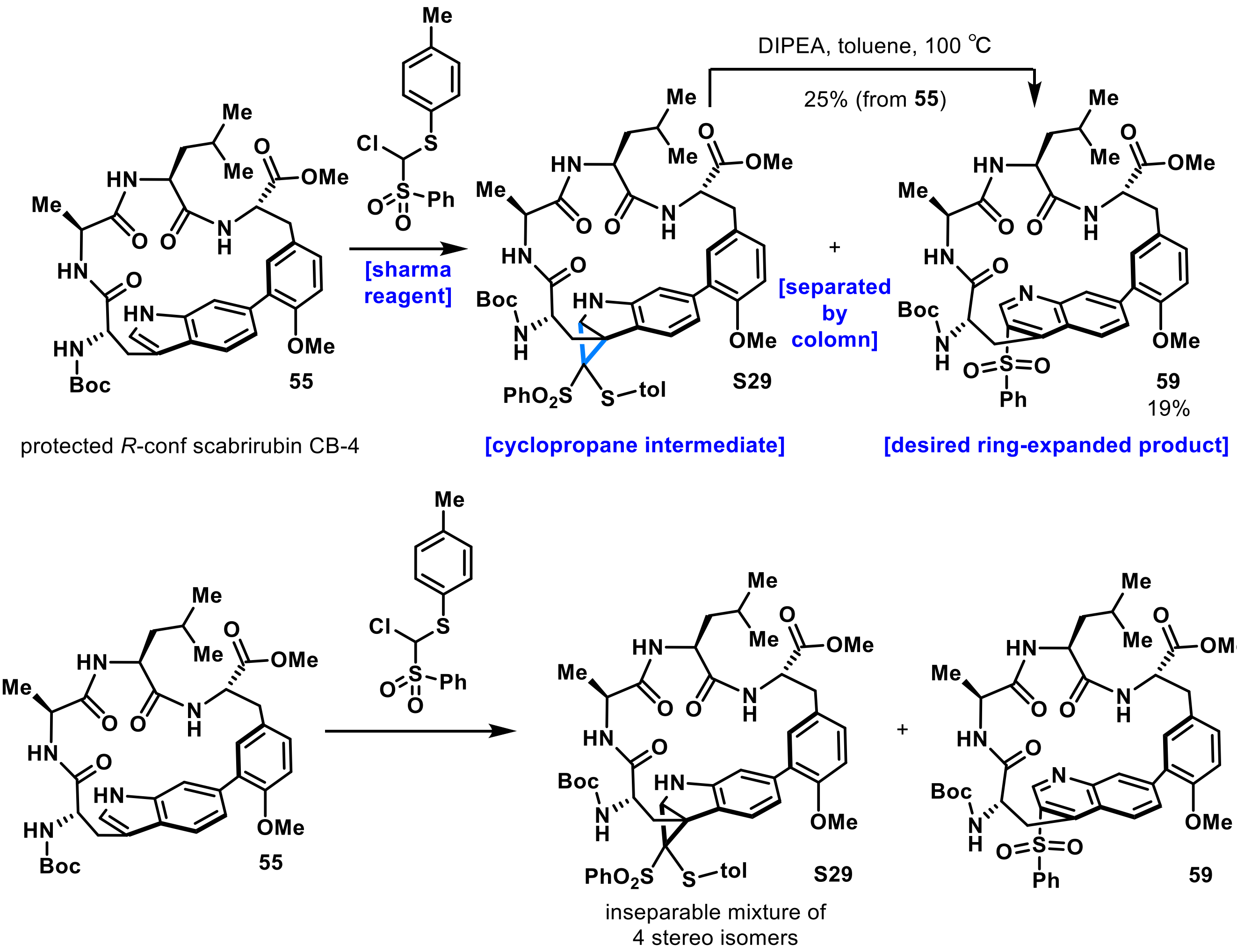

To a solution of compound **55** (100 mg, 148 μmol, 1.0 eq.) and Cs_2CO_3 (241 mg, 738 μmol, 5.0 eq.) in THF (1.5 mL, 0.1 M), (chloro(phenylsulfonyl)methyl)(p-tolyl)sulfane (92.1 mg, 295 μmol, 2.0 eq.) was added portion-wise (0.5 equiv. every 1 h) at room temperature, and the resulting mixture was stirred for the next 4 h. The reaction was quenched with saturated aq. $Na_2S_2O_3$, extracted with EtOAc (5mL) three times. The

organic layers were combined and washed with brine and concentrated under reduced pressure to give the crude. The crude was purified by preparative thin-layer chromatography (EtOAc:Hexane 3:1) to give the compound **59** (23.3 mg, 19% yield), along with the fraction containing cyclopropane intermediate **S28** (67.7 mg), which was obtained as an inseparable mixture of 4 stereoisomers.

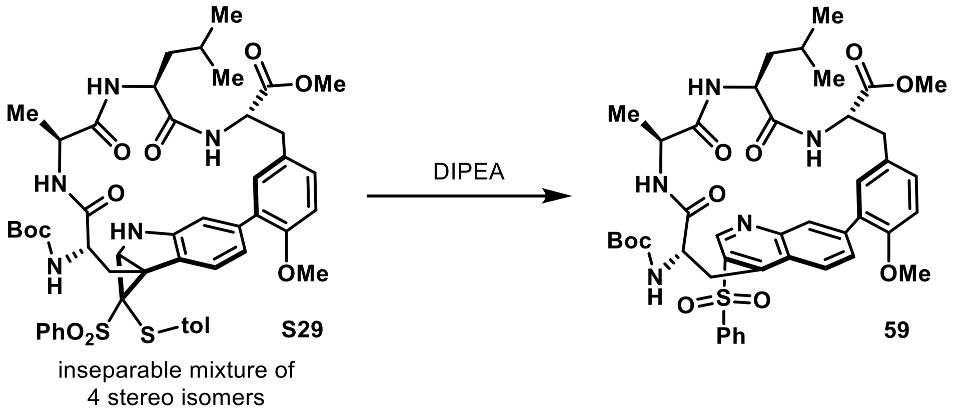

The obtained fraction containing cyclopropane intermediate **S29** (67.7 mg) was dissolved in toluene (2 mL), and DIPEA (0.2 mL) was added. The solution was heated at 100 °C until the compound **S29** is completely consumed, judged by TLC. The solvent was evaporated, and the crude was purified by preparative thin-layer chromatography (EtOAc:Hexane 3:1) to give the compound **59** (30.6 mg, 25% yield from compound **55**). The characterization data were identical to those of compound **59** originally obtained in the previous step.

LC-MS spectrum of cyclopropane intermediate S29

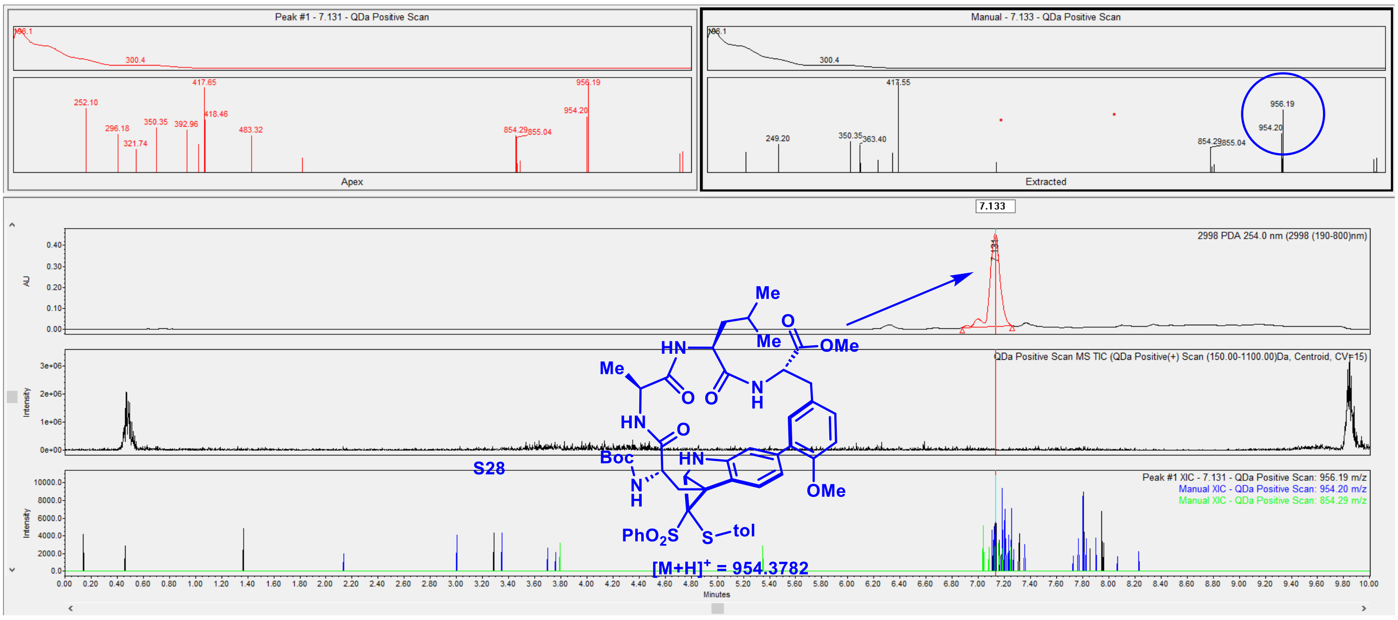

Figure S1. LC-MS spectrum of cyclopropane intermediate **S29**.

Computational Details

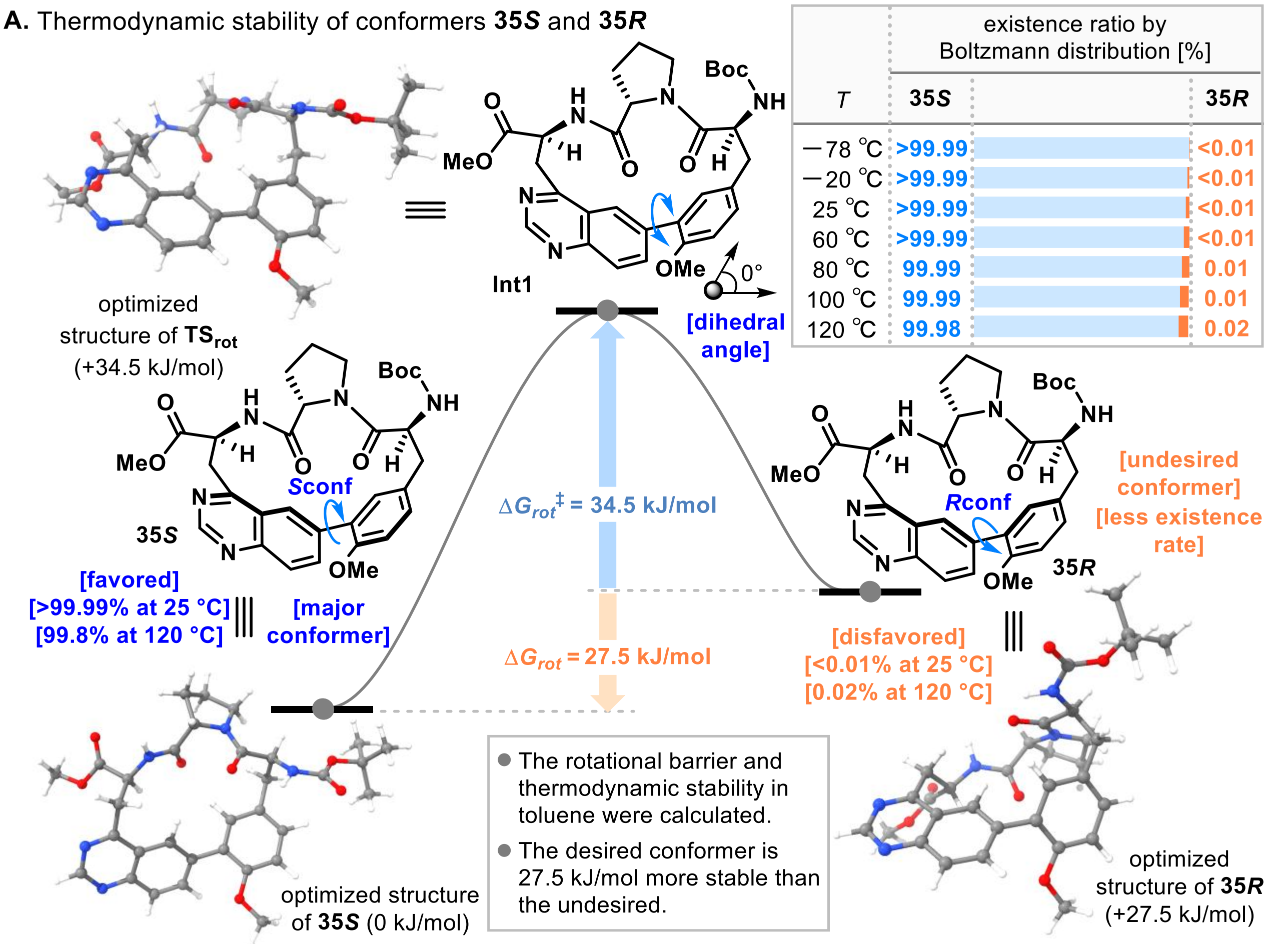

T	existence ratio by Boltzmann distribution [%] 35*S*	35*R*
−78 °C	>99.99	<0.01
−20 °C	>99.99	<0.01
25 °C	>99.99	<0.01
60 °C	>99.99	<0.01
80 °C	99.99	0.01
100 °C	99.99	0.01
120 °C	99.98	0.02

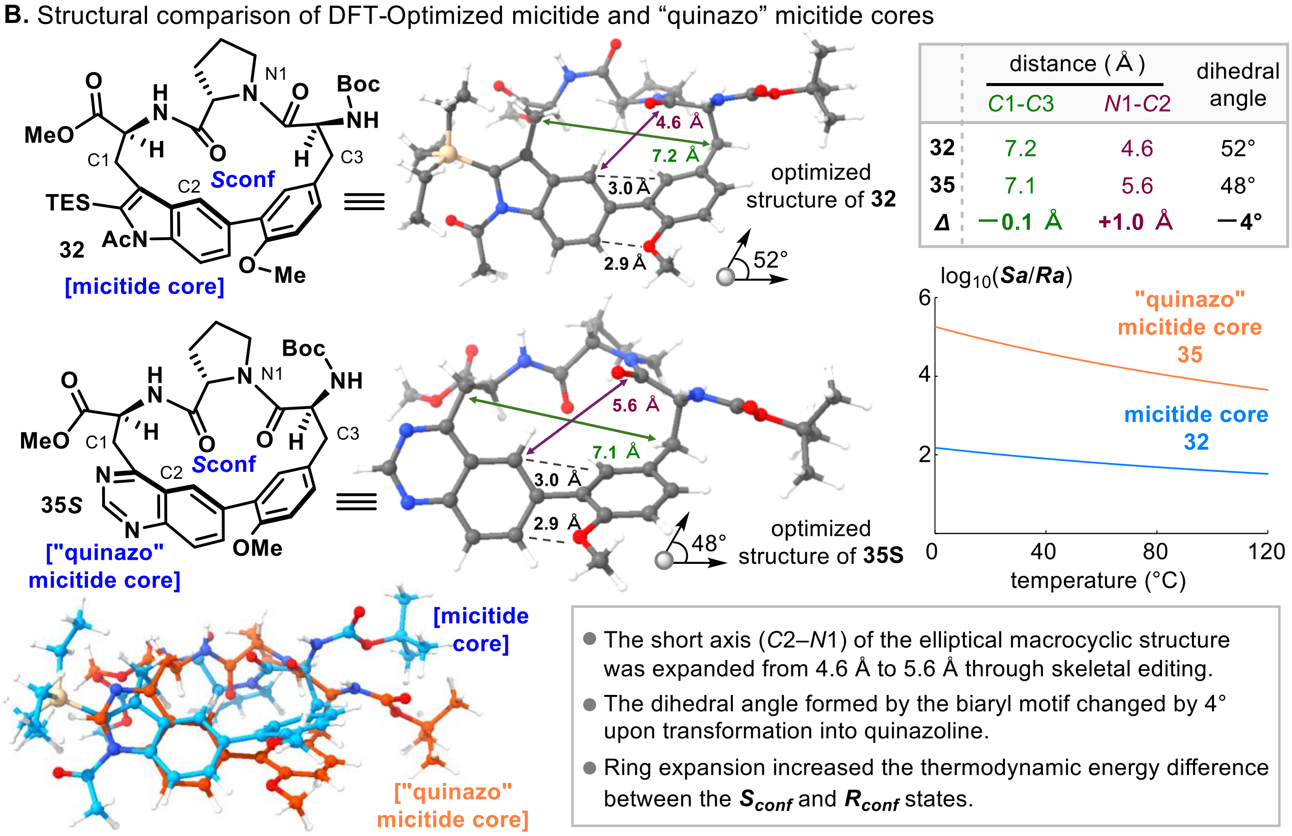

	distance (Å) C1-C3	distance (Å) N1-C2	dihedral angle
**32**	7.2	4.6	52°
**35**	7.1	5.6	48°
Δ	**−0.1 Å**	**+1.0 Å**	**−4°**

Figure S2. (A) Thermodynamic stability of conformers **35*S*** and **35*R***. (B) Structural comparison of DFT-optimized structures.

Equilibrium structures and a transition state were optimized employing GRRM23[3–5] with Gaussian 16, Revision C.02,[6] at the ωB97X-D/def2-SVP level of theory in conjunction with the SMD solvation model (methanol) at 298.15 K. Subsequent vibrational frequency analyses were performed at the ωB97X-D/def2-TZVP level with the SMD solvation model (methanol) to evaluate the Gibbs free energy at 298.15 K. The visualization was conducted by UCSF ChimeraX version 1.9.[7,8]

Table S3. Summary of the DFT calculations

	Free energy (hartree)	Relative free energy (hartree)	Relative free energy (kJ/mol)
Compound-**35S**	-2039.494037	0	0
Compound-**Int1**	-2039.479568	0.014469125	37.98868677
Compound-**35R**	-2039.482872	0.011164835	29.31327395

Cartesian Coordinates

Compound-35S

C	-1.912412849580	3.486379636046	-2.152935426064
C	0.050131378709	1.995278807415	-1.597368903561
C	-3.258740158463	3.639034269604	-2.494722533042
C	-1.382449007802	2.191248306237	-1.950856866661
C	-2.239930529180	1.093337015403	-2.063685178320
C	-4.081809761049	2.520342903298	-2.613226360109
C	-3.594467173711	1.231368634620	-2.389232273292
H	-1.833196540169	0.092047374363	-1.896708480660
H	-3.680451898827	4.629862177305	-2.663397819146
H	-5.129309913727	2.666598525923	-2.890974277495
C	-4.517343937542	0.041767370306	-2.449987093445
C	0.410952554853	1.152677442188	-0.564274559440
C	1.077329547300	2.656167440402	-2.334618955398
C	2.401101801450	2.498673676461	-2.016023259441
C	1.777000662860	0.959603824056	-0.222750111446
C	2.782308824736	1.657854948368	-0.938865631535
H	0.798631048602	3.301397803225	-3.169129693944
H	3.185052250607	3.012611913078	-2.575704092590
H	-0.373141199036	0.651071502472	0.007528457566
H	-3.963118917151	-0.858991593248	-2.753336156777
C	-5.241474129377	-0.252298843833	-1.103187861263
H	-5.297995390293	0.214668702941	-3.204354468068
C	-4.248408899378	-0.825284684456	-0.101558033160
N	-5.824165188558	0.922801679123	-0.498302774561
H	-6.038119056991	-0.981348865821	-1.297657128410
O	-3.611134042786	-0.076724737800	0.643855274121
N	-4.062237370210	-2.154251695518	-0.096916161519
C	-4.722269309538	-3.159377971590	-0.939889582772
C	-3.010622183193	-2.720525514109	0.731687533655

C	-3.820305177003	-4.384208429296	-0.800554402327
C	-3.224195819915	-4.236646661177	0.598867820554
H	-4.800840958489	-2.824246215725	-1.982288478390
H	-5.738488486125	-3.358113980939	-0.561847600162
C	-1.647111011228	-2.304083717195	0.181619208495
H	-2.290432313228	-4.797504786797	0.743991767081
H	-3.022637681189	-4.347003762987	-1.557170884248
H	-4.375581122563	-5.322020289616	-0.934444916409
H	-3.941901744036	-4.563462004428	1.366188974019
O	-1.472842054863	-2.094775325692	-1.015384308131
N	-0.664045751098	-2.225307461707	1.096040130214
H	-0.870362594299	-2.438950508616	2.067193318906
C	0.692481704847	-1.924720152242	0.734359447477
H	0.670747152487	-1.628048947958	-0.325071110825
C	1.276928339245	-0.771286087789	1.587291368259
C	1.553665904619	-3.177864215288	0.796614384690
O	2.784774790085	-2.927868030401	0.387406443606
O	1.166007538023	-4.262560629783	1.161060065081
C	3.721000837399	-4.003771598059	0.410584137135
H	0.430362151838	-0.160656427911	1.931346542893
H	1.785969765679	-1.164067226127	2.477438533224
C	2.238138721396	0.097390276810	0.823757304725
N	3.515147046332	0.028128432791	1.130274692512
H	-5.226823711267	1.439381753114	0.141419754404
C	-6.900764753731	1.594833238356	-0.968304682982
O	-1.067257498771	4.526096672770	-1.980868196666
C	-1.540908512762	5.842975812997	-2.160952322965
O	-7.243038819997	2.687705081516	-0.543536794602
O	-7.526923286744	0.902115480297	-1.920103925585
C	-8.687186810104	1.407248744913	-2.647720414072
C	-9.848934107237	1.647042231646	-1.688515871041
C	-8.300683047240	2.660800746881	-3.426221553596
C	-9.011034139850	0.263245240935	-3.601009009462
H	-8.158392505130	0.056323679609	-4.265162684848
H	-9.249065582207	-0.653373818848	-3.040503067023
H	-9.879093031231	0.525740936344	-4.222687627013
H	-10.035030185422	0.747346889655	-1.082217196919
H	-9.656095225473	2.492277356302	-1.016401732054
H	-10.758962011726	1.863945252845	-2.267381610384
H	-8.037082091392	3.489285841939	-2.756220400905
H	-7.446767842730	2.453059158671	-4.089109379532
H	-9.148574222665	2.977872602857	-4.051340370445
H	-1.896950396243	6.008272652204	-3.191155051960
H	-2.350466212232	6.082180823914	-1.451828128146
H	-0.688673920787	6.506027593662	-1.966961800330

H	3.834947730114	-4.392178086147	1.432417880301
H	3.394195538003	-4.812546253548	-0.258168771116
H	4.670416877454	-3.585158942079	0.059689291555
N	4.098709197236	1.540180451290	-0.610558698877
C	4.384343611441	0.763640328105	0.399589939083
H	5.437796970247	0.680622794844	0.689947776671
H	-3.104037860047	-2.376759561313	1.771981626921

Compound-Int1

C	-1.385256069664	3.203992374812	-1.195760218447
C	0.897046835171	2.608434592496	-0.121701786697
C	-2.643916209463	2.779693653908	-1.639458587349
C	-0.471285659285	2.266176286142	-0.639658764457
C	-0.895643553224	0.928476407397	-0.606528860589
C	-3.012437672075	1.439973656631	-1.580216303632
C	-2.131449829545	0.484353164440	-1.077787693109
H	-0.233088234341	0.173501220778	-0.191568696464
H	-3.348654768779	3.501737319817	-2.050518049935
H	-3.996189684031	1.138953589112	-1.949682816627
C	-2.492249783048	-0.980657839993	-1.090316554916
C	1.765221225489	1.598286250080	0.267519414325
C	1.376117120017	3.950367750650	0.016905440395
C	2.642897095562	4.235646866387	0.465172235496
C	3.061958704752	1.865263047095	0.766078120038
C	3.528836990884	3.199521399308	0.842285603734
H	0.721925318356	4.776341944141	-0.242764982113
H	2.984717999113	5.269484866991	0.546797213803
H	1.468772947273	0.555343926739	0.189848642216
H	-1.782942045078	-1.509302599174	-1.745082678741
C	-2.510907386692	-1.692439046881	0.299219608112
H	-3.484738467491	-1.097174037951	-1.544234149925
C	-1.133462912633	-2.267620007889	0.625465331272
N	-2.876121849492	-0.835249243539	1.399655258322
H	-3.227819061306	-2.524307439686	0.234007859342
O	-0.389204549837	-1.702441216988	1.431349621744
N	-0.738731119754	-3.364919556065	-0.039923230846
C	-1.500570214292	-4.176969291533	-0.995801937594
C	0.658570665129	-3.774444844621	0.044425353481
C	-0.408874631398	-4.795025659341	-1.861014313455
C	0.737423526348	-5.007191676785	-0.873342261828
H	-2.206877191044	-3.575196406874	-1.578961400903
H	-2.072740650811	-4.949219754481	-0.455098674307
C	1.545027439743	-2.622496057133	-0.439777245450
H	1.721513210200	-5.102446800087	-1.352239819540
H	-0.110370639110	-4.083367463457	-2.645949017016

H	-0.737526808838	-5.724512235232	-2.344677419248
H	0.565186767484	-5.911872621180	-0.271534342101
O	1.163121804532	-1.830110526596	-1.294175406595
N	2.764986053706	-2.560387890766	0.131838931237
H	2.986234270692	-3.210983021380	0.880271444999
C	3.585286714273	-1.381437105739	0.020392278734
H	3.185800947625	-0.777671936035	-0.804851191407
C	3.527294380727	-0.580693915189	1.346927174411
C	5.021693341200	-1.739314427502	-0.304370174449
O	5.654650656782	-0.714137684783	-0.848630731692
O	5.537050984411	-2.802751289867	-0.051867009080
C	7.057767120878	-0.848957394957	-1.069707362325
H	2.486981206739	-0.633722286977	1.696873139611
H	4.156496643664	-1.081271167482	2.096830265718
C	3.955119185587	0.857150034459	1.252594233973
N	5.159453061774	1.171117815405	1.677665482170
H	-2.114968871516	-0.526705114698	1.998417925971
C	-4.063604705457	-0.212712539617	1.565478250327
O	-1.007444304697	4.495441026221	-1.295241232881
C	-1.879539264972	5.453358828590	-1.854602237838
O	-4.283292632419	0.575831585744	2.472865452998
O	-4.941089752748	-0.585229781543	0.632832172914
C	-6.321092240714	-0.111832345867	0.595976633072
C	-7.061876420644	-0.597458613839	1.837744318852
C	-6.363120876366	1.406302878744	0.448536353045
C	-6.872311746092	-0.782589343139	-0.656591685520
H	-6.323265027876	-0.448804136071	-1.550111134854
H	-6.786081145707	-1.876993412847	-0.580779087743
H	-7.933448350928	-0.525405126091	-0.786167157156
H	-6.969545712374	-1.689932030558	1.935981762251
H	-6.673420135884	-0.126662379647	2.750107347393
H	-8.129871344558	-0.349361762026	1.748128362731
H	-5.998068757692	1.914651891236	1.349314571573
H	-5.752917641818	1.726623466140	-0.409105528996
H	-7.401243288581	1.722216949885	0.267076445309
H	-2.130467969042	5.211240753566	-2.900250713022
H	-2.807149827139	5.550203364558	-1.267190698935
H	-1.339336249295	6.407875384913	-1.828615972870
H	7.578345531474	-1.026768401352	-0.117996766345
H	7.261234766570	-1.675917412056	-1.764285045363
H	7.387607573166	0.099607646723	-1.506869312078
N	4.786416805129	3.488506809064	1.279276532352
C	5.523709330940	2.475635081456	1.647412148407
H	6.543579906176	2.690356379534	1.985959792451
H	0.932294126142	-4.019869367620	1.080951531581

Compound 35R

C	-1.066272195002	1.169243169041	-4.003086728361
C	-0.233601932518	1.908915542123	-1.746418495967
C	-2.084960214398	0.630995947546	-4.792864591096
C	-1.338317324488	1.525262377830	-2.664734022891
C	-2.630979277835	1.349507066389	-2.168864088995
C	-3.358685457350	0.441318282122	-4.256792378123
C	-3.659932671138	0.799854923200	-2.942174349881
H	-2.825682667375	1.639502413574	-1.133703582445
H	-1.892327354798	0.333150916534	-5.823738065950
H	-4.135434137569	0.001356908334	-4.888664306342
C	-5.056923262245	0.605373746514	-2.403402083315
C	-0.078033653755	1.174895616673	-0.590324505280
C	0.695388886706	2.951206008952	-2.027090743394
C	1.735144008829	3.225315419911	-1.171401424876
C	1.020203037452	1.392652964514	0.273393768416
C	1.931267013847	2.439873571606	-0.004622865836
H	0.570224727861	3.545811968944	-2.934134610618
H	2.436854253558	4.036627065115	-1.375175020554
H	-0.798421450813	0.389222812461	-0.370031593528
H	-5.625116444800	-0.013988785474	-3.110470879726
C	-5.204204381144	0.009921070227	-0.974652149989
H	-5.582444662526	1.572103057138	-2.382907939160
C	-4.049238429161	-0.886075127998	-0.499223444115
N	-5.350011289177	1.030230189713	0.040939497910
H	-6.108521736448	-0.619554801508	-0.979170082819
O	-3.352060002520	-0.543416823505	0.460009216582
N	-3.841769305326	-2.059211235383	-1.113265430247
C	-4.537022123469	-2.618057224865	-2.274523205100
C	-2.823378686691	-2.966051496237	-0.582399622530
C	-3.555865175187	-3.671382906024	-2.773918841855
C	-2.936455351820	-4.207962892405	-1.484288483213
H	-4.738386390929	-1.850307302140	-3.028692787633
H	-5.496355154040	-3.067310831258	-1.966276659046
C	-1.440438772881	-2.320555791026	-0.663187056701
H	-1.965183380255	-4.699004867030	-1.634406516095
H	-2.786030513036	-3.188606321003	-3.394687566798
H	-4.048352616822	-4.449837044677	-3.371743875256
H	-3.611332535850	-4.934410956034	-1.007947915295
O	-1.055356155736	-1.761243582626	-1.684074048442
N	-0.690160856840	-2.446746775947	0.451436130815
H	-1.106763642410	-2.885104749345	1.266839682728
C	0.567938046912	-1.772393868188	0.628369780295
H	0.837188512031	-1.343445738797	-0.347719904583
C	0.471279325669	-0.644595738398	1.698841897206

C	1.667534218074	-2.757594292617	0.983151170881
O	2.847216724402	-2.159531378625	0.987372181336
O	1.496863044960	-3.924780860852	1.242051496395
C	3.981318335922	-2.941116037196	1.358584292176
H	-0.584042451786	-0.338820997973	1.745621518531
H	0.744034294987	-1.029572834797	2.690737181397
C	1.318432051633	0.563294598632	1.399121324551
N	2.345699884552	0.829494077972	2.175279575900
H	-4.584121705907	1.124042425576	0.702470291461
C	-6.451701628437	1.778445018363	0.262134582451
O	0.204092580691	1.339592561304	-4.429573914570
C	0.549626136908	0.963072295139	-5.744589792277
O	-6.523971927649	2.615709613470	1.150083140698
O	-7.423093534441	1.480261618250	-0.602808356850
C	-8.702163634465	2.181370154831	-0.646075531701
C	-9.465190616769	1.957304817912	0.655581795352
C	-8.476371351261	3.660110857040	-0.944768658866
C	-9.416618266914	1.503962777902	-1.809440198808
H	-8.845021070809	1.628870524397	-2.741550268007
H	-9.539093910931	0.427608137941	-1.615720673097
H	-10.412799805044	1.947442897342	-1.950236643071
H	-9.545113117749	0.880848683787	0.871342371701
H	-8.975252579030	2.452150803594	1.503591533462
H	-10.482729460224	2.363697285804	0.556567515288
H	-7.969945171837	4.169502907522	-0.115141430620
H	-7.870583190960	3.778758611915	-1.856249376339
H	-9.446992867013	4.150087879717	-1.112193638886
H	0.388298020998	-0.114769774268	-5.911827293434
H	-0.019384354065	1.538153045651	-6.493676663231
H	1.617617997333	1.186427221924	-5.858960234267
H	3.856287647627	-3.341896479512	2.374392849406
H	4.126892009418	-3.768412314990	0.649707799779
H	4.840038647256	-2.261876158062	1.325882618311
N	2.984494061630	2.687399510918	0.823719508408
C	3.116607684514	1.897979964306	1.856409653628
H	3.948464630268	2.100550303192	2.540416690186
H	-3.049630932430	-3.204928644101	0.467496474856

Reference

(1) He, B.-B.; Liu, J.; Cheng, Z.; Liu, R.; Zhong, Z.; Gao, Y.; Liu, H.; Song, Z.-M.; Tian, Y.; Li, Y.-X. Bacterial Cytochrome P450 Catalyzed Post-Translational Macrocyclization of Ribosomal Peptides. *Angew. Chem. Int. Ed.* **2023**, *62* (46), e202311533.

(2) Ogawa, H.; Nagata, Y.; Chan, T. K.; Matsuda, Y.; Nakamura, H. Rapid Construction of a Tyr C6–Trp C5′ Linkage: Application in the Total Synthesis of Micitide 982, a Noncanonical Cyclic Peptide. *Angew. Chem. Int. Ed.* **2025**, *64*, e202516053.

(3) Mita, T.; Takano, H.; Hayashi, H.; Kanna, W.; Harabuchi, Y.; Houk, K. N.; Maeda, S. Prediction of High-Yielding Single-Step or Cascade Pericyclic Reactions for the Synthesis of Complex Synthetic Targets. *J. Am. Chem. Soc.* **2022**, *144* (50), 22985–23000.

(4) Sumiya, Y.; Harabuchi, Y.; Nagata, Y.; Maeda, S. Quantum Chemical Calculations to Trace Back Reaction Paths for the Prediction of Reactants. *JACS Au* **2022**, *2* (5), 1181–1188.

(5) Maeda, S.; Ohno, K.; Morokuma, K. Systematic Exploration of the Mechanism of Chemical Reactions: The Global Reaction Route Mapping (GRRM) Strategy Using the ADDF and AFIR Methods. *Phys. Chem. Chem. Phys.* **2013**, *15* (11), 3683–3701.

(6) M. J. Frisch, G. W. Trucks, H. B. Schlegel, G. E. Scuseria, M. A. Robb, J. R. Cheeseman, G. Scalmani, V. Barone, G. A. Petersson, H. Nakatsuji, X. Li, M. Caricato, A. V. Marenich, J. Bloino, B. G. Janesko, R. Gomperts, B. Mennucci, H. P. Hratchian, J. V. Ortiz, A. F. Izmaylov, J. L. Sonnenberg, D. Williams-Young, F. Ding, F. Lipparini, F. Egidi, J. Goings, B. Peng, A. Petrone, T. Henderson, D. Ranasinghe, V. G. Zakrzewski, J. Gao, N. Rega, G. Zheng, W. Liang, M. Hada, M. Ehara, K. Toyota, R. Fukuda, J. Hasegawa, M. Ishida, T. Nakajima, Y. Honda, O. Kitao, H. Nakai, T. Vreven, K. Throssell, J. A. Montgomery, Jr., J. E. Peralta, F. Ogliaro, M. J. Bearpark, J. J. Heyd, E. N. Brothers, K. N. Kudin, V. N. Staroverov, T. A. Keith, R. Kobayashi, J. Normand, K. Raghavachari, A. P. Rendell, J. C. Burant, S. S. Iyengar, J. Tomasi, M. Cossi, J. M. Millam, M. Klene, C. Adamo, R. Cammi, J. W. Ochterski, R. L. Martin, K. Morokuma, G. Farkas, J. B. Foresman, and D. J. Fox, *Gaussian 16, Revision C.02*, Gaussian, Inc., Wallingford CT, 2019.

(7) Meng, E. C.; Goddard, T. D.; Pettersen, E. F.; Couch, G. S.; Pearson, Z. J.; Morris, J. H.; Ferrin, T. E. UCSF ChimeraX: Tools for Structure Building and Analysis. *Protein Sci.* **2023**, *32* (11), e4792.

(8) Pettersen, E. F.; Goddard, T. D.; Huang, C. C.; Meng, E. C.; Couch, G. S.; Croll, T. I.; Morris, J. H.; Ferrin, T. E. UCSF ChimeraX: Structure Visualization for Researchers, Educators, and Developers. *Protein Sci.* **2021**, *30* (1), 70–82.

NMR Spectrums

Compound 26 ¹H NMR (600 MHz, DMSO-d_6)

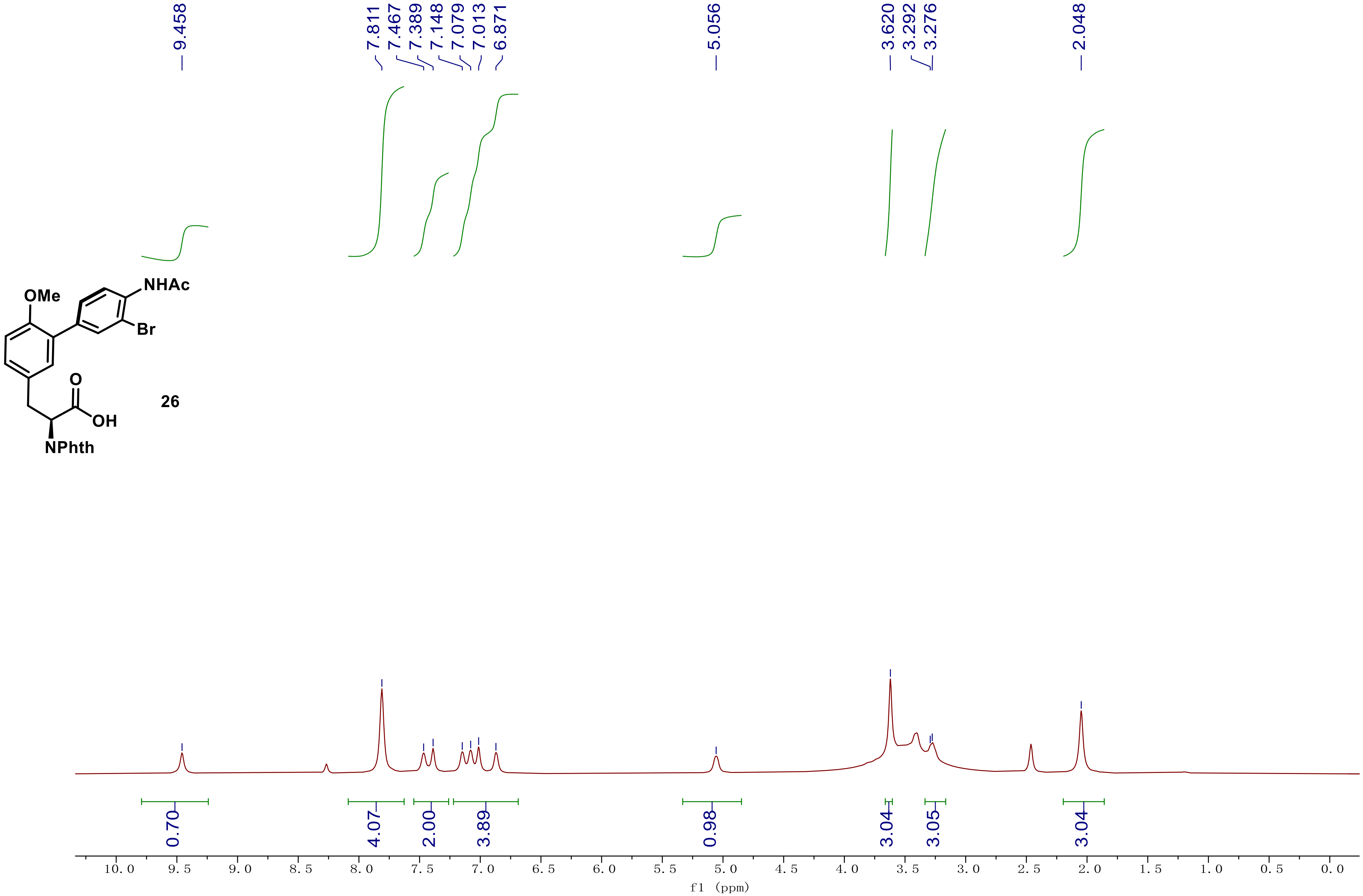

Compound 26 ^{13}C NMR (151 MHz, DMSO-d_6)

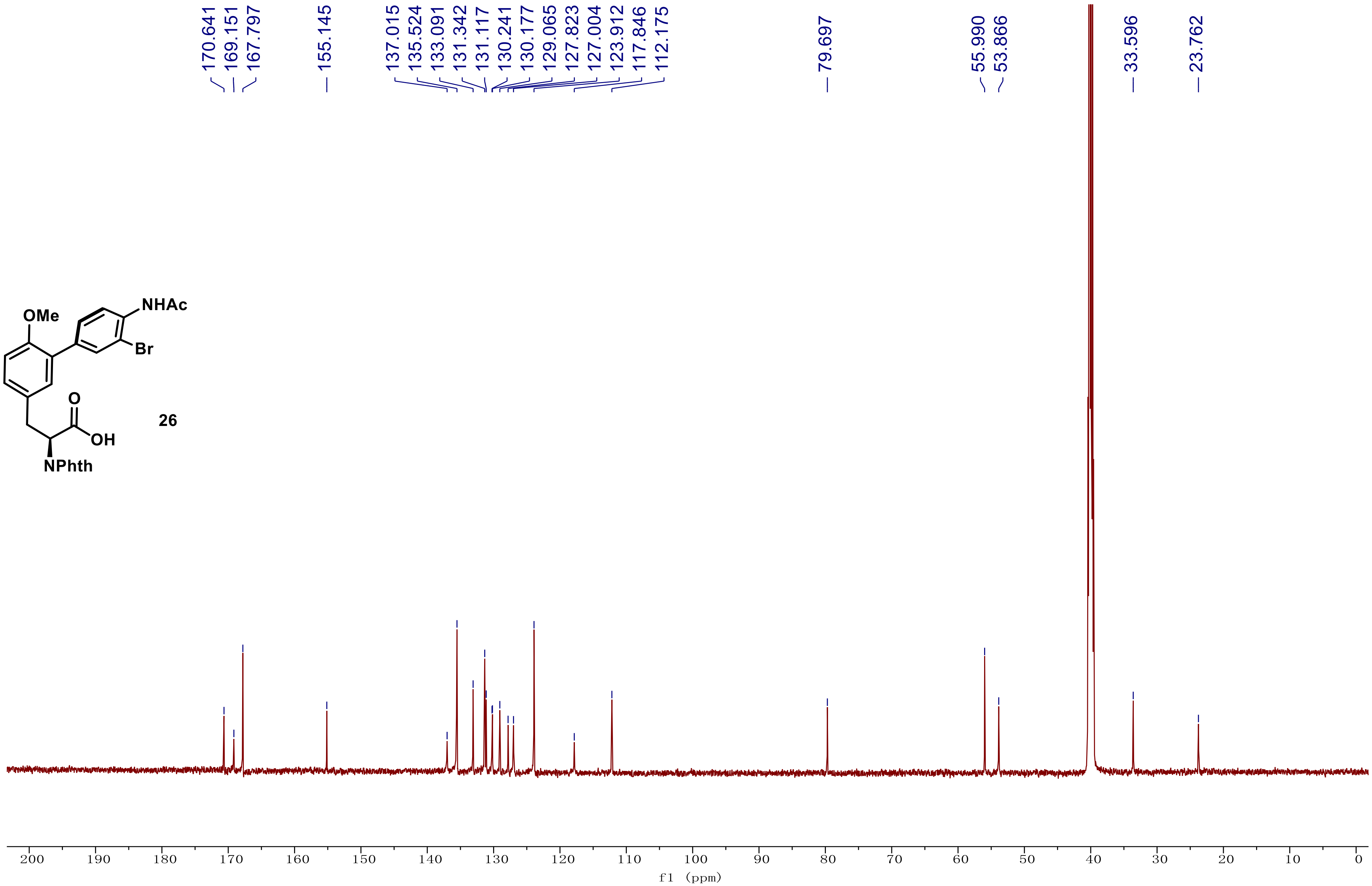

Compound 27 ¹H NMR (600 MHz, CDCl₃)

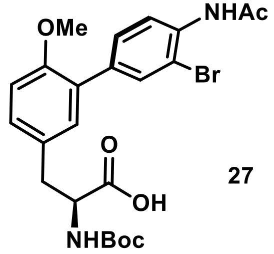

27

A mixture of two rotamers.
A:**B** = 3:1

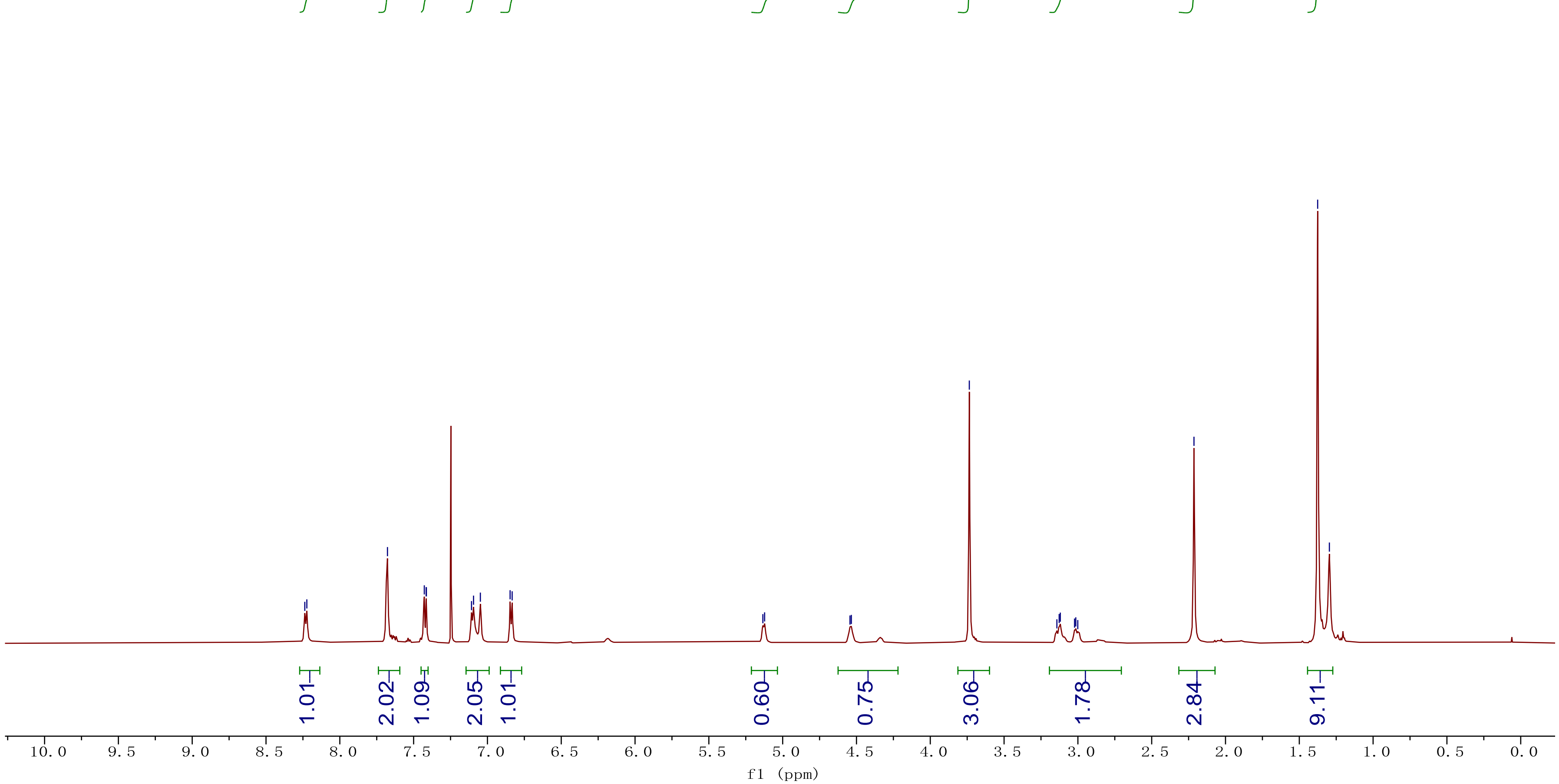

Compound 27 ^{13}C NMR (151 MHz, $CDCl_3$)

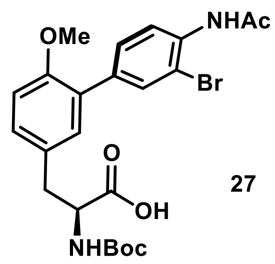

27

A mixture of two rotamers.
A:**B** = 3:1

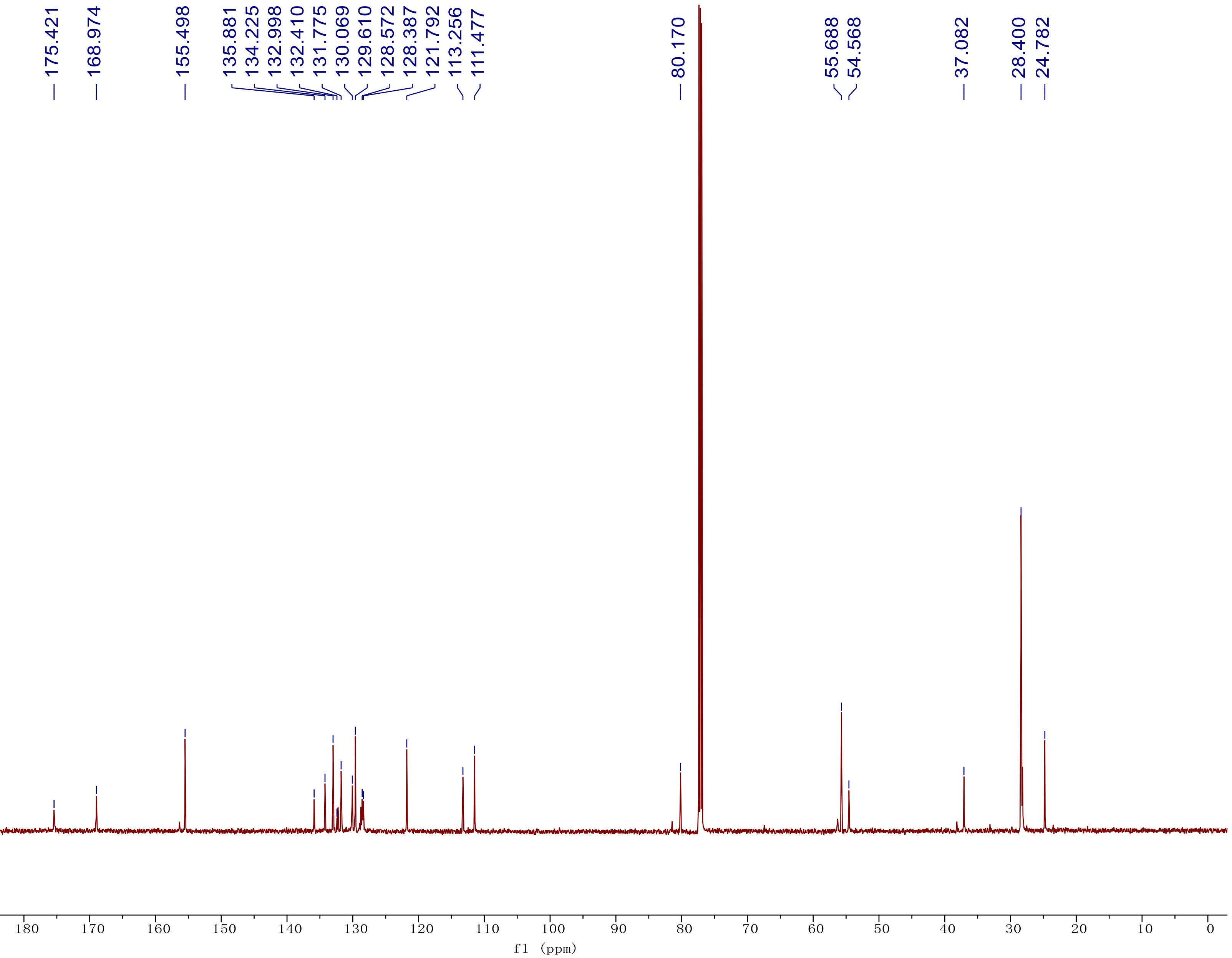

Compound 29 ^{1}H NMR (400 MHz, DMSO-*d6*, 60 °C)

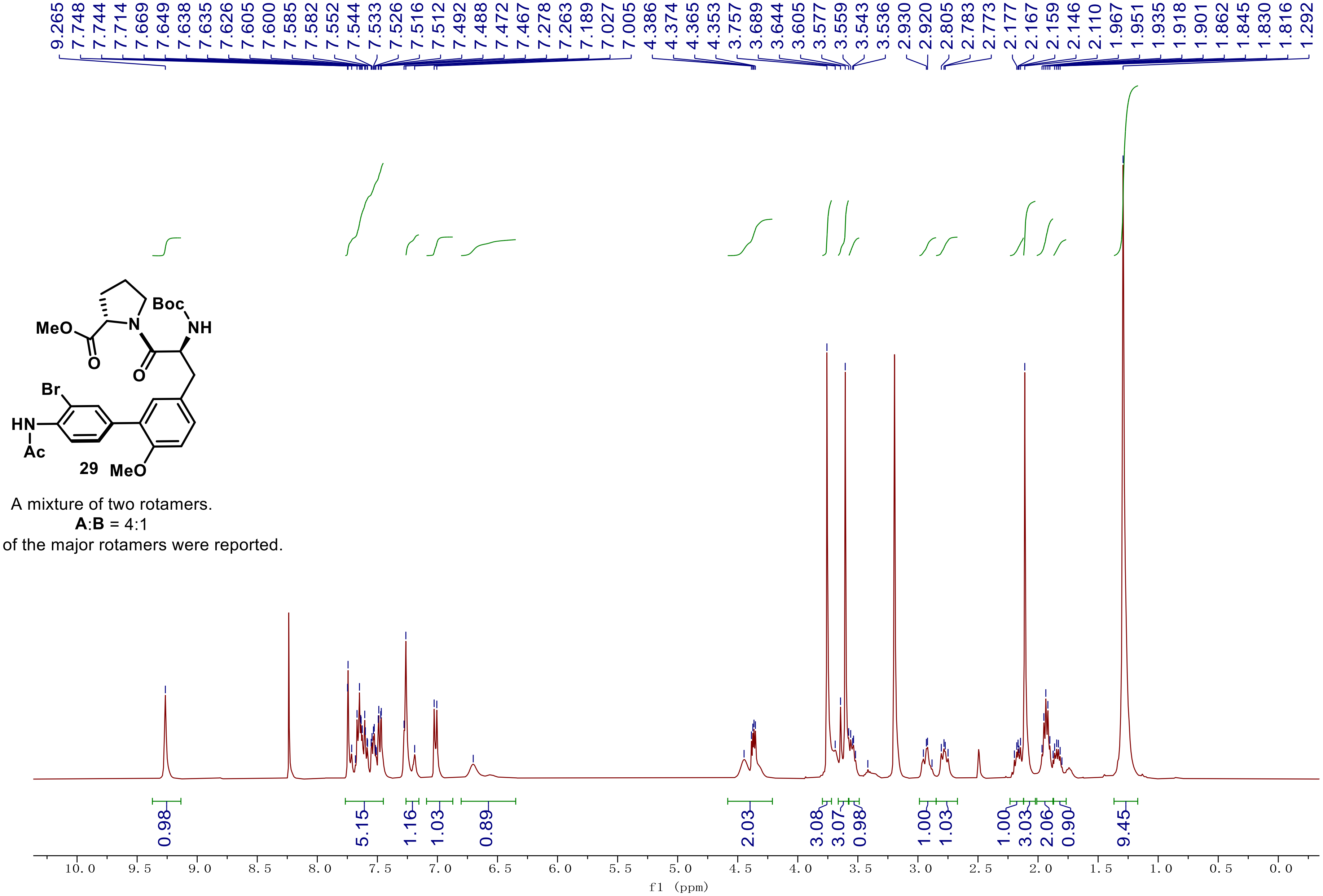

A mixture of two rotamers.
A:**B** = 4:1
SIganls of the major rotamers were reported.

Compound 29 ^{13}C NMR (101 MHz, DMSO-*d6*, 60 °C)

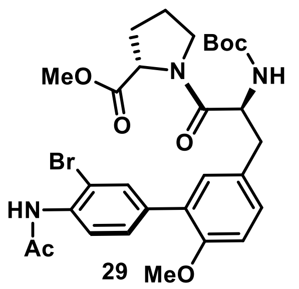

29

A mixture of two rotamers.
A:**B** = 4:1
SIganls of the major rotamers were reported.

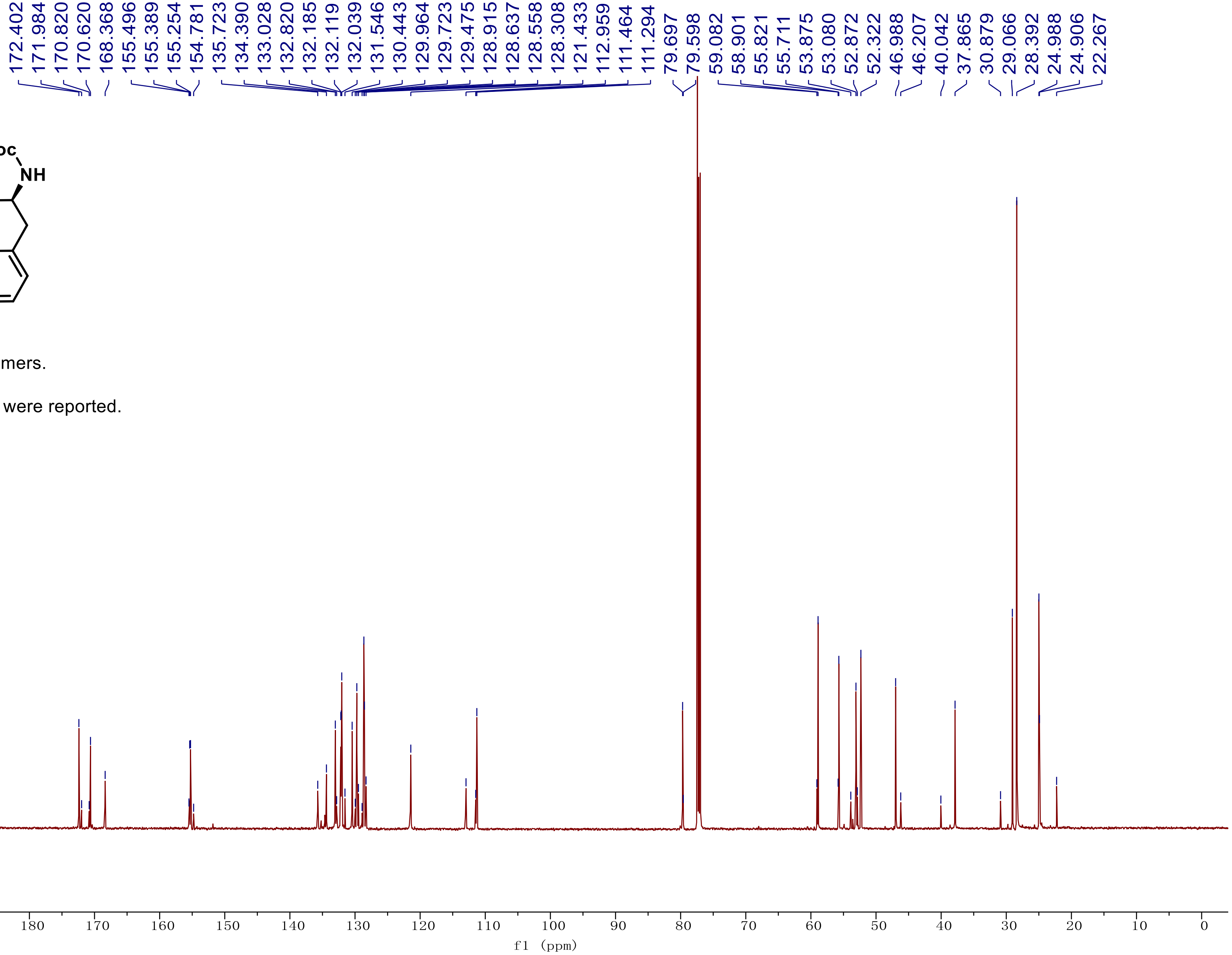

Compound 31 ^{1}H NMR (400 MHz, DMSO-*d6*, 60 °C)

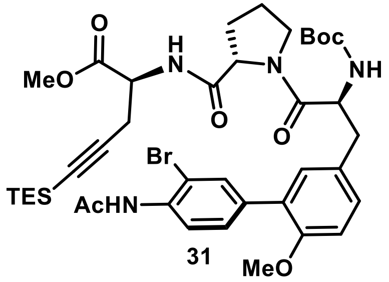

A mixture of two rotamers.

A:**B** = 4:1

Signals of the major rotamers were reported.

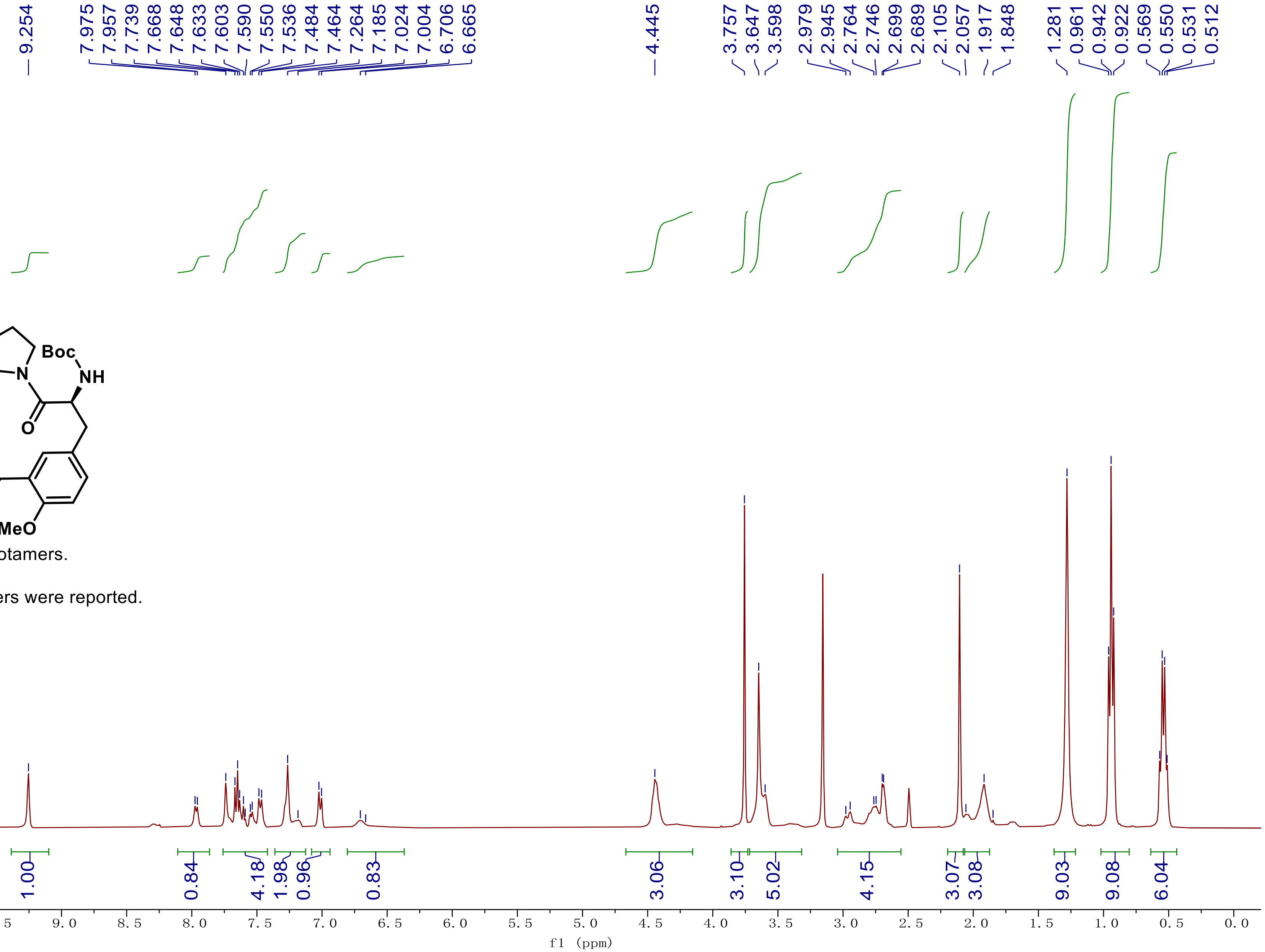

Compound 31 ^{13}C NMR (101 MHz, DMSO-*d6*, 60 °C)

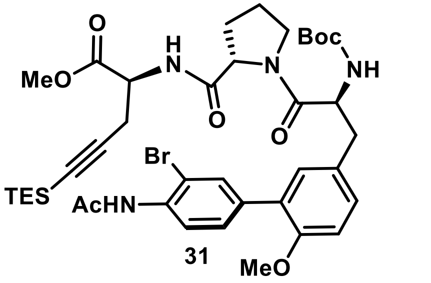

A mixture of two rotamers.
A:**B** = 4:1
Signals of the major rotamers were reported.

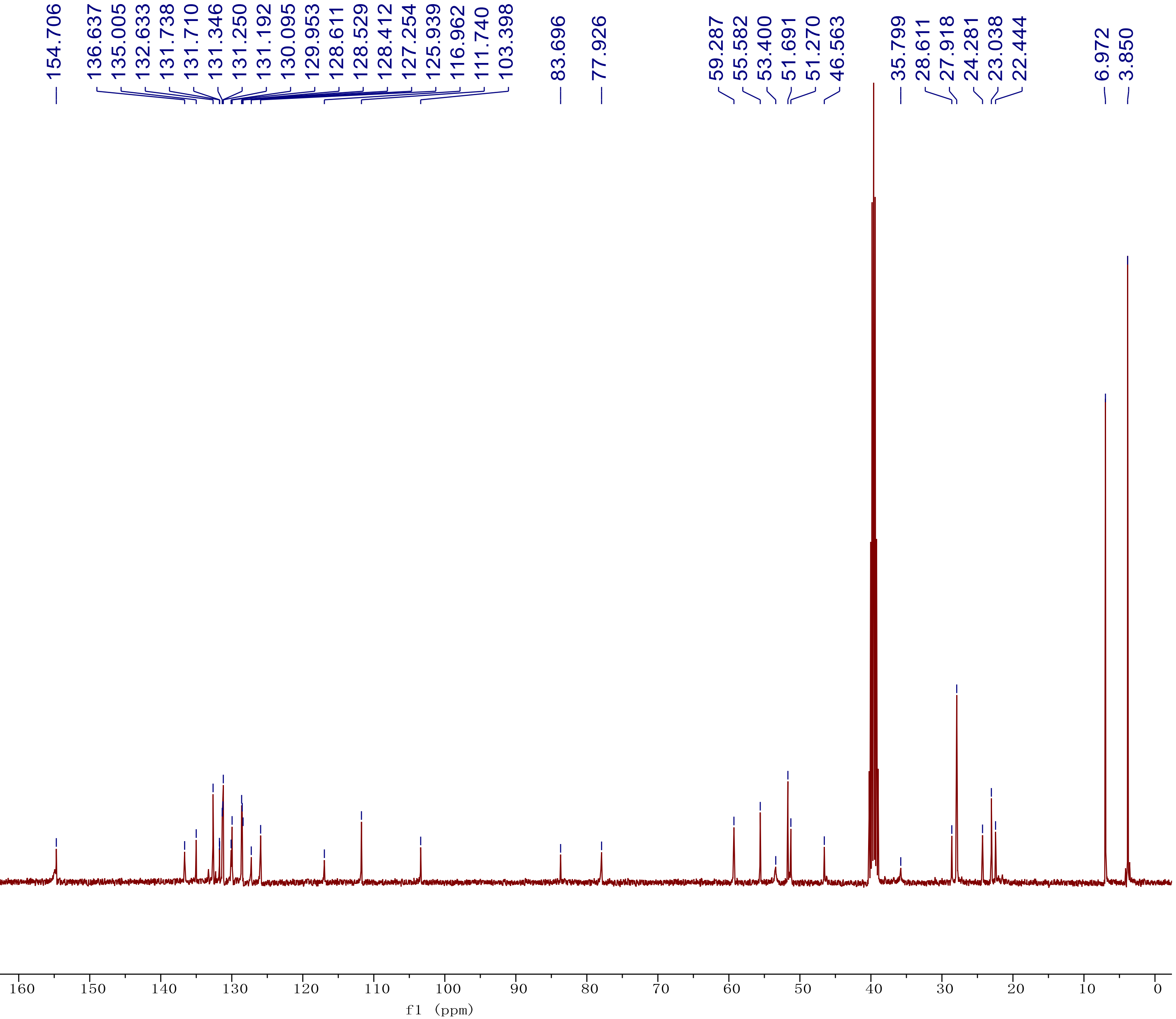

Compound 32 ^{1}H NMR (600 MHz, $CDCl_3$)

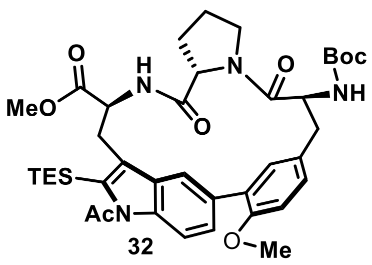

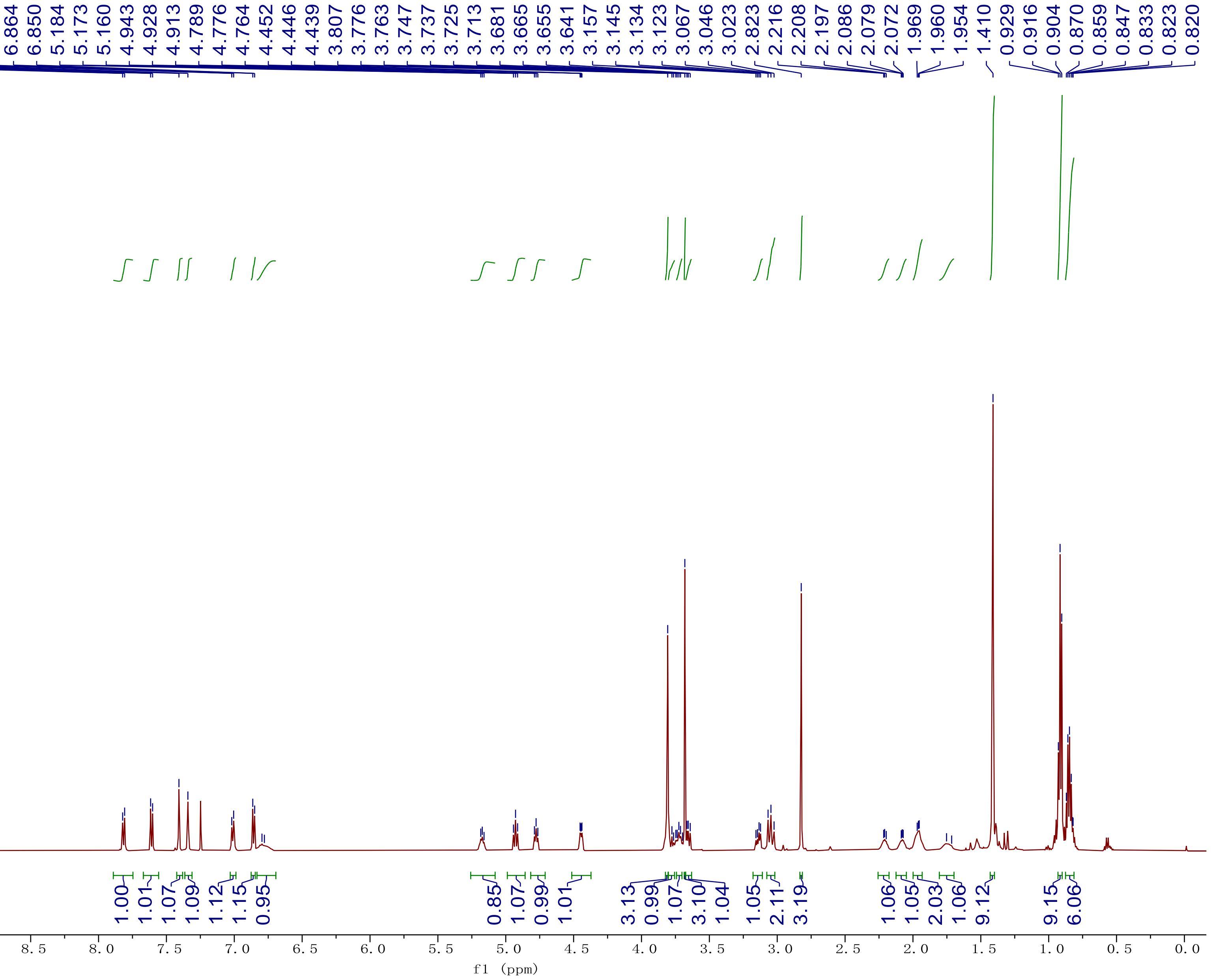

Compound 32 ^{13}C NMR (151 MHz, $CDCl_3$)

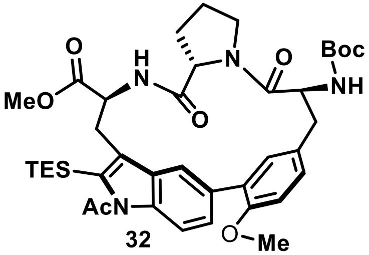
32

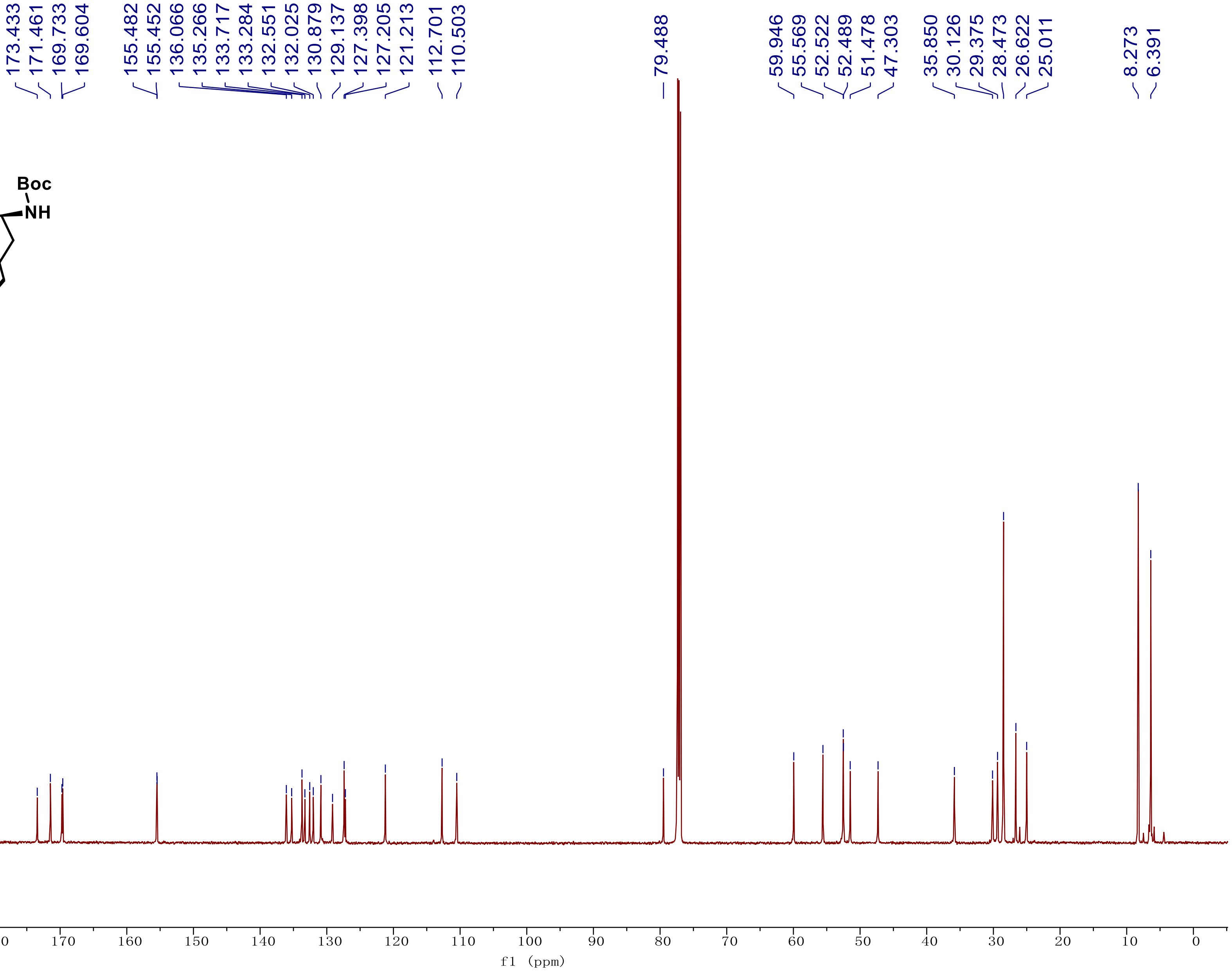

Compound 32 ^{1}H-^{1}H COSY NMR (600 MHz, $CDCl_3$)

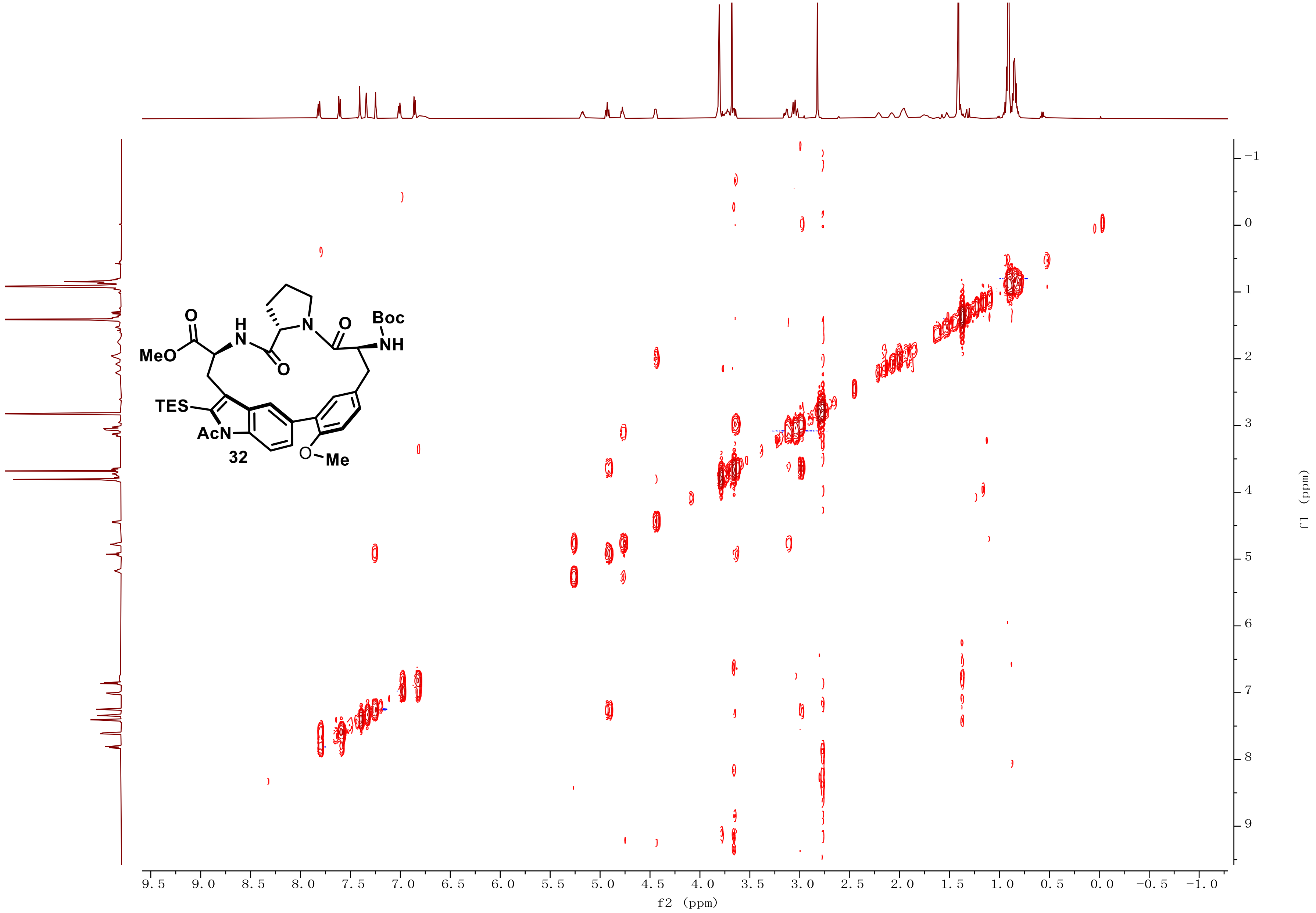

Compound 32 ^{1}H-^{13}C HSQC NMR (600 MHz, $CDCl_3$)

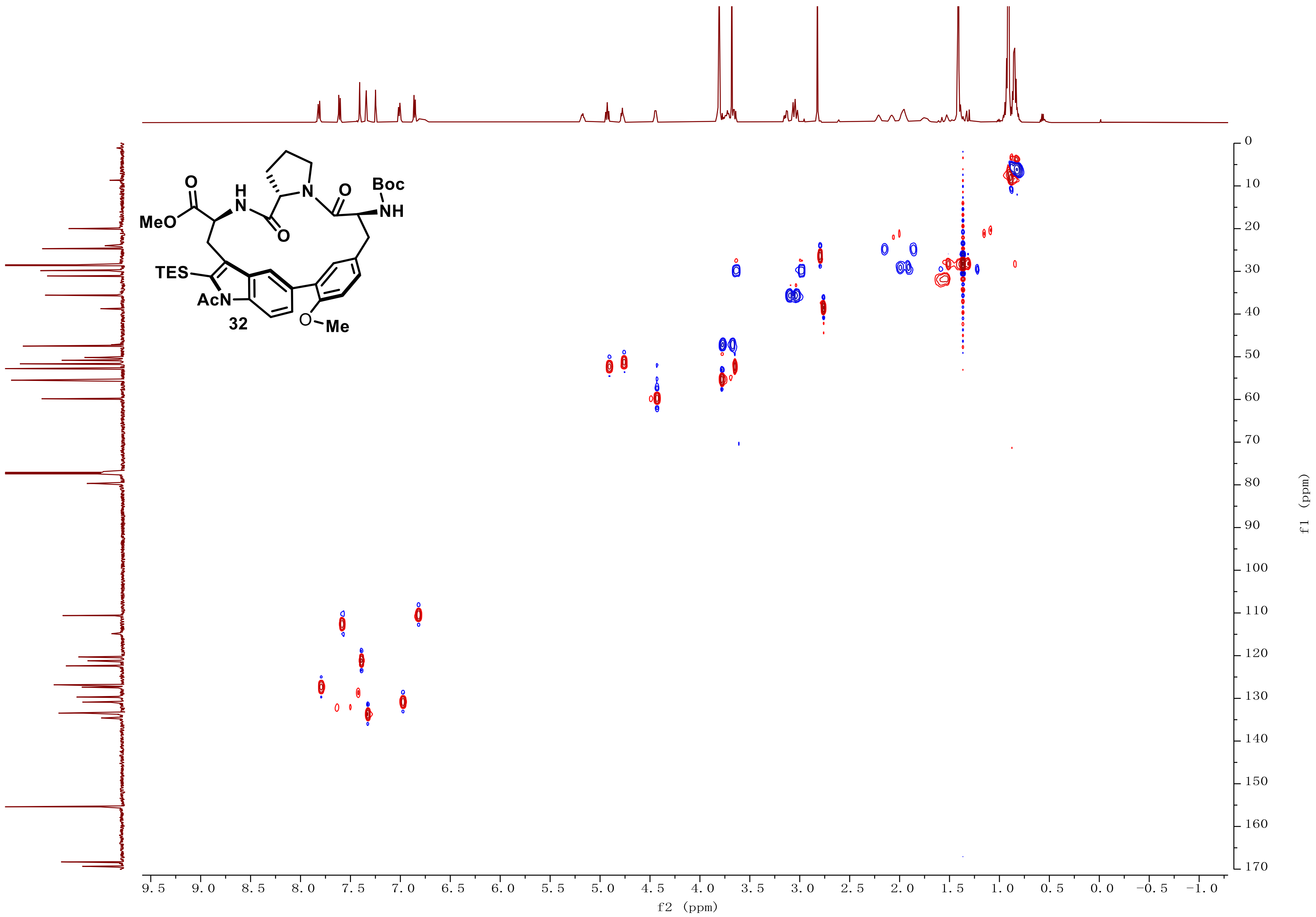

Compound 32 ^{1}H-^{1}H NOESY NMR (600 MHz, $CDCl_3$)

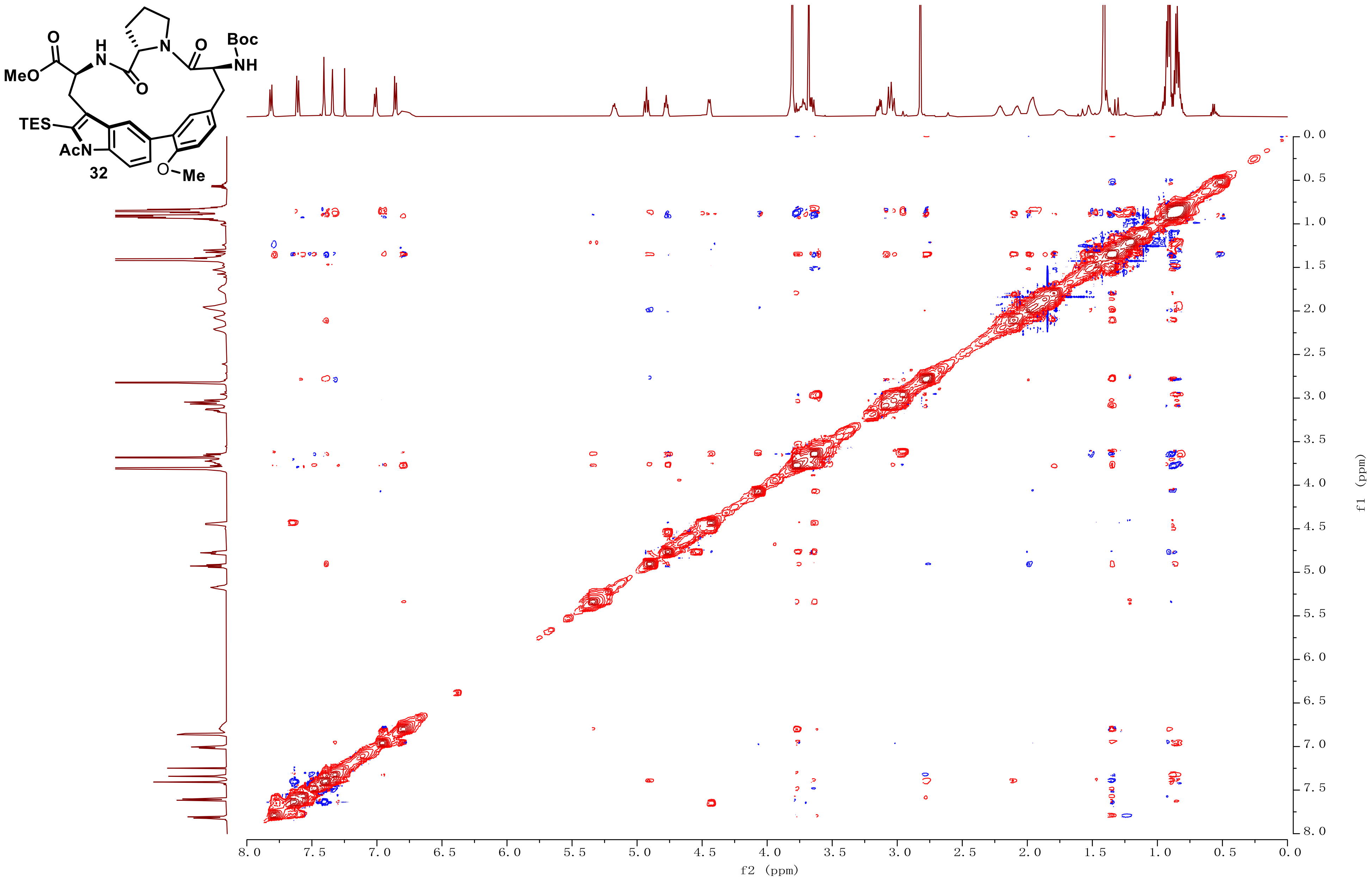

Compound 33 ^{1}H NMR (400 MHz, $CDCl_3$)

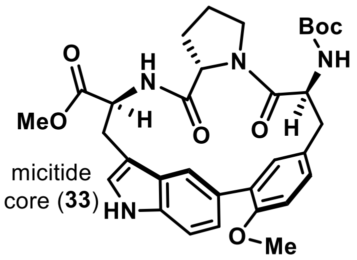

micitide core (**33**)

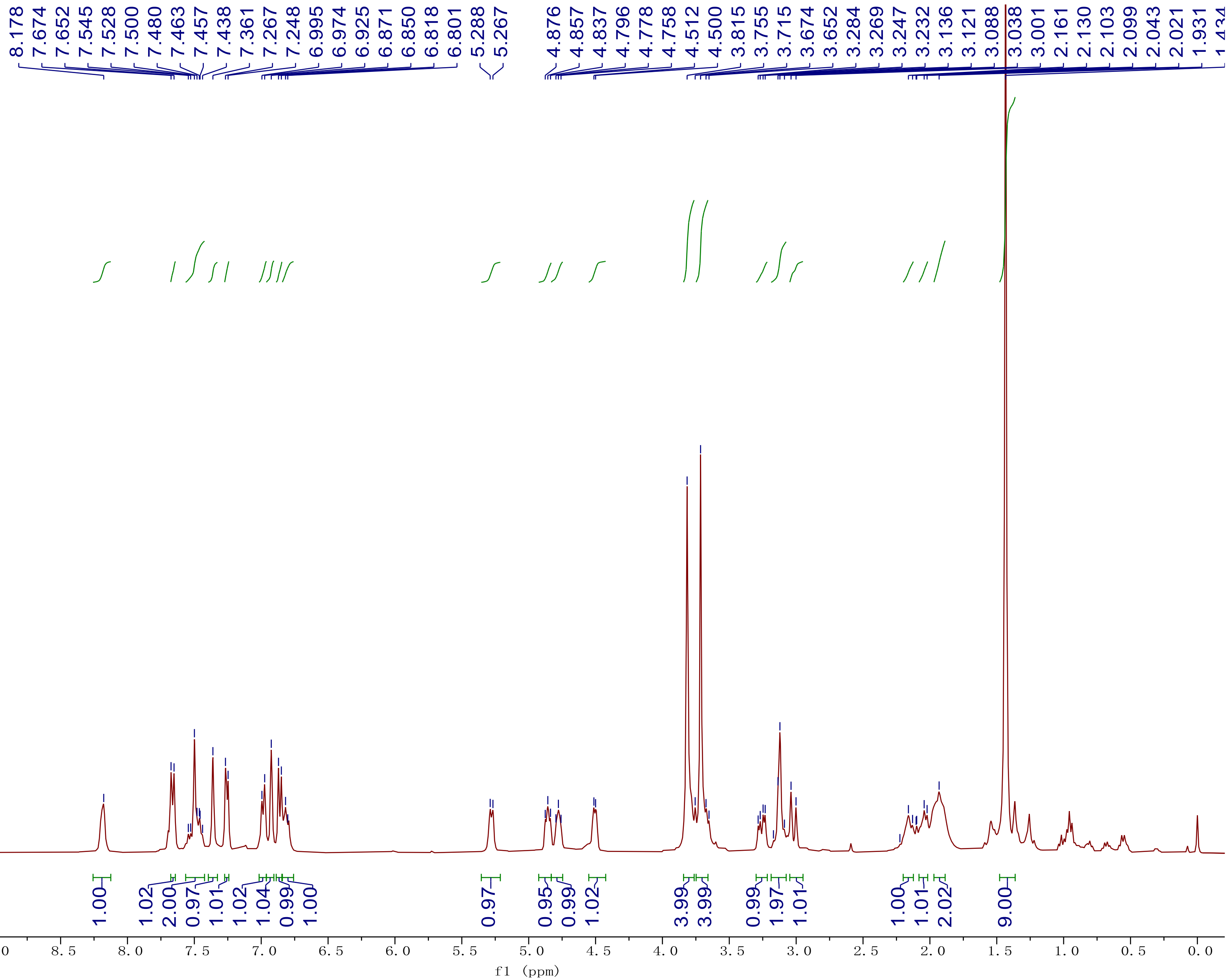

Compound 33 ¹H NMR (101 MHz, CDCl₃)

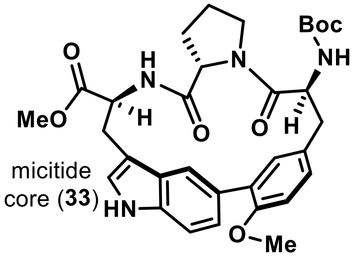

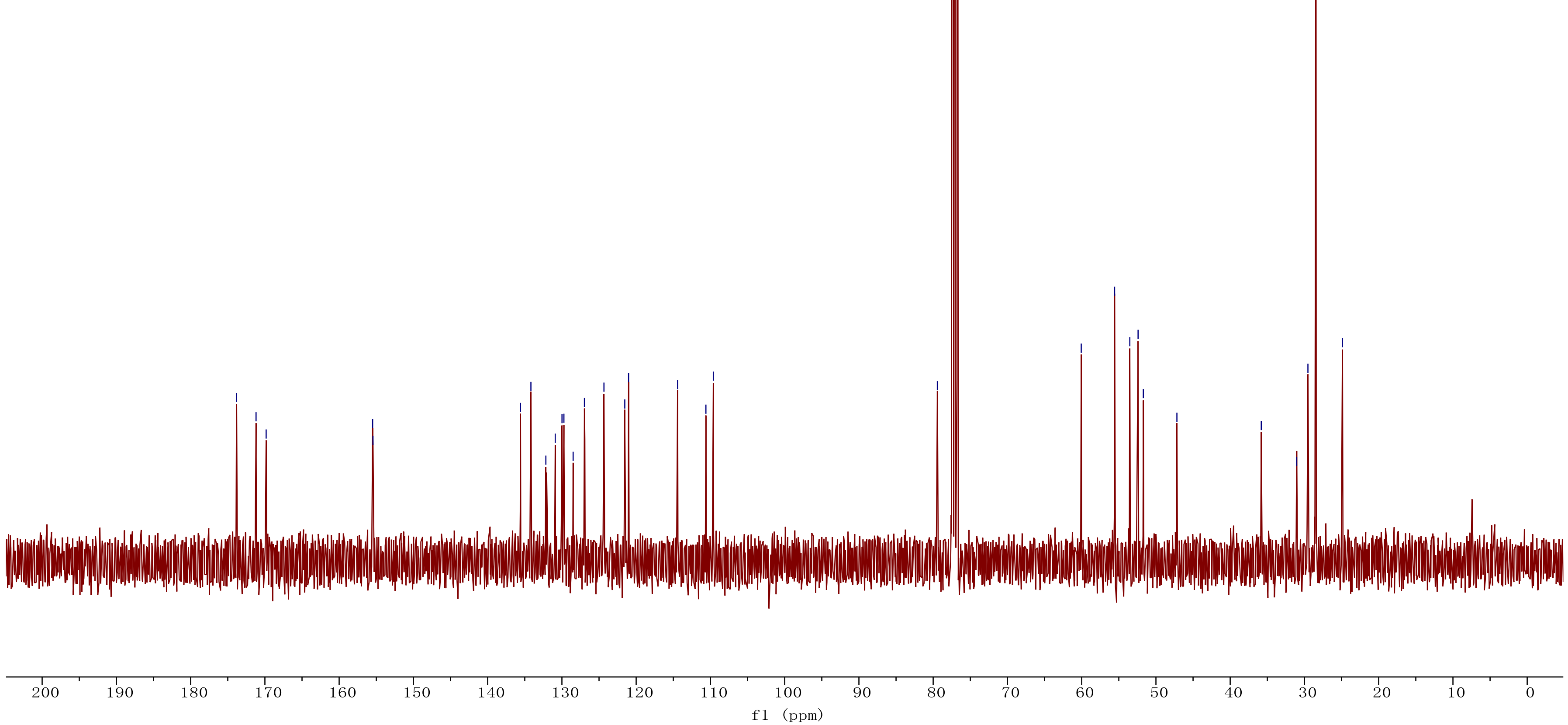

Compound 35 ^{1}H NMR (400 MHz, $CDCl_3$)

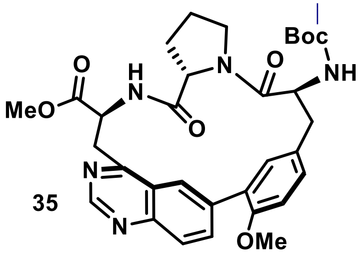

35

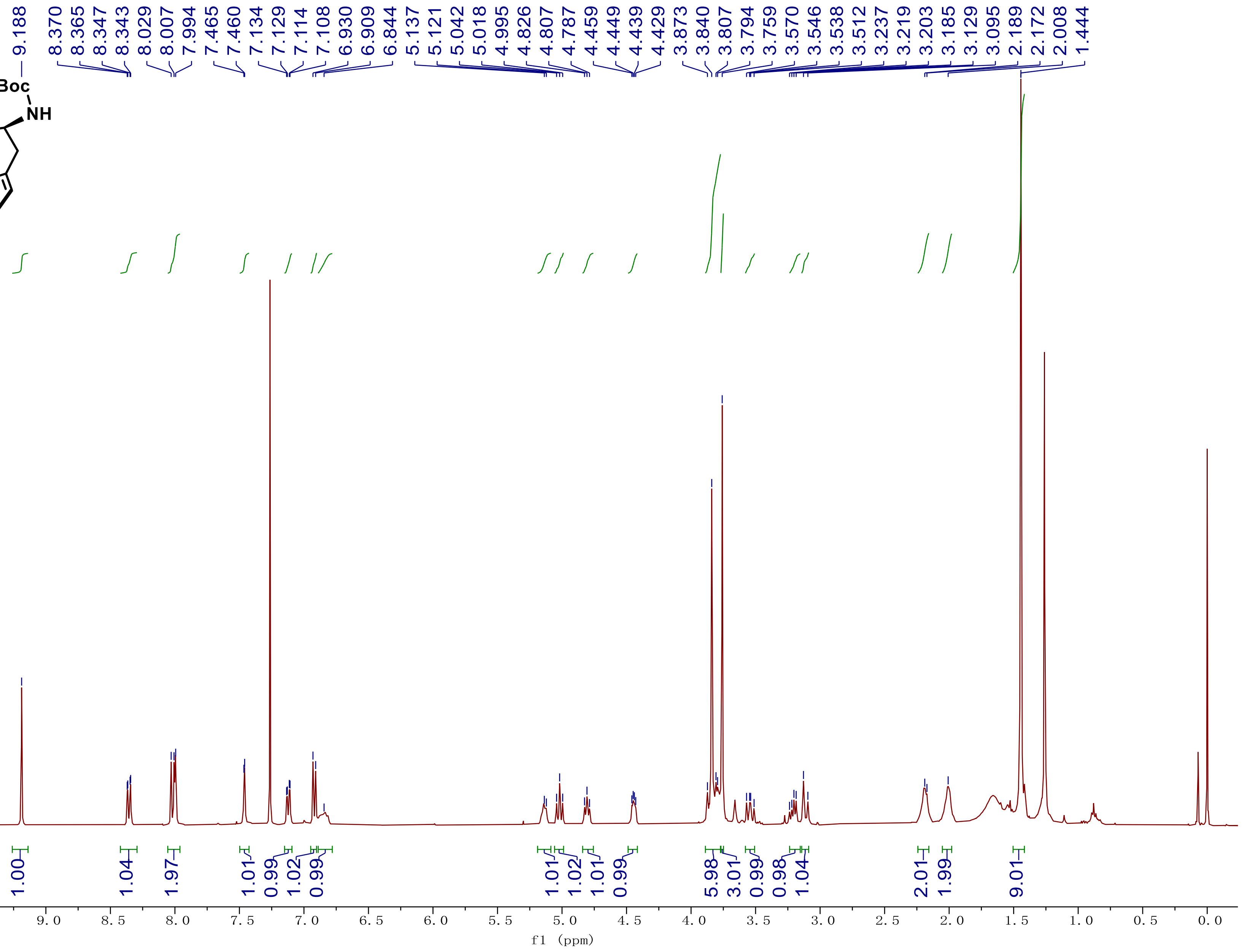

Compound 35 ^{13}C NMR (101 MHz, $CDCl_3$)

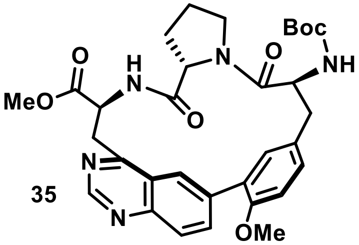

35

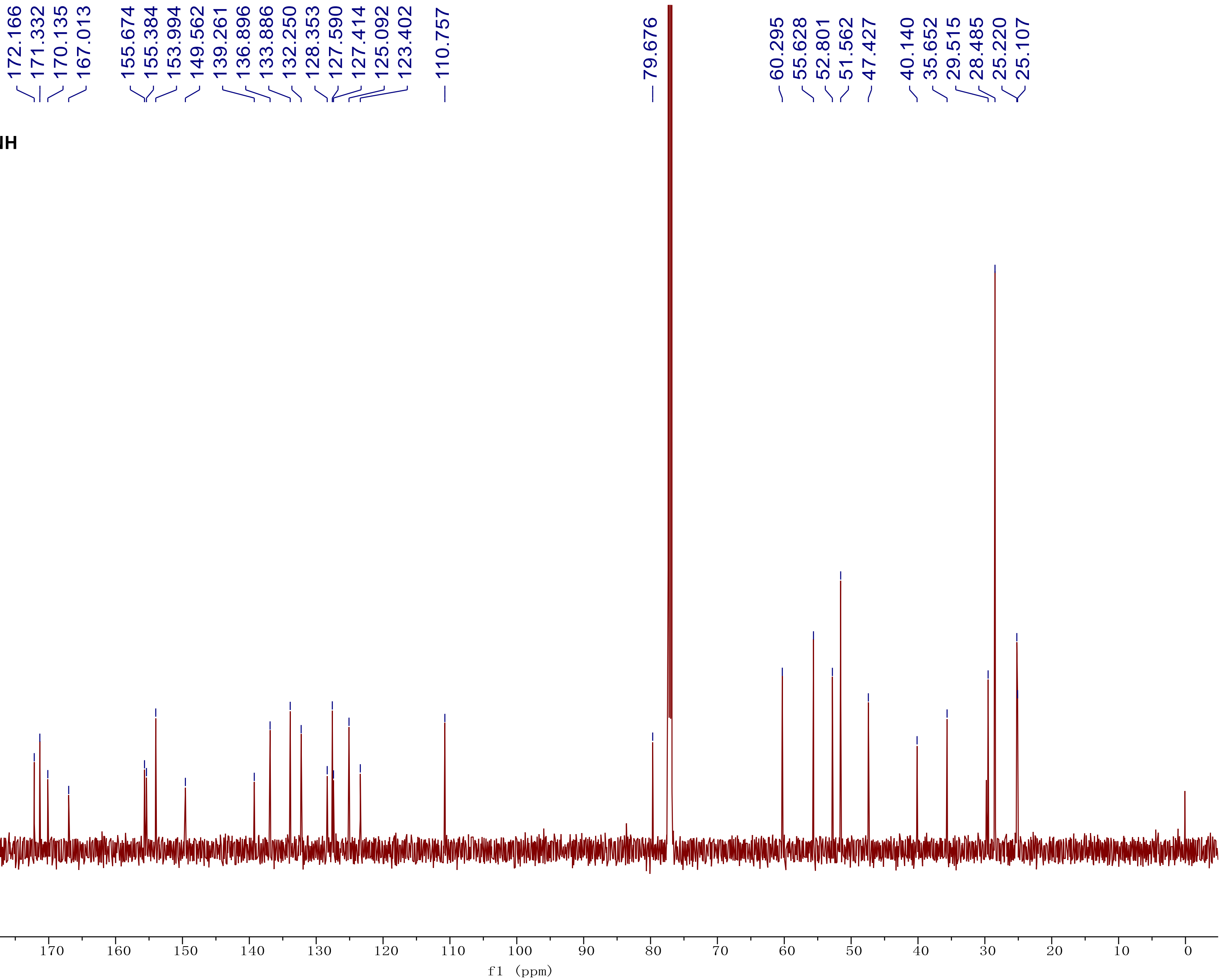

Compound 35 ^1H-^1H COSY (400 MHz, $CDCl_3$)

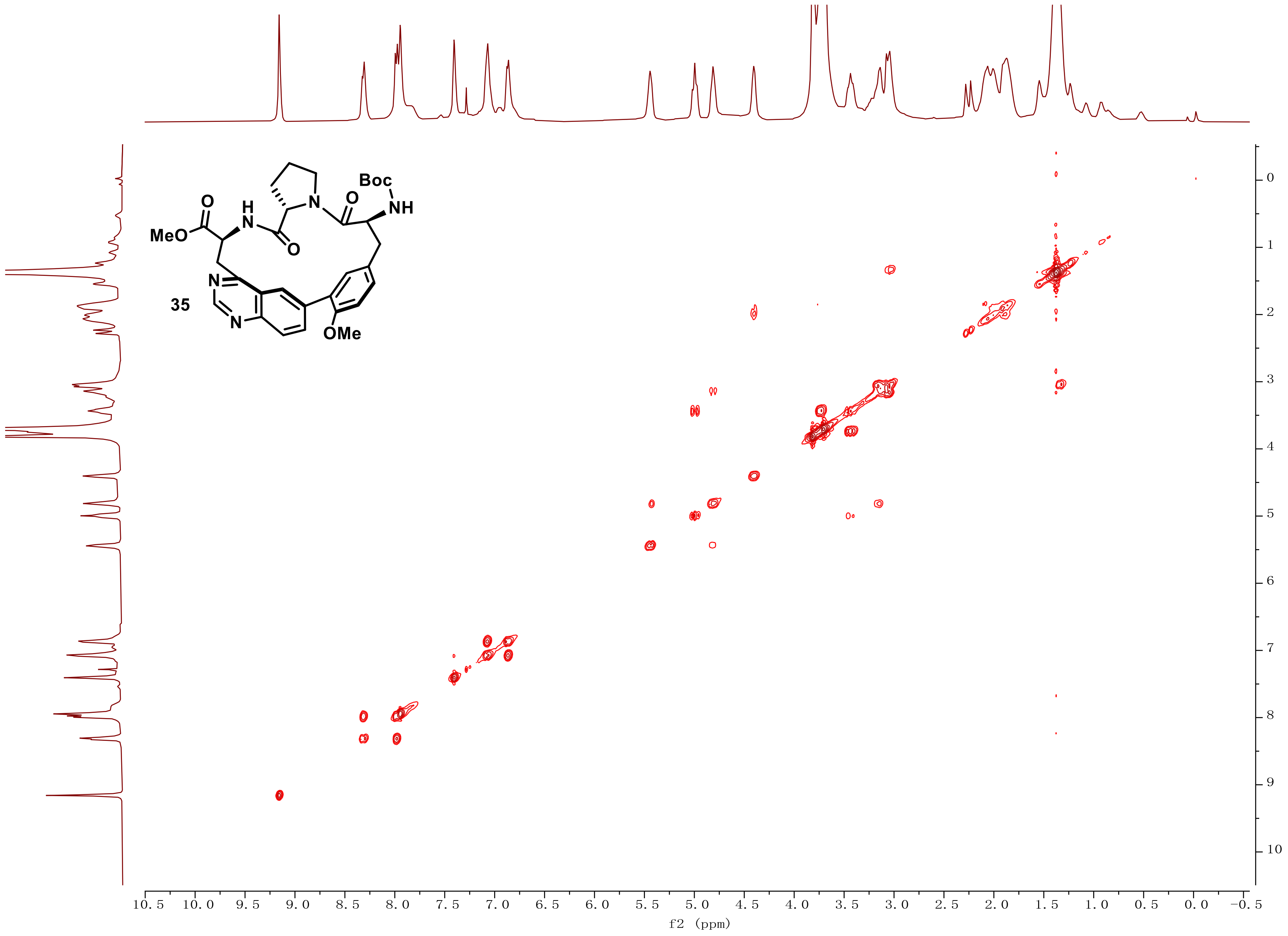

Compound 35 ^{1}H-^{13}C HSQC (400 MHz, $CDCl_3$)

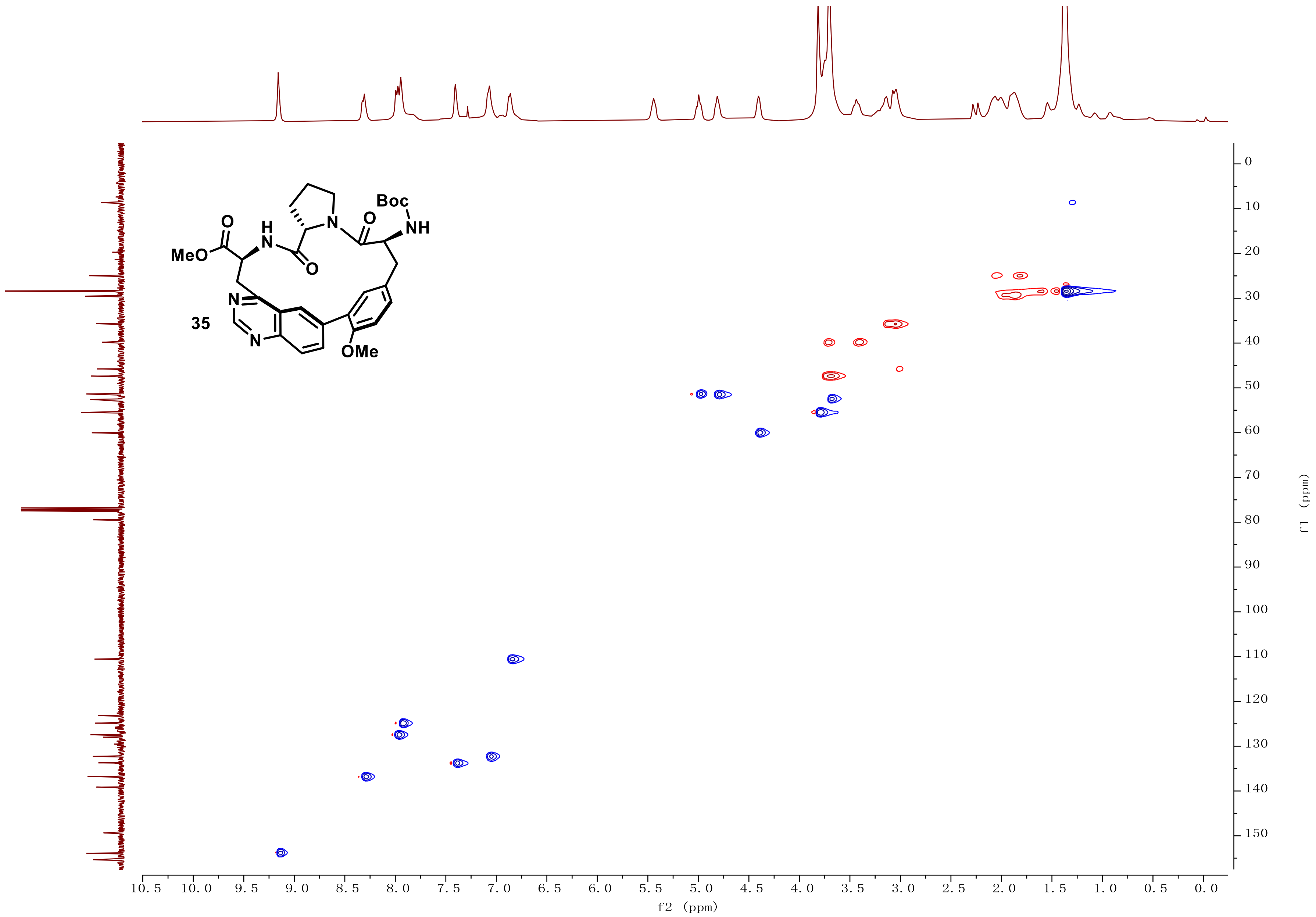

Compound 35 ^{1}H-^{1}H ROESY (400 MHz, CDCl$_3$)

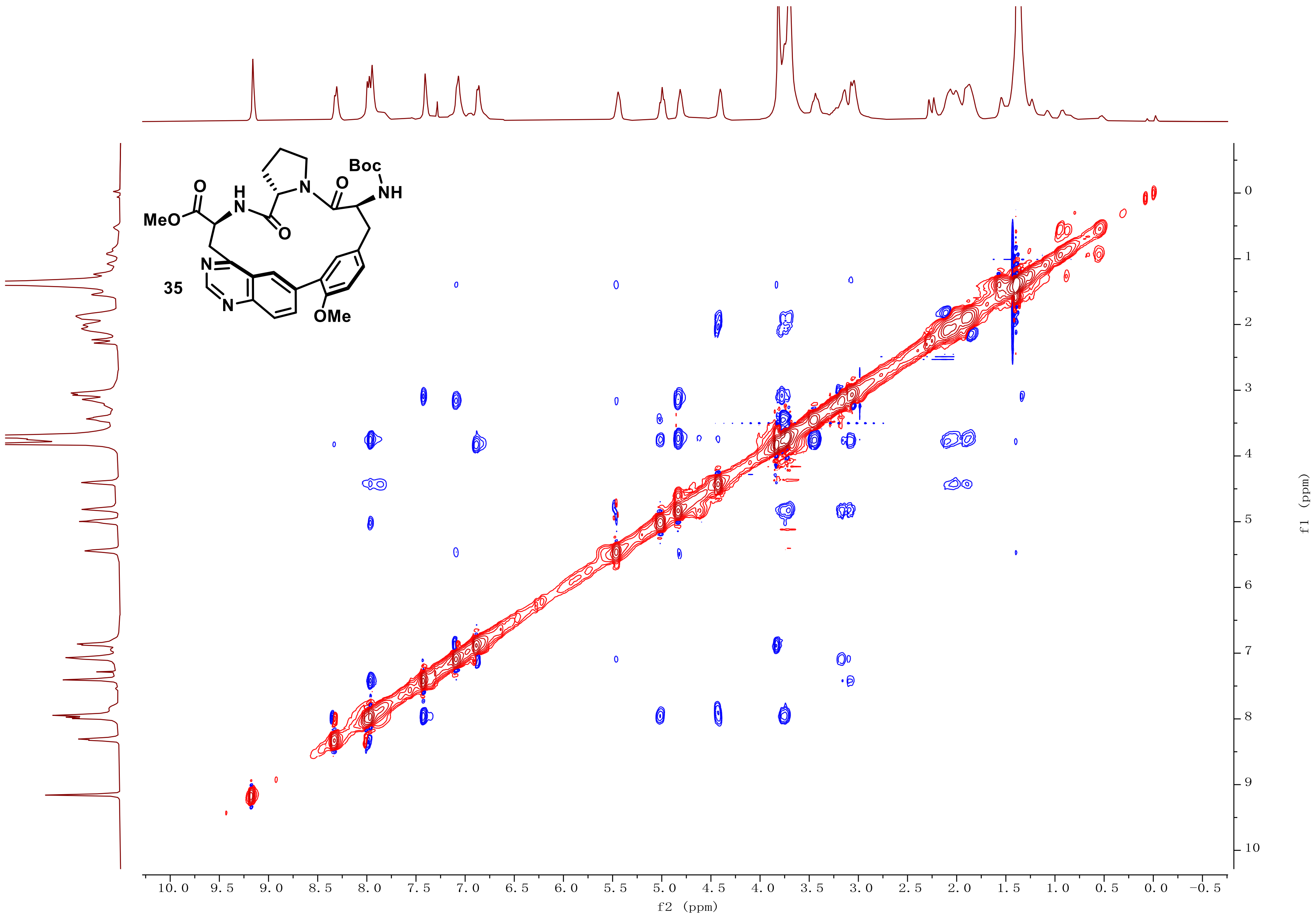

Compound 39 ^{1}H NMR (400 MHz, $CDCl_3$)

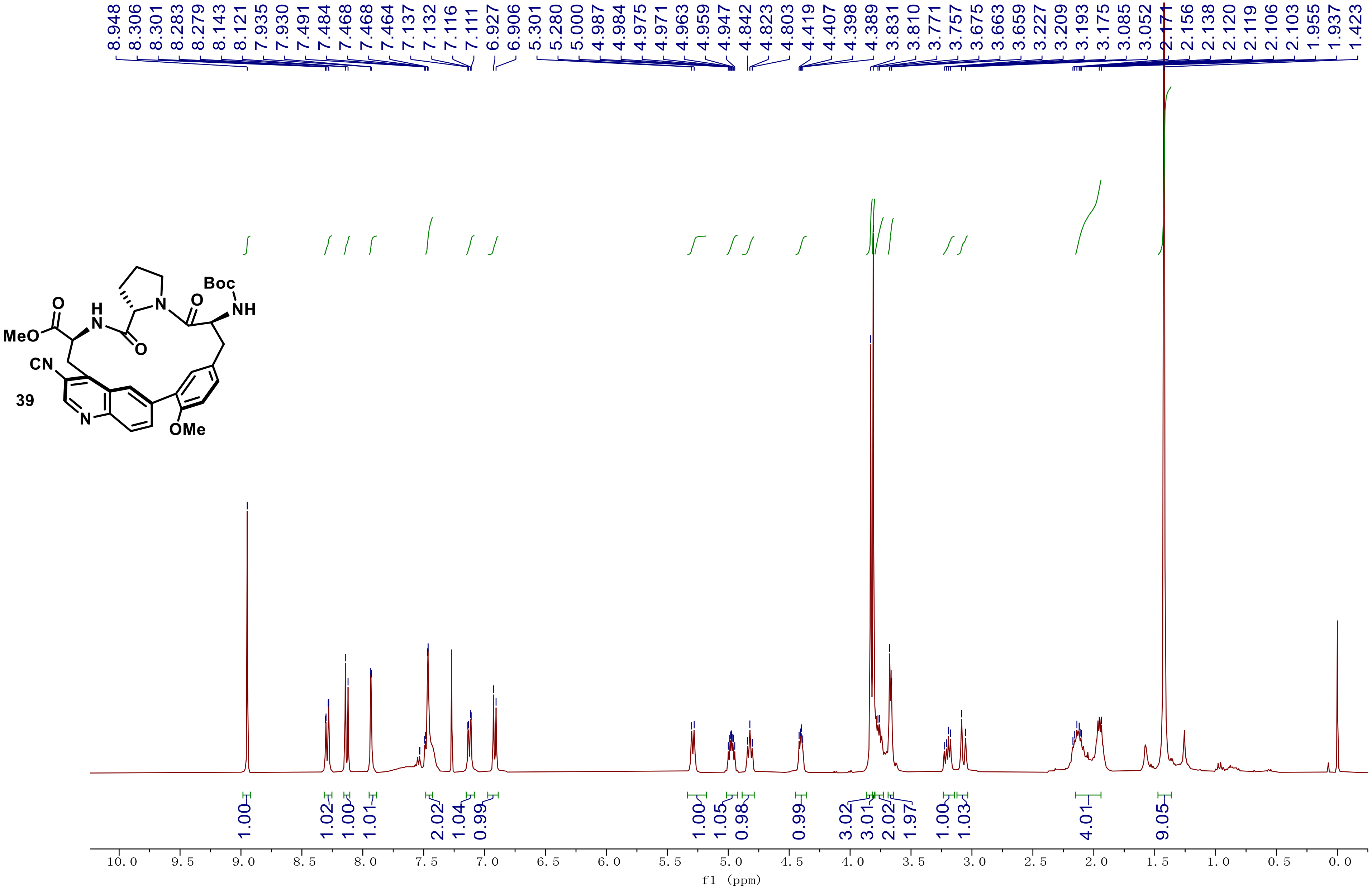

Compound 39 ^{13}C NMR (101 MHz, CDCl$_3$)

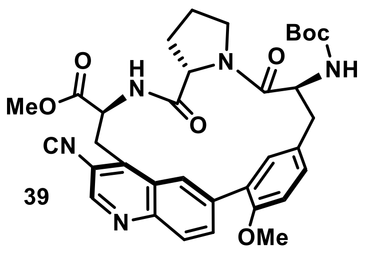

39

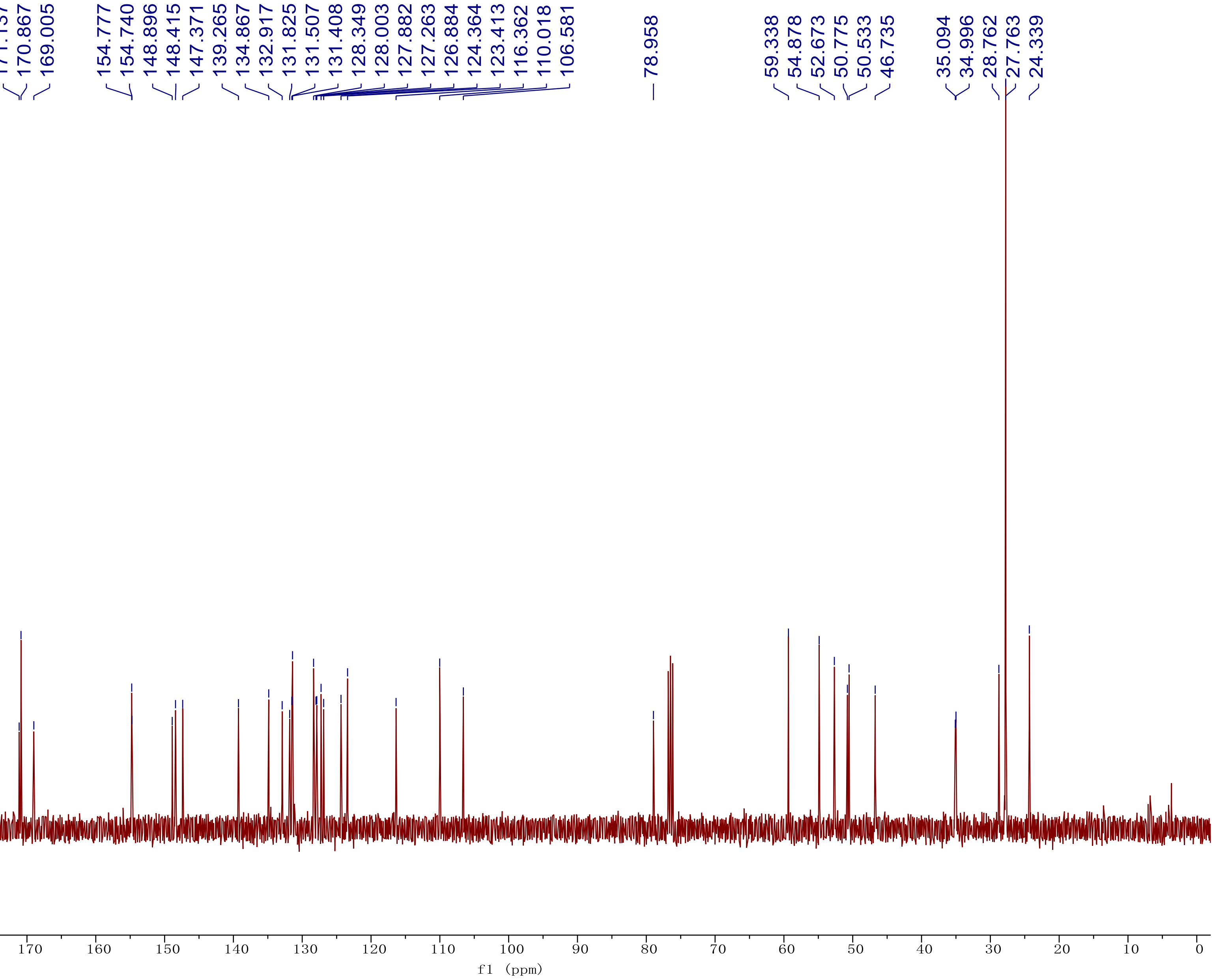

Compound 40 ^{1}H NMR (400 MHz, $CDCl_3$)

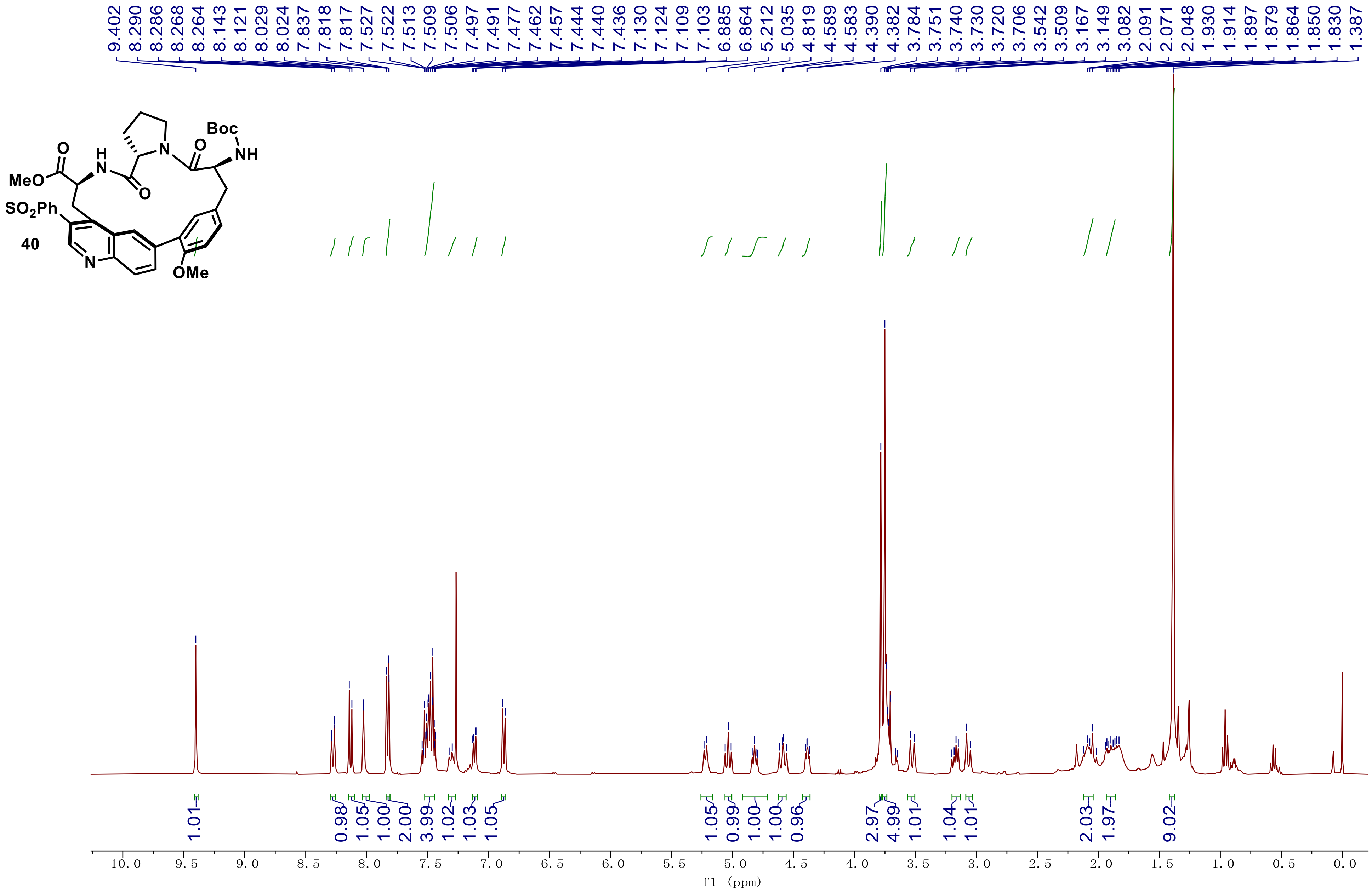

Compound 40 ^{13}C NMR (101 MHz, CDCl$_3$)

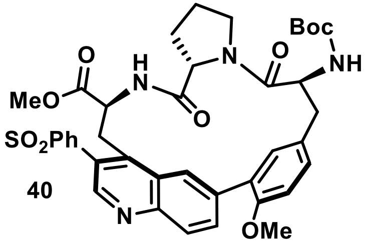

40

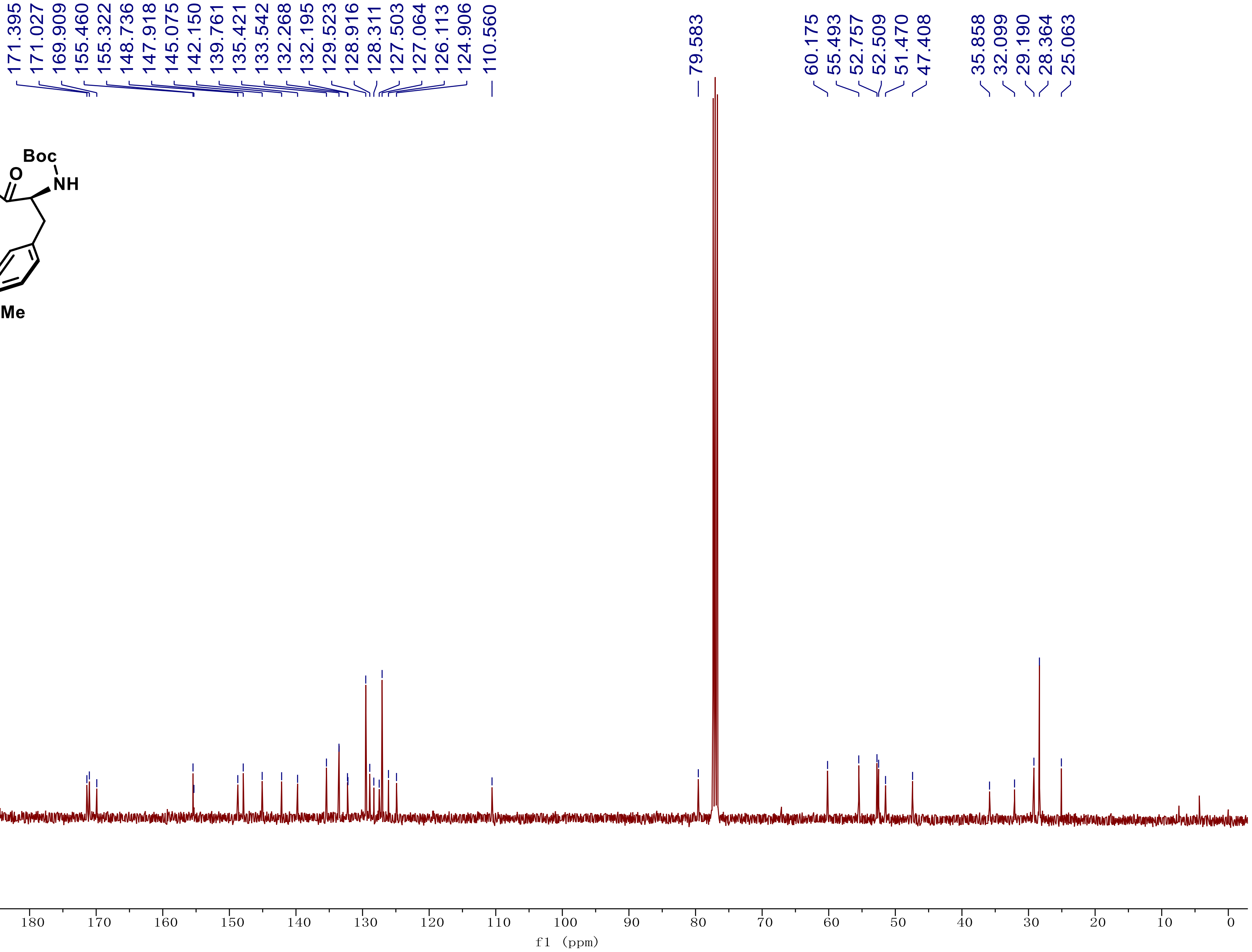

Compound 40 ^{1}H-^{1}H COSY NMR (400 MHz, $CDCl_3$)

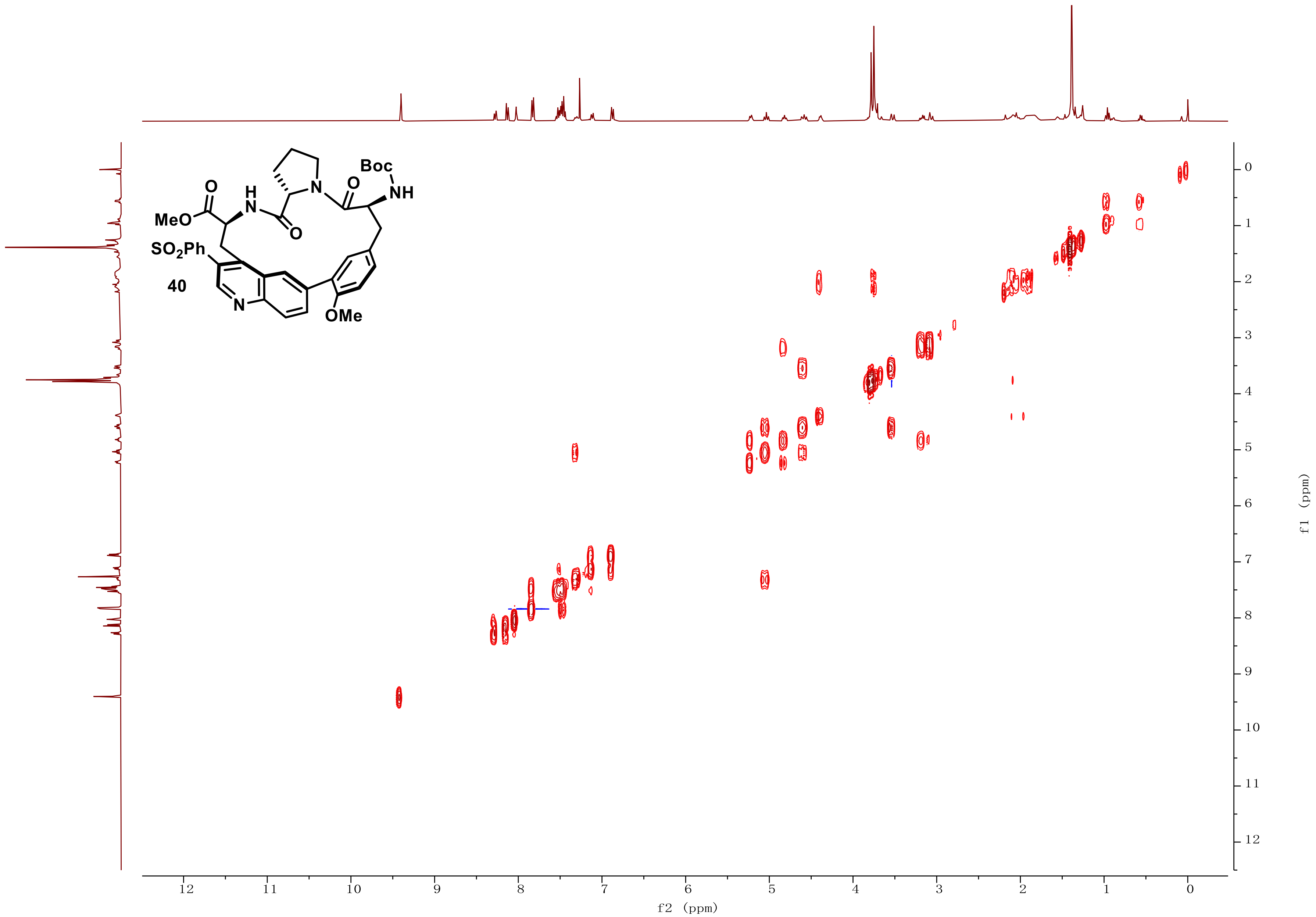

Compound 40 ^{1}H-^{13}C HSQC NMR (400 MHz, $CDCl_3$)

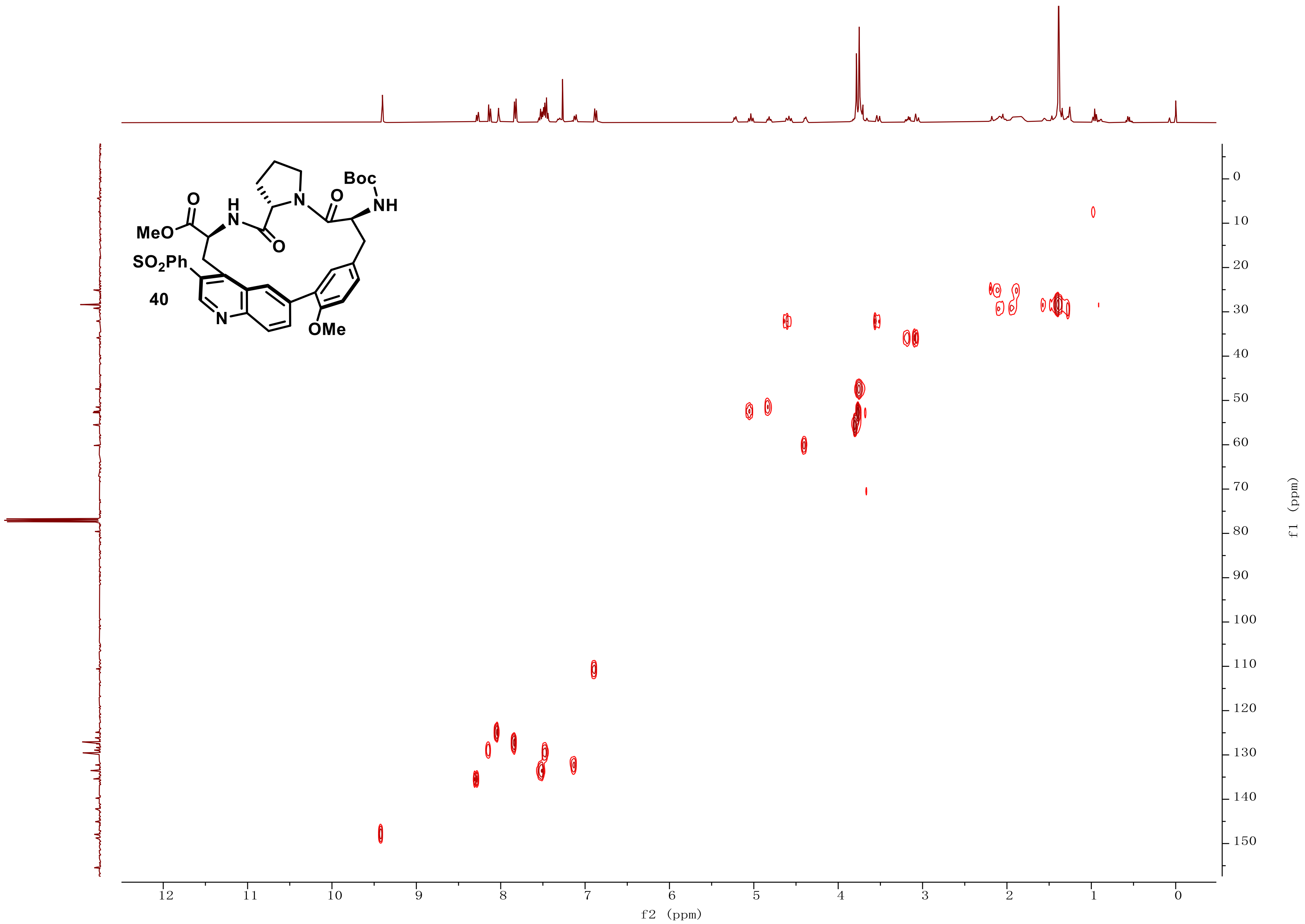

Compound 42 ^{1}H NMR (400 MHz, CD_3OD)

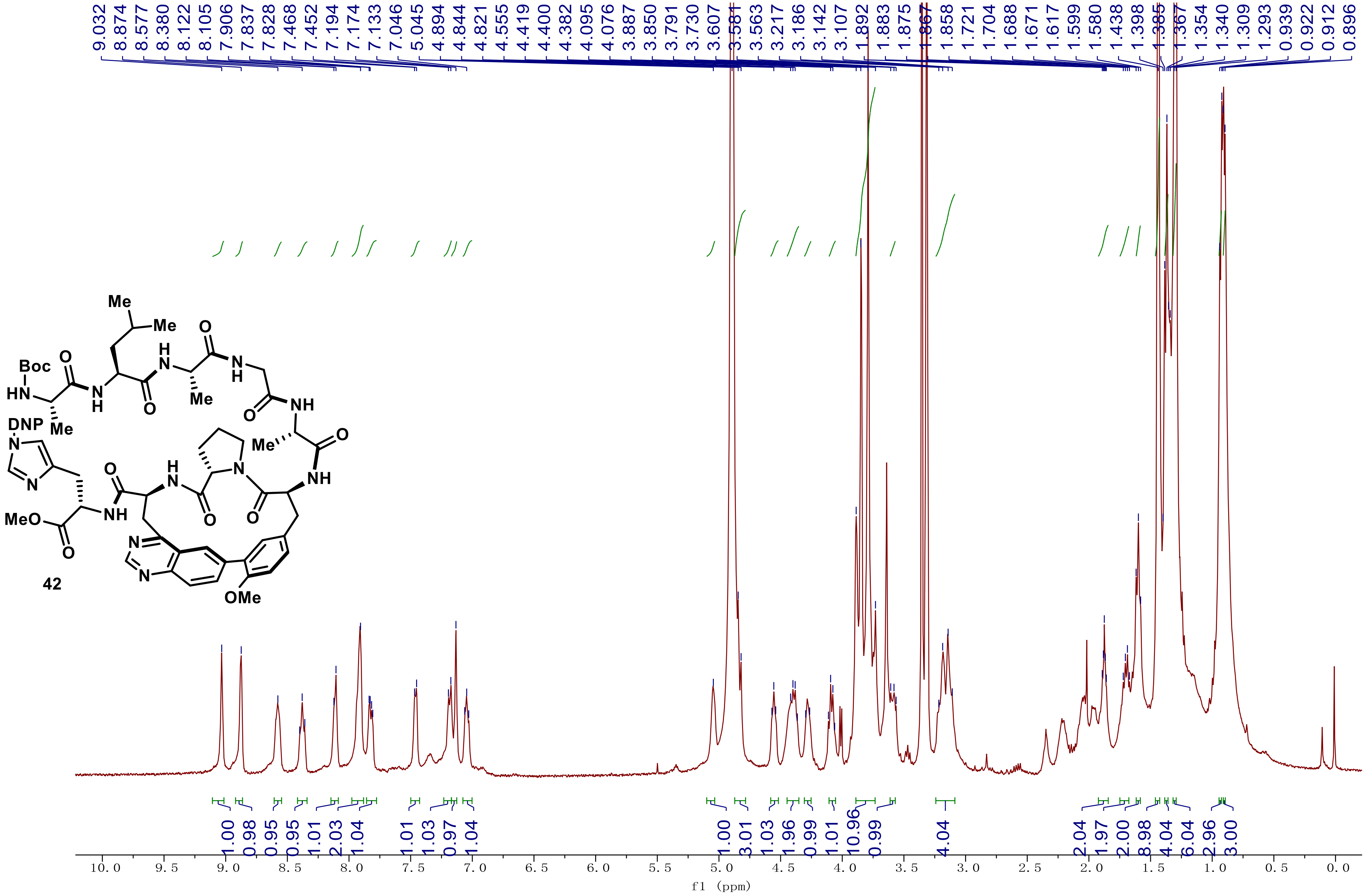

Compound 42 ^{13}C NMR (101 MHz, CD_3OD)

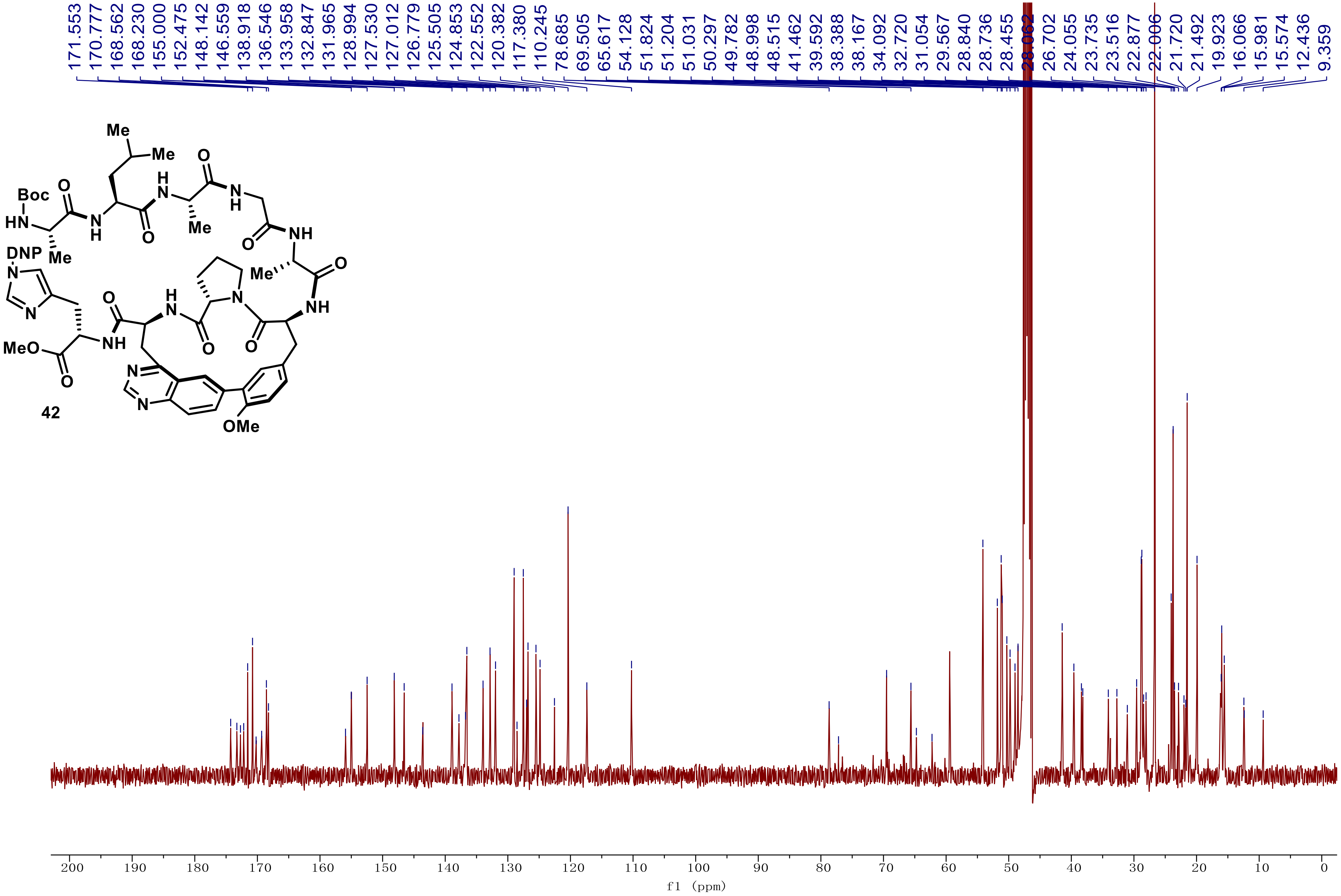

Compound 43 ^{1}H NMR (400 MHz, DMSO-*d6*)

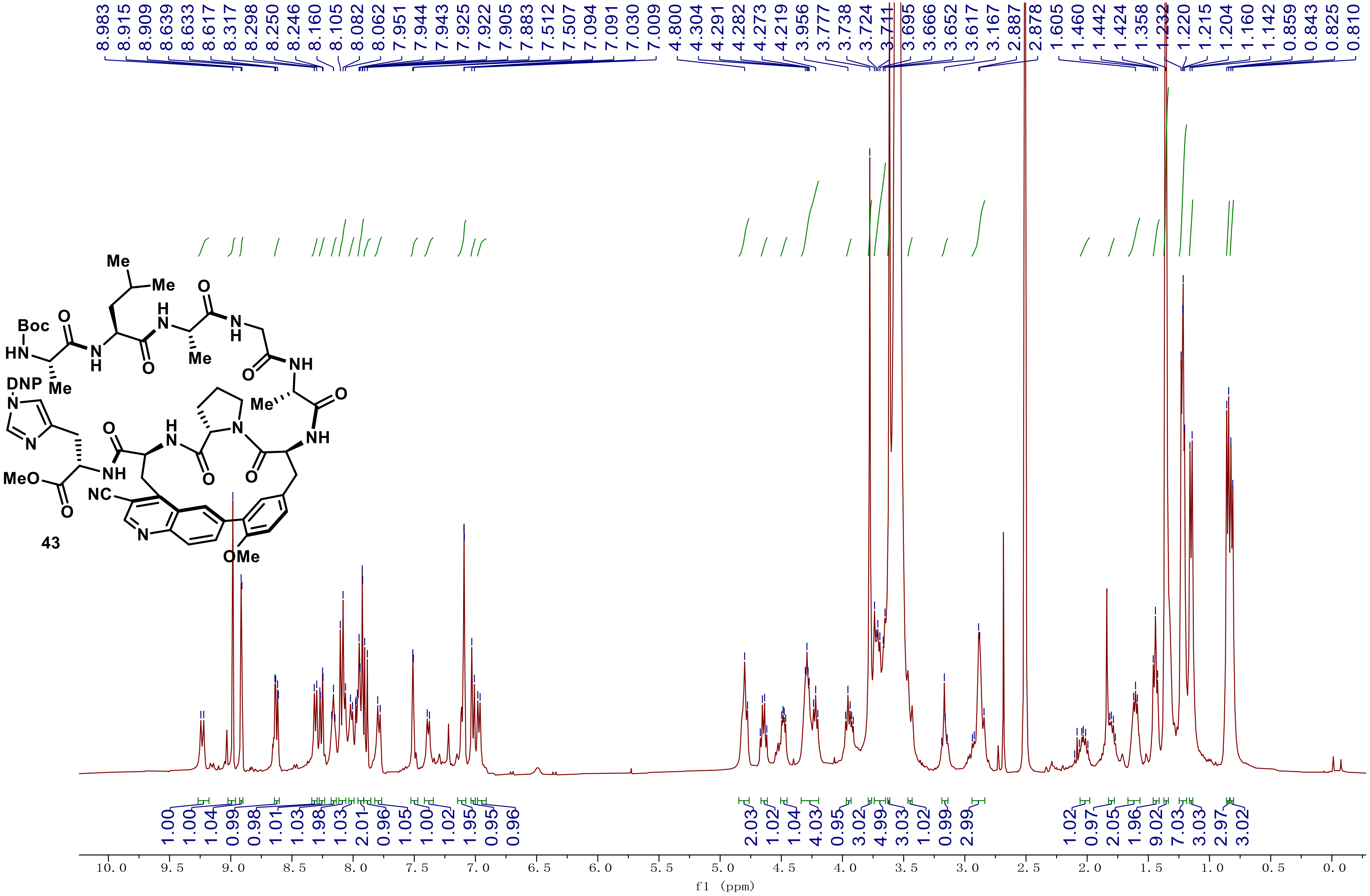

Compound 43 ^{13}C NMR (101 MHz, DMSO-*d6*)

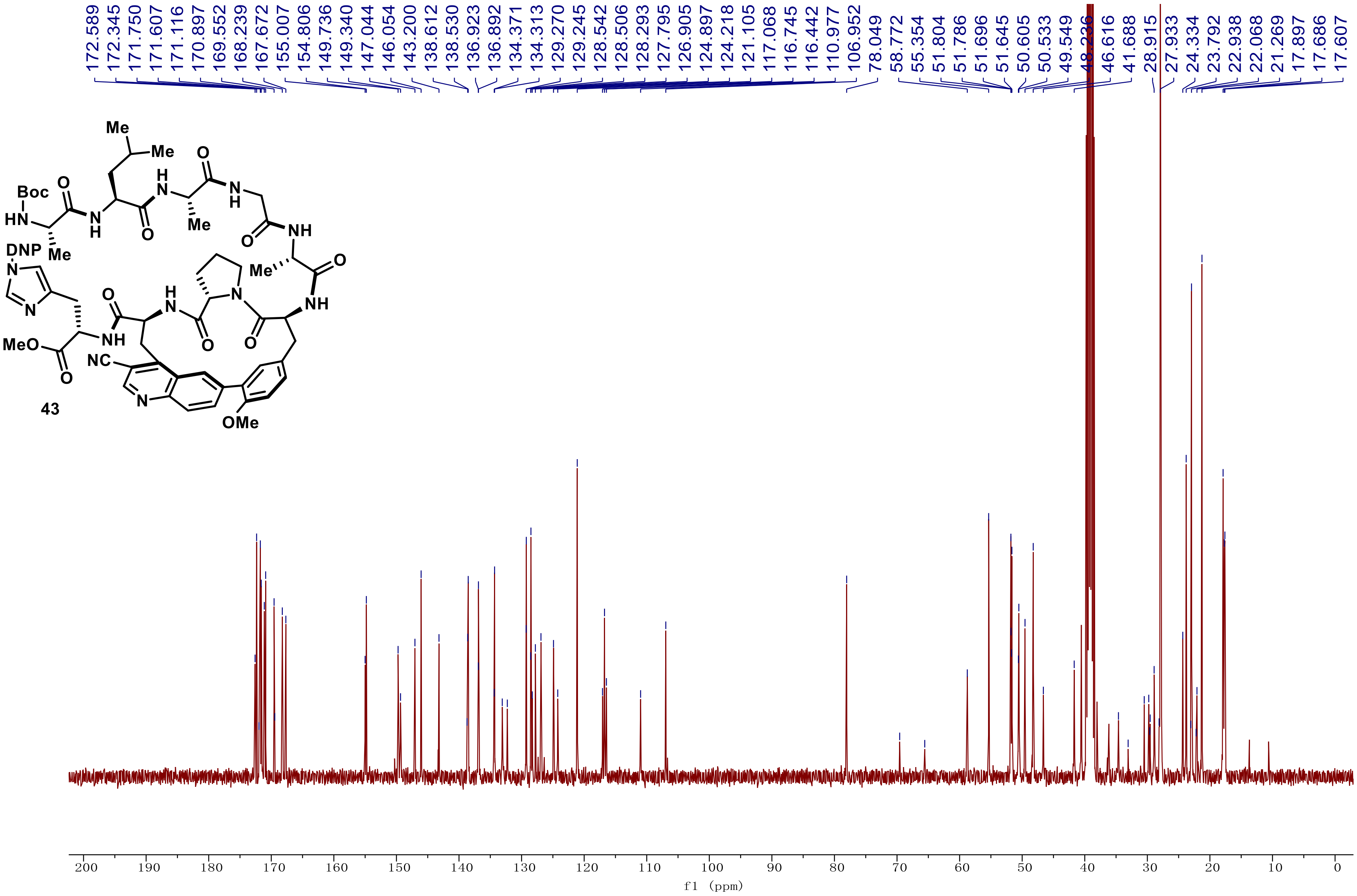

Compound 44 ^{1}H NMR (400 MHz, DMSO-*d6*)

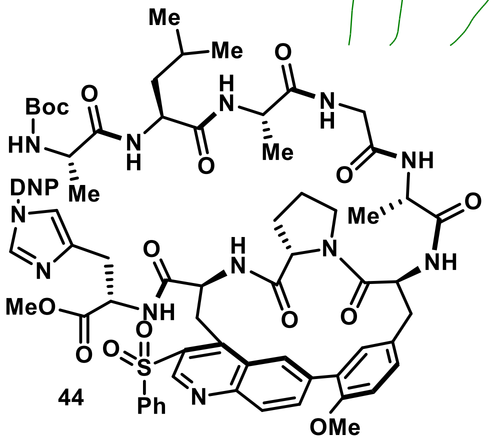

44

mixtures of multiple rotamers.

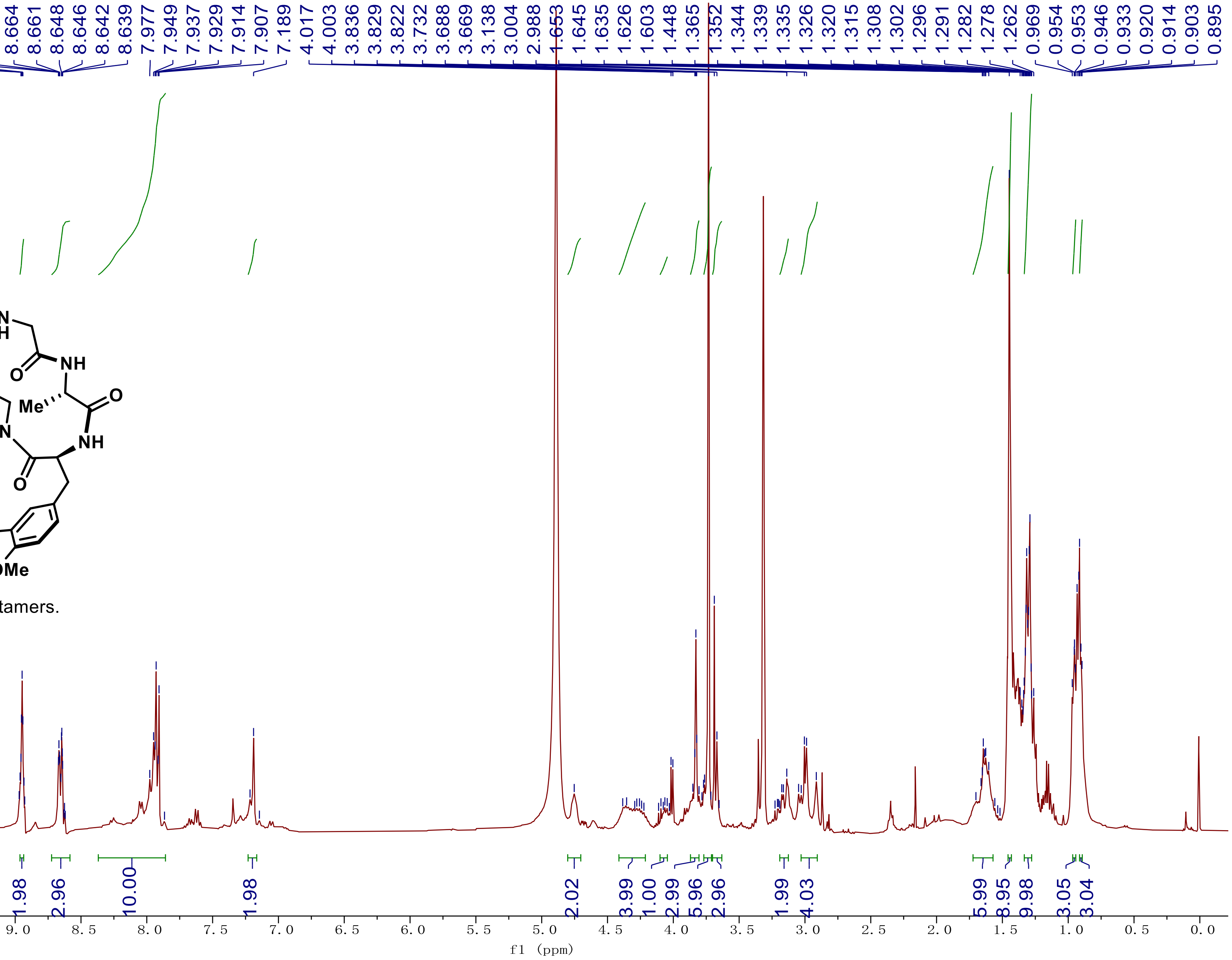

Compound 44 ^{13}C NMR (101 MHz, DMSO-*d6*)

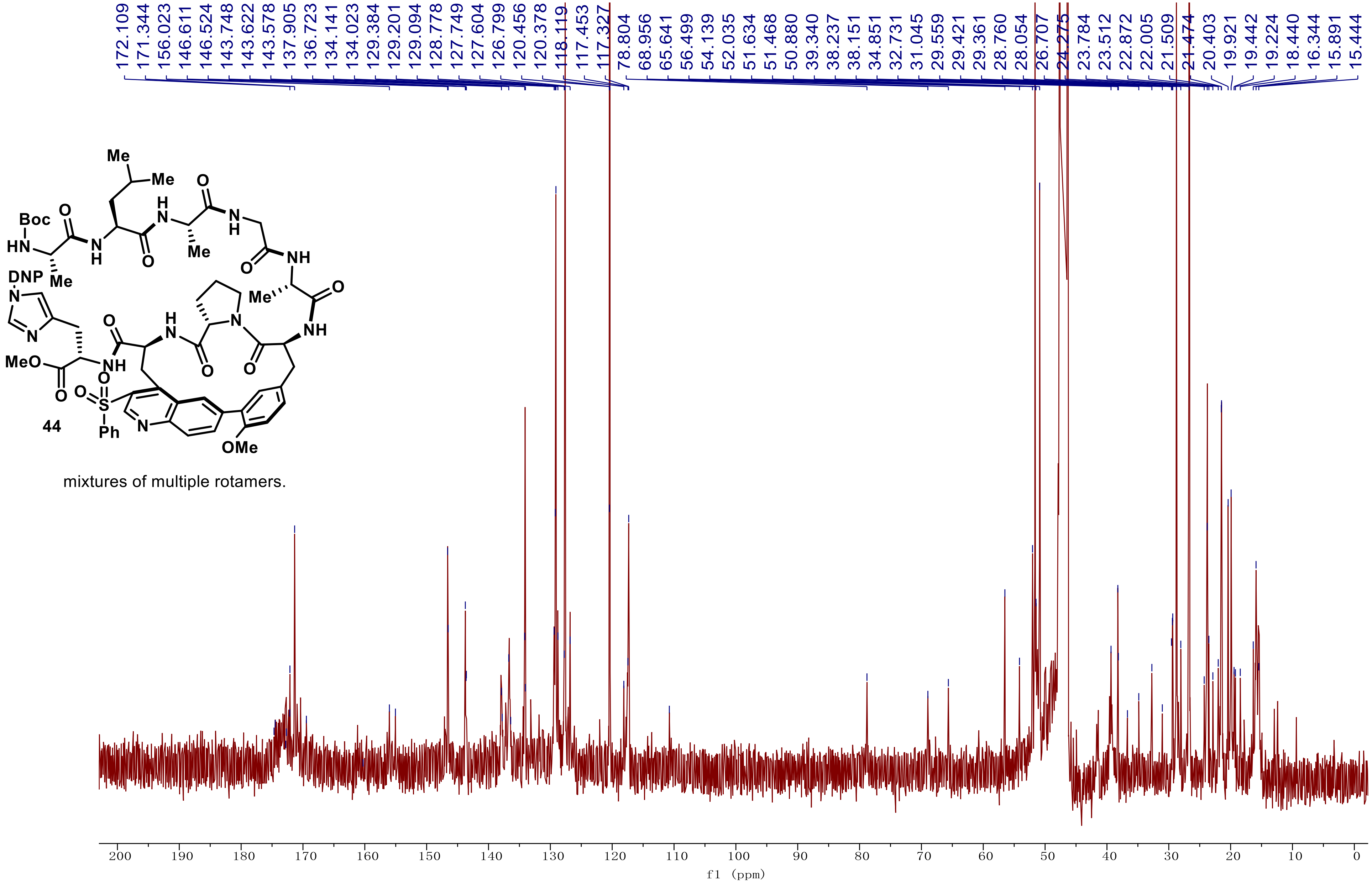

mixtures of multiple rotamers.

Compound 45 ^{1}HNMR (600 MHz, CD3OD)

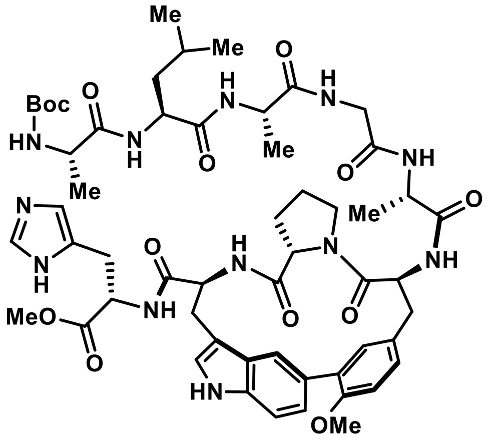

protected micitide 982 (**25**)

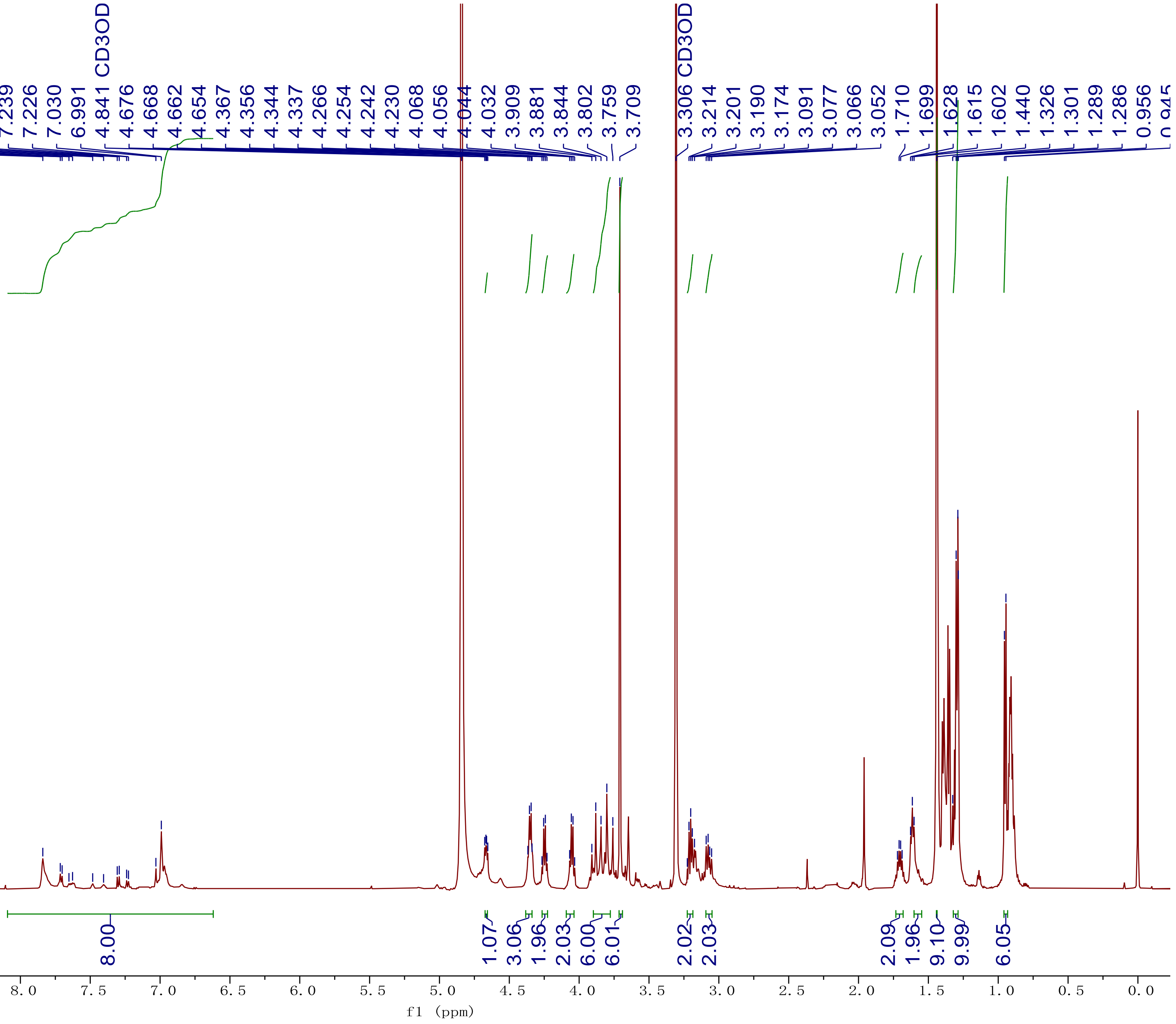

Compound 45 ^{13}CNMR (151 MHz, CD_3OD)

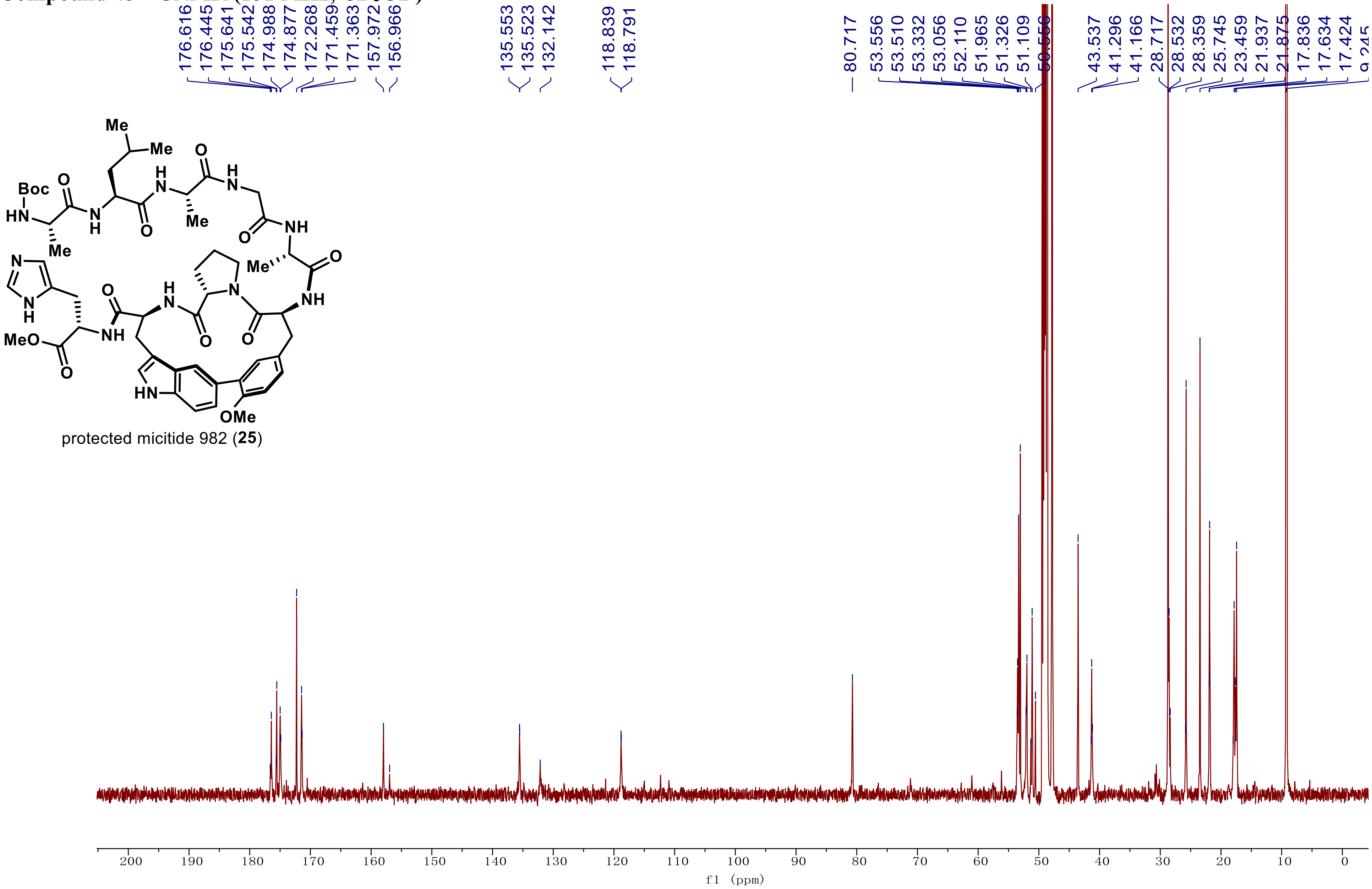

protected micitide 982 (**25**)

Compound 5 ^{1}HNMR (400 MHz, DMSO-*d6*)

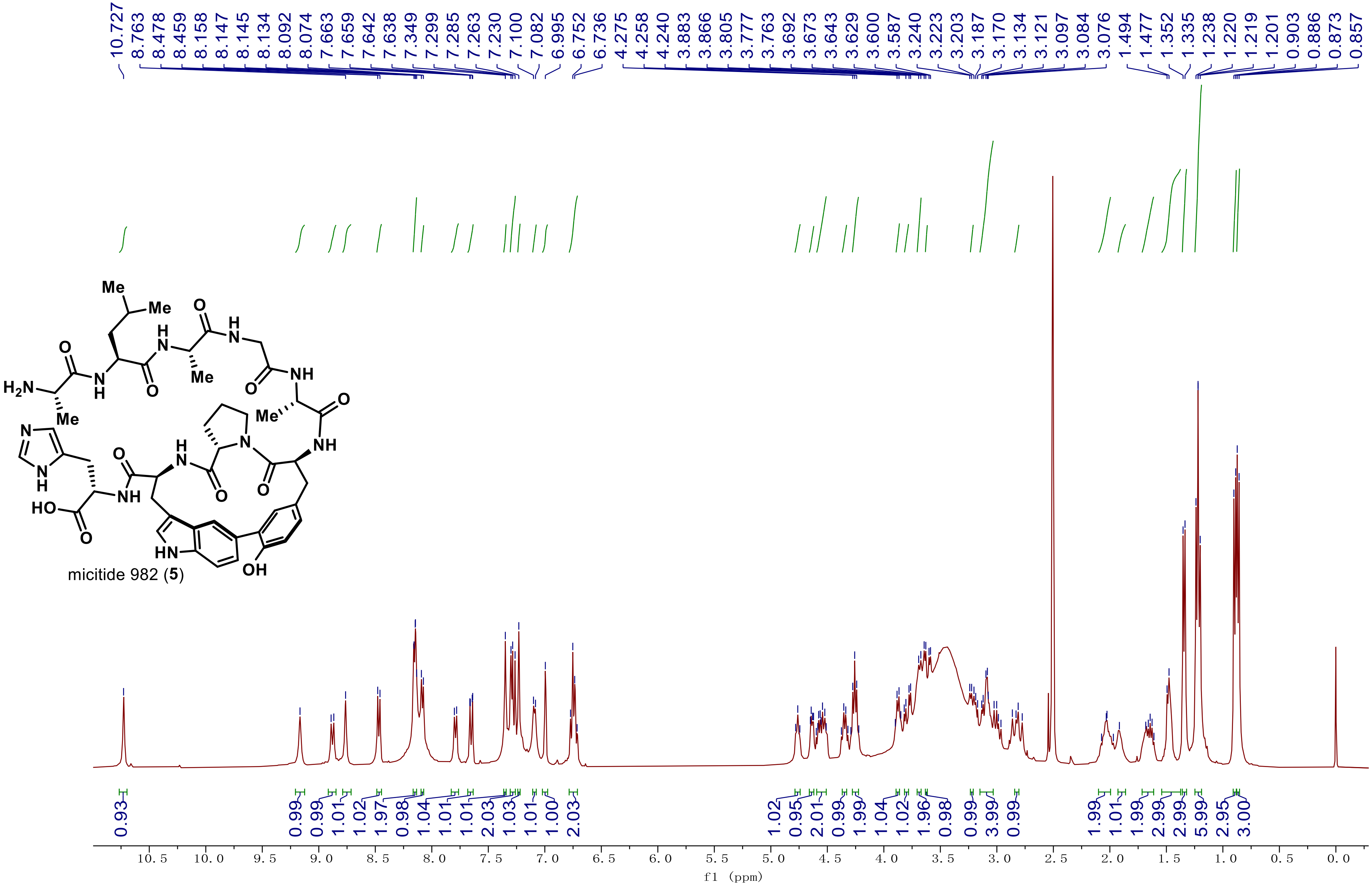

^{1}HNMR Comparison of Micitide 982 (1) [1, 2]

Reported[1]
in DMSO-*d6* (600 MHz)

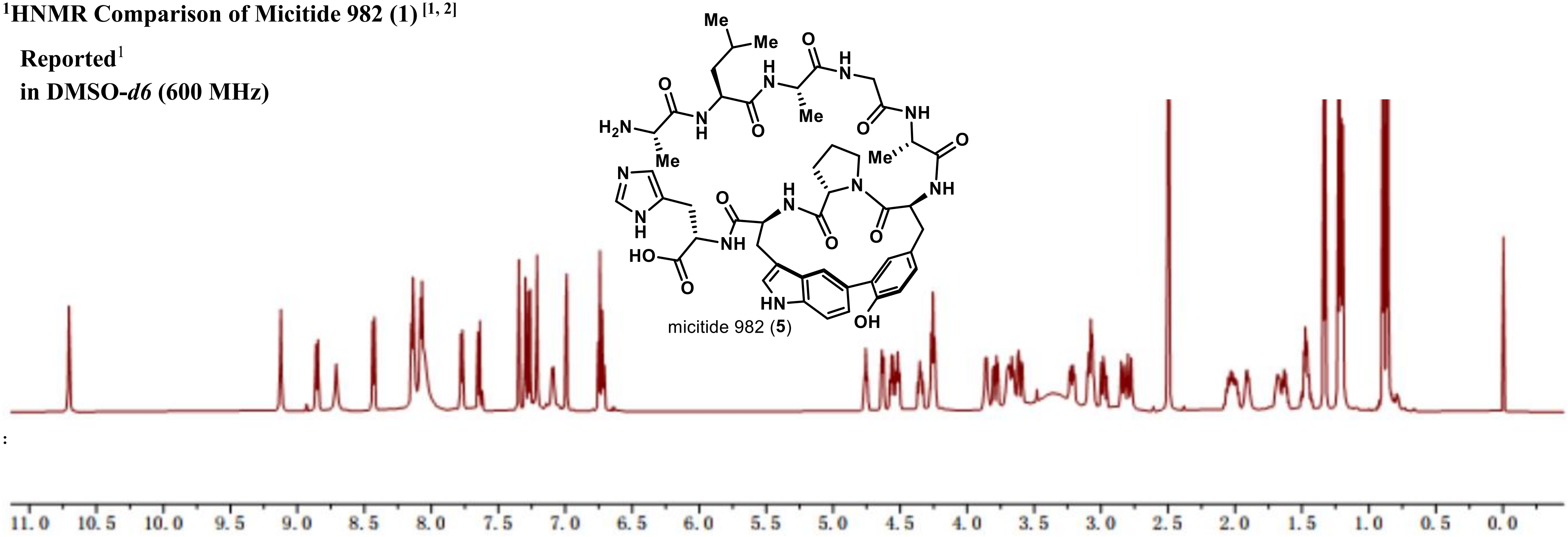

micitide 982 (5)

:

Synthetic (This work)
in DMSO-*d6* (400 MHz)

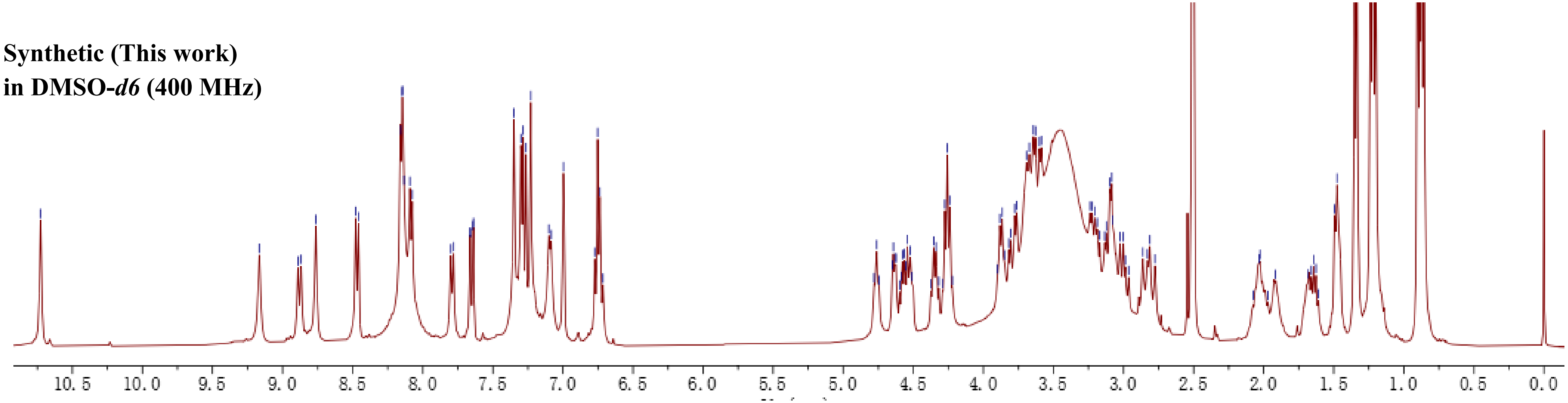

Compound 5 ^{13}CNMR (151 MHz, DMSO-*d6*)

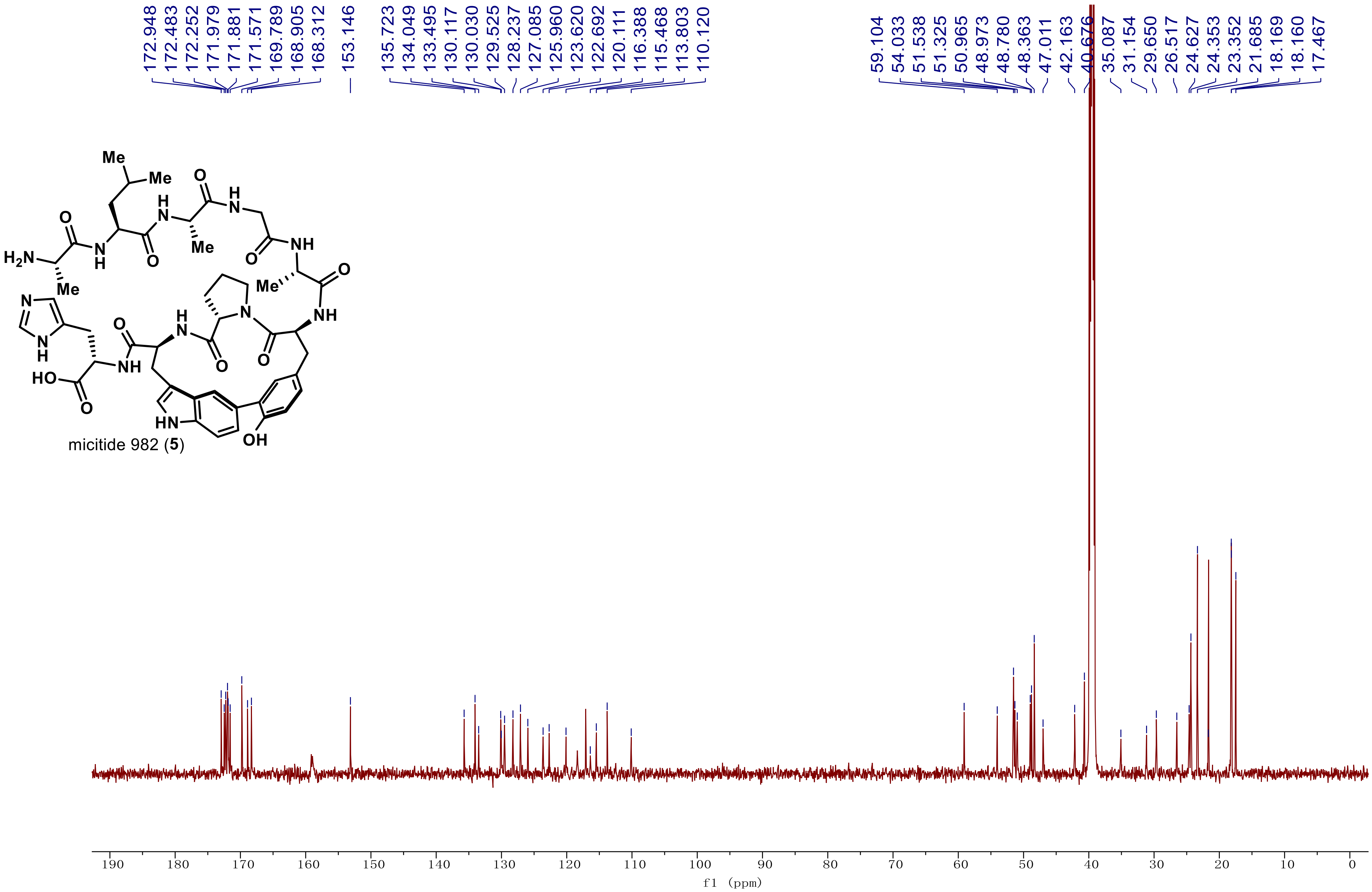

^{13}CNMR Comparison of Micitide 982 (5) [1,2]

Reported[1]
in DMSO-*d6* (151 MHz)

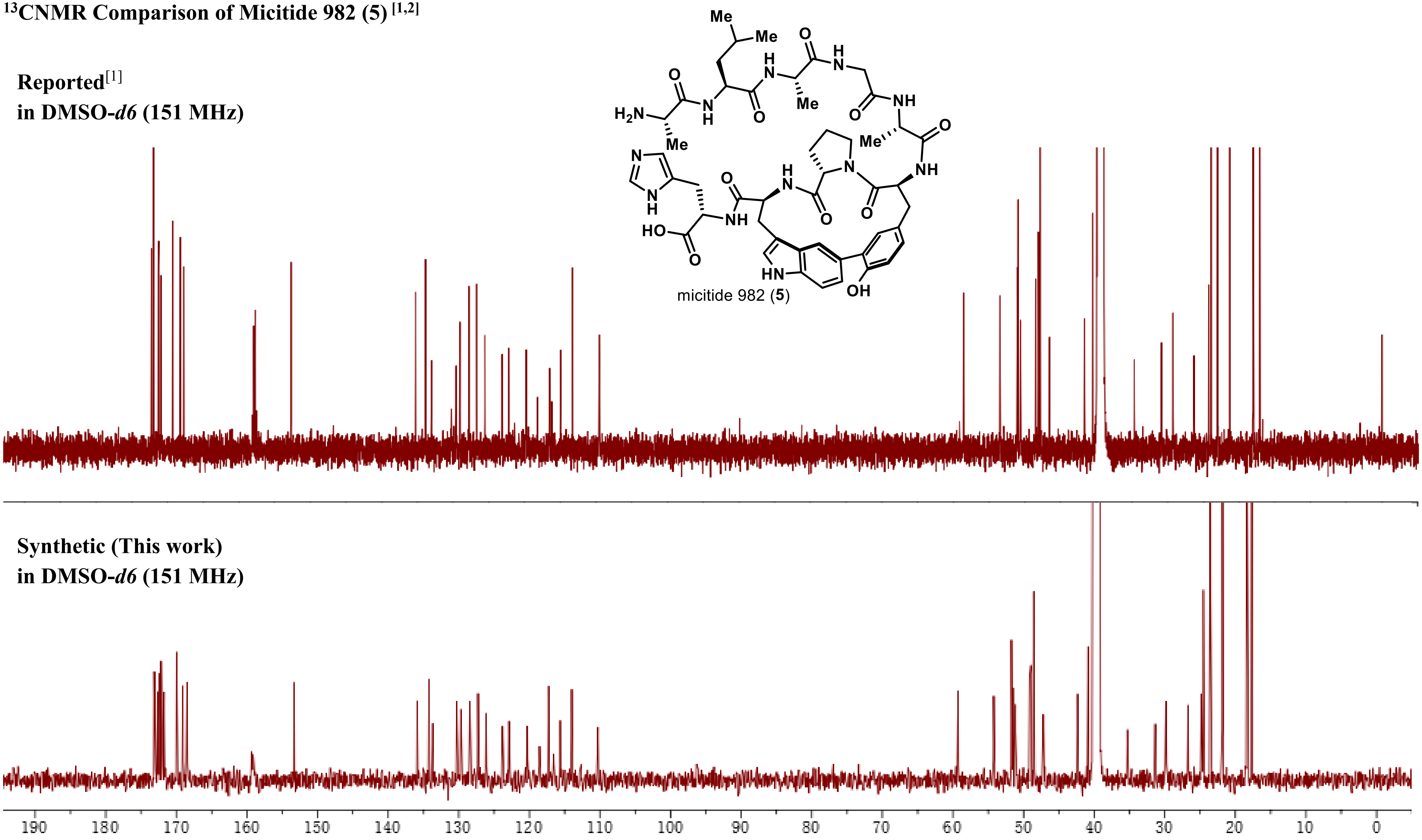

Synthetic (This work)
in DMSO-*d6* (151 MHz)

Compound S9 ^{1}H NMR (600 MHz, CD_3OD)

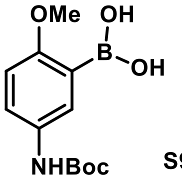

S9

mixture of rotamers
A:B = 3:2

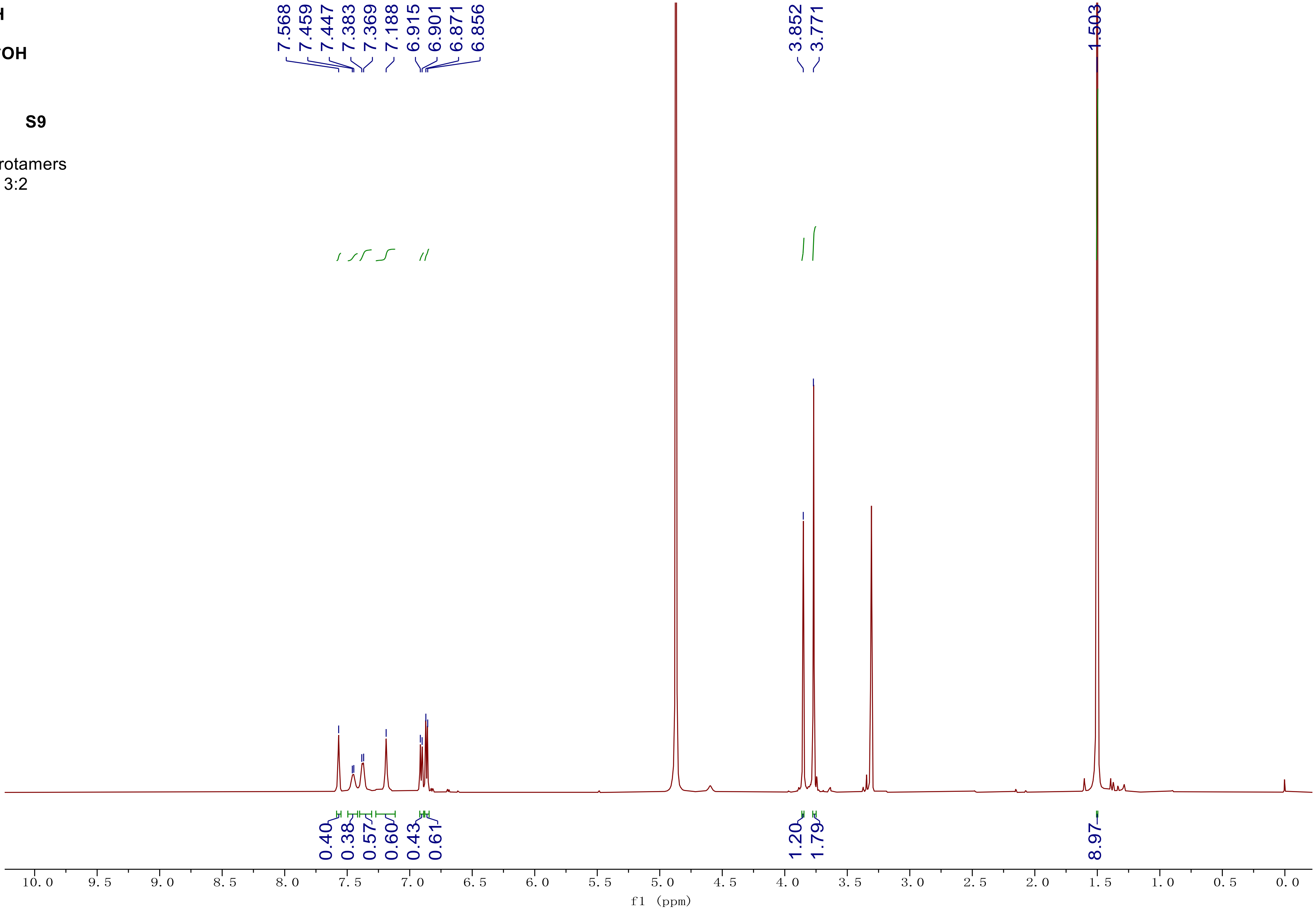

Compound S3 ^{13}C NMR (151 MHz, CD_3OD)

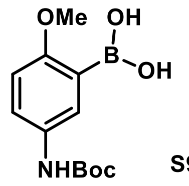

S9

mixture of rotamers
A:B = 3:2

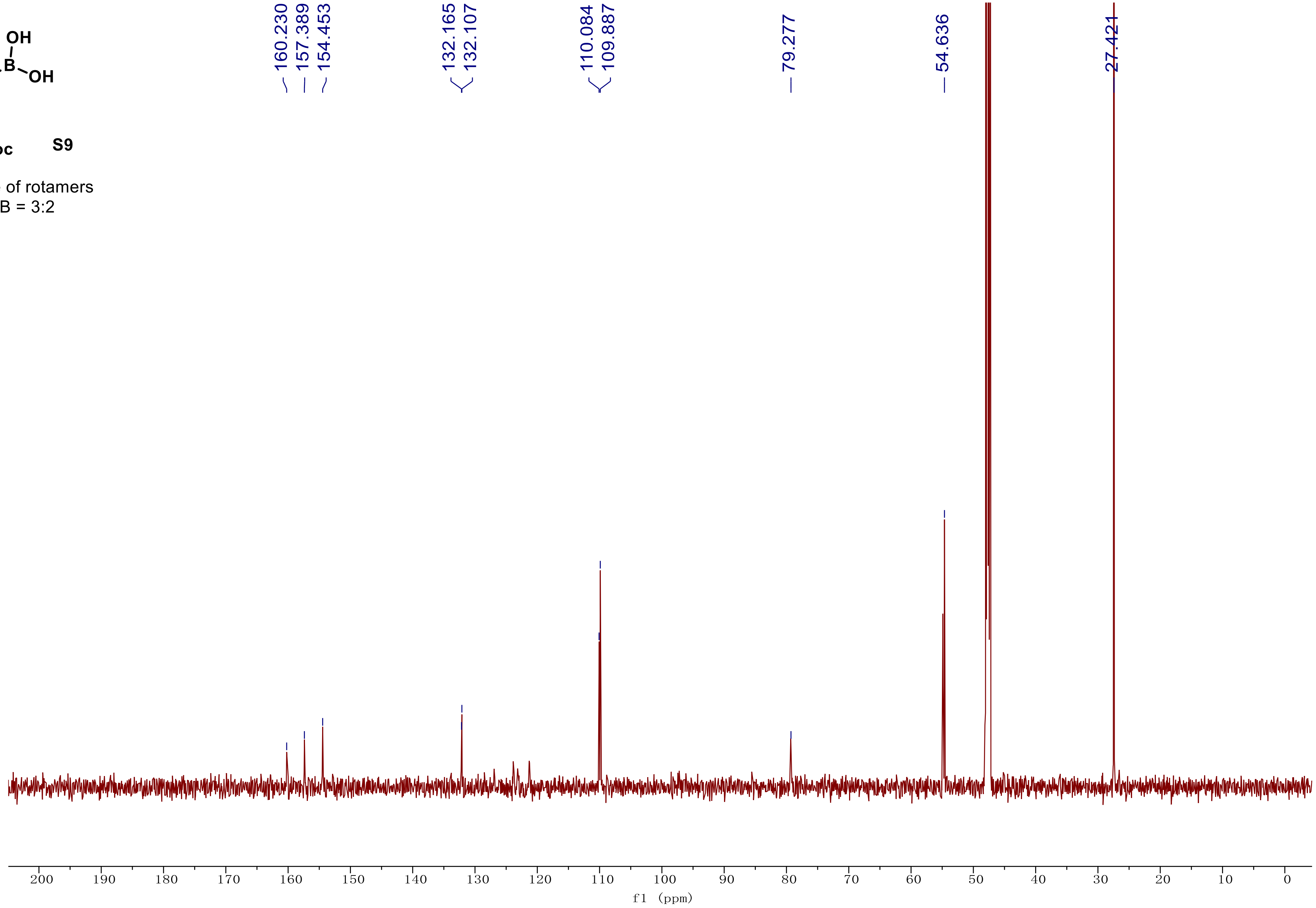

Compound S11 ^{1}H NMR (400 MHz, $CDCl_3$)

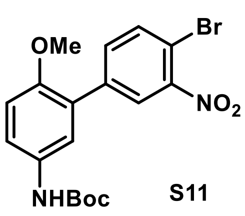

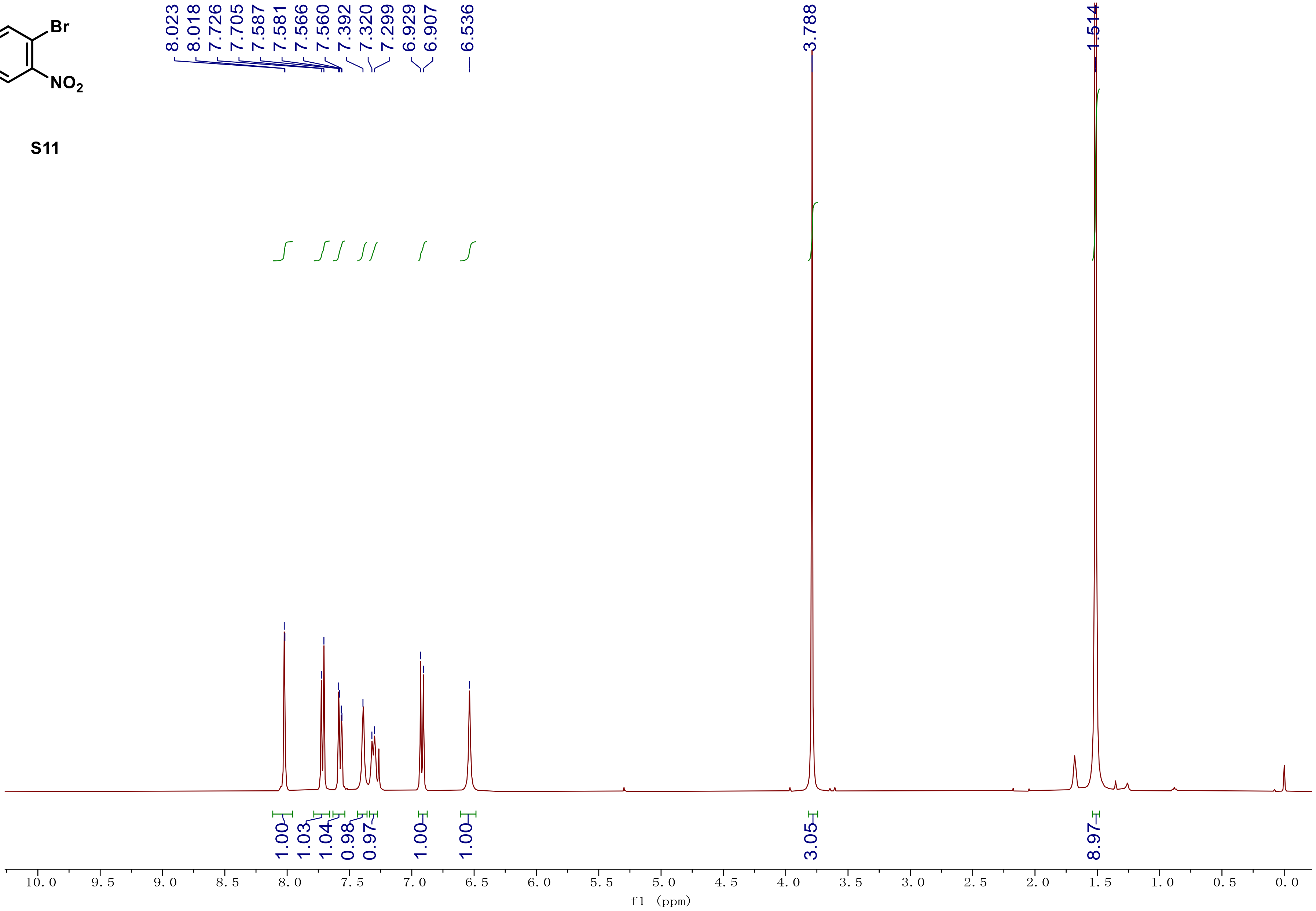

Compound S11 ^{13}C NMR (101 MHz, $CDCl_3$)

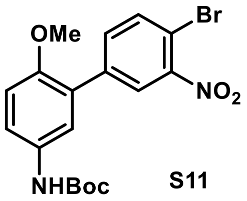

S11

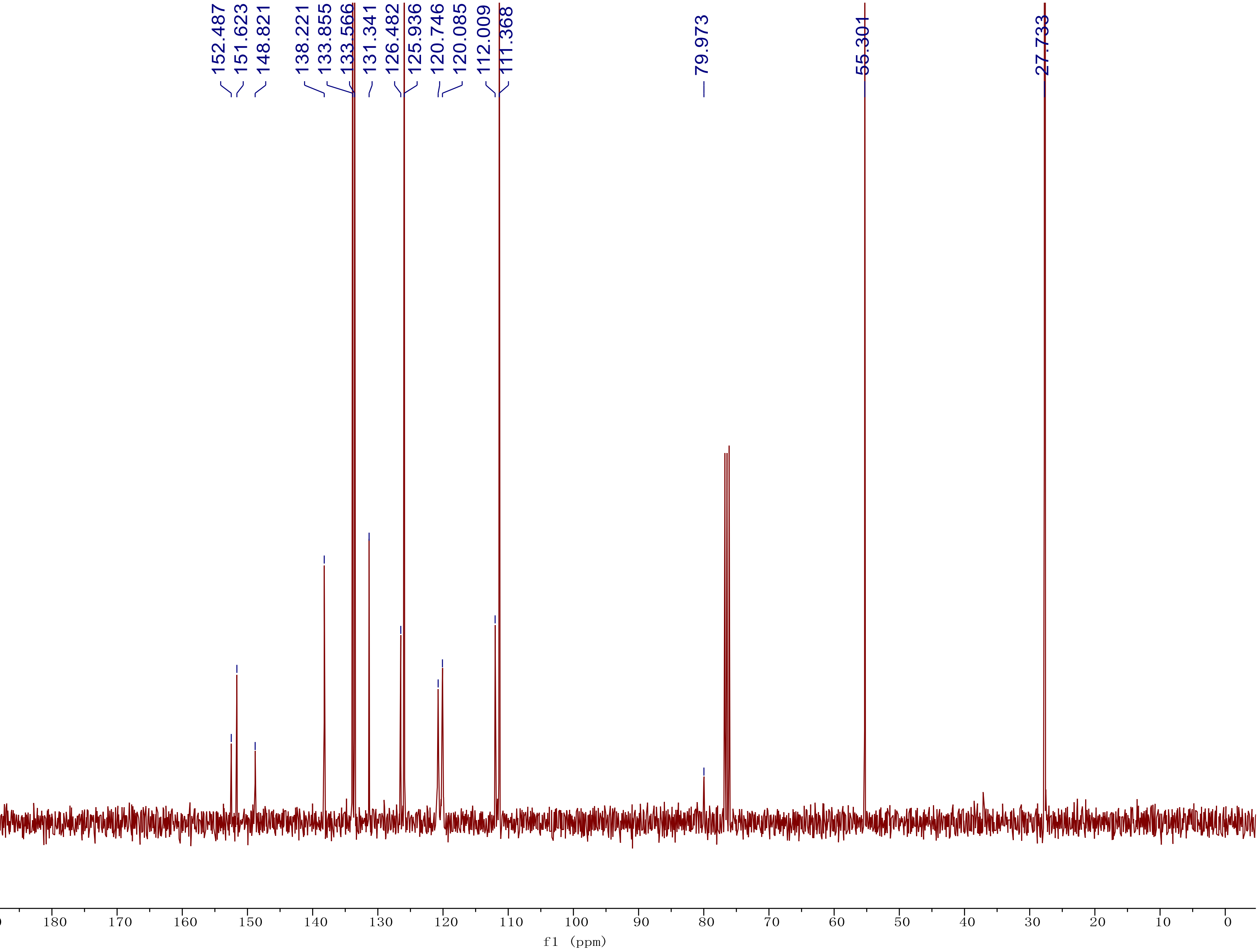

Compound 23 ^{1}H NMR (400 MHz, $CDCl_3$)

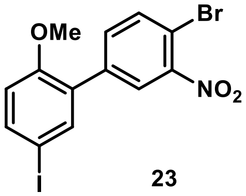

23

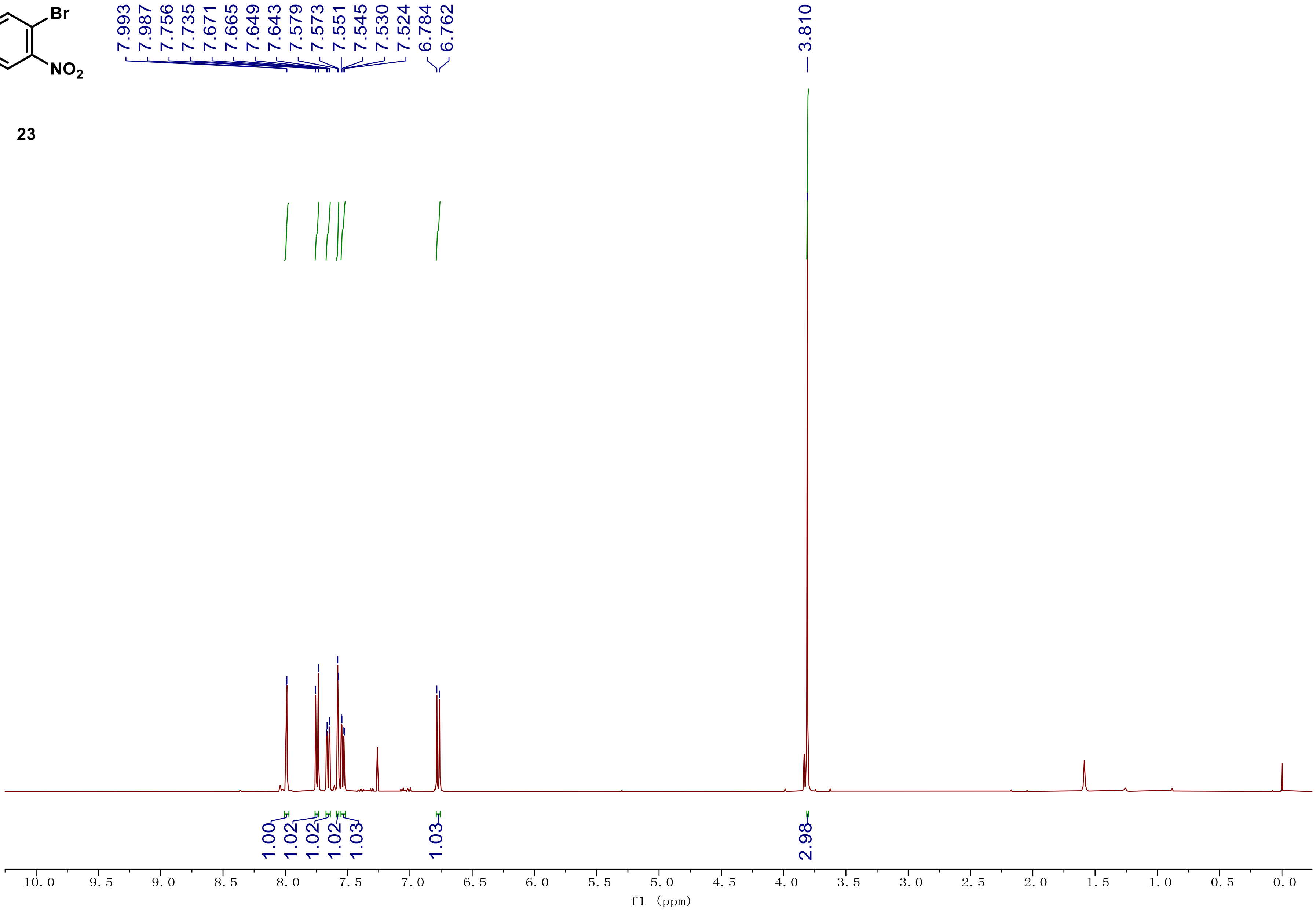

Compound 23 ^{13}C NMR (101 MHz, $CDCl_3$)

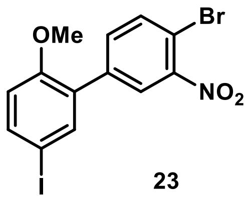

23

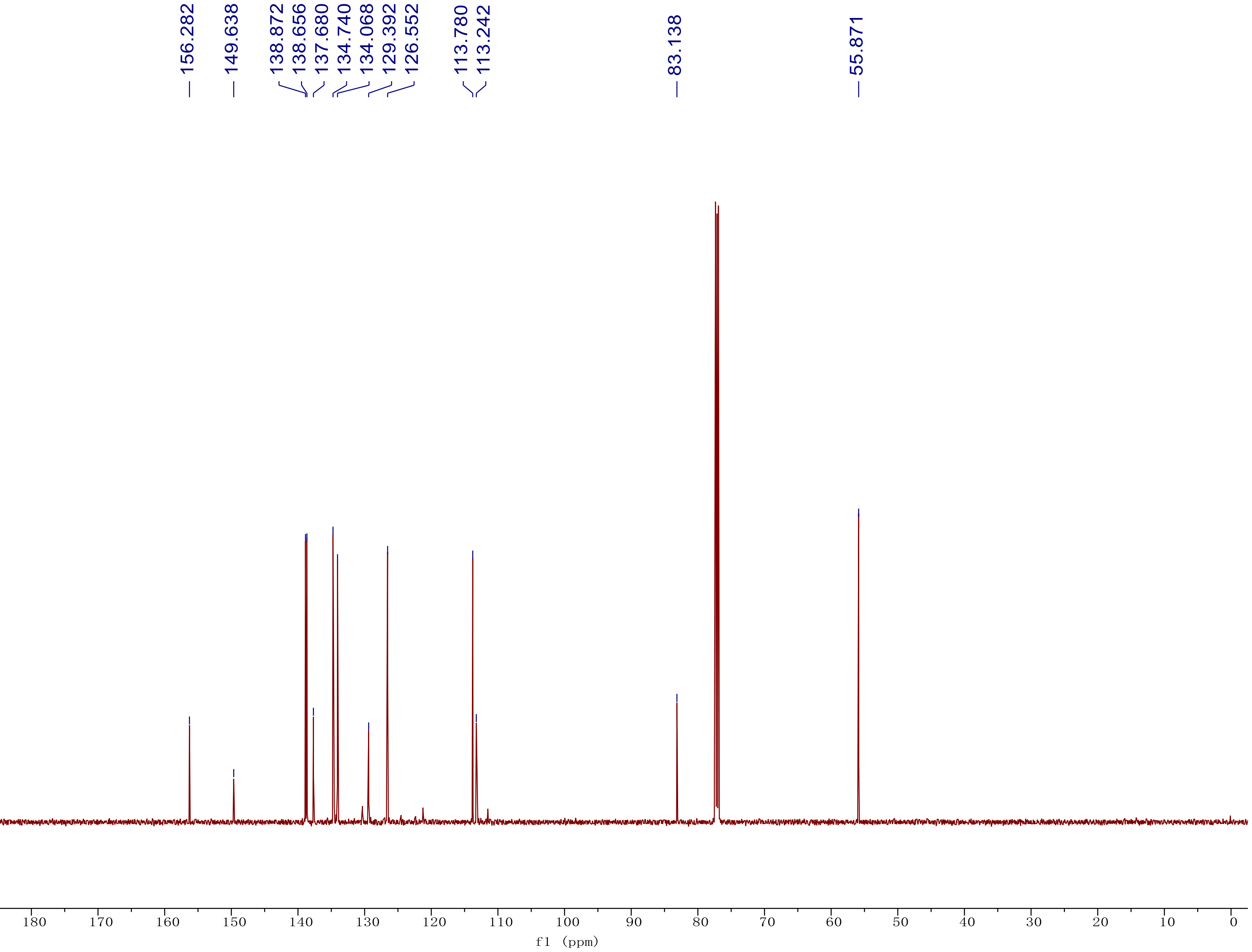

Compound S14 ^{1}H NMR (400 MHz, $CDCl_3$)

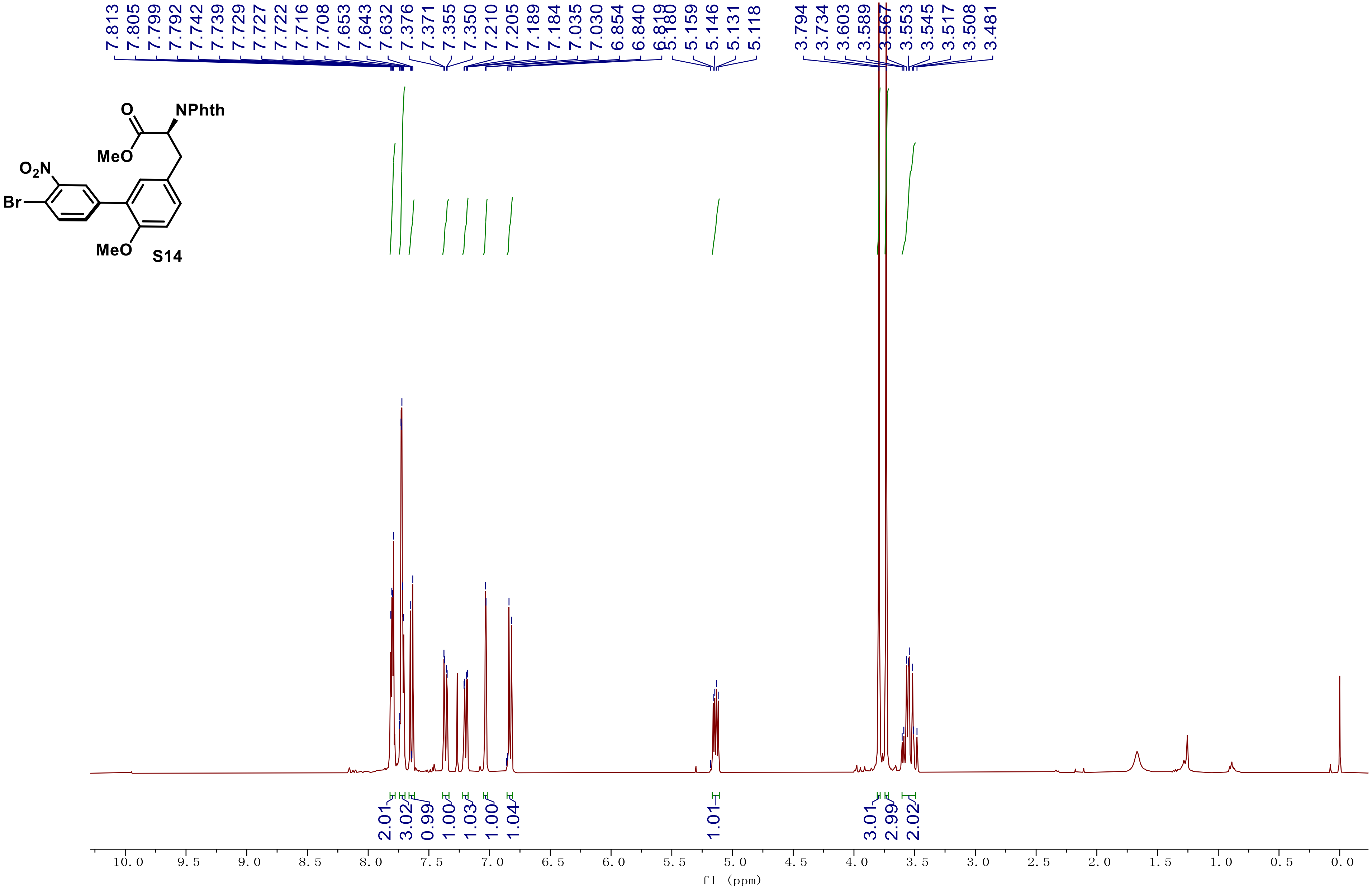

Compound S14 ^{13}C NMR (101 MHz, $CDCl_3$)

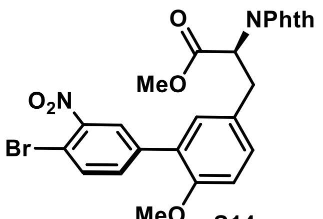

S14

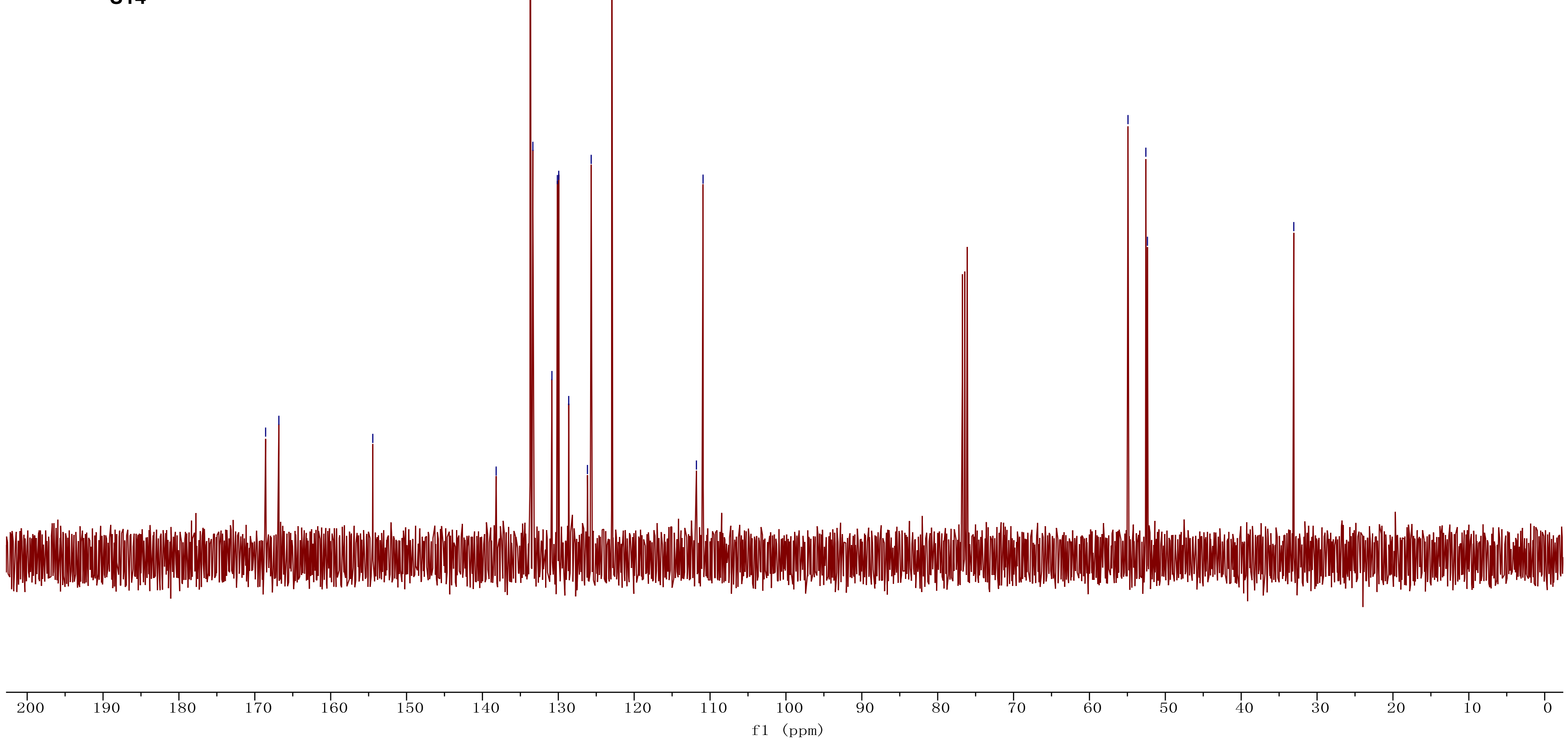

Compound 49 (400 MHz, CD_3OD, 60 °C)

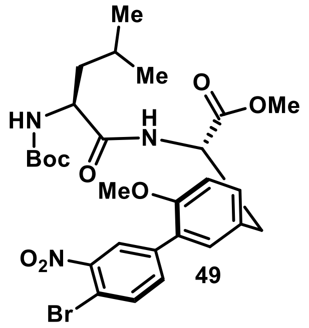

mixture of multiple rotamers
signals of the major isomer were reported

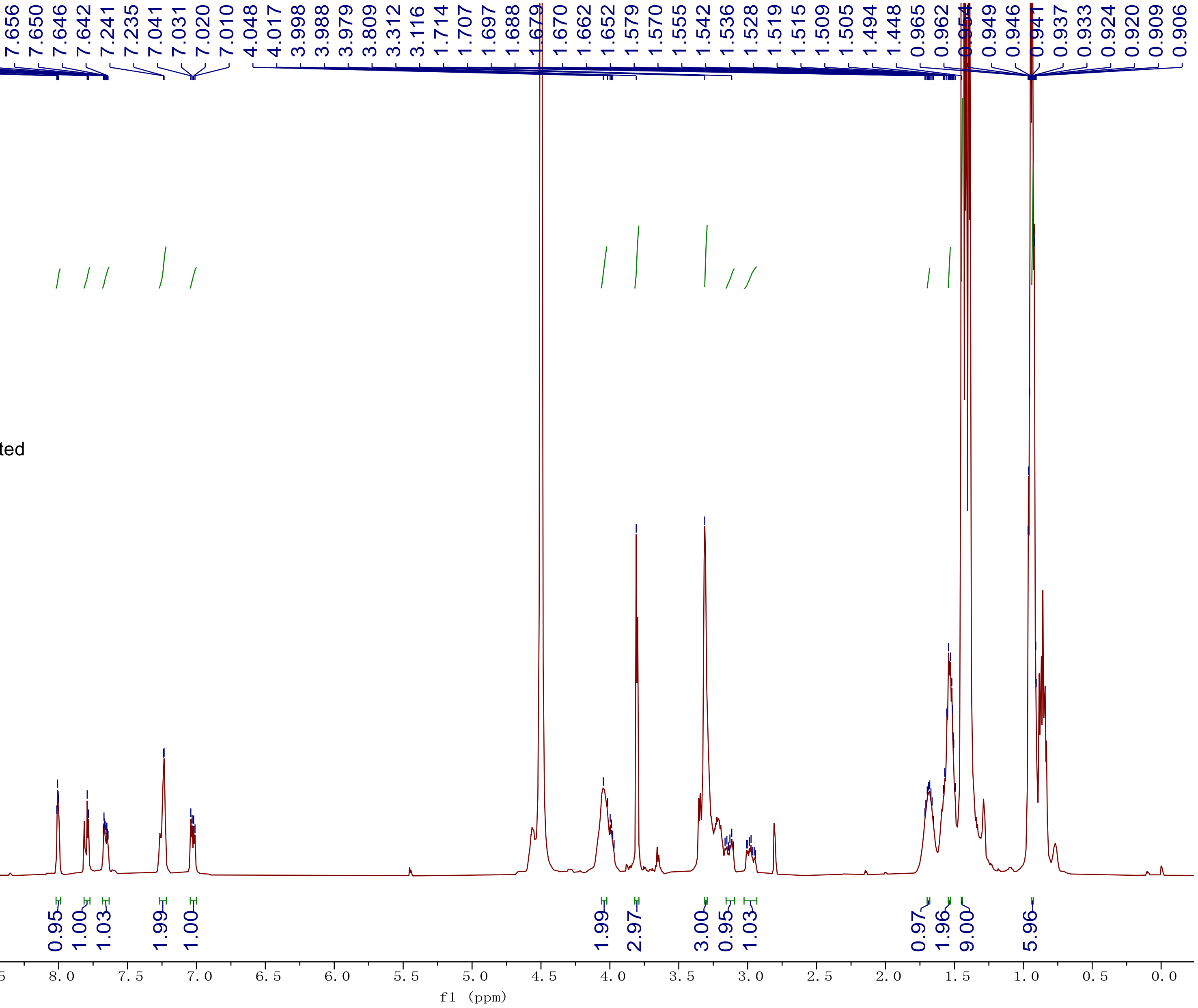

Compound 49 ^{13}C NMR (101 MHz, CD_3OD, 60 °C)

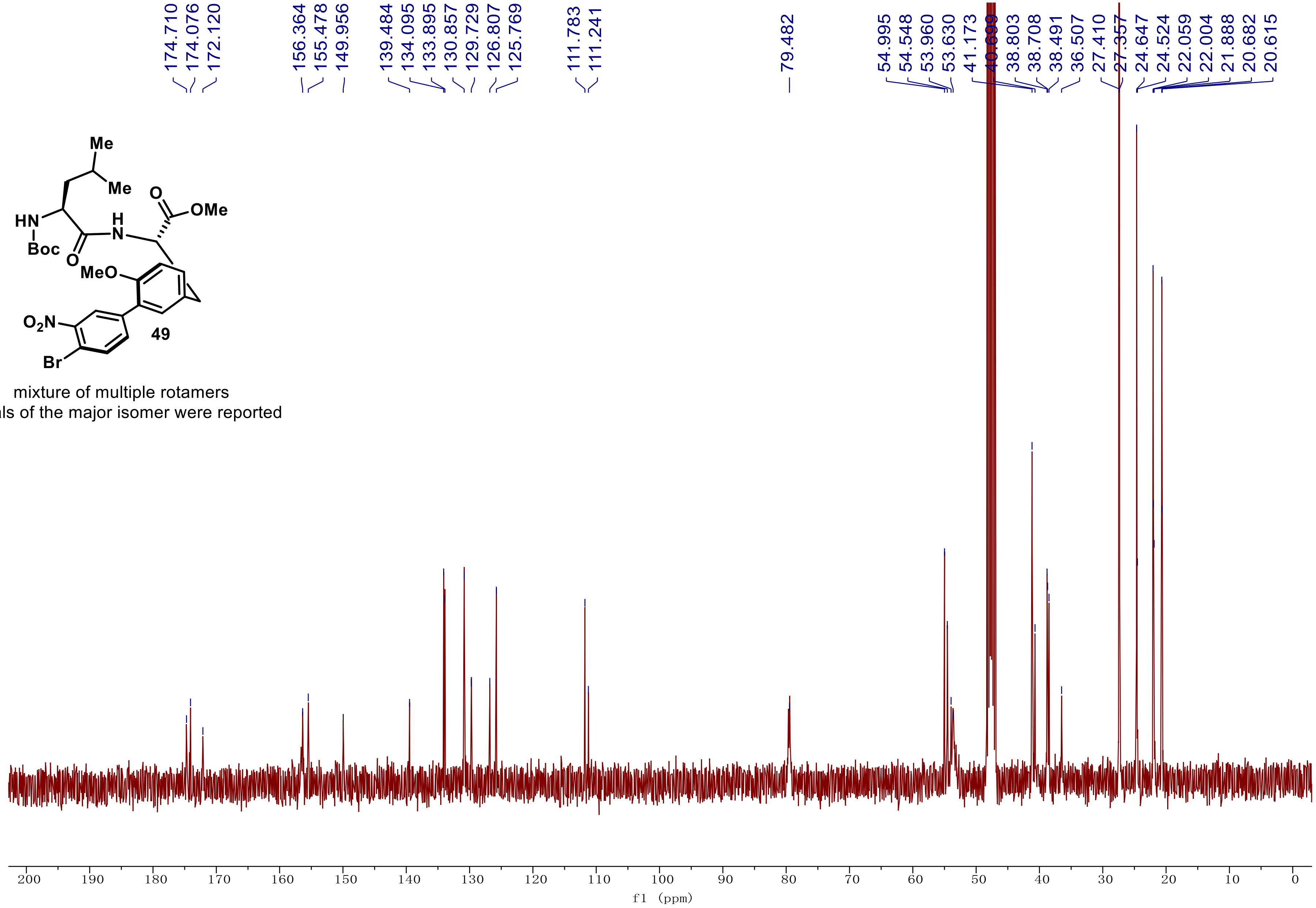

mixture of multiple rotamers
signals of the major isomer were reported

Compound S15 (600 MHz, CD3OD, 60 °C)

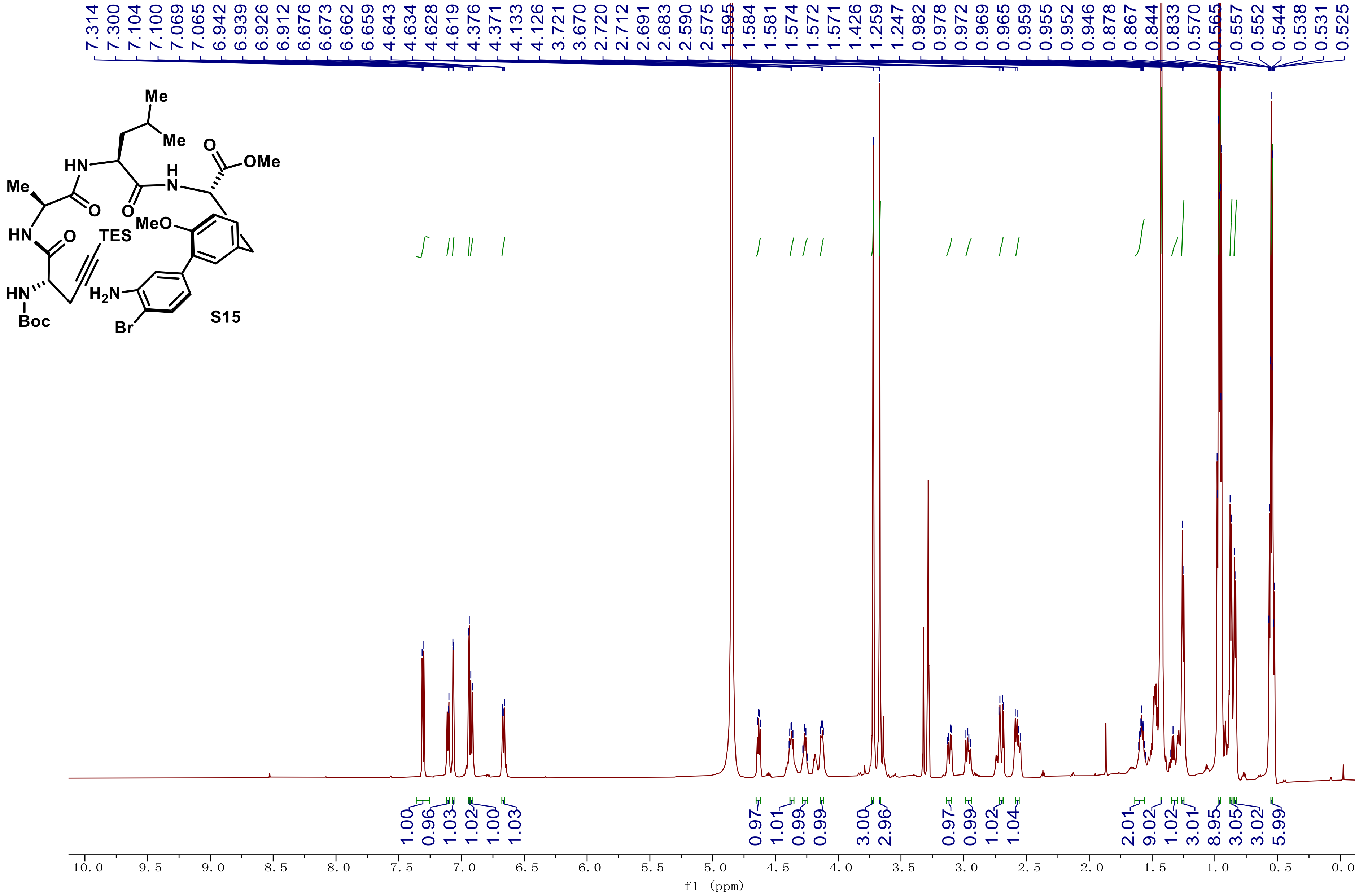

Compound S15 ^{13}C NMR (101 MHz, CD_3OD, 60 °C)

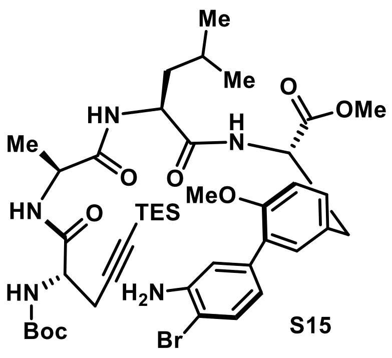

S15

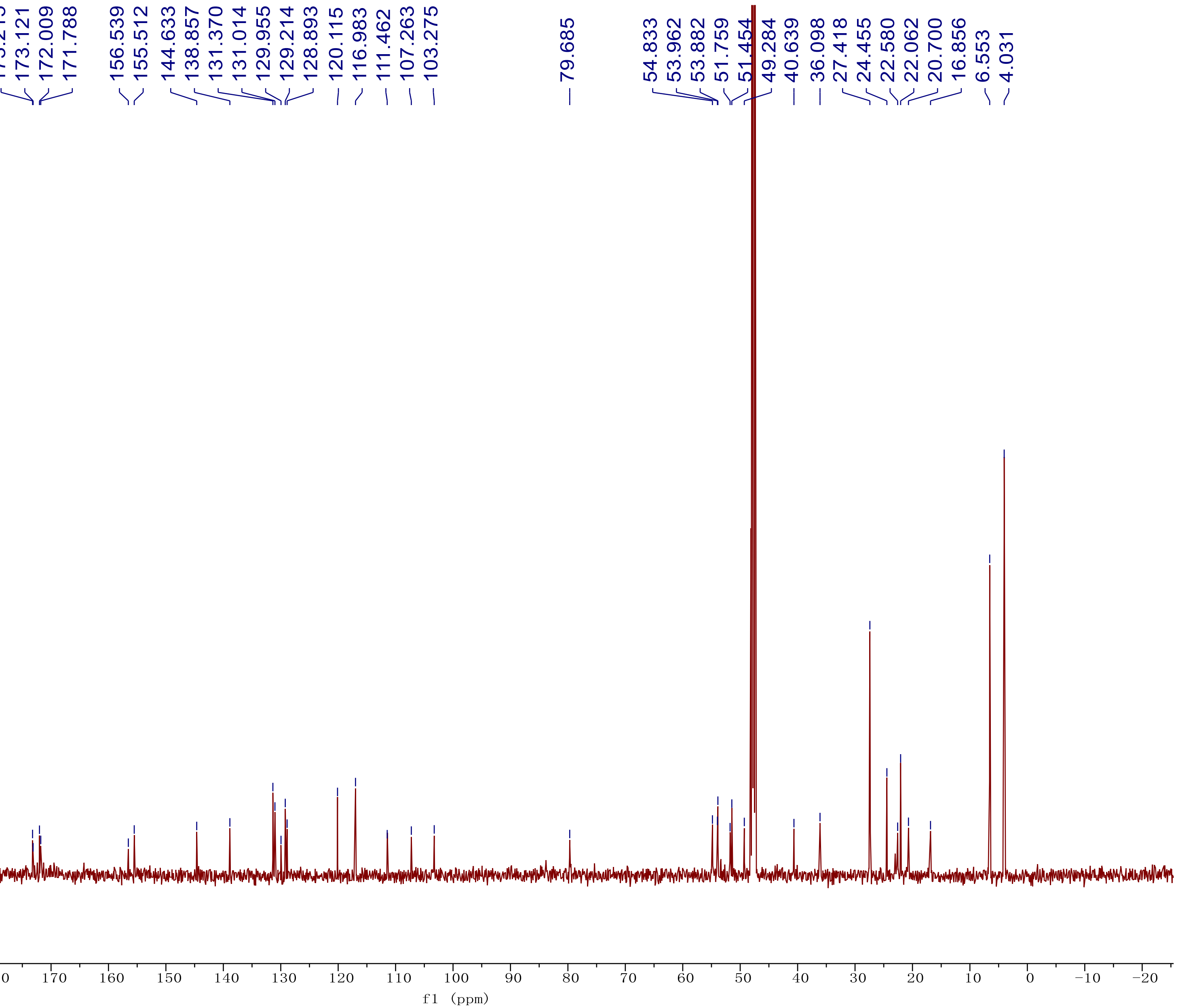

Compound 54 (400 MHz, CDCl$_3$)

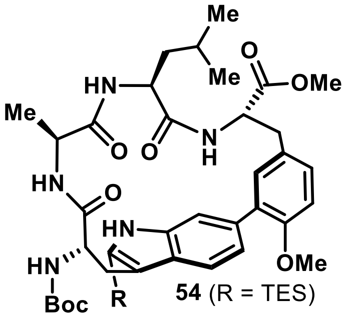

54 (R = TES)

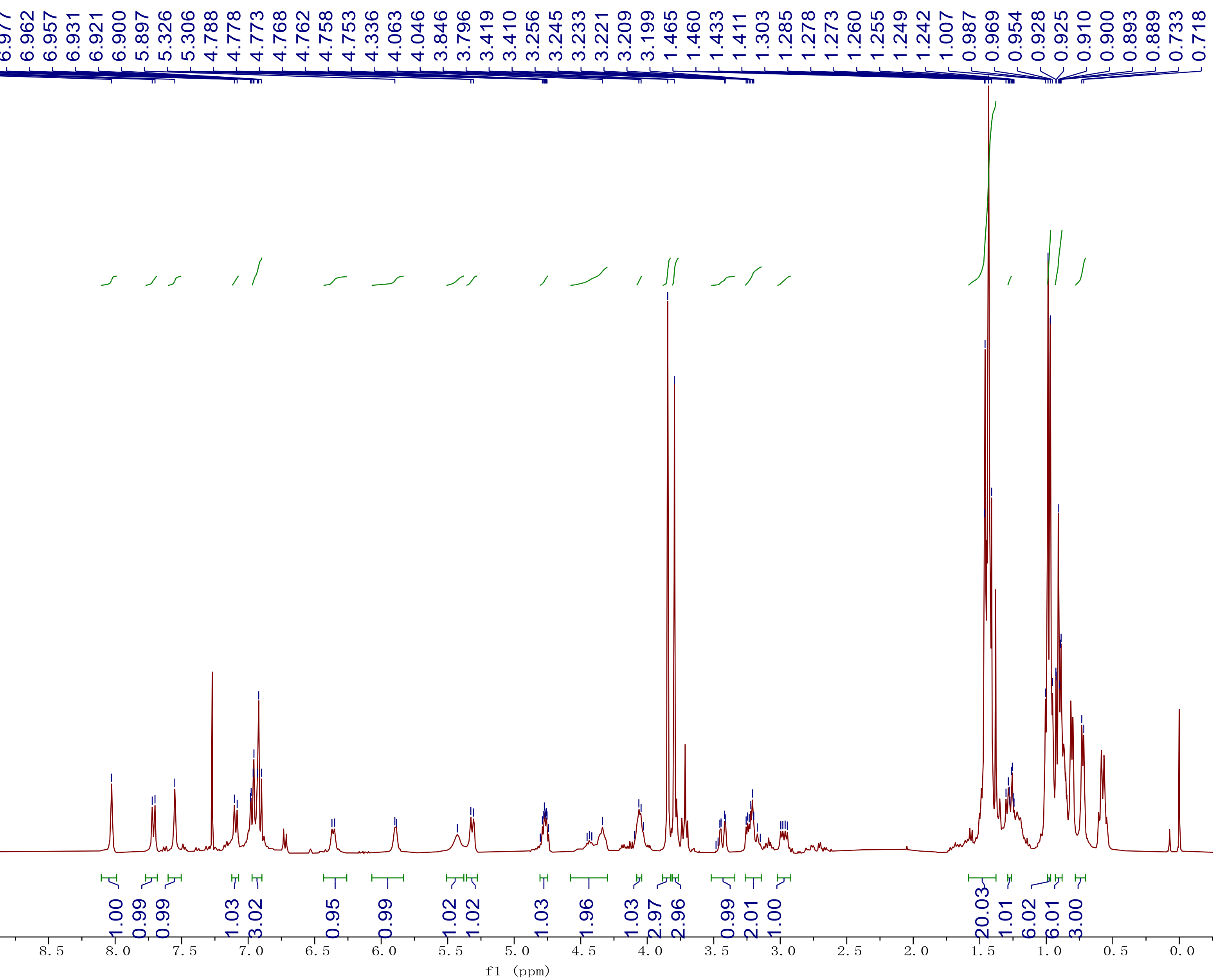

Compound 54 ^{13}C NMR (101 MHz, $CDCl_3$)

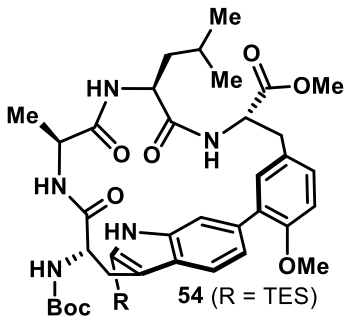

54 (R = TES)

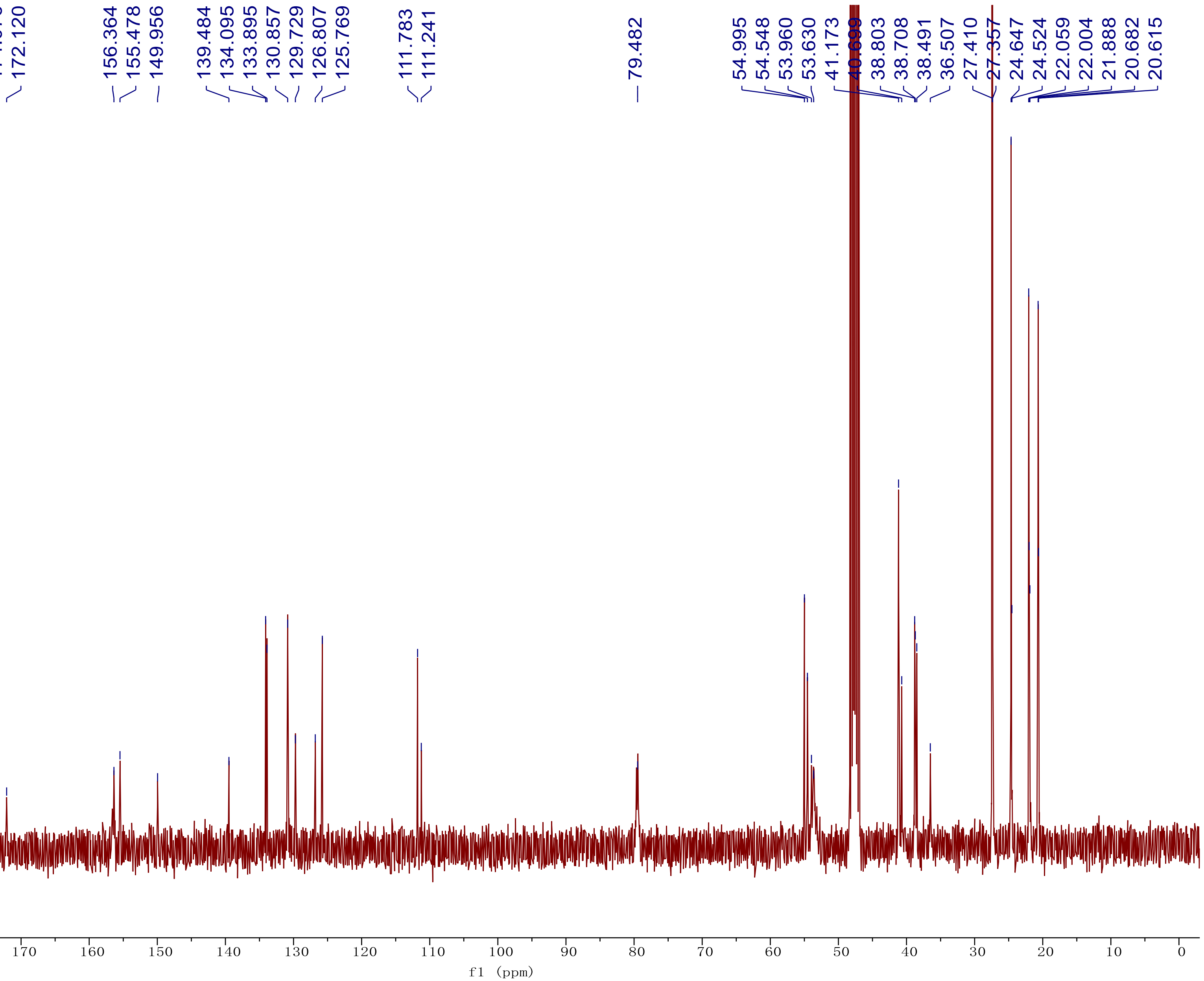

Compound 9 ¹H NMR (600 MHz, DMSO-*d6*)

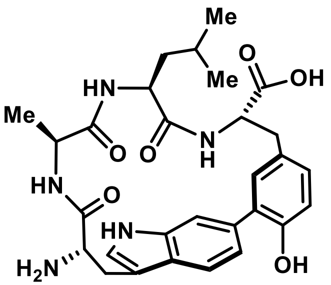

*R*conf-scabrirubin CB-4 (**9**)

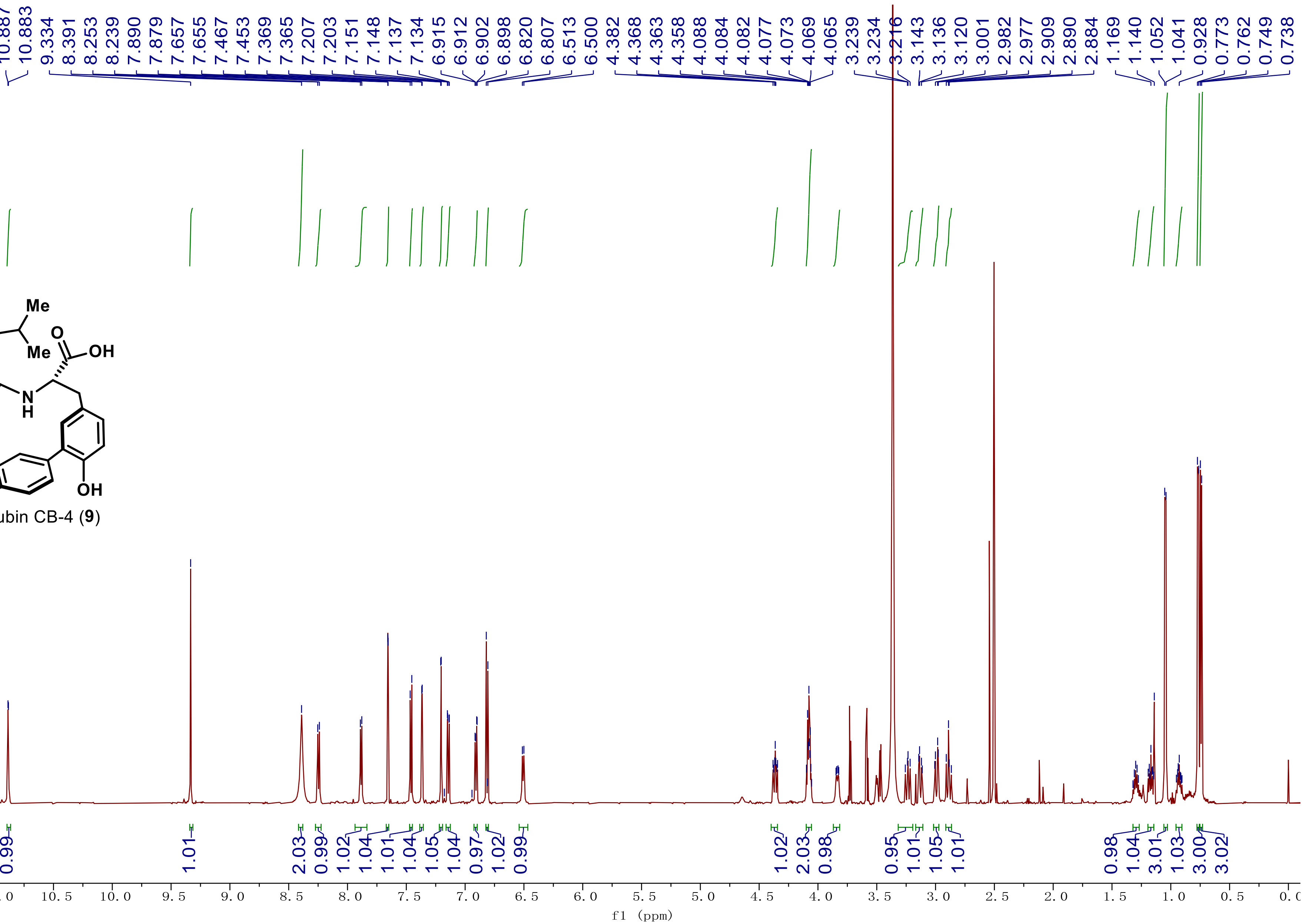

Compound 9 ^{13}C NMR (151 MHz, DMSO-d_6)

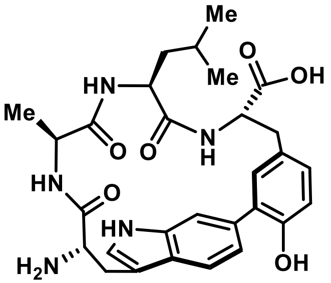

*R*conf-scabrirubin CB-4 (**9**)

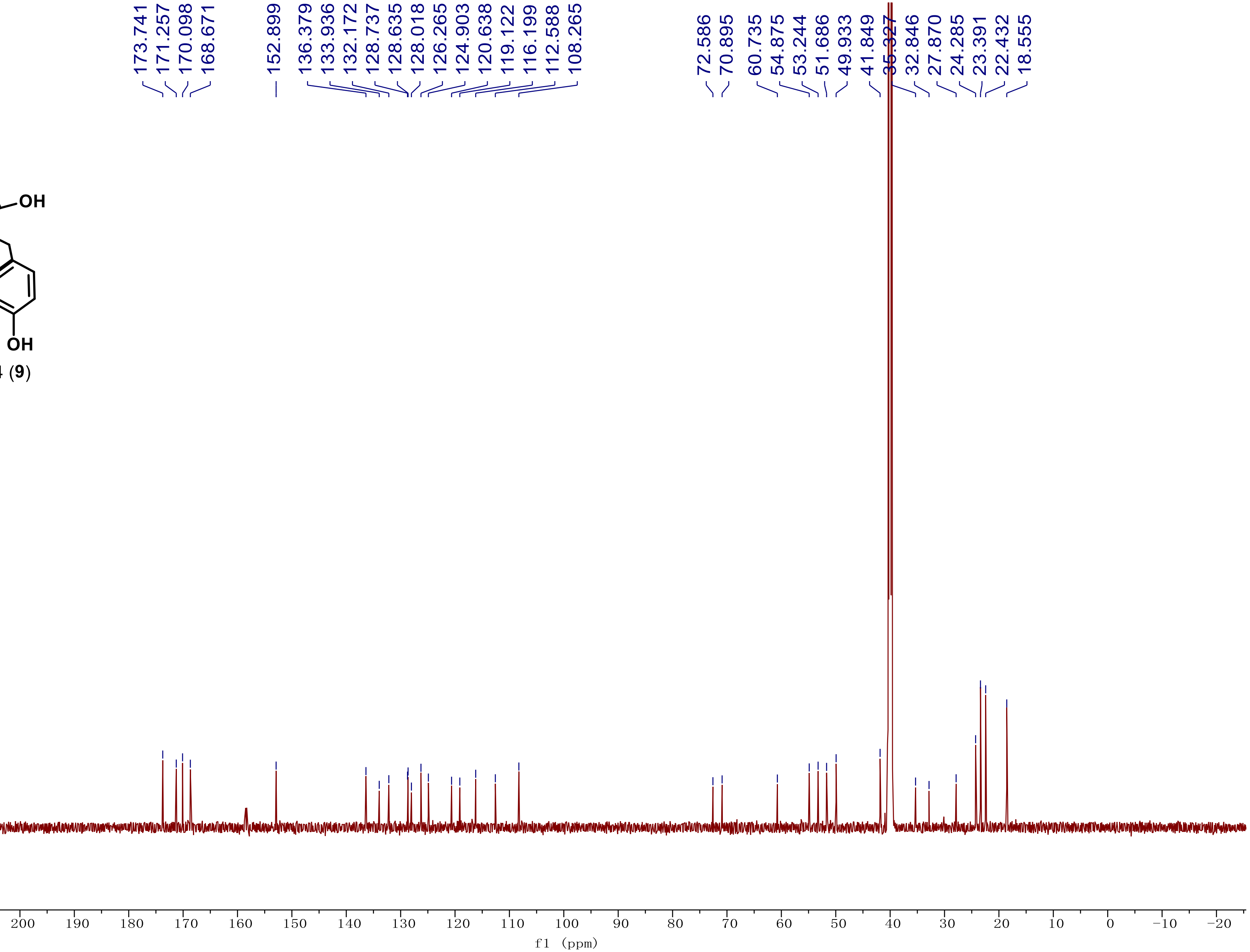

Compound 9 ^1H-^1H COSY NMR (400 MHz, DMSO-d_6)

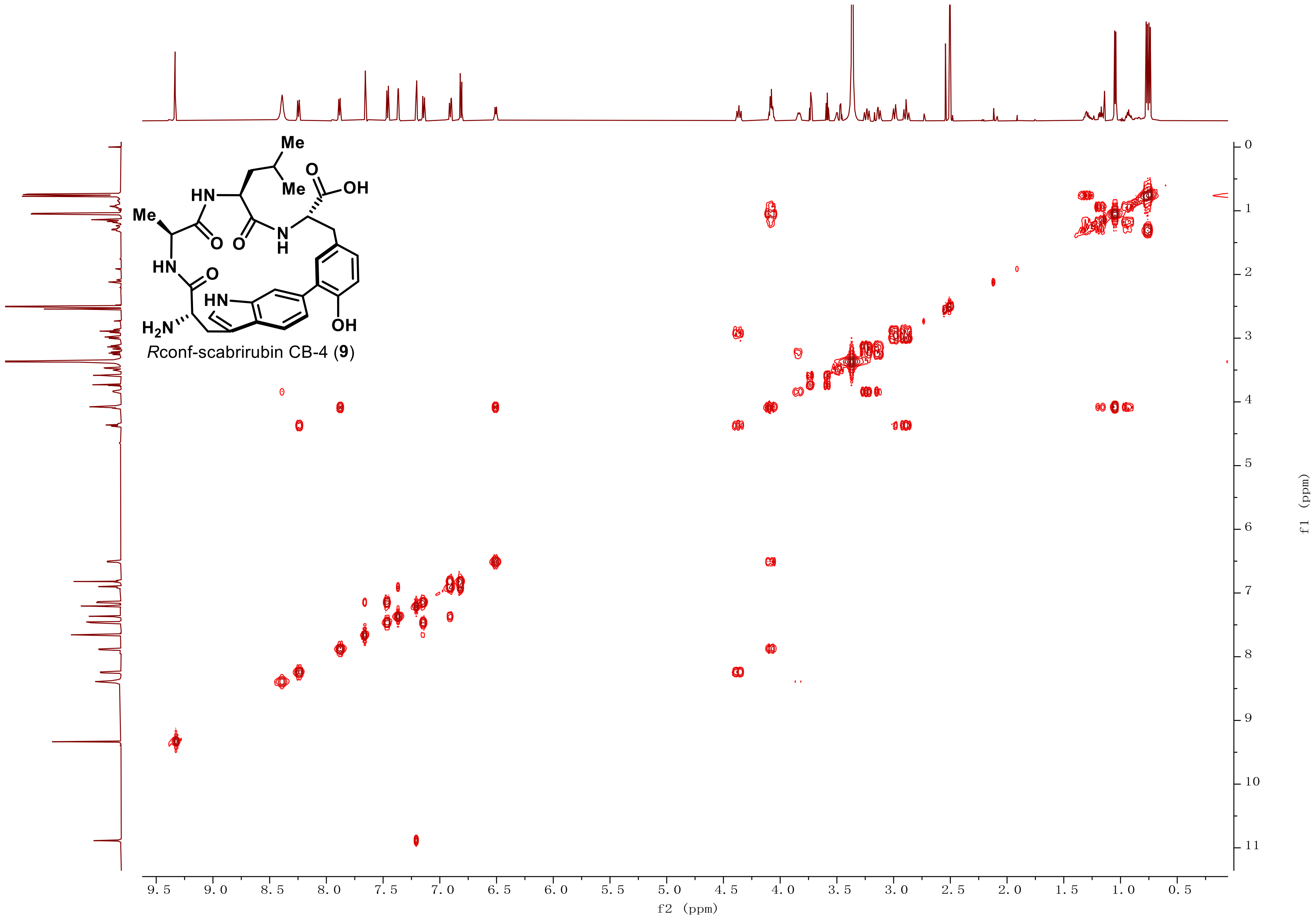

Compound 9 ^{1}H-^{13}C HSQC NMR (600 MHz, DMSO-d_6)

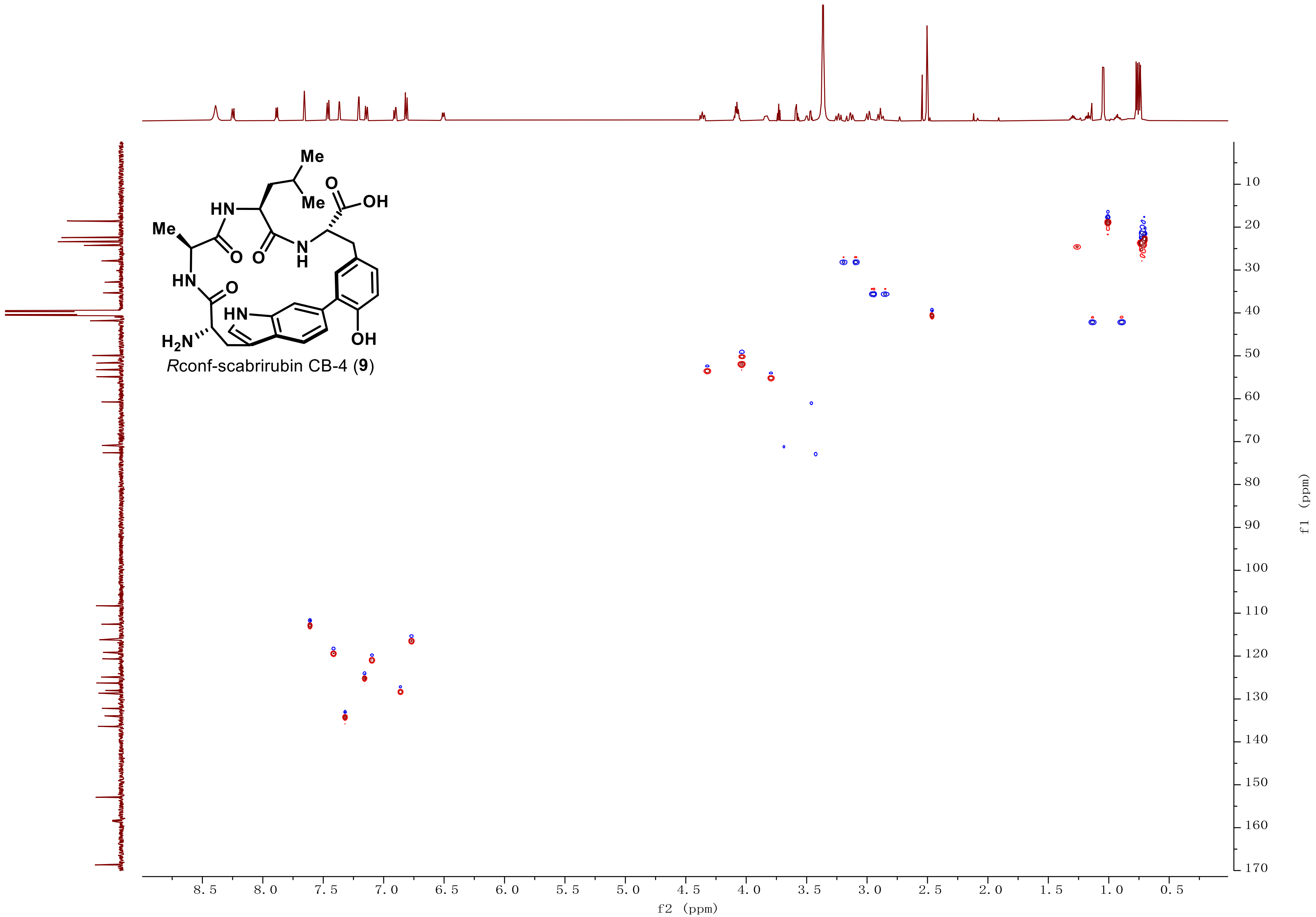

Compound 9 ^{1}H-^{13}C HMBC NMR (600 MHz, DMSO-d_6)

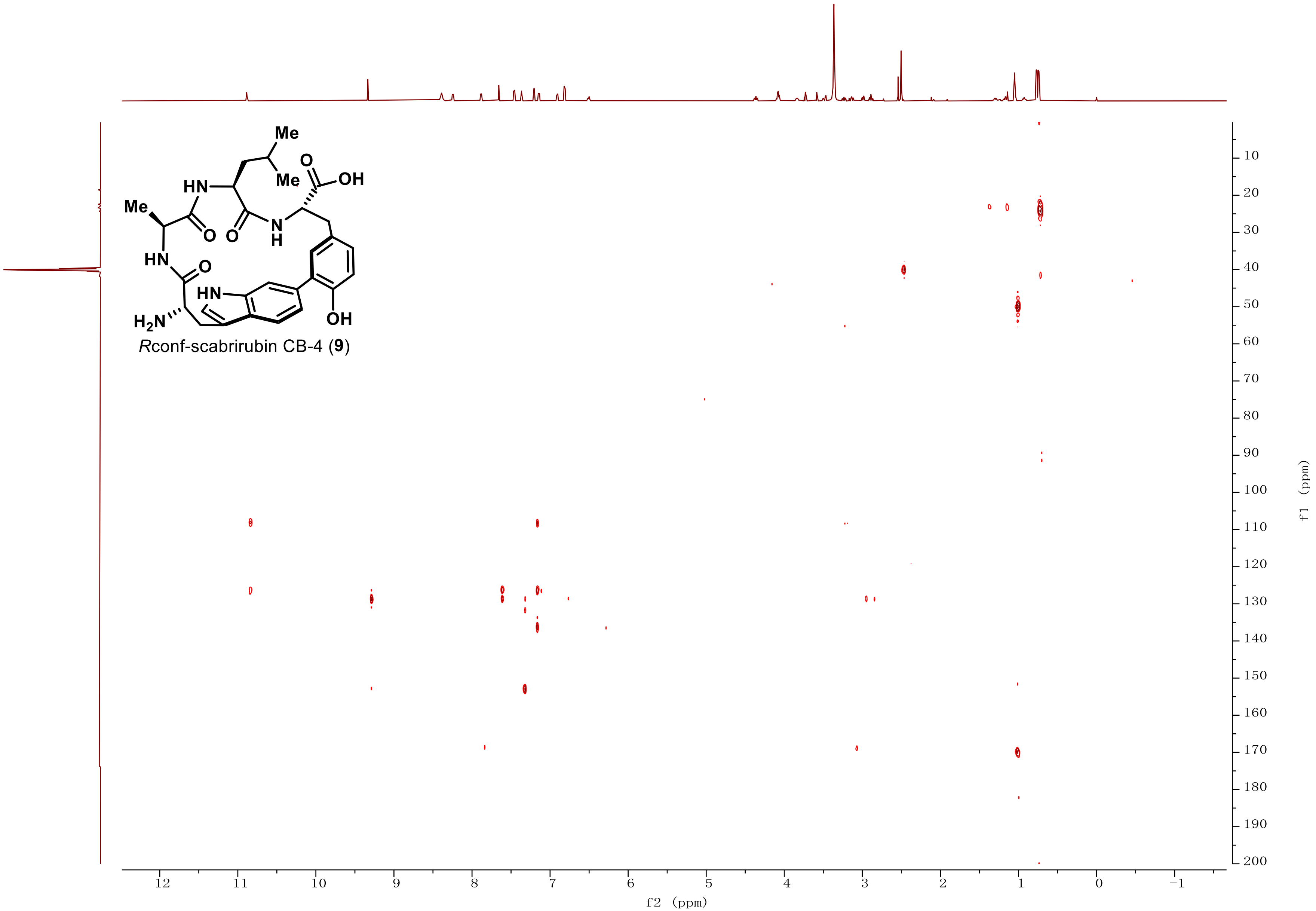

Compound 9 ^1H-^1H ROESY NMR (400 MHz, DMSO-d_6)

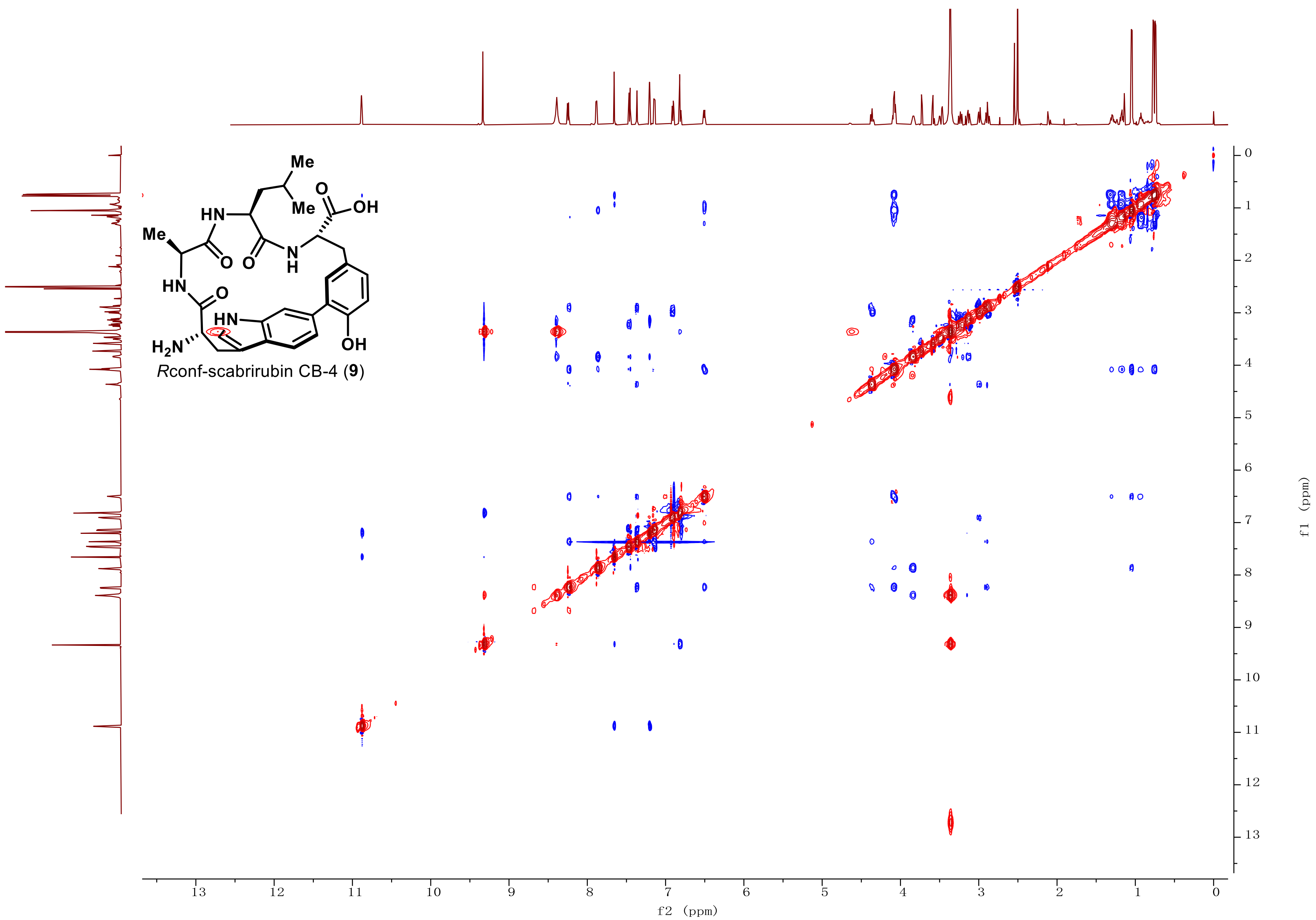

*R*conf-scabrirubin CB-4 (**9**)

Compound 55 ^{1}H NMR (400 MHz, CDCl$_3$)

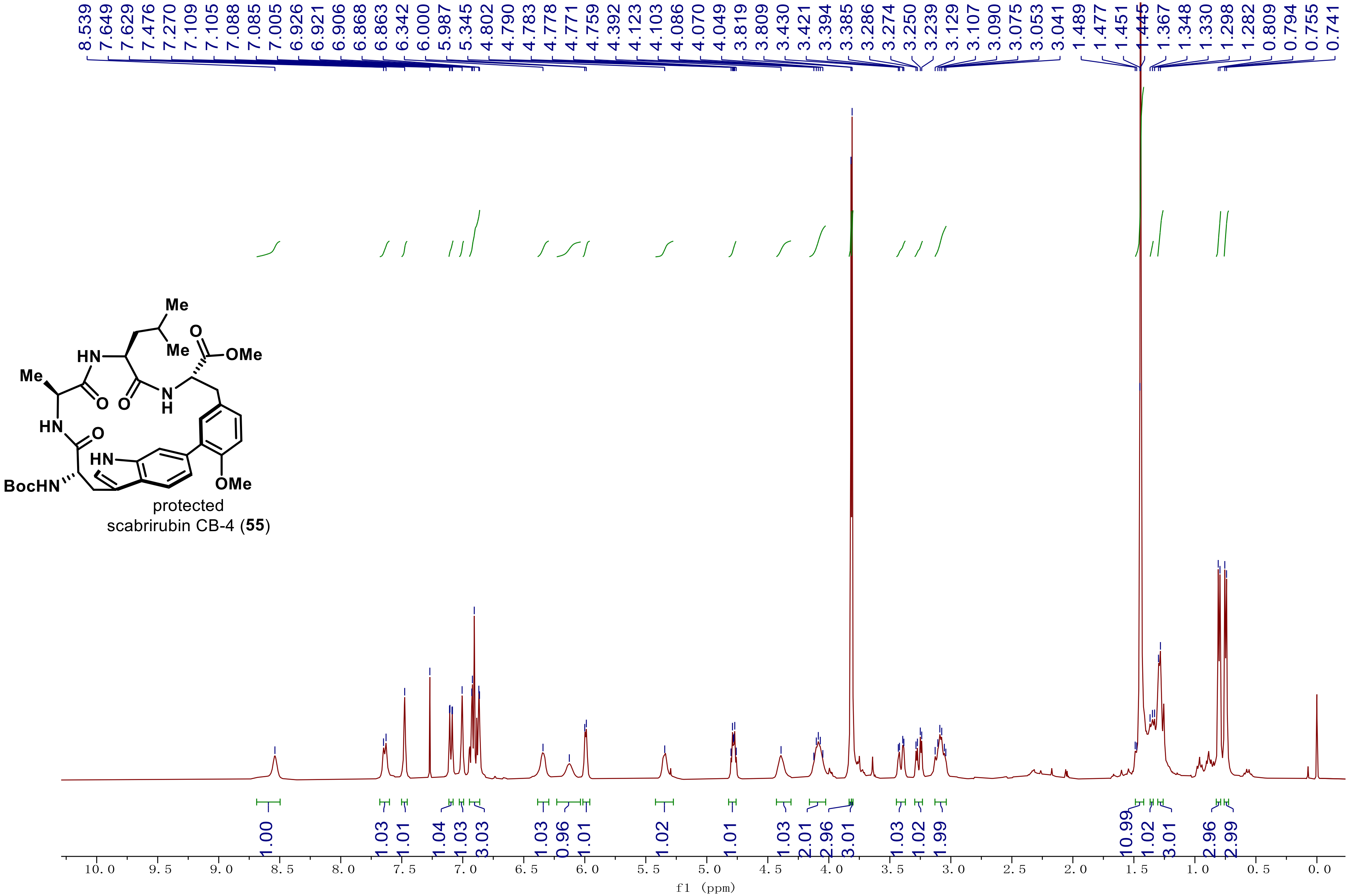

protected
scabrirubin CB-4 (**55**)

Compound 55 ^{13}C NMR (101 MHz, $CDCl_3$)

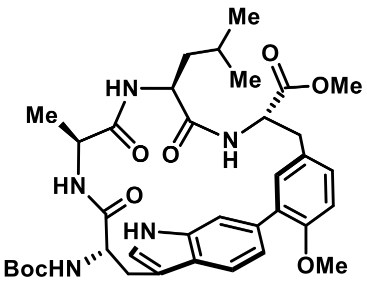
protected
scabrirubin CB-4 (**55**)

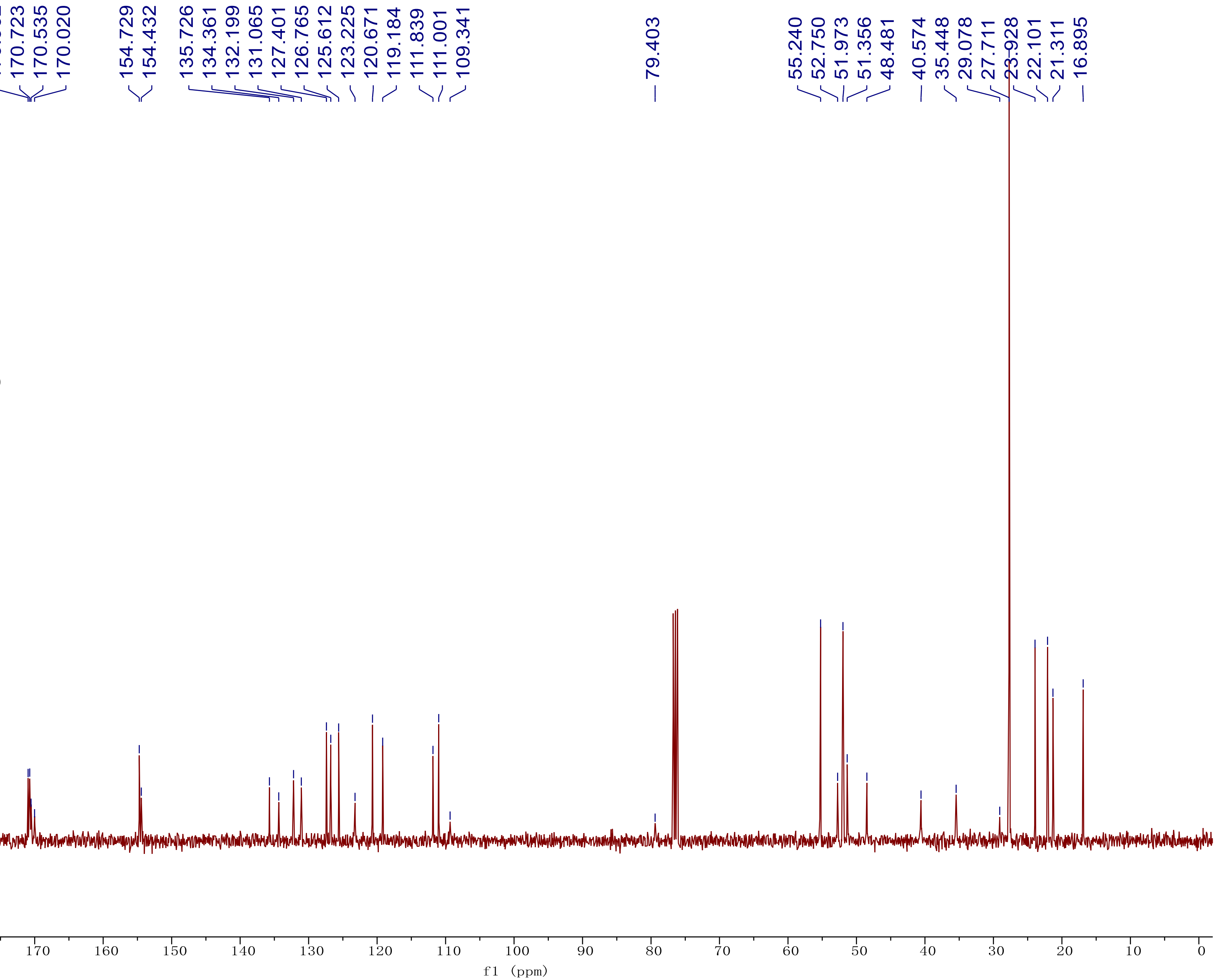

Compound 57 ^{1}H NMR (400 MHz, $CDCl_3$)

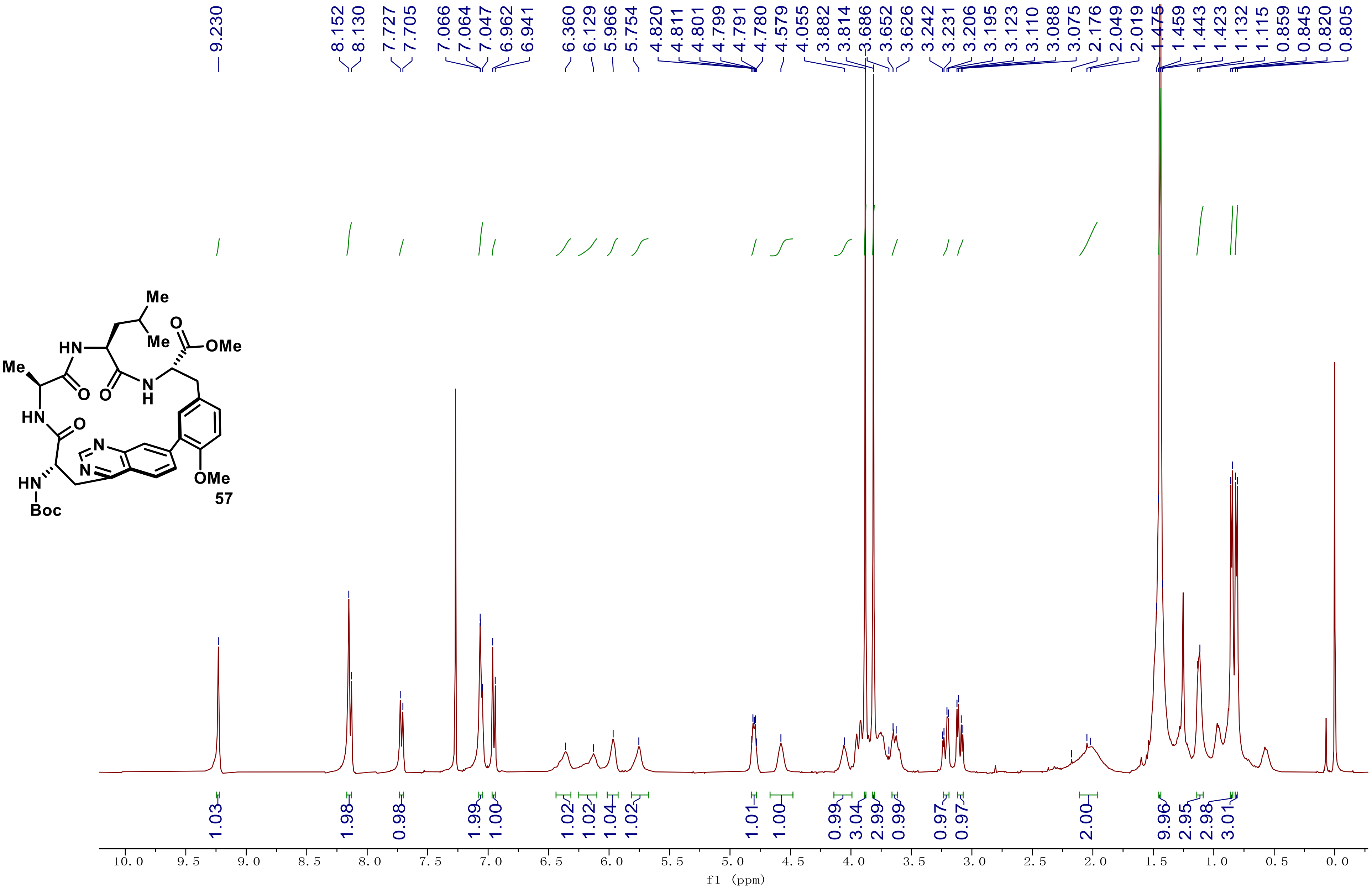

Compound 57 ^{13}C NMR (101 MHz, $CDCl_3$)

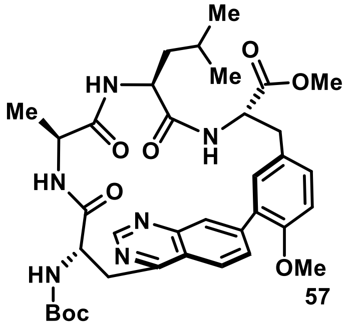

57

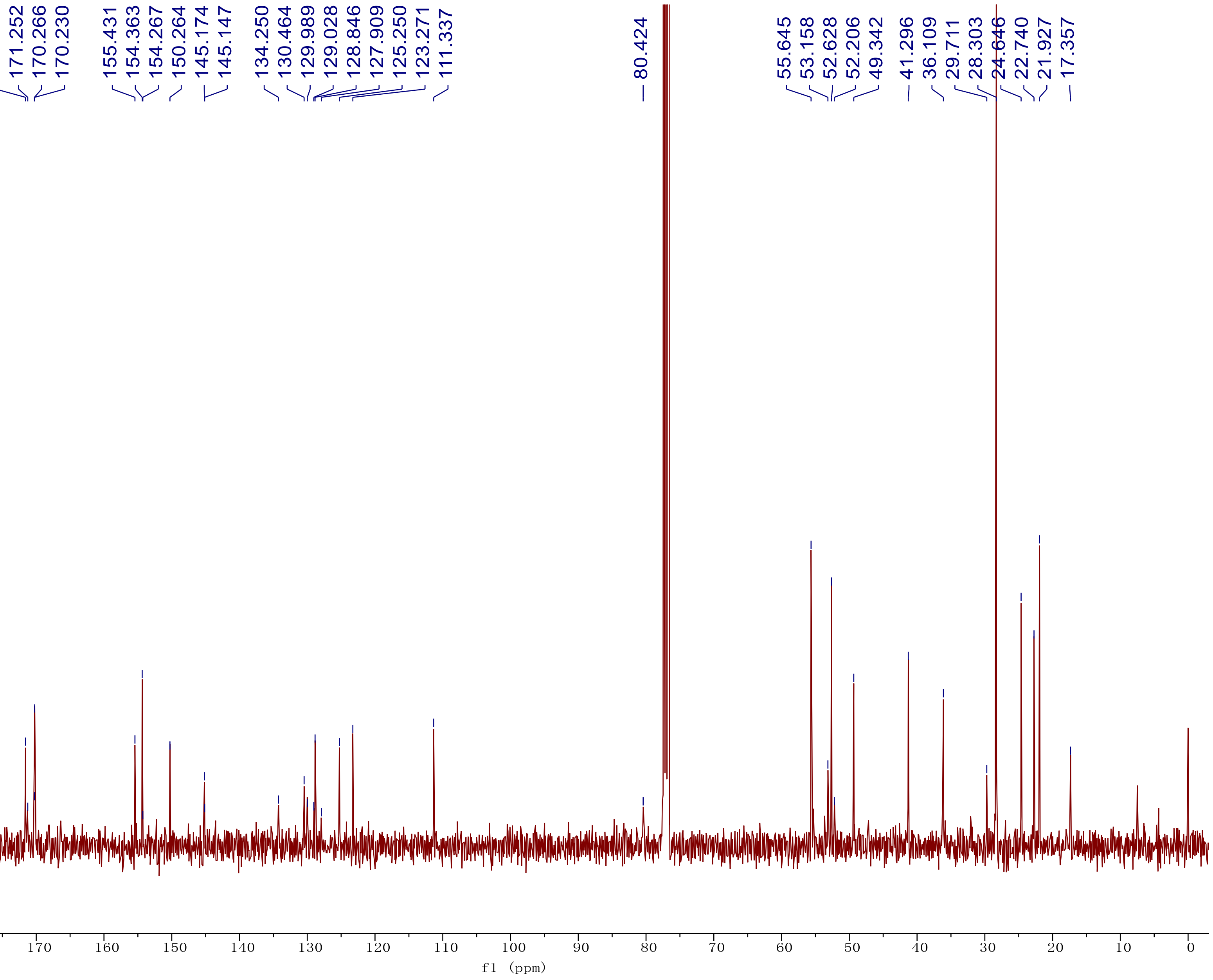

Compound 61 ^{1}H NMR (400 MHz, $CDCl_3$)

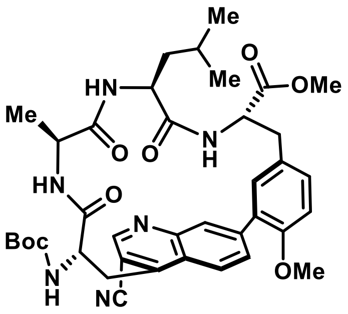

61

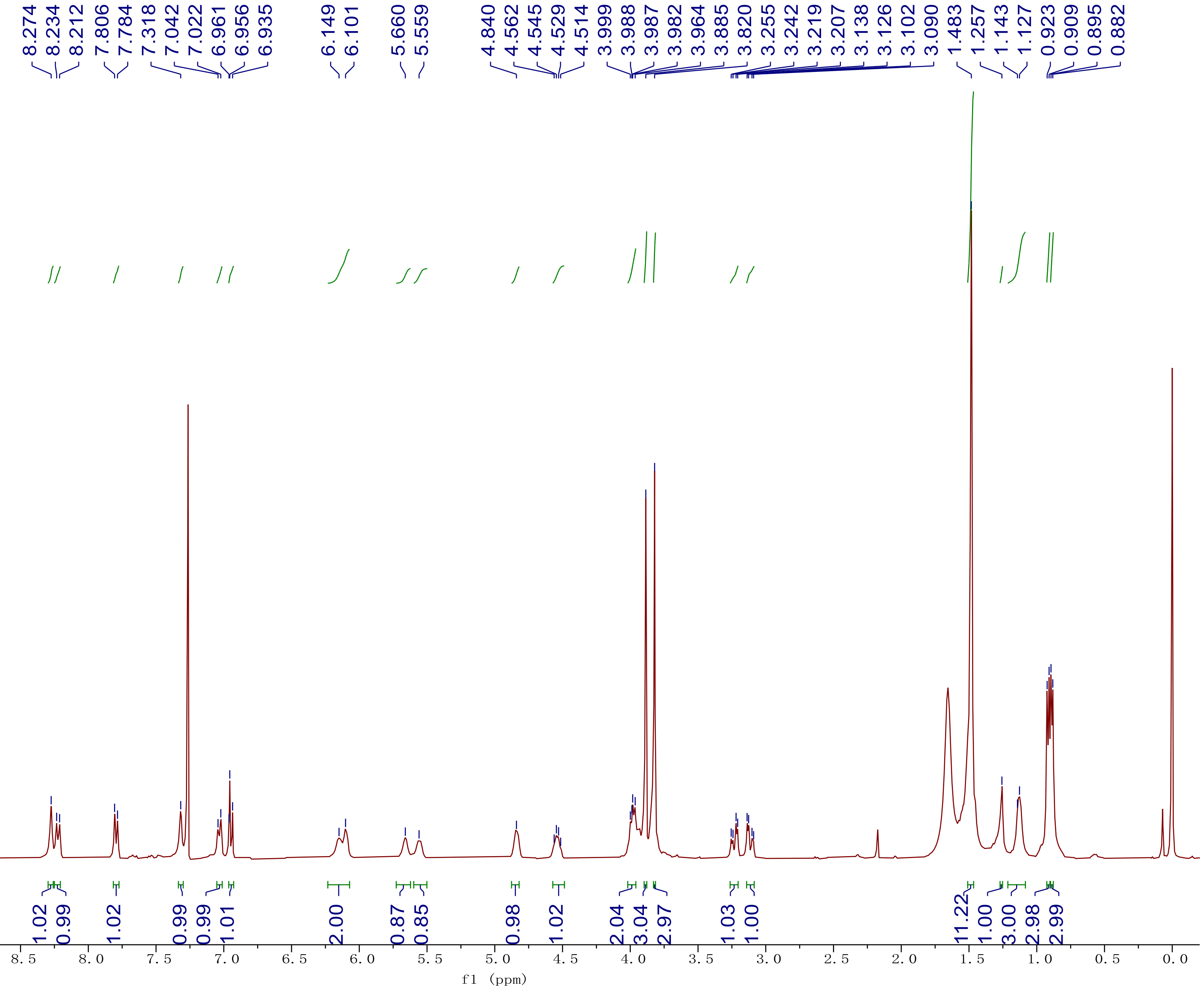

Compound 61 ^{13}C NMR (101 MHz, CDCl$_3$)

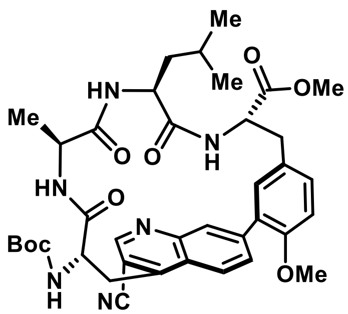

61

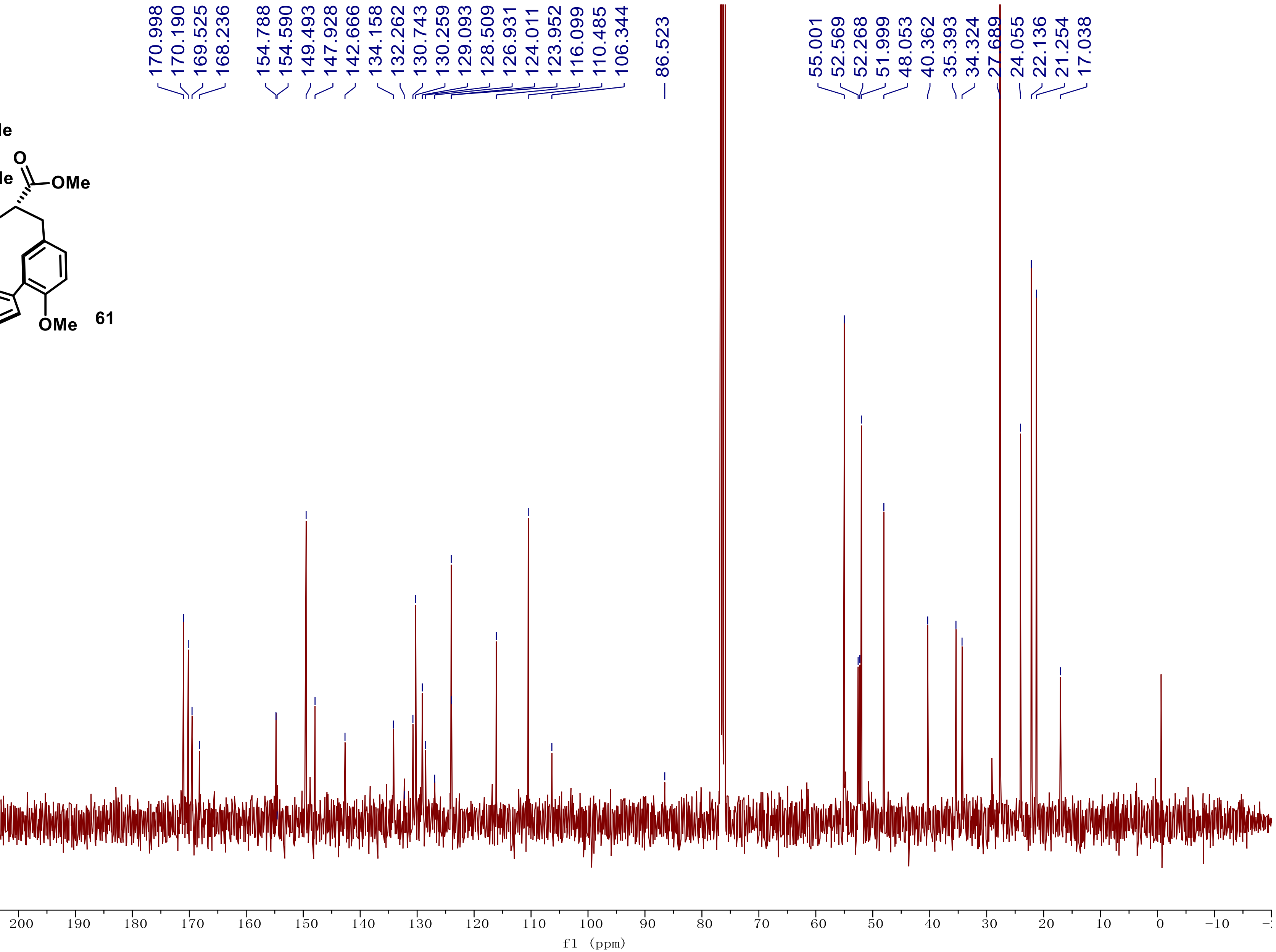

Compound 59 ^{1}H NMR (400 MHz, CD_3OD, 60 °C)

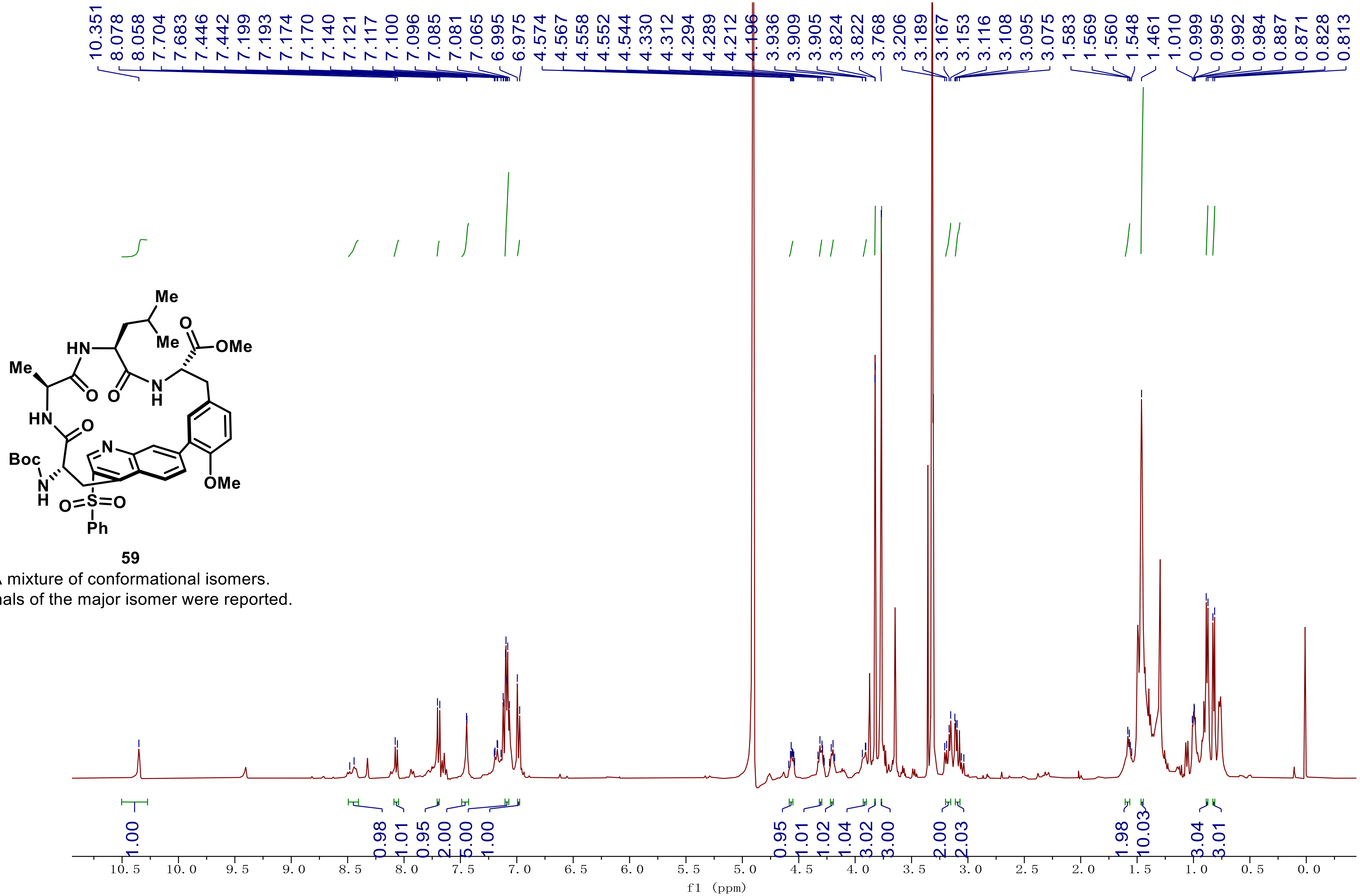

59

A mixture of conformational isomers.
Signals of the major isomer were reported.

Compound 59 ^{13}C NMR (101 MHz, CD_3OD, 60 °C)

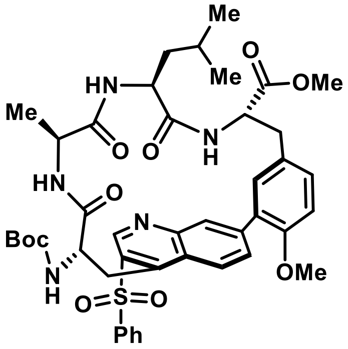

59

A mixture of conformational isomers.
Signals of the major isomer were reported.

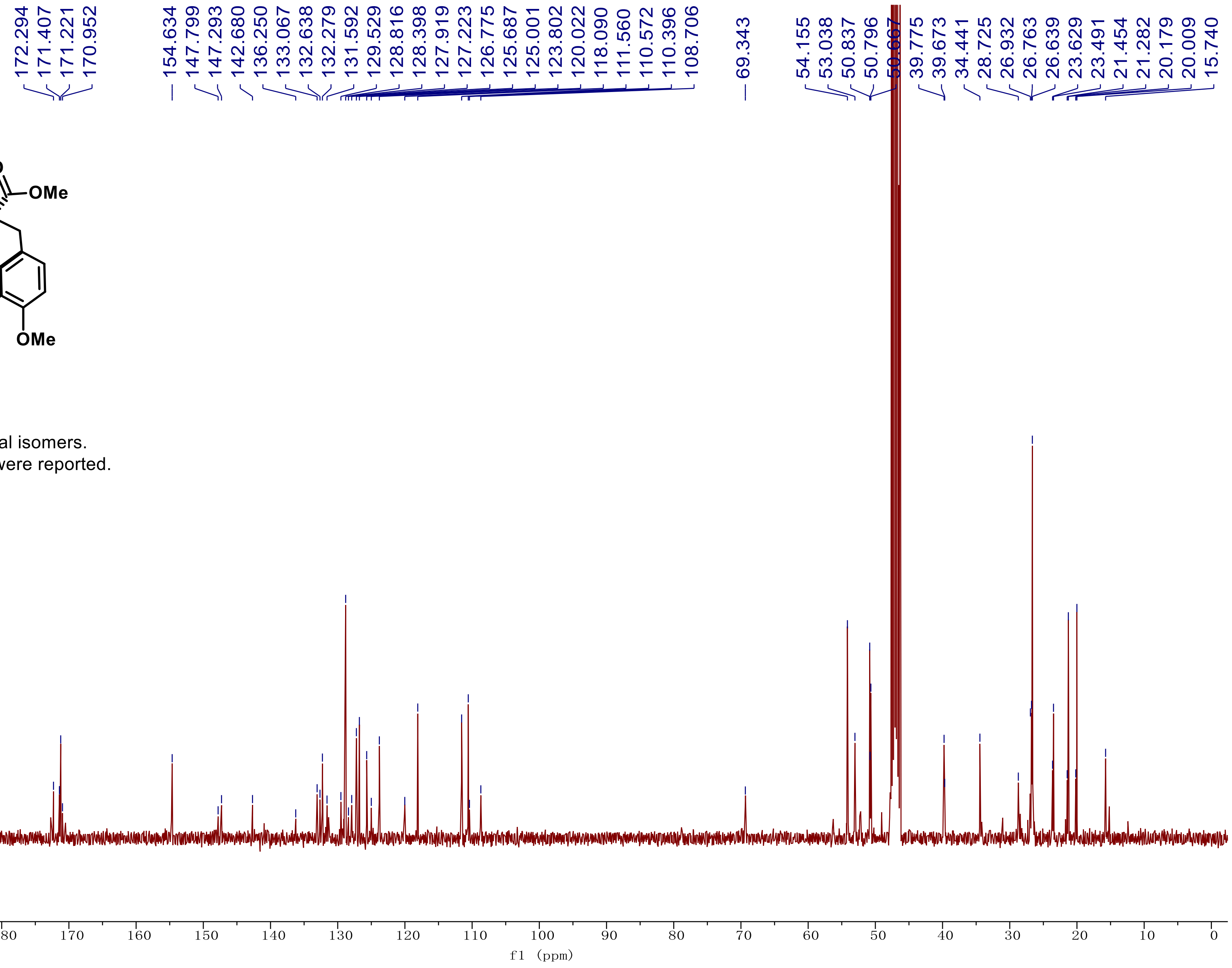

Compound 60 ^{1}H NMR (400 MHz, $CDCl_3$)

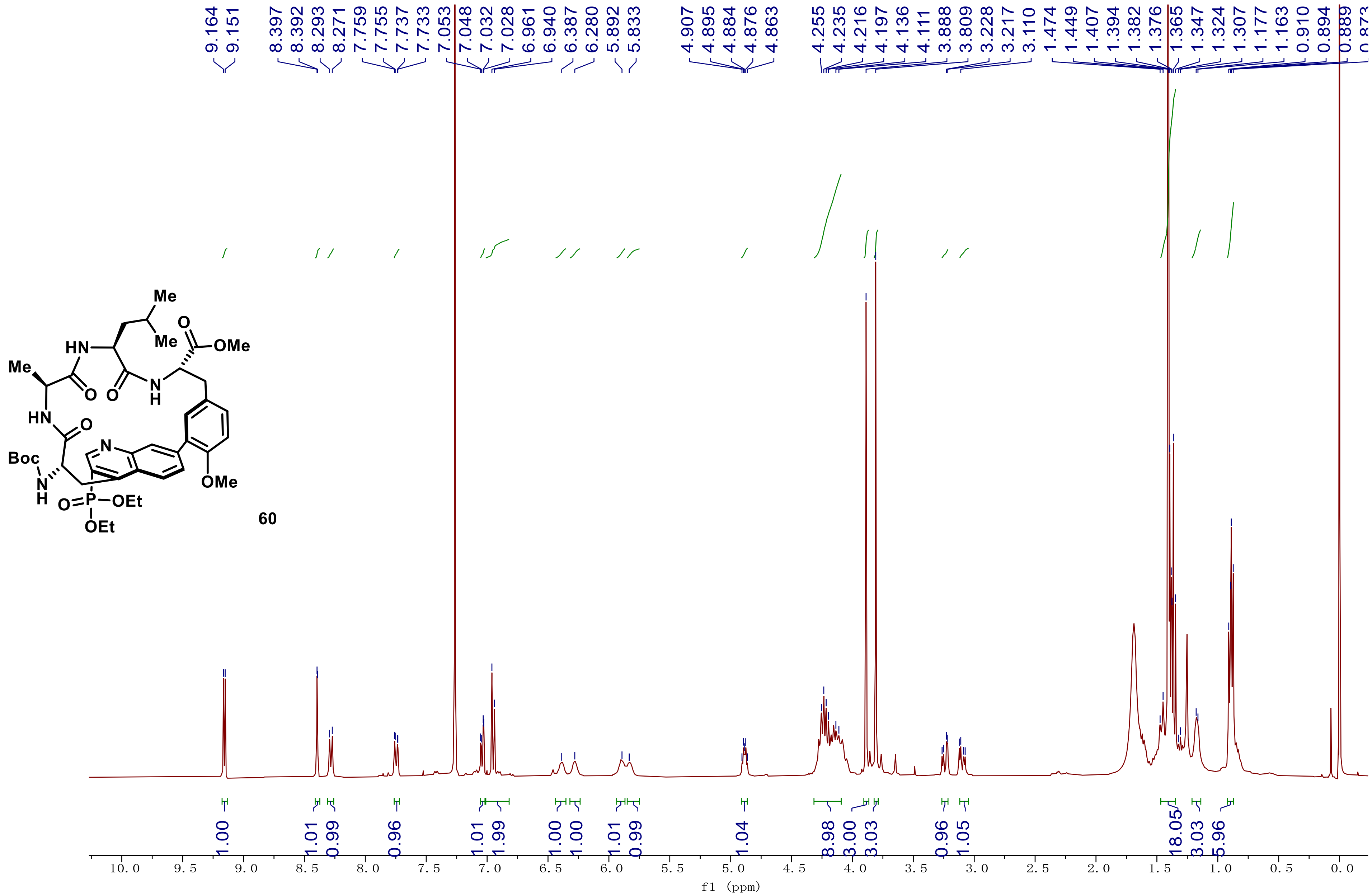

Compound 60 ^{13}C NMR (101 MHz, $CDCl_3$)

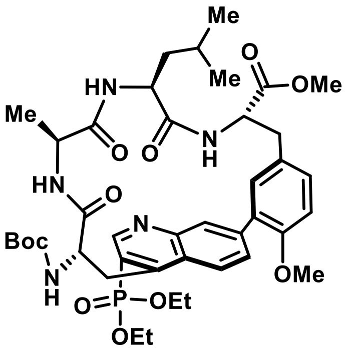

60

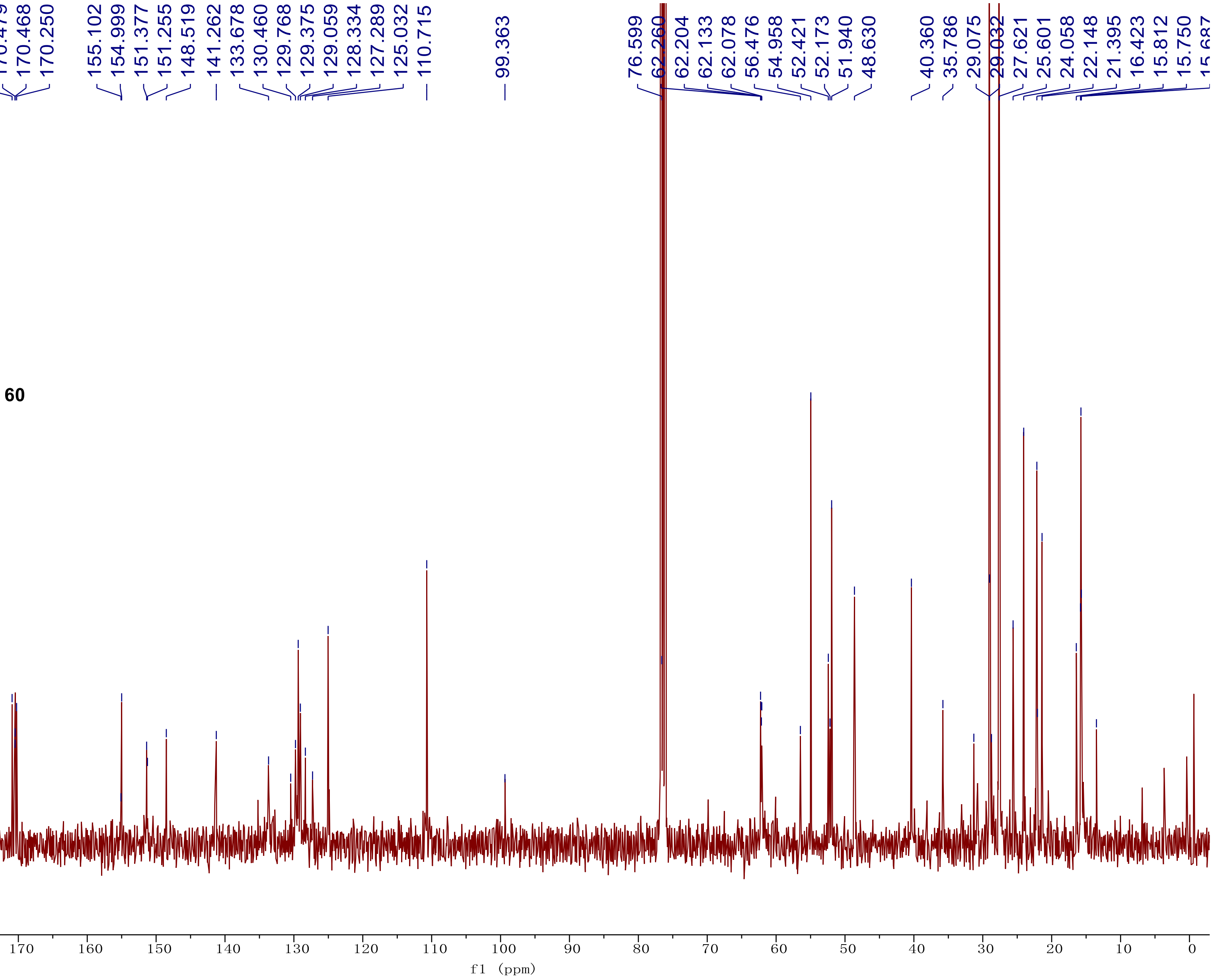

Compound S18 ^{1}HNMR (600 MHz, CDCl$_3$)

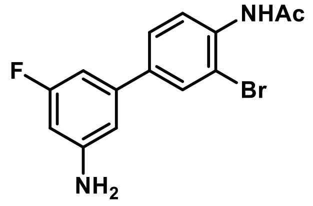

S18

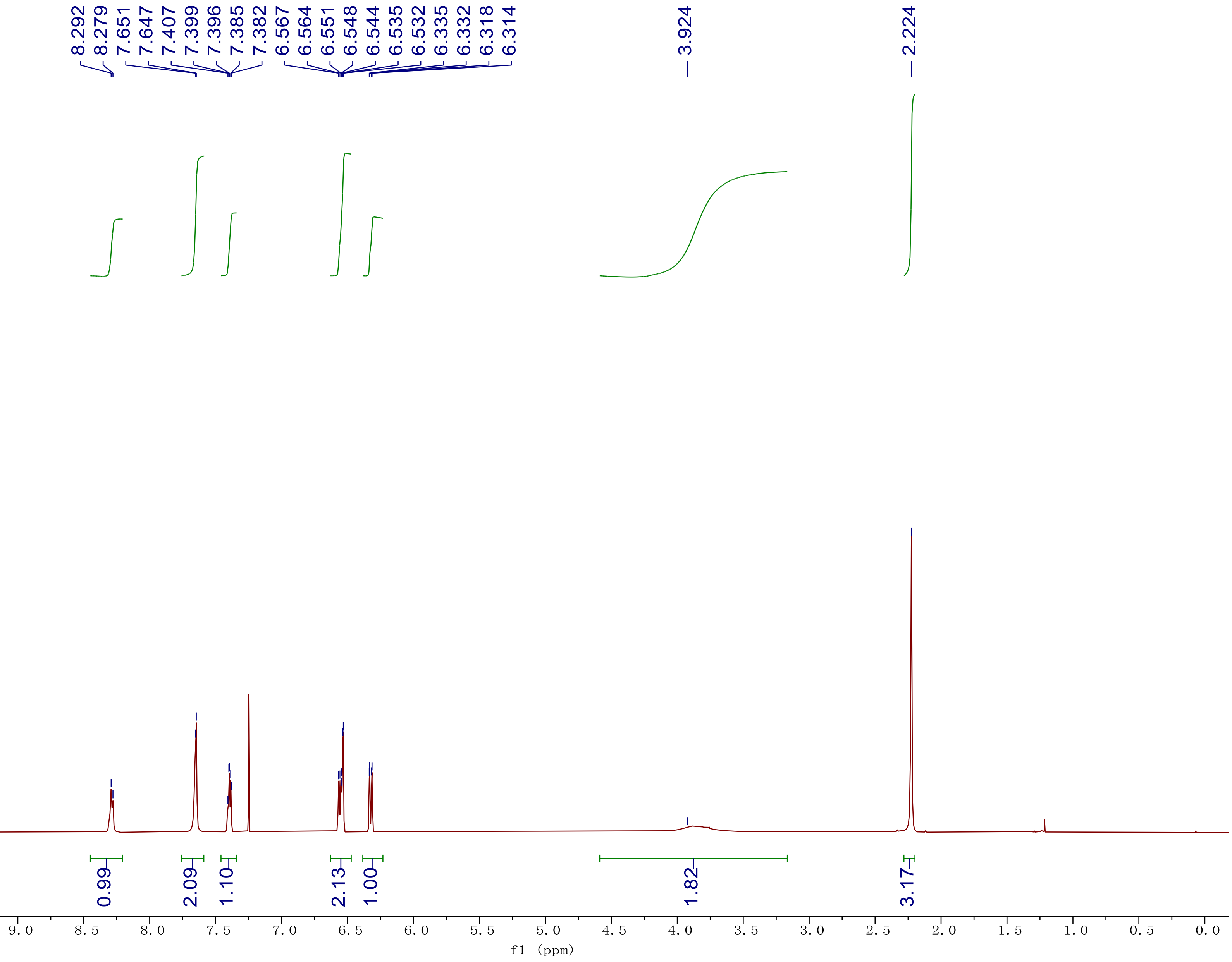

Compound S18 ^{13}CNMR (151 MHz, $CDCl_3$)

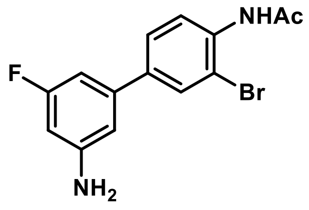

S18

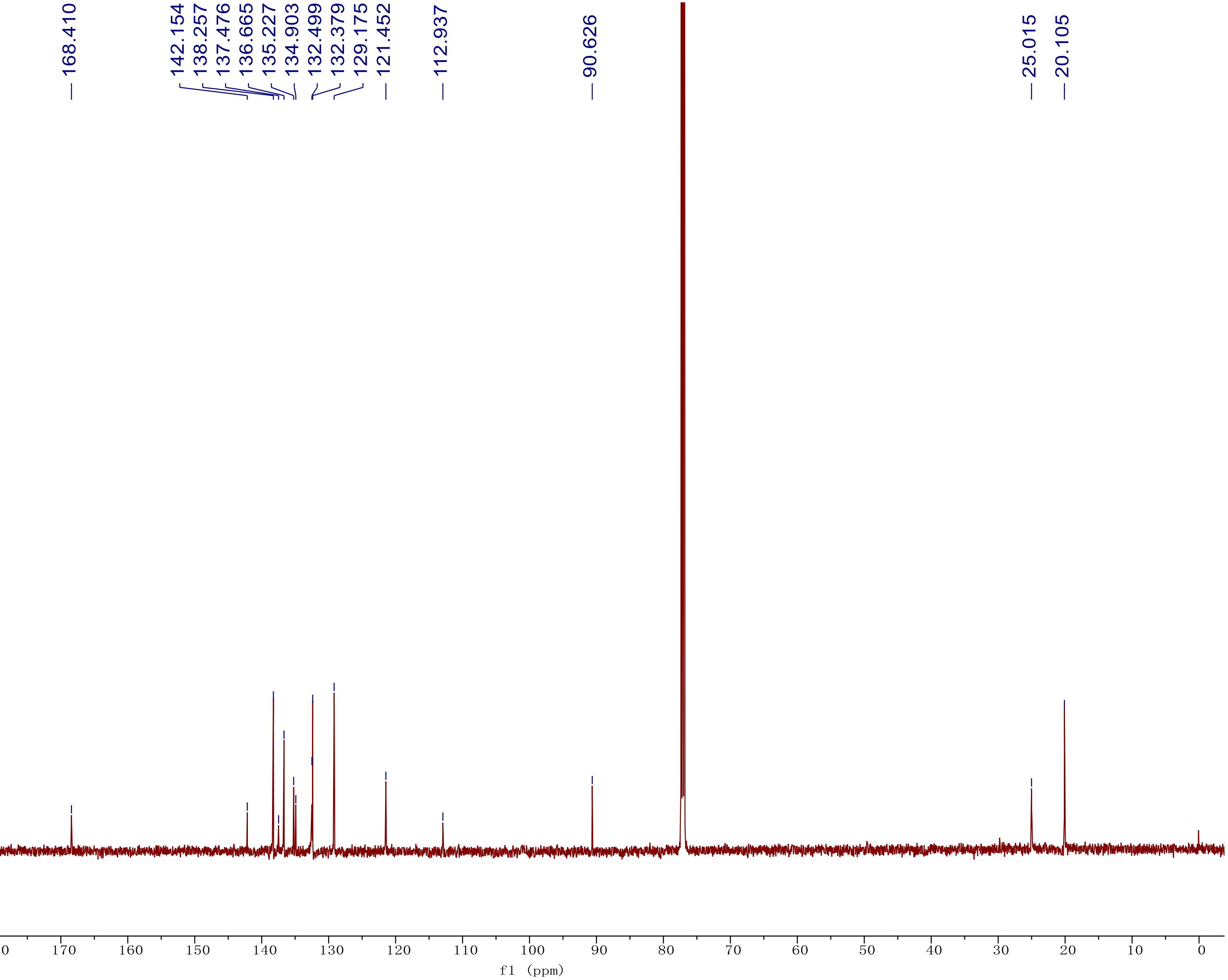

Compound S18 ^{19}F NMR (565 MHz, $CDCl_3$)

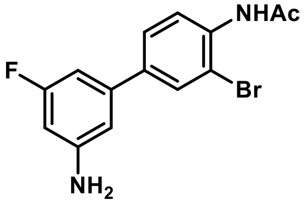

S18

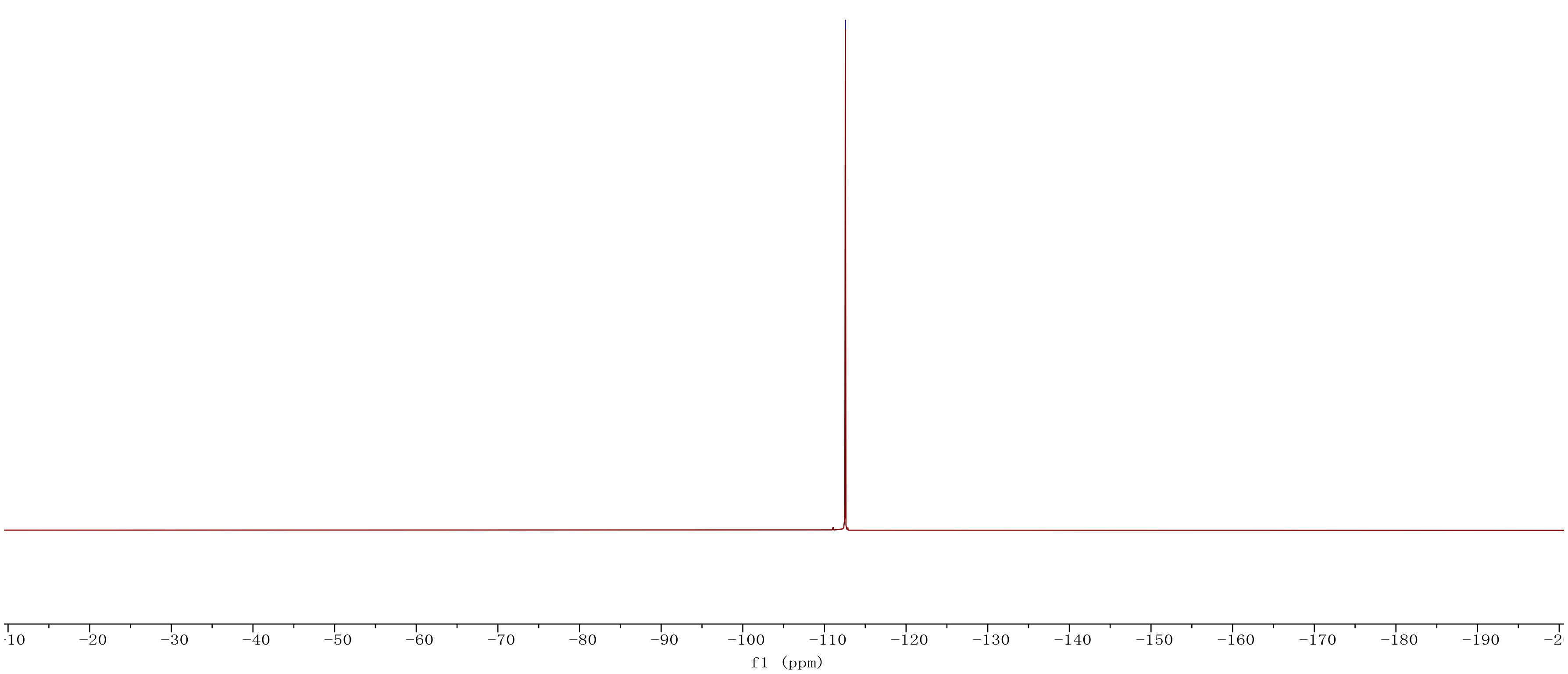

Compound S19 ¹HNMR (600 MHz, $CDCl_3$)

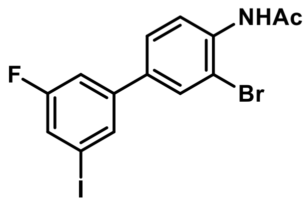

S19

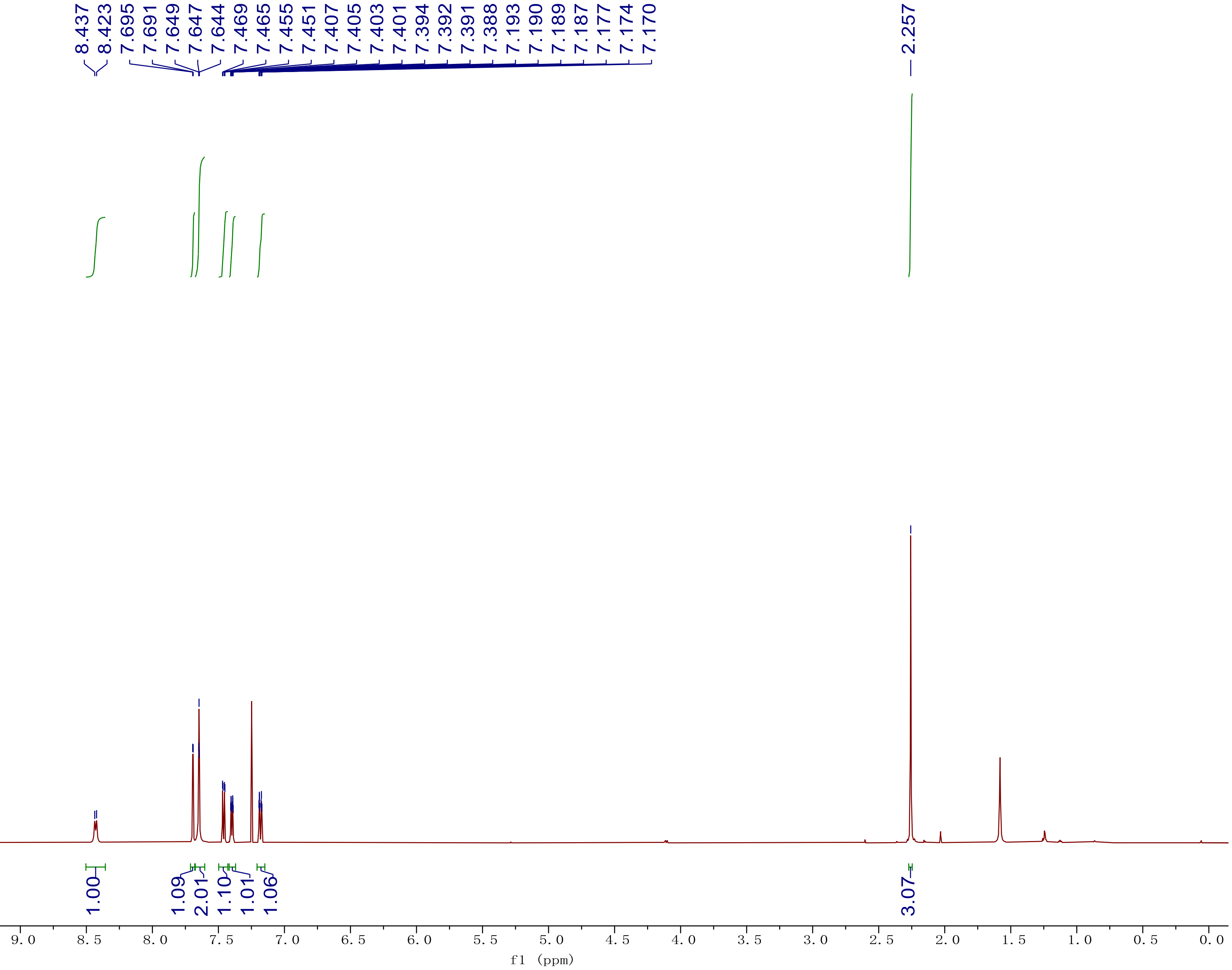

Compound S19 ^{13}CNMR (151 MHz, $CDCl_3$)

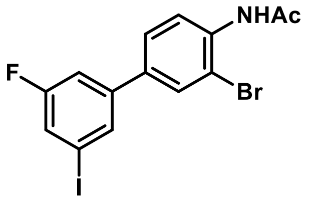

S19

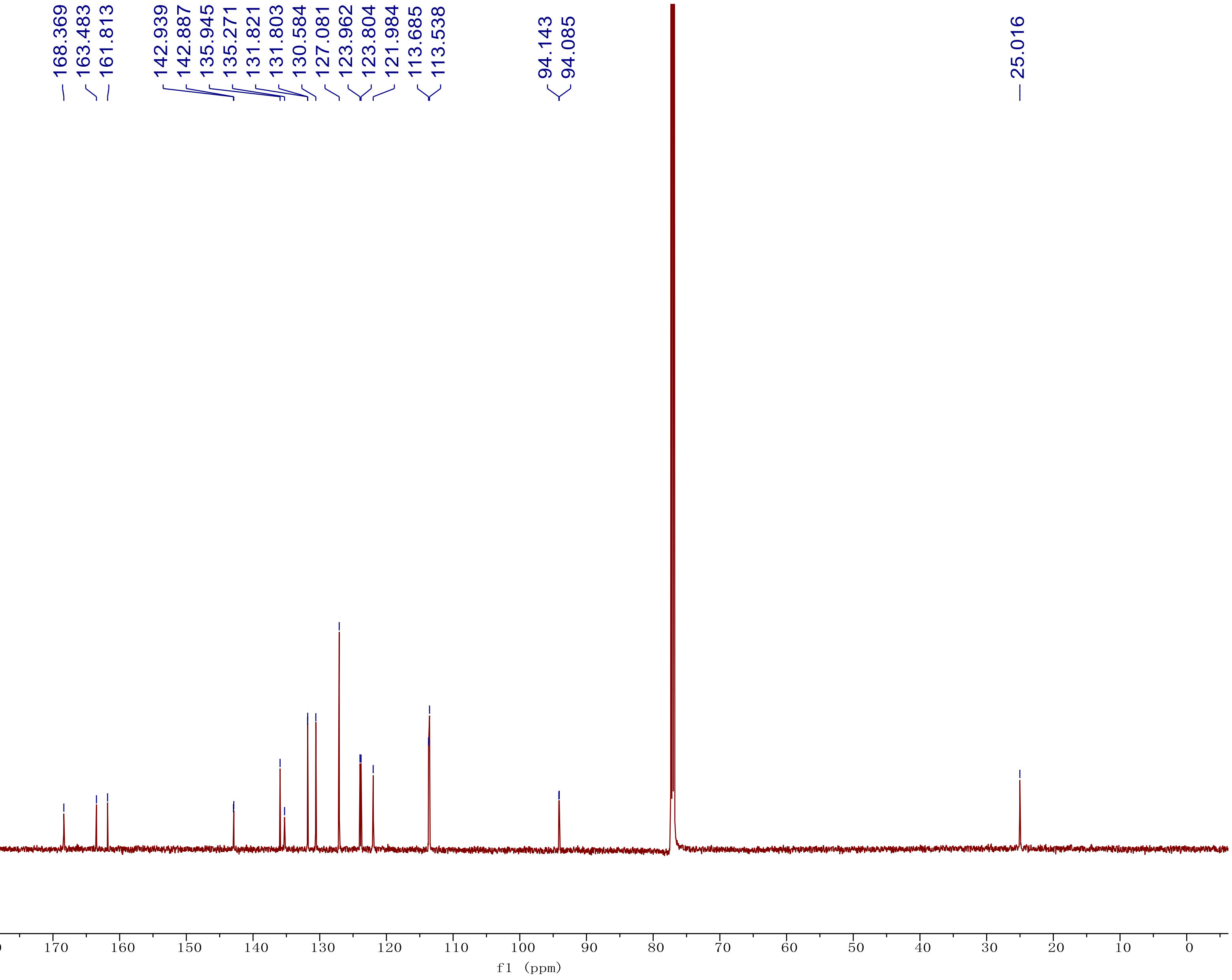

Compound S10 ^{19}F NMR (565 MHz, $CDCl_3$)

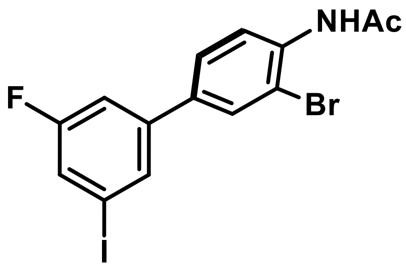

S10

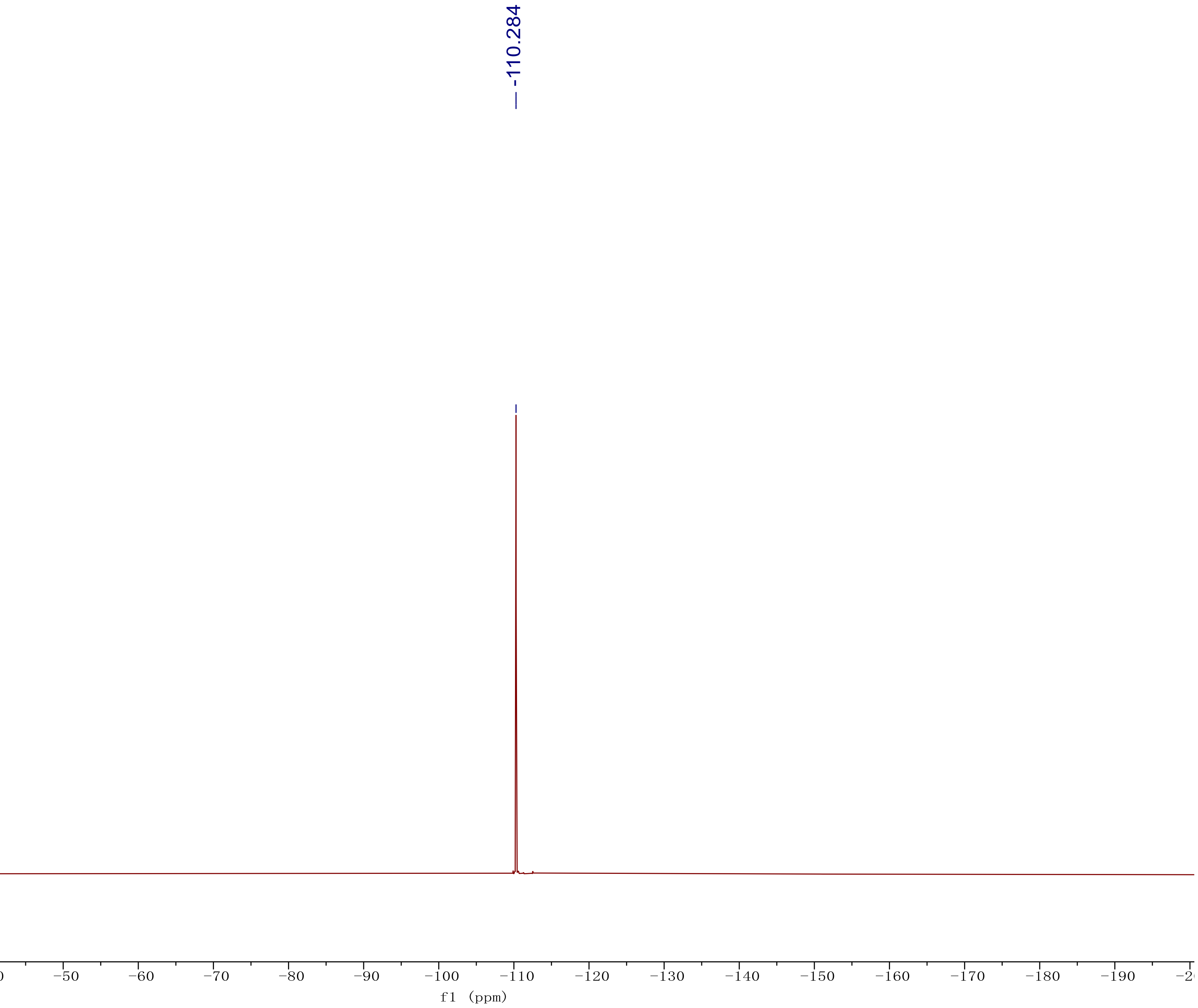

Compound 63 ^{1}HNMR (600 MHz, $CDCl_3$)

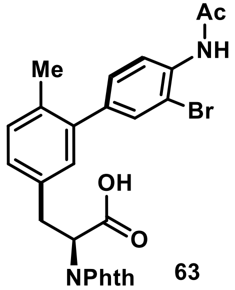

63

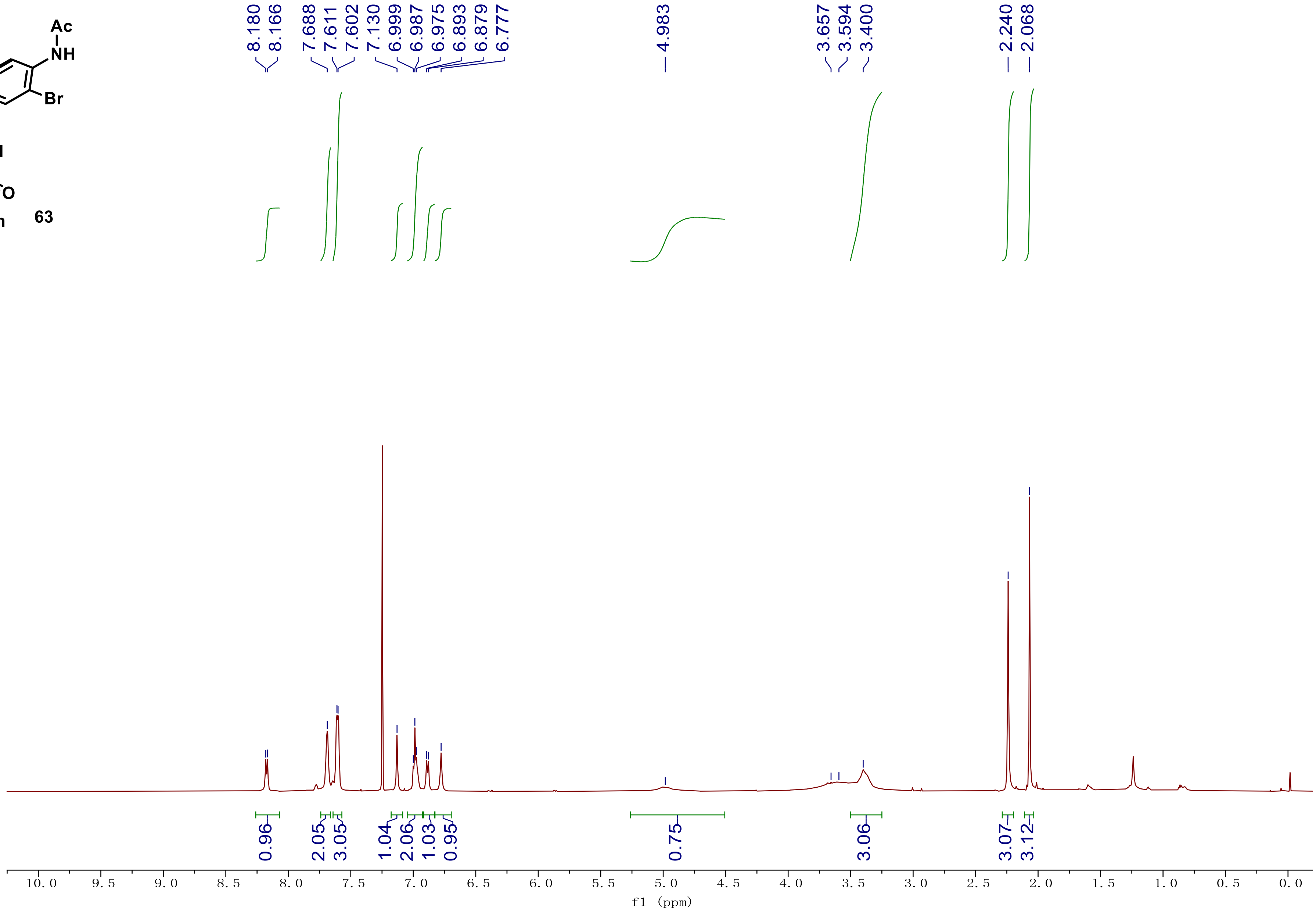

Compound 63 ^{13}CNMR (151 MHz, CDCl$_3$)

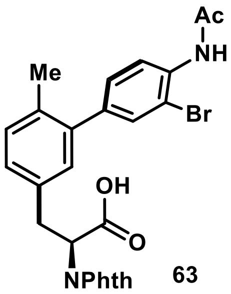

63

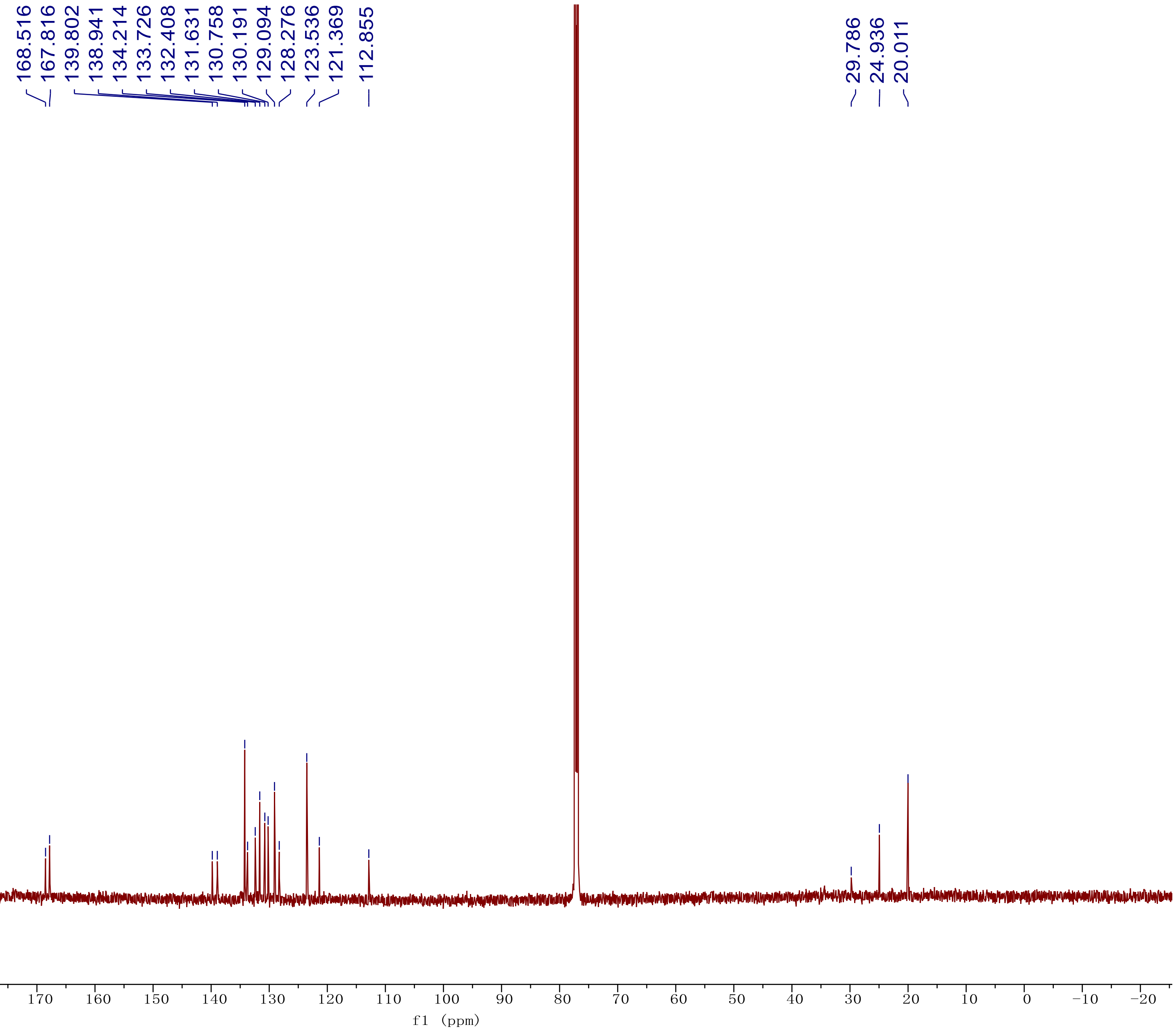

Compound 67 ^{1}HNMR (600 MHz, CDCl$_3$)

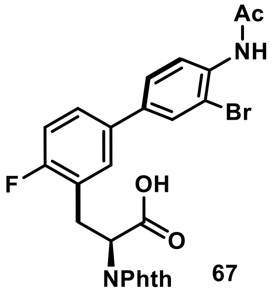

67

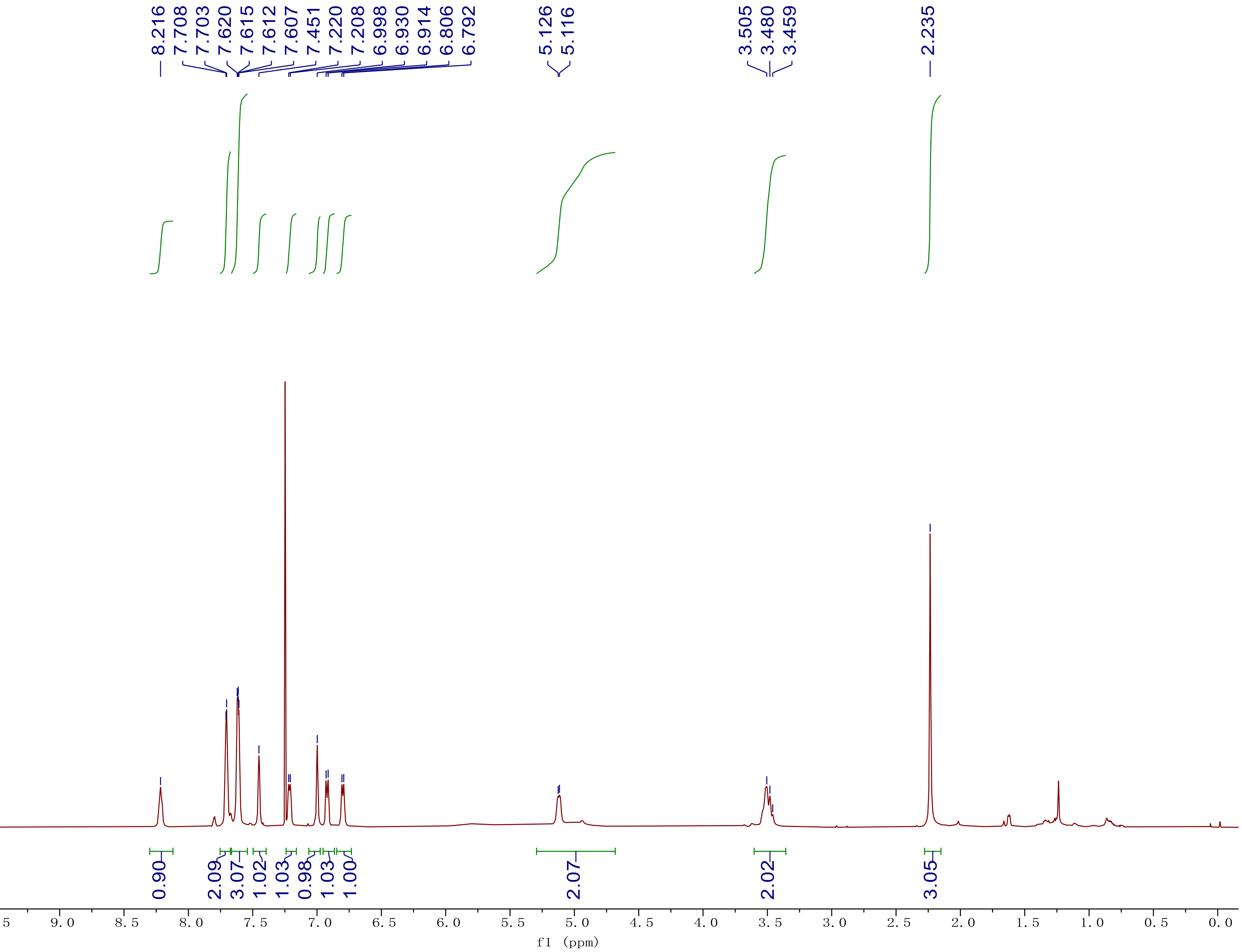

Compound 67 ^{13}CNMR (151 MHz, CDCl$_3$)

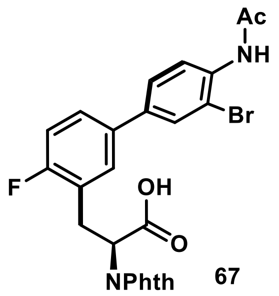

67

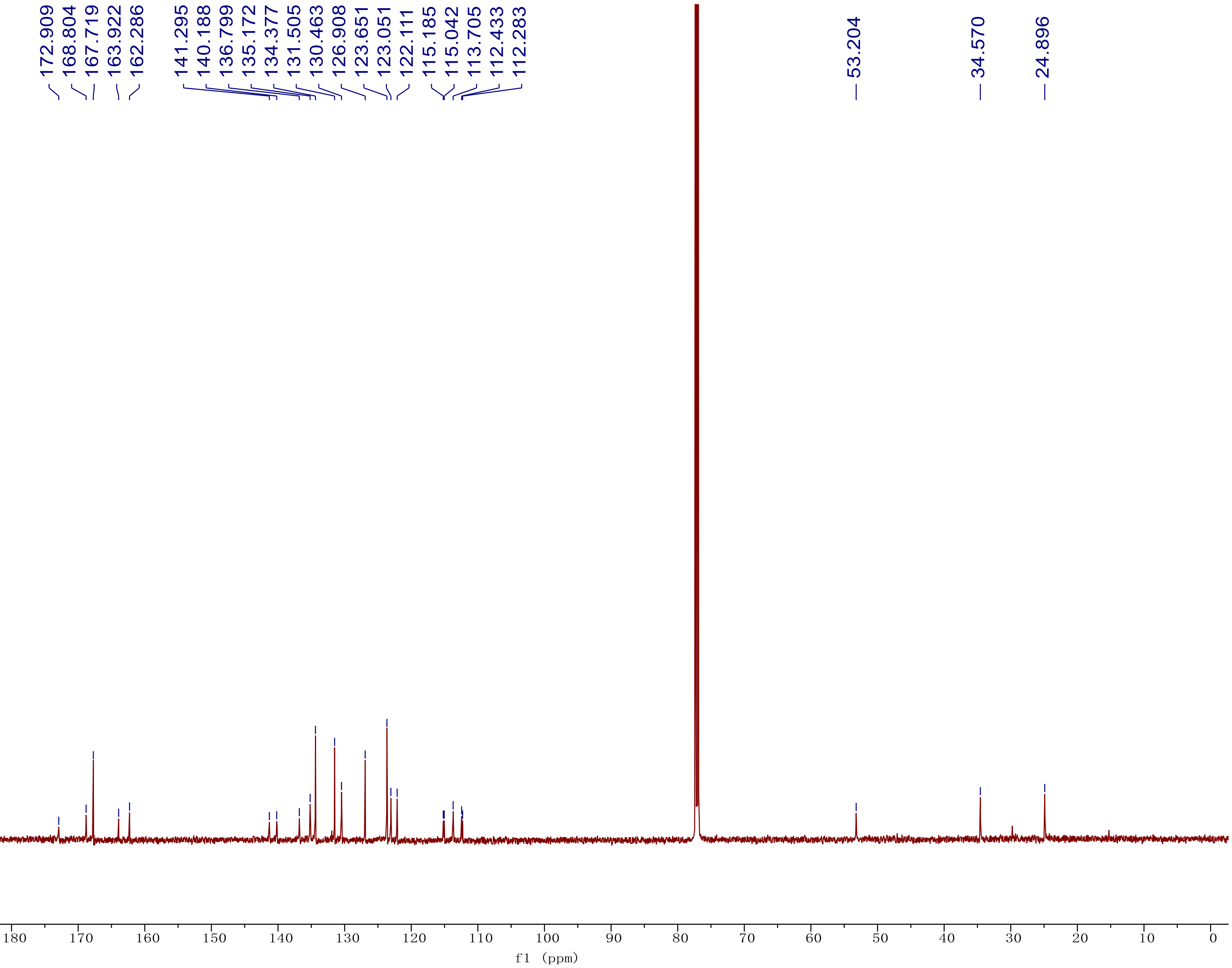

Compound 67 ^{19}F NMR (565 MHz, $CDCl_3$)

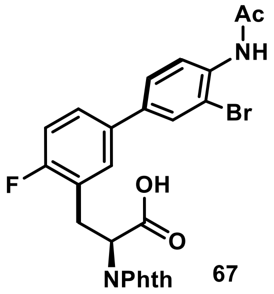

67

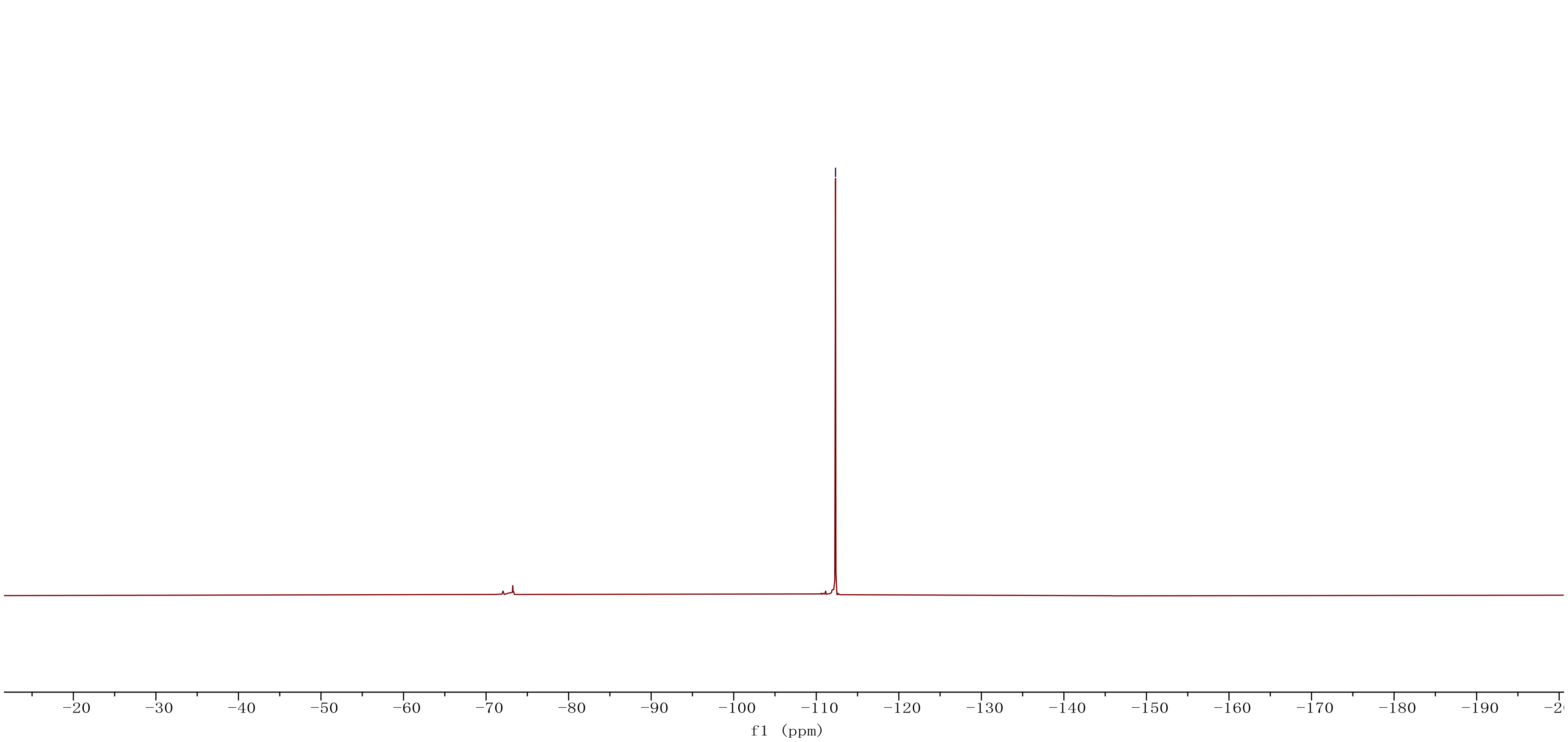

Compound 66 ^{1}HNMR (400 MHz, $CDCl_3$)

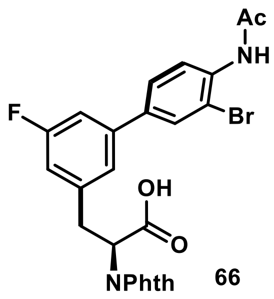

66

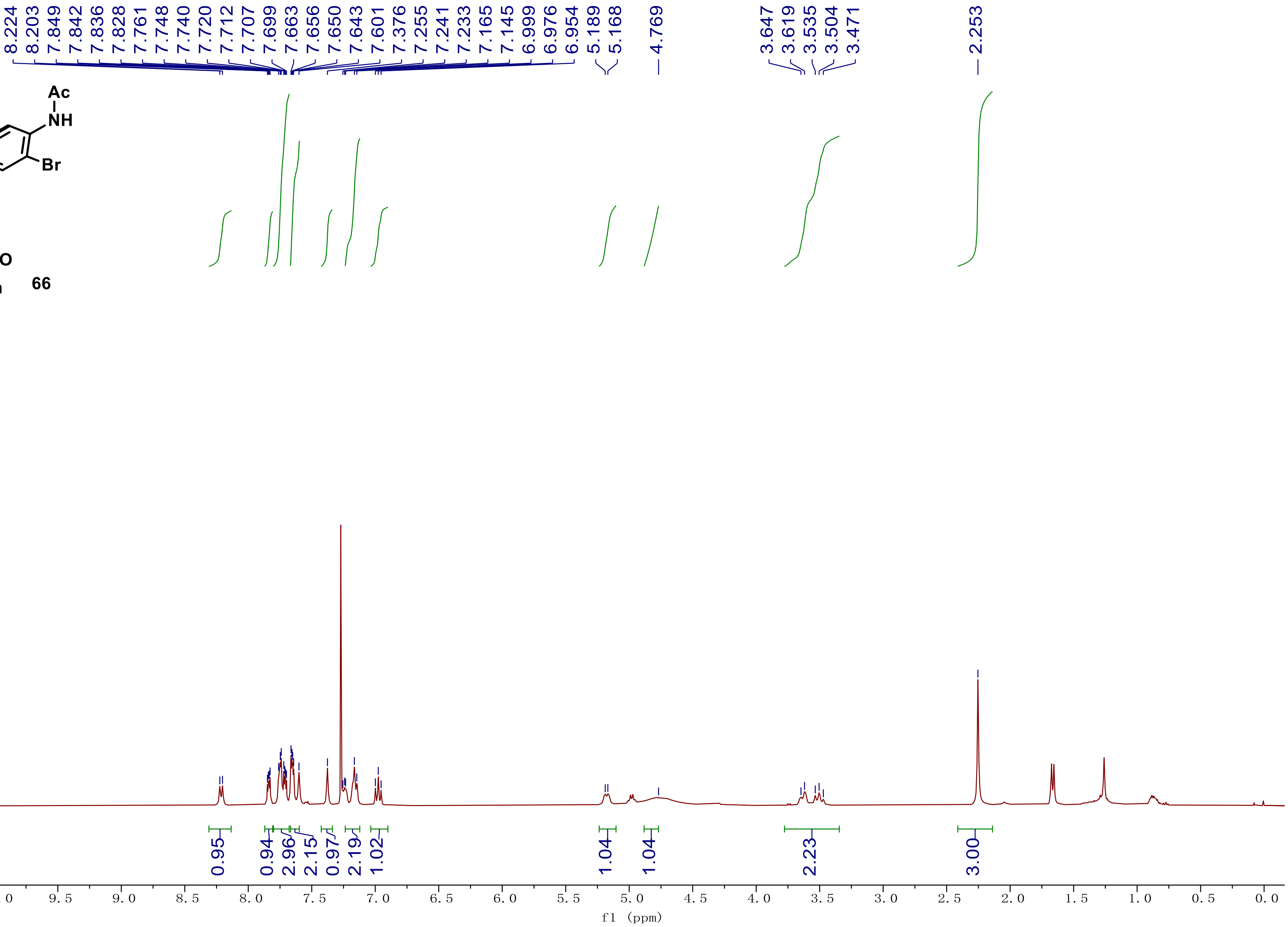

Compound 66 ^{13}CNMR (101 MHz, $CDCl_3$)

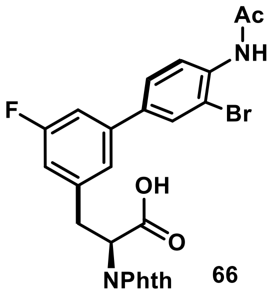

66

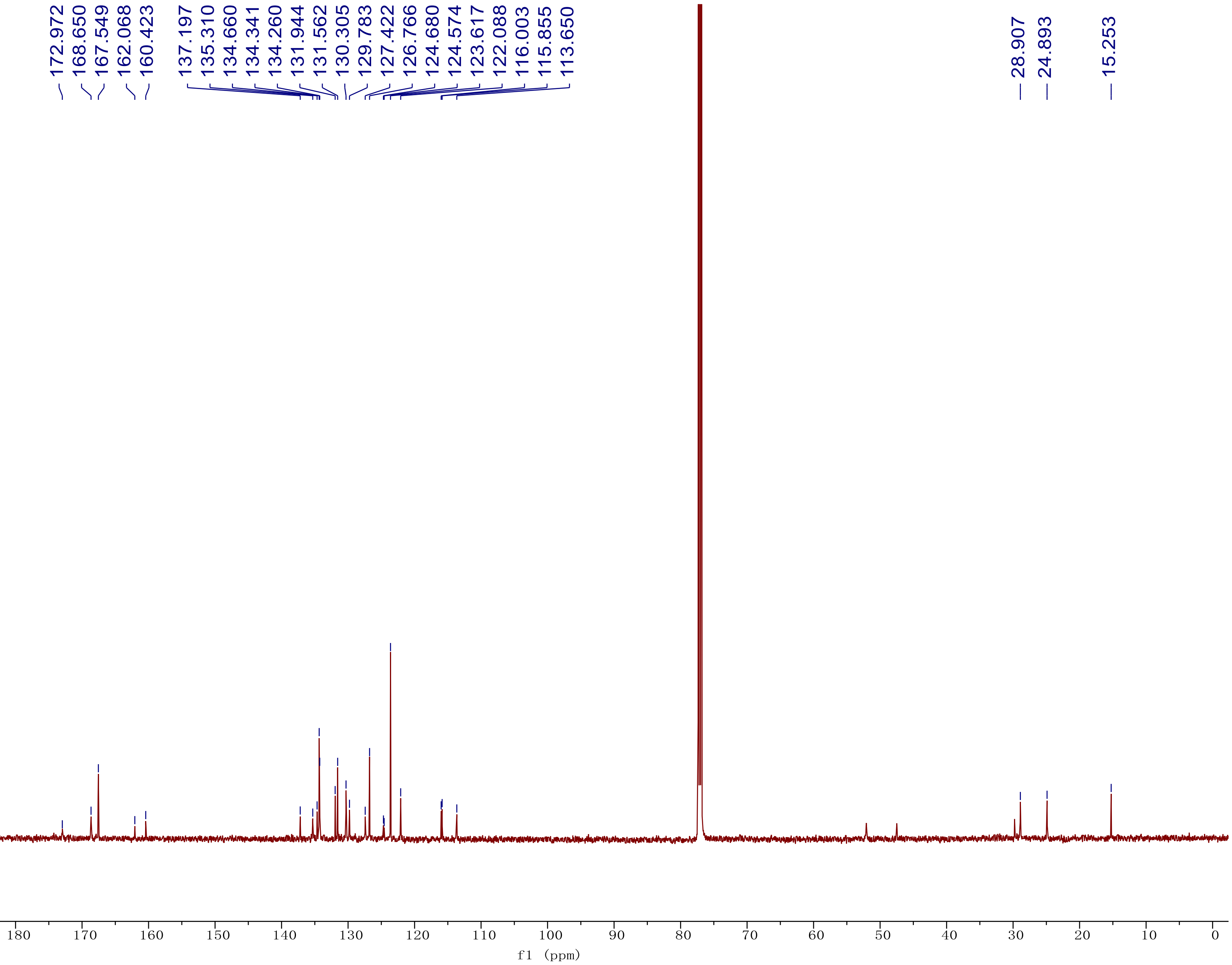

Compound 66 ^{19}F NMR (565 MHz, $CDCl_3$)

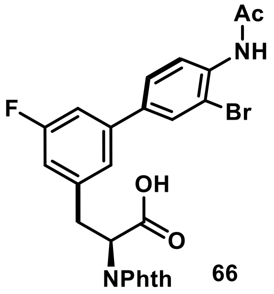

66

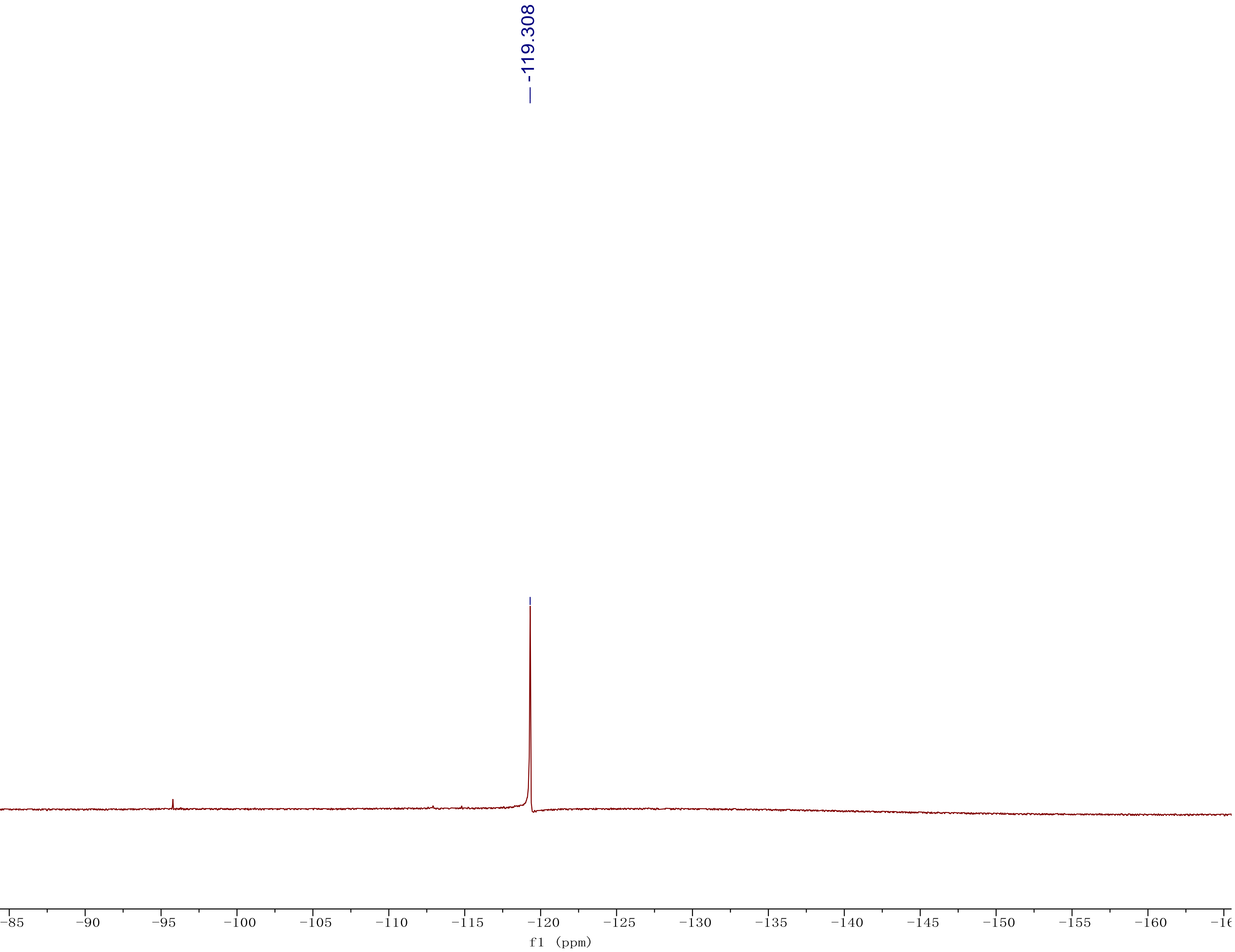

Compound S23 ¹HNMR (400 MHz, CDCl₃)

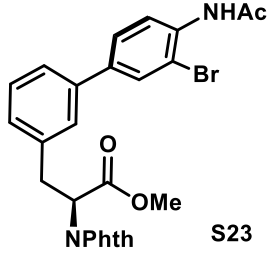

S23

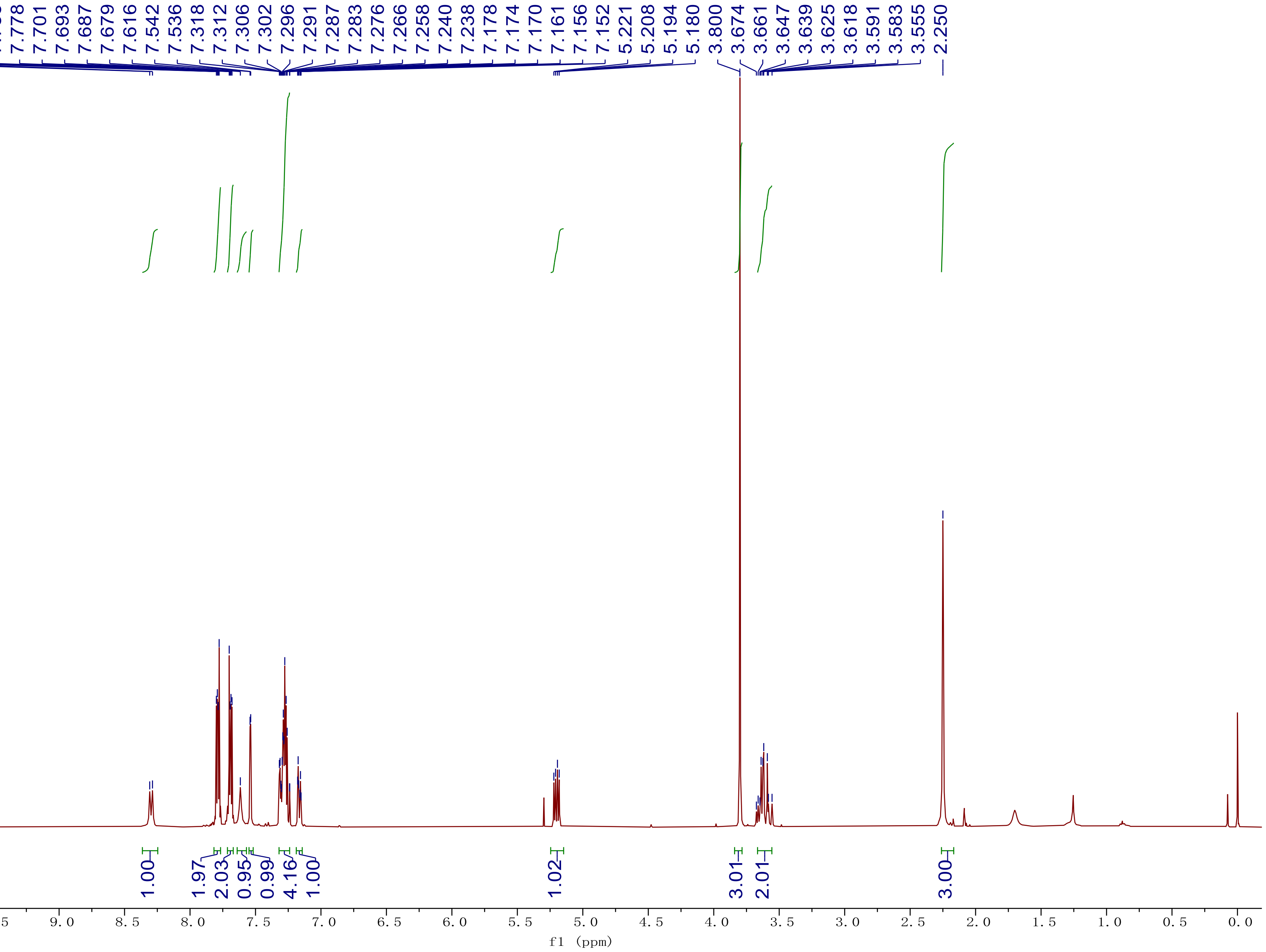

Compound S23 ^{13}C NMR (101 MHz, $CDCl_3$)

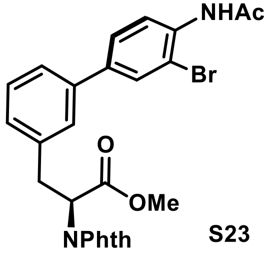

S23

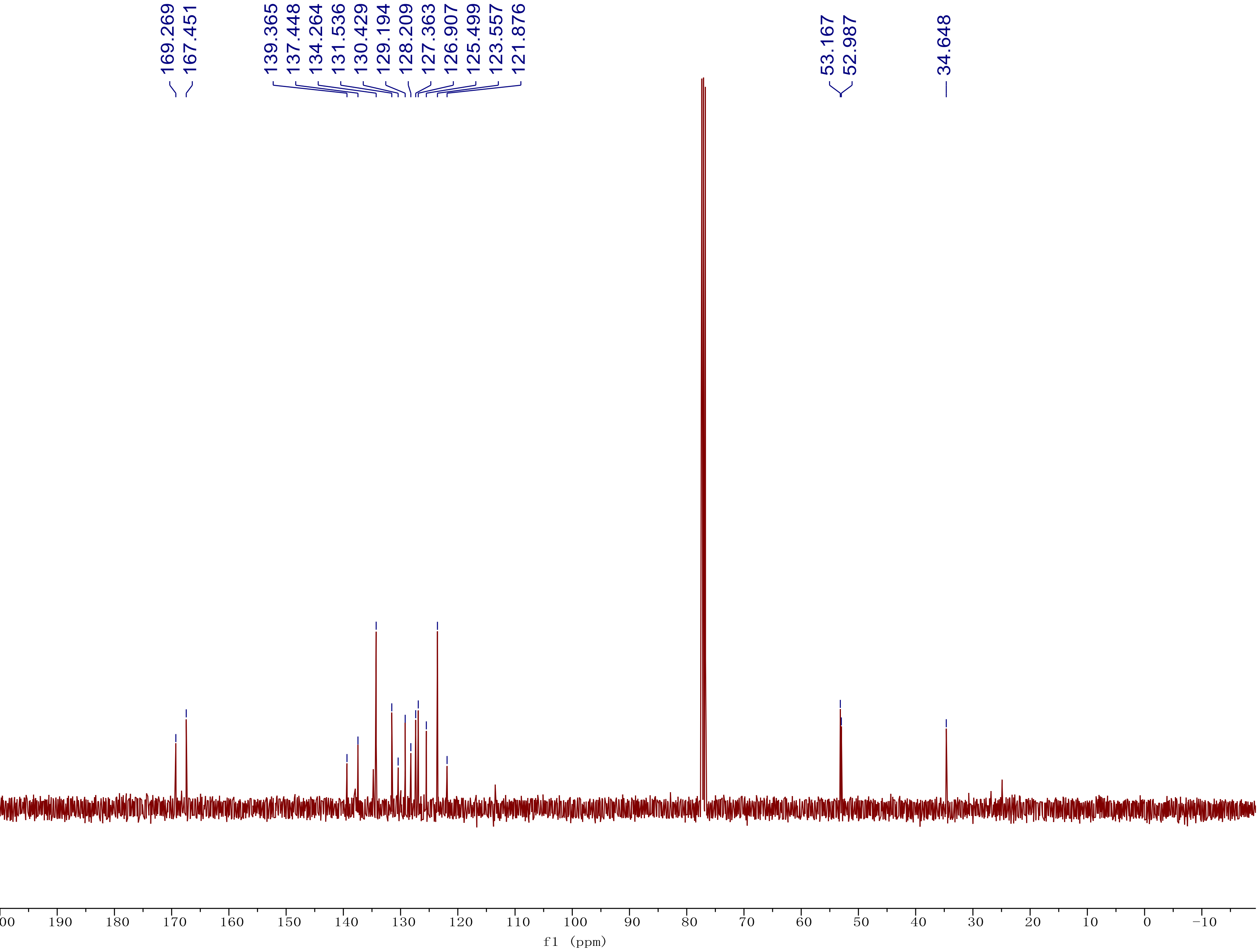

Compound S25 ^{1}H NMR (400 MHz, $CDCl_3$)

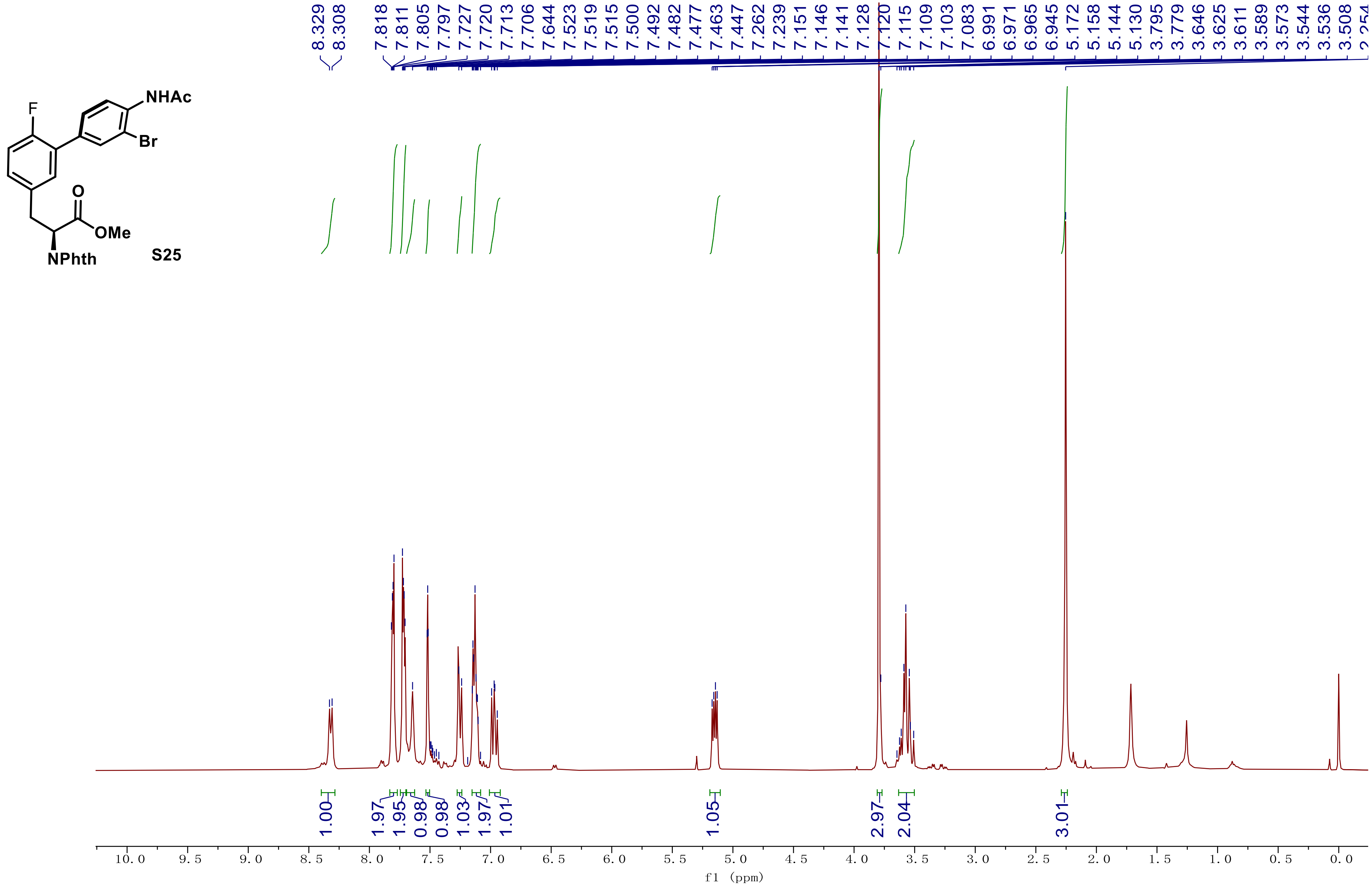

Compound S25 ^{13}C NMR (101 MHz, $CDCl_3$)

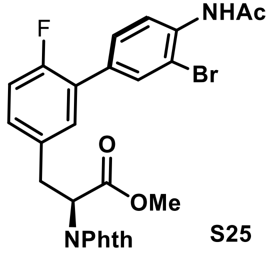

S25

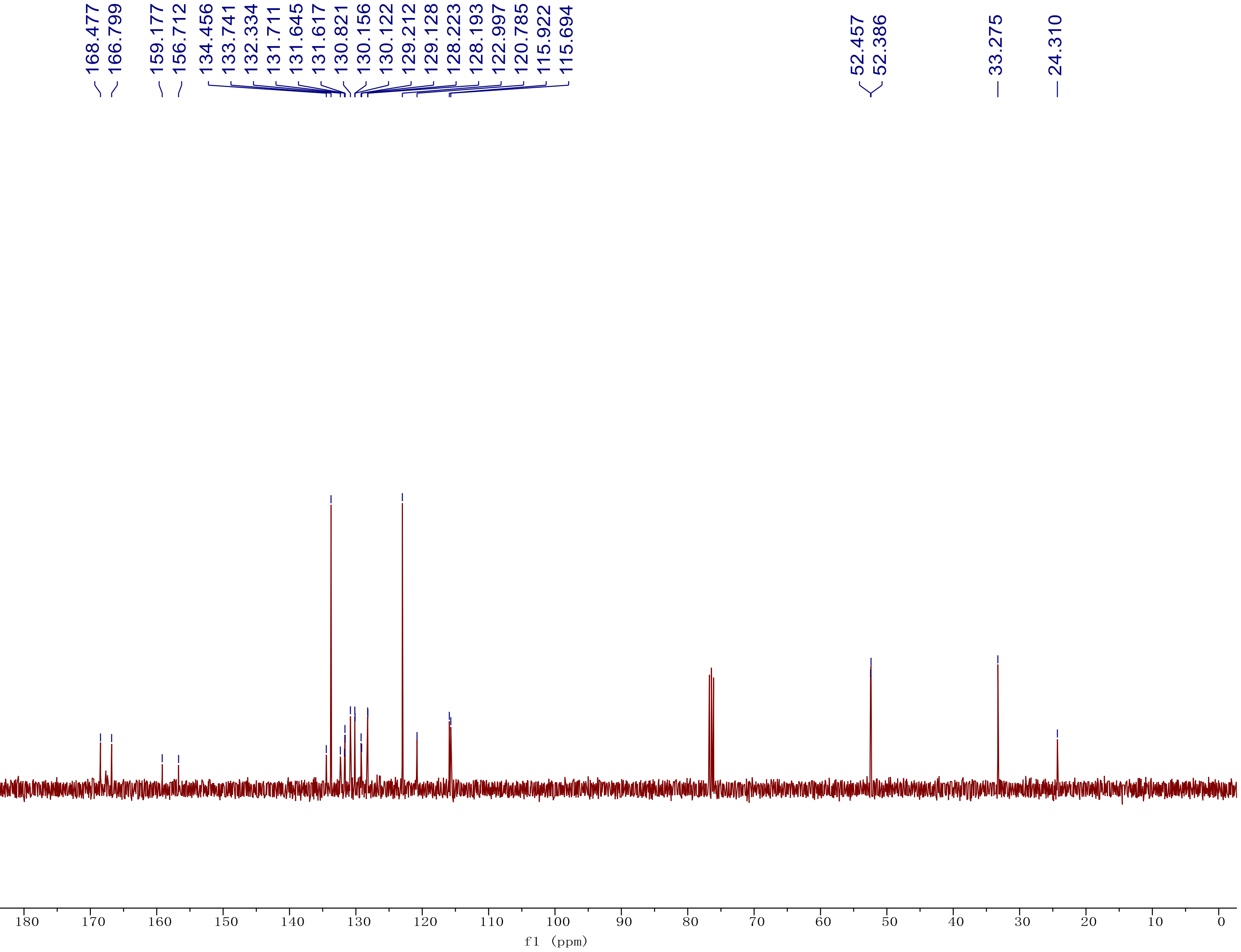

Compound S25 ^{19}F NMR (376 MHz, CDCl$_3$)

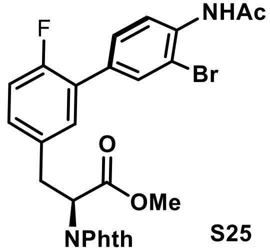

S25

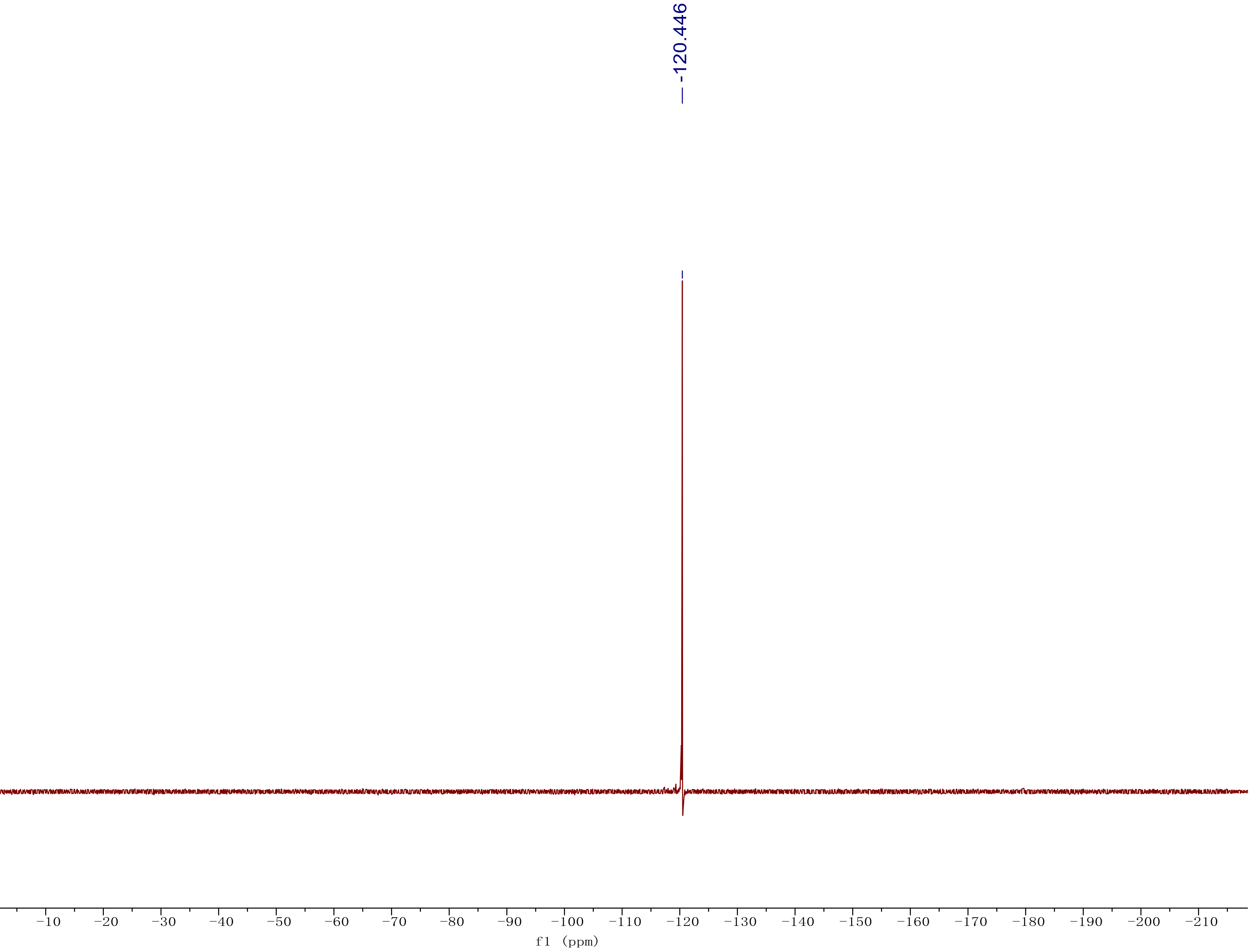

Compound 68 ^{1}H NMR (400 MHz, $CDCl_3$)

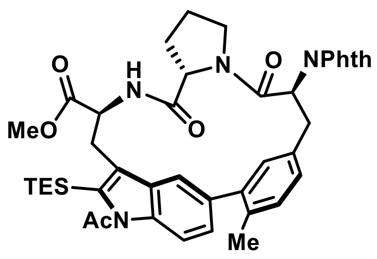

micitide 982 analog (**68**)

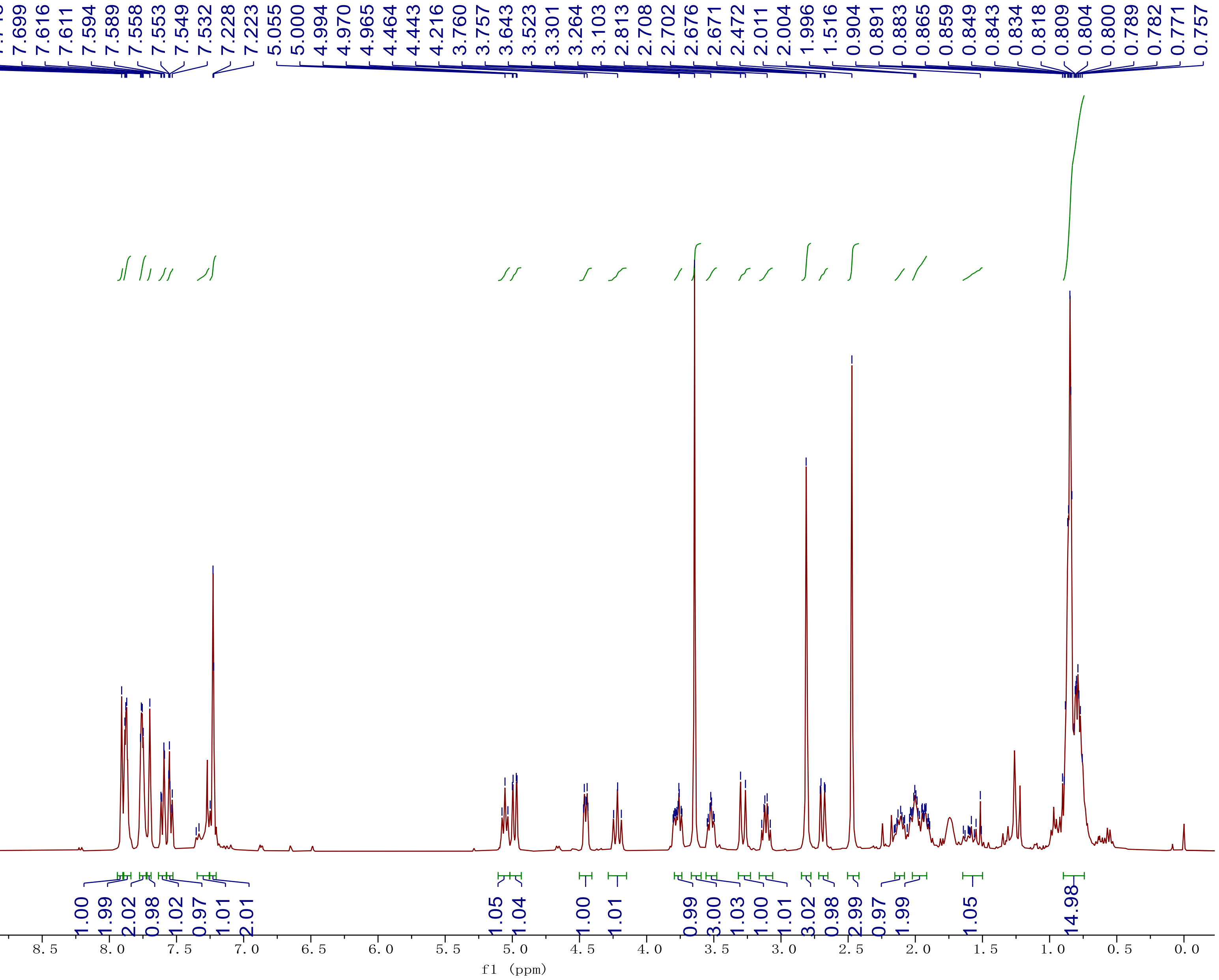

Compound 68 ^{13}C NMR (101 MHz, CDCl$_3$)

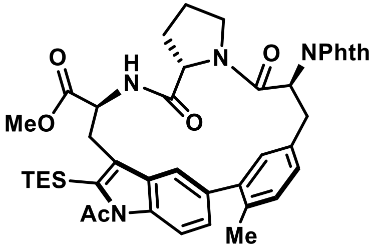

micitide 982 analog (**68**)

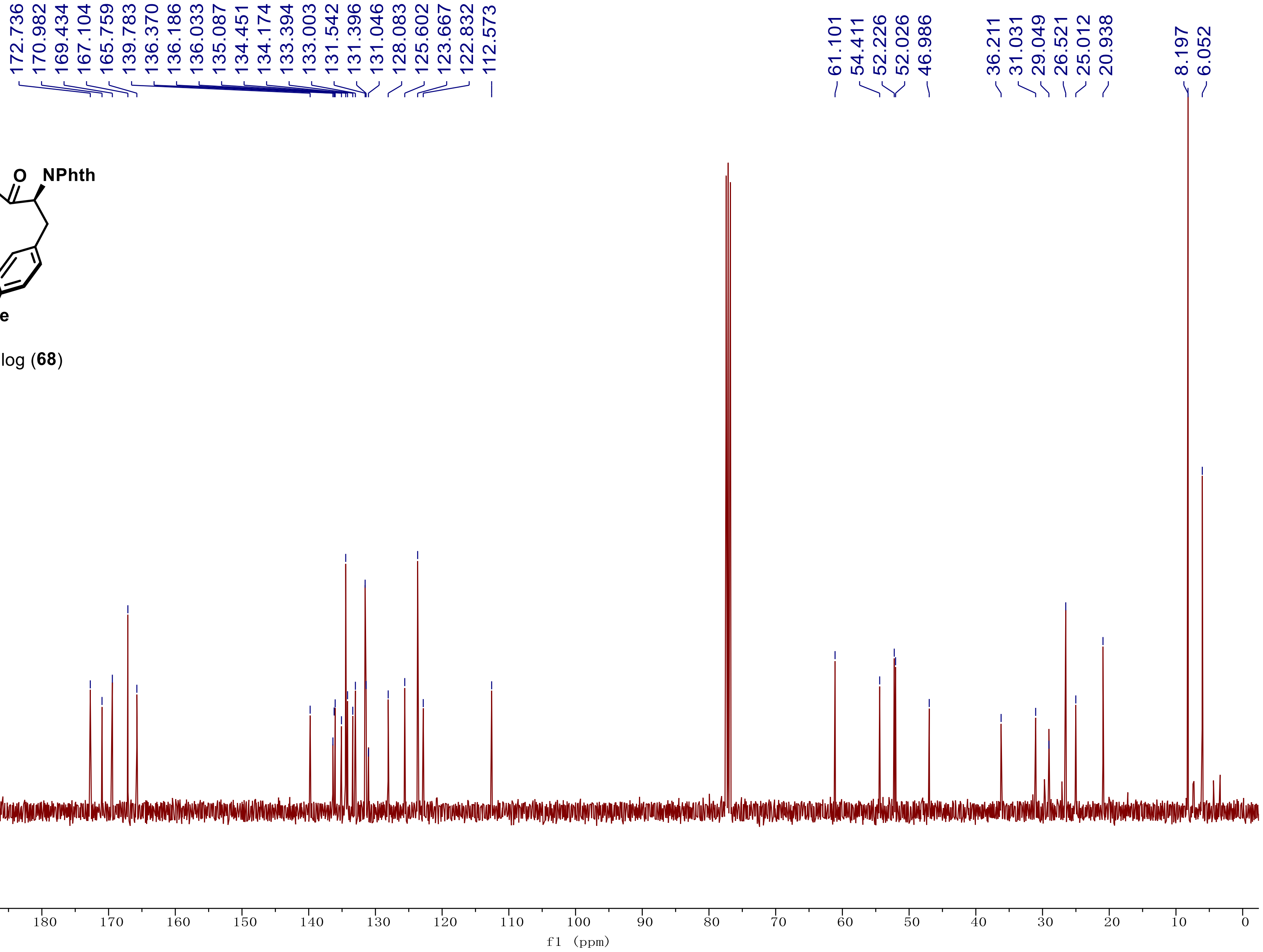

Compound 68 ^{1}H-^{1}H COSY NMR (400 MHz, $CDCl_3$)

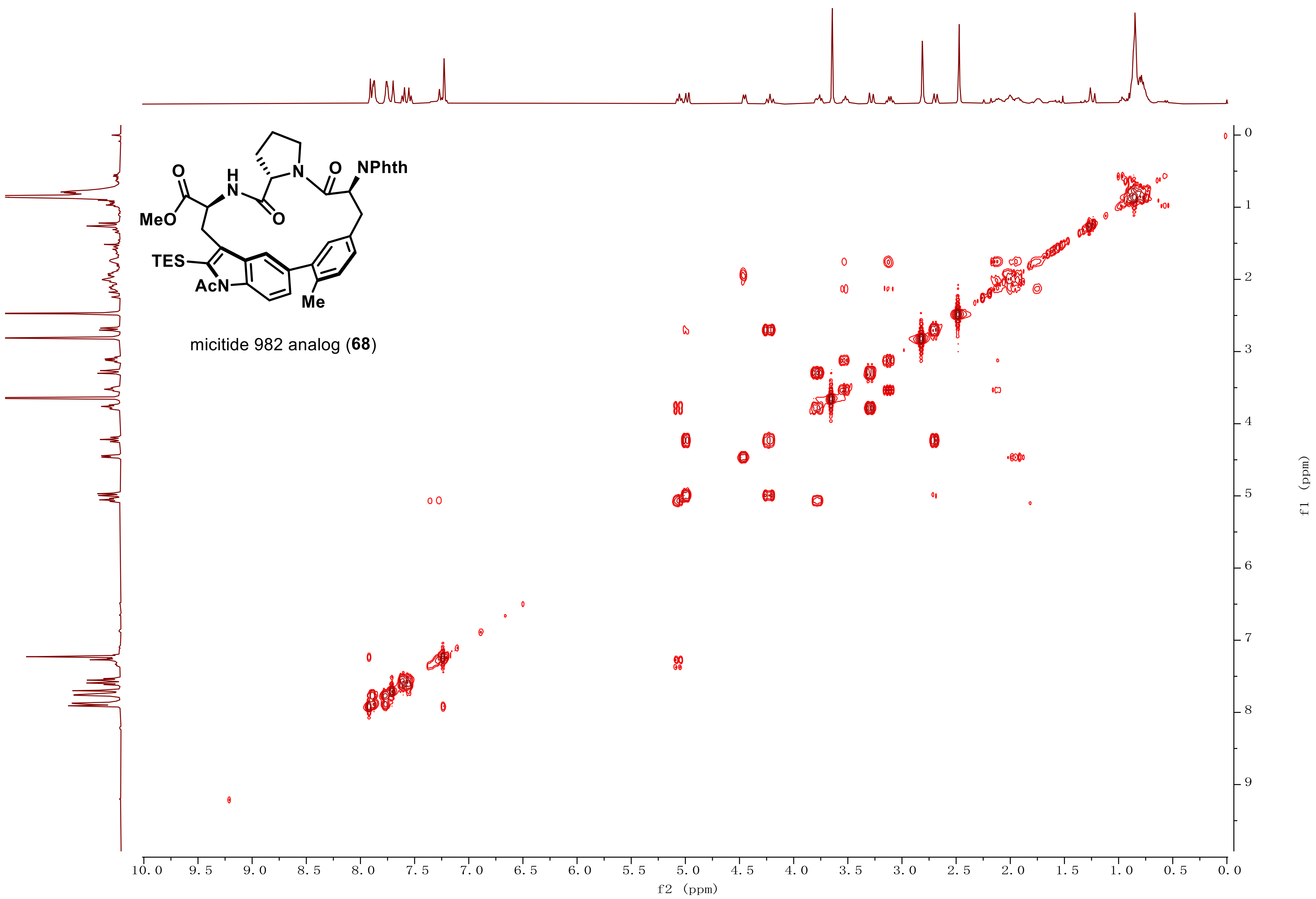

Compound 68 ^{1}H-^{13}C HSQC NMR (400 MHz, $CDCl_3$)

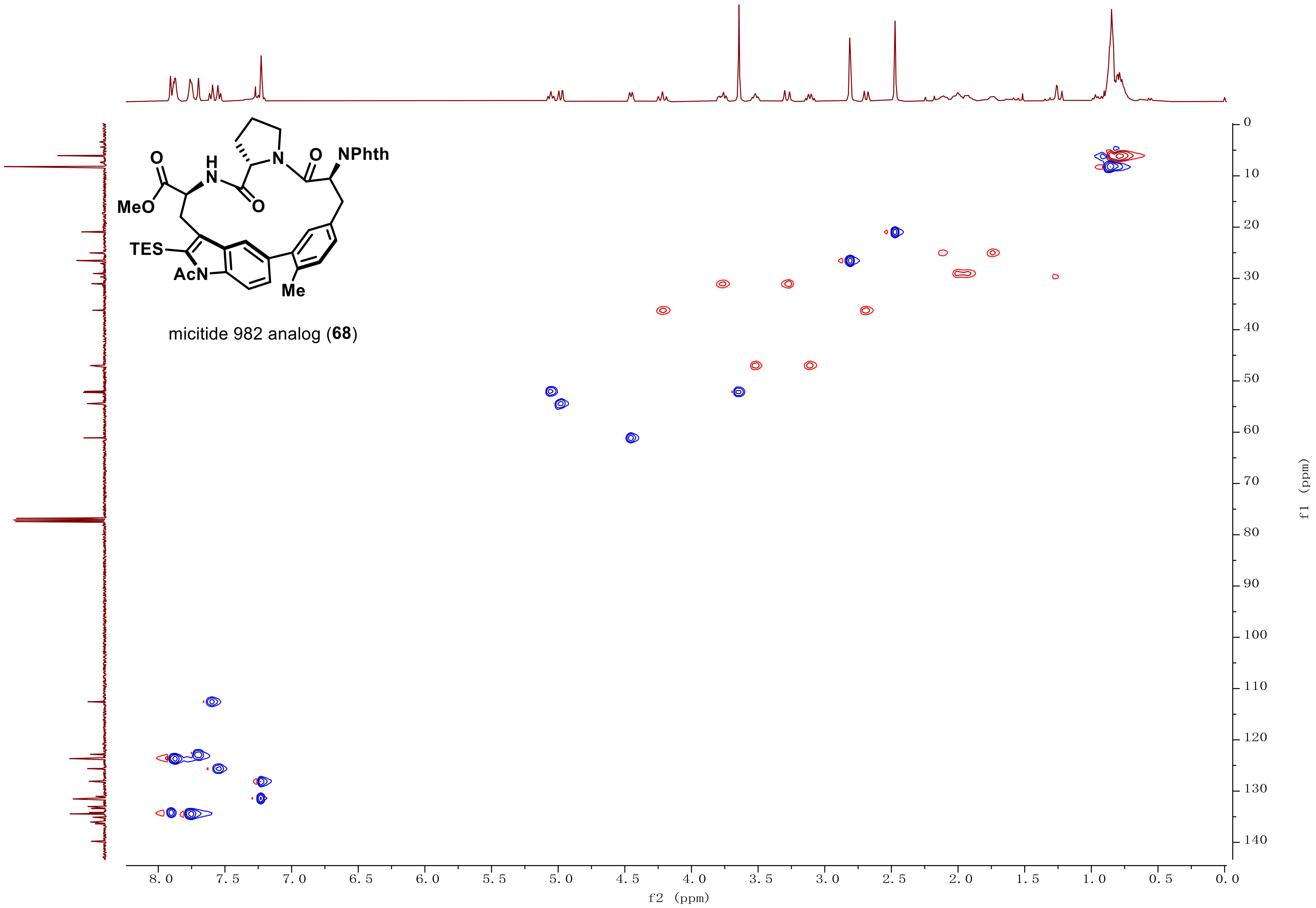

Compound 68 ^{1}H-^{1}H ROESY NMR (400 MHz, CDCl$_3$)

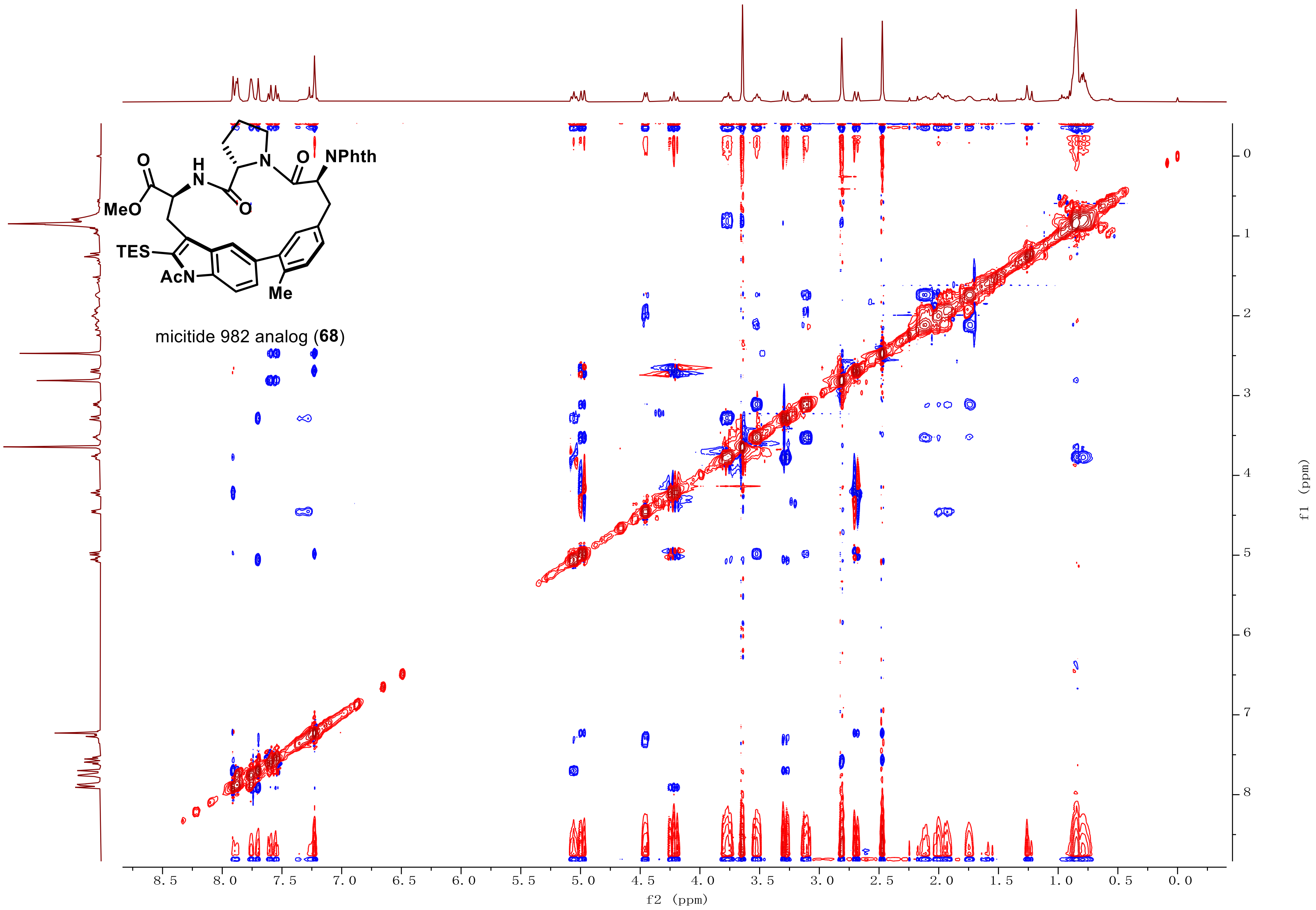

Compound 69 ^{1}H NMR (400 MHz, CDCl$_3$)

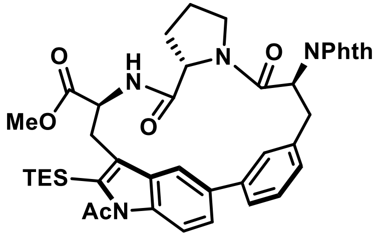

micitide 982 analog (**69**)

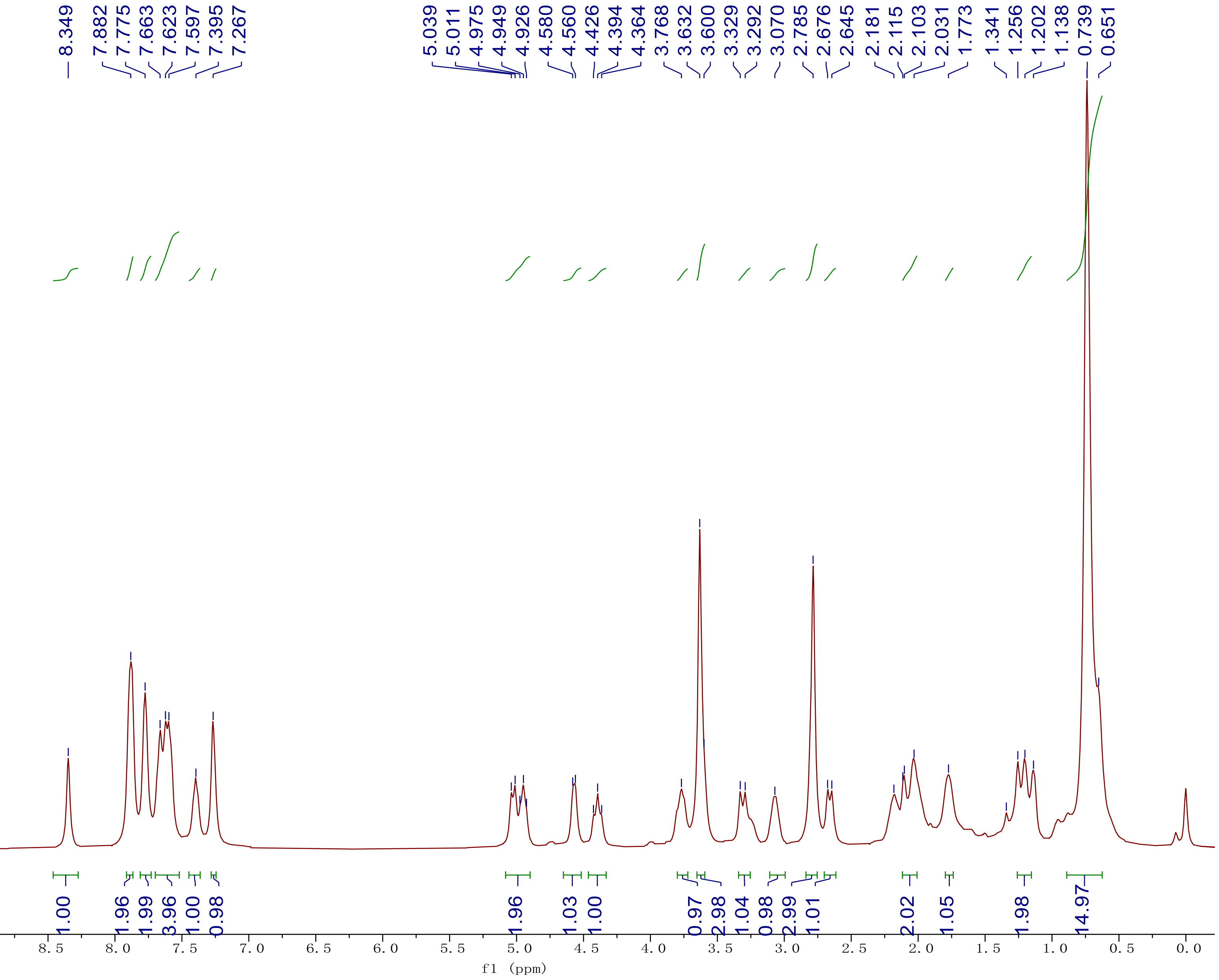

Compound 69 ^{13}C NMR (101 MHz, CDCl$_3$)

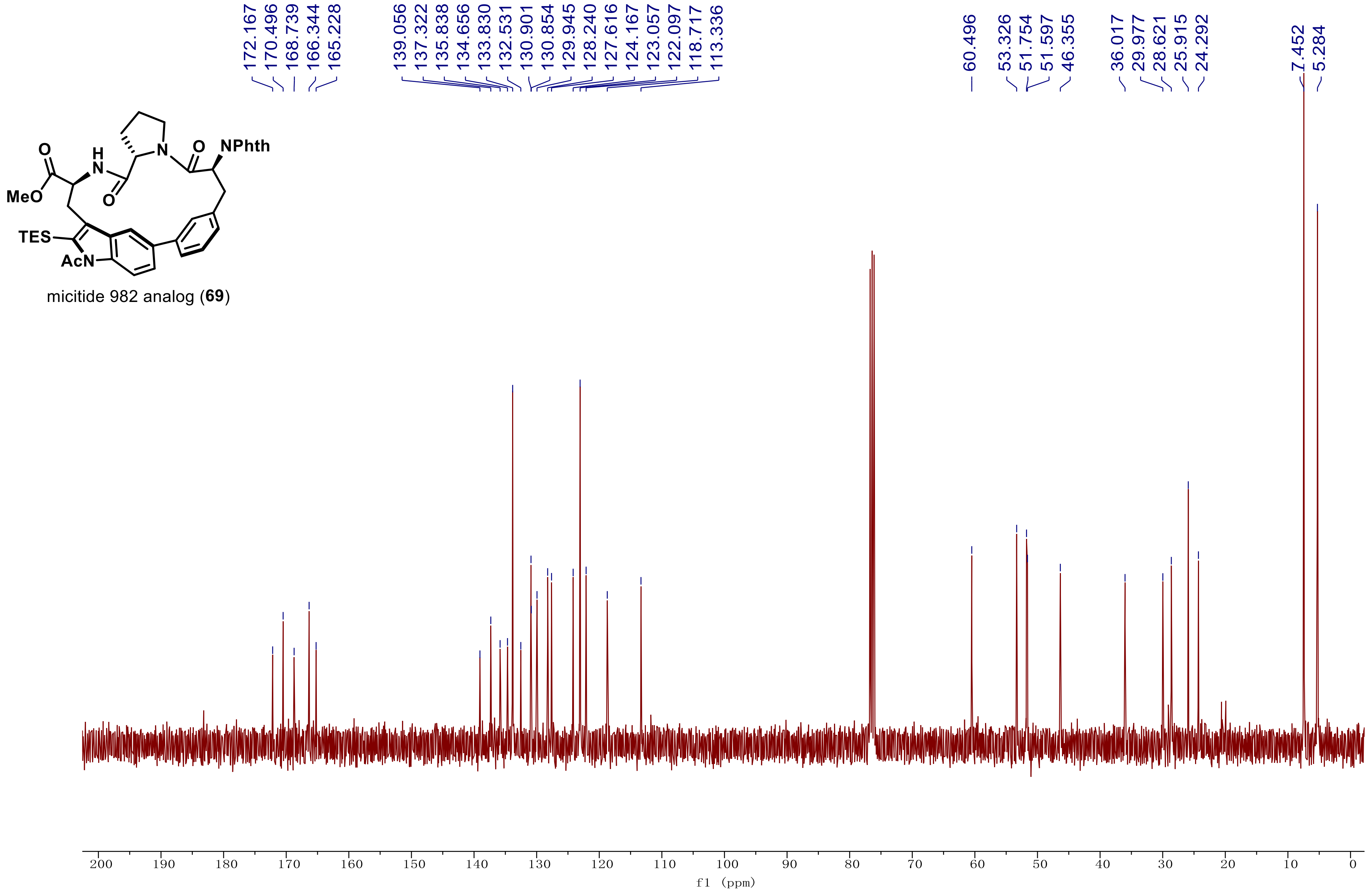

micitide 982 analog (**69**)

Compound 70 ^{1}H NMR (600 MHz, $CDCl_3$)

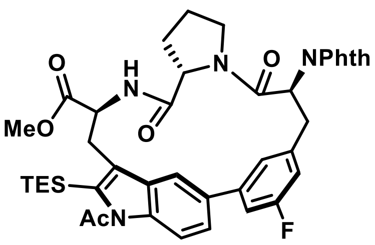

fluoro-micitide 982 analog (**70**)

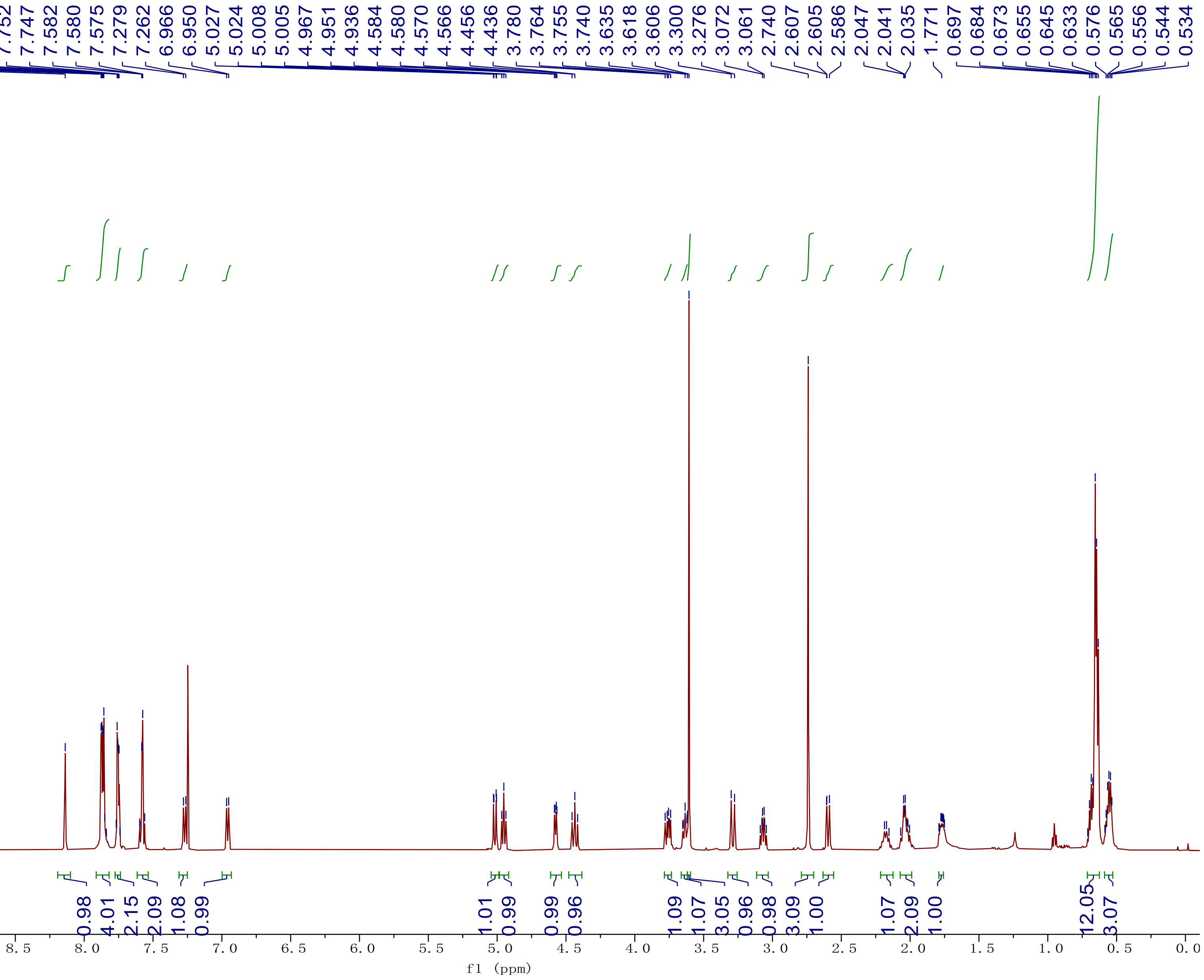

Compound 70 ^{13}C NMR (101 MHz, $CDCl_3$)

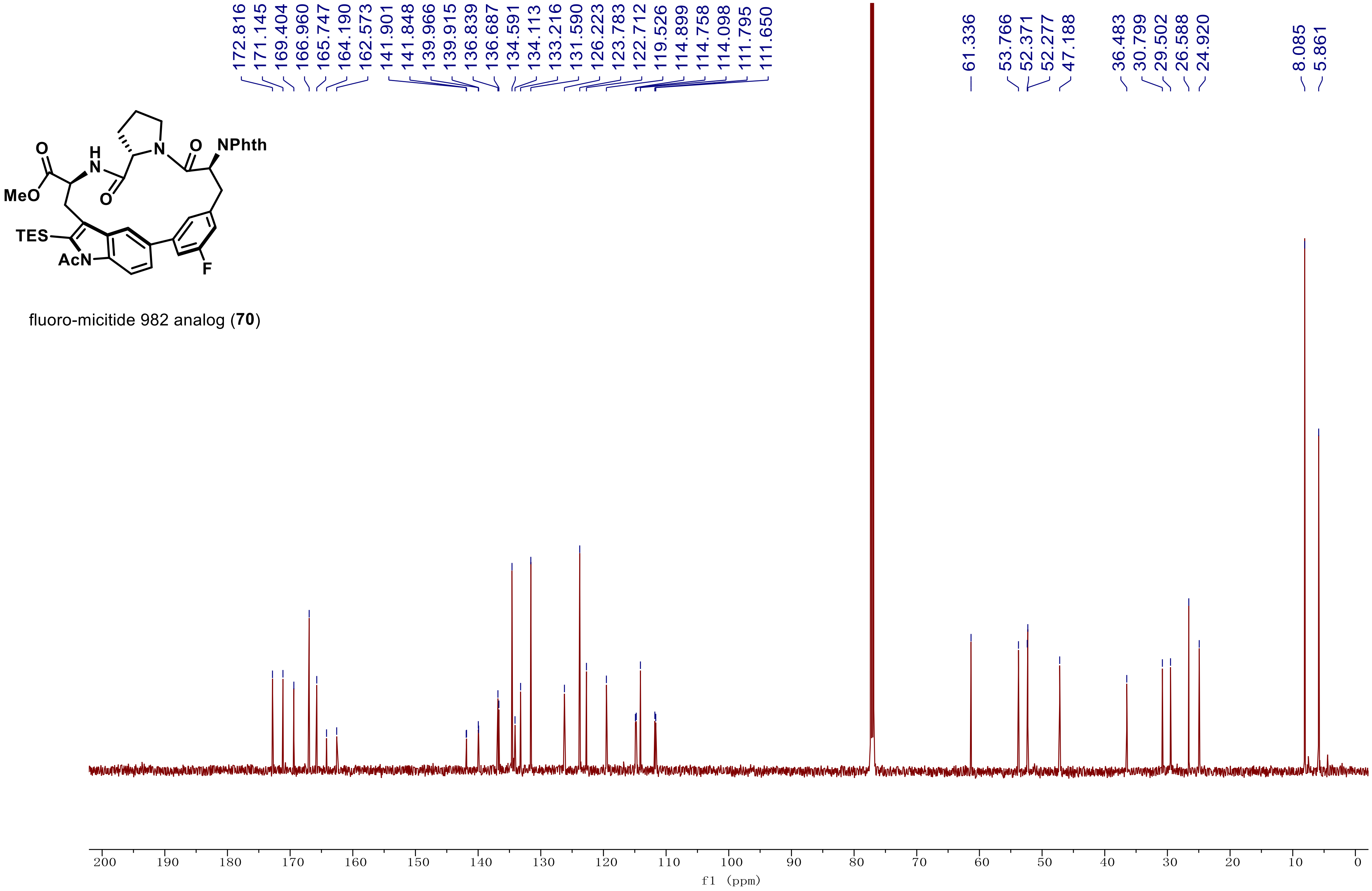

fluoro-micitide 982 analog (**70**)

Compound 70 ^{13}C NMR (376 MHz, CDCl$_3$)

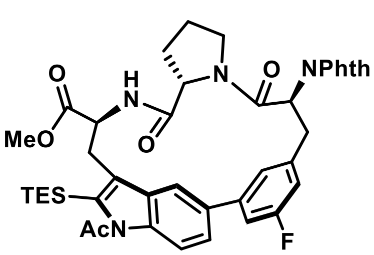

fluoro-micitide 982 analog (**70**)

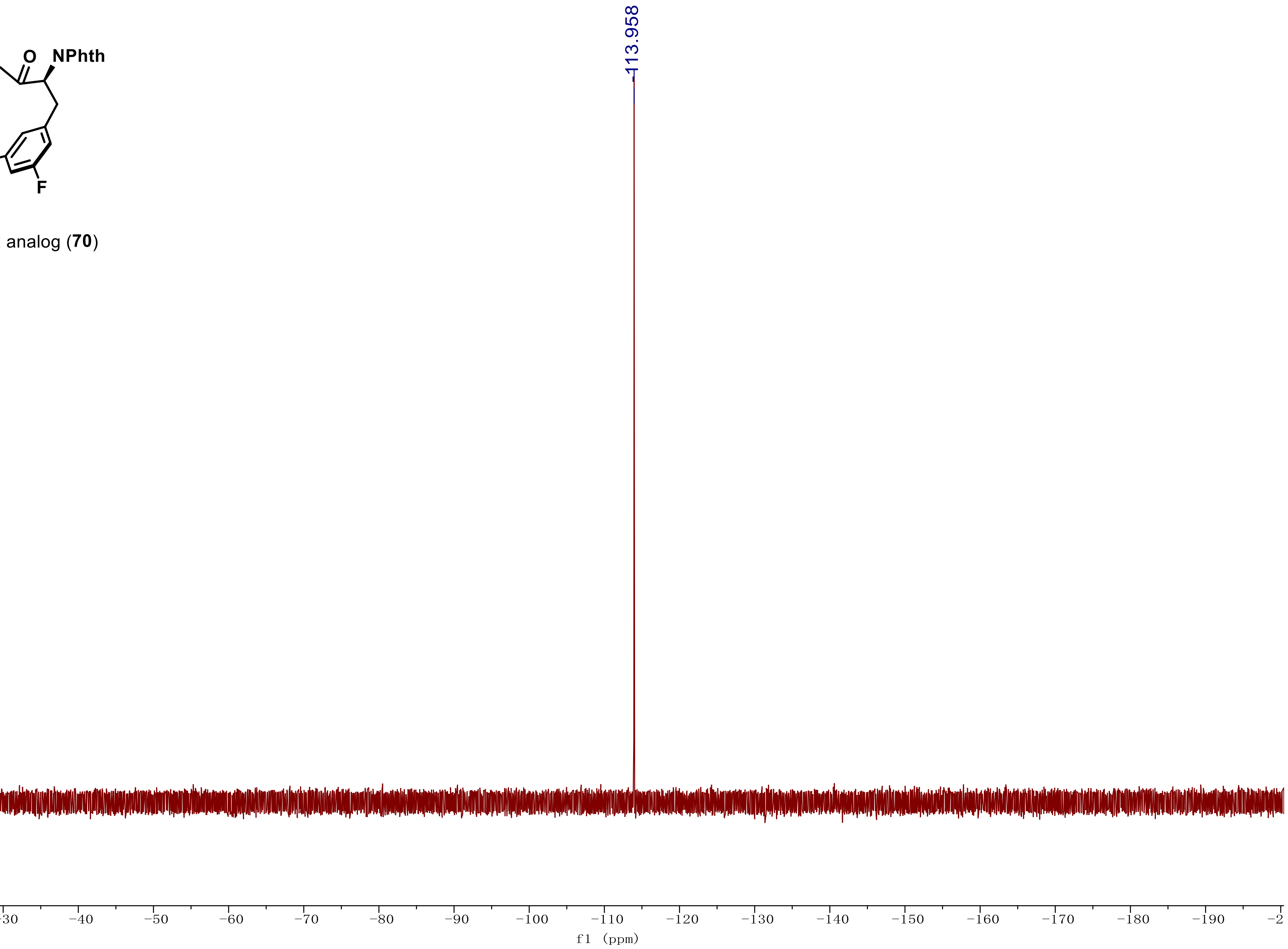

Compound 70 ^{1}H-^{1}H COSY NMR (600 MHz, CDCl$_3$)

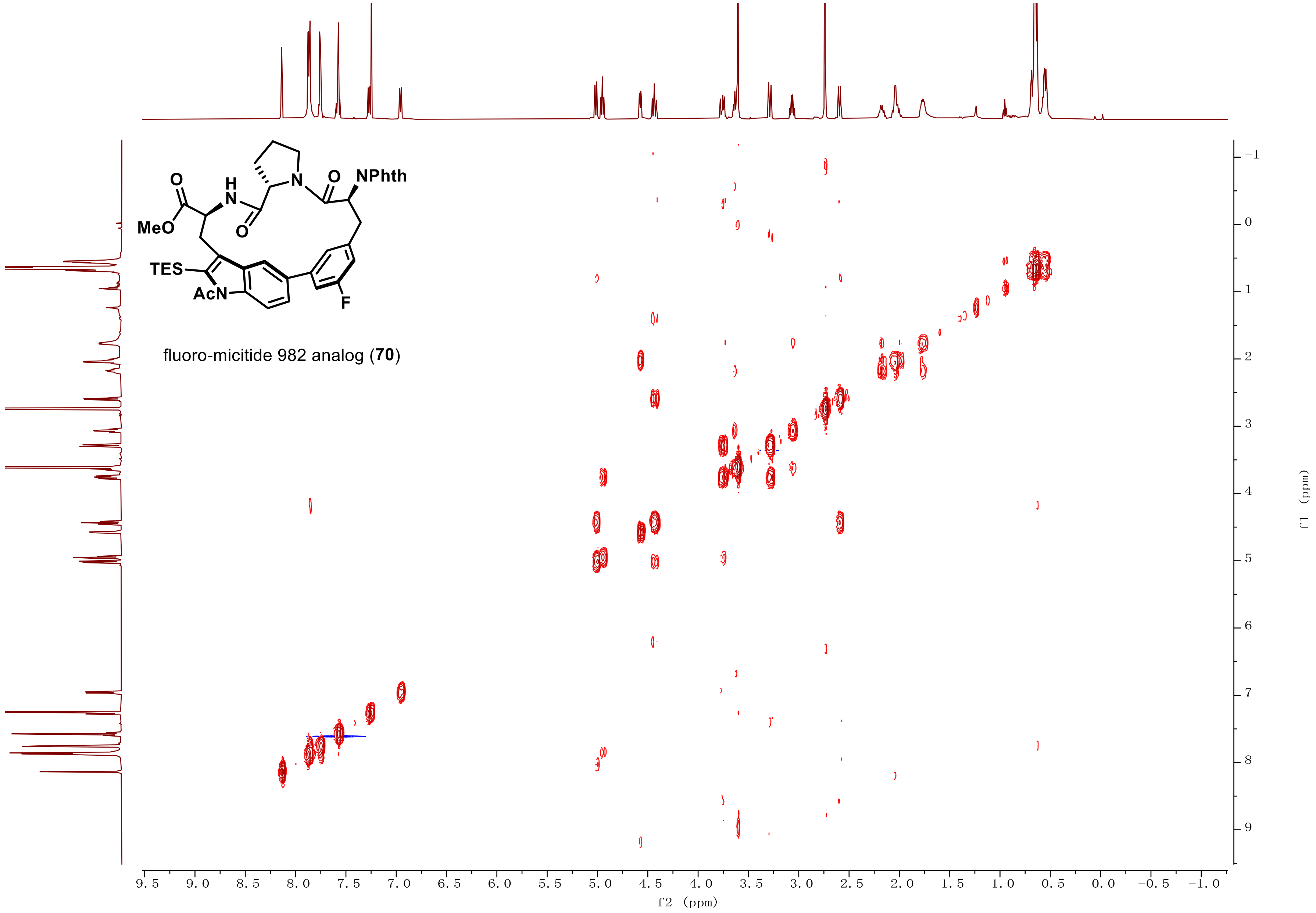

Compound 70 ^{1}H-^{13}C HSQC NMR (400 MHz, $CDCl_3$)

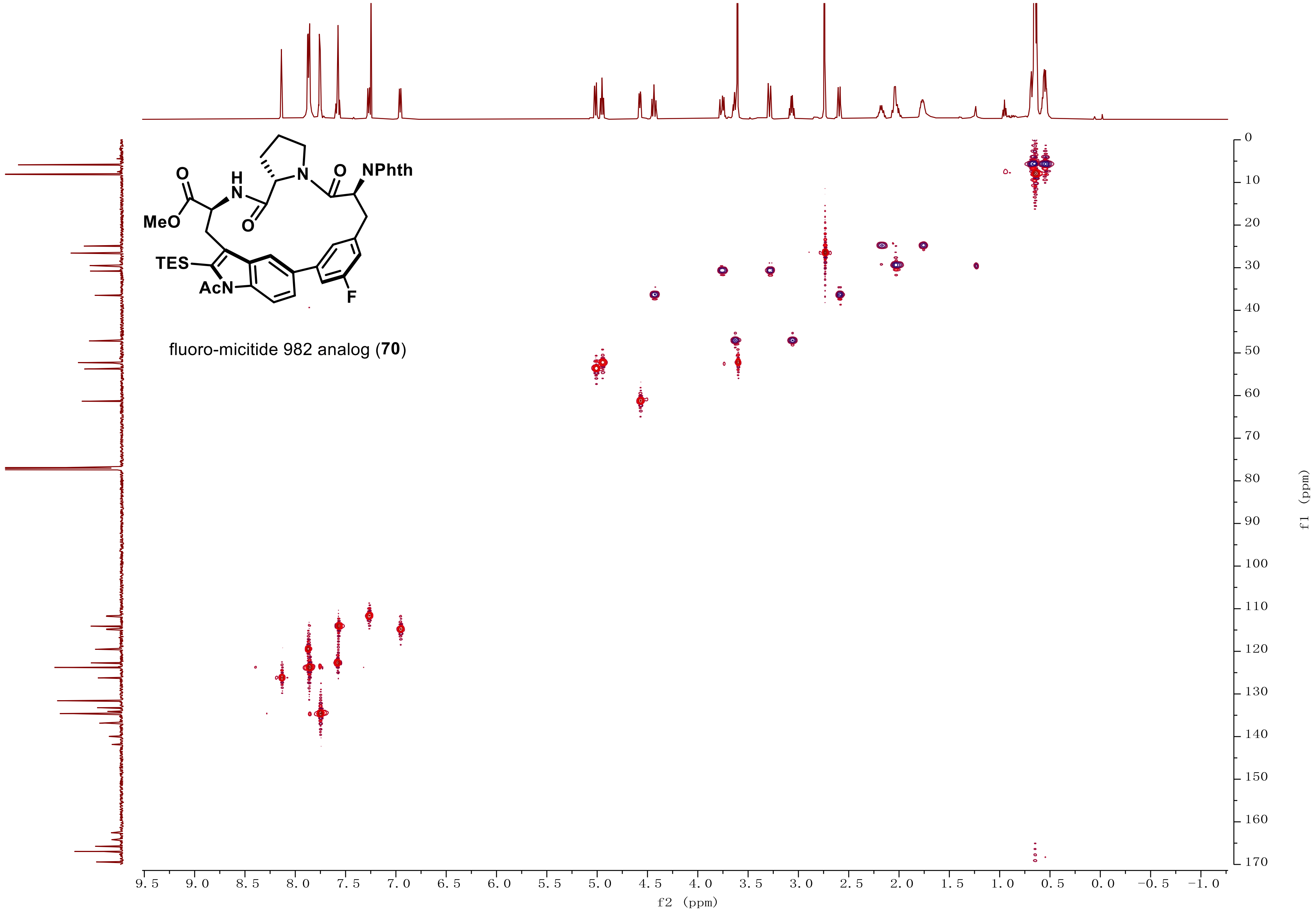

Compound 70 ^{1}H-^{1}H NOESY NMR (400 MHz, $CDCl_3$)

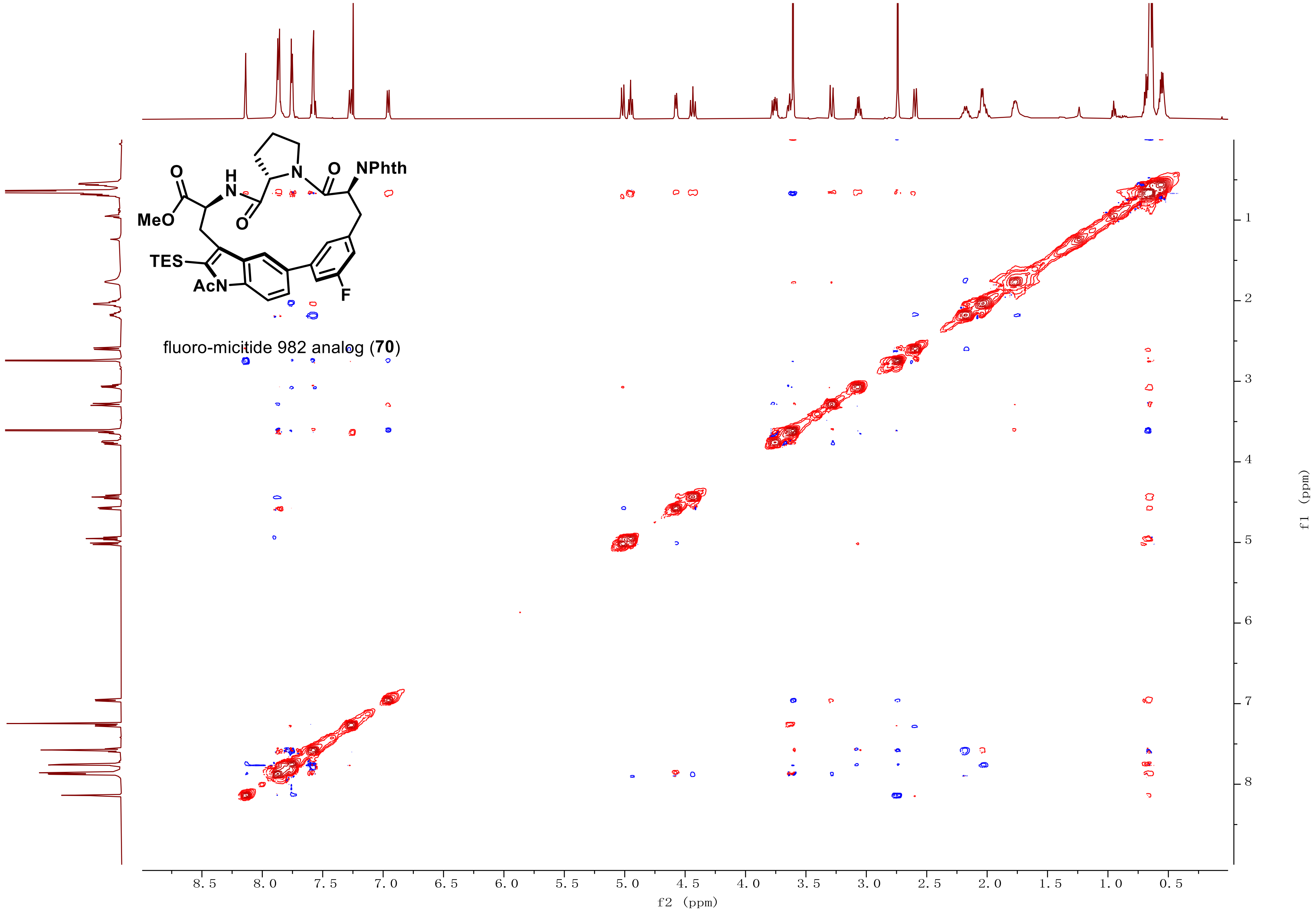

Compound 71 ^{1}H NMR (600 MHz, $CDCl_3$)

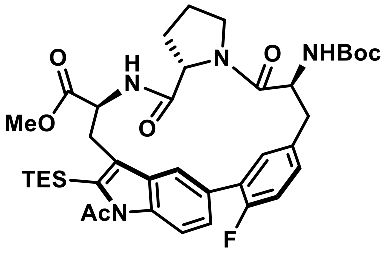

fluoro-micitide 982 analog (**71**)

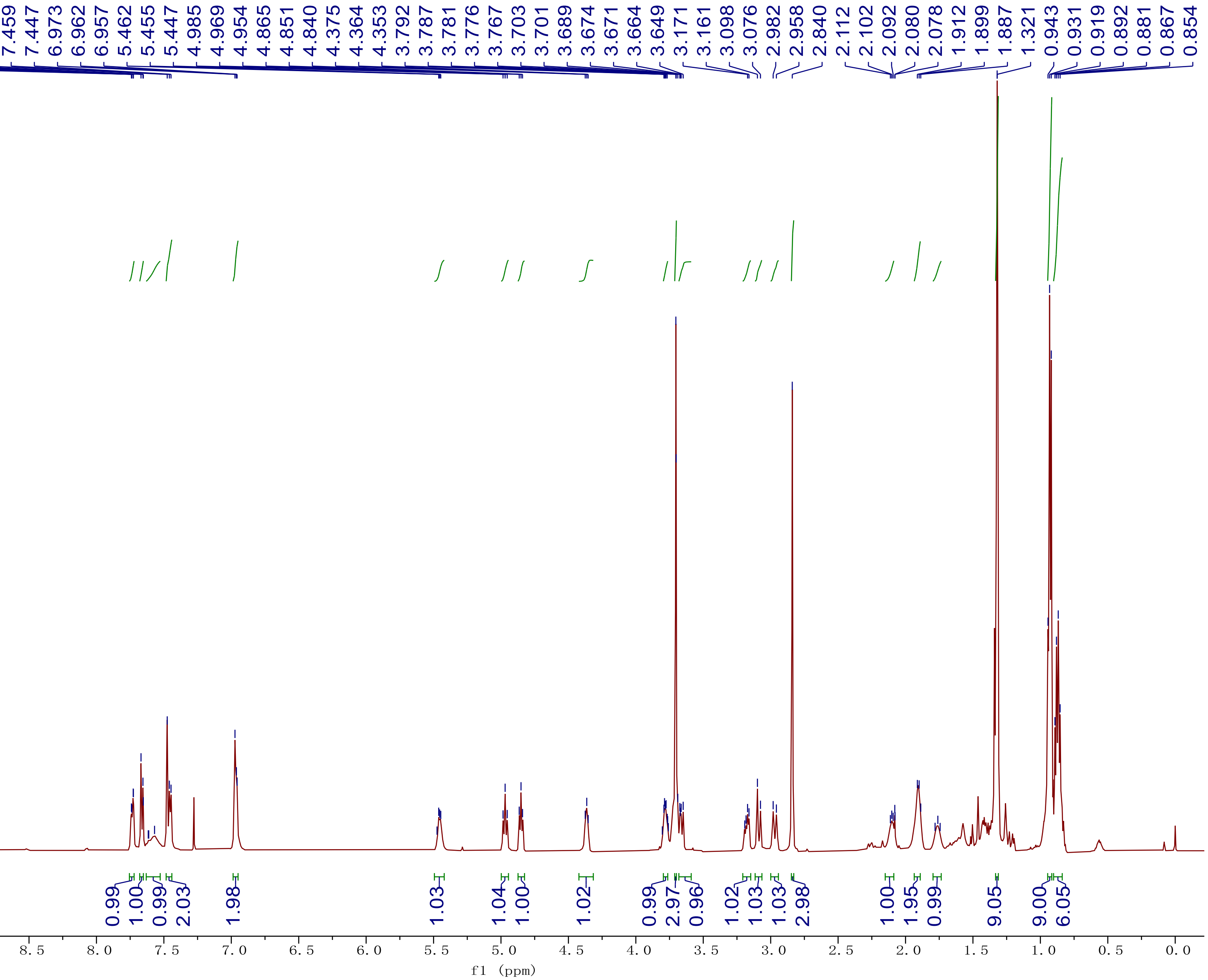

Compound 71 ^{13}C NMR (151 MHz, CDCl$_3$)

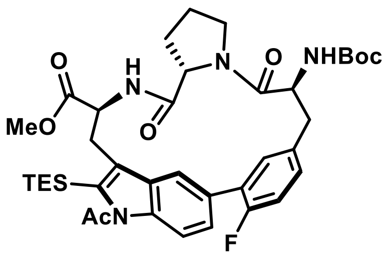

fluoro-micitide 982 analog (**71**)

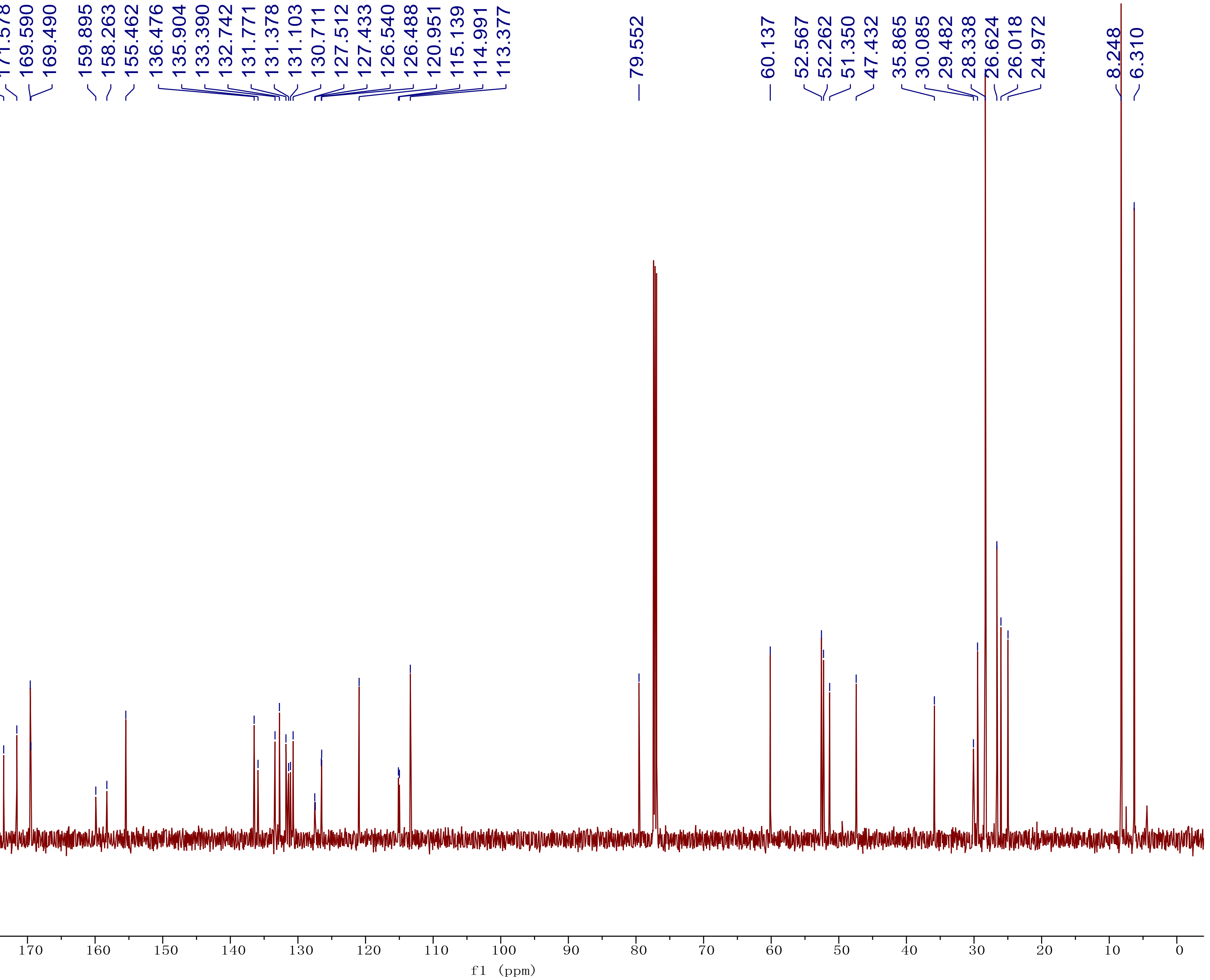

Compound 71 ^{19}F NMR (565 MHz, $CDCl_3$)

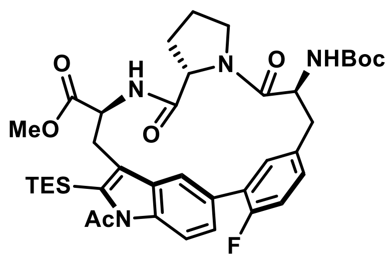

fluoro-micitide 982 analog (**71**)

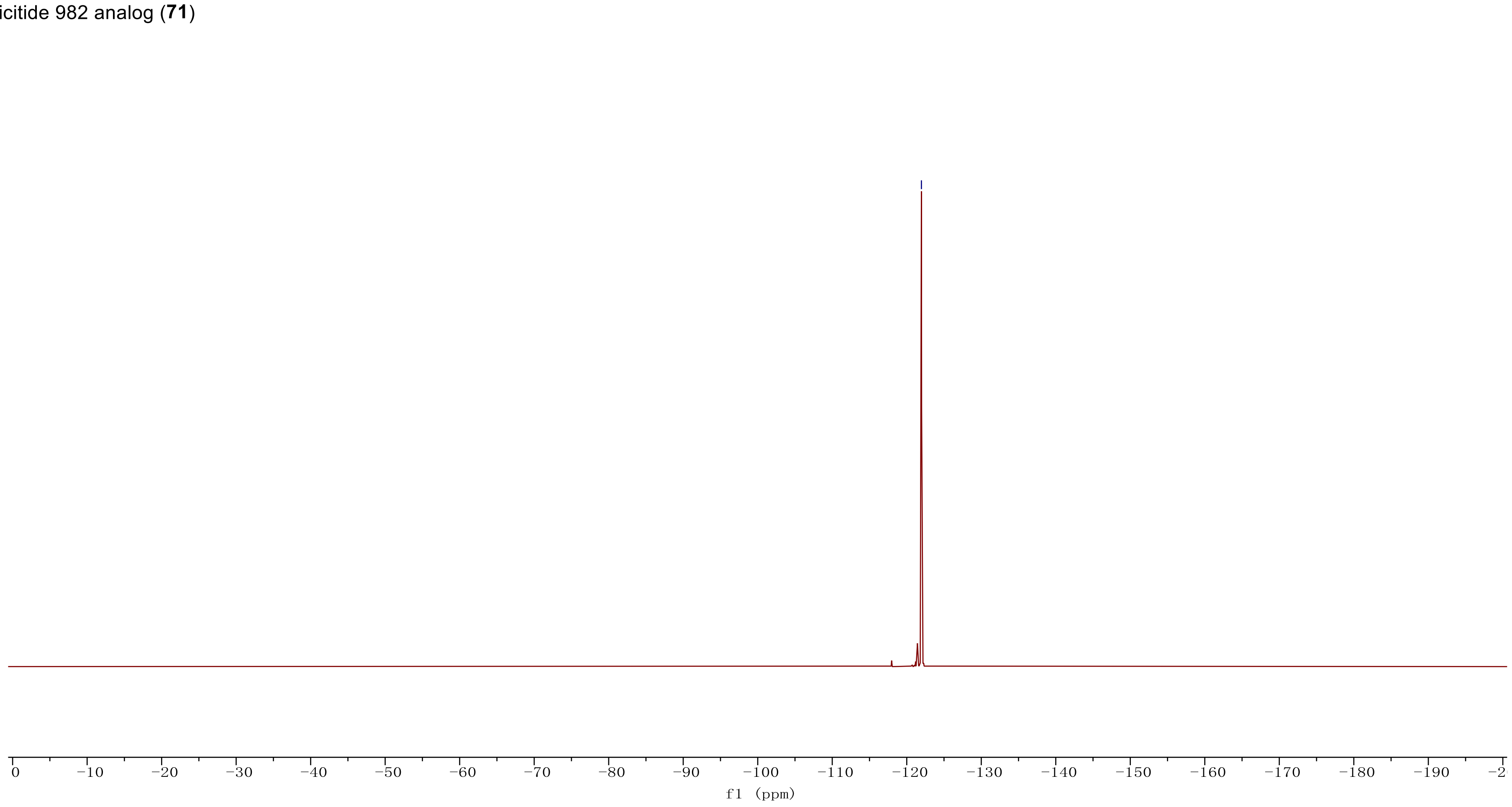

Compound 72 ^{1}H NMR (400 MHz, $CDCl_3$)

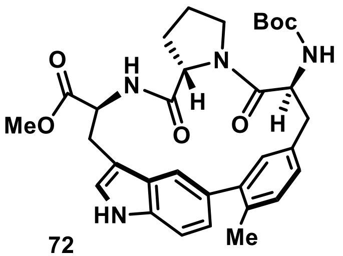

72

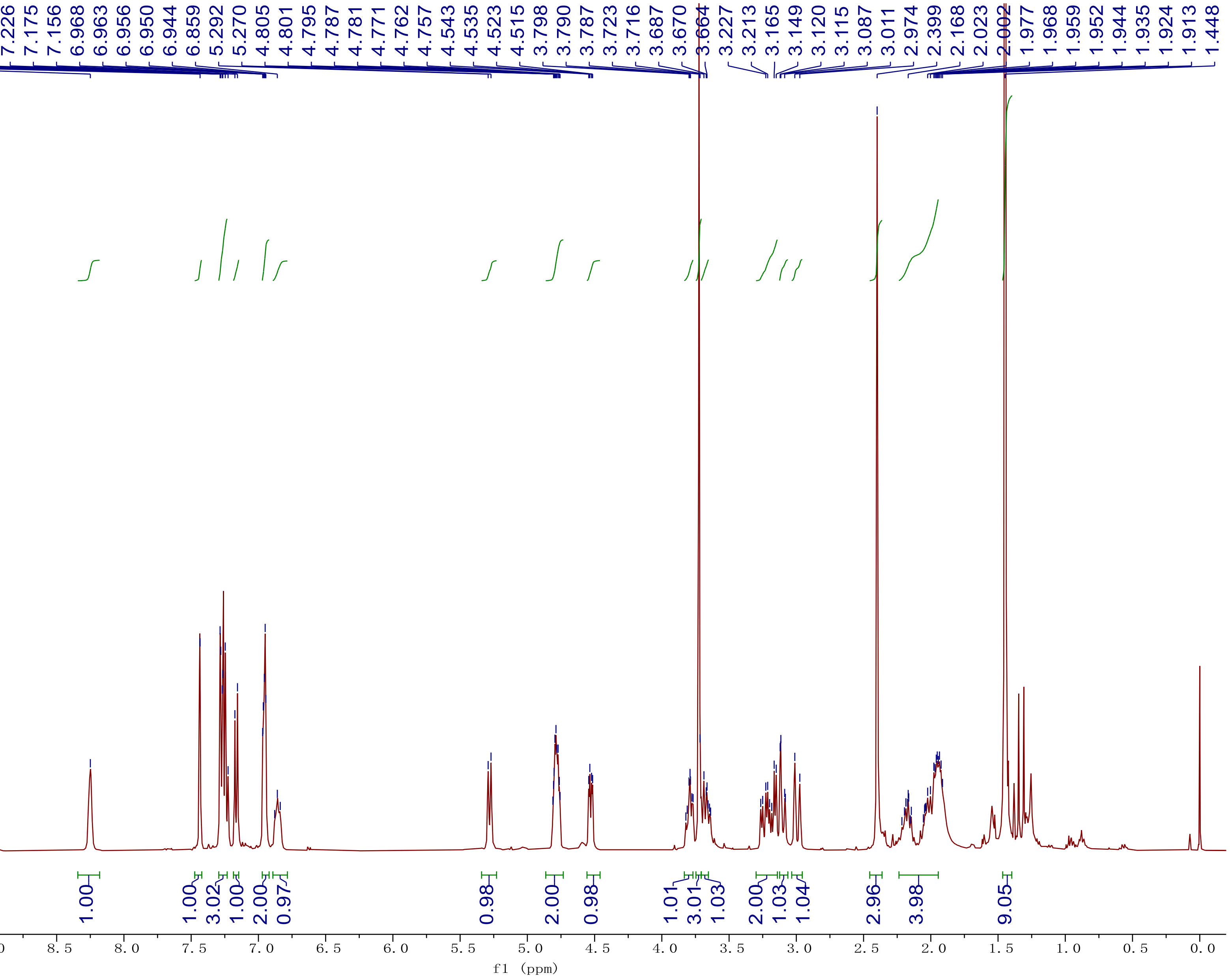

Compound 72 ^{13}C NMR (101 MHz, $CDCl_3$)

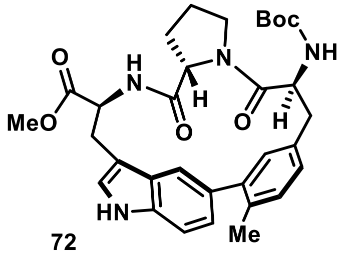

72

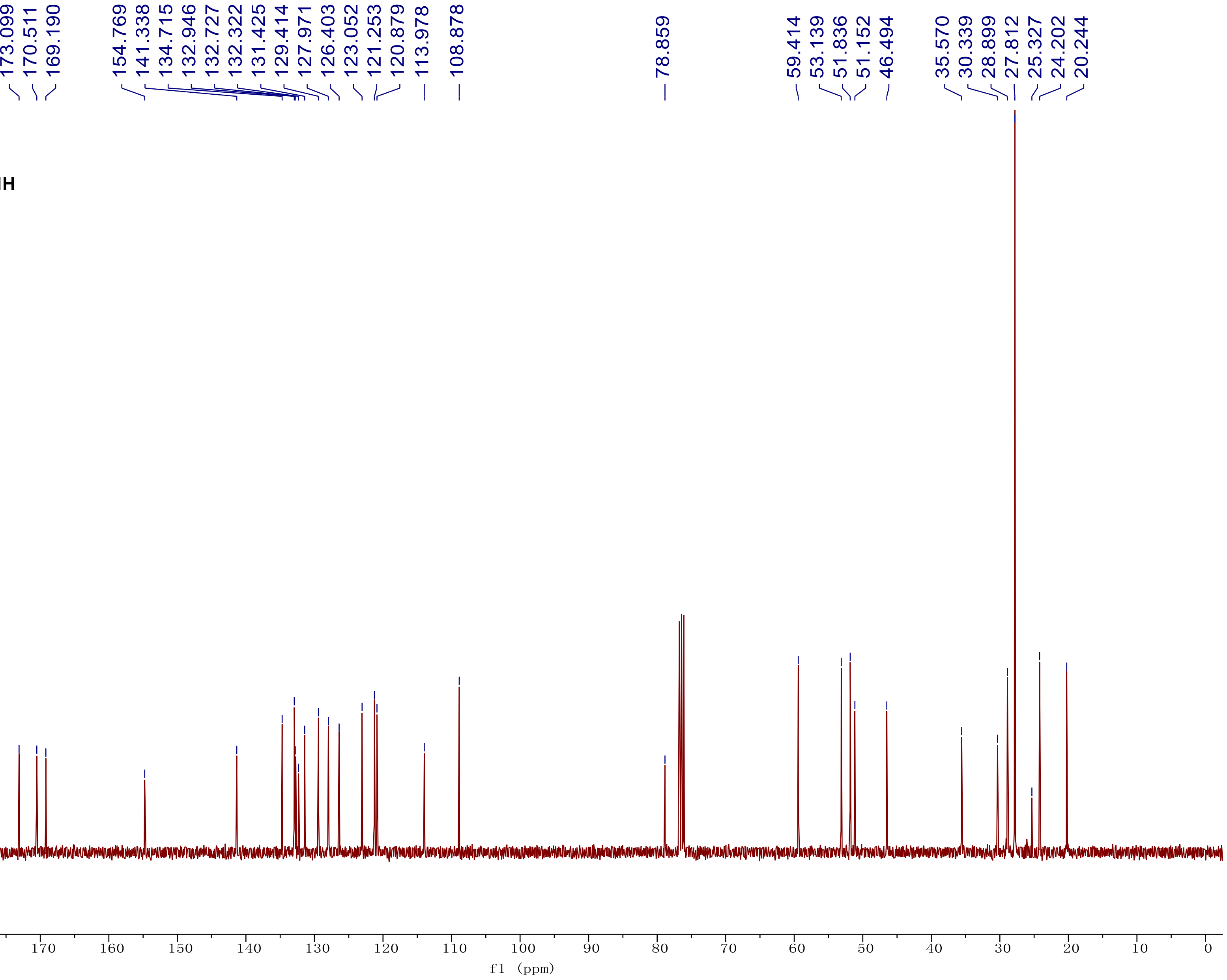

Compound S26 ^{1}H NMR (600 MHz, $CDCl_3$)

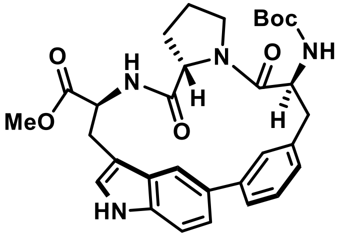

S26

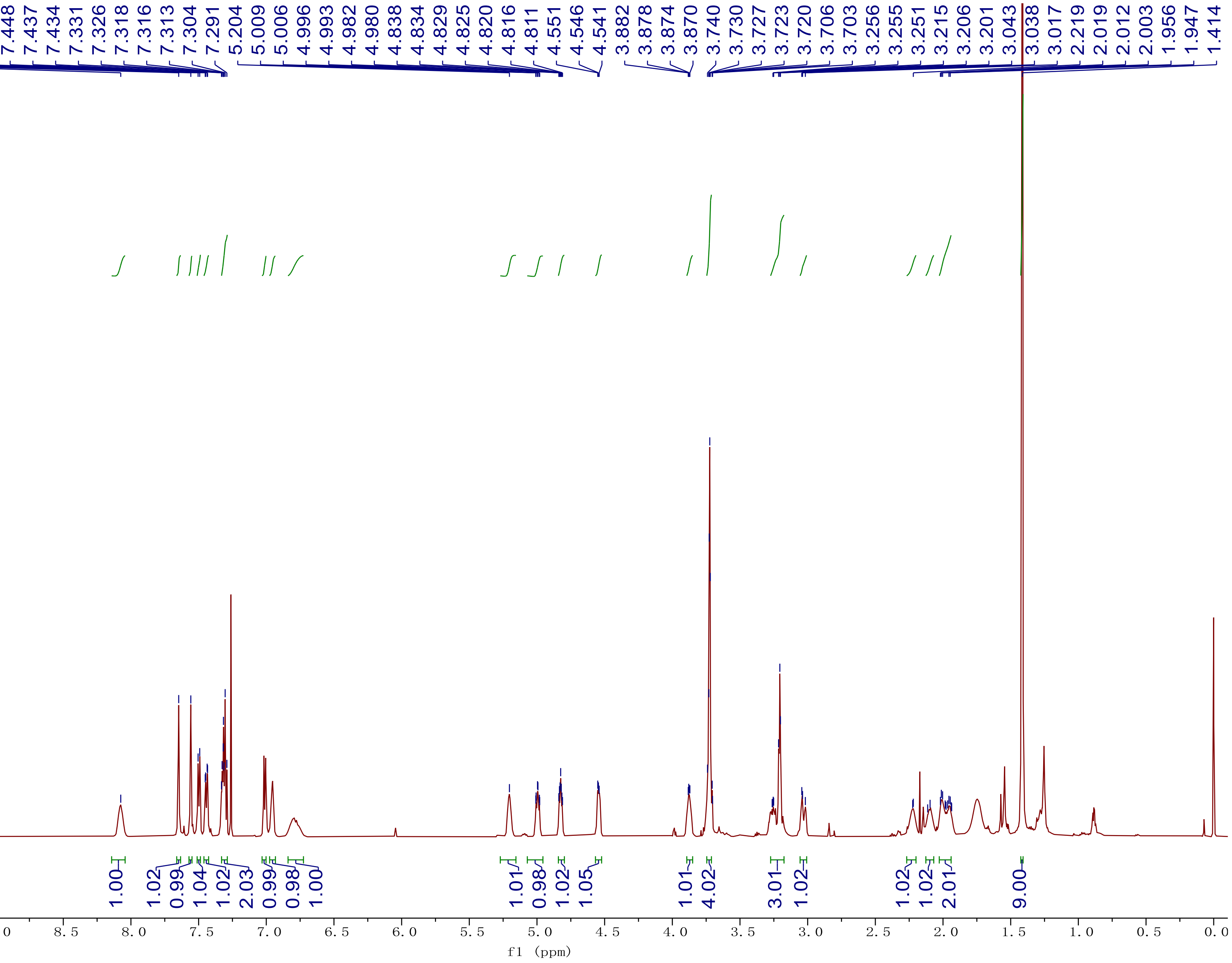

Compound S26 ^{13}C NMR (151 MHz, CDCl$_3$)

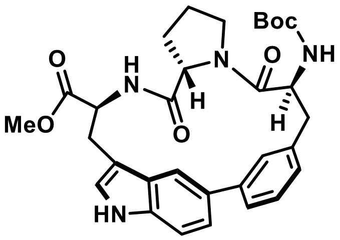

S26

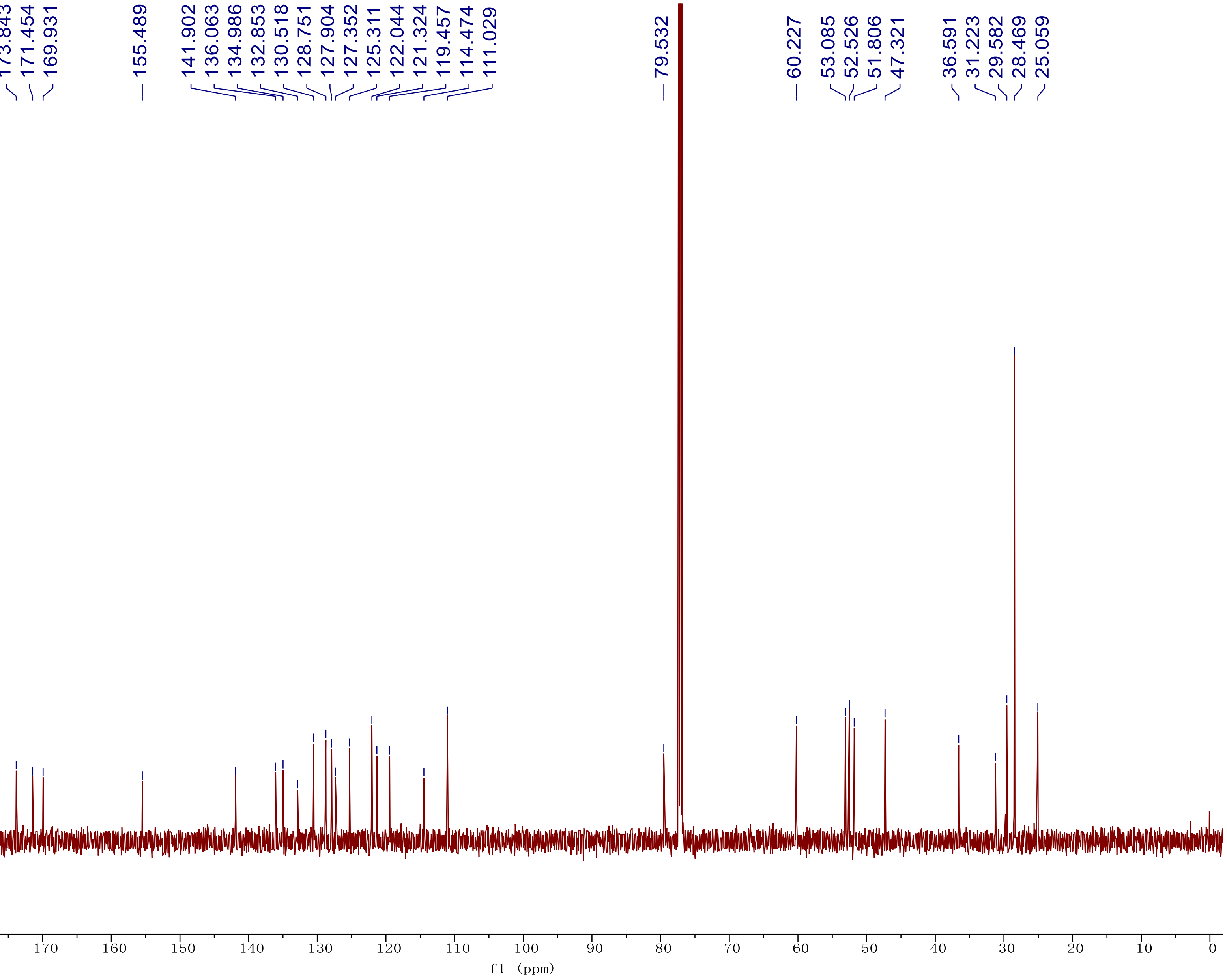

Compound S26 ^1H-^1H COSY NMR (400 MHz, $CDCl_3$)

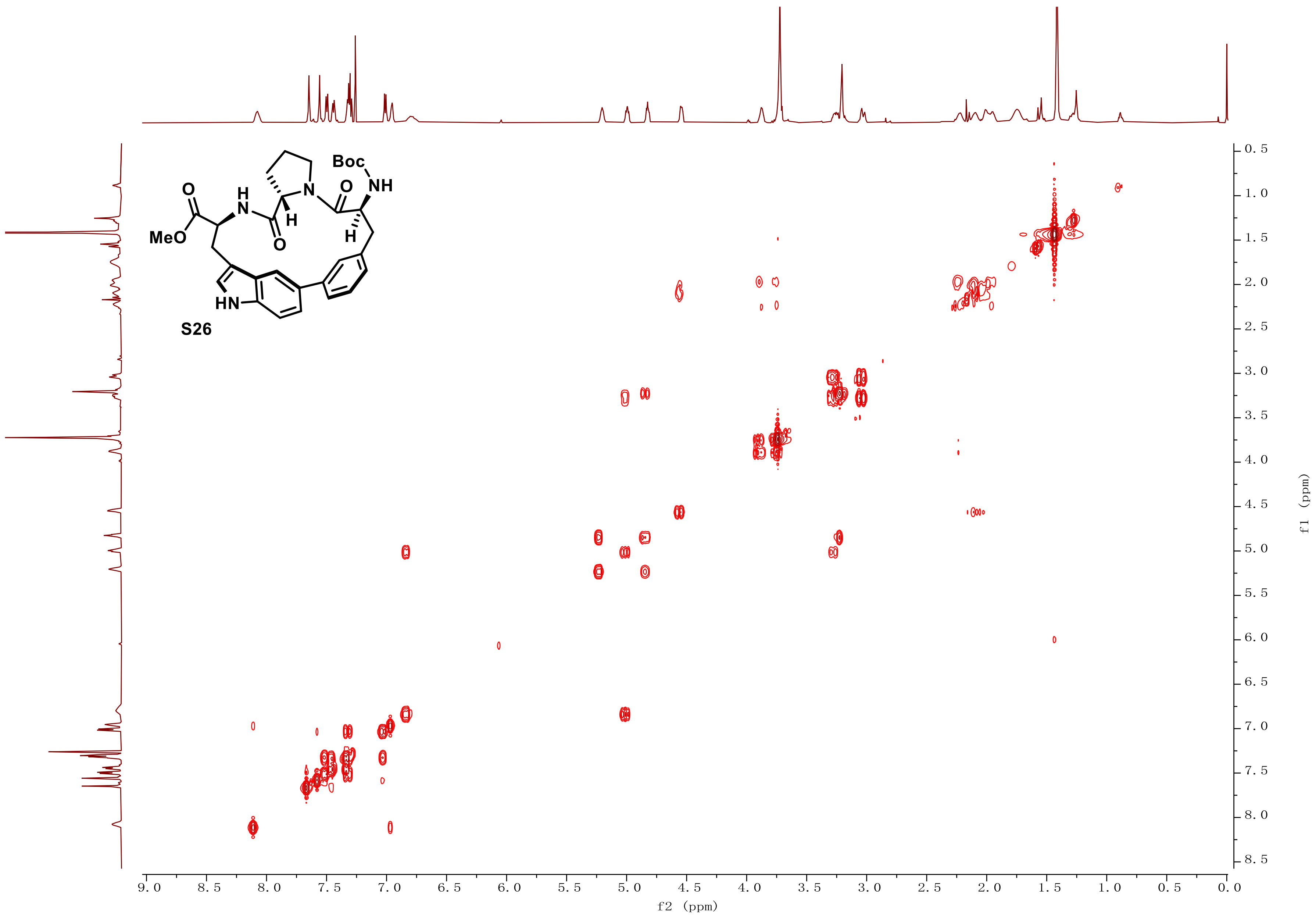

Compound S26 ^{1}H-^{13}C HSQC NMR (400 MHz, $CDCl_3$)

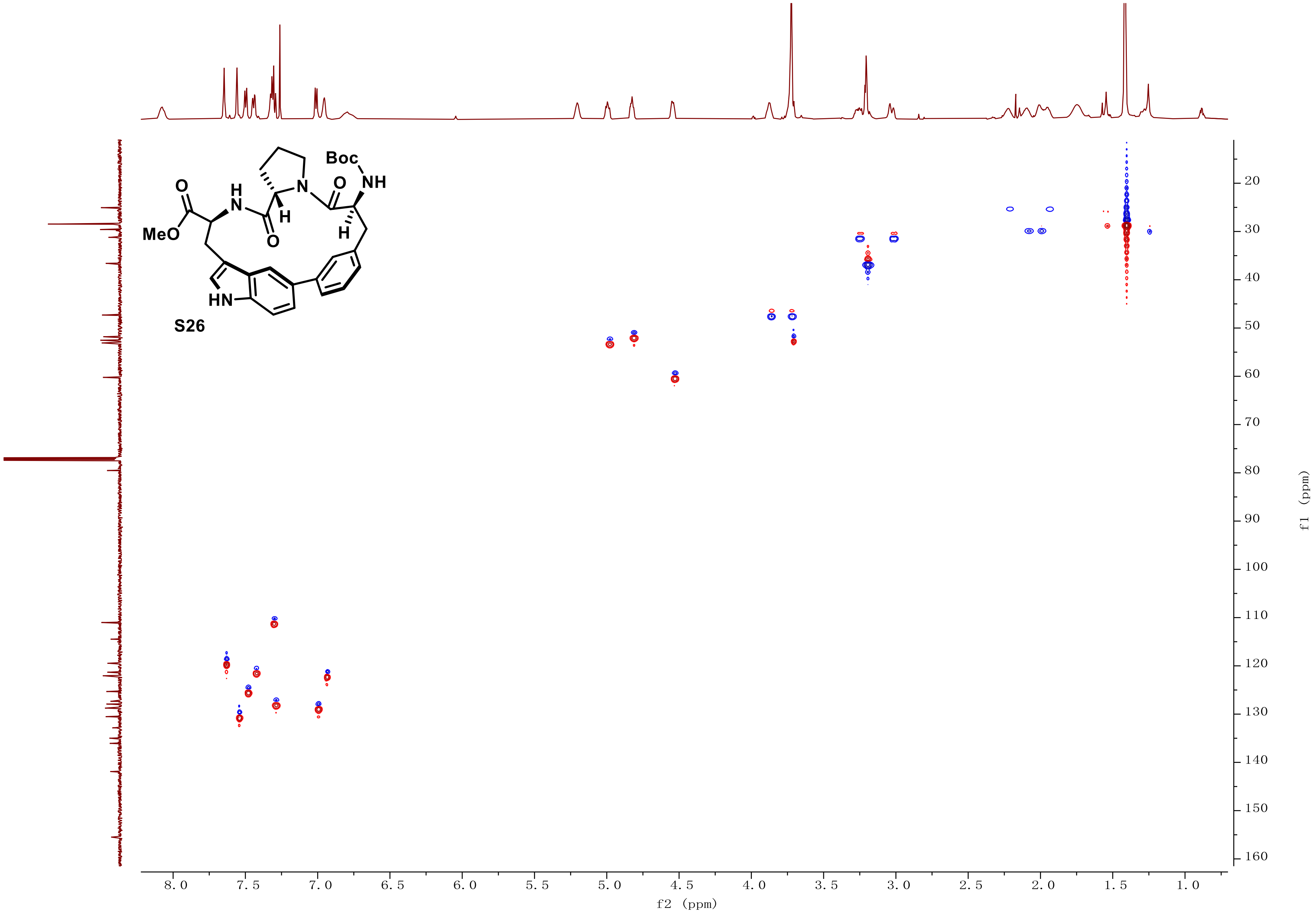

Compound S26 ^{1}H-^{1}H NOESY NMR (400 MHz, $CDCl_3$)

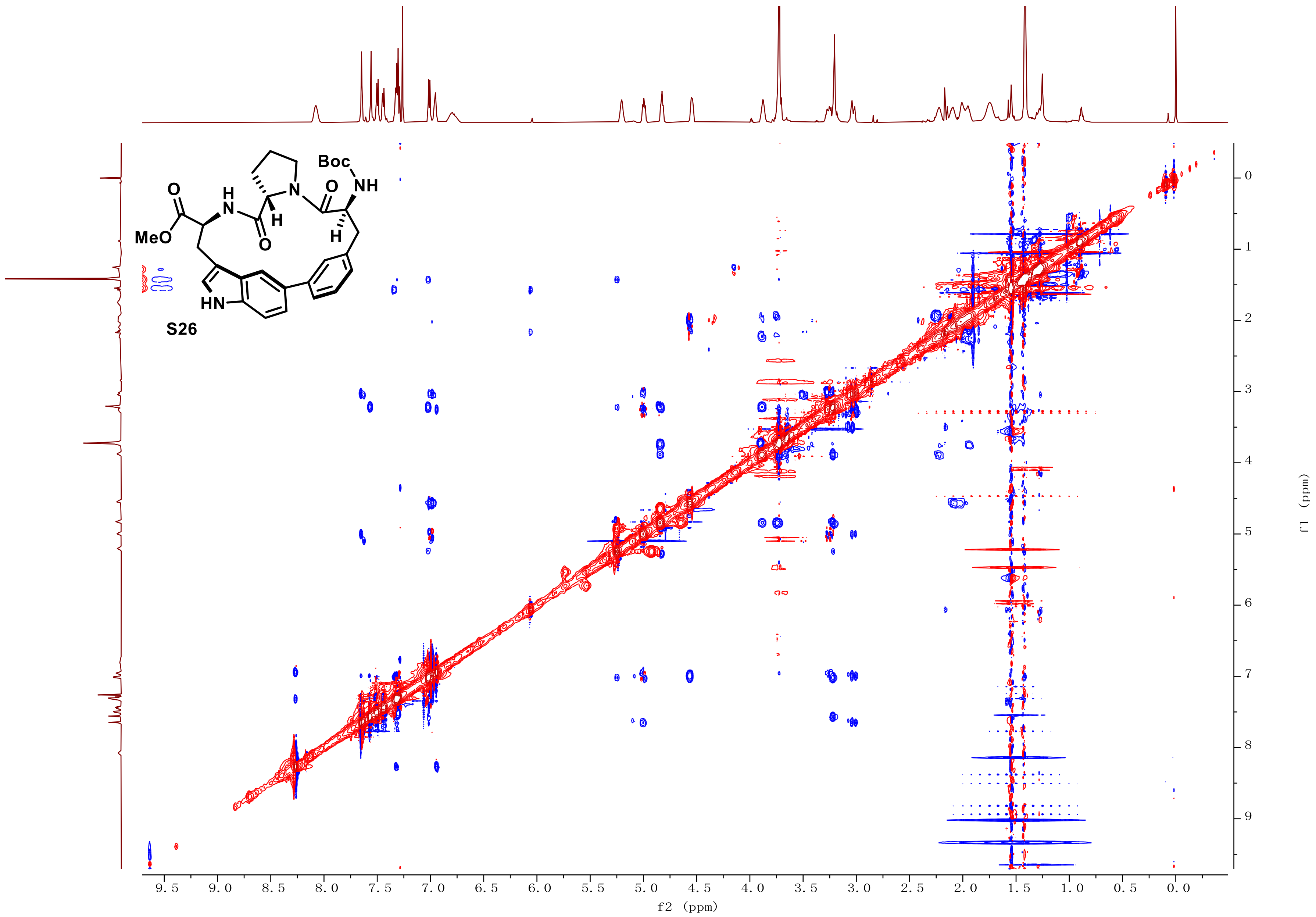

Compound 73 ^{1}H NMR (400 MHz, CD_3OD)

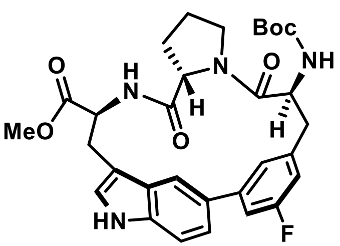

73

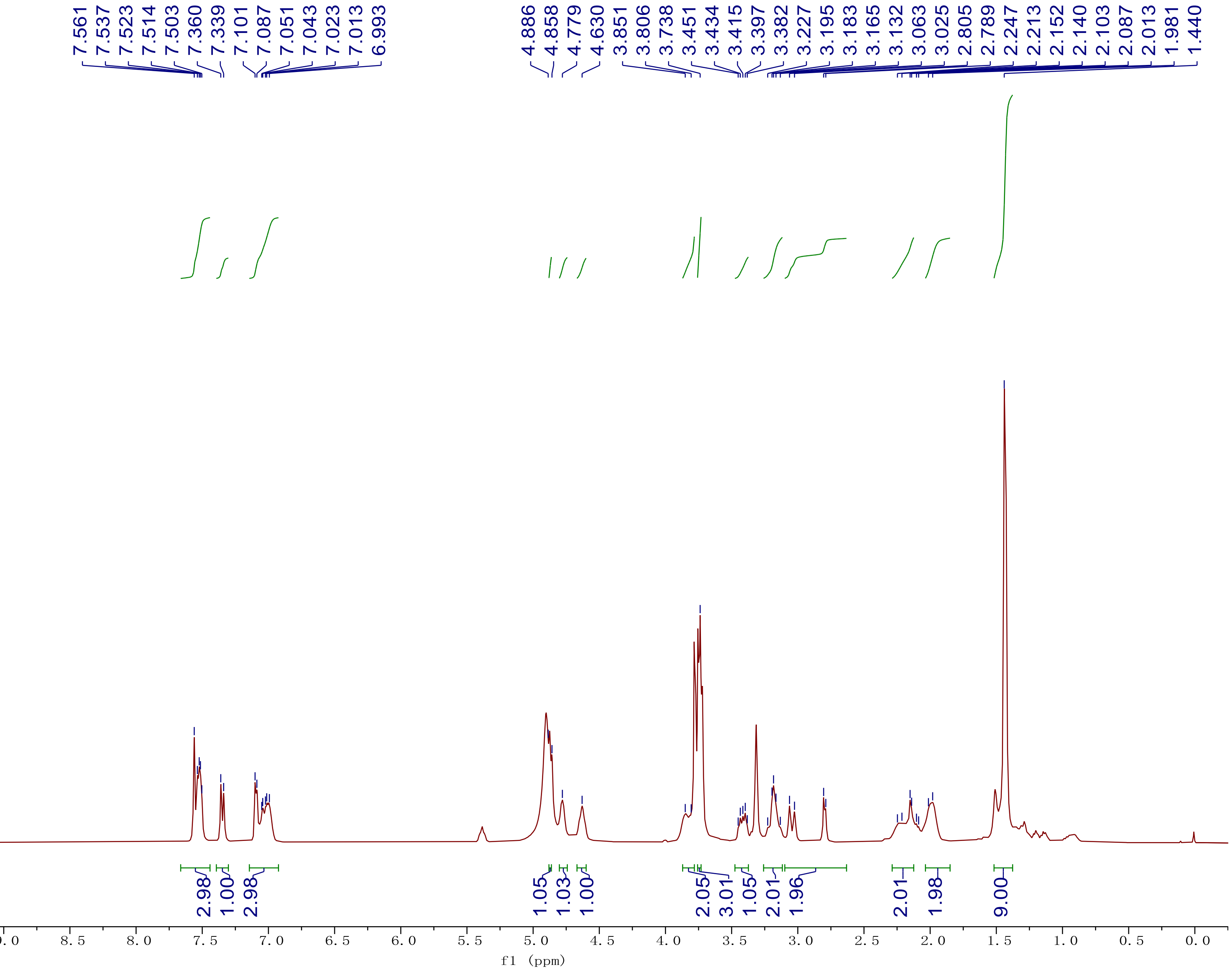

Compound 73 ^{13}C NMR (101 MHz, CD_3OD)

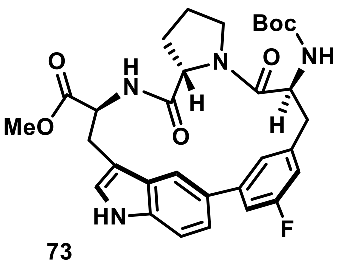

73

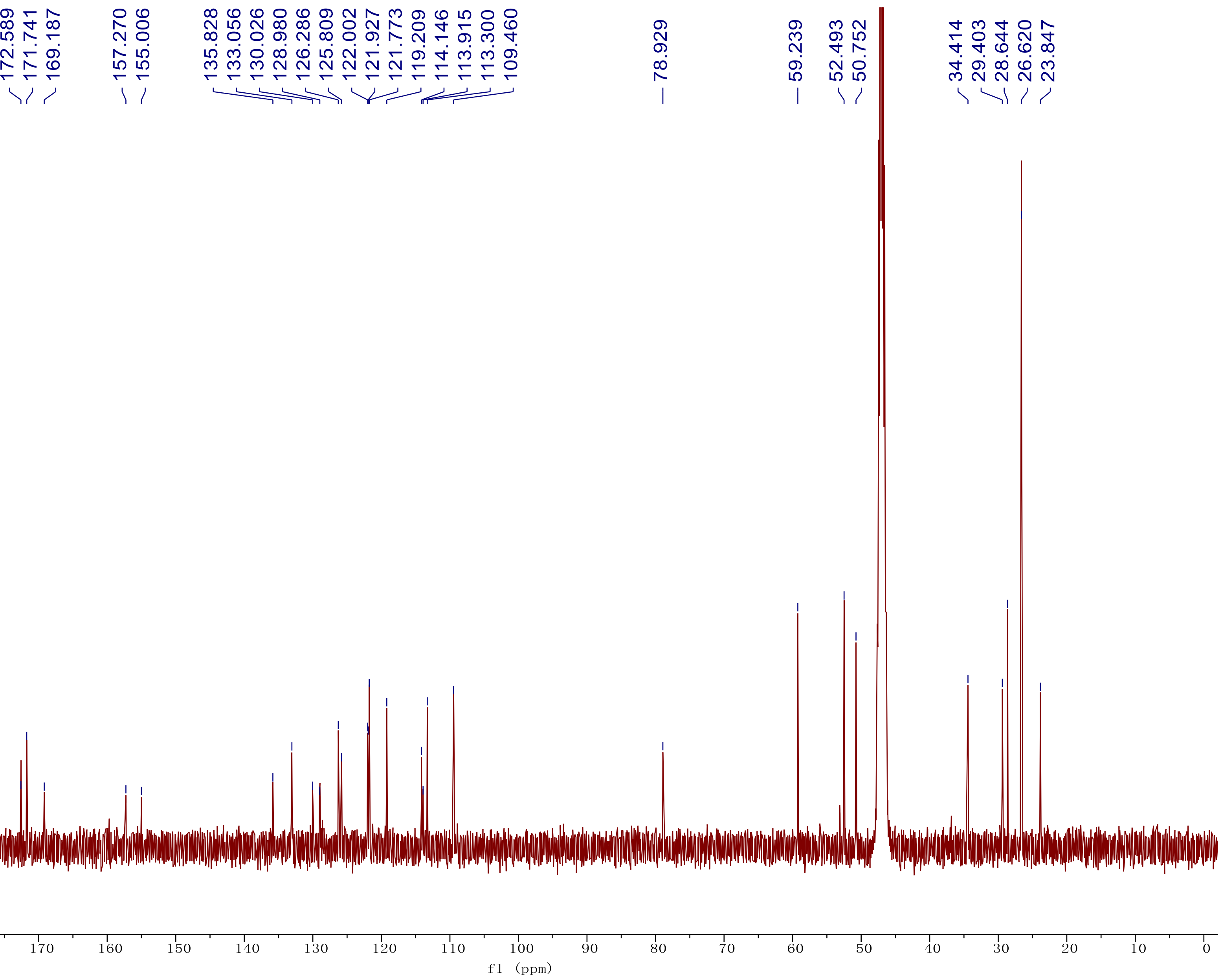

Compound 73 ^{19}F NMR (377 MHz, CD_3OD)

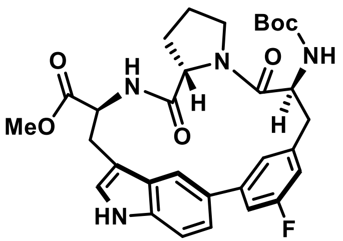

73

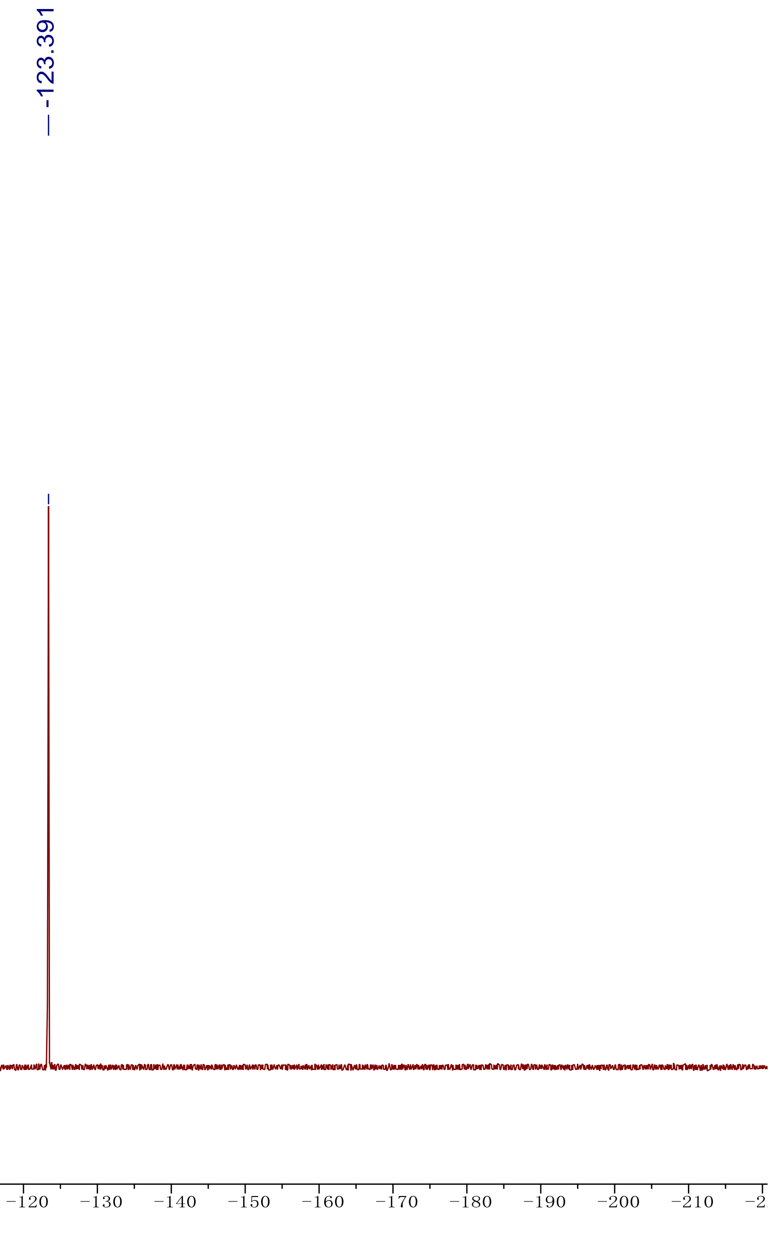

Compound 74 ^{1}H NMR (600 MHz, $CDCl_3$)

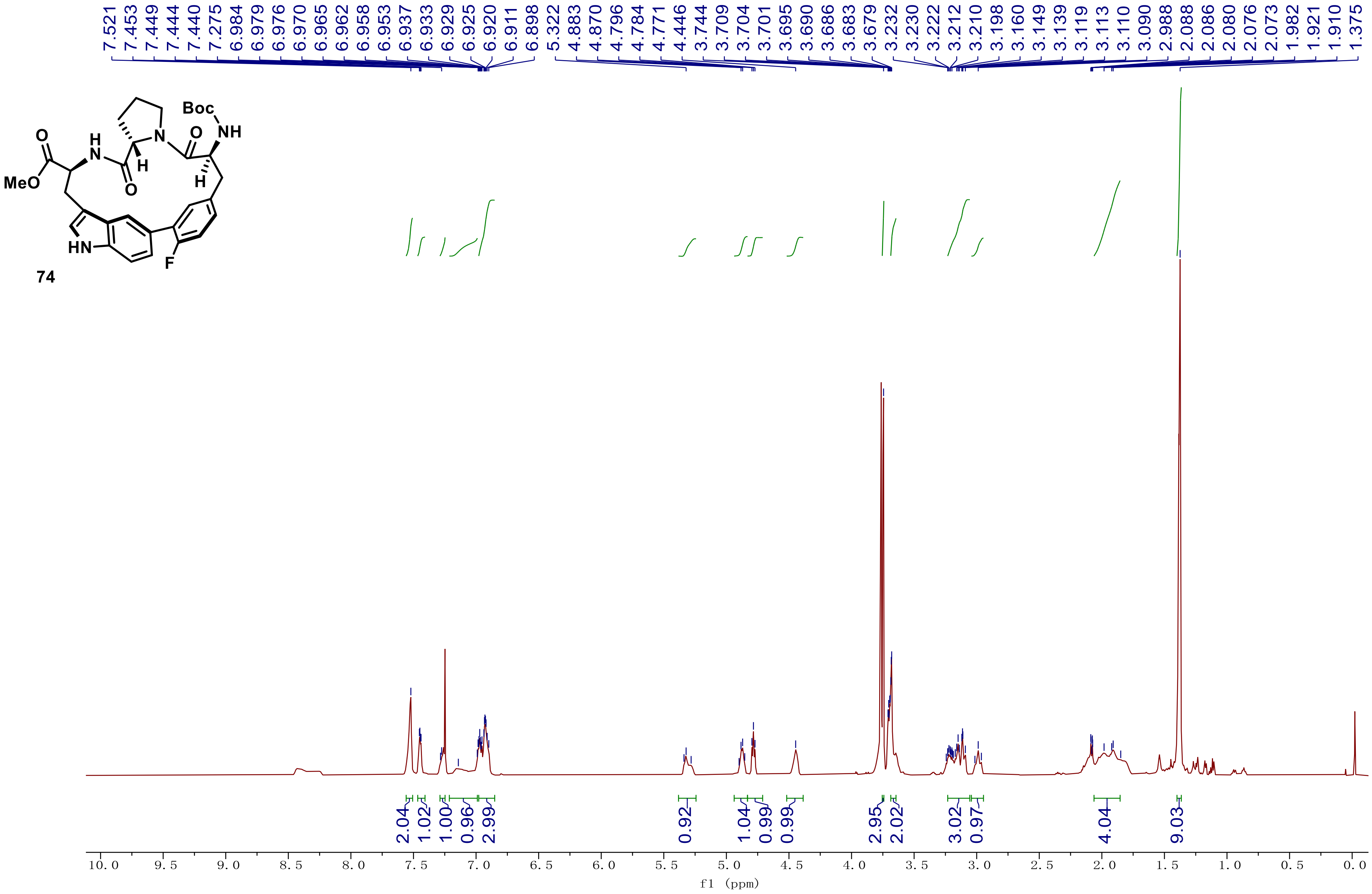

Compound 74 ^{13}C NMR (151 MHz, $CDCl_3$)

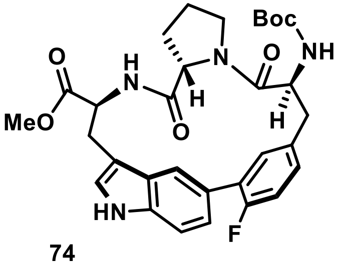

74

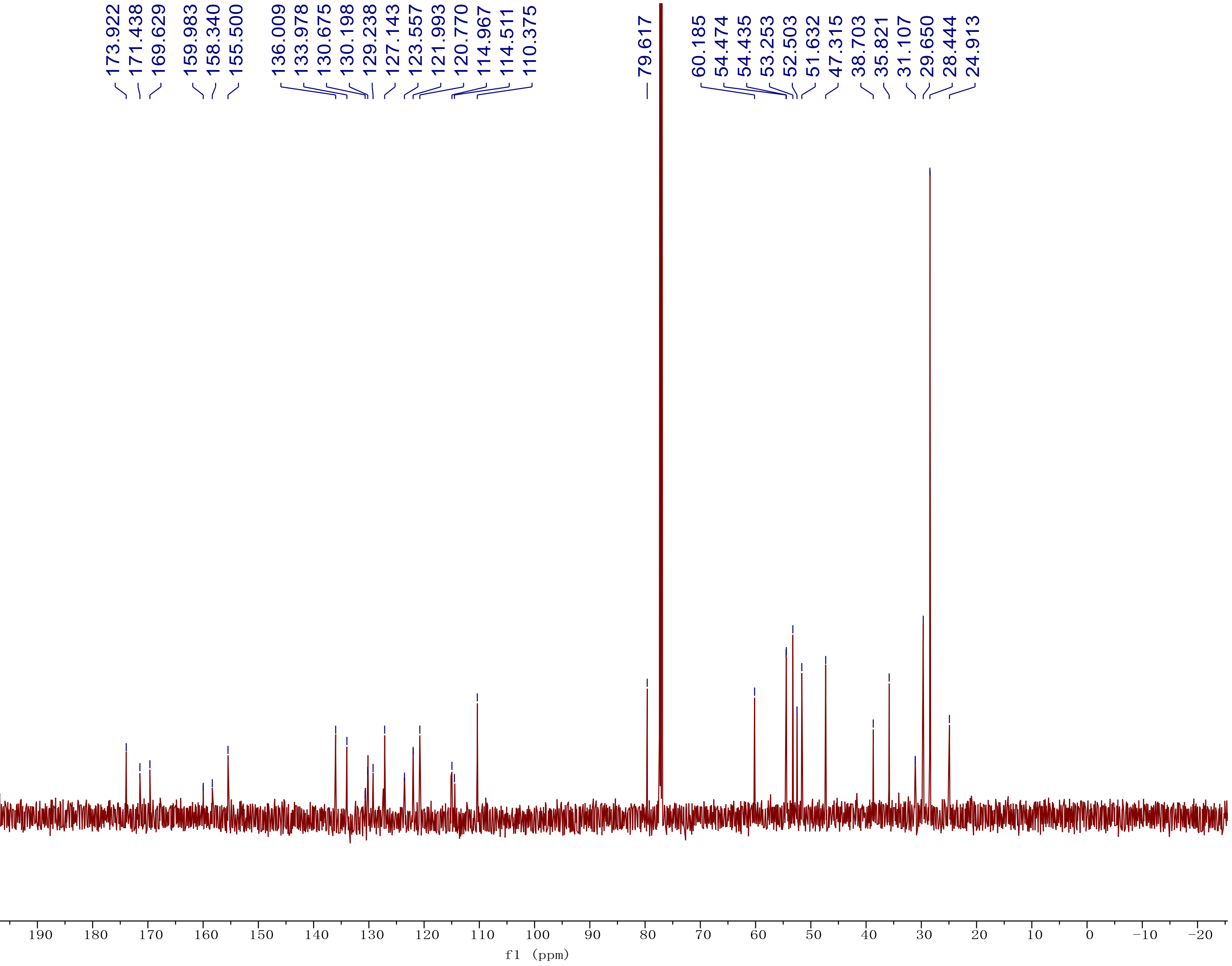

Compound 74 ^{19}F NMR (575 MHz, CDCl$_3$)

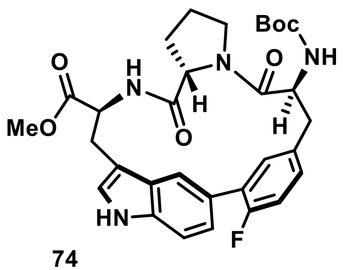

74

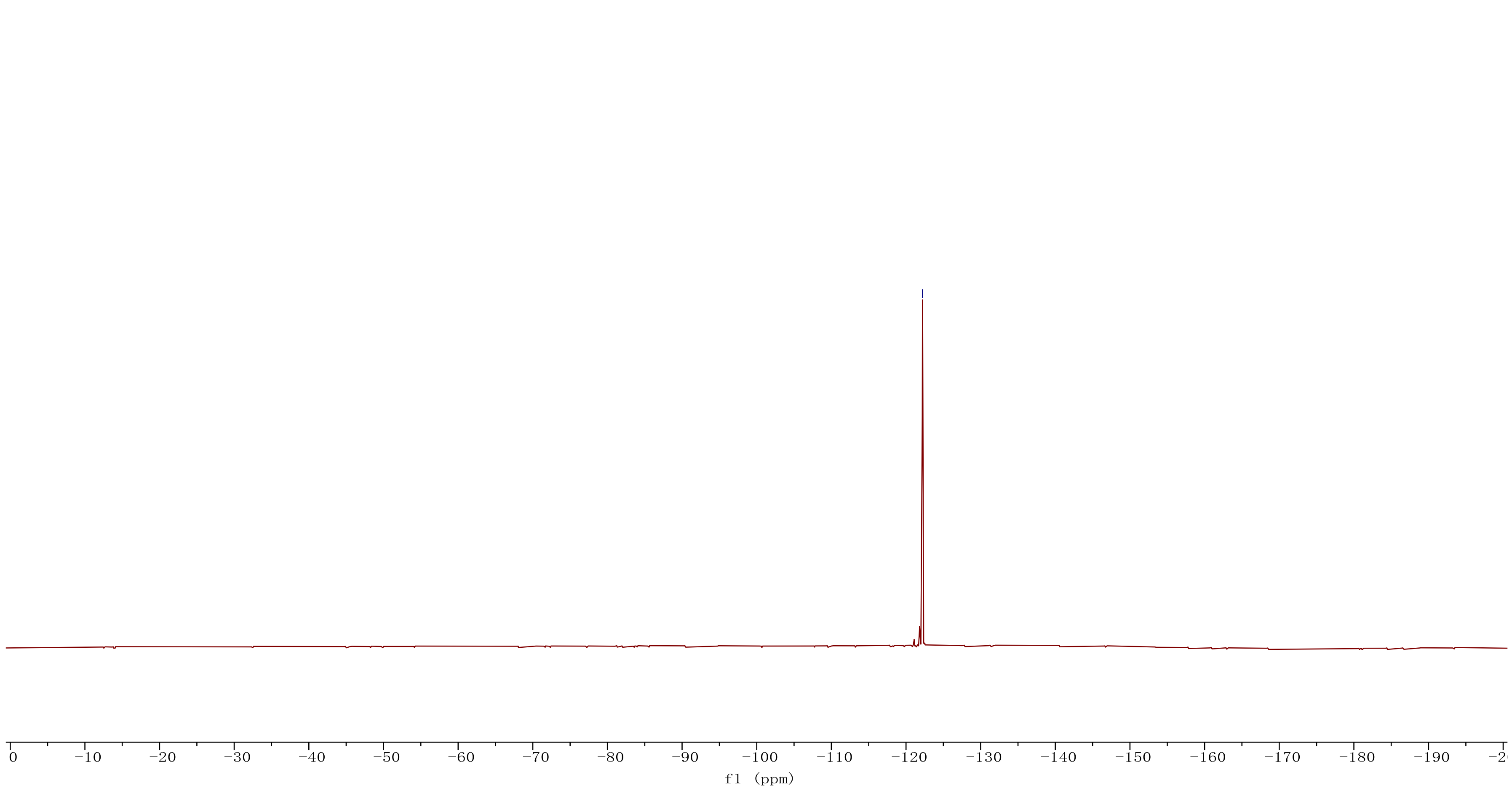

Compound 78 ^{1}H NMR (400 MHz, CDCl$_3$)

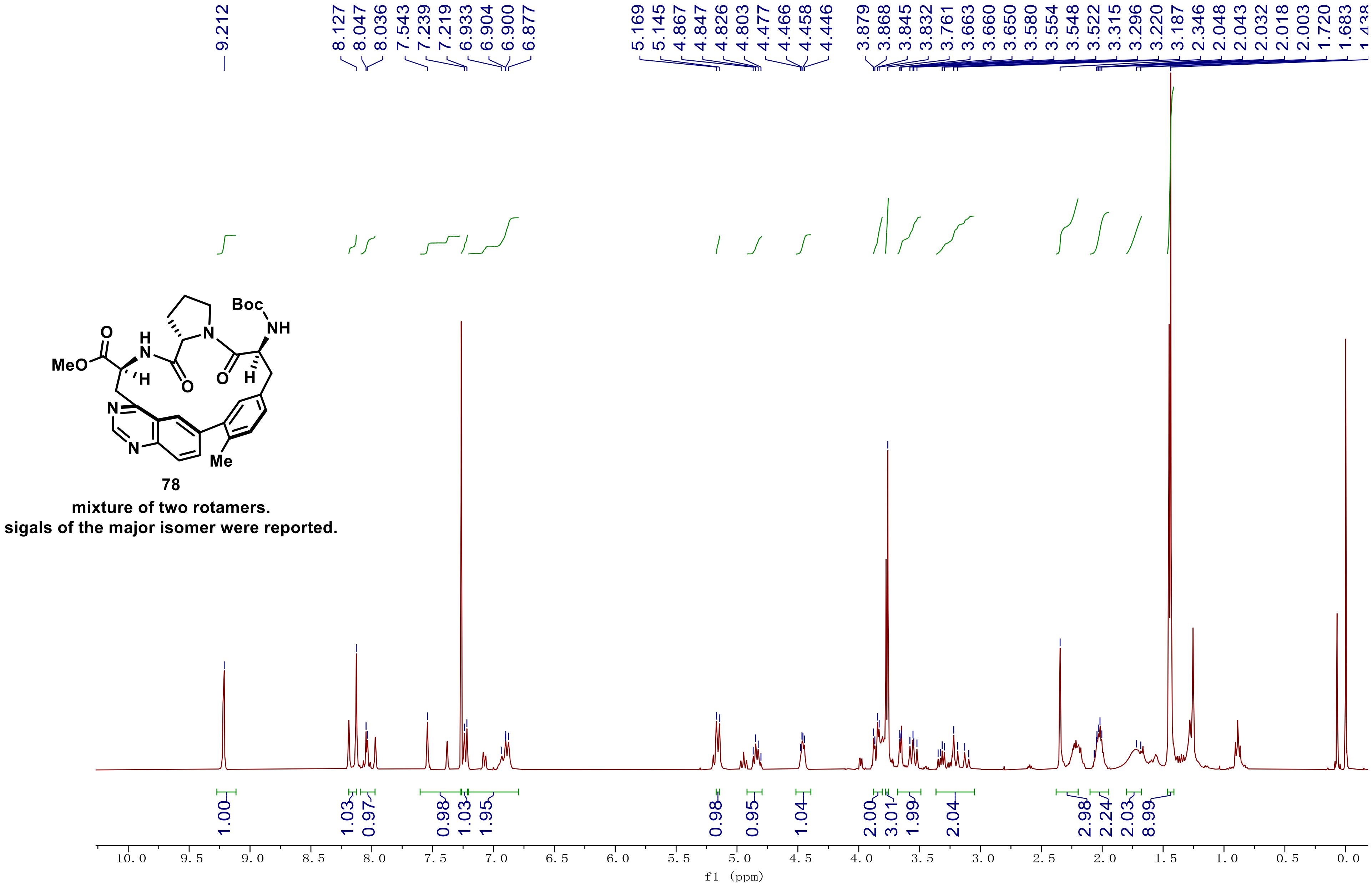

78

mixture of two rotamers.

sigals of the major isomer were reported.

Compound 78 ^{13}C NMR (151 MHz, CDCl$_3$)

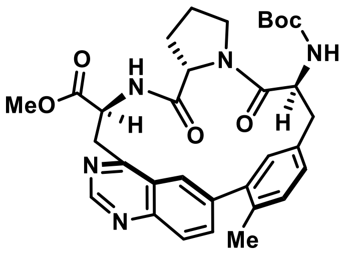

78
mixture of two rotamers.
sigals of the major isomer were reported.

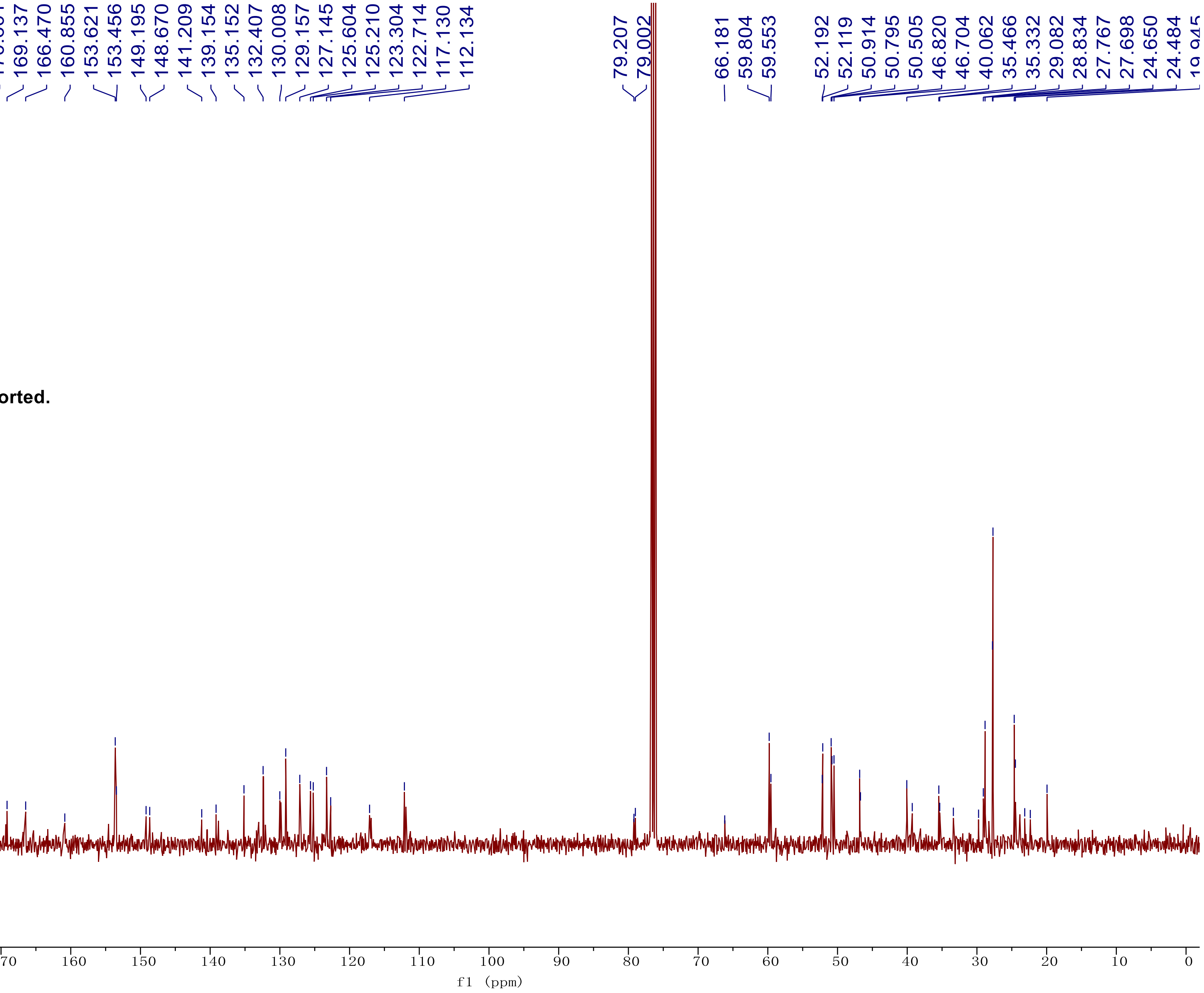

Compound 79 ^{1}H NMR (400 MHz, CD_3OD)

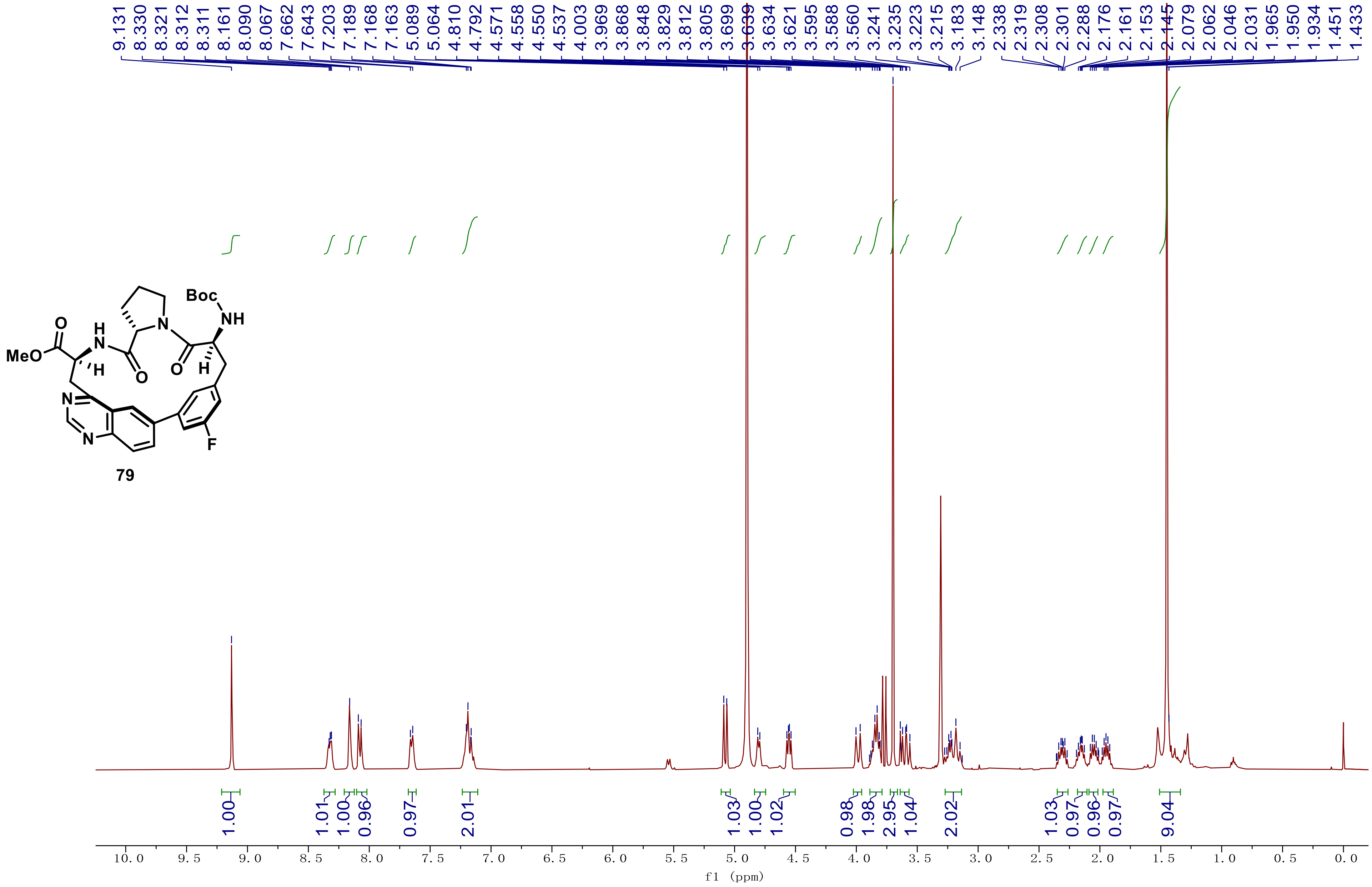

Compound 79 ^{13}C NMR (101 MHz, CD_3OD)

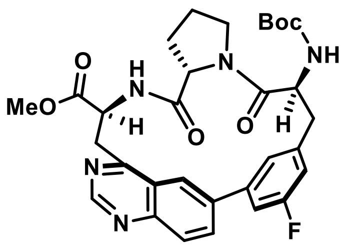
79

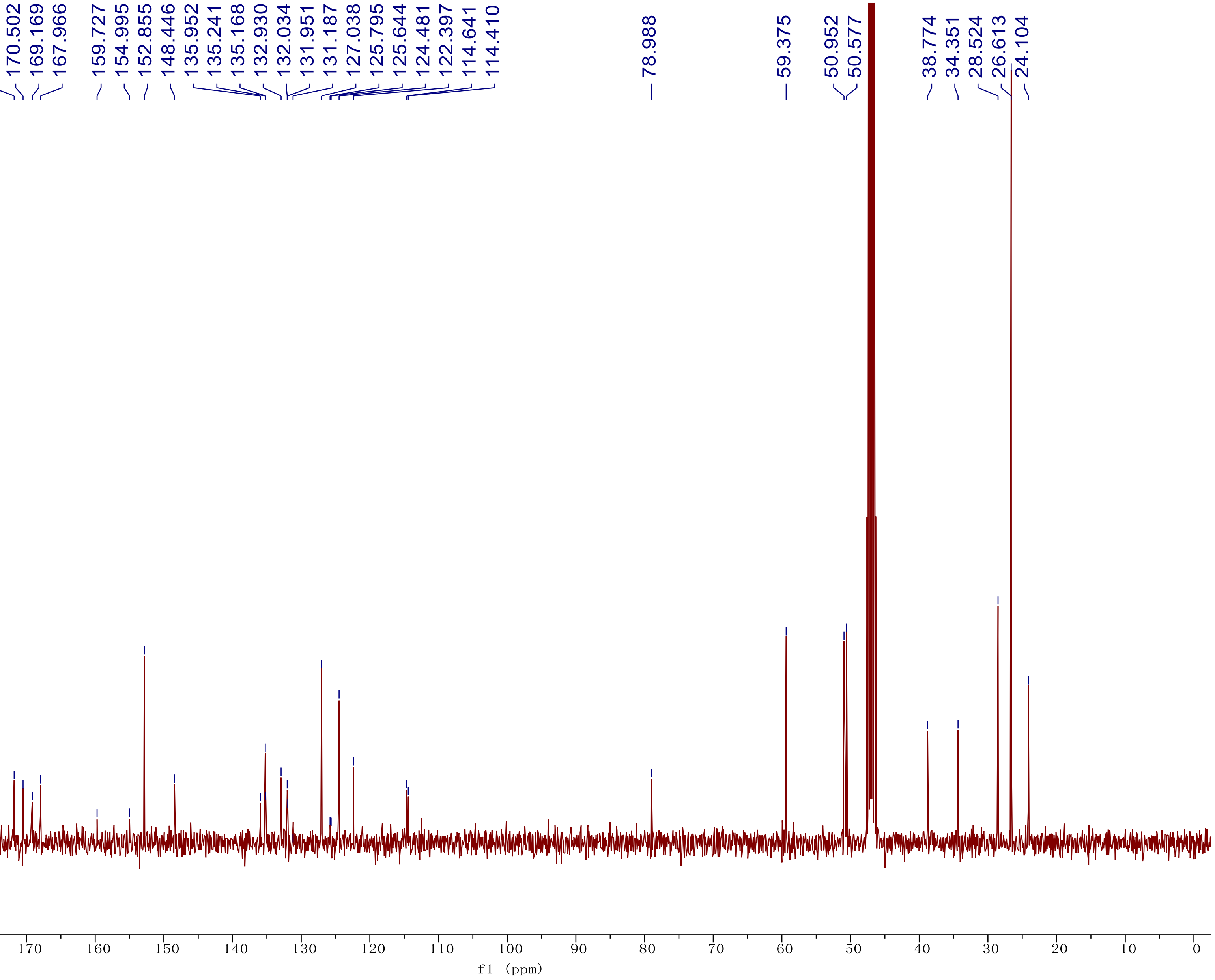

Compound 79 ^{19}F NMR (565 MHz, CD_3OD)

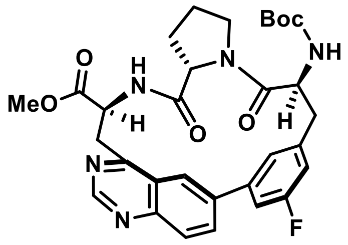

79

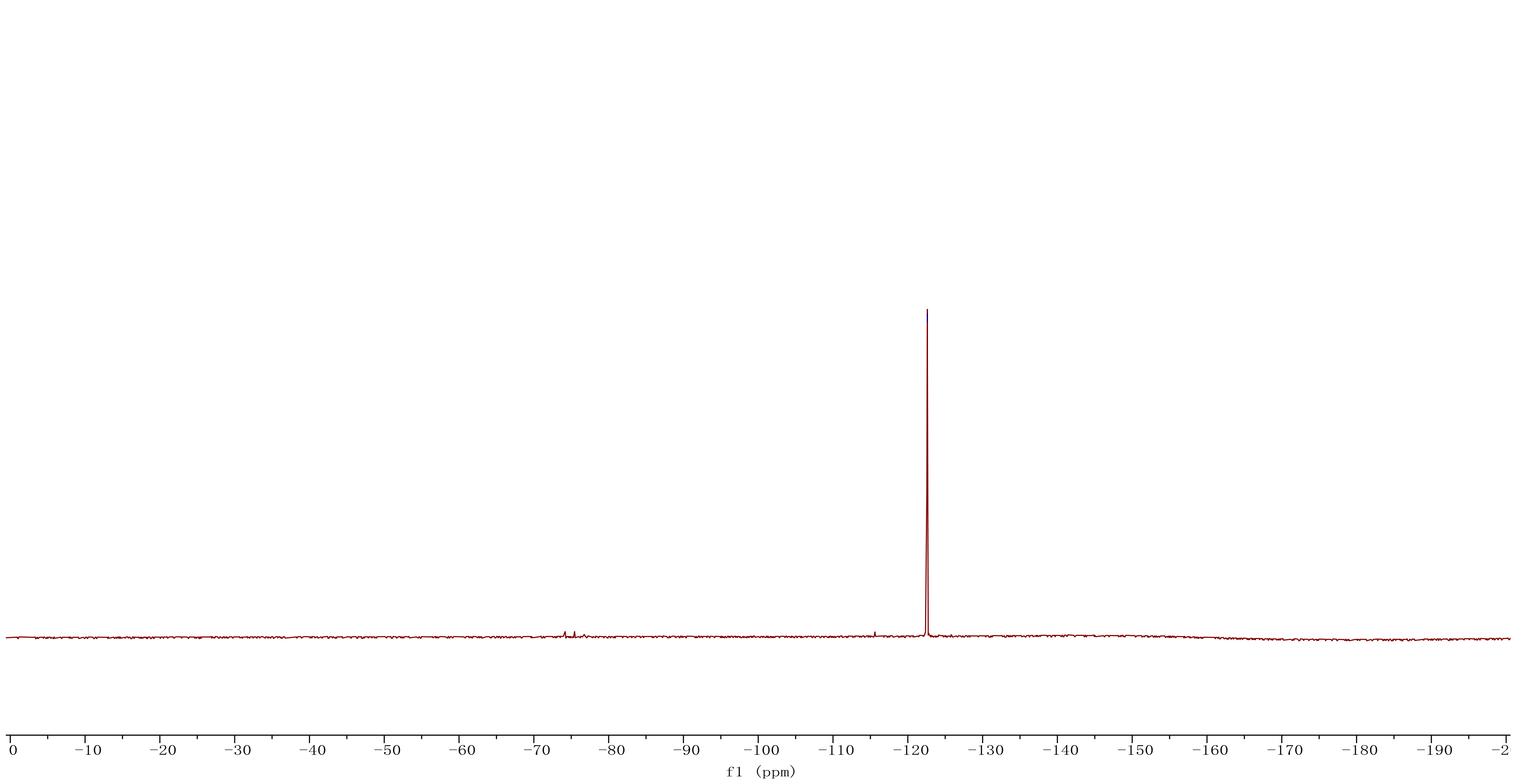

Compound 80 ¹H NMR (400 MHz, $CDCl_3$)

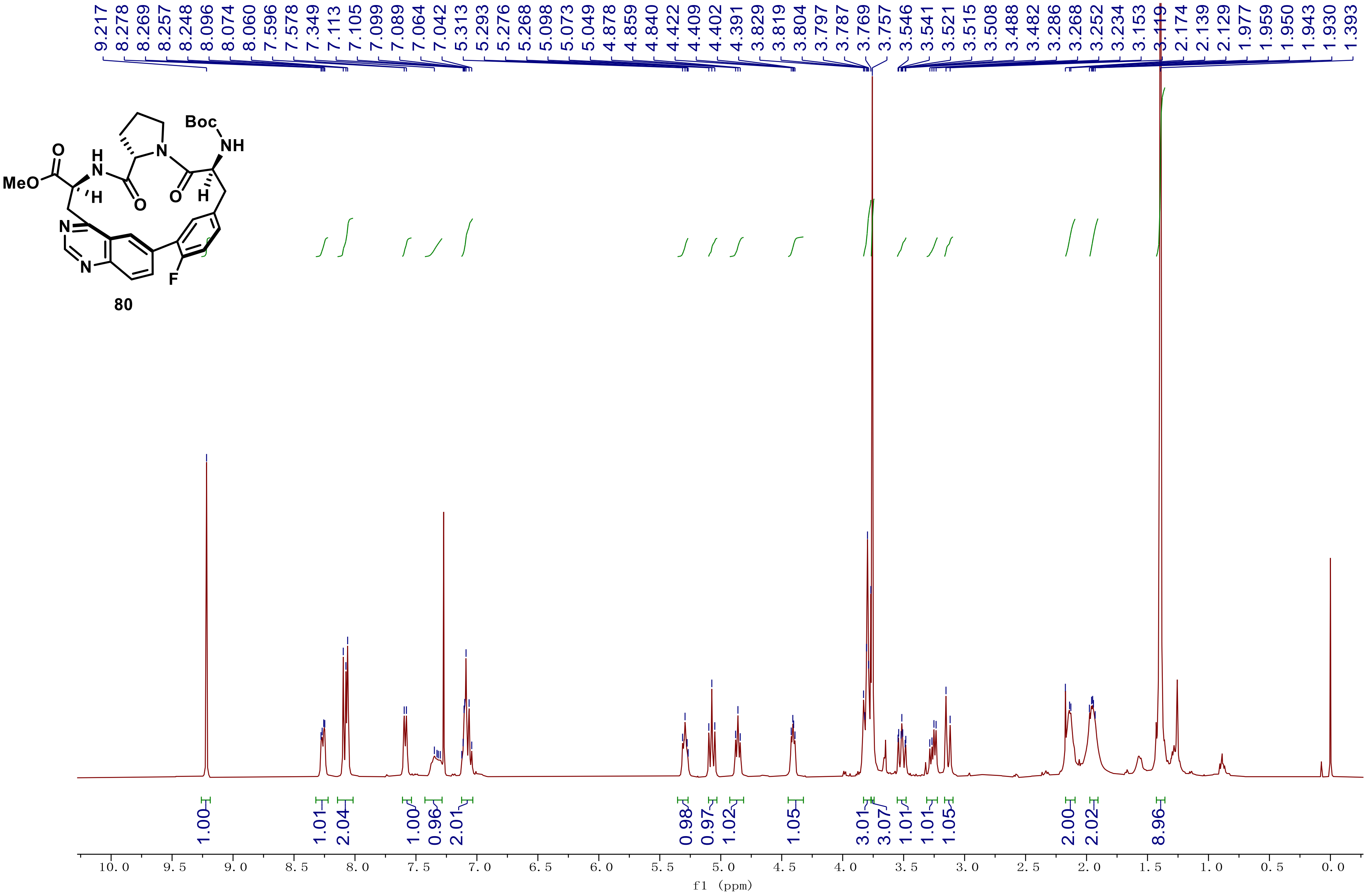

Compound 80 ^{13}C NMR (151 MHz, $CDCl_3$)

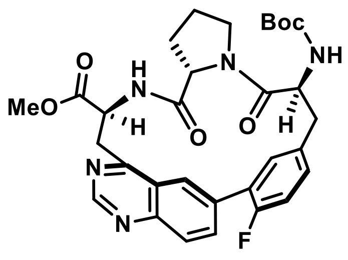

80

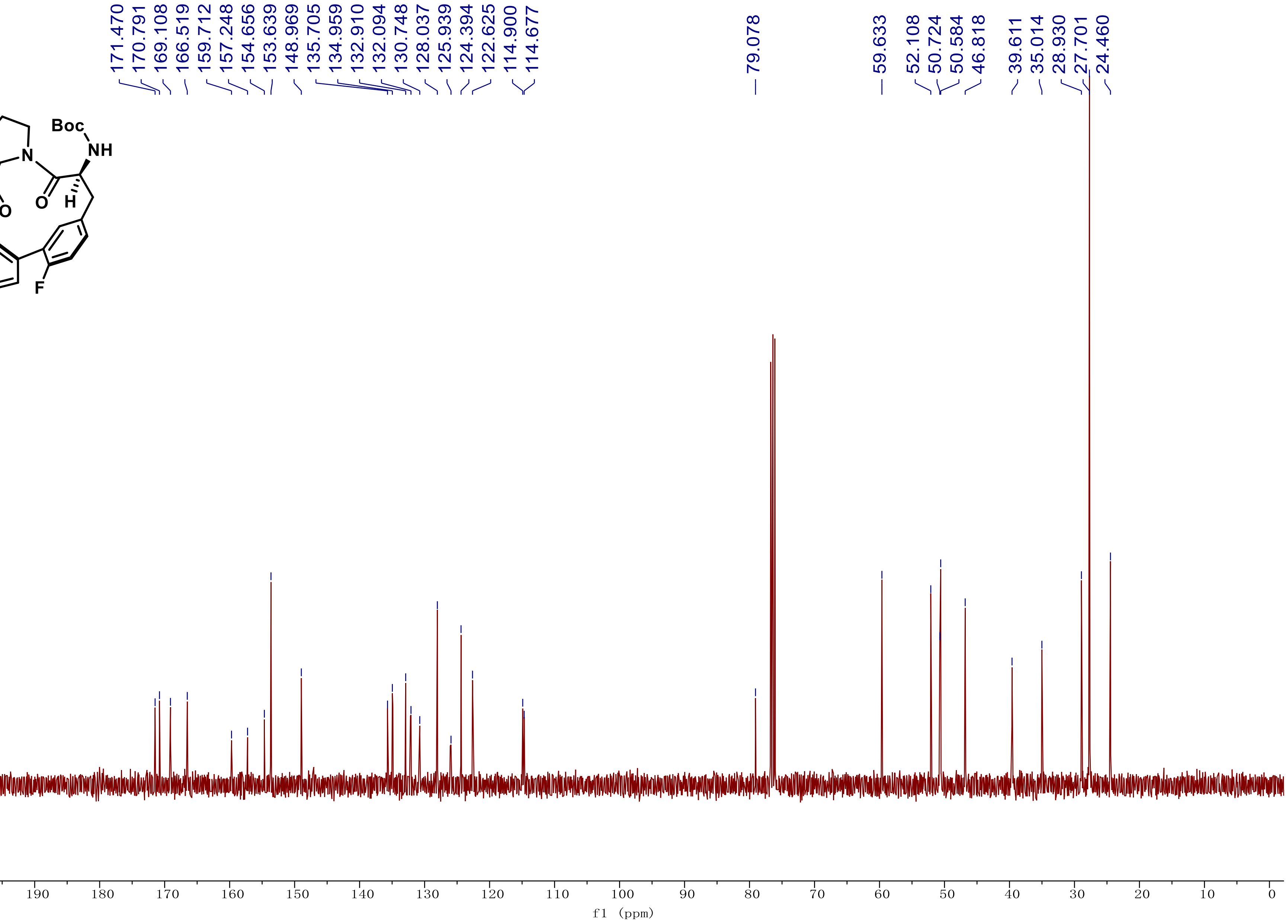

Compound 80 ^{19}F NMR (377 MHz, $CDCl_3$)

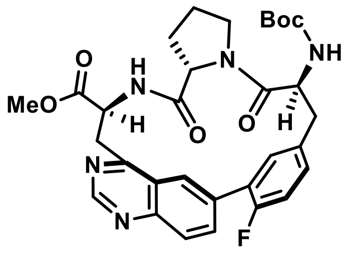

80

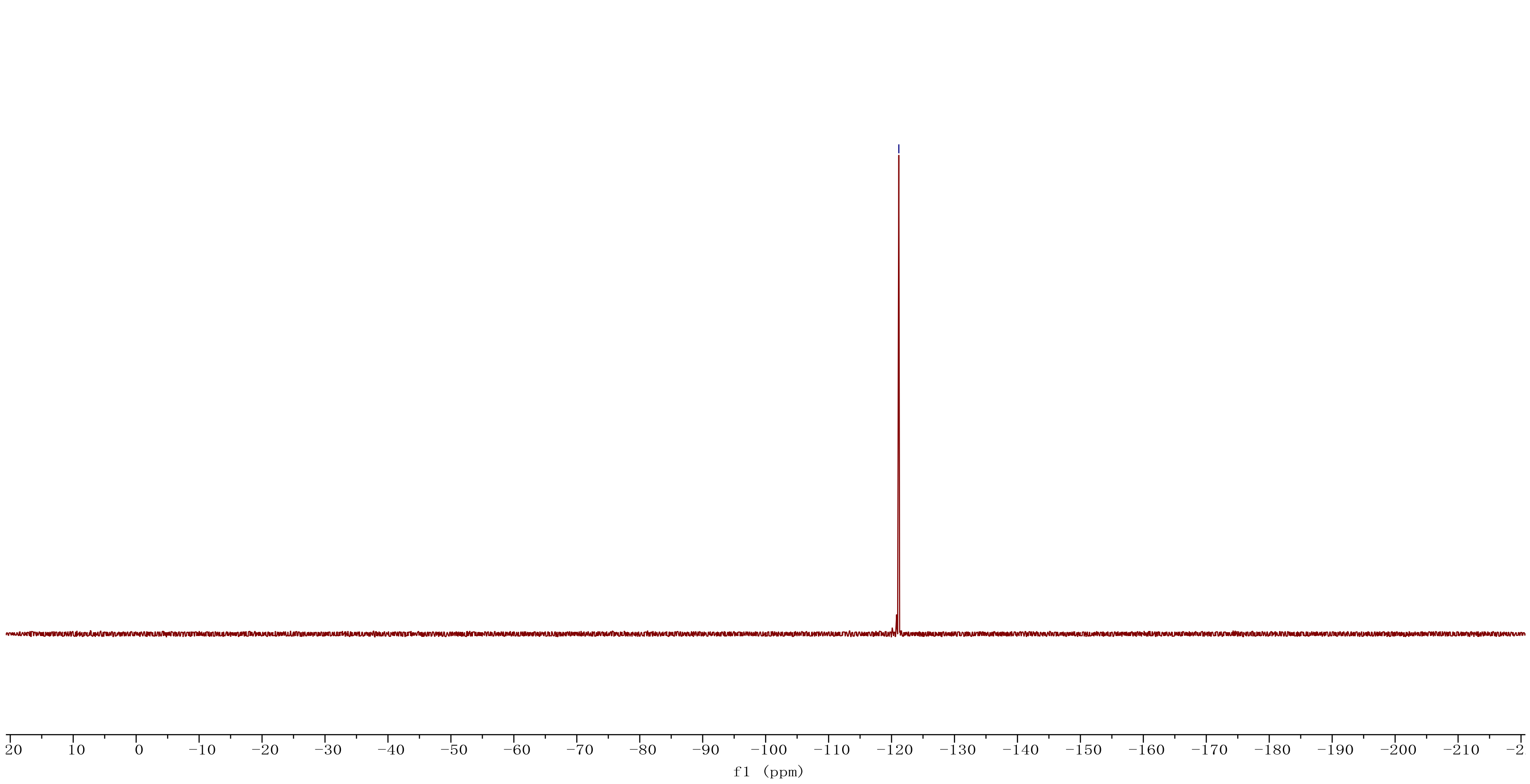

Compound 75 ^{1}H NMR (400 MHz, CDCl$_3$)

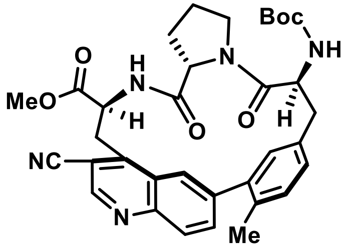

75

A mixture of rotamers (**A**:**B** = 2.2:1)
SIngals of the major rotamers were reported.

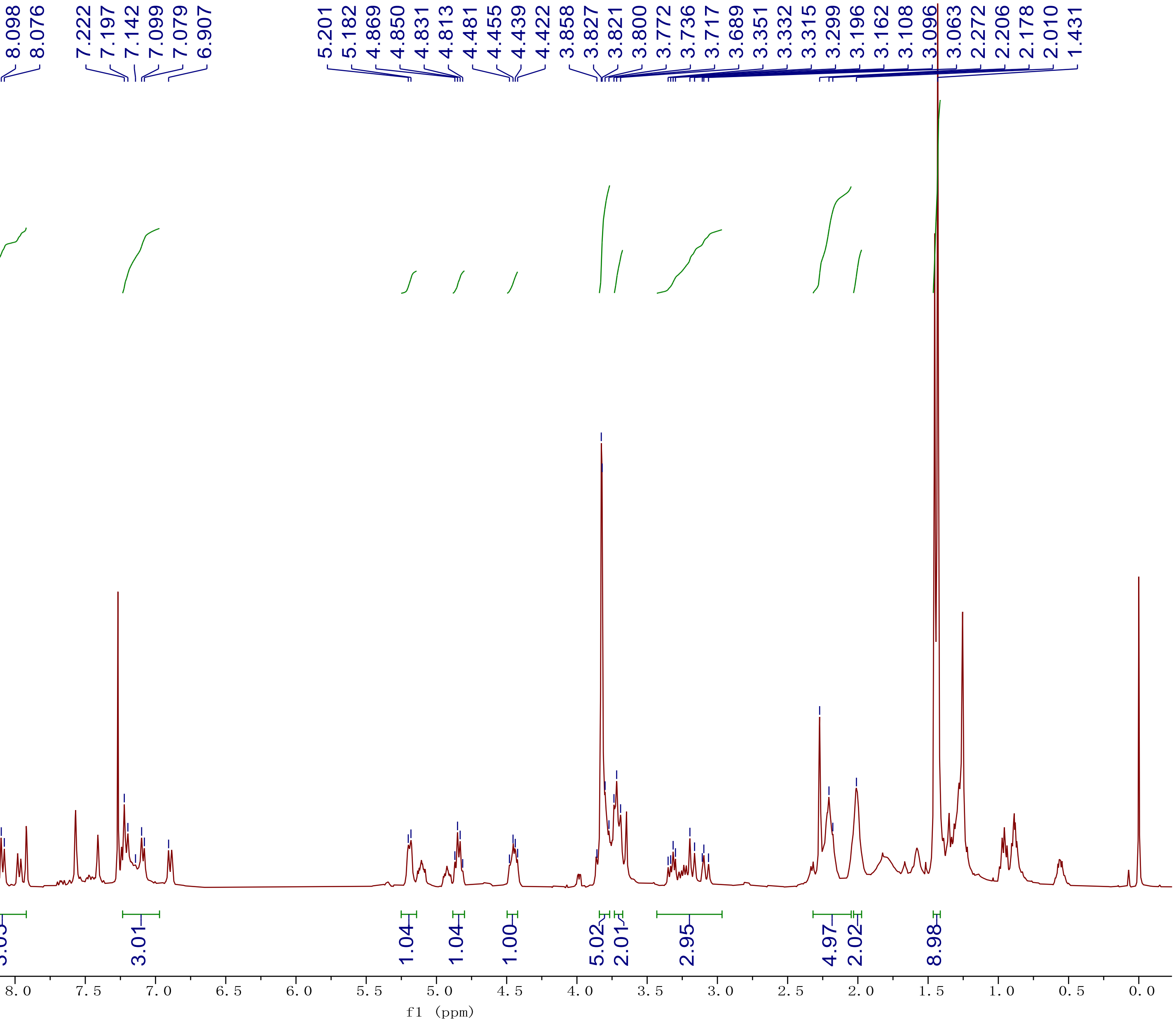

Compound 75 ^{13}C NMR (101 MHz, $CDCl_3$)

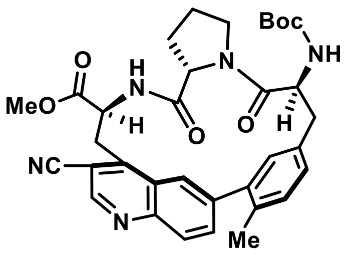

75

A mixture of rotamers (**A**:**B** = 2.2:1)
SIngals of the major rotamers were reported.

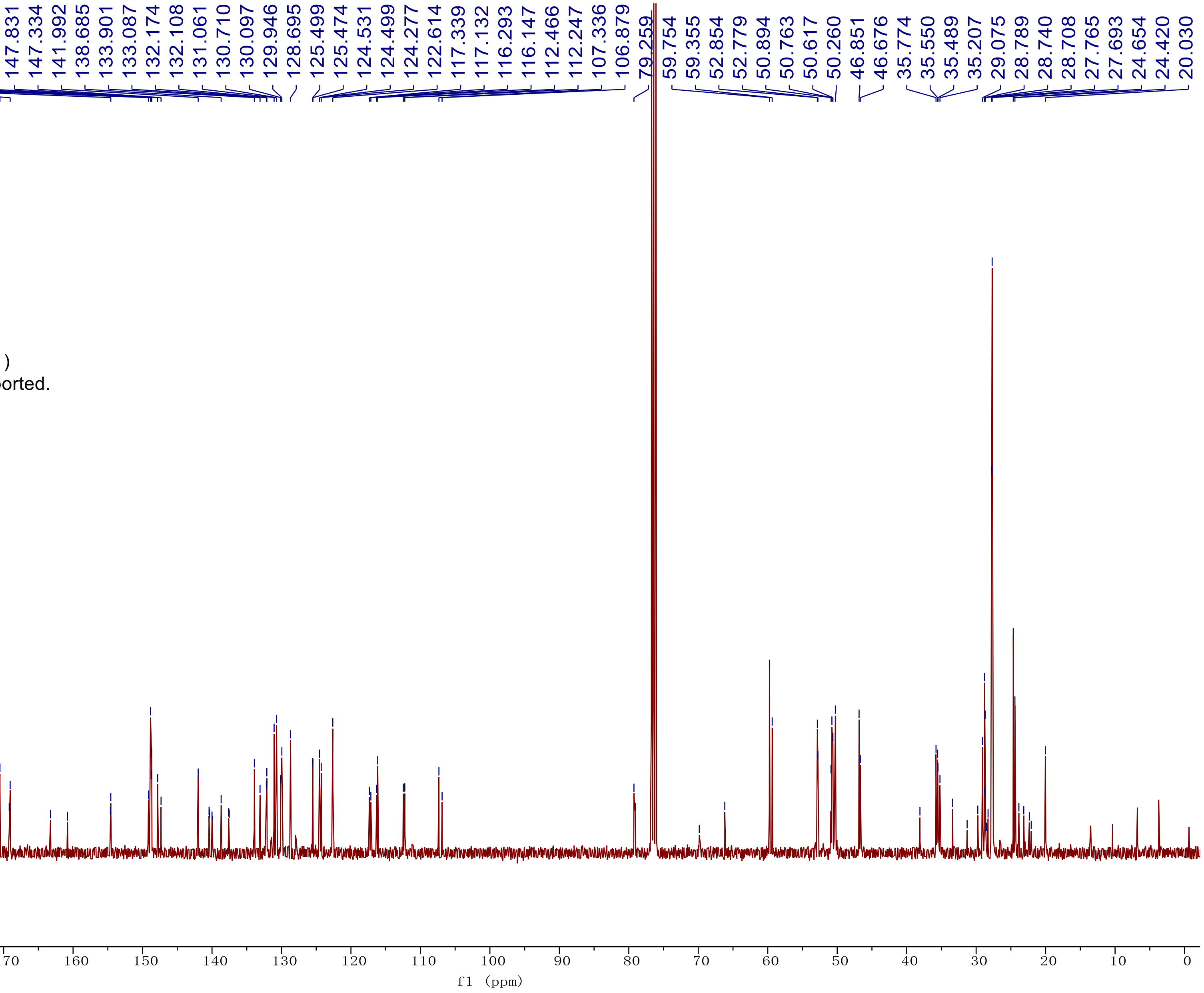

Compound 76 ^{1}H NMR (400 MHz, $CDCl_3$)

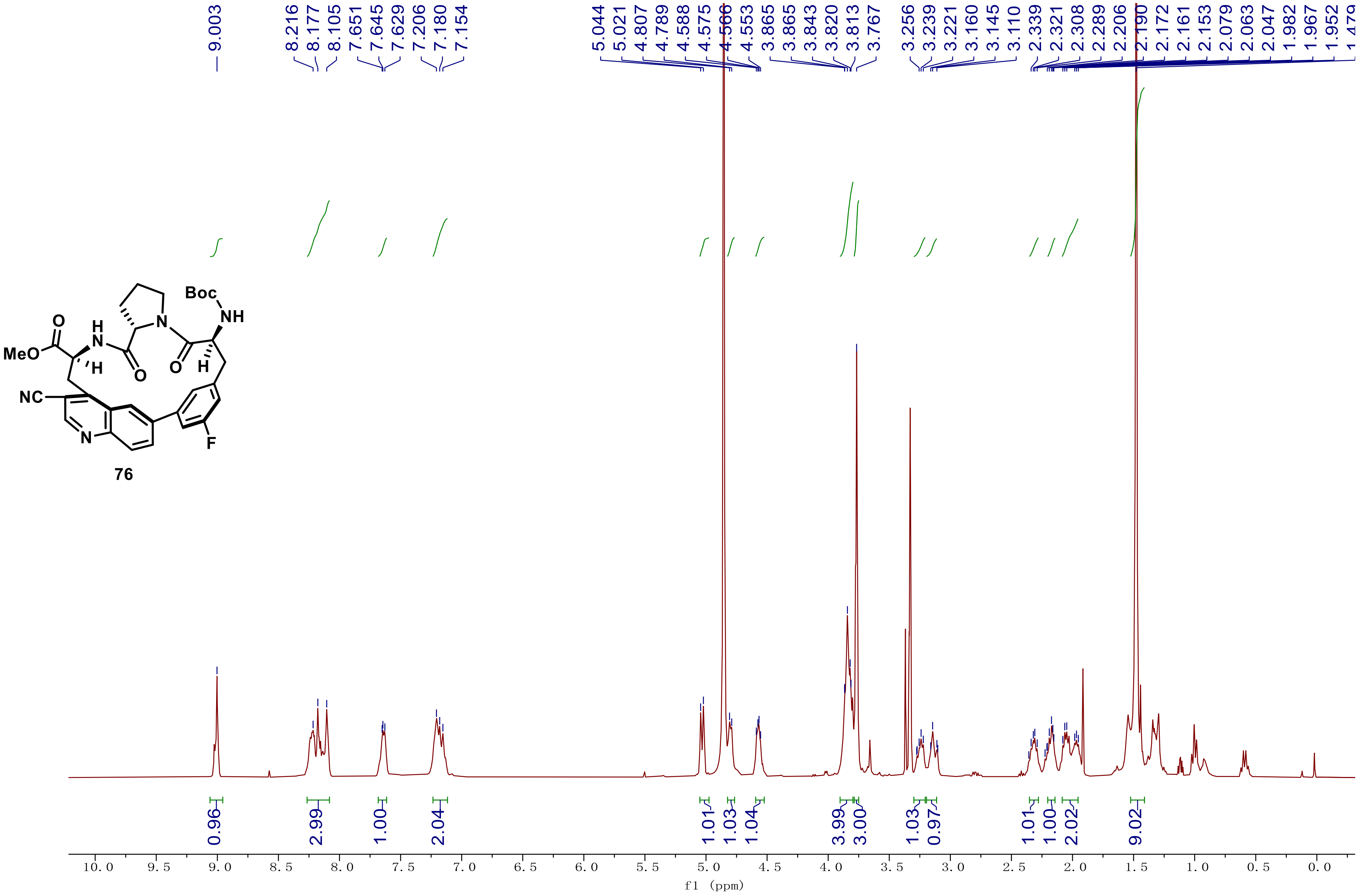

Compound 76 ^{13}C NMR (101 MHz, CD_3OD)

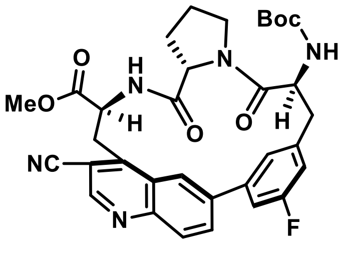

76

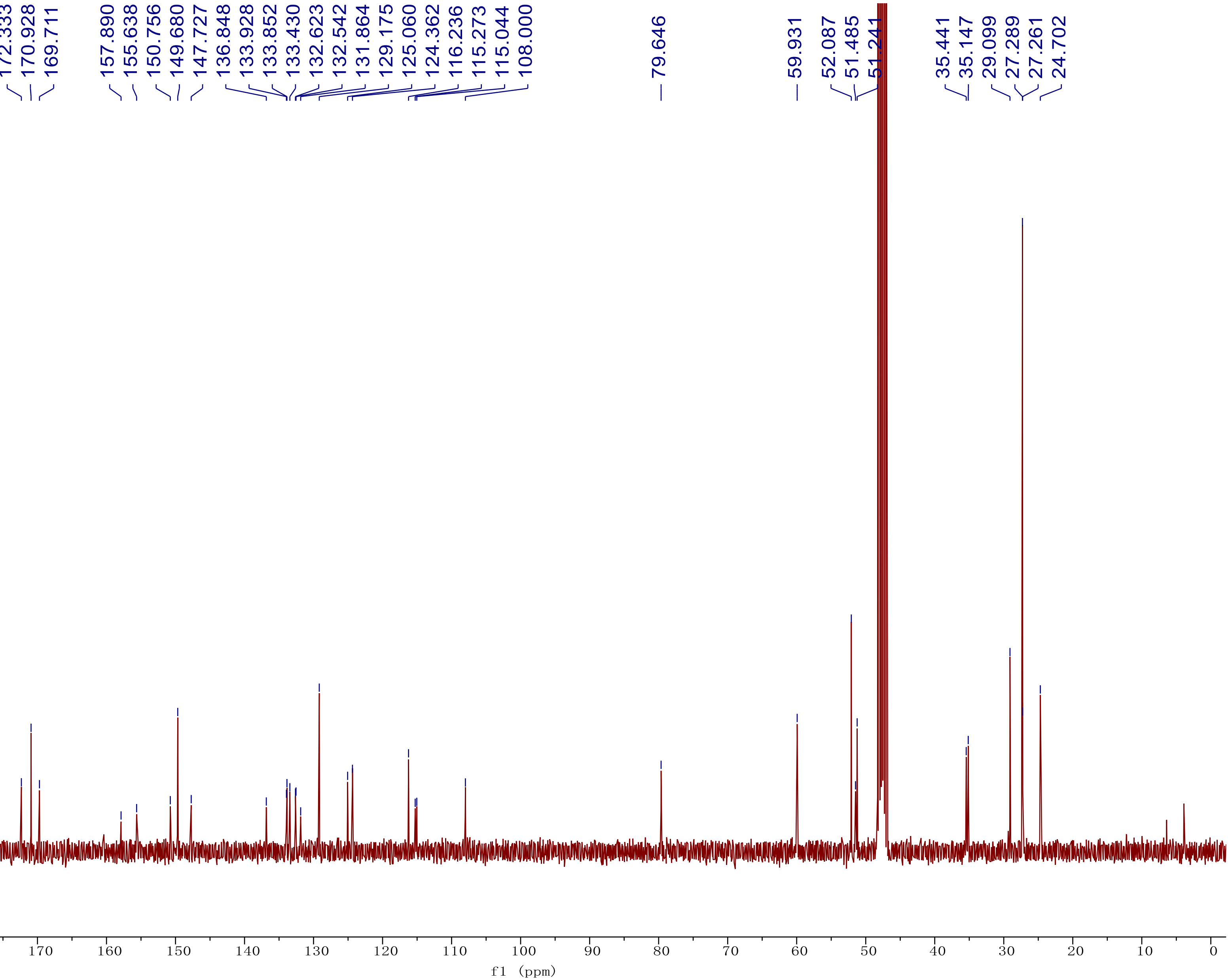

Compound 76 ^{19}F NMR (575 MHz, CD_3OD)

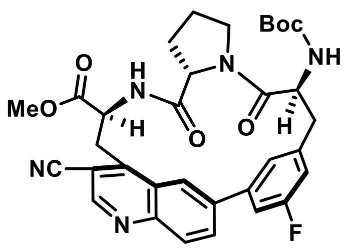

76

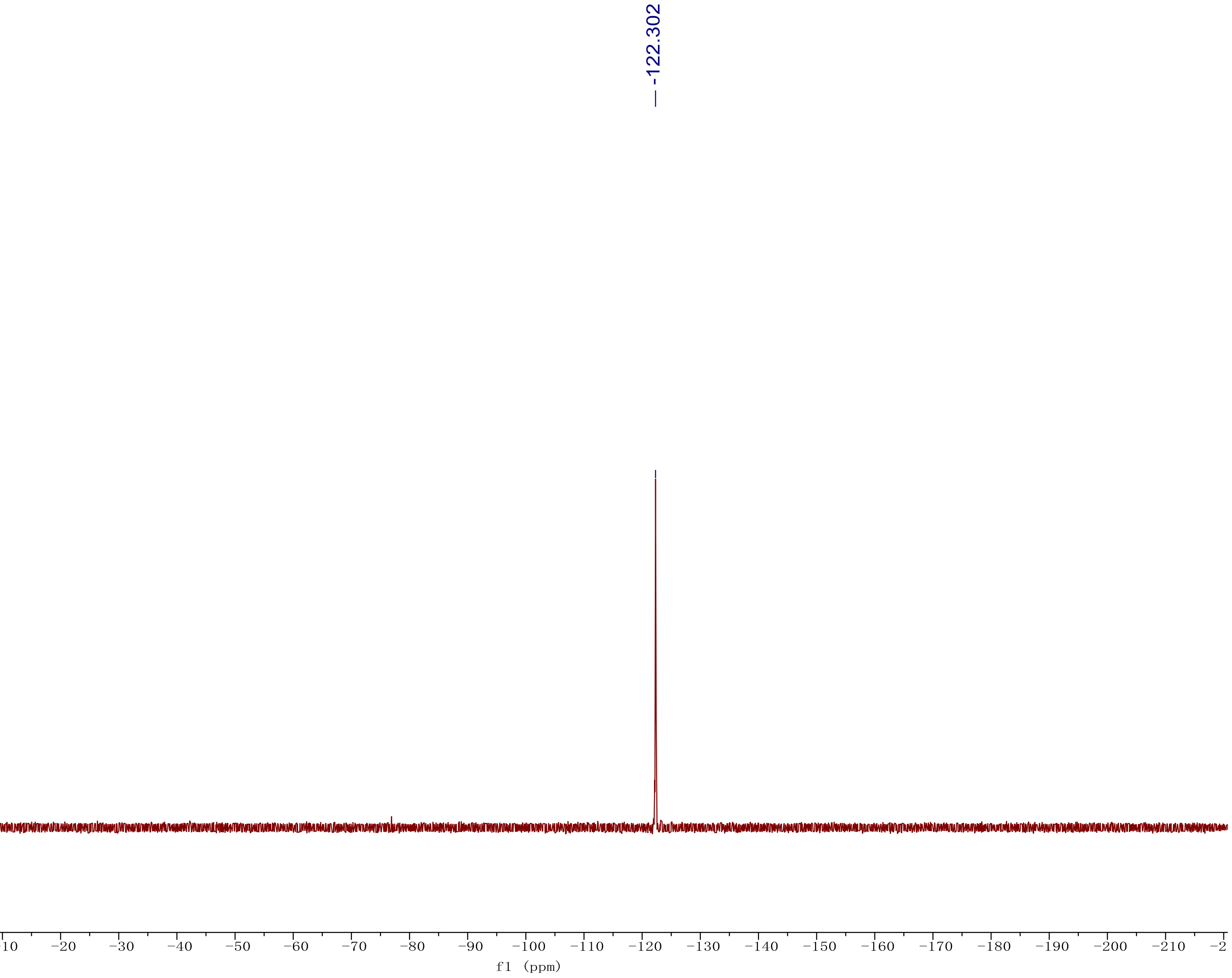

Compound 77 ^{1}H NMR (400 MHz, $CDCl_3$)

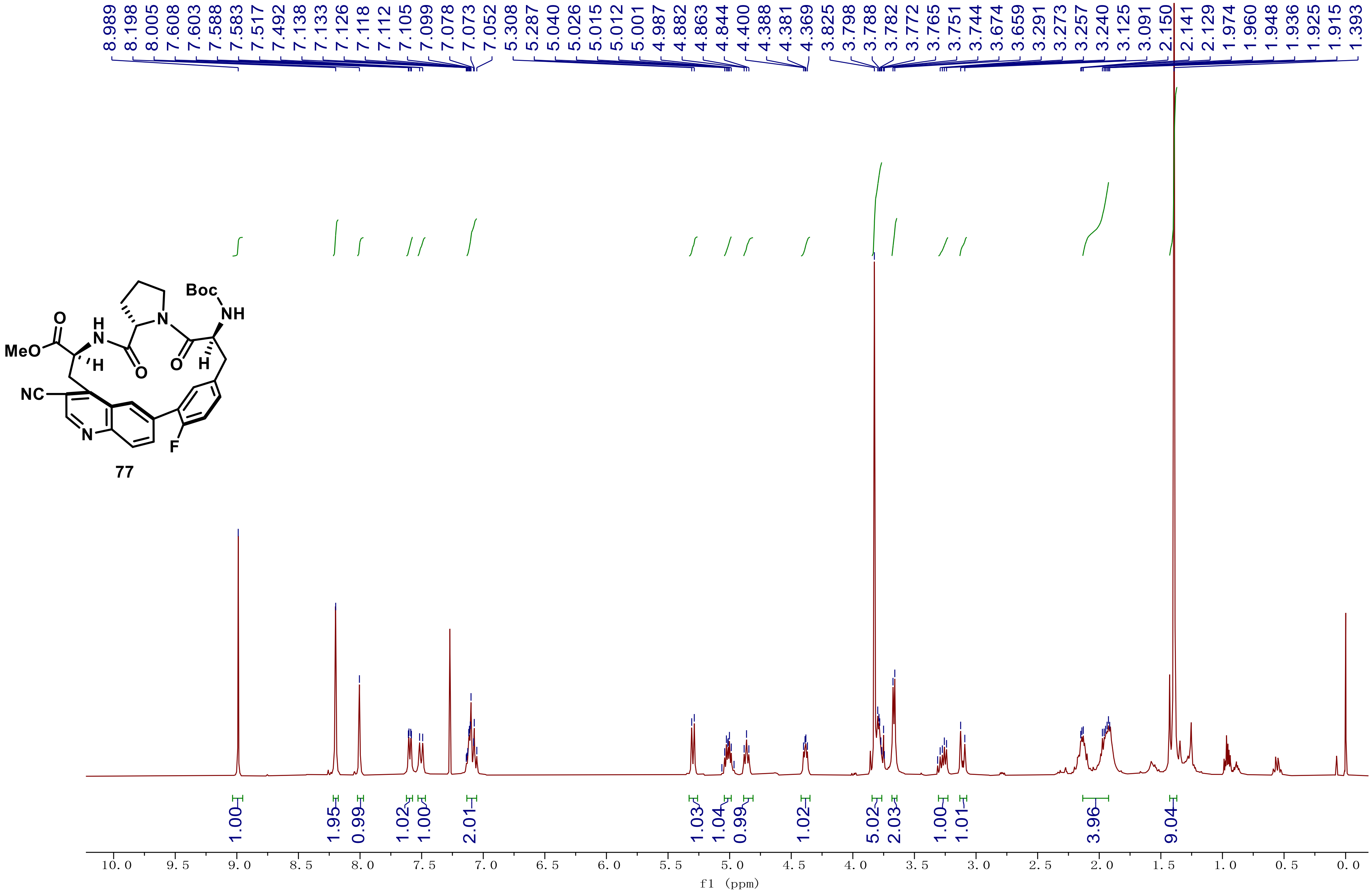

77

Compound 77 ^{13}C NMR (101 MHz, $CDCl_3$)

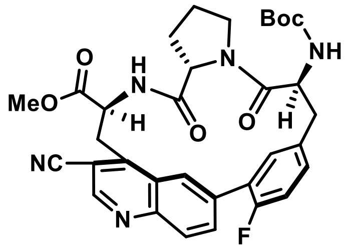

77

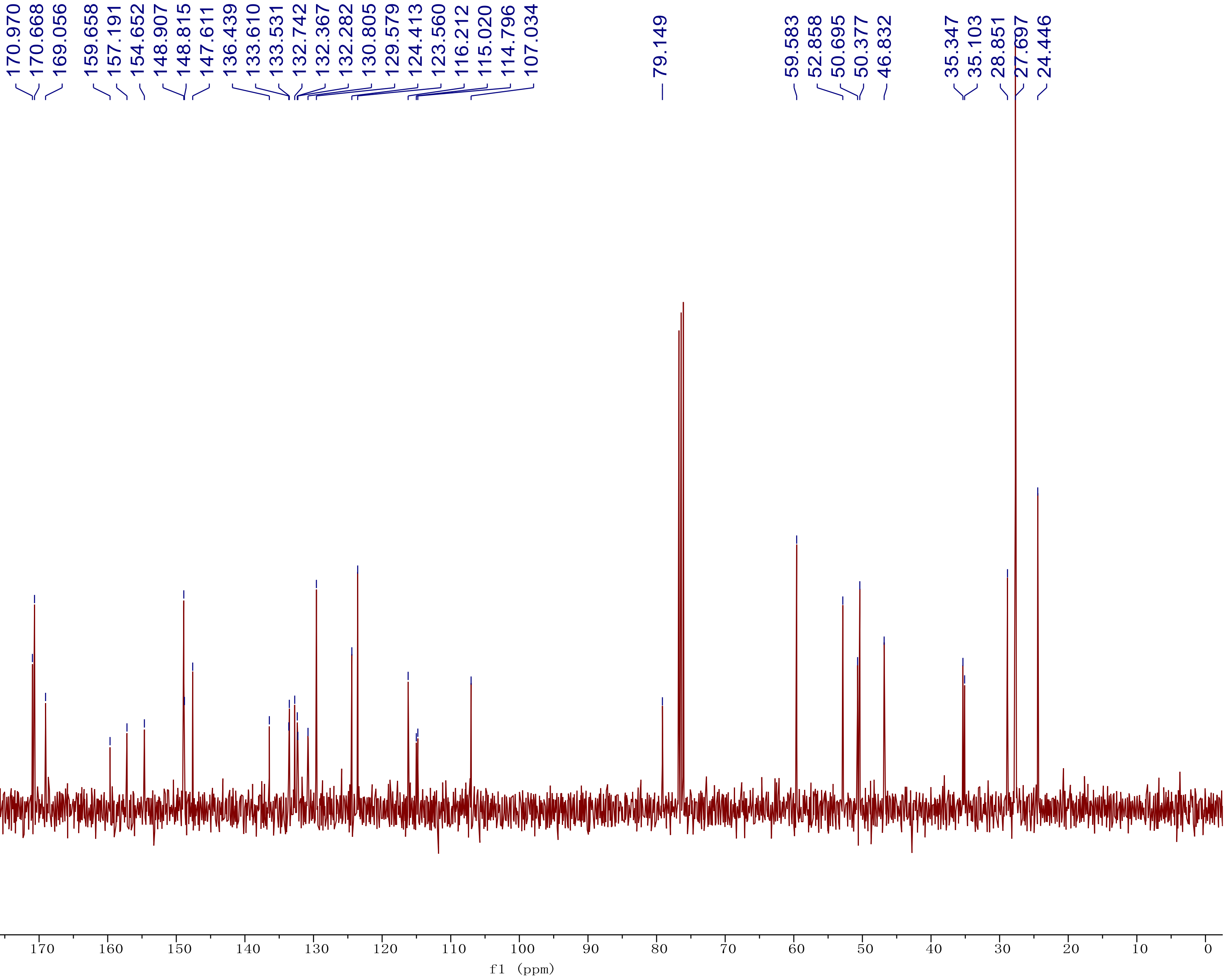

Compound 77 ^{19}F NMR (575 MHz, CDCl$_3$)

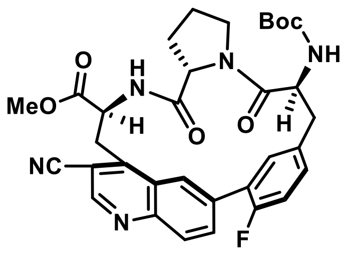

77

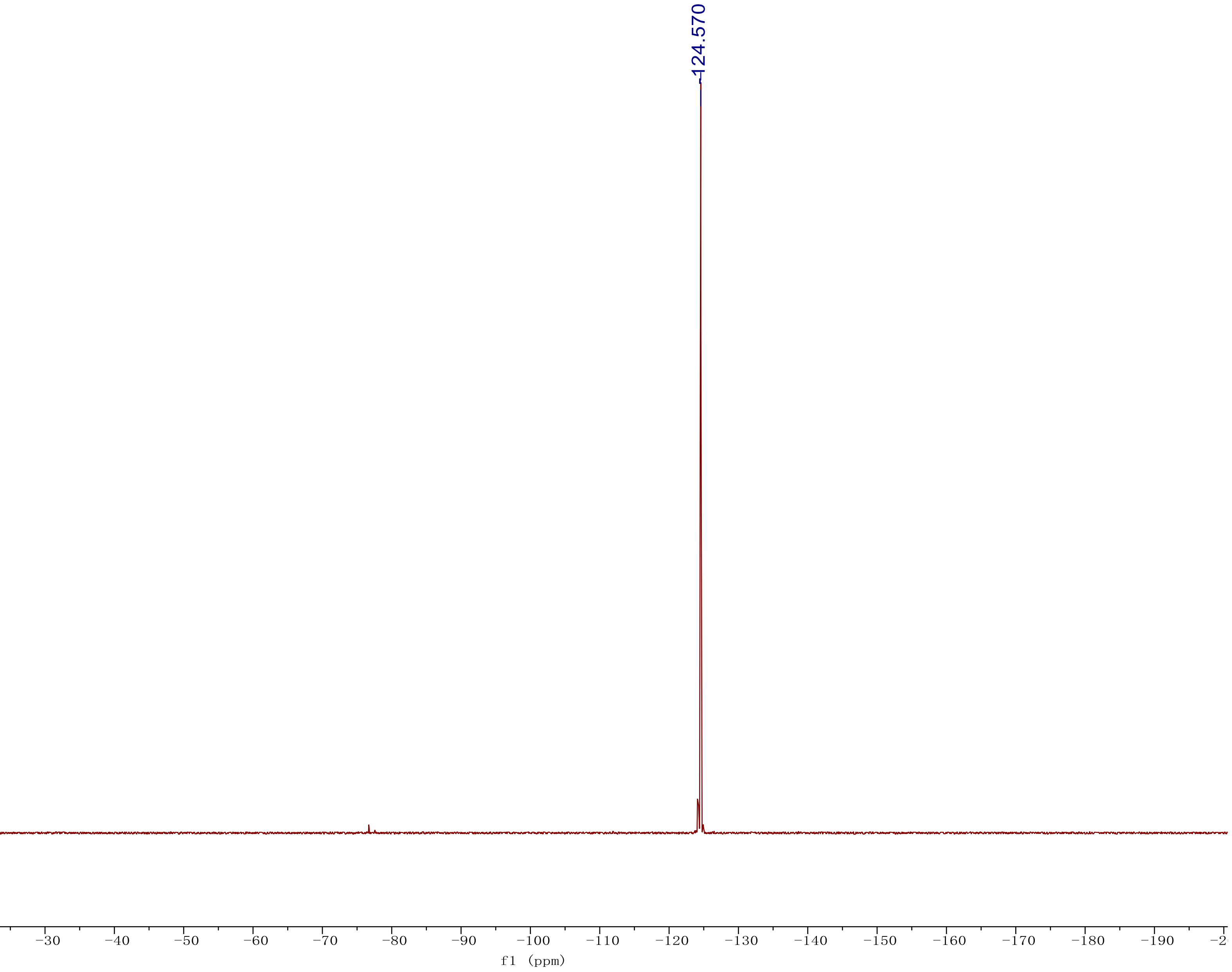

Compound 81 ^{1}H NMR (400 MHz, $CDCl_3$)

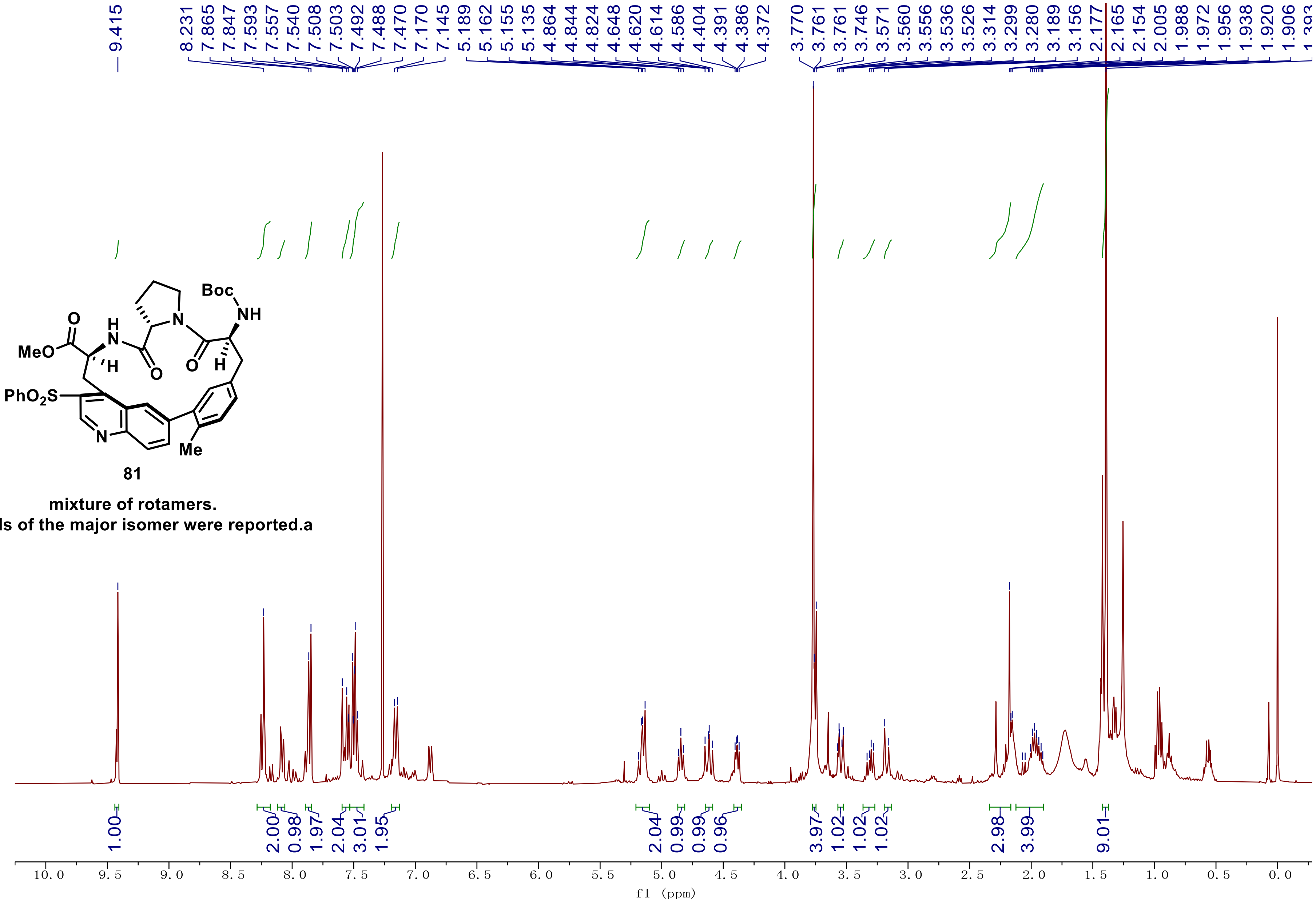

81

mixture of rotamers.
signals of the major isomer were reported.a

Compound 81 ^{13}C NMR (101 MHz, $CDCl_3$)

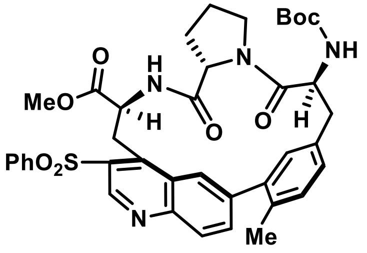

81

mixture of rotamers.
signals of the major isomer were reported.a

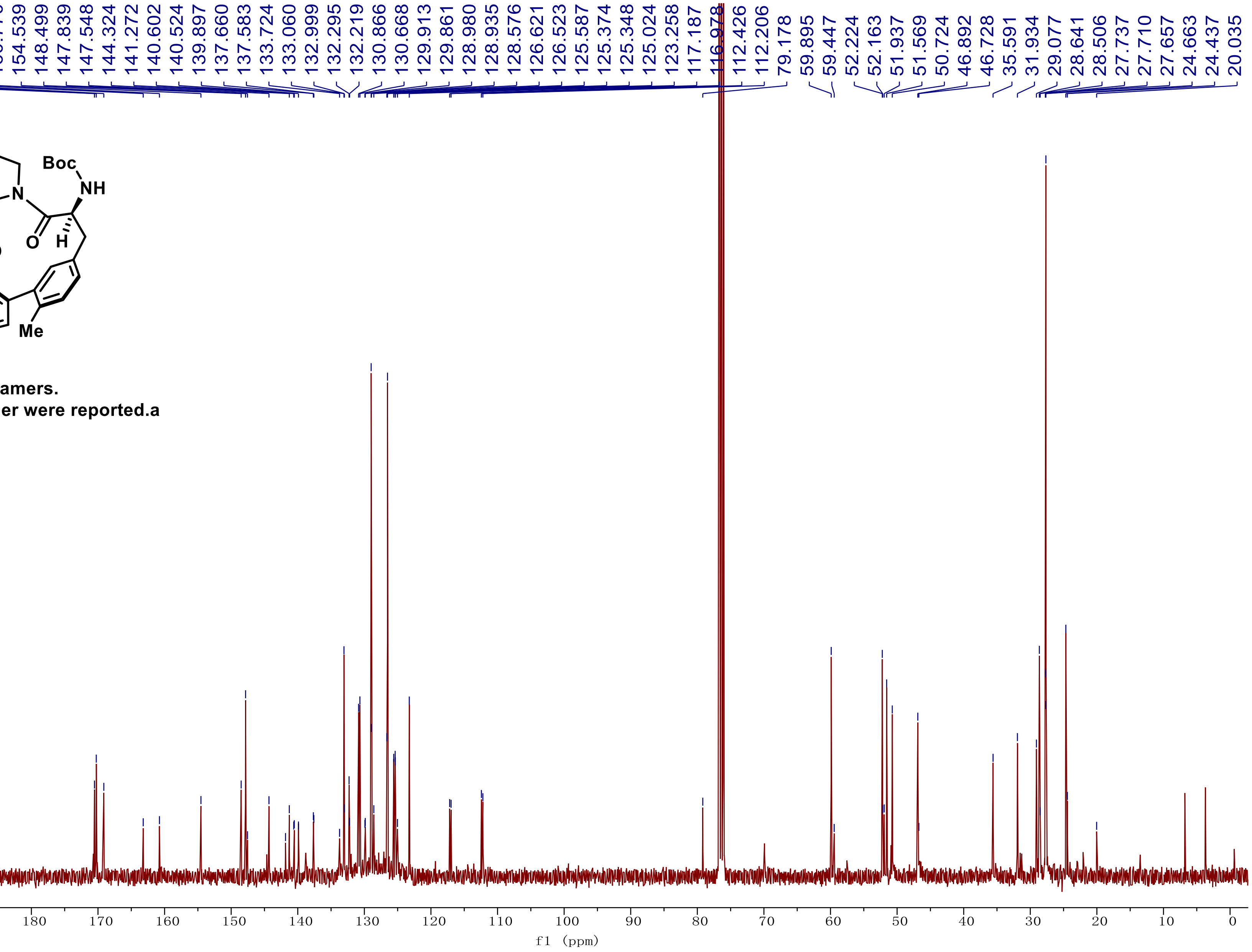

Compound 82 ^{1}H NMR (600 MHz, CD_3OD)

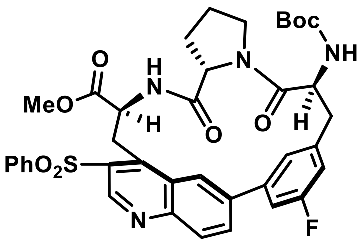

82

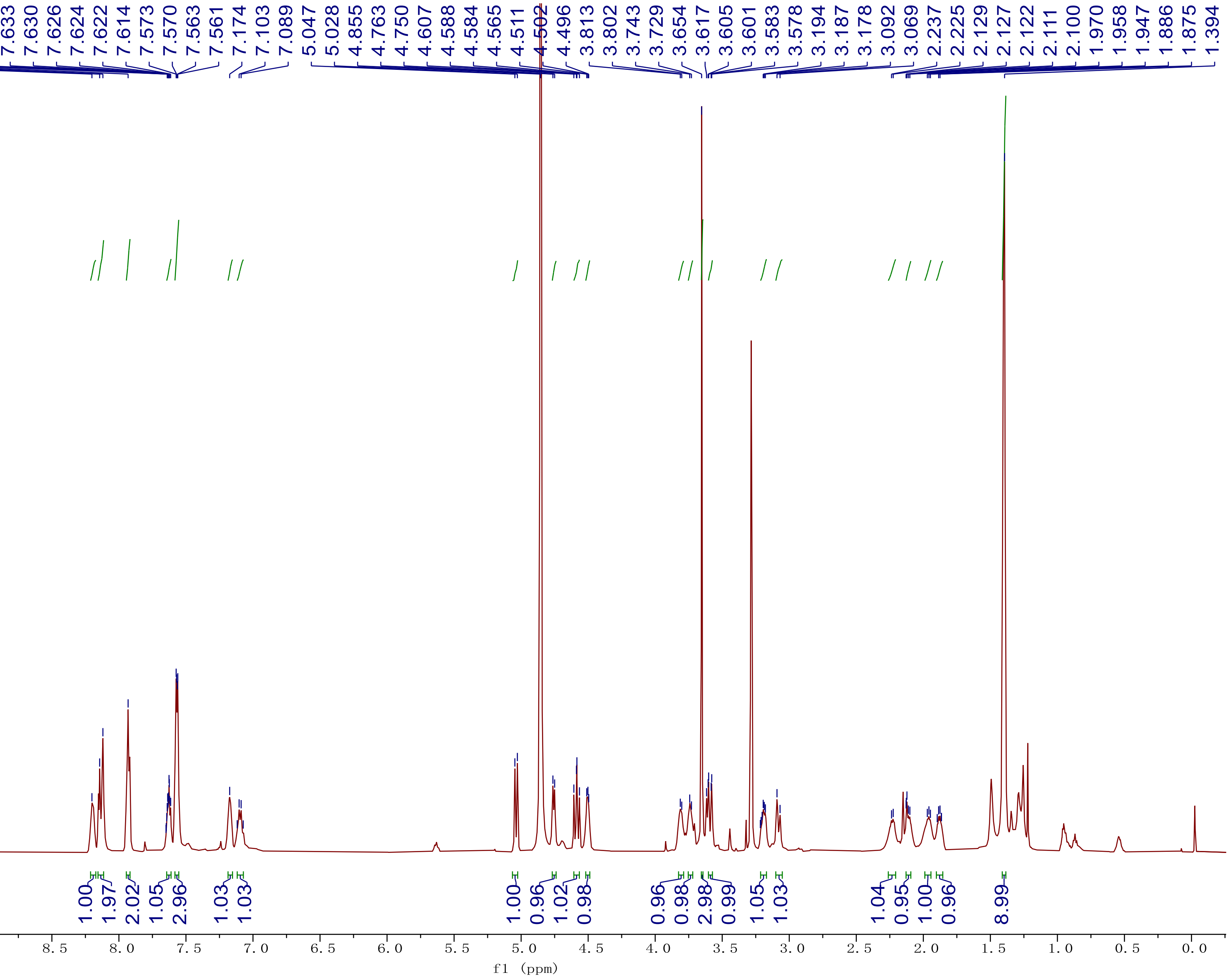

Compound 82 ^{13}C NMR (151 MHz, CD_3OD)

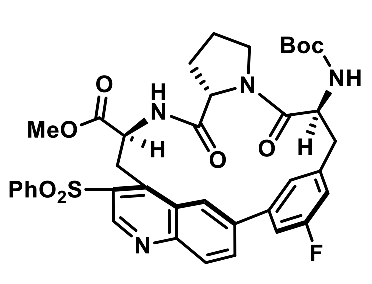

82

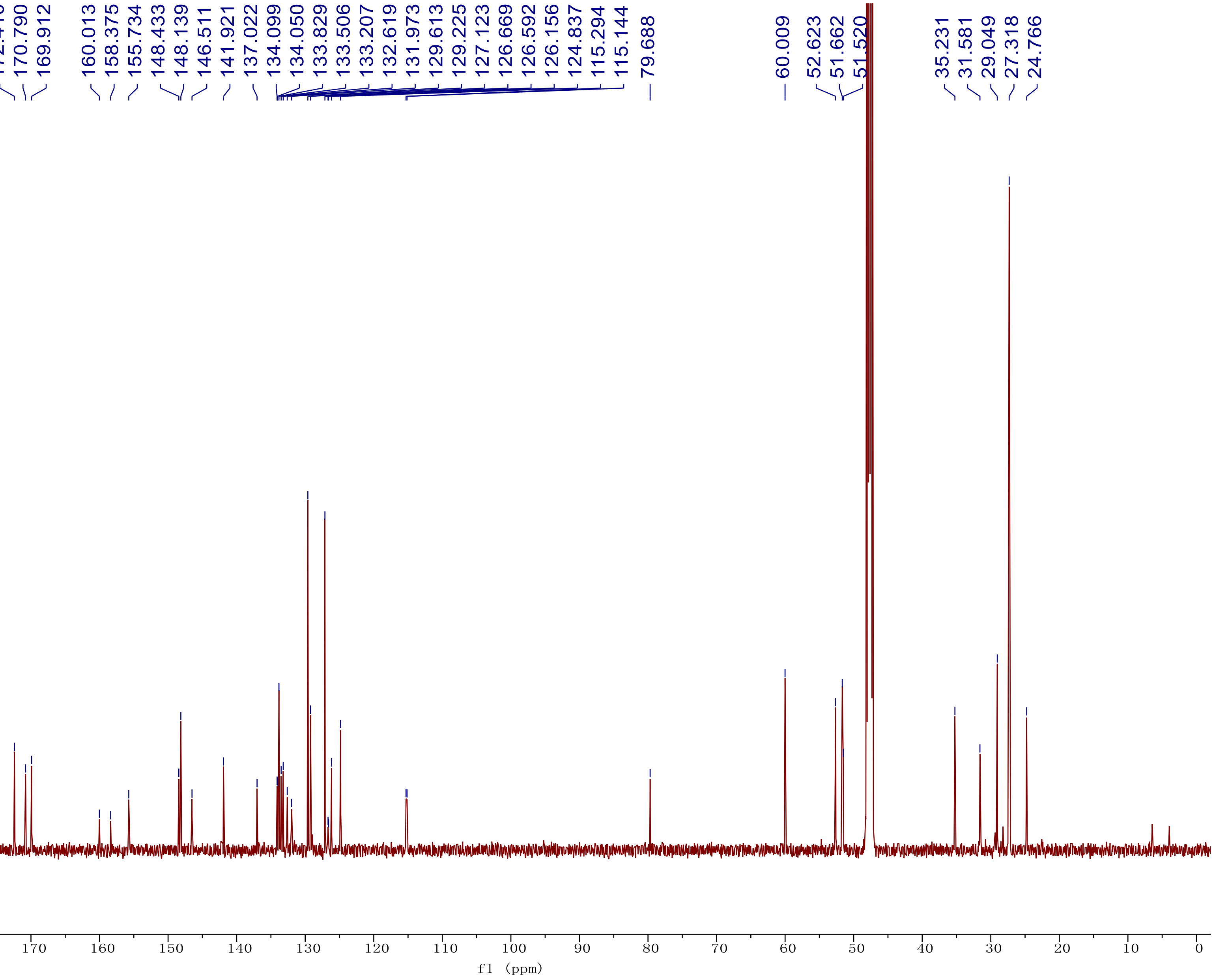

Compound 82 ^{19}F NMR (575 MHz, CD_3OD)

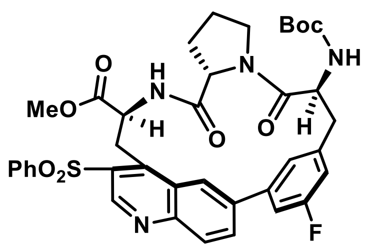

82

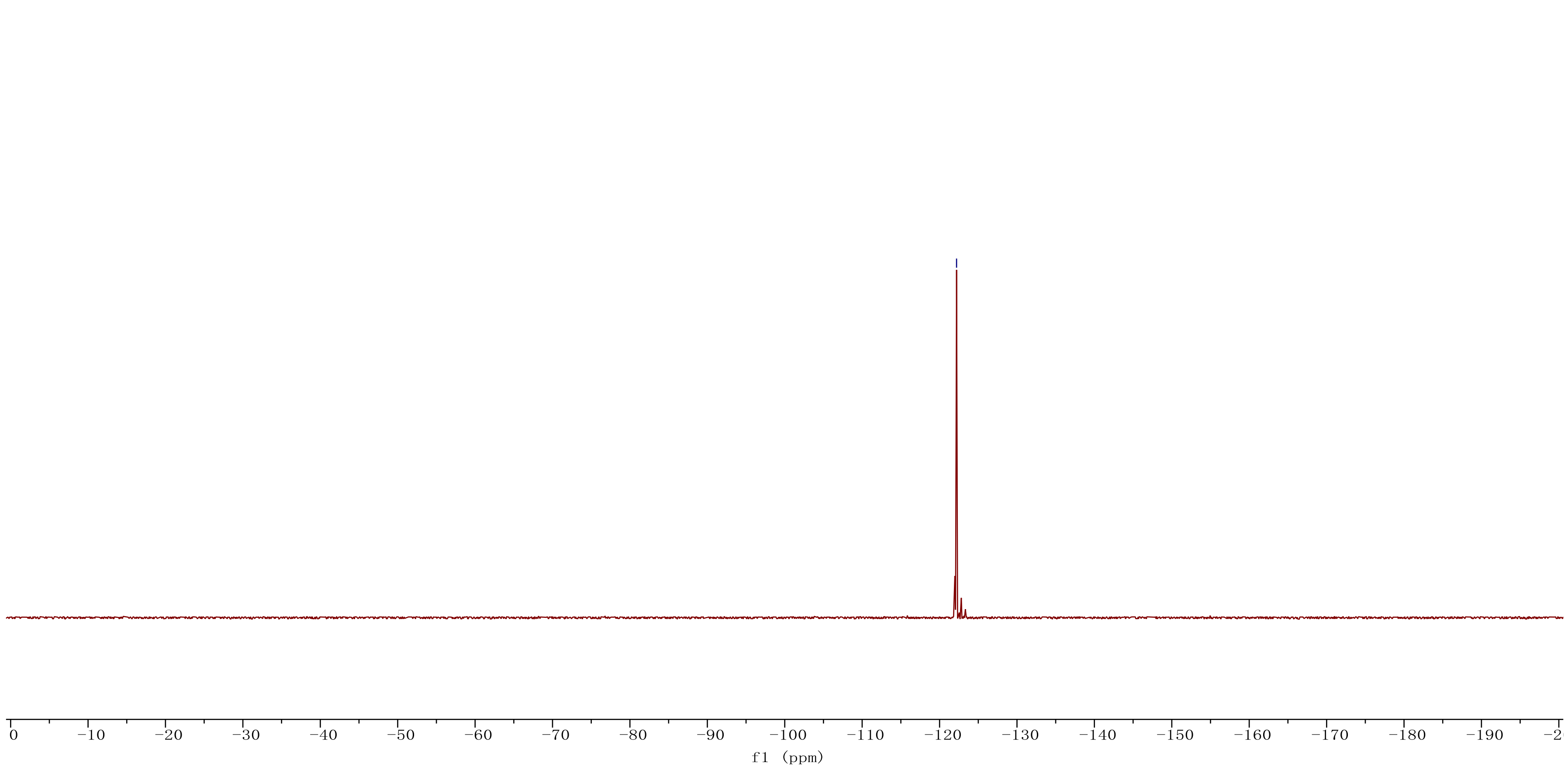

Compound 83 ^{1}H NMR(400 MHz, CD_3OD)

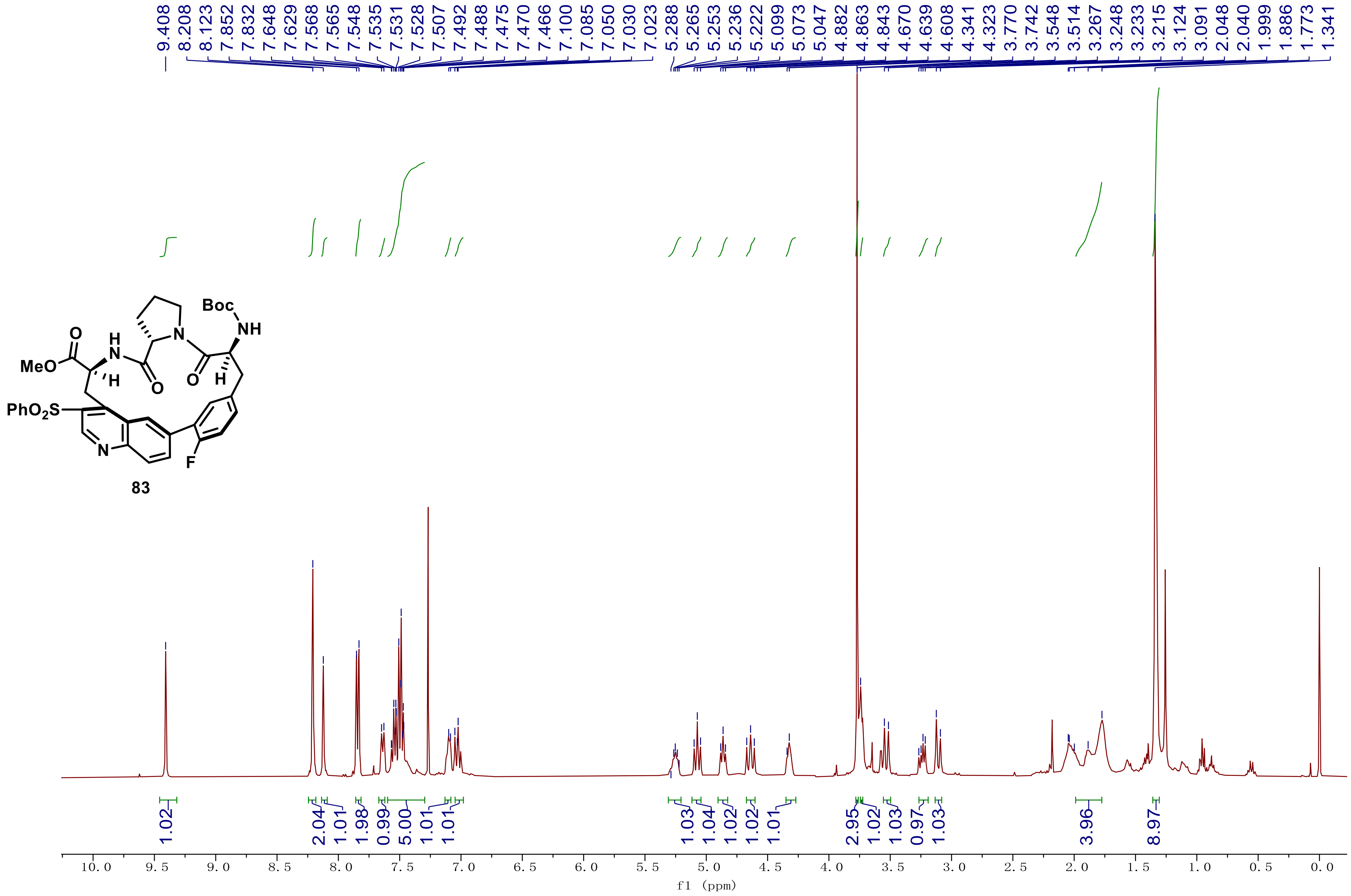

Compound 83 ^{13}C NMR (101 MHz, $CDCl_3$)

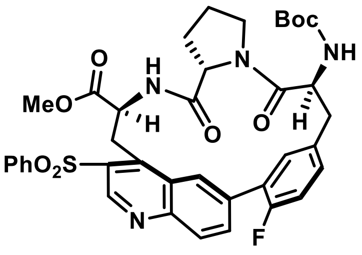

83

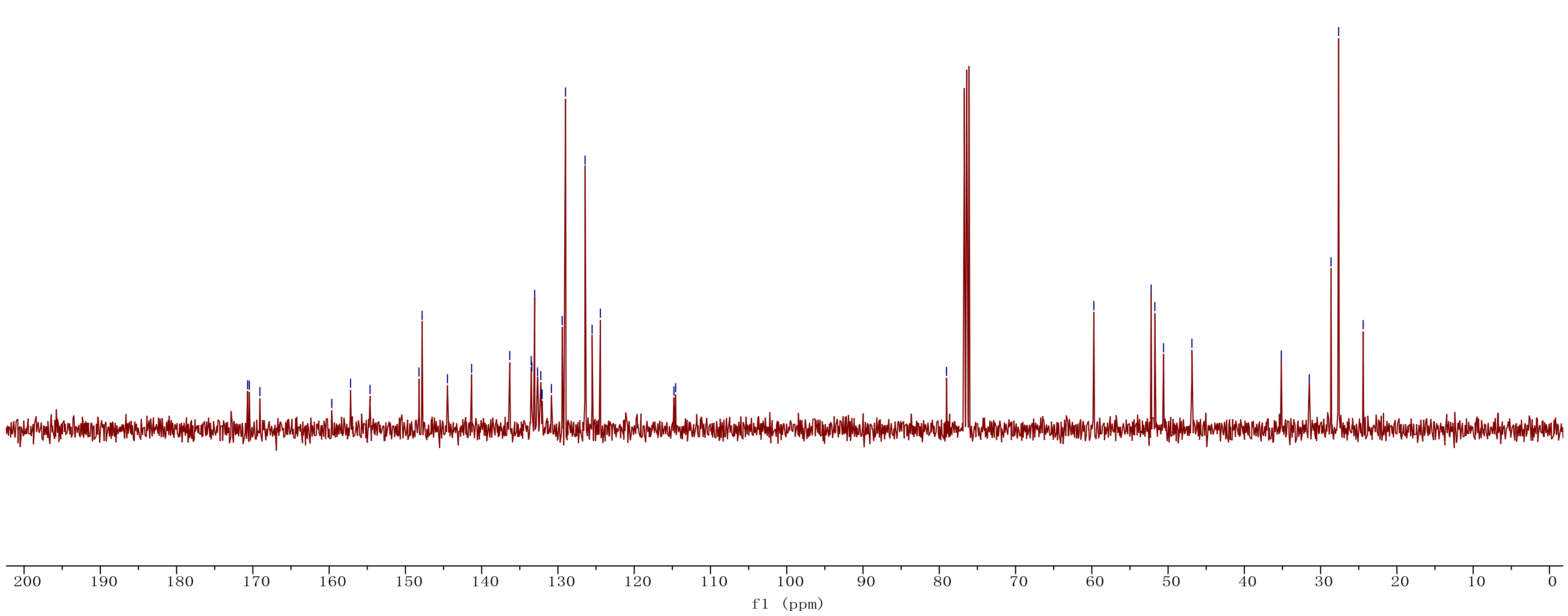

Compound 83 ^{19}F NMR (376 MHz, $CDCl_3$)

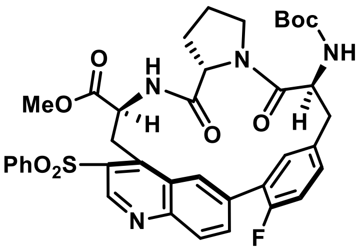

83

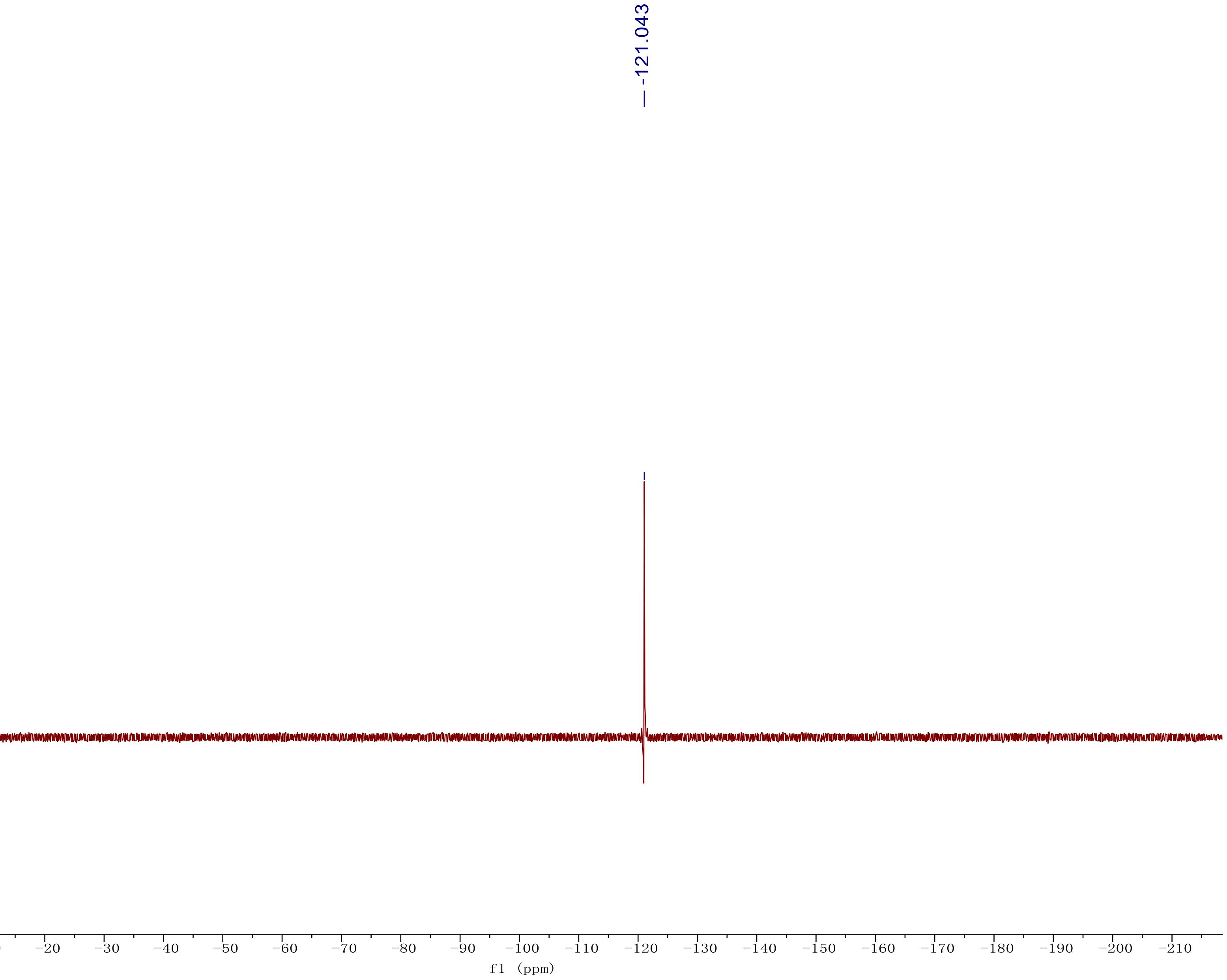

Compound S28 ^{1}H NMR (400 MHz, $CDCl_3$)

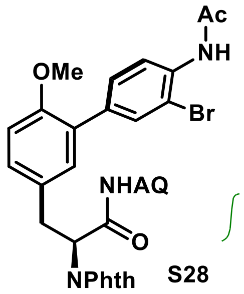

S28

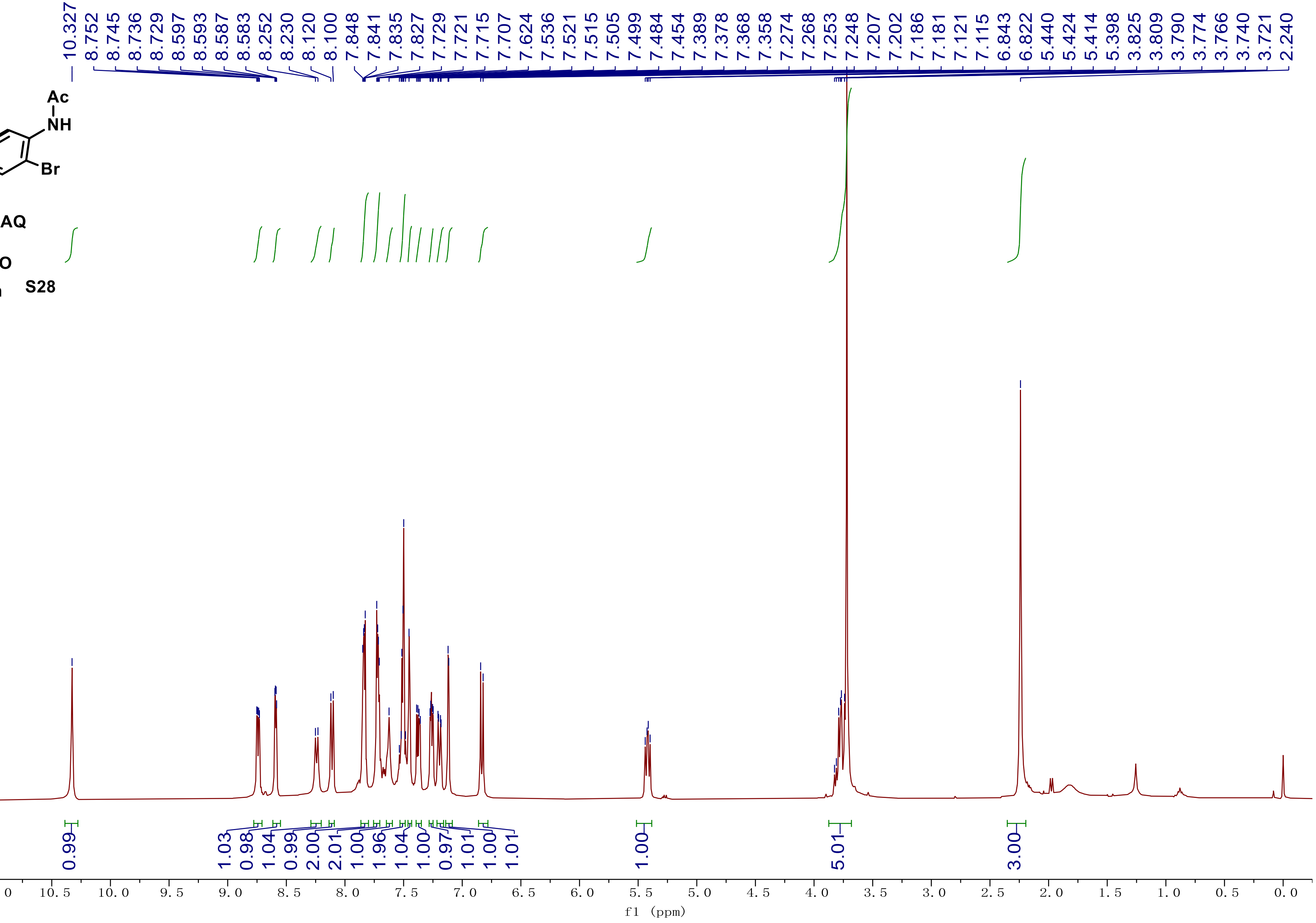

Compound S28 ^{13}C NMR (101 MHz, $CDCl_3$)

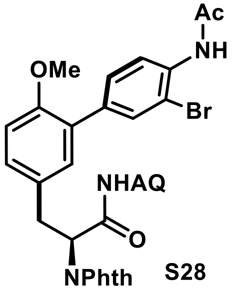

S28

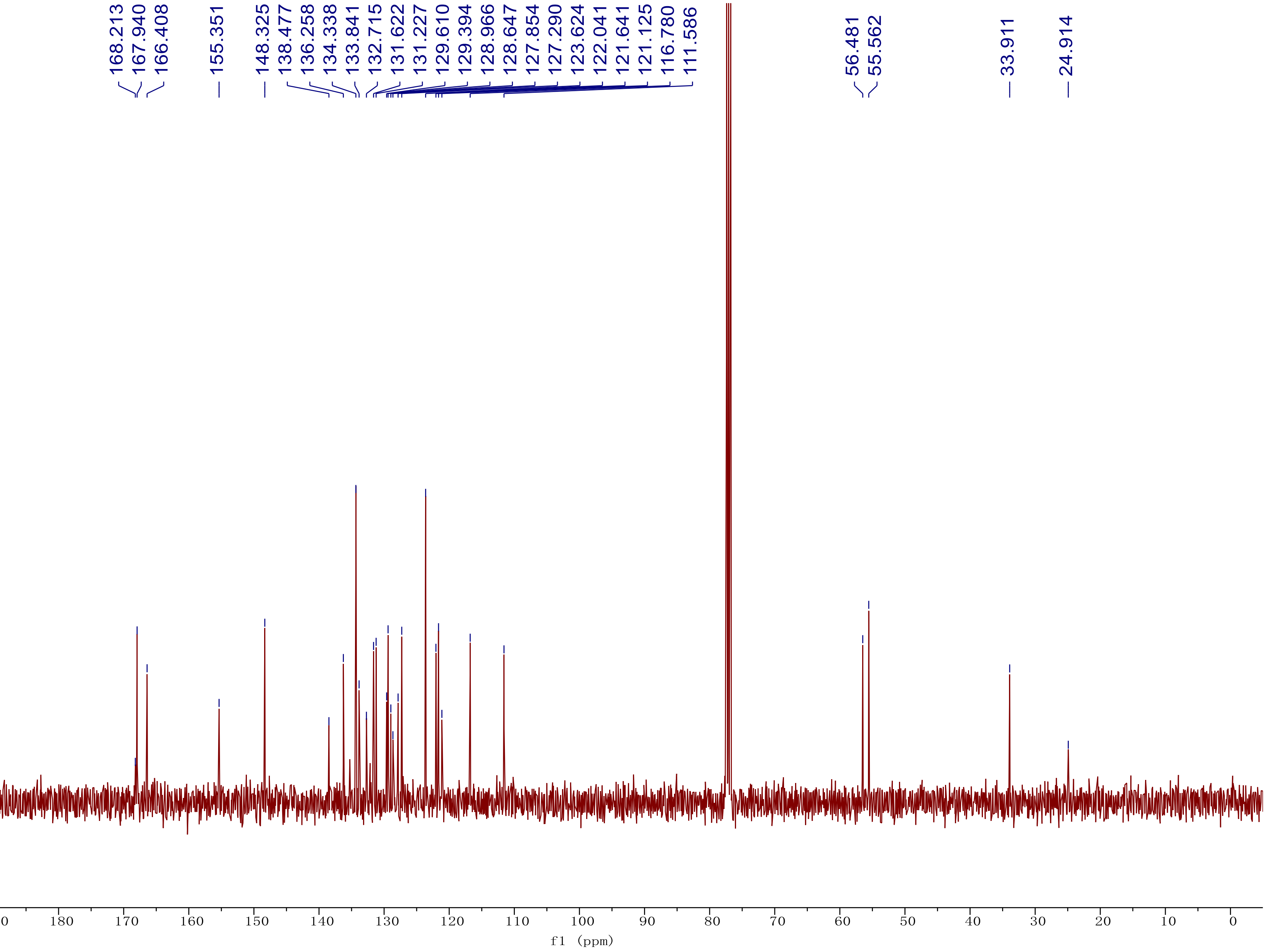

Supplement: SC-017-D6SC03088B-s001 [file SC-017-D6SC03088B-s001.pdf]
